# Development of Rapidly Evolving Intron Markers to Estimate Multilocus Species Trees of Rodents

**DOI:** 10.1371/journal.pone.0096032

**Published:** 2014-05-07

**Authors:** Ana Rodríguez-Prieto, Javier Igea, Jose Castresana

**Affiliations:** 1 Institut de Biologia Evolutiva (CSIC-Universitat Pompeu Fabra), Barcelona, Spain; 2 Imperial College London, Silwood Park Campus, Ascot, Berkshire, United Kingdom; University of Münster, Germany

## Abstract

One of the major challenges in the analysis of closely related species, speciation and phylogeography is the identification of variable sequence markers that allow the determination of genealogical relationships in multiple genomic regions using coalescent and species tree approaches. Rodent species represent nearly half of the mammalian diversity, but so far no systematic study has been carried out to detect suitable informative markers for this group. Here, we used a bioinformatic pipeline to extract intron sequences from rodent genomes available in databases and applied a series of filters that allowed the identification of 208 introns that adequately fulfilled several criteria for these studies. The main required characteristics of the introns were that they had the maximum possible mutation rates, that they were part of single-copy genes, that they had an appropriate sequence length for amplification, and that they were flanked by exons with suitable regions for primer design. In addition, in order to determine the validity of this approach, we chose ten of these introns for primer design and tested them in a panel of eleven rodent species belonging to different representative families. We show that all these introns can be amplified in the majority of species and that, overall, 79% of the amplifications worked with minimum optimization of the annealing temperature. In addition, we confirmed for a pair of sister species the relatively high level of sequence divergence of these introns. Therefore, we provide here a set of adequate intron markers that can be applied to different species of Rodentia for their use in studies that require significant sequence variability.

## Introduction

In the last few years, studies of closely related species, speciation and phylogeography have greatly benefited from the use of multiple sequence markers and their analysis with methods that take coalescent theory into account [1]–[3]. Coalescent theory predicts that different unlinked loci will have different genealogical histories as a result of the stochastic process of coalescence of alleles due to random genetic drift. In addition, conflicting gene trees are more likely to arise in the analyses of closely related species, where intra-specific variability represents a significant proportion of the tree branch lengths [4]. As a consequence, a single gene may give rise to a tree that differs from the true species or population tree. Currently, different approaches can be used to reconstruct the species tree of a set of species by using multiple loci and taking coalescent theory into account. These species tree approaches also provide information about hybridization and demographic changes along the tree [5], [6]. In addition, species tree approaches produce more accurate estimations of speciation times than gene trees, which is particularly noticeable for shallow phylogenies [4]. However, one of the major challenges of these studies is to have markers that show enough variability to be informative for the reconstruction of the species tree. Introns are in principle an ideal type of marker for such purposes due to their high phylogenetic information content [7]–[9] but they have not been routinely used in most taxonomic groups at shallow levels. Although exons have been successfully used in higher-level phylogenies, their lower sequence variability makes them less adequate than introns for the reconstruction of species trees of closely related species [4].

As indicated above, the main feature that makes introns attractive for these studies is their high evolutionary rate relative to exons [10]. In this sense, anonymous, intergenic regions also have fast evolutionary rates [11]–[13]. However, the advantage of introns is that they are flanked by exons, which are very convenient for placing conserved primers that may function across a wide range of species. This approach has led some to refer to introns as EPIC (exon-primed, intron-crossing) markers [7], [14], [15].

Currently, there are many methods that exploit sequence information in coalescent and species tree frameworks [16]–[19] into which intron sequences can be naturally integrated. Thus, introns have been successfully used in various studies including the reconstruction of well-resolved species trees [20], the estimation of more accurate speciation times [21], the analysis of dispersal patterns [22], and the testing of different speciation scenarios [23]. Introns can also be helpful to corroborate inferences from mitochondrial data, for example, to analyze variation in genetic diversity among populations [24], [25].

Despite the obvious advantages of introns, few studies have been directed to the systematic development of intron markers for their use with populations or closely related species [9], [26], [27]. Most efforts have been directed towards the development of exons [28], [29], which are mainly useful for higher-level relationships. Therefore, there is a critical need to develop rapidly evolving intron markers that may function at lower taxonomic levels [10].

Rodents comprise around half of the mammalian species [30]. Despite the importance of this group, no attempt has been made so far to develop a set of variable sequence markers for them. A previous analysis of mammalian genomes performed to develop intron markers [9] excluded rodents from the analysis because they have genomic features that would have made the comparisons with the rest of mammals problematic. For example, species of Rodentia and, particularly, of Murinae, have very attenuated isochores and show very fast evolutionary rates compared to other mammals [31]–[34]. Here, we applied a bioinformatic pipeline similar to the one used for the development of introns in non-rodent mammals [9]. This pipeline consists of the analysis of the available rodent genomes in databases to extract orthologous introns with several characteristics that make them adequate for their amplification by PCR, including a length of a few hundred base pairs and being single copy. In addition, it is known that some introns are highly conserved due to selective pressures [35] and therefore, to avoid including conserved introns that would not be useful for shallow phylogenies, we applied specific filters that select introns with high evolutionary rates as estimated from phylogenetic trees reconstructed for each intron. These filters are necessary for getting the best possible markers from genomes when sequence variability is a crucial factor. Finally, we carried out an experimental study of ten of the introns developed here in a panel of 11 species belonging to different representative rodent families [36], [37]. The high success rate obtained in the 110 PCR amplifications performed indicates that these introns can be amplified in a wide variety of rodent groups. We also analyze the variability of these introns in relation to their usefulness in multilocus analyses of closely related species.

## Materials and Methods

### Ethics Statement

All tissue samples were obtained according to relevant national and international guidelines. Tissue samples of *Octodontomys gliroides*, *Atherurus macrourus* and *Proechimys guairae* were obtained from the Ambrose Monell Cryo Collection (AMCC) at the American Museum of Natural History (AMNH). Samples of *Myocastor coypus*, *Hydrochoerus hydrochaeris* and *Cynomys ludovicianus* were from deceased animals from a zoo and were obtained from the Banco de Tejidos Animales de Cataluña (BTAC). The sample of *Microtus duodecimcostatus* was obtained from a specimen captured with permit SF/069 given by the Departament d’Agricultura, Ramaderia, Pesca, Alimentació i Medi Natural of the Catalan Government. Tissue samples of *Sciurus vulgaris*, *Glis glis* and *Apodemus flavicollis* were obtained from a specimen of each species found dead in the field in public areas; according to relevant guidelines we are aware of, no permit is required to collect a small tissue sample from a specimen found dead and belonging to these species (listed as “Least concern” by the IUCN). The sample of *Microtus lusitanicus* was obtained from one of the authors of previous studies (JV) about this species [38], [39].

### Bioinformatic Pipeline

The genomes of three rodent species sequenced with high coverage and fully annotated were downloaded in GenBank format from the Ensembl database [40]. These genomes were: mouse (*Mus musculus*) version NCBIm37 [32], rat (*Rattus norvegicus*) version RGSC 3.4 [31], and guinea pig (*Cavia porcellus*) version cavPor3 [40]. We also downloaded the human genome (*Homo sapiens*) version GRch37 [41], [42], which we used for rooting the rodent trees. From these genomes, we extracted introns of manageable size (<50 000 bp) as well as the corresponding flanking exons. A series of custom-made Perl and UNIX scripts were specifically designed to extract these sequences and to automatize subsequent steps of the pipeline.

We next recovered from the rodent genomes the full lists of orthologous groups of genes using the BioMart database [43], which has information on orthologous relationships of genes between different species pairs. For this purpose, we first obtained the complete list of orthologous genes of the pair of closest related species in our set, mouse and rat. This table, using mouse as the reference, was then crossed with the genes of guinea pig to obtain an orthologous gene set of these rodent species. Finally, this set was compared with the human genes to obtain a set of putative one-to-one orthologous genes for the four mammalian species. As an additional filter, we eliminated all genes in which the number of exons was not identical in all four species. From this gene set, we took the introns and the flanking exons that had been previously extracted from the genome sequences.

We then selected introns with lengths of between 200 and 1600 bp in the mouse, which should be ideal for PCR amplification. In addition, we favored introns with low variability in length among species in order to avoid introns that could be difficult to align in subsequent analyses. For this purpose, we made a pairwise comparison of the intron length between the different species. Only introns were kept where the difference in length between human and any of the other species was lower than 90% of the human intron length (i.e. we only kept introns whose size was between 10% and 190% of the corresponding human intron length; for example, with a human reference intron of 1000 bp, we only kept rodent introns whose length was between 100 and 1900 bp). This allowed us to discard introns with a very large difference in length, that is, which were much shorter or much larger than the human intron. For closer species pairs, this criterion was more stringent: we only allowed a difference in length between guinea pig and the other species lower than 80%, and a difference in length between mouse and rat lower than 70%. At this point, we applied two quality filters, one to eliminate redundant introns (that were annotated more than once) and the other to eliminate introns with more than nine ambiguous bases in any species. An additional filter eliminated introns with either flanking exon shorter than 40 bp, to ensure that flanking exons were long enough for primer design.

To avoid multiple bands in the PCR reactions, all introns of genes with duplicated copies in the genome were eliminated. To do this, the flanking exons of every selected intron were used to search the genome of the respective species using Blast [44] and applying a threshold of 10^−4^ for the e-value.

In order to apply a series of phylogenetic filters, all introns of each orthologous group were aligned using Mafft version 6.708 [45]. The Gblocks program version 0.91 [46] was then used with relaxed parameters [47] to discard poorly aligned positions. From each alignment, a maximum-likelihood tree was constructed using RaxML version 7.0.4 with a GTR model of nucleotide substitution and a gamma distribution of evolutionary rates [48]. In addition, a maximum-likelihood tree was obtained, using the same method, from the concatenated alignments of all the introns present at this stage of the filtering process, to be used as a reference tree. Every single intron tree was then compared to the reference tree, and the scaling factor and the K tree score were calculated using the Ktreedist software [49]. The scaling factor indicates the relative overall rate of a particular tree with respect to a reference tree, and the K tree score reflects the topological and relative branch-length differences between a given tree and a reference tree (and therefore reveals anomalous variations in the molecular clock among branches in the give tree). Using these data, we selected introns as divergent as possible (scaling factor <0.8) and with small differences in molecular clock variation with respect to the reference tree (K tree score <0.09). After the sequential application of all these filters, the resulting intron markers were considered adequate for their amplification in different rodent species.

### Primer Design

From the final set of introns, we selected 35 markers with sequence lengths in mouse of between 300 and 500 bp. After a preliminary inspection of the intron alignments, and especially after taking into account the existence of appropriate regions in the exons for primer design, we kept 10 introns that we considered readily amplifiable. Primers were designed from the flanking exons of the three rodent species considered here plus additional sequences of rodents and lagomorphs downloaded from Ensembl. These species were the rodents *Dipodomys ordii* (version dipOrd1) of the family Heteromyidae and *Ictidomys tridecemlineatus* (speTri1) of the family Sciuridae, and the lagomorphs *Oryctolagus cuniculus* (OryCun2) of the family Leporidae and *Ochotona princeps* (OchPri2.0) of the family Ochotonidae. Some exons could not be recovered from these species due to low coverage and annotation quality of their respective genomes. All available sequences of the flanking exons of each intron were aligned as described above and the primers were designed in the most conserved regions. Degenerate nucleotides were used to accommodate sequence variation in the alignment. Primers were designed to have a low degeneration level (less than 48 combinations), to be between 20 and 25 bp in length, and to have a similar optimal annealing temperature between the primer pairs. These introns were then tested in a panel of 11 rodent species obtained from different sources and biological collections (Table S1) and selected to belong to different representative families [36], [37].

All tissues of the different rodent species were preserved in ethanol at −20°C. Genomic DNA was extracted from tissue (around 25 mg) using the DNeasy Blood & Tissue kit (Qiagen) following the manufacturer’s instructions, and it was eluted in a final volume of 50 µl of water. PCR was performed in 25 µl reactions containing 50–100 ng of DNA, 1 µM of each primer, 0.2 mM dNTPs, 0.75 units of Promega GoTaq DNA polymerase and 17.5 µg of bovine serum albumin. PCR was carried out using a “touch-down” procedure [50] with the following conditions: an initial denaturation of 3 min at 95°C, followed by a first set of 15 cycles of denaturation (30 s at 95°C), annealing (30 s starting at 65°C) and extension (30 s at 72°C). In each successive cycle the annealing temperature was gradually reduced until it reached the annealing temperature of the second set of cycles, which was one of 60, 55 or 50°C. The conditions of the second set of cycles (with 20 additional cycles) were otherwise as those of the initial set. A final extension of 10 min at 72°C was added. PCR products were revealed by electrophoresis in a 1% agarose SYBR-Safe (Invitrogen) stained gel.

All DNA extractions from the different species were tested for each intron, first with a touch-down PCR with an annealing temperature ramp of 65-55°C. If products contained multiple bands, a more specific PCR with an annealing temperature ramp of 65-60°C was attempted. Conversely, if there was no amplification, another PCR was attempted with an annealing temperature ramp of 65-50°C.

Successful PCR products were purified with ExoSAP-It (Affymetrix) and sequenced in both directions using the original PCR primers at Macrogen Inc. (Seoul, South Korea). Sequences were inspected, trimmed and assembled using Geneious Pro (Biomatters Ltd.).

All sequences obtained in this study were deposited in GenBank under accession numbers KF129522–KF129599 and KF916621–KF916629 (Table S2).

### Phylogenetic Analysis

The newly obtained intron sequences were added to the sequences of mouse, rat, guinea pig and human, and were aligned using Mafft [51]. Regions of ambiguous alignment were cleaned with Gblocks using relaxed parameters [46], [47]. Maximum-likelihood phylogenetic trees of each intron alignment were reconstructed with RaxML as described above [48].

In addition, a maximum-likelihood phylogenetic tree was reconstructed from all concatenated introns. A Bayesian approach was also applied to reconstruct a phylogenetic tree with BEAST version 1.61 [52] including the ten intron alignments as independent partitions. The most appropriate substitution model was set as suggested by jModeltest [53] (Table S3). In order to set the most appropriate molecular clock (strict or relaxed uncorrelated lognormal), a test was performed with PAUP* version 4.0b10 [54] by estimating the likelihood of the PhyML topology with and without forcing a molecular clock and applying a likelihood-ratio test [55] (Table S3). A Yule speciation model was used as tree prior and the human sequence was used to root the tree. The analysis was run for 50 million generations, sampling every 1000 generations, and 10% of the samples were discarded as burn-in. Convergence was checked with the BEAST utility Tracer to ensure that all effective sample size values were greater than 200. We computed the corresponding maximum clade credibility tree with median node heights using the BEAST utility TreeAnnotator.

The SH test [56] implemented in RaxML [48] was used to compare the maximum-likelihood phylogenetic trees obtained from each individual intron with alternative topologies. As alternative topologies the maximum-likelihood and Bayesian trees obtained from all intron sequences were used.

Alignments and trees of the 10 tested introns have been deposited in TreeBASE under accession number S15064 (http://purl.org/phylo/treebase/phylows/study/TB2:S15064).

## Results

### Development of Intron Markers from the Comparison of Rodent Genomes

**Figure 1:**
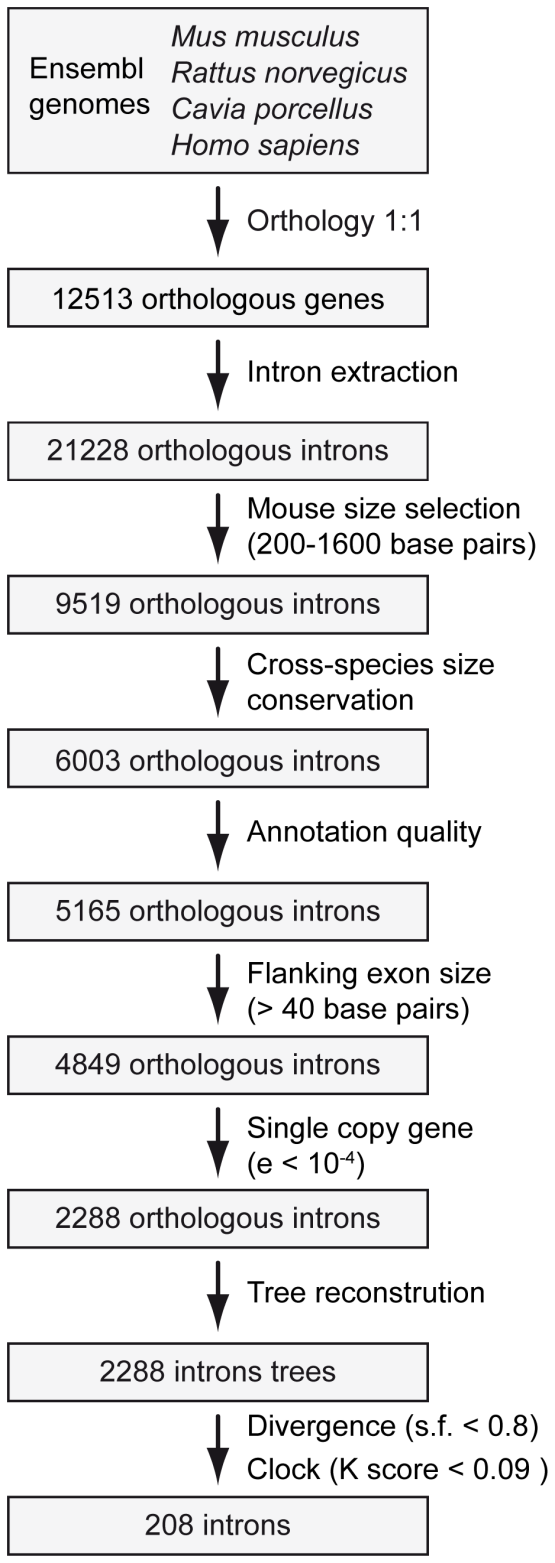
Scheme of the bioinformatic pipeline. The main steps followed for the intron extraction and filtering processes and the number of introns remaining in each step are shown.

After crossing the orthology information of the mouse, rat, guinea pig and human genomes, we obtained 12 513 putative one-to-one orthologous genes (Figure 1). From these genes, we extracted 21 228 introns together with their flanking exons. We then selected introns between 200 and 1600 bp in mouse, resulting in a set of 9519 introns. After the application of an additional length filter that removed introns with large differences in length between species, 6003 introns remained. Passing these introns through two further filters that eliminated introns with redundant annotations or with low sequence quality (many ambiguous bases) resulted in 5165 introns remaining. We also removed introns with short flanking exons (<40 bp), which resulted in a set of 4849 introns. The next filter was applied to eliminate introns belonging to genes with multiple copies. For this purpose, Blast searches were performed with the adjacent exons against the corresponding genomes (the use of exons rather than introns allowed a much greater sensitivity). This led to the elimination of more than half of the remaining introns, leaving 2288 introns that we used for various phylogenetic tests.

Phylogenetic trees of the alignments of individual introns were compared with a reference tree obtained from the concatenation of the 2288 introns (Figure S1) in order to extract information about branch lengths and global divergence features of each intron. We first estimated the overall divergence of each tree as the scaling factor that needs to be applied to the branches of the individual tree to minimize length differences from the reference tree [49]. The scaling factor ranged between 0.064 and 4.936 in the intron trees (Figure S2). All values smaller than 1 correspond to trees that are more divergent than the reference tree. We therefore retained trees with a scaling factor smaller than an arbitrarily-selected 0.8 threshold. This resulted in discarding introns that might be too conserved to accumulate variability within a species or between closely related species. As indicated, the scaling factor should reflect the overall divergence of a tree [49]. To test this, we also took the sum of branch lengths in the individual trees. This variable was indeed highly correlated with the scaling factor (r = −0.84; p<0.0001); therefore a filter based on this sum would have rendered similar results and was not utilized. We also calculated the sum of the branch lengths of the two Murinae species (mouse and rat) as a measure of more recent divergences in this part of the tree. As expected, this variable showed a smaller correlation with the scaling factor (r = −0.29; p<0.0001). A parameter of this type may help to select better optimized introns for Murinae species, but it was not applied in this study which instead sought introns of general validity for rodents.

In addition, we calculated the K tree score, which is indicative of trees that have some highly accelerated or decelerated branches with respect to the reference tree, regardless of the overall tree divergence [49]. This value ranged between 0.012 and 0.467 (Figure S2). Trees with high K tree scores may have very different evolutionary rates in different rodent groups, making it difficult to predict whether they have a high rate in all species. These introns may also cause difficulties in the estimation of evolutionary rates, even with the application of relaxed clocks [57]. Therefore, we set an arbitrarily chosen threshold of 0.09 and retained those intron trees with smaller values.

After applying these filters, 208 introns remained in our final data set. We elaborated a catalog of these introns together with relevant information for each marker (Appendix S1 and S2). This information included the alignments of both the intron and the flanking exons, the function of the gene to which the intron belongs, the genomic location in mouse, several parameters about the divergence of the intron (K tree score, scaling factor and total Murinae branch length), and the phylogenetic tree of the four orthologous intron sequences. The examination of this information will allow the selection of optimal markers for specific studies and the design of exon primers with different degrees of specificity.

### Experimental Validation of the Newly Developed Intron Markers

In order to test if these introns could be amplified as expected in different rodent species, we selected 10 introns and designed primers from the exon alignments after including, where possible, more species from genomic databases (Table 1). These primers were tested in 11 species selected from across different representative rodent families [36], [37] (Table S1). Out of the 110 PCR amplifications performed with an initial temperature ramp of the touch-down PCR at 65-55°C, we obtained a single, clear PCR band in 80 reactions (Table 2). In reactions with multiple bands, a PCR with a more specific annealing temperature ramp at 65-60°C was performed, resulting in two additional successful, unique bands. For reactions with no band obtained from the first attempt, the PCR was repeated with the annealing temperature ramp at 65-50°C, and five additional bands were obtained. Therefore, we obtained 87 positive reactions and an overall success rate of 79% (Table 2). However, not all introns were amplified similarly across the rodent lineages. All introns were amplified successfully in at least some species but only the intron Dhcr24–7 was amplified in all species. The least successful introns were Rras-4 and Trpv4–8, which worked in six species. Regarding the species, none were successfully amplified for all introns and the success rate varied between 30 and 90% of the PCR reactions.

**Table 1:** Introns used in this work for amplification in different rodent species.

Intronname	Ensembl code	Intron length(mouse)	Primers sequences (Forward/Reverse)
Abcb9-2	ENSMUSG00000029408	423	GCATYGTSATCCAGAARAGCAYGGA/CTGTGCGRTTCTCRTCRAARAAGCT
Agxt-10	ENSMUSG00000026272	420	GGCTACAACTGGAGGGACATC/TGCAGGGCCTCCYTCAGGGCCT
Catsper3-5	ENSMUSG00000021499	382	TGCTKGCMTCSTTCATCTT/AGRATYAYYTGCTTCTYCTCC
Dhcr24-7	ENSMUSG00000034926	357	CAGGACATGCTGGTGCCCATGAA/CCTGGCTGGCTGGGCAGGATGAA
Ivd-8	ENSMUSG00000027332	478	CTGGACCTRGARCGCCTGGT/CTGRAAKTGSCCRATYTTCT
Nadsyn1-4	ENSMUSG00000031090	498	GTYCGYTACAAYTGCAGAGT/TCCTKSHCCAKGGGGTRAACCA
Rras-4	ENSMUSG00000038387	488	ACWCAGATCCTCMGRGTYAAGGA/AGTTTGGCDGAKGCCTCRAAGTA
Smo-9	ENSMUSG00000001761	344	GCCACCCTGCTCATCTGGAGGCG/TTGGCRATCATCTTGCTYTTCTTGA
Trpv4-8	ENSMUSG00000014158	398	TTACCRBACCACVGYGGACTACCT/CTGGAAGGAGCCRTCGAYGAAGA
Wls-7	ENSMUSG00000028173	364	AAYCACATYGCMGGSTAYTGGAA/TCYGTKCCAACRTCYGTRGTCCA

The intron name (gene name followed by the intron number), Ensembl code, the length in mouse and the designed primers are shown.

**Table 2:** Introns amplified in this work and results obtained in 11 different species.

	Abcb9-2	Agxt-10	Catsper3–5	Dhcr24-7	Ivd-8	Nadsyn1–4	Rras-4	Smo-9	Trpv4–8	Wls-7
*Octodontomys gliroides*	65-55	65-55	65-55	65-55	65-55	65-55	–	65-55	65-55	65-55
*Atherurus macrourus*	65-55	65-60	65-55	65-55	65-55	65-55	–	65-55	65-55	65-55
*Proechimys guairae*	65-55	65-55	–	65-55	65-55	65-55	–	–	–	65-55
*Myocastor coypus*	65-55	–	–	65-55	–	65-55	–	–	–	–
*Hydrochoerus hydrochaeris*	65-55	65-55	65-55	65-55	65-55	–	65-55	65-55	65-55	65-55
*Cynomys ludovicianus*	65-55	–	65-55	65-60	65-55	65-55	65-55	65-55	65-55	65-55
*Sciurus vulgaris*	65-55	–	65-55	65-55	65-55	65-55	65-55	65-55	65-55	65-55
*Glis glis*	–	–	65-55	65-55	65-55	65-55	–	65-55	–	65-55
*Apodemus flavicollis*	65-55	65-55	–	65-55	65-55	65-55	65-55	65-55	65-55	65-55
*Microtus duodecimcostatus*	65-55	65-55	65-55	65-55	65-55	65-55	65-55	65-55	–	65-55
*Microtus lusitanicus*	65-50	65-50	65-50	65-55	65-55	65-50	65-50	65-55	–	65-55

When the amplification was successful, the temperature ramp of the touch-down PCR is indicated, with the optimized temperature ramp underlined.

### Phylogenetic Analysis of the Amplified Intron Sequences

**Figure 2:**
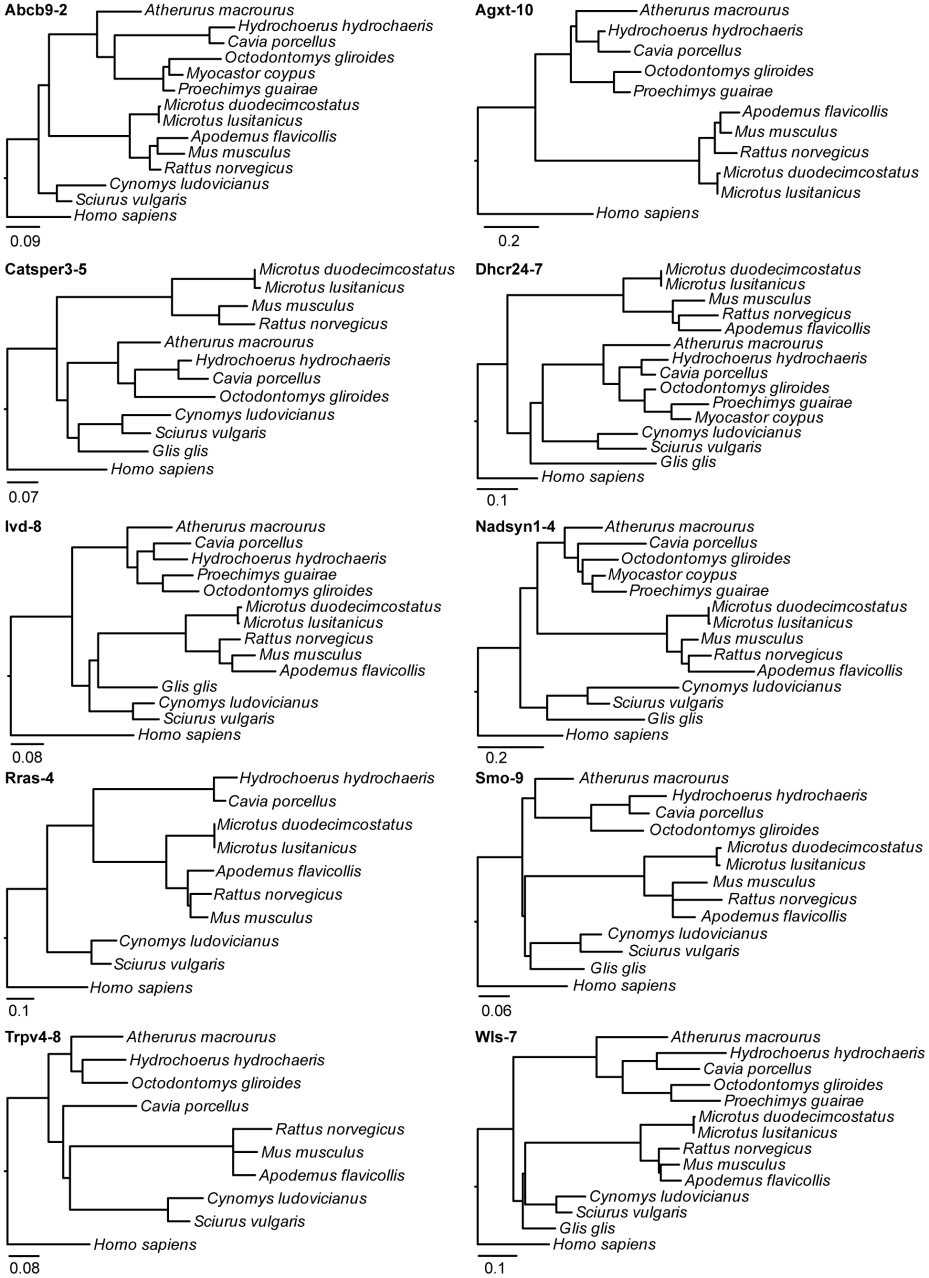
Maximum-likelihood phylogenetic trees of the 10 individual introns sequenced. A phylogenetic tree of each individual intron is shown. The analysis includes the rodent species successfully amplified in each intron plus mouse (*Mus musculus*), rat (*Rattus norvegicus*), guinea pig (*Cavia porcellus*) and human (*Homo sapiens*). The scale bars are in substitutions per position.

**Figure 3:**
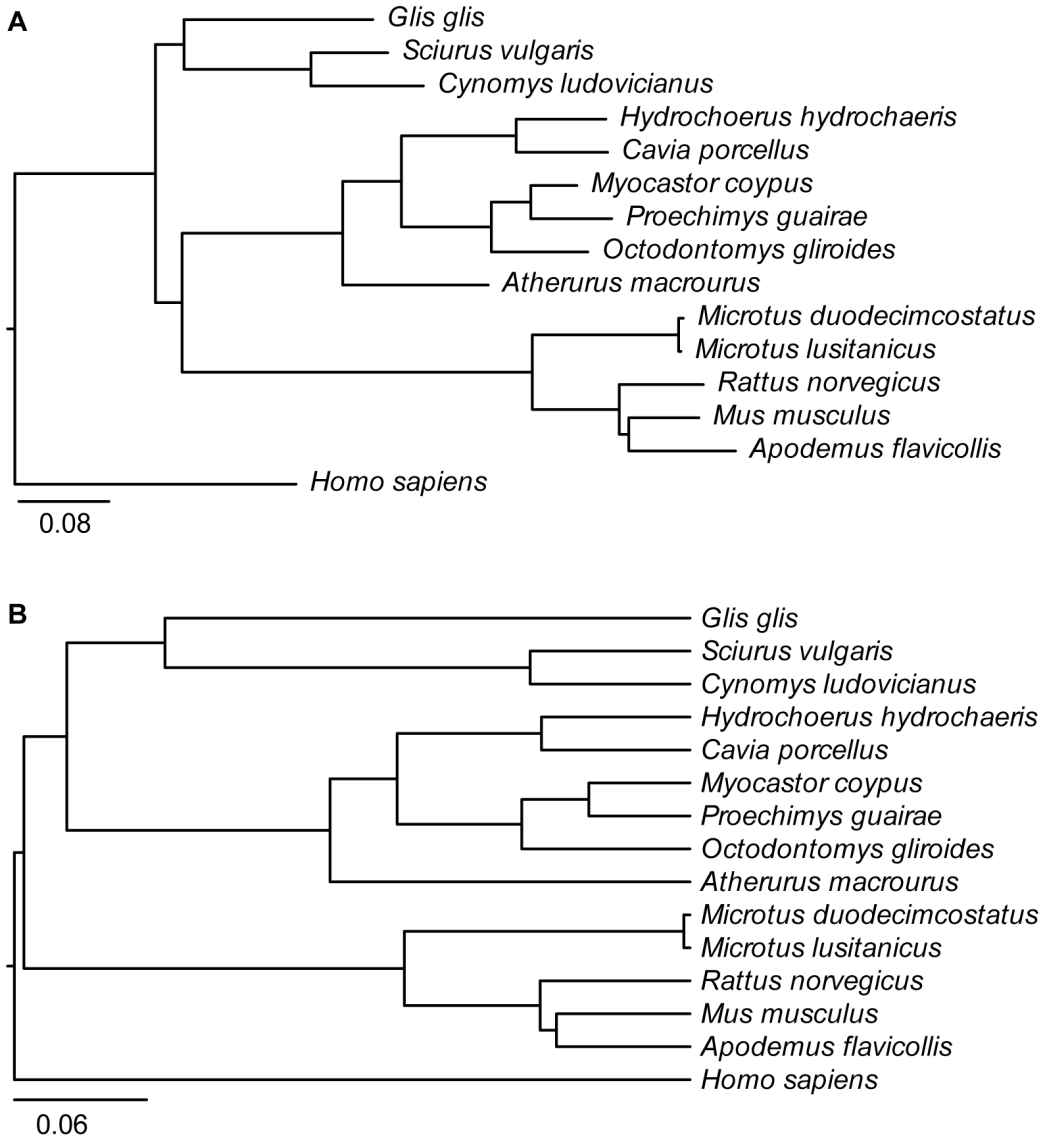
Phylogenetic analysis of the set of 10 introns sequenced. All introns were included in the phylogenetic analysis. (A) Maximum-likelihood phylogenetic tree. (B) Bayesian tree. The analysis includes the 11 rodent species sequenced in this work plus mouse (*Mus musculus*), rat (*Rattus norvegicus*), guinea pig (*Cavia porcellus*) and human (*Homo sapiens*). The scale bars are in substitutions/position.

We reconstructed maximum-likelihood phylogenetic trees of each individual intron obtained from the new rodent sequences, together with the sequences of mouse, rat, guinea pig and human (Figure 2). In addition, we reconstructed a maximum-likelihood tree and a Bayesian tree obtained from the set of all these sequences, which totaled 3055 positions (Figure 3). These trees, based on a large amount of information, agreed in general terms with the known tree of Rodentia [36], [37], [58]–[60] and were used as reference to evaluate the individual intron trees. To test if the individual intron trees were congruent with the rodent phylogeny, we first compared the maximum-likelihood tree obtained from each intron with the maximum-likelihood tree obtained from the set of all sequences using an SH test. In all cases the alternative topology could not be rejected. The same result was obtained when the alternative topology was the Bayesian tree. The congruence of the individual phylogenetic trees and the reference trees indicates that the new sequences obtained from these introns are likely to be orthologs in all species.

### Variability of the Introns between Closely Related Species

In order to assess the variability of these introns, we performed a closer inspection of the nine introns successfully amplified and sequenced in the two closely related species of the *Microtus* genus (Table 3). Seven out of the 18 sequence loci were heterozygous, as indicated by clearly ambiguous bases in the sequence traces. In addition, none of the introns were identical between the two species. There were 28 different positions between *M. lusitanicus* and *M. duodecimcostatus* in the alignments of the nine introns, which totaled 3720 positions. Therefore there was a mean intron divergence of 0.0075 substitutions/position (0.75%) between these two species. When this divergence was measured in the phylogenetic tree of mammals that included information of all introns (Figure 3), the sum of the terminal branch lengths of both species was 0.0061 and 0.0056 substitutions/position for the maximum-likelihood and Bayesian trees, respectively. The slightly shorter distances estimated in the trees are likely due to the fact that the alignments were cleaned with Gblocks previous to their use in the phylogenetic reconstruction. However, most positions eliminated with Gblocks were due to long individual insertions, which do not affect distances, and therefore the patristic distances measured from the mammalian trees are comparable to the ones measured from the alignments of the *Microtus* sequences. In addition, four of the introns showed differences in length between *M. lusitanicus* and *M. duodecimcostatus* due to the presence of indels (Table 3).

**Table 3:** Variability of introns in *Microtus lusitanicus* and *M. duodecimcostatus*.

Intron name	Heterozygous positions (*M. l.*, *M.d.*)	Alignment length (bp)	Number of differences	Number and length of indels
Abcb9-2	0, 0	403	1	
Agxt-10	0, 0	376	2	2 indels: 1 bp, 1 bp
Catsper3-5	0, 0	311	6	2 indels: 4 bp, 10 bp
Dhcr24-7	1, 1	340	3	
Ivd-8	1, 1	528	3	1 indel: 3 bp
Nadsyn1-4	3, 0	491	4	
Rras-4	1, 0	506	1	1 indel: 6 bp
Smo-9	0, 0	398	4	
Wls-7	3, 0	367	4	

## Discussion

The bioinformatic pipeline applied here (Figure 1) allowed us to obtain a set of 208 introns that constitutes a valuable tool for different studies of closely related species of rodents (Appendix S1 and S2). We tested 10 of these introns in a panel of 11 representative rodent species and found 79% of successful PCR reactions. Most of the positive reactions (73%) were obtained with the initial PCR annealing temperature and a further 6% of reactions produced clean PCR bands when the temperature was optimized. The fact that we obtained this high success rate is likely to be due to the different quality filters applied to the initial set of downloaded introns. In particular, the orthology and Blast filters were likely to have contributed for the most part to obtaining single PCR bands rather than multiple bands.

It is interesting to note that even in species for which no prior sequence information was available from close lineages for primer design, such as the sciurids *Sciurus vulgaris* and *Cynomys ludovicianus* or the Old World porcupine *Atherurus macrourus*, a high success rate was achieved. This indicates that the primers designed from the available rodent sequences may be valid for most rodent species. Although we did not test any Lagomorph, it is likely that, due to the proximity of this group to Rodentia [60]–[62], primers designed from the rodent genomes will also work with lagomorphs. Exon sequences of the lagomorphs *Oryctolagus cuniculus* and *Ochotona princeps* were actually included in the design of some primers; these sequences matched many of the designed primers and therefore they should also work with lagomorphs, but specific PCR tests are needed to demonstrate this.

Apart from the annealing temperature, we did not perform any additional optimization of the PCR reactions in this work but our experience with other species shows that designing primers in different position of the exons greatly helps to obtain further successful amplifications. Our data set (Appendix S1 and S2) includes the upstream and downstream exon alignments, which facilitates the design of different primers. It is also worth noting that the quality of the tissue is very important and, as with many other PCR applications, the use of well preserved tissue generally helps for additional intronic markers to be obtained. A typical study based on multiple loci should begin with a pilot study in which a number of candidate intron markers are tested. Those introns that amplify better (after some optimizations, if necessary) can be used for an in-depth study that includes the separate sequencing of the different alleles. A 79% success rate means that around 10 introns should be tested in order to be able to start using 8 suitable introns in the study.

The primary intention of the set introns developed here was to use them in studies of closely related species, which need highly informative markers. To test whether these introns showed adequate variability, we sequenced them in two sister species of the genus *Microtus* [63]. The mean divergence between the introns in these two species was 0.75%. While this degree of variability is much lower than the ∼4.5% divergence reported for mitochondrial genes [63], it should be sufficient for different analyses such as the reconstruction of species trees as long as several introns are included. The use of multiple independent loci is actually a fundamental requisite to reconstruct species trees under coalescent theory. Furthermore, the use of a high number of loci helps to reduce the variance of the different estimated parameters [4].

The maximum-likelihood and Bayesian trees obtained from the set of the 10 intron sequences obtained in this study (Figure 3) differed in the relationship among the three major rodent groups included in this species set: the squirrel-related clade (Sciuridae and Gliridae in our set), the mouse-related clade (Muridae and Cricetidae in our set) and the Hystricomorpha (Octodontidae, Hystricidae, Echimyidae, Myocastoridae and Caviidae in our set). Otherwise, the internal relationships within these groups are in agreement with the known rodent tree [37]. The most basal part of the tree remains controversial in the literature and, actually, our maximum-likelihood and Bayesian trees support two different views, with the most basal group being either the squirrel-related clade [36], [37], [58], [59] or the mouse-related clade [60], respectively. The overall congruence of the phylogeny of Rodentia obtained here with current studies indicates that, even though these introns were selected to have high evolutionary rates for their use in closely related species, they also contain useful information for reconstructing trees at higher taxonomic levels, as previously shown [64]–[67]. In fact, the use of Gblocks in the intron alignments led to the elimination of major insertions and deletions present in individual rodent species, but many informative blocks, which constitute the core of the introns in all species, remained. In total, 54% of the positions were eliminated, mainly due to these indels. Therefore both the core of the introns as well as indels are important aspects of the evolution of introns. The fact that a large fraction of these introns can be used for higher-level phylogenies can be potentially very helpful to estimate evolutionary rates for each intron in phylogenies with fossil calibrations [57].

For typical studies of groups of closely related species, a significant number of introns can be sequenced from different individuals using traditional sequencing techniques. However, the amplification of nuclear markers can also be used in combination with next generation sequencing (NGS) techniques for obtaining thousands of sequences of barcoded individuals, as recently shown [68], [69]. This parallel tagged sequencing method has the advantage of using optimally designed markers and, at the same time, exploiting the power of NGS techniques to rapidly generate large amounts of multi-locus DNA sequences. This opens the way to efficiently sequence a large number of intron markers such as those developed here, and to address different questions about speciation and phylogeography in a broad range of rodent species.

## Supporting Information

Figure S1 **Maximum-likelihood phylogenetic tree of 2288 concatenated rodent introns.**
(PDF)

Figure S2 **Distribution of scaling factor and K tree score in 2288 rodent introns.**
(PDF)

Table S1 Species used in the study.
(PDF)

Table S2 GenBank accession numbers.
(PDF)

Table S3 Nucleotide substitution model and type of molecular clock used in the Bayesian analysis of the rodent introns.
(PDF)

Appendix S1 **Final set of 208 introns selected for the phylogeny of closely related mammalian species (part a).**
(PDF)

Appendix S2 **Final set of 208 introns selected for the phylogeny of closely related mammalian species (part b).**
(PDF)
