# Supplementary material for: Development of Rapidly Evolving Intron Markers to Estimate Multilocus Species Trees of Rodents
Source: PLoS One. 2014 May 7;9(5):e96032. doi: 10.1371/journal.pone.0096032 (PMC4012946; doi:10.1371/journal.pone.0096032)
Supplement: Figure S1 — Maximum-likelihood phylogenetic tree of 2288 concatenated rodent introns. (PDF) [file pone.0096032.s001.pdf]

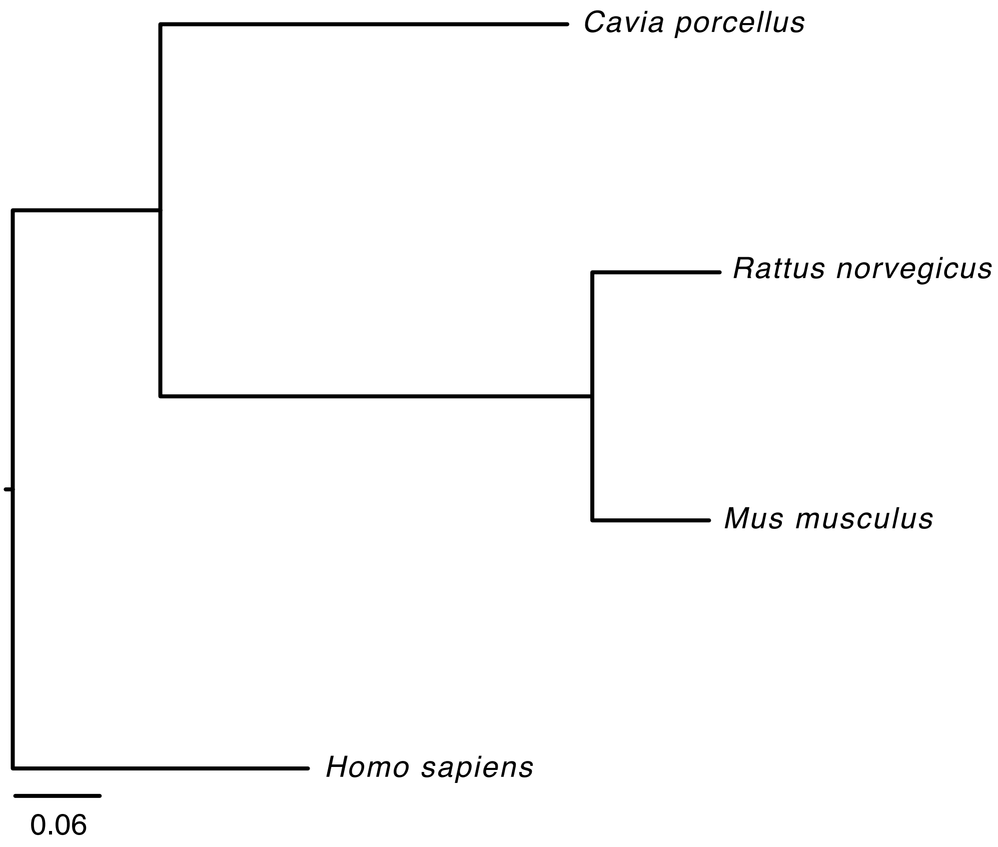


**Figure S1**. Maximum-likelihood phylogenetic tree of 2288 concatenated rodent introns.The scale bar is in substitutions per position.
