# Supplementary figures and images for: Development of Rapidly Evolving Intron Markers to Estimate Multilocus Species Trees of Rodents
Source: PLoS One. 2014 May 7;9(5):e96032. doi: 10.1371/journal.pone.0096032 (PMC4012946; doi:10.1371/journal.pone.0096032)

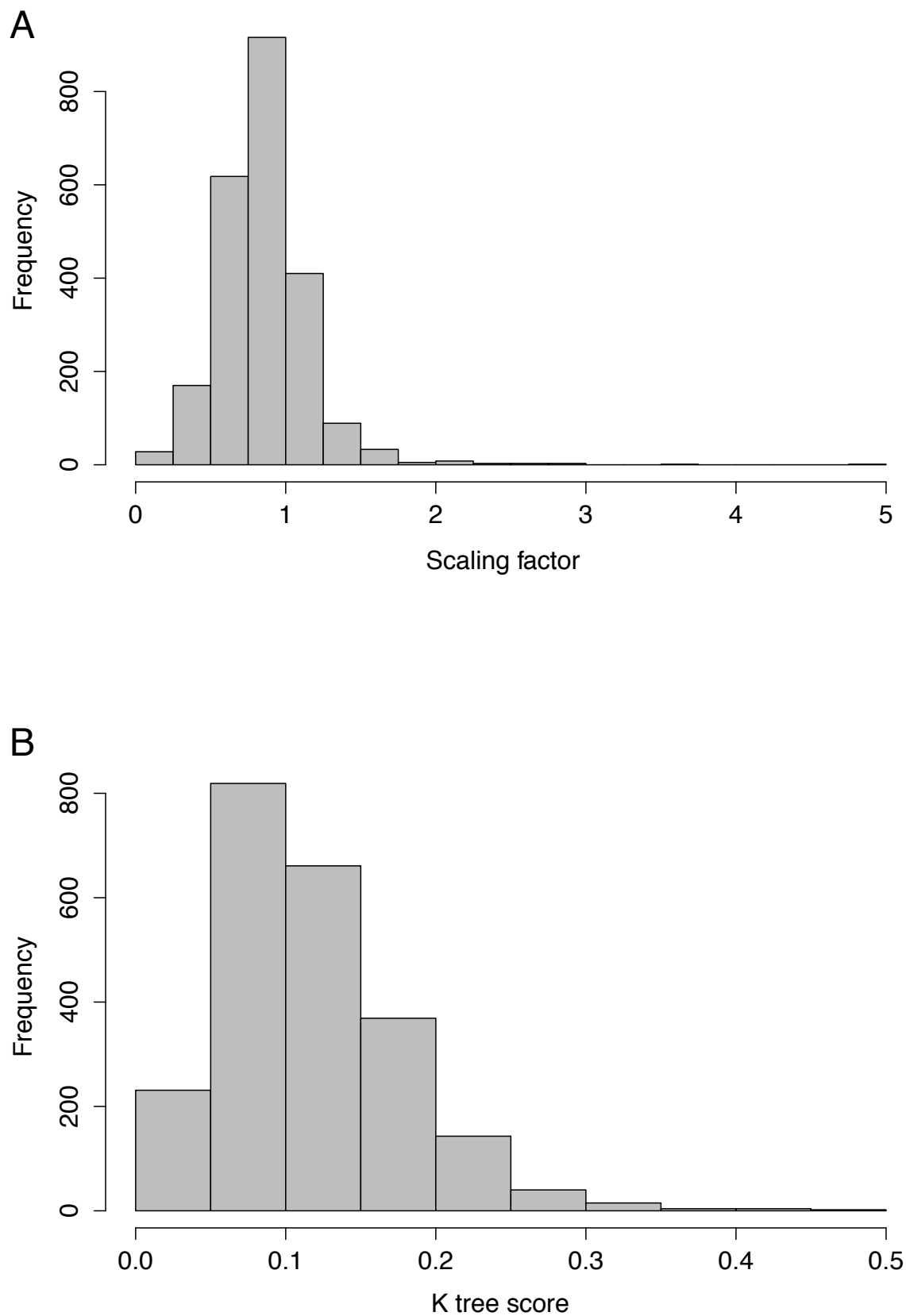

**Figure S2.** Distribution of scaling factor (A) and K tree score (B) in 2288 rodent introns.

Supplement: Figure S2 — Distribution of scaling factor and K tree score in 2288 rodent introns. (PDF) [file pone.0096032.s002.pdf]
