# Supplementary material for: Development of Rapidly Evolving Intron Markers to Estimate Multilocus Species Trees of Rodents
Source: PLoS One. 2014 May 7;9(5):e96032. doi: 10.1371/journal.pone.0096032 (PMC4012946; doi:10.1371/journal.pone.0096032)
Supplement: Table S1 — Species used in the study. (PDF) [file pone.0096032.s003.pdf]

**Table S1**. Species used in the study. The specimen code, family, locality and MGRS coordinates (when available) and donor (when not of our own collection) are indicated for each species. AMCC, The Ambrose Monell Cryo Collection at AMNH. BTAC, Banco de Tejidos Animales de Cataluña.

| Species | Specimen code | Family | Locality | Donor |
|---|---|---|---|---|
| *Octodontomys gliroides* | AMCC 103825 | Octodontidae | Sama, Tarija, Bolivia | AMCC |
| *Atherurus macrourus* | AMNH 275683 = AMCC 125125 | Hystricidae | Huu Lien Nature Reserve, Lang Son, Vietnam | AMCC |
| *Proechimys guairae* | AMCC 175991 | Echimyidae | Cabure, Serrania de San Luis, Venezuela | AMCC |
| *Myocastor coypus* | 131/02 | Myocastoridae | | BTAC |
| *Hydrochoerus hydrochaeris* | Z1107MS | Caviidae | | BTAC |
| *Cynomys ludovicianus* | 2009/57 | Sciuridae | | BTAC |
| *Sciurus vulgaris* | IBE-C2063 | Sciuridae | Villazzano, Trento, Italy (32TPS6501) | |
| *Glis glis* | IBE-C2161 | Gliridae | Villa Lagarina, Trento, Italy (32TPR5690) | |
| *Apodemus flavicollis* | IBE-C2054 | Muridae | Gavorrano, Grosseto, Italy (32TPN5554) | |
| *Microtus duodecimcostatus* | IBE-C2393 | Cricetidae | Belianes, Lleida, Spain (31TCG3305) | |
| *Microtus lusitanicus* | IBE-C1383 | Cricetidae | Matarrosa del Sil (29TQH03) | |
