# Supplementary material for: Development of Rapidly Evolving Intron Markers to Estimate Multilocus Species Trees of Rodents
Source: PLoS One. 2014 May 7;9(5):e96032. doi: 10.1371/journal.pone.0096032 (PMC4012946; doi:10.1371/journal.pone.0096032)
Supplement: Table S2 — GenBank accession numbers. (PDF) [file pone.0096032.s004.pdf]

Table S2. GenBank accession numbers.

	Abcb9-2	Agxt-10	Catsper3-5	Dhcr24-7	Ivd-8	Nadsyn1-4	Rras-4	Smo-9	Trpv4-8	Wls-7
Hydrochoerus hydrochaeris	KF129522	KF129531	KF129537	KF129544	KF129554		KF129572	KF129577	KF129585	KF129591
Proechimys guairae	KF129523	KF129532		KF129545	KF129555	KF129563				KF129592
Apodemus flavicollis	KF129524	KF129533		KF129546	KF129556	KF129564	KF129573	KF129578	KF129586	KF129593
Sciurus vulgaris	KF129525		KF129538	KF129547	KF129557	KF129565	KF129574	KF129579	KF129587	KF129594
Myocastor coypus	KF129526			KF129548		KF129566				
Atherurus macrourus	KF129527	KF129534	KF129539	KF129549	KF129558	KF129567		KF129580	KF129588	KF129595
Octodontomys gliroides	KF129528	KF129535	KF129540	KF129550	KF129559	KF129568		KF129581	KF129589	KF129596
Glis glis			KF129541	KF129551	KF129560	KF129569		KF129582		KF129597
Cynomys ludovicianus	KF129529		KF129542	KF129552	KF129561	KF129570	KF129575	KF129583	KF129590	KF129598
Microtus duodecimcostatus	KF129530	KF129536	KF129543	KF129553	KF129562	KF129571	KF129576	KF129584		KF129599
Microtus lusitanicus	KF916621	KF916622	KF916623	KF916624	KF916625	KF916626	KF916627	KF916628		KF916629
