# Supplementary material for: Development of Rapidly Evolving Intron Markers to Estimate Multilocus Species Trees of Rodents
Source: PLoS One. 2014 May 7;9(5):e96032. doi: 10.1371/journal.pone.0096032 (PMC4012946; doi:10.1371/journal.pone.0096032)
Supplement: Table S3 — Nucleotide substitution model and type of molecular clock used in the Bayesian analysis of the rodent introns. (PDF) [file pone.0096032.s005.pdf]

Table S3. Nucleotide substitution model and type of molecular clock used in the Bayesian analysis of the rodent introns.

Intron name	Nucleotide substitution model	Molecular clock
Abcb9-2	HKY+I	Relaxed
Agxt-10	HKY+G	Relaxed
Catsper3-5	HKY+G	Relaxed
Dhcr24-7	GTR+I	Relaxed
Ivd-8	HKY+G	Strict
Nadsyn1-4	HKY+G	Relaxed
Rras-4	HKY+G	Strict
Smo-9	HKY+G	Relaxed
Trpv4-8	GTR+G	Strict
Wls-7	GTR+G	Relaxed
