# Supplementary material for: Development of Rapidly Evolving Intron Markers to Estimate Multilocus Species Trees of Rodents
Source: PLoS One. 2014 May 7;9(5):e96032. doi: 10.1371/journal.pone.0096032 (PMC4012946; doi:10.1371/journal.pone.0096032)
Supplement: Appendix S1 — Final set of 208 introns selected for the phylogeny of closely related mammalian species (part a). (PDF) [file pone.0096032.s006.pdf]

ENSMUSG00000022469 intron 14

Description: Rap guanine nucleotide exchange factor 3 (Rapgef3)
Intron number: 14
Mouse chromosome: 15
Upstream exon length: 83
Downstream exon length: 40
Mouse intron length: 538
Intron alignment length: 669
Total murinae branch length: 0.23482
K_score: 0.08642
Scaling factor: 0.40374

ENSMUSG00000022469 exon 14 (ORF 0)

```
Mus     AAACTCTCAGACCTGGTGAGCAGAGATGCCCGACTTAGCAACTTGCTGAGGGAACAGTATCCAGAGAGACGGCGACACCA 80
Rattus  AAACTCTCAGACCTGGTGAGCAGAGATACCCGACTTAGCAACTTGCTGAGGGAACAGTATCCGGAGAGACGGCGACACCA 80
Cavia   AAACTCTCTGACCTGGTGAGCAGGGATGCCCGGCTCAGCAACCTACTGAGGGAGCAGTGGCCAGAGAGGCGGCGACATCA 80
Homo    AAACTCTCAGACCTGGTGGGCAGGGACACCCGACTCAGCAACCTGCTGAGGGAGCAGTGGCCAGAGAGGCGGCGATGCCA 80

Mus     CAG 83
Rattus  CAG 83
Cavia   CAG 83
Homo    CAG 83
```

ENSMUSG00000022469 exon 15 (ORF 1)

```
Mus     GTTGGAAAATGGCTGTGGGAACGTATCTCCTCAGACCAAG 40
Rattus  GTTGGAGAATGGCTGTGGGAATGTATCTCCTCAGACCAAG 40
Cavia   GTTGGAAAATGGCTGCGGGAGTGCATCTCCACAGTTGAAG 40
Homo    GTTGGAGAATGGCTGTGGGAATGCATCTCCTCAGATGAAG 40
```

ENSMUSG00000022469_intron_14

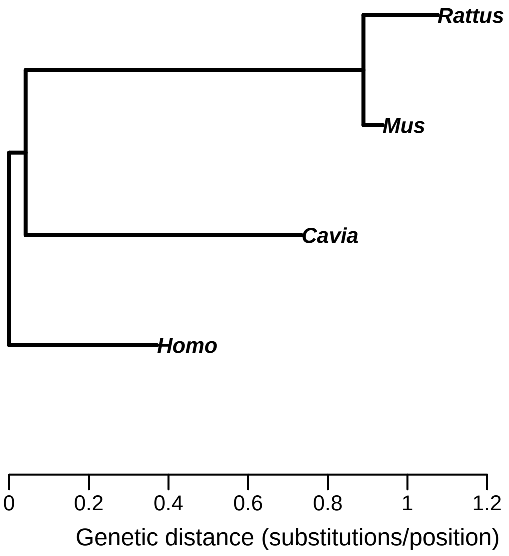

```
Mus     GTGACCAGTGACGAAAGCTTATCCATCCTCTTTGTG-----------CATCCAGGCC--------------------------------------TCCAGGCTAGACAGC 61
Rattus  GTGACCAGTGACCAGAGCTCATGGGTCCTCCTTGTG-----------CAGCAG---------------------------------------------AGGCTGAGCTGC 54
Cavia   GTGACCTTTG--------CCTTCACTCAGCCTCGTG-----------TACTAGGGATGGAGGATGCATGTGCAGGGCTGGCCTCTAGCTTTAGAATCCAGGCAGCACCAC 91
Homo    GTGATGCTTTGACTCTGTTAATTTGTCATTTCCATAACTGGCAGTGCCATGGGGACCAAGAGCATACGCTCTGGAGTCAAGCACTTGGCTTCAGATCCAGGCTGCACCAC 110

Mus     TTGTCTTAGCCCGTG-----------------------AGGGGTTCTTATCAGCCATTGTTCCGTTCCCACATATGCTACAGTCATCCTTTCGTTCATTCT-ATACATTG 147
Rattus  TGGCCCTGGTGCATC-----------------------CAGGCCTCCAG---------GCTCTGTCCCCACATATGCTACGGTCATTCATTTGCTTGTGCT-TTACA--- 128
Cavia   TCATTTCAGTTAGTGGCTTTTATTGGTCCCCTCGCTGTTCACCCCAGTGCCAGGTGT-CCACCATTCCCATAGTCC--GCAGTCACCTGCTTTGCTCCTGTCCCATCCGG 198
Homo    TTACTTCAATTCCTTGTTCTTATCAGTCCCCTTGCTGTTCACCCTCACACCACATGTCCCTCCATTCTCATGCATCAGGCACTCATTCATTTATTCATTCTCATATCCTG 220

Mus     AATGCACTTGC-GTACTTACATCTCCCCTTTGGC------CAATTACTGTGGACTAT-GGTACCGATGTCCACTGGCCCTGTCCATCGATCAACTCCTTTGAGTTTA-TC 248
Rattus  -------TTGT-GTGCCTATGTCTCCCTTTTGGC------CAATTACTGTGGACTAT-GGTACCCAGGTCCACTGGCCCTGCCCAGCAATCAACTCCTCTGAGTTTAATC 223
Cavia   AGTGCACCTCG-GCTCTCACTTCCTCCACCACACTTGTATCACTTGCTATCTA-----AGTGGCAGTCCCTGCCAGCCCCACCTGTCTACTAAG---------------- 286
Homo    AGTGCACCTGCTGTTCTCACTTCCTCCATTGGGCATGCATTTATTACCAAATACCCTCAGTGCCAGCTTCTGCCAGTCCCGCCCGGTA---CATTTCCTAGACTTTACCT 327

Mus     TTACTTCCTCAGACATCTCTGGGGG-----------------------------TAGCTGGGGCAAGAGCTCACTCAGATCCCAAGAACCTGTATCTTGCTCCTGCTTCC 329
Rattus  TTGCTTCCCCAGAAGCT-----GGG-----------------------------GTTCAAGGGCAAGAGCTTCTTTAGATCCCAAGAACCTGTATCTTGCTCCTGCTTCC 299
Cavia   -------------------------------------------------------------------------------------------------------------- 286
Homo    TCACCTTTGCAGATATTCCCACGGGAGCAGCTGAGATTCAGGGGGGTTGGGAGCTCATCTGAAACAGCACTTGATCTGAAACCCTGGGTCTGCATCTGCCTTTAACTTTT 437

Mus     TCCTT--CCTTCTGGCCTCTGGGAAACCACAGAAGCC--AATCCGGGTTTTAAACACA---CCTTGCCCC-CTGCCCTGTGTATGTCCCTTCTCACATCCCTCTGAACTT 431
Rattus  TCCTT--CCTTCTGGCCTCT-AGAAACCACAGAAGCC--CGTCCGGGTCTTAAGCGCA---CCTCACCCCGCTGCCCTGTGTACG-CTCTTCCCACATCCCTCTGAACTT 400
Cavia   -------CTTCCTAGACTGA-----------------------------------------------------------------------------TACCTTCTGAGTT 312
Homo    TCCTTCCCCTCCTGGCCTTG-GAAAATCACAAAGTCTGGGGGCCTGGTTTCAAGACCGTGTTCTCCCCTCTTTATGTGATACTTACCTGGTCGTGCTTACCTCTGAACCA 546

Mus     CTG---GGAAGGGCAAGATGGGATCCAGACCACTGTGGCCACTGGCCCTGAGAGTGTCCTGCCTG------CCAGGGTCCTCAGTGGCTC--TTTCTCTCTCTCACTCTT 530
Rattus  CTGGGTGGGGGGGCAAGATGGGATCCAGACCACTGTGGCCACTGGCCCTGAGAACACCCTACCTG------CCAGGGTCCTCAGTGGCTC--TCTCTCTCTCTCACTCTT 502
Cavia   CTG---ATGGGGACAGAATGGGATCCAGAGC----------CTGGCCCTG-------CCTCTGTG---CCCTGAGCGTGCCTGGTGCCCCCATTACAGCCCTTCTCTCTT 399
Homo    CTG---GTGAGGGCCGGGGAGAATCCAGA---------------GCCCCAAGGGCCTCCCTGCTGCAGCCCCAGGGATACCTGGGCTCAC--TCACCTCCCTTCTCTCTT 636

Mus     -CTCTGCAG 538
Rattus  -CTCTGCAG 510
Cavia   GCTCCGCAG 408
Homo    -GTCTGCAG 644
```

ENSMUSG00000021238 intron 2

Description: Methylmalonate-semialdehyde dehydrogenase (Aldh6a1)
Intron number: 2
Mouse chromosome: 12
Upstream exon length: 60
Downstream exon length: 75
Mouse intron length: 1583
Intron alignment length: 3383
Total murinae branch length: 0.18311
K_score: 0.07773
Scaling factor: 0.46395

ENSMUSG00000021238 exon 2 (ORF 0)

```
Mus     GTTTCTTCTAAGGTAAA------TGCCACATGGTATCCAGCATCCTCCTTCTCTTCCTCATCAGTG 60
Rattus  GTTTCTTCTAAAGTAAA------CTCCACATGGTGTCCAGCATCTTCCTTCTCTTCATCATCAGTG 60
Cavia   GTTTCTTCCAAGGTGAA------CTCTGTCTGGTATCCAGCATCCTCCTTCTCTTCTTCA---GTG 57
Homo    GTTTCTTCCAAGGTGAAATCCAGTCCCACCTGGTATTCAGCATCTTCCTTCTCTTCTTCA---GTG 63
```

ENSMUSG00000021238 exon 3 (ORF 0)

```
Mus     CCAACTGTAAAGCTCTTCATTGATGGAAAATTTGTTGAATCAAAAAGTGATAAATGGATTGACATCCACAACCCA 75
Rattus  CCAACTGTAAAGCTCTTCATTGATGGGAAATTCGTTGAATCCAAAAGTGACAAATGGATTGACATCCACAACCCA 75
Cavia   CCAACTGTAAAACTCTTCATTGATGGGAAATTTGTTGAATCCAAAAGTGACAAATGGATTGACATTCACAACCCA 75
Homo    CCAACTGTAAAGCTCTTCATTGGTGGGAAATTCGTTGAATCCAAAAGTGACAAATGGATCGATATCCACAACCCA 75
```

ENSMUSG00000021238_intron_2

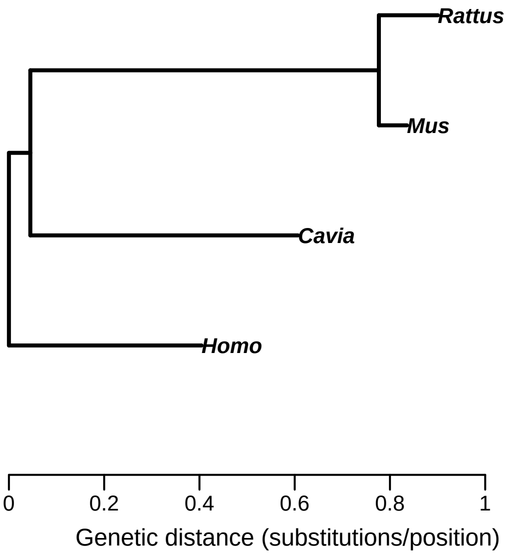

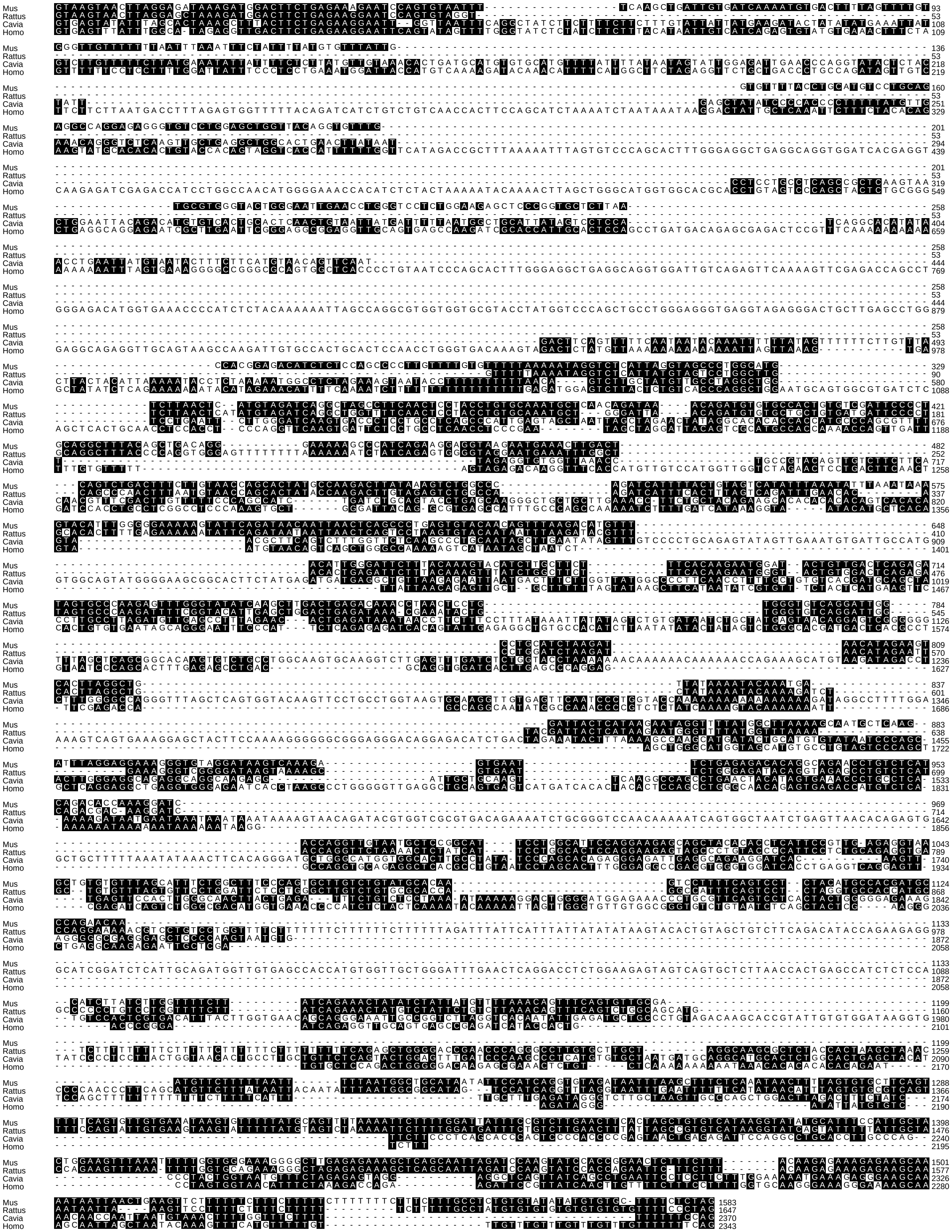

ENSMUSG00000036733 intron 6

Description: RNA-binding protein 42 (Rbm42)
Intron number: 6
Mouse chromosome: 7
Upstream exon length: 176
Downstream exon length: 333
Mouse intron length: 508
Intron alignment length: 833
Total murinae branch length: 0.18830
K_score: 0.07239
Scaling factor: 0.46866

ENSMUSG00000036733 exon 6 (ORF 2)

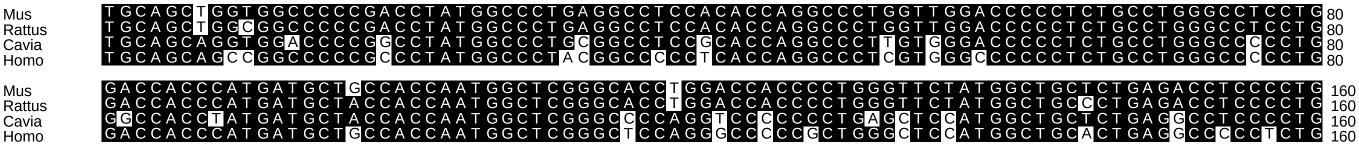

ENSMUSG00000036733 exon 7 (ORF 0)

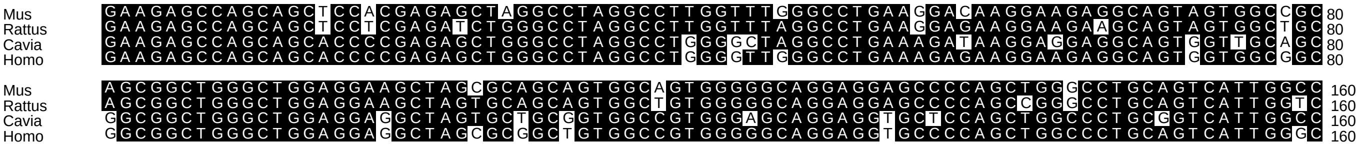

ENSMUSG00000036733_intron_6

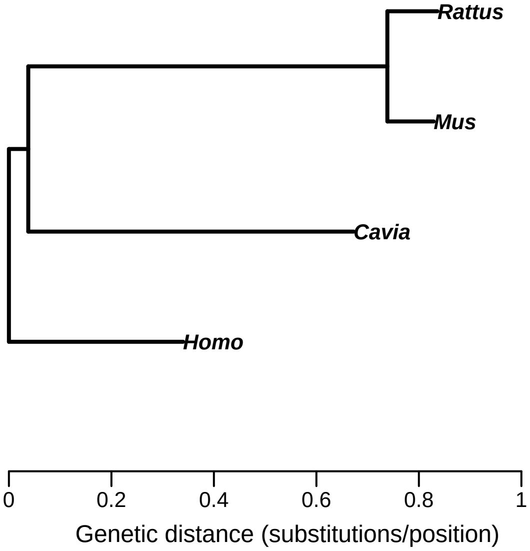

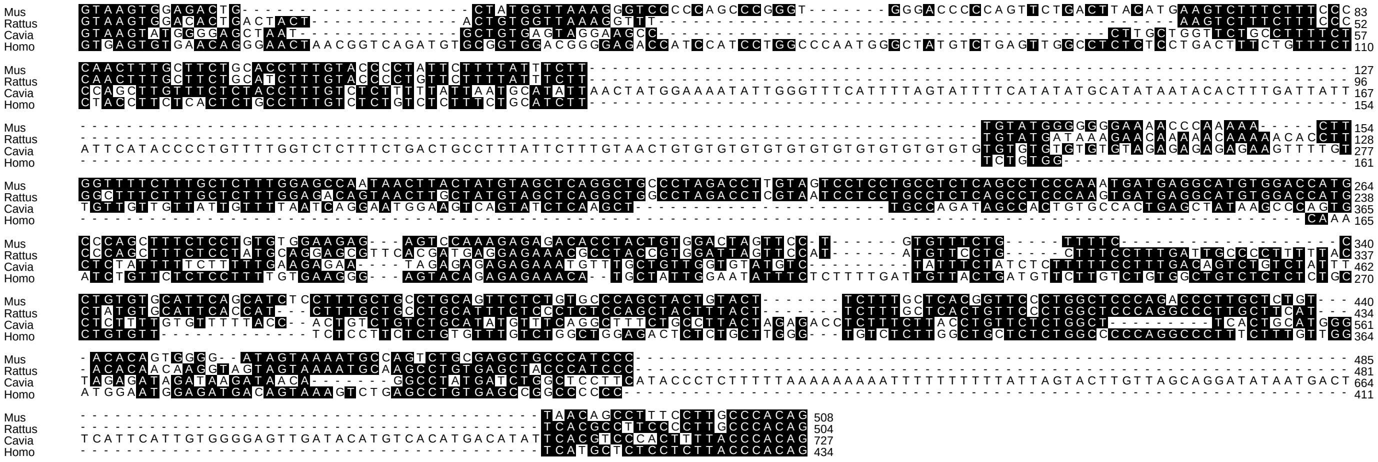
Mus
Rattus
Cavia
Homo

ENSMUSG00000029512 intron 11

Description: Serine/threonine-protein kinase ULK1 (Ulk1)
Intron number: 11
Mouse chromosome: 5
Upstream exon length: 51
Downstream exon length: 89
Mouse intron length: 289
Intron alignment length: 460
Total murinae branch length: 0.18766
K_score: 0.08762
Scaling factor: 0.48092

ENSMUSG00000029512 exon 11 (ORF 2)

```
Mus     ATGAATTTTTCCACCACCCTTTCTTGGATGCCAGCACCCCCATCAAGAAAT 51
Rattus  ATGAATTTTTCCACCACCCTTTCTTAGATGCCAGCACCCCCATCAAGAAAT 51
Cavia   ATGAGTTTTTCCACCACCCTTTTCTGGACGCCAGCGCCTCTGTCAGGAAGT 51
Homo    ATGAGTTTTTTCATCACCCTTTCCTCGATGCCAGCCCCTCGGTCAGGAAAT 51
```

ENSMUSG00000029512 exon 12 (ORF 2)

```
Mus     CCCCACCTGTGCCTGTGCCCTCATATCCAAGCTCAGGGTCTGGCAGCAGCTCCAGCAGCAGCTCTGCCTCCCACCTGGCC 80
Rattus  CCCCACCTGTGCCTGTGCCCTCATATCCAAGCTCAGGGTCCGGCAGCAGCTCCAGCAGCAGCTCTGCCTCCCACCTGGCA 80
Cavia   CCCCGCCCGTGCCTGTACCCTCCTACCCGAGCTCAGGGTCCGGCAGCAGCTCCAGCAGCAGCTCCACCTCGCACCTGGCC 80
Homo    CCCCACCCGTGCCTGTGCCCTCGTACCCAAGCTCGGGGTCCGGCAGCAGCTCCAGCAGCAGCTCCACCTCCCACCTGGCC 80

Mus     TCTCCACCG 89
Rattus  TCTCCCCCG 89
Cavia   TCTCCTCCG 89
Homo    TCCCCGCCG 89
```

ENSMUSG00000029512_intron_11

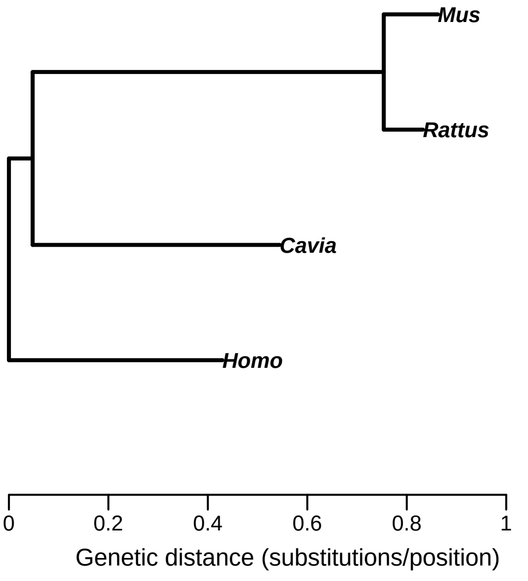

```
Mus     GTGAGTCTCTGGCAGACTTGGTCACAGACAGGGCTTGGGGCATGCTTCCCAATGTCTGGAGGT---GCCACATTGCTATCT-------------------------------- 78
Rattus  GTGAGTCTCTGGCAGTCTTGGGCACAGACAGGGCTTGTGACATGCTCCCCAATGTCTGTAGGT---GCCACATTGCTATCT-------------------------------- 78
Cavia   GTGAGTGCTGGTGGG--CTGGGCCTGCTGGTGG-TTGAGGGAGGCATCTCATGCTGGGTGGGGATGGTCATGGTGCGGTGG-------------------------------- 78
Homo    GTGAGTTTCTGTGGGTCCTGGGGCTCCGCATGG-GCCCTGCATGCACCCCTTCCTCCCCGGGTTTGGTTAGGGCACTGAGTGTGGGGCAGAGGGAGCAGGTCTTGAGCTG 109

Mus     ------------------------------------------------------------------------------------------TGGGACATATTCTGGAAAGG 98
Rattus  ------------------------------------------------------------------------------------------TGGGAGATAGTCTGGAAAGG 98
Cavia   ------------------------------------------------------------------------------------------TTGGGGT------------- 85
Homo    CGGCCAGCTCCCAGGGAGGGCAGGACACCCGGCTCCTTGCTCTGCTGCAGCGGGGACCCCAAGGCCAGGGGTTCGCATGGTGCAGCAGGCTTGGGATGGGGATGGGGTGT 219

Mus     TGGAGTGCCTCGTTCTGCTTTTTC---AAGGCCATG---------TGTTACCTGGGTGGCTCTGCTGT------CATTGGCTTAGGGACA---CTGGAGGTATCAGTGTA 187
Rattus  TGGAACGTCTCATTCTGCCTCTTCTTGAAGGCCATG---------TA-TACTTGGGTGGTTCTGCTGT------TGTGGGCTTGGGGGCA---CTGGAGGTACCAGTGCA 189
Cavia   ---TGAGGCTGATGCCACCTTGTG---CTGGCCATGACAGGATGATGGGGCCACAGAGGGTCTGCTGTCACAGTCATGGGTCGGGGTGAATGTCTTTATGCTTCAGGGCT 189
Homo    TGGAGGGGCTGGTGCTGCCCTGTG---ACAGTCATA-------GGGTGGCCCAGGAGGGAGCCTCCCTCCTTCCTGCTGGTCGGGGTGTA-----------------GCC 302

Mus     GGGTGGCTGCTTGTCCCTGTGCTGGGAAT-TGGAGTACTGGTGCCAGTC--AGGCAGCCA-TGTCTCCAAAGCTGTGATG----------------CACACTGACCCATA 277
Rattus  GGGTG---GCATAACCTGATACTGGGTGCATGTCACTGTGGTATCAGTCAAAGGCAGCCA-TGTCTCCAAAGCTGTAGTGATAT-CCAGCAGTGTGCACACTGCCCCATT 294
Cavia   GGGGTACTGAGAACCATACTGCAGGGGCTAGGCCTGGGTGCACCTGCCCTGGGGAAGTGGCTGTCCCCTGTGC--------------AGGGTGGGACAGCACGTCCCACA 285
Homo    GGAAG--------------TGGAGGGGATATGGTTGAGCTTGTCCAGTCTGTGG-----GTTGGCTCGAGGGTGAGGAGGAGACAGGAGGATGGCCCAGACTGACCTATG 393

Mus     TC--------CCTCTTGCAG 289
Rattus  TCCTTC----CCTCTTGCAG 310
Cavia   GACGCTCTCGCCCTCCACAG 305
Homo    CATG------TTTATCTCAG 407
```

ENSMUSG00000014158 intron 8

Description: Transient receptor potential cation channel subfamily V memb (Trpv4)
Intron number: 8
Mouse chromosome: 5
Upstream exon length: 93
Downstream exon length: 74
Mouse intron length: 398
Intron alignment length: 433
Total murinae branch length: 0.27211
K_score: 0.08782
Scaling factor: 0.51597

ENSMUSG00000014158 exon 8 (ORF 0)

```
Mus     CCACCCTACCCTTACCGGACCACAGTGGACTACCTGAGGCTGGCTGGCGAGGTCATCACGCTCTTCACAGGAGTCCTGTT 80
Rattus  CCACCCTACCCTTACCGTACCACGGTGGACTACCTGAGGCTGGCTGGTGAGGTCATCACGCTCCTCACAGGAGTCCTGTT 80
Cavia   CCACCCTACCCTTACCGCACCACAGTGGACTACCTGAGGCTGGCCGGCGAGGTCATCACGCTCTTCACGGGGGTGCTGTT 80
Homo    CCGCCGTACCCTTACCGCACCACGGTGGACTACCTGCGGCTGGCTGGCGAGGTCATTACGCTCTTCACTGGGGTCCTGTT 80

Mus     CTTCTTTACCAGT 93
Rattus  CTTCTTTACCAGT 93
Cavia   CTTCTTCACCAAT 93
Homo    CTTCTTCACCAAC 93
```

ENSMUSG00000014158 exon 9 (ORF 0)

```
Mus     ATCAAAGACTTGTTCACGAAGAAATGCCCTGGAGTGAATTCTCTCTTCGTCGATGGCTCCTTCCAGTTACTCTA 74
Rattus  ATCAAAGACTTGTTCATGAAGAAATGCCCTGGAGTGAATTCTCTCTTCGTCGATGGCTCCTTCCAGTTGCTCTA 74
Cavia   ATCAAAGACTTGTTCATGAAAAAATGTCCTGGAGTGAACTCACTCTTCATCGACGGCTCCTTCCAGCTGCTCTA 74
Homo    ATCAAAGACTTGTTCATGAAGAAATGCCCTGGAGTGAATTCTCTCTTCATTGATGGCTCCTTCCAGCTGCTCTA 74
```

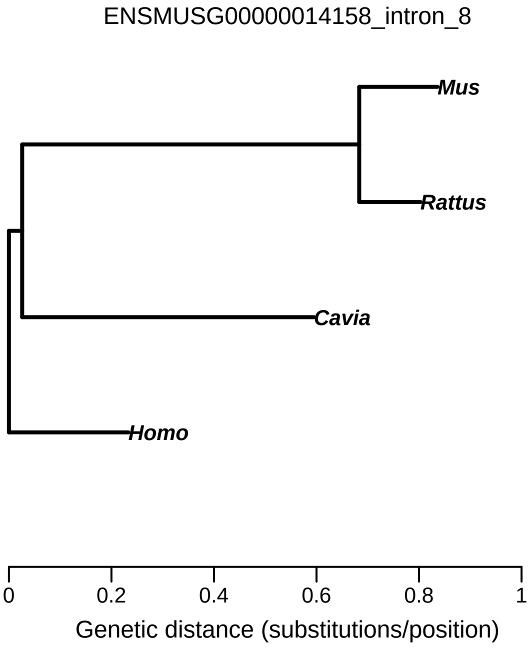

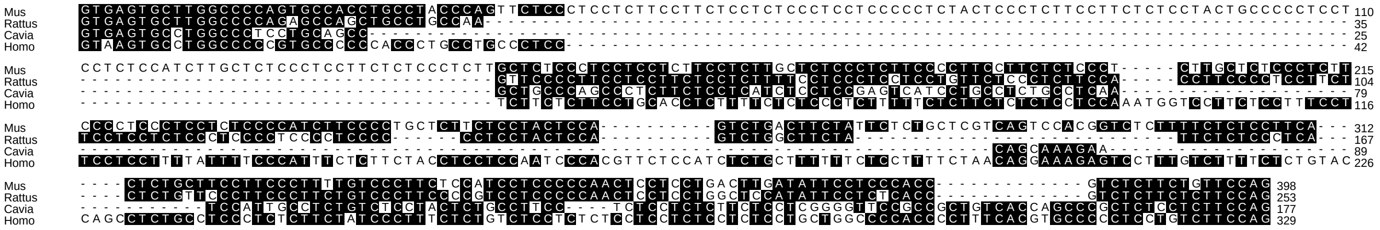
Mus
Rattus
Cavia
Homo
110
35
25
42
215
104
79
116
312
167
89
226
398
253
177
329

ENSMUSG00000037820 intron 12

Description: Protein-glutamine gamma-glutamyltransferase 2 (Tgm2)
Intron number: 12
Mouse chromosome: 2
Upstream exon length: 137
Downstream exon length: 151
Mouse intron length: 895
Intron alignment length: 734
Total murinae branch length: 0.15886
K_score: 0.08338
Scaling factor: 0.52074

ENSMUSG00000037820 exon 12 (ORF 0)

```
Mus     GTCCTGGGAGAACCCAAGCAAAACCGCAAACTGGTGGCTGAGGTGTCCCTGAAGAACCCACTTTCCGATCCCCTGTATGA 80
Rattus  ATCCTGGGGGAGCCCAAGCAGAACCGCAAACTGGTGGCTGAGGTGTCCCTGAAGAACCCACTTTCTGATTCCCTGTATGA 80
Cavia   GTCTTGGGGGAGCCCAAGCAGAACCGCAAGCTGATTGCTGAGGTGTCTCTGAAGAATCCGCTCCCTGTGCCGCTGCTGGG 80
Homo    ATCCTTGGGGAGCCCAAGCAGAAACGCAAGCTGGTGGCTGAGGTGTCCCTGCAGAACCCGCTCCCTGTGGCCCTGGAAGG 80

Mus     CTGCATCTTCACTGTGGAGGGGGCTGGCCTGACCAAGGAGCAGAAGTCTGTGGAAGT 137
Rattus  CTGTGTCTTCACTGTGGAGGGGGCTGGCCTGACCAAGGAACAGAAGTCTGTGGAGGT 137
Cavia   TTGTATCTTCACCGTGGAAGGAGCTGGCCTGACCAAGGACCAGAAGTCGGTGGAGGT 137
Homo    CTGCACCTTCACTGTGGAGGGGGCCGGCCTGACTGAGGAGCAGAAGACGGTGGAGAT 137
```

ENSMUSG00000037820 exon 13 (ORF 1)

```
Mus     CTCAGACCCGGTGCCAGCGGGCGATTTGGTCAAGGCACGGGTCGACCTGTTCCCGACTGATATTGGCCTCCACAAGCTGG 80
Rattus  CTCAGACCCTGTGCCAGCAGGAGATGCGGTCAAGGTGCGGGTTGACCTGTTCCCGACTGATATTGGCCTCCACAAGTTGG 80
Cavia   CCCAGACCCCGTGGAAGCAGGGGAGCAAGCGAAGGTACGGGTGGACCTGCTGCCGACGGAGGTGGGCCTCCACAAGCTGG 80
Homo    CCCAGACCCCGTGGAGGCAGGGGAGGAAGTTAAGGTGAGAATGGACCTGCTGCCGCTCCACATGGGCCTCCACAAGCTGG 80

Mus     TGGTGAACTTCCAGTGTGACAAGCTGAAGTCGGTGAAGGGTTACCGGAATGTTATCATCGGCCCGGCCTAA 151
Rattus  TGGTGAACTTCCAGTGTGACAAGCTGAAGTCGGTCAAGGGTTACCGGAATATCATCATCGGCCCGGCCTAA 151
Cavia   TGGTGAACTTCGAGTGCGACAAGCTGAAGGCCGTGAAGGGCTATCGGAACGTCATCATCGGCCCCGCCTAA 151
Homo    TGGTGAACTTCGAGAGCGACAAGCTGAAGGCTGTGAAGGGCTTCCGGAATGTCATCATTGGCCCCGCCTAA 151
```

ENSMUSG00000037820_intron_12

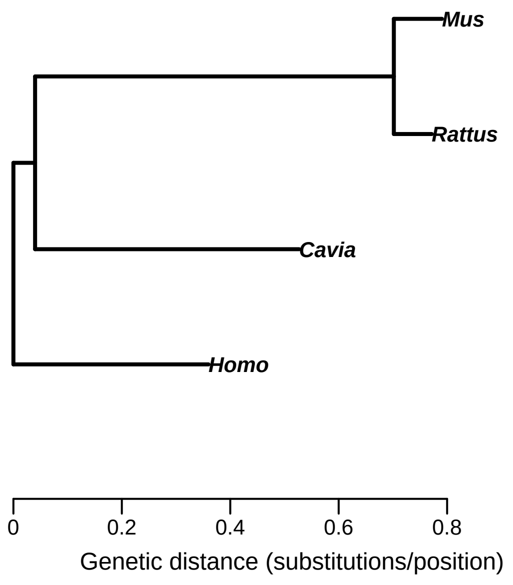

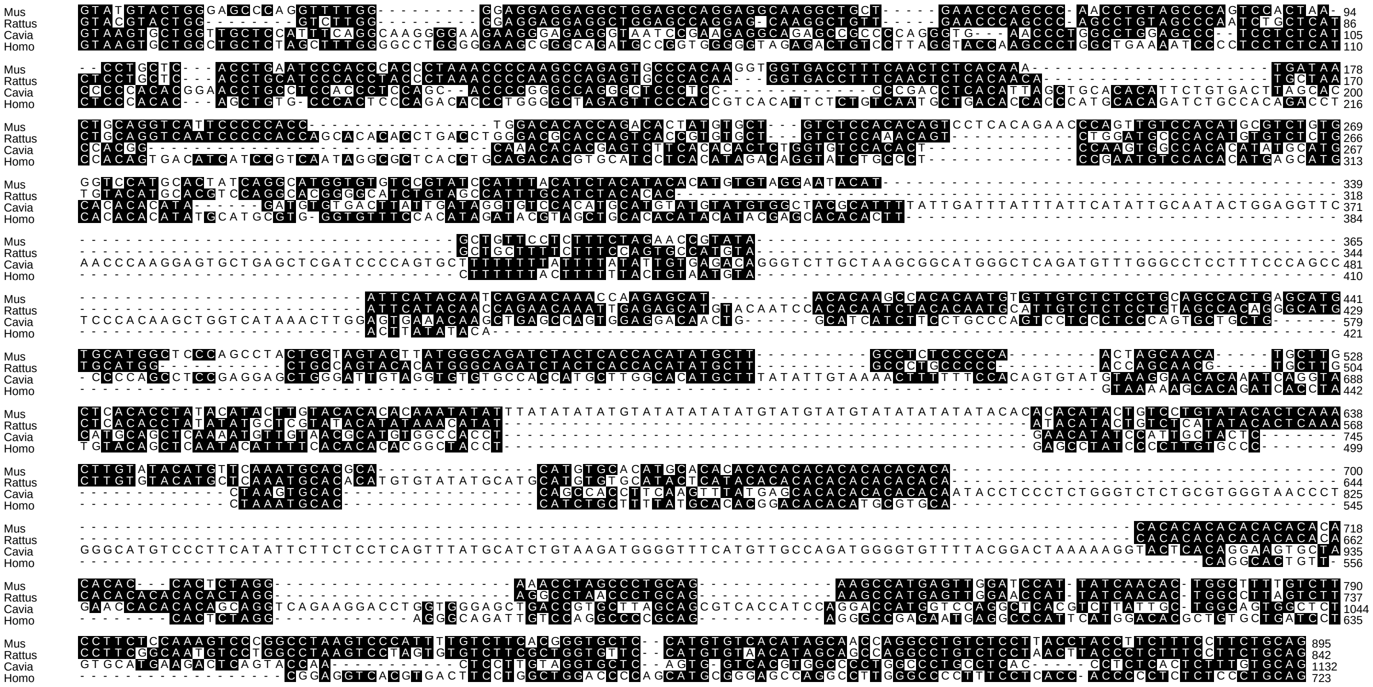
Mus
Rattus
Cavia
Homo

ENSMUSG00000013921 intron 4

Description: CLIP-170-related protein (Clip3)
Intron number: 4
Mouse chromosome: 7
Upstream exon length: 162
Downstream exon length: 119
Mouse intron length: 403
Intron alignment length: 527
Total murinae branch length: 0.18591
K_score: 0.07984
Scaling factor: 0.52602

ENSMUSG00000013921 exon 4 (ORF 2)

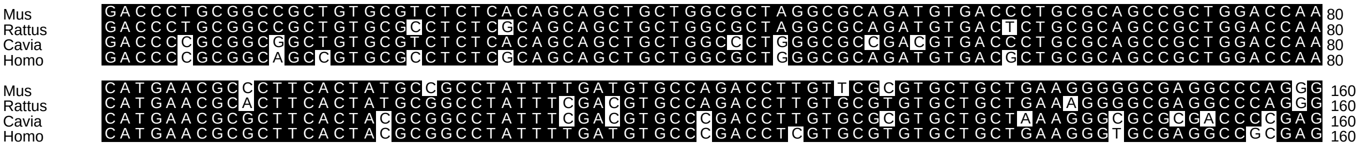

ENSMUSG00000013921 exon 5 (ORF 2)

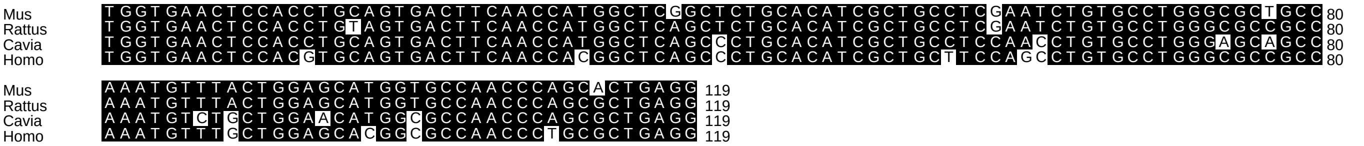

ENSMUSG00000013921_intron_4

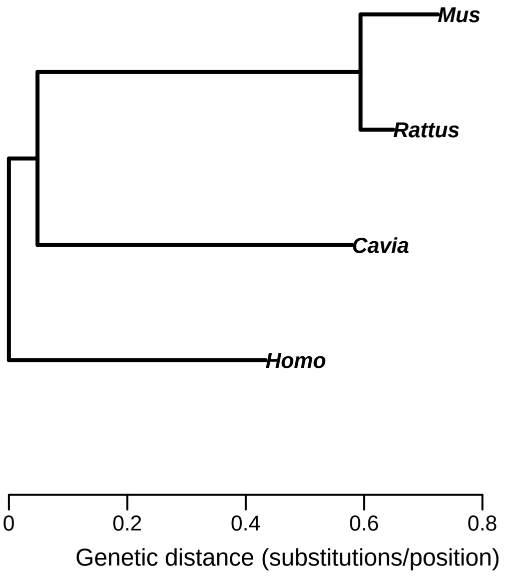

```
Mus     GTGAGGAGGCGGAGTCTCTCTGGAACGGGG-TGTGGCTTAGATGAAGGGTGGAGTTTC-------------------------------------------------TTA 60
Rattus  GTGAGGAGGCGGAGCCTTGCTGGGATGGGG-AGGGGCCTGGATGAGGGGTGGAGTTTC-------------------------------------------------TT- 59
Cavia   GTGAGGAGAAAGGTTCTGGAAGCAAGGAGG-AGGACCTAGGGGAAAGGGTGGGGACGTGGGGTGGCGGGCACGACCCAGGCCTGTCTGAGCGAGATGGTGACAGACGCGA 109
Homo    GTGAGGAGGAGAGCCCTGGCTGGGAGGGGGCAAGGACCTGGGTGAGGGACGCGGAGAC-------------------------------------------------CCA 61

Mus     TAGGGAGAAGGGTACCCAGGGTTTTGAGAGCGTGGGGGCCACCATCCATCTGGTTAAGGGGGGAAAAA------------------------------CTCAAGATGAAA 140
Rattus  ---GGAGGAGGGGGCCCAGGGTTTTGAGAGTGTGGGGGCCACCATCCATCTGGTTAAGGGGGAAAAAAT-----------------------------CCCAAGACGAAA 137
Cavia   GGCGGGCATGGGAAGGGTAGGTTGGGTGGGGGCGGGGCCGGGGGCGGGGCCAGCGACTGGGGGCGGGACTCTAGGCGAGGTTTTGGGAGGGAACCCTTCATAGAGCTGGG 219
Homo    GGTGAGGCCAGGCTGCGAGGGGAAGGAGGACGGTGATCTGAGGAGATAGCGGGAGGTTGGAGG-----------------------------------CGTGGGAGAGGG 136

Mus     GGATAAAAGACTGAGGCCTAAGGTTGAAGGGTGCCAGGATTCAAACAGGAAACAAGGGATAGATAGACAT--GCACGGGCTCGGGATGGCAGGTTGTGTTGGTTGTGTTG 248
Rattus  GGATAAAAGACTGAGGCTTAACTTTGAAGGGTGCGGGGATTCAAACAGGAAACAAGGGCTAGAGGGGCATGCGCATGCGCACAGGGTAGGGGGGGCTGTTGGCC------ 241
Cavia   AGAGAGTATCCTAACACCCTGGGATGCTGTGGCCAGAGACTCACAAGTGGCGGCCAGCCT-------------------------------------------------- 279
Homo    GGCTCAGGGACTACCACTCTAGG-----GAGGGTCGGGGCTGCCAAGGGAGAGGGGGGCT-------------------------------------------------- 191

Mus     GTGGGGGGTAAGTGGTGATGGGGTCATAT---------------ATGTGACAGAAGGGTAGGTTGGGCAAGATGTTTAGAGACTGGGAGCCCTGAGCAG----------- 332
Rattus  ----AGGGTATGTAGTGATTGGCTTATATACAGGATGGACAACAATGGGATAGAAGGGCAGGTTGGGAAAGATGCTTAGAGCCAGGAACCCCG-----A----------- 331
Cavia   ----GGGCAATTTAATGATTTAGCCAGAC---------------TAGAAATAAAAAA---GGGTGGGGATGTAACTCAGTGCGAAGGCCCTGGGTGCAGTTTCTGGTACC 367
Homo    ---------------------------------------------------------CCGAGGTGGGGGCGGGGCTCTGCGTCGGAGGCTCTGGGACCT----------- 233

Mus     GCAGGGGTGAGGCTCCATGCAGATGTGGCAGA-------------CACTCACTCTTACCC---TTCCCTCACCTGCCCACTTCCCAG 403
Rattus  GCAGAGGTGAGGCTCCATGCAGATGCTGCAGGAGG---------TCACTCACTCTTACCC---TTCCCTCACATGCCCACTTCGCAG 406
Cavia   GCAGGGGGAAAAT----------GGTAGGAGAGCTGTATTATTTCCACTTG-GCTCACCTAGAACTCTGTTCTTACCCCCTTCGCAG 443
Homo    GCAAGTTAAGAGATGAGCCCCGAGGTGGGAGGAGGGCTGGGGTCCCACAGGCGCTCACCCGGAACCCCTCCTCTTGCCCACTTCCAG 320
```

ENSMUSG00000032413 intron 11

Description: Ras GTPase-activating protein 2 (Rasa2)
Intron number: 11
Mouse chromosome: 9
Upstream exon length: 149
Downstream exon length: 115
Mouse intron length: 860
Intron alignment length: 1224
Total murinae branch length: 0.26616
K_score: 0.06252
Scaling factor: 0.53066

ENSMUSG00000032413 exon 11 (ORF 0)

```
Mus     CCAGTATCCGCCTCAGCTGCTTATATTTTGGGTGAAATATGTCAAGATCAAAAGGATGCTGTTTTGCCCCTTGTACGACT 80
Rattus  CCAGTATCCGCCTCAGCTGCTTATATTTTGGGTGAAATATGTCGAGACCAGAAGGATGCCGTTTTGCCCCTCGTACGACT 80
Cavia   CCAATATCTGCCTCAGCTGCTTACATTTTGGGTGAAATATGTCGAGATAAAAATGATGCTGTTTTGCCCCTTGTACGACT 80
Homo    CCAATATCTGCCTCAGCTGCTTACATTTTGAGTGAAATATGTCGAGATAAAAATGATGCTGTTTTGCCCCTTGTACGACT 80

Mus     GTTGCTGCATCACAATAAACTTGTCCCTTTTATCACTGCGGTGGCCGAGCTGGACTTGAAGGATACTCC 149
Rattus  GTTGCTGCATCACAATAAGCTTGTTCCCTTTATCACTGCGGTGGCTGACCTGGACTTGAAGGATACTCA 149
Cavia   GTTGCTGCACCATGATAAACTTGTTCCTTTTGTCACTGCAGTGGCTGAATTAGACTTGAAGGATACCCA 149
Homo    GCTGCTGCACCATGATAAACTTGTTCCTTTTGCCACTGCTGTGGCTGAATTAGACTTGAAGGATACACA 149
```

ENSMUSG00000032413 exon 12 (ORF 1)

```
Mus     AGATGCCAACGCAATATTTAGAGGAAACTCCCTGGCTACCCAGTGTCTGACTGAGATGATGAAGATAGTGGGAGGGCACT 80
Rattus  AGATGCCAACGCAATATTTAGAGGAAACTCCCTGGCTACCCAGTGTCTGACTGAGATGATGAAGATAGTGGGAGGGCACT 80
Cavia   AGATGCAAACACAATTTTTAGAGGAAATTCCCTGGCTACCCGATGTCTGGATGAGATGATGAAAATAGTGGGAGGGCACT 80
Homo    AGATGCAAACACAATTTTTAGAGGAAATTCCCTGGCTACCCGATGTCTGGATGAGATGATGAAAATAGTGGGAGGGCACT 80

Mus     ACCTGAAAGTCACGCTGAAGCCCGTTCTAGATGAG 115
Rattus  ACCTGAAAGTCACATTGAAGCCTGTTCTGGATGAG 115
Cavia   ACCTGAAAGTAACATTAAAATCTATTCTAGATGAG 115
Homo    ACCTGAAAGTAACATTAAAACCTATTCTTGATGAG 115
```

ENSMUSG00000032413_intron_11

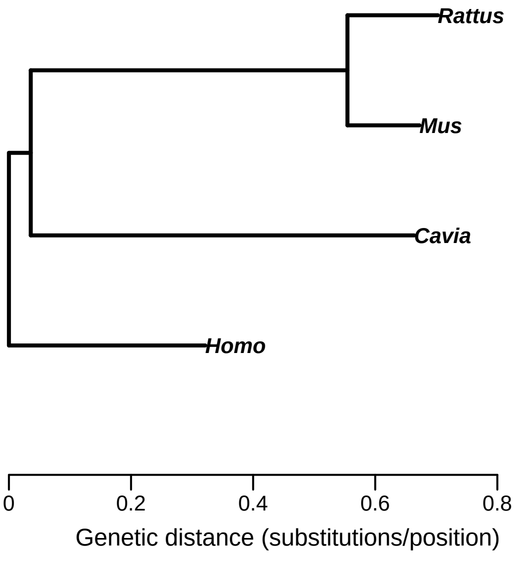

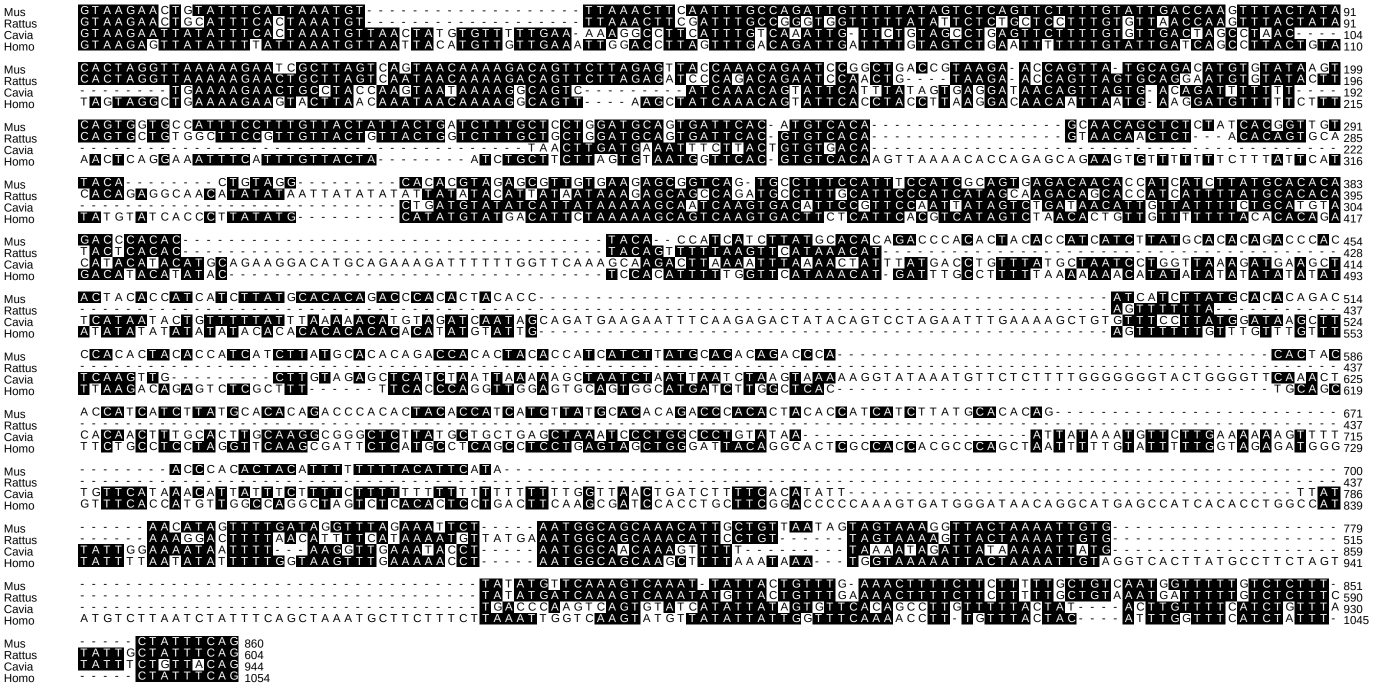
Mus
Rattus
Cavia
Homo

ENSMUSG00000021171 intron 20

Description: Extended synaptotagmin-2 (Esyt2)
Intron number: 20
Mouse chromosome: 12
Upstream exon length: 106
Downstream exon length: 50
Mouse intron length: 1170
Intron alignment length: 1641
Total murinae branch length: 0.21862
K_score: 0.05612
Scaling factor: 0.53278

ENSMUSG00000021171 exon 20 (ORF 1)

```
Mus     CTTCGACTTCAGTGTTTCGCTGCCTGAGGTGCAAAGGAGAACATTGGATGTGGCTGTGAAGAACAGTGGTGGCTTCCTGT 80
Rattus  CTTCGACTTCAGTGTTTCGCTGCCCGAGGTGCAGAGGAGAACACTGGATGTGGCGGTGAAGAACAGTGGCGGCTTCTTGT 80
Cavia   CTTTGATTTCAACGTATCACTCCCTGAGGTGCAGAGGAGAACGCTGGATGTAGCTGTGAAGAACAGTGGCGGCTTCCTGT 80
Homo    CTTTGATTTCAGTGTTTCGTTACCAGAAGTGCAGAGGAGAACGCTCGACGTTGCCGTGAAGAACAGTGGCGGCTTCCTGT 80

Mus     CCAAAGATAAAGGGCTTCTTGGCAAA 106
Rattus  CCAAAGACAAAGGGCTTCTTGGCAAA 106
Cavia   CCAAAGATAAAGGGCTTCTTGGCAAA 106
Homo    CCAAAGACAAAGGGCTCCTTGGCAAA 106
```

ENSMUSG00000021171 exon 21 (ORF 0)

```
Mus     GTGCTGGTTGTTCTGGCATCTGAAGAACTCGCCAAGGGCTGGACCCAGTG 50
Rattus  GTGCTGGTTGCTCTGGCATCTGAAGAACTCGCCAAGGGCTGGACCCAGTG 50
Cavia   GTGCTGGTGGCTCTGGCATCTGAAGAGCTCGCCAAGGGCTGGACCCAGTG 50
Homo    GTATTGGTTGCTCTGGCATCTGAAGAACTTGCCAAAGGCTGGACCCAGTG 50
```

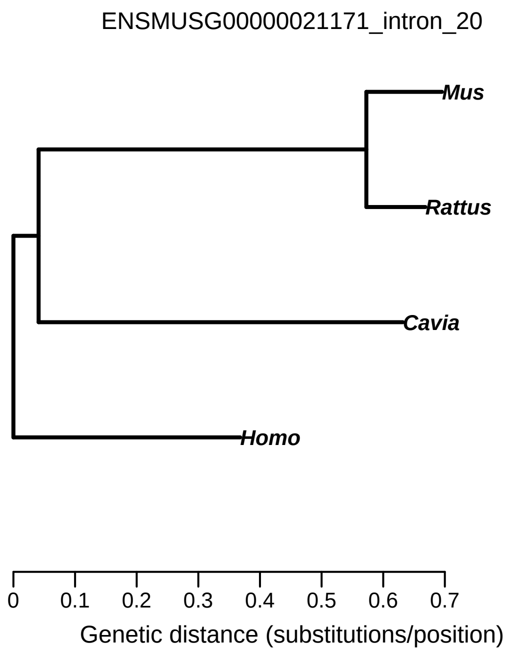

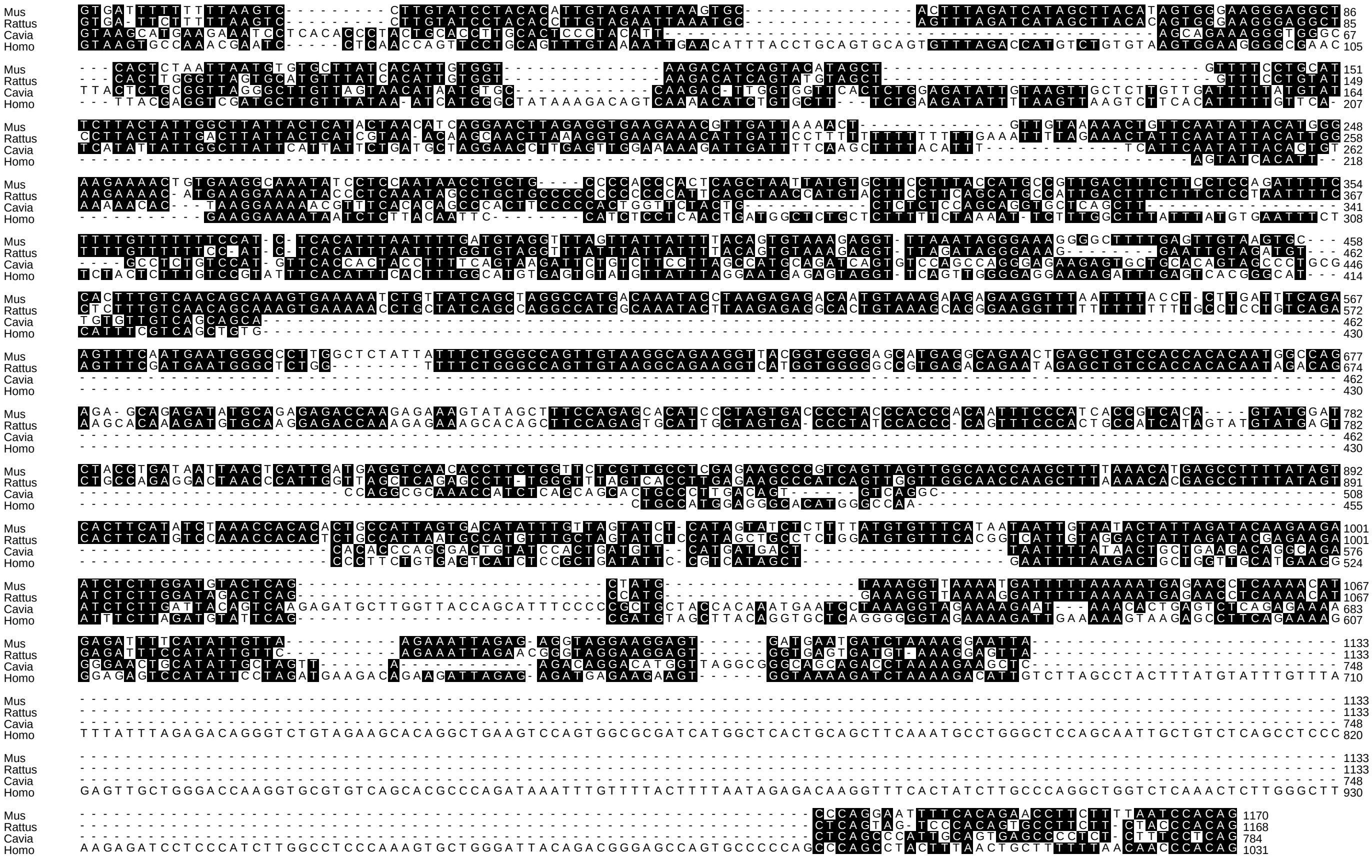

ENSMUSG00000039263 intron 9

Description: Probable aminopeptidase NPEPL1 (Npepl1)
Intron number: 9
Mouse chromosome: 2
Upstream exon length: 124
Downstream exon length: 177
Mouse intron length: 280
Intron alignment length: 388
Total murinae branch length: 0.26144
K_score: 0.04856
Scaling factor: 0.53744

ENSMUSG00000039263 exon 9 (ORF 1)

```
Mus     GACCGTAGAGATCAACAACACGGATGCTGAGGGCAGGCTGGTGCTGGCAGACGGGGTGTCTTACGCTTGCAAAGACCTGG 80
Rattus  GACGGTAGAGATCAACAACACGGACGCTGAGGGCAGGCTGGTGCTGGCAGACGGGGTGTCTTATGCTTGCAAAGACCTGG 80
Cavia   GACTGTGGAAATCAACAACACAGATGCCGAGGGCAGGCTGGTGCTGGCCGATGGTGTTGCTTATGCCTGTAAAGACCTGG 80
Homo    GACGGTGGAAATCAACAACACGGATGCCGAGGGCAGGCTGGTGCTGGCAGATGGCGTGTCCTATGCTTGCAAGGACCTGG 80

Mus     GGGCTGACATCATCGTGGACATGGCCACCCTGACAGGGGCACAA 124
Rattus  GTGCTGACATCATTGTGGACATGGCCACCCTGACAGGGGCACAG 124
Cavia   GCGCTGACATCATCCTGGACATGGCCACGCTGACCGGGGCGCAG 124
Homo    GGGCCGACATCATCCTGGACATGGCCACCCTGACCGGGGCTCAG 124
```

ENSMUSG00000039263 exon 10 (ORF 0)

```
Mus     GGCATTGCCACAGGGAAGTACCATGCAGCCGTGCTCACCAACAGCGCCGAGTGGGAGGCGGCCTGCGTGAAGGCTGGCCG 80
Rattus  GGCATTGCCACAGGGAAATACCATGCAGCCGTGCTCACCAACAGCGCCGAGTGGGAGGCGGCCTGTGTGAAGGCTGGGCA 80
Cavia   GGCATCGCCACTGGGAAGTACCACGCTGCGGTGCTCACCAACAACGCCGAGTGGGAAGCTGCCTGCGTGAAGGCCGGCCG 80
Homo    GGCATTGCCACAGGGAAGTACCACGCCGCGGTGCTCACCAACAGCGCTGAGTGGGAGGCCGCCTGTGTGAAGGCGGGCAG 80

Mus     GAAGTGCGGGGACCTGGTCCACCCGCTAGTCTACTGCCCTGAGCTGCACTTCAGCGAATTCACCTCGGCTGTGGCTGACA 160
Rattus  GAAGTGCGGGGACCTGGTCCACCCGCTGGTCTACTGCCCTGAGCTGCACTTCAGCGAGTTCACCTCAGCTGTGGCTGACA 160
Cavia   GCAGTGCGGGGACCTGGTGCACCCCCTGGTCTACTGCCCCGAGCTGCACTTCAGCGAGTTCACTTCGGCCGTGGCTGACA 160
Homo    GAAGTGTGGGGACCTGGTGCACCCGCTGGTCTACTGCCCCGAGCTGCACTTCAGCGAGTTCACCTCAGCTGTGGCGGACA 160
```

ENSMUSG00000039263_intron_9

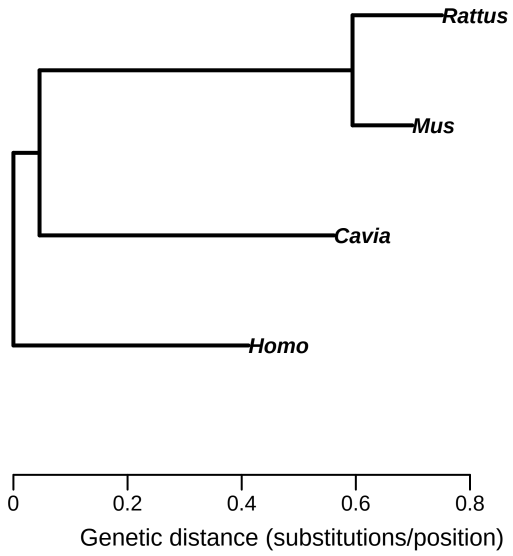

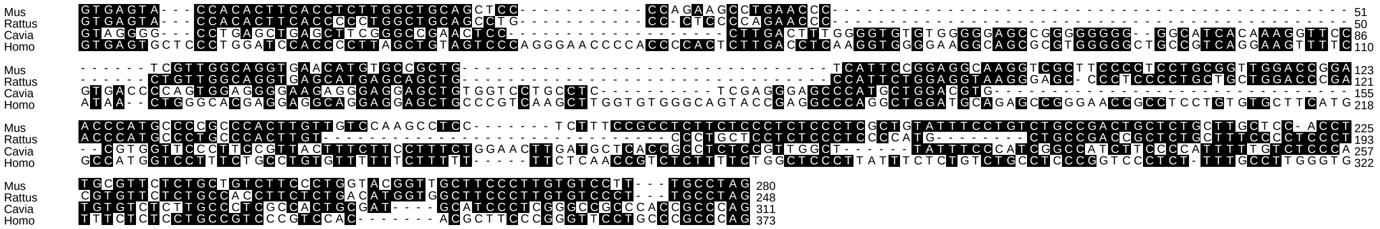
Mus
Rattus
Cavia
Homo

ENSMUSG00000003759 intron 9

Description: SH3KBP1-binding protein 1
Intron number: 9
Mouse chromosome: 7
Upstream exon length: 191
Downstream exon length: 116
Mouse intron length: 1088
Intron alignment length: 1620
Total murinae branch length: 0.14486
K_score: 0.08771
Scaling factor: 0.53832

ENSMUSG00000003759 exon 9 (ORF 1)

```
Mus     TTCTCCAGCCCGCGCCTGGACTGGCCCATCGAGCGCCTGGCTCTTGCGGCTCGGGTGCTTGGCGGTGCTCCGGGTGAGCA 80
Rattus  TTCTCCAGTCCTCGCCTAGACTGGCCCATTGAGCGCCTGGCTCTCACAGCTCGGGTGCTCGGTGGTGCTCCAGGCGAGCA 80
Cavia   TTTTCCAGCCCCCGCCTGGACTGGCCCATTGAACGCCTGGCTCTTACAGCCCGGGTACCTGGTGGGGCTTTGGGCGAGCA 80
Homo    TTTTCCAGCCCCCGCCTGGACTGGCCCATCGAACGACTGGCGCTCACAGCCCGGGTGCATGGTGGGGCTTTGGGTGAACA 80

Mus     TGACAAGATGGTGGCTGCAGCTACTGGCAGCGAGATCCTTCTGTGGGCCCTACAAGCACAAGGTGGTGGCTCTGAGATAG 160
Rattus  TGACAAGATGGTGGCTGCAGCCACAGGCAGCGAGATCCTTCTGTGGGCGCTACAGGCACAAGGTGGTGGCTCTGAGATAG 160
Cavia   TGACAAGATGGTAGCTGCAGCCACTGGCAGTGAGATCCTGTTGTGGGCTCTGCAGCCAGAAGGTGGTGGCTCTGAGATAG 160
Homo    TGACAAGATGGTGGCAGCAGCCACCGGCAGCGAGATCCTGCTATGGGCTCTGCAGGCGGAAGGCGGTGGCTCCGAGATAG 160
```

ENSMUSG00000003759 exon 10 (ORF 2)

```
Mus     GTGTCTTCCACCTGGGTGTGCCAGTGGAGGCCTTGTTCTTTGTTGGGAATCAGCTCATTGCCACAAGTCACACAGGGCGC 80
Rattus  GTGTCTTCCACCTGGGTGTGCCTGTGGAAGCCTTGTTCTTTGTTGGGAACCAGCTCATTGCCACAAGCCACACAGGGCGC 80
Cavia   GAGTCTTCCATCTAGGGGTACCTGTGGAGGCTTTGTTCTTCGTGGGGAACCAGCTCATTGCCACGAGCCACACTGGGCGC 80
Homo    GGGTCTTTCATCTGGGGGTGCCTGTGGAGGCCTTGTTCTTCGTCGGGAACCAGCTCATTGCTACAAGCCACACAGGGCGC 80

Mus     ATCGGTGTCTGGAACGCTGTGACCAAGCACTGGCAG 116
Rattus  ATCGGTGTCTGGAACGCCGTGACCAAGCACTGGCAG 116
Cavia   ATTGGAGTGTGGAATGCTGTCACCAAGCACTGGCAG 116
Homo    ATCGGGGTGTGGAATGCCGTCACCAAGCACTGGCAG 116
```

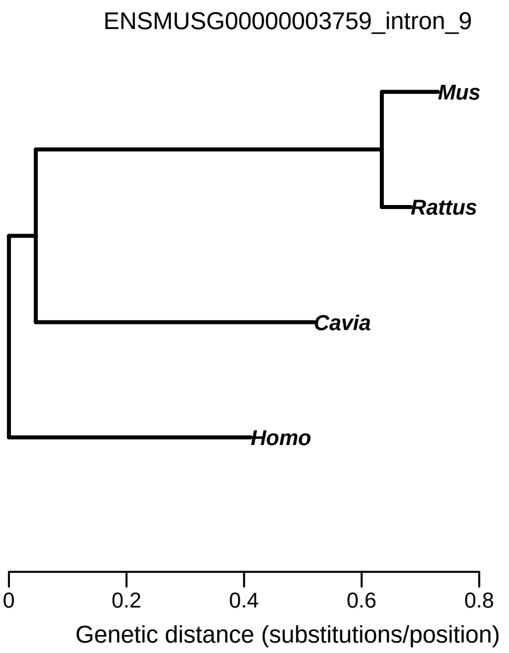

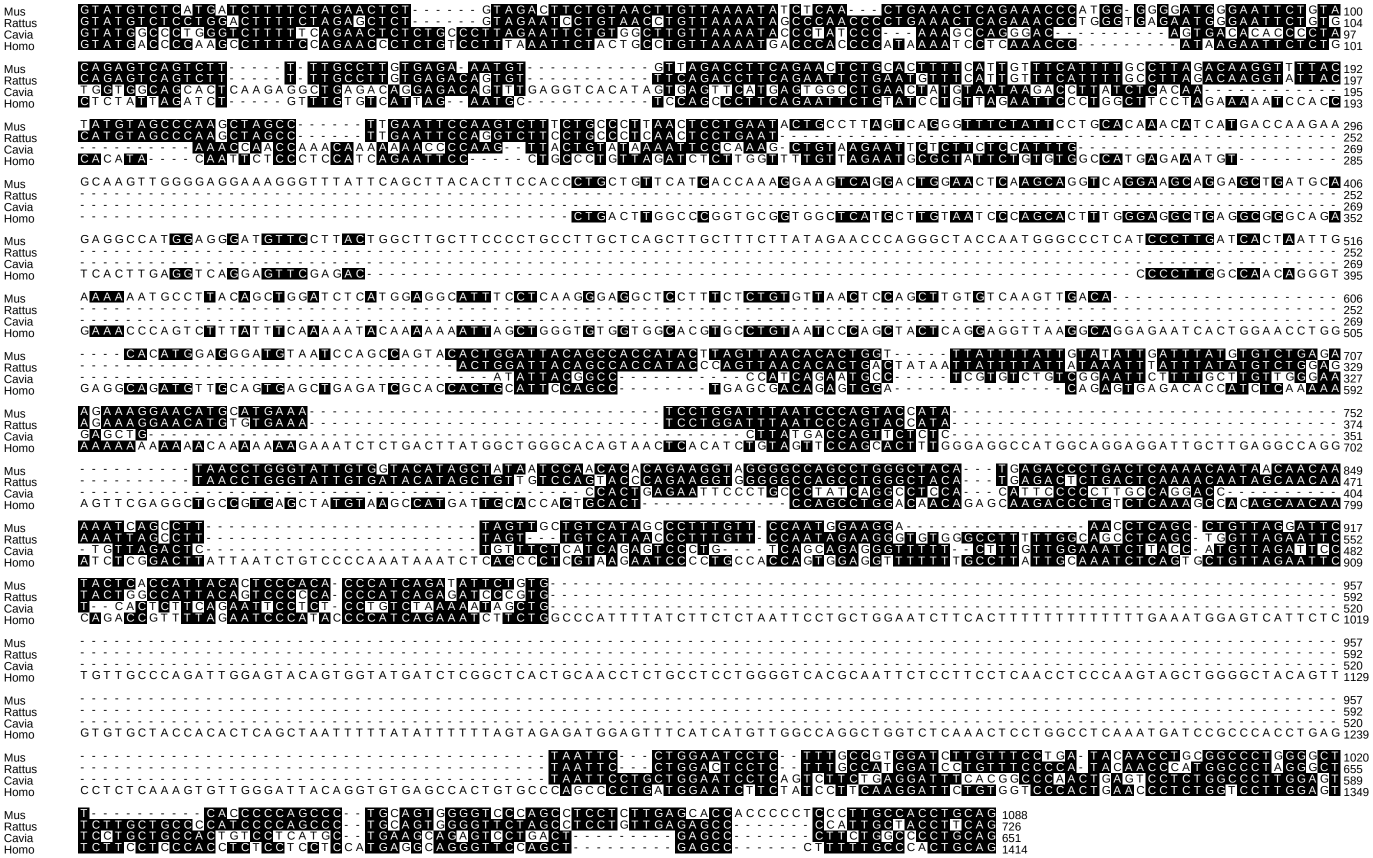
Mus
Rattus
Cavia
Homo

ENSMUSG00000028532 intron 15

Description: VWFA and cache domain-containing protein 1 Precursor (Cachd1)
Intron number: 15
Mouse chromosome: 4
Upstream exon length: 182
Downstream exon length: 111
Mouse intron length: 1385
Intron alignment length: 1934
Total murinae branch length: 0.19311
K_score: 0.06399
Scaling factor: 0.54129

ENSMUSG00000028532 exon 15 (ORF 0)

```
Mus     TGGCGACCAGCCACGTCACAGATGAATGGATGACACAAATGGAAATGAGCAGCCTGAACACGTACATTGTCCGCCGTTAC 80
Rattus  TGGCGACCAGCCACGTCACAGATGAATGGATGACACAAATGGAAATGAGCAGCCTGAACACGTACATTGTCCGCCGTTAC 80
Cavia   TGGCGACCAGCCACGTCACTGATGAATGGATGACACAGATGGAAATGAGCAGCCTGAACACTTACATTGTTCGCCGTTAC 80
Homo    TGGCTACCAGCCACGTCACAGATGAATGGATGACACAAATGGAAATGAGTAGCCTGAACACTTACATTGTCCGCCGTTAC 80

Mus     ATAGCAACACCCAATGGCGTCCTCAGAATTTATCCTGGTTCCCTTATGGACAAAGCATTTGATCCCACTAGGAGACAGTG 160
Rattus  ATAGCAACACCCAATGGCGTCCTCAGAATTTATCCTGGTTCCCTCATGGACAAAGCATTTGATCCCACGAGGAGACAGTG 160
Cavia   ATAGCAACGCCCAACGGTGTCCTCAGAATTTACCCTGGCTCCCTCATGGACAAAGCCTTCGACCCGACCCGCAGACAGTG 160
Homo    ATAGCAACACCCAATGGCGTCCTCAGAATTTATCCTGGTTCCCTCATGGACAAAGCATTTGATCCCACTAGGAGACAATG 160
```

ENSMUSG00000028532 exon 16 (ORF 1)

```
Mus     GTATCTTCATGCAGTAGCCAATCCAGGGCTGATTTCCTTGACGGGCCCTTACCTGGATGTTGGAGGAGCTGGCTATGTCG 80
Rattus  GTATCTTCATGCAGTAGCCAATCCAGGGCTGATTTCCTTGACGGGCCCTTACCTAGATGTTGGAGGAGCTGGCTATGTCG 80
Cavia   GTACCTTCATGCCGTGGCCAACCCAGGCTTGATTTCCCTGACCGGCCCTTACCTGGACGTCGGTGGAGCCGGCTACGTCG 80
Homo    GTATCTCCATGCAGTAGCTAATCCAGGGTTGATTTCTTTGACTGGTCCTTACTTAGATGTTGGAGGAGCTGGTTATGTTG 80

Mus     TGACAATCAGCCACACAATTCACTCATCCAG 111
Rattus  TGACAATCAGCCACACAATTCACTCATCCAG 111
Cavia   TGACCATCAGCCACACCATCCACTCGTCAAG 111
Homo    TGACAATCAGTCACACAATTCATTCATCCAG 111
```

ENSMUSG00000028532_intron_15

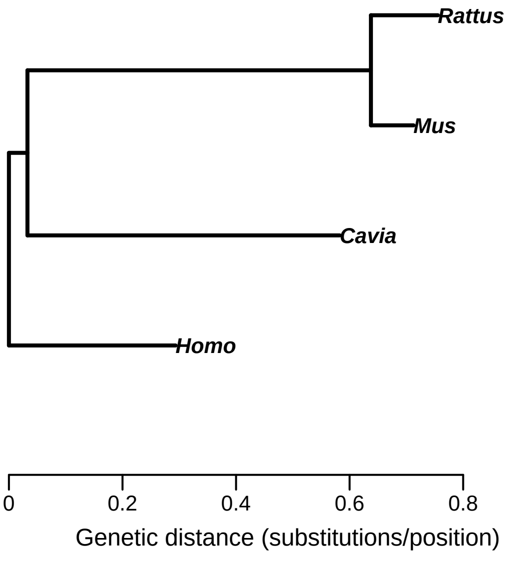

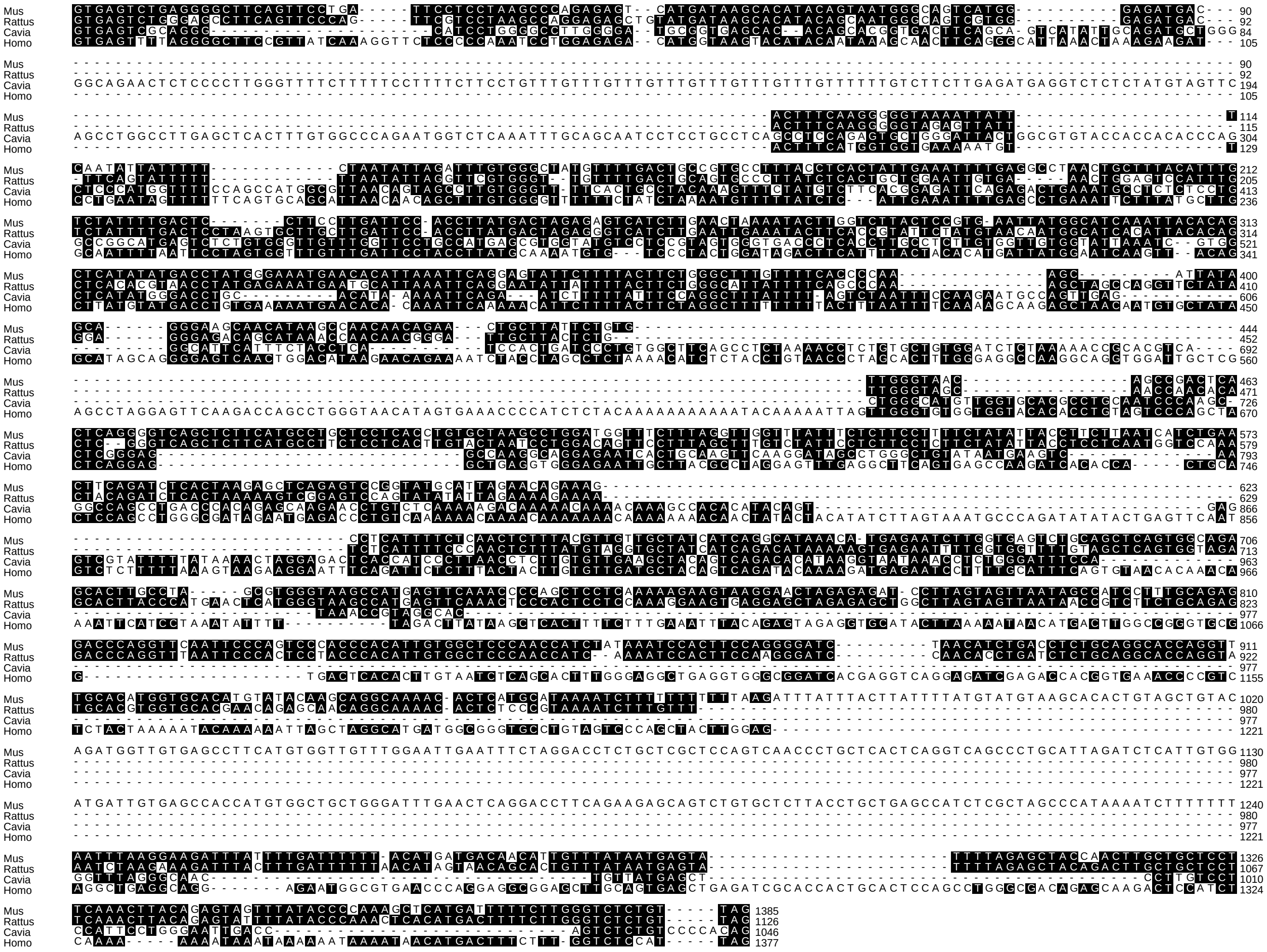
Mus
Rattus
Cavia
Homo

ENSMUSG00000024911 intron 9

Description: Acidic fibroblast growth factor intracellular-binding protei (Fibp)
Intron number: 9
Mouse chromosome: 19
Upstream exon length: 98
Downstream exon length: 70
Mouse intron length: 493
Intron alignment length: 626
Total murinae branch length: 0.30319
K_score: 0.01867
Scaling factor: 0.54739

ENSMUSG00000024911 exon 9 (ORF 0)

```
Mus     TTTGTGGAACCCTGCCGCTCTGACCACTGGCCACTGAGTGATGTGCGGCTCTTCCTCAGCCAGTATTCAGCGTCAGTCCA 80
Rattus  TTTGTGGAACCCTGCCGCTCTGACCACTGGCCACTGAGTGACGTGAGGCTCTTCCTCAACCAGTATTCAGCGTCTGTCCA 80
Cavia   TTTGTGGAACCCTGCCGCTCCGACCACTGGCCACTGGCTGATGTGCGGCTCTTCCTGAATCAGTATTCAGCATCTGTCCA 80
Homo    TTTGTGGAACCCTGCCGCTCCGACCACTGGCCACTCAGCGACGTGCGGTTCTTCCTGAATCAGTATTCAGCGTCTGTCCA 80

Mus     CTCCCTGGATGGCTTCCG 98
Rattus  CTCCTTGGATGGCTTCCG 98
Cavia   TTCCCTGGATGGCTTCCG 98
Homo    CTCCCTCGATGGCTTCCG 98
```

ENSMUSG00000024911 exon 10 (ORF 1)

```
Mus     GCACCAGGCACTCTGGGACCGCTACATGGGCACCCTCCGTGGCTGCCTTCTGCGCCTCTATCATGATTAA 70
Rattus  GCACCAGGCCCTCTGGGACCGCTACATGGGCACCCTCCGTGGCTGCCTTCTGCGCCTTTATCATGATTAA 70
Cavia   GCACCAGGCCCTCTGGGACCGCTACATGGGCACCCTCCGTGGCTGCCTCCTCCGCCTCTATCATGACTAA 70
Homo    ACACCAGGCCCTCTGGGACCGCTACATGGGCACCCTCCGCGGCTGCCTCCTGCGCCTGTATCATGACTGA 70
```

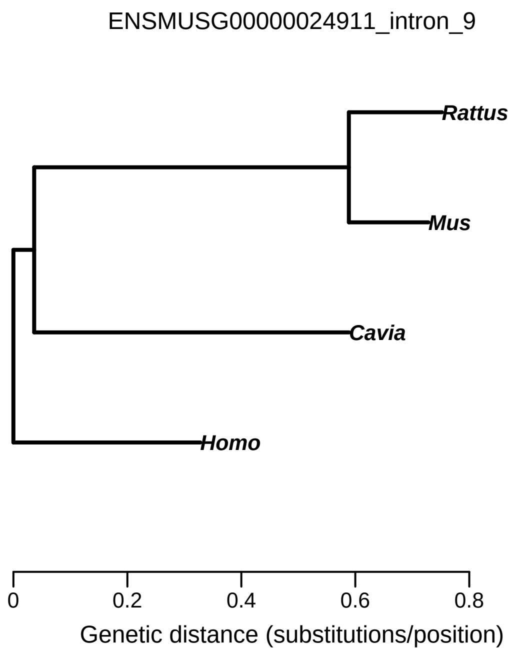

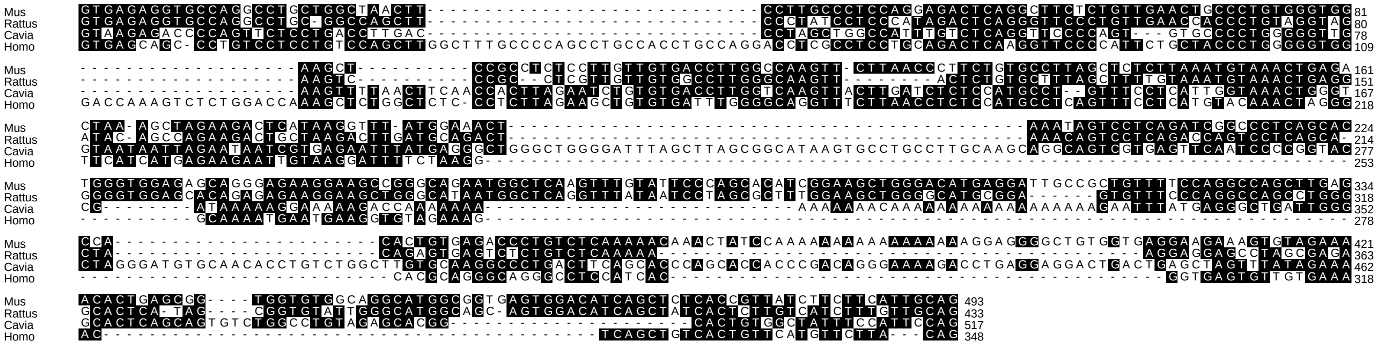
Mus
Rattus
Cavia
Homo
81
80
78
109
161
151
167
218
224
214
277
253
334
318
352
278
421
363
462
318
493
433
517
348

ENSMUSG00000038387 intron 4

Description: Ras-related protein R-Ras Precursor (Rras)
Intron number: 4
Mouse chromosome: 7
Upstream exon length: 109
Downstream exon length: 119
Mouse intron length: 488
Intron alignment length: 1140
Total murinae branch length: 0.14869
K_score: 0.07154
Scaling factor: 0.55008

ENSMUSG00000038387 exon 4 (ORF 1)

```
Mus     TTTCAATGAGGTGGGCAAGCTCTTCACACAGATCCTCAGAGTCAAGGACCGGGATGATTTCCCCATTGTGTTGGTTGGGA 80
Rattus  TTTCATTGAAGTAAGCAAGCTCTTCACTCAGATCCTCAGGGTCAAGGACCGCGATGACTTCCCCATTGTGCTGGTTGGGA 80
Cavia   CTTCAACGAGGTGAGCAAGCTCTTCACACAGATCCTCCGAGTCAAGGATCGAGATGATTTCCCCGTTGTGCTGGTTGGGA 80
Homo    TTTCAACGAGGTGGGCAAGCTCTTCACGCAGATTCTGCGGGTCAAGGACCGCGACGACTTCCCCGTTGTGTTGGTCGGGA 80

Mus     ACAAGGCAGATCTGGAGAACCAGCGCCAG 109
Rattus  ACAAGGCAGATCTGGAGACACAGCGCCAG 109
Cavia   ACAAGGCAGATCTGGAAGCACAGCGGCAG 109
Homo    ACAAGGCAGATCTGGAGTCACAGCGCCAG 109
```

ENSMUSG00000038387 exon 5 (ORF 0)

```
Mus     GTCCTCCGGTCTGAAGCCTCCTCTTTCAGTGCTTCCCACCACATGACTTACTTTGAGGCCTCAGCCAAACTGCGTCTGAA 80
Rattus  GTCCTTCGATCAGAGGCCTCCTCCTTTAGTGCTTCCCACCATATGACTTACTTTGAGGCCTCAGCCAAACTGCGTCTAAA 80
Cavia   GTCCCCCGATCTGAAGCCTCCAACTTTGGGGCCTCCCACCATGTGGCCTACTTTGAGGCCTCTGCCAAACTGAGGCTCAA 80
Homo    GTCCCCCGATCAGAAGCCTCTGCCTTCGGCGCCTCCCACCACGTGGCCTACTTTGAGGCCTCGGCCAAACTGCGTCTCAA 80

Mus     TGTCGATGAGGCATTTGAGCAGCTGGTGCGCGCTGTCCG 119
Rattus  TGTCGATGAGGCATTTGAGCAGCTGGTGCGGACTGTCCG 119
Cavia   CGTGGACGAGGCCTTCGAGCAGCTGGTGCGGGCTGTCCG 119
Homo    CGTGGACGAGGCTTTTGAGCAGCTGGTGCGGGCTGTCCG 119
```

ENSMUSG00000038387_intron_4

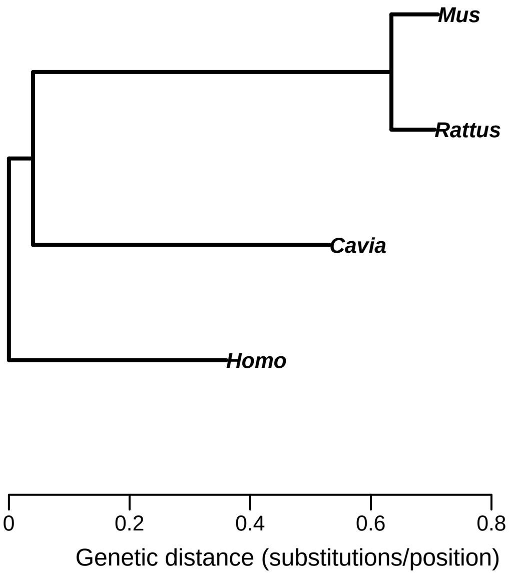

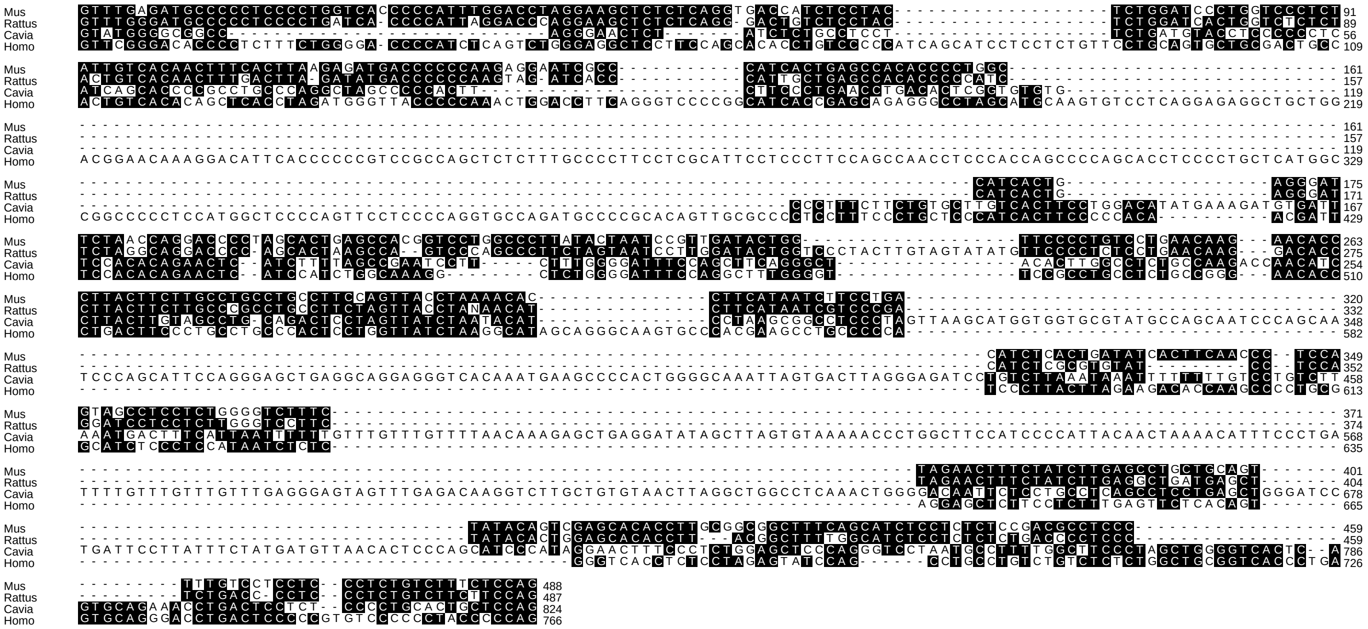
Mus
Rattus
Cavia
Homo

ENSMUSG00000031349 intron 6

Description: Sterol-4-alpha-carboxylate 3-dehydrogenase, decarboxylating (Nsdhl)
Intron number: 6
Mouse chromosome: X
Upstream exon length: 103
Downstream exon length: 333
Mouse intron length: 842
Intron alignment length: 1541
Total murinae branch length: 0.17556
K_score: 0.08711
Scaling factor: 0.55452

ENSMUSG00000031349 exon 6 (ORF 1)

```
Mus     AAATGGGGAAAACCTGGTGGACTTCACCTTCGTGGAGAATGTGGTTCATGGACACATCTTAGCCGCTGAGCACCTCTCCC 80
Rattus  AAATGGGAAGAACCTGGTGGACTTCACCTTCGTGGAGAATGTAGTTCATGGACACATCTTAGCAGCTGAGCACCTCTCTC 80
Cavia   AAATGGGCAGAACCTGGTGGACTTCACCTTTGTGGAGAACGTGGCCCATGGTCACATCCTGGCGGCAGAGCACCTCTCCC 80
Homo    AAATGGGAAGAACTTGGTGGACTTCACCTTTGTGGAGAACGTGGTCCATGGACACATCCTGGCGGCAGAGCAGCTCTCCC 80

Mus     AAGATGCAGCTCTAGGTGGAAAG 103
Rattus  GAGATGCAGGTCTGGGTGGGAAG 103
Cavia   GAGACGCCCCACTGTGCGGAAAG 103
Homo    GAGACTCGACACTGGGTGGGAAG 103
```

ENSMUSG00000031349 exon 7 (ORF 0)

```
Mus     GCATTTCACATCACCAACGATGAACCAATCCCTTTCTGGACGTTCCTGTCCCGCATTCTGACAGGCCTCAATTATGAGGC 80
Rattus  GCATTTCACATCACCAACGATGAGCCAATCCCTTTCTGGACGTTCCTGTCCCGCATTCTGACAGGCCTCAATTATGAGGC 80
Cavia   GCGTTTCACATCACCAATGACGAGCCCATCCCTTTCTGGACCTTCCTGTCCCGCATCCTGAGCGGCCTCAATTATGAGCC 80
Homo    GCATTTCACATCACCAATGATGAGCCCATCCCTTTCTGGACATTCCTGTCTCGCATCCTGACAGGCCTCAATTATGAGGC 80

Mus     CCCTAAGTACCACATCCCCTACTGGATGGCCTATTACCTTGCTTTCCTGCTATCTCTACTGGTGATGGTGGTCAGCCCTC 160
Rattus  CCCCAAGTACCACATCCCCTACAGGGTGGCCTACTATCTTGCTTTCCTGCTATCTCTACTGGTAATGGTGCTCAGCCCTC 160
Cavia   CCCCAAGTACCATATCCCCTACTGGGTGGCCTACTACCTGGCCCTCCTGGTGTCCCTGCTGGTGACACTGGTCAGTCCCA 160
Homo    CCCCAAGTACCACATCCCCTACTGGGTGGCCTACTACCTGGCCCTCCTGCTATCCCTGCTGGTGATGGTGATCAGTCCTG 160
```

ENSMUSG00000031349_intron_6

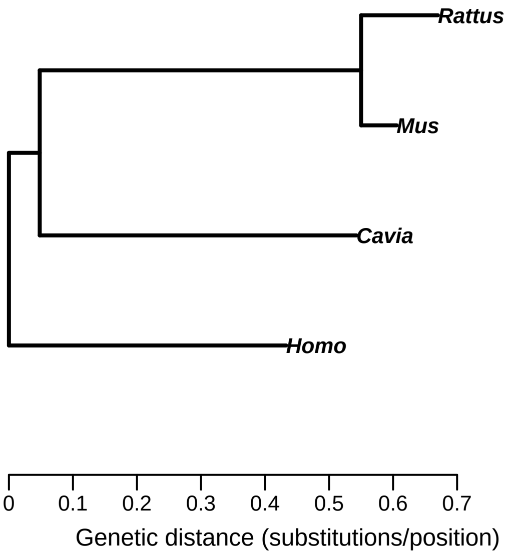

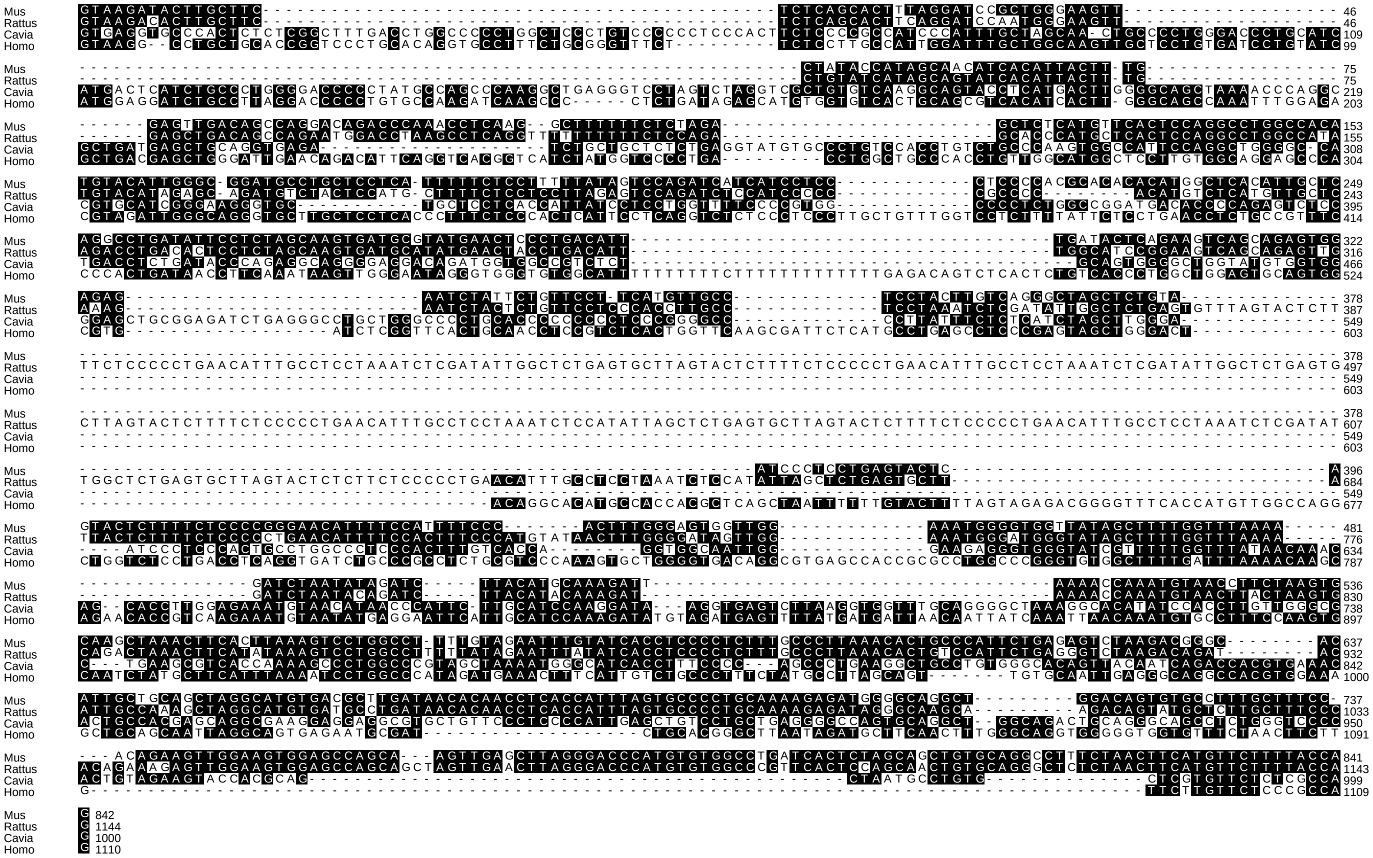

ENSMUSG00000032115 intron 13

Description: Hypoxia up-regulated protein 1 Precursor (Hyou1)
Intron number: 13
Mouse chromosome: 9
Upstream exon length: 139
Downstream exon length: 61
Mouse intron length: 1326
Intron alignment length: 1700
Total murinae branch length: 0.24623
K_score: 0.06876
Scaling factor: 0.55665

ENSMUSG00000032115 exon 13 (ORF 1)

```
Mus     GGTATTTGGCTCCCAGAATCTGACCACAGTAAAACTAAAAGGCGTGGGAGAGAGCTTCAAGAAATATCCCGACTATGAGT 80
Rattus  GGTATTTGGCTCCCAGAATCTGACCACAGTGAAACTAAAAGGTGTGGGAGAGAGCTTCAAGAAATATCCTGACTATGAGT 80
Cavia   GGTATTTGGCTCTCAGAATCTGACCACAGTGAAACTAAAAGGTGTGGGTGACAGCTTTAAGAAGTATCCCGACTACGAGT 80
Homo    GGTATTTGGCTCCCAGAATCTGACCACAGTGAAGCTAAAAGGGGTGGGTGACAGCTTCAAGAAGTATCCTGACTACGAGT 80

Mus     CCAAAGGCATCAAGGCCCACTTTAACCTGGATGAGAGTGGCGTGCTCAGTTTAGACAGG 139
Rattus  CCAAAGGCATCAAGGCCCACTTTAACCTAGACGAGAGTGGAGTGCTCAGTTTAGACAGG 139
Cavia   CCAAGGGCATTAAGGCACACTTCAATCTGGATGAGAGTGGTGTGCTCAGTCTGGACAGG 139
Homo    CCAAGGGCATCAAGGCTCACTTCAACCTGGATGAGAGTGGCGTGCTCAGTCTAGACAGG 139
```

ENSMUSG00000032115 exon 14 (ORF 0)

```
Mus     GTGGAGTCCGTATTTGAGACCCTGGTGGAGGATAGCCCAGAGGAAGAATCTACTCTTACCA 61
Rattus  GTGGAGTCCGTATTCGAGACCCTGGTGGAGGACAGCCCAGAGGAAGAGTCTACTCTTACCA 61
Cavia   GTAGAGTCTGTATTTGAGACCCTGGTGGAAGACAGCCCAGAGGAAGAATCTACTCTCACCA 61
Homo    GTGGAGTCTGTATTTGAGACACTGGTAGAGGACAGCGCAGAAGAGGAATCTACTCTCACCA 61
```

ENSMUSG00000032115_intron_13

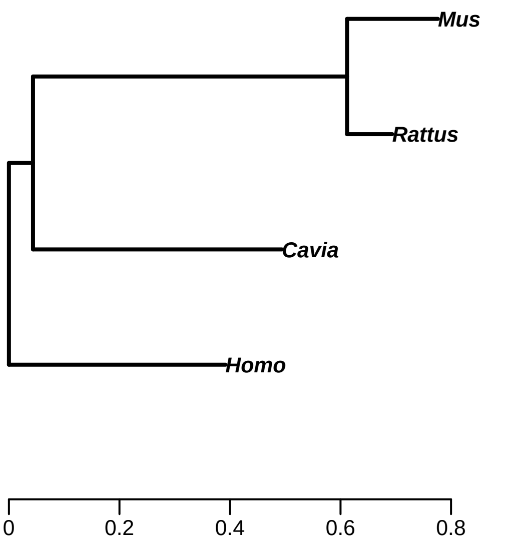

Genetic distance (substitutions/position)

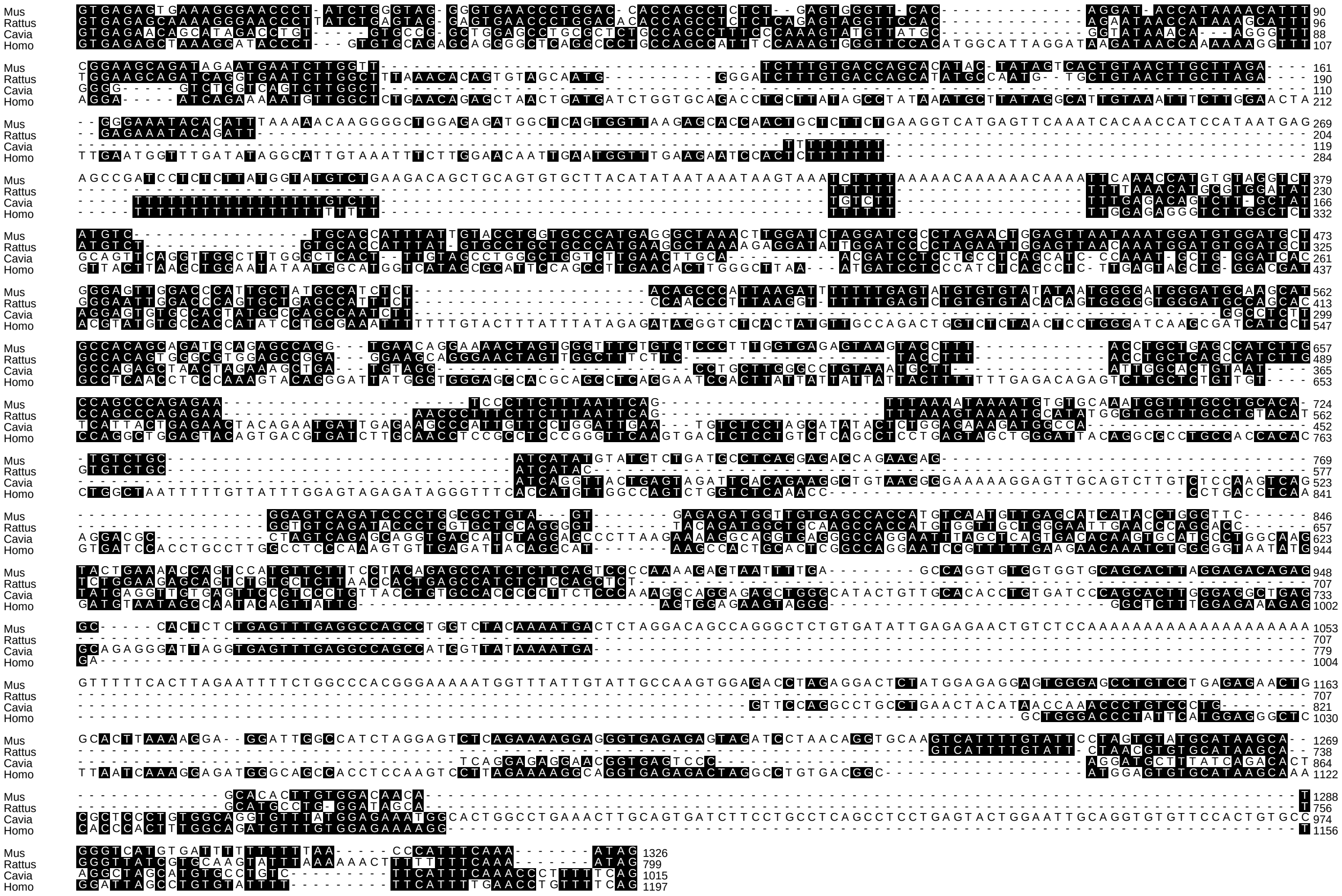

ENSMUSG00000024382 intron 4

Description: TFIIH basal transcription factor complex helicase XPB subuni (Ercc3)
Intron number: 4
Mouse chromosome: 18
Upstream exon length: 50
Downstream exon length: 136
Mouse intron length: 640
Intron alignment length: 718
Total murinae branch length: 0.43263
K_score: 0.07292
Scaling factor: 0.56463

ENSMUSG00000024382 exon 4 (ORF 0)

```
Mus     CTGTGCACTGTCAGCTATGGGAAGGTCAAGCTGGTCCTCAAGCACAACAG 50
Rattus  CTGTGCACTGTCAGCTACGGAAAAGTCAAGCTGGTCCTGAAGCACAACAG 50
Cavia   TTGTGTACTGTCAGCTATGGAAAAGTCAAGCTGGTCCTGAAGCACAACAG 50
Homo    TTGTGTACTGTCAGCTATGGAAAAGTCAAGCTGGTCTTGAAGCACAACAG 50
```

ENSMUSG00000024382 exon 5 (ORF 1)

```
Mus     GTACTTTGTTGAAAGTTCCCACCCTGATGTTATCCAGCATCTTCTTCAAGACCCAGTGATCCGGGAATGTCGCTTGAGGA 80
Rattus  GTACTTTGTTGAAAGTTCTCACCCTGATGTCATCCAGCATCTTCTTCAAGACCCAGTAATACGGGAATGTCGCTTGAGGA 80
Cavia   GTACTTCGTTGAAAGCTCCCATCCCGATGTCATCCAGCACCTCCTTCAAGACCCAGTGATCCGGGAGTGCCGCCTGAGGA 80
Homo    ATACTTCGTTGAAAGTTGCCACCCTGATGTAATCCAGCATCTTCTCCAGGACCCCGTGATCCGAGAATGCCGCTTAAGAA 80

Mus     ACGCCGAGGGCGAGGCCACCGAACTCATCACAGAGACTTTTACAAGCAAATCTGCT 136
Rattus  ATGCCGAGGGAGAGGCTACTGAACTCATCACAGAGACGTTTACGAGCAAATCTGCT 136
Cavia   ATGCTGAGGGGGAAGCCACTGAACTCATCACAGAGACTTTCACGAGCAAATCTGCT 136
Homo    ACTCTGAAGGGGAGGCCACTGAGCTCATCACAGAGACTTTCACAAGCAAATCTGCC 136
```

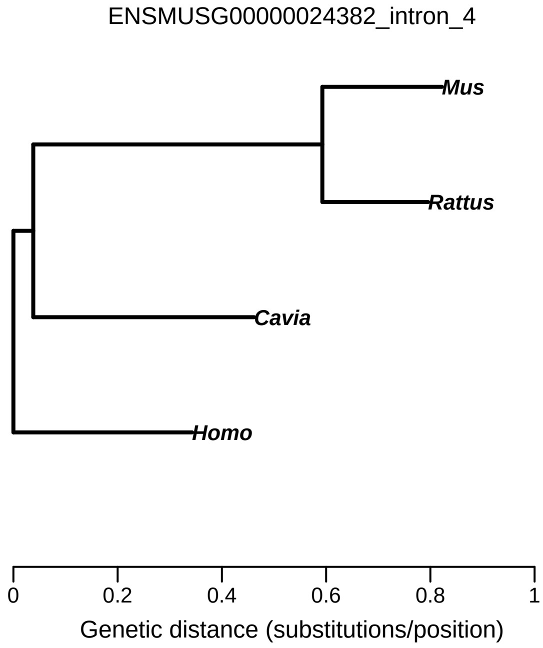

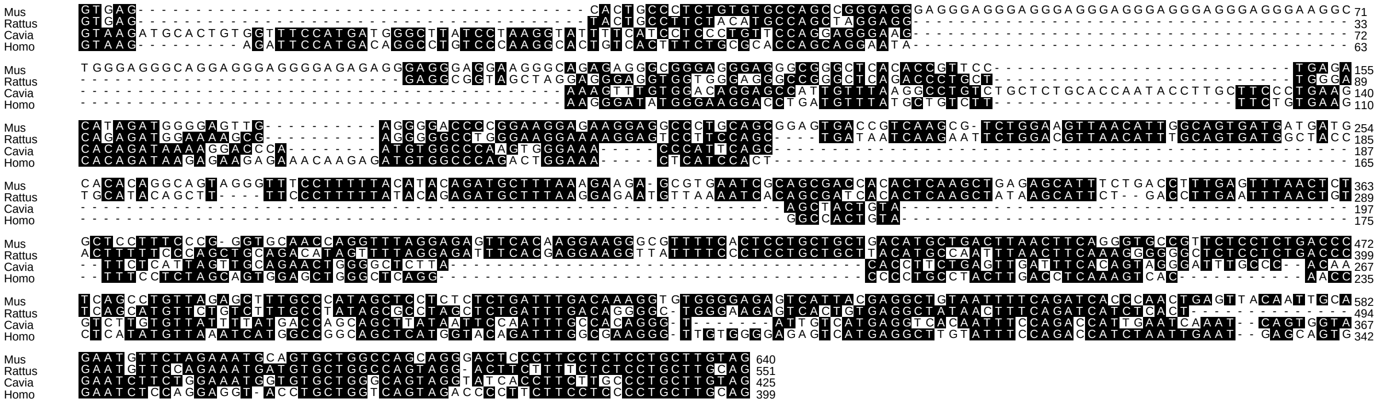
Mus
Rattus
Cavia
Homo

ENSMUSG00000036733 intron 7

Description: RNA-binding protein 42 (Rbm42)
Intron number: 7
Mouse chromosome: 7
Upstream exon length: 333
Downstream exon length: 118
Mouse intron length: 209
Intron alignment length: 248
Total murinae branch length: 0.21614
K_score: 0.07753
Scaling factor: 0.56546

ENSMUSG00000036733 exon 7 (ORF 0)

```
Mus     GGCCCTCGCCATGCCCTTGCCTGAGCCTGAGCCCTTGCCCCTCCCACTGGAGGTGGTACGTGGCCTGCTGCCTCCGCTGA 80
Rattus  GGCCCTAGCCATGCCCTTGCCTGAGCCCGAGCCCTTGCCCCTCCCGCTGGAGGTGGTCCGTGGCCTGCTGCCTCCACTTC 80
Cavia   GGCCCTGGCCATGCCACTGCCTGAGCCTGAGCCTCTGCCCCTCCCACTGGAGGTAGTACGGGGCCTGCTGCCCCCATTGC 80
Homo    GGCCCTGGCCATGCCATTGCCCGAGCCTGAGCCCCTGCCCCTCCCGTTGGAGGTCGTCCGCGGCCTCCTGCCCCCGCTGC 80

Mus     GAATTCCTGAGCTCTTGTCTCTCCGTCCACGACCTCGGCCCCCTCGGCCTGAGCCTCCTCCTGGCCTCATGGCCCTAGAG 160
Rattus  GAATTCCTGAGCTCCTGTCTCTGCGCCCACGACCTCGGCCCCCTCGGCCTGAGCCACCTCCTGGCCTCATGGCCCTAGAG 160
Cavia   GCATTCCTGAGCTCCTGTCCCTTCGTCCACGACCTCGGCCCCCTCGGCCTGAGCCACCCCCCGGCCTCATGGCTCTTGAG 160
Homo    GCATTCCTGAACTCCTGTCCCTGCGTCCTCGGCCCCGGCCCCCTCGGCCAGAGCCACCCCCAGGCCTCATGGCTCTTGAG 160
```

ENSMUSG00000036733 exon 8 (ORF 0)

```
Mus     GTGCCAGAGCCACTGGGTGAAGACAAGAAGAAAGGCAAGCCAGAGAAACTGAAACGCTGCATTCGCACAGCAGCGGGGAG 80
Rattus  GTTCCAGAGCCACTGGGTGAAGACAAGAAGAAAGGCAAGCCAGAGAAATTGAAACGCTGCATTCGTACAGCAGCAGGGAG 80
Cavia   GTGCCAGAGCCCATGGGTGAAGACAAGAAGAAAGGGAAGCCAGAGAAATTGAAACGCTGCATTCGCACAGCAGCCGGAAG 80
Homo    GTCCCAGAGCCCCTGGGTGAAGACAAGAAGAAGGGGAAGCCAGAGAAATTGAAACGGTGCATTCGCACAGCGGCAGGGAG 80

Mus     CAGCTGGGAGGACCCCAGCCTGCTGGAGTGGGATGCCG 118
Rattus  CAGCTGGGAGGACCCCAGCCTGCTGGAGTGGGATGCCG 118
Cavia   CAGCTGGGAGGACCCCAGCCTGTTGGAGTGGGATGCTG 118
Homo    CAGCTGGGAGGACCCCAGCCTGCTGGAGTGGGATGCAG 118
```

ENSMUSG00000036733_intron_7

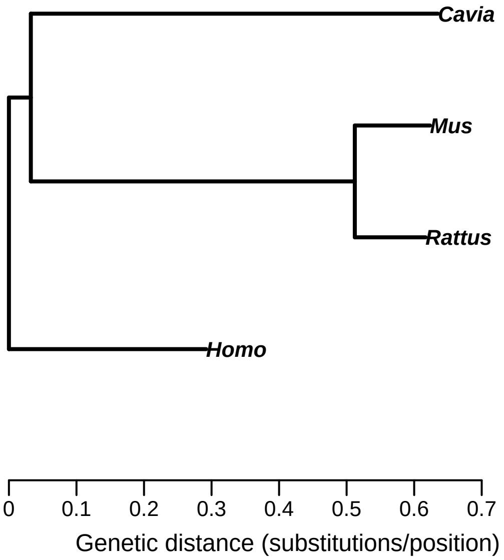

```
Mus     GTAAGCAGGGAATT--GCGGAGAGGGGACAGAAAGGGCCAGGAGAGACCAGAAGCCTATGAG-TTCCACACACAGA--------------------GGGTGCCGGGTGAG 87
Rattus  GTAAGCAGGGAATGT-GCAGAGAGGGGACAGAAAGGGCCAGGAGAAAGCAGGAGCCCATGAGCTTCCACACACAGAACACAGTCCTCTGACCAGAGGGTGGGTGGGAGGG 109
Cavia   GTAAGCAGAGAACATAGCAGTGGGAAGACAG----------------GCAGGAATCTGTGGGCTTTAACACACAGTCCTCTGTCCAGAGCAGAGA-GGACTCAAGGGAGA 93
Homo    GTAAGCAGGGAGCCTAGCGGTGAAGGGACAGAAGGGACGGGGGGCAGACAGGAGCCCAGGAACTTCCACACAGACAGACCGGACAGCAG-------GAGGACCTGGGGGA 103

Mus     GGGAGGCAACCCCTCATACTCCACGTCTGGAAGCAGATCTAGTGCTTGAATGGAAGGTGCCATGGCTGGAGAC-GTTGCAGAGCATGAGACTTCCA-------------- 182
Rattus  GAGAGGCAGCCCCTTACACTCCACATCTGGAAGCGGGTCTAGTACTTGAATCTGAGGTGGCATGGCTGGAGGC-ACTGCAGAGCAGAAGACTTCCA-------------- 204
Cavia   GGGAGGCAGACACGCATACTCTAC--CCCGAAGCATATC--AGTCCTGTTACTGGAGTAAGGGGGACAAGGTCTGAAGCAAAGTGAAAGACTGTAGCATCAAGTCCTTTG 199
Homo    GGGAGGCGGACACATGTGCCCCACATCCAGAAGCACATCCAGCCCTTACTGTGGTGGCAGGAGGGCCGGGGACCAAAGGACAGTGGGAGACCCCAATACCAACCCCTTTG 213

Mus     -ACACCCTTCCCTTCCTTCCCCCTATAG 209
Rattus  -ACAGCCTTCCCTTCCCCC----TGTAG 227
Cavia   TACGCCATCTGCCTTGCTC----TGCAG 223
Homo    CAC-CTCTCCCCTTTCCTC----TGCAG 236
```

ENSMUSG00000030865 intron 1

Description: Calcineurin B homologous protein 2 (2010110P09Rik)
Intron number: 1
Mouse chromosome: 7
Upstream exon length: 67
Downstream exon length: 73
Mouse intron length: 698
Intron alignment length: 847
Total murinae branch length: 0.17131
K_score: 0.07041
Scaling factor: 0.5674

ENSMUSG00000030865 exon 1 (ORF 0)

```
Mus     ATGGGCTCACGAAGCTCTCATATCGCGCTCATTCCAGACGTGGAGCACATTCGCAGAGAGACAGGCT 67
Rattus  ATGGGCTCACGGAGCTCTCATGTCGCGCTCATTCCTGATGTGGACCAAATTCGCAGAGAGACGGGCT 67
Cavia   ATGGGCTCGTGCAGCTCCCACGCCGCGGTCATTCCCGACTTGGACAACATTCGGCGGGAGACTGGCT 67
Homo    ATGGGGTCGCGCAGCTCCCACGCCGCGGTCATTCCCGACGGGGACAGTATTCGGCGAGAGACCGGCT 67
```

ENSMUSG00000030865 exon 2 (ORF 2)

```
Mus     TCTCGCAAGCCAGCCTGCTCCGCCTCTACCACCGGTTCCAAGCCCTGGACAGGGATGAAAAGGGCTTCCTAAG 73
Rattus  TCTCCCAAGCTAGCCTGCTCCGTCTCTACCACAGGTTCCAAGCCCTAGACAGGGAGGAAAAGGGCTTCCTGAG 73
Cavia   TTTCGCAAGCCAGTCTGCTCCGCCTCTACCACCGGTTCCGGGCGCTGGACAGGAACAATAAGGGCTATTTGAG 73
Homo    TCTCCCAAGCCAGCCTGCTCCGCCTGCACCACCGGTTCCGGGCACTGGACAGGAATAAGAAGGGCTACCTGAG 73
```

ENSMUSG00000030865_intron_1

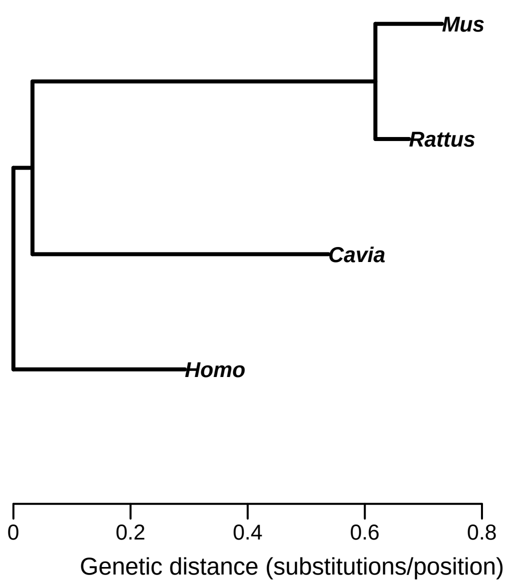

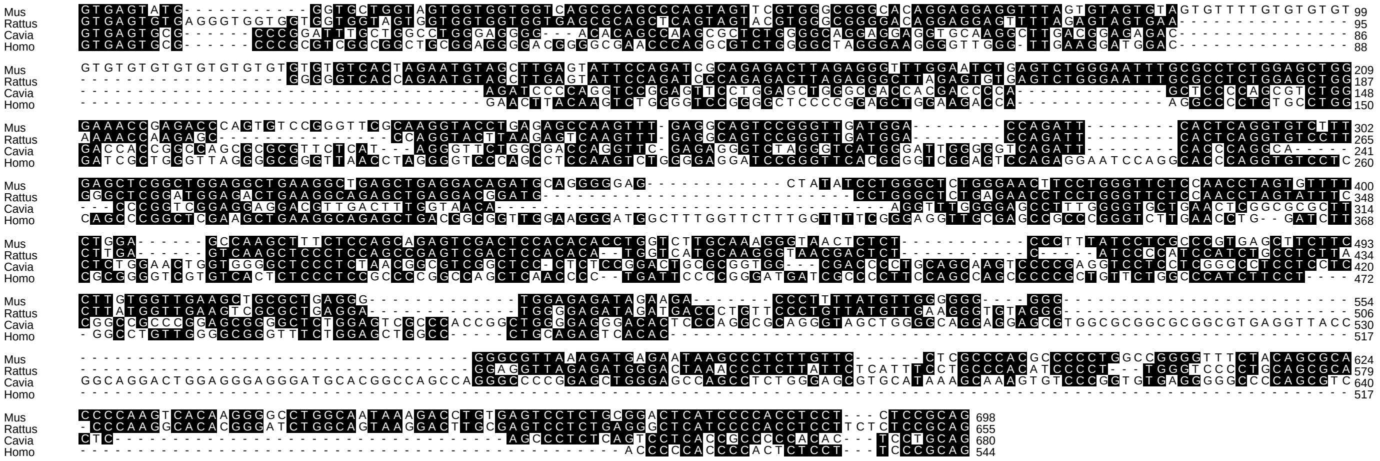
Mus
Rattus
Cavia
Homo

ENSMUSG00000020051 intron 12

Description: Phenylalanine-4-hydroxylase (Pah)
Intron number: 12
Mouse chromosome: 10
Upstream exon length: 116
Downstream exon length: 47
Mouse intron length: 1568
Intron alignment length: 2102
Total murinae branch length: 0.27535
K_score: 0.05589
Scaling factor: 0.56744

ENSMUSG00000020051 exon 12 (ORF 1)

```
Mus     GACTTTTGCTGCCACAATCCCCCGGCCCTTCTCCGTTCGCTATGACCCCTACACTCAAAGGGTTGAGGTCCTGGACAATA 80
Rattus  GACTTTTGCTGCCACGATCCCTCGGCCCTTCTCGGTTCGCTATGACCCTTACACTCAAAGGGTTGAGGTCCTGGACAATA 80
Cavia   GAACTTCGCTGCCACCATCCCCCGGCCCTTCTCAGTTCATTACAACCCCTACACCCAGAGGATCGAGGTCTTGGACAACG 80
Homo    GAACTTTGCTGCCACAATACCTCGGCCCTTCTCAGTTCGCTACGACCCATACACCCAAAGGATTGAGGTCTTGGACAATA 80

Mus     CTCAGCAGTTGAAGATTTTAGCTGACTCCATTAATA 116
Rattus  CTCAGCAGTTGAAGATTTTAGCTGACTCCATTAATA 116
Cavia   CCCAGCAGCTGAAGATCCTGGCGGACTCCATCAACA 116
Homo    CCCAGCAGCTTAAGATTTTGGCTGATTCCATTAACA 116
```

ENSMUSG00000020051 exon 13 (ORF 2)

```
Mus     GTGAGGTTGGAATCCTTTGCCATGCCCTGCAGAAAATAAAGTCATGA 47
Rattus  GTGAGGTCGGAATCCTTTGCAATGCCCTGCAGAAGATAAAGTCGTGA 47
Cavia   GTGAAGTTGGAATCCTTTGCAGTGCTCTGCAGAAAATAAAGT---GA 44
Homo    GTGAAATTGGAATCCTTTGCAGTGCCCTCCAGAAAATAAAGT---AA 44
```

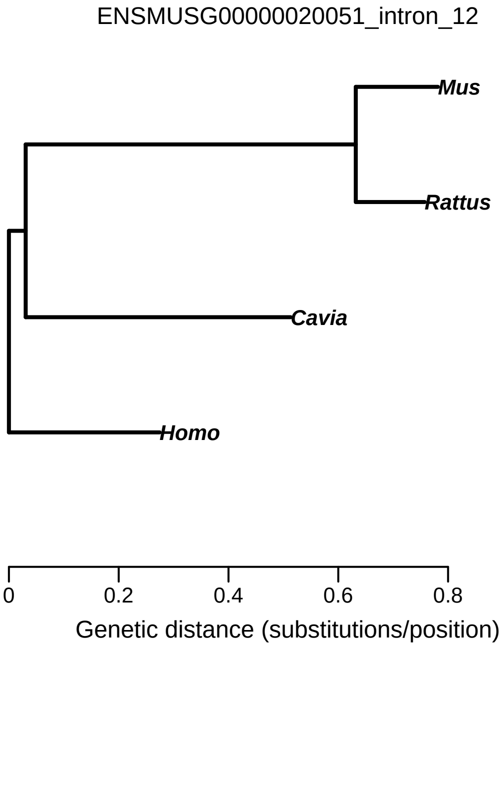

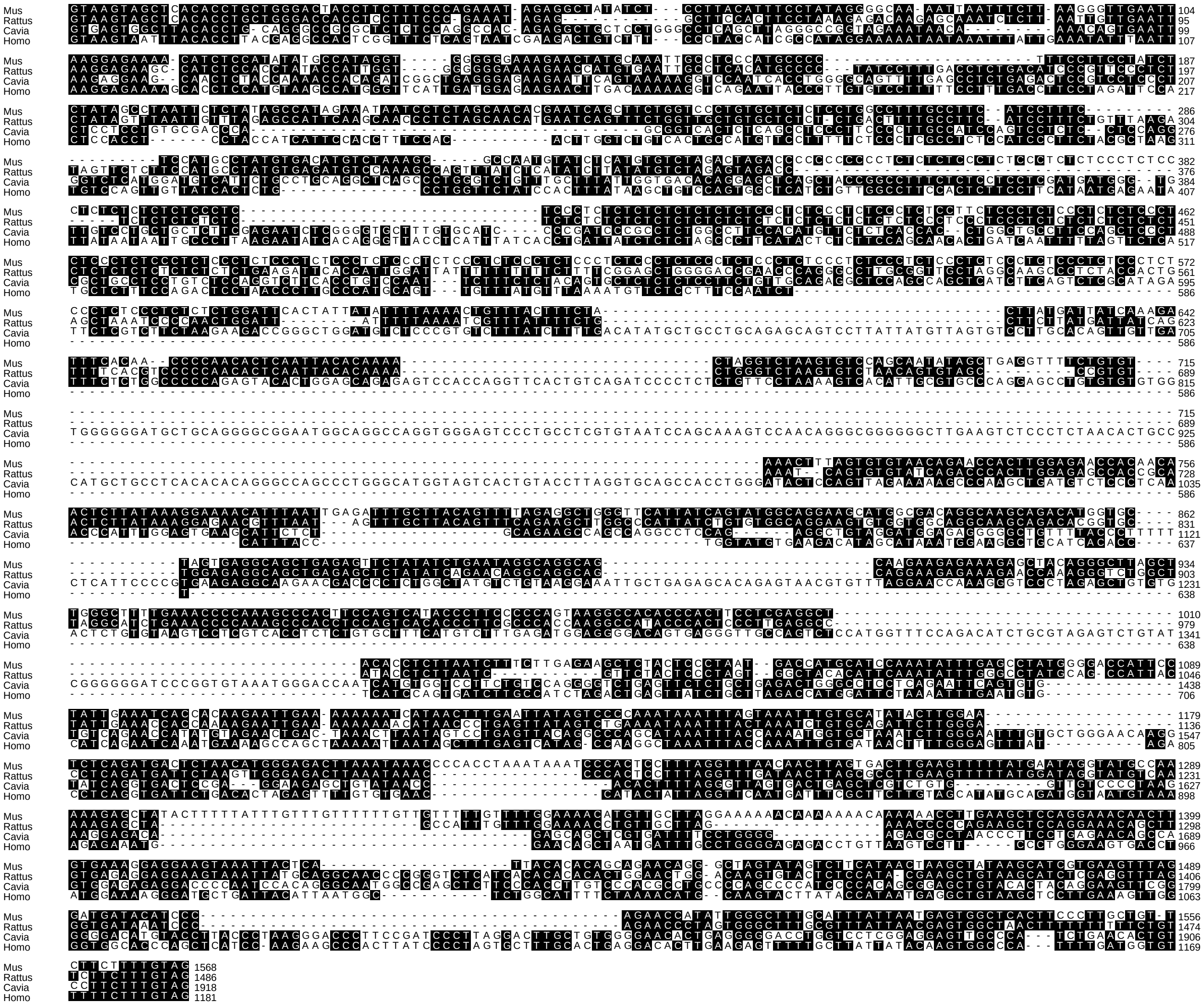

ENSMUSG00000064158 intron 2

Description: Izumo sperm-egg fusion protein 1 Precursor (Izumo1)
Intron number: 2
Mouse chromosome: 7
Upstream exon length: 75
Downstream exon length: 87
Mouse intron length: 766
Intron alignment length: 856
Total murinae branch length: 0.23960
K_score: 0.06154
Scaling factor: 0.5676

ENSMUSG00000064158 exon 2 (ORF 2)

```
Mus     ACGAGAACACACTGGAACAAGCAACCTGGAGTTTTCTGAAGGATCTGAAGCGTATTACAGACAGTGACTTAAAAG 75
Rattus  ATGAGGACACACTGGAACAAGCATCCTGGAGTTTTCTGAAGGATCTGAAACGTATCACGGACAGTGACGTAAAAG 75
Cavia   ATGAGGATACATTGCAACAGGCATCCTGGAGTTTCCTGAAGGATCTGAAACGCCTTACAGAAAGTGATGTAAGAG 75
Homo    ATGAGGCCACACTGCAAAAGGGGTCCTGGAGTTTGCTGAAGGATCTGAAACGCATCACAGACAGTGATGTAAAAG 75
```

ENSMUSG00000064158 exon 3 (ORF 2)

```
Mus     GAGAGCTCTTTATAAAGGAACTATTGTGGATGCTTCGTCATCAAAAGGACATCTTTAACAATCTTGCTAGACAGTTCCAA 80
Rattus  GAGAGCTCTTTGTAAAGGAGCTATTTTGGATGCTTCGTTTGCAAAAGGACATCTTTGCCACCCTTGTTGCACGTTTCCAA 80
Cavia   AGGAGCTCTTCGTGAAGGAGCTGTTTTGGATGCTGCATCTACAGAAAGAGAAGTTTTTCATCCTTGCCAAGCGGTTCCAA 80
Homo    GCGATCTCTTCGTGAAGGAGCTATTTTGGATGTTGCACTTGCAAAAGGAAACCTTTGCCACCTATGTTGCTCGATTCCAA 80

Mus     AAGGAAG 87
Rattus  AAGGAAG 87
Cavia   AAGGAGG 87
Homo    AAAGAGG 87
```

ENSMUSG00000064158_intron_2

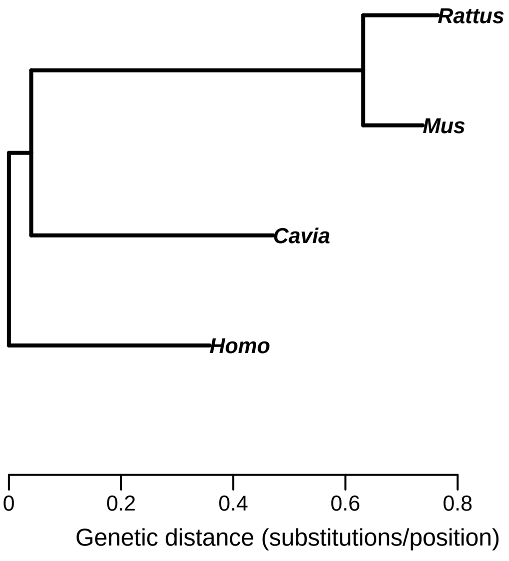

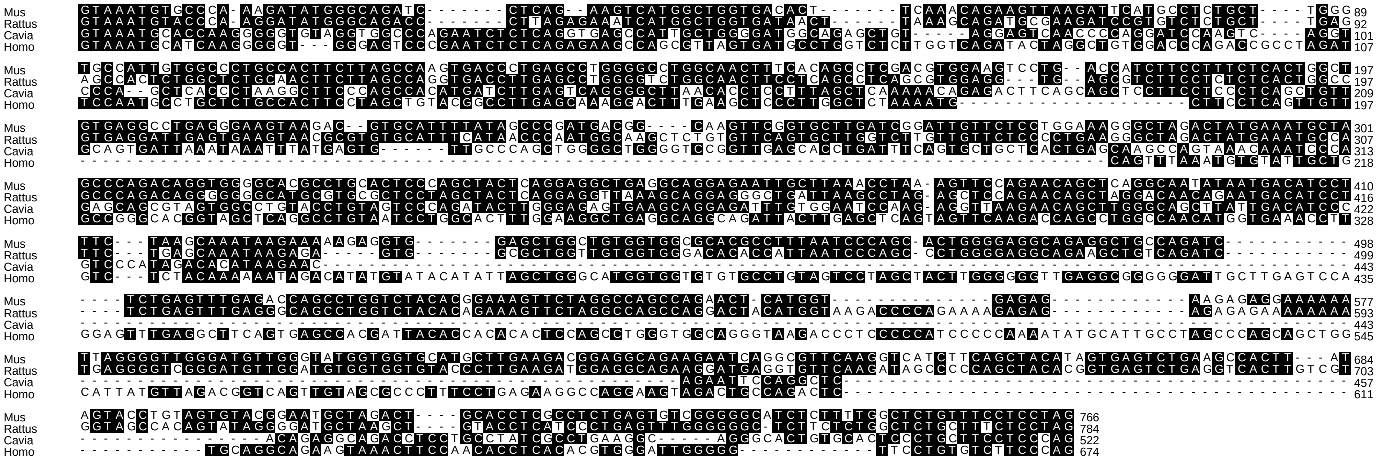
Mus
Rattus
Cavia
Homo

ENSMUSG00000030703 intron 1

Description: Glycerophosphodiester phosphodiesterase domain-containing pr (Gdpd3)
Intron number: 1
Mouse chromosome: 7
Upstream exon length: 139
Downstream exon length: 43
Mouse intron length: 292
Intron alignment length: 411
Total murinae branch length: 0.14735
K_score: 0.08684
Scaling factor: 0.57245

ENSMUSG00000030703 exon 1 (ORF 0)

```
Mus     ATGATCCCTCTCCTGTACTTTGTTCTGCCCACCCTGGGCAGTTACGTCATGCTCTCCATCTTCTTCCTGCGCCGACCTCA 80
Rattus  ATGATCCCCCTCGTGTACTTCGTTCTGCCCACCCTGGGCAGCTATGTAATTCTCTCCATCTTCTTCCTGCGCCGACCTCA 80
Cavia   ATGAGCACCCTGCTCTACTATGCCCTGCCTGCCCTGGGCAGCTATGTCATACTCTCCATCTTCTTCCTGCGCCGGCCTCG 80
Homo    ATGAGCCTTTTGCTGTACTATGCCCTCCCTGCCCTGGGCAGCTATGCCATGCTCTCCATCTTCTTCCTGCGCCGGCCTCA 80

Mus     TCTGCTGCACACGCCGCGGGCTCCCGTCTTCCCTATCCGCCTGGCTGCCCACCGTGGAG 139
Rattus  CCTGCTGCACACACCCTGGGCTCCAGGGTTCTCCATCCGCCTGGCTGCACACCGTGGAG 139
Cavia   CCTGCTGCATACACCTTGGACTCCAGTCTTCCGTCCTCGCCTGGCAGCCCACCGGGGAG 139
Homo    TCTGCTGCACACGCCCAGGGCTCCCACCTTCCGCATCCGCCTGGGGGCCCACCGAGGAG 139
```

ENSMUSG00000030703 exon 2 (ORF 2)

```
Mus     GGTCTGGGGAGCGACTGGAGAACACCATGGAAGCAGTAGAGAA 43
Rattus  GGTCTGGGGAGCGGCTGGAGAACACCATGGAGGCAATAGAGAA 43
Cavia   GGTCTGGAGAGCGACTGGAGAACACCATGGAGGCCATAATGAA 43
Homo    GATCTGGAGAGCTGCTGGAGAACACCATGGAGGCCATGGAGAA 43
```

ENSMUSG00000030703_intron_1

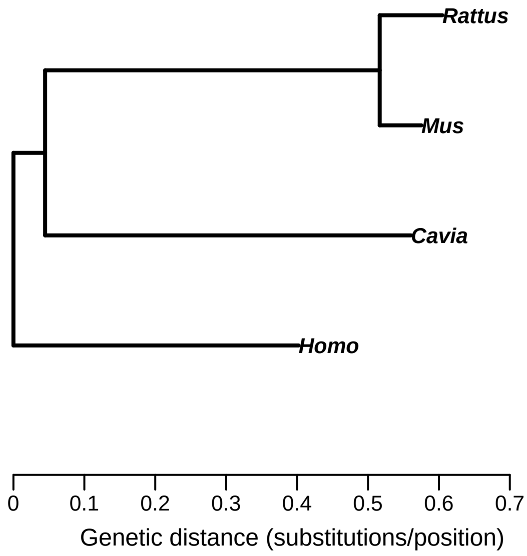

```
Mus     GTGAGCTGAGTCAGGGAGGTGGAA--GGGCTGGAATCCATGTAGGGTGGAGTGAGCCTAACAAGACAGGTGTCAGGGTGG---GCAGCCCA---------TCCTCAAGG- 95
Rattus  GTGAGCTGAATCAGGGATGTGGAA--GGGATGGAATCCATTTAGGATAGAGTGAGCCAAACCAGACAGGTGCCATGGTGGGCAGCAGCACA---------TTGTCAAGG- 98
Cavia   GTAAGCTGGGCAGAGAAGGAAGGGCCAGGGTGGCAGCTGTTAGGAGTGGAGTGAG---------------GTCAACGTGG---GCAGCACTGAGGGGTAGGTCCCAAGGC 92
Homo    GTGAGCCGGGTAGGGGTGA-------------------------------------------GAACAGGTGCCAAAGTGG---ACAGCACAGAGGGGTAGGTCCAAGGG- 63

Mus     TGCAAGGCATGATTGAATGTTAAA--AAGAGCG-AGGAAAGGAGAGTCTTGGGGCCTAGAGGGCATGTGGCAGGGAAGGAGAGGCCAGTTCAACTGATTGGAAGAGAGGA 202
Rattus  TGCAGGGCAGGAGTGAATGTTAAACCAAGAGCG-AGGTAAGGAGAGTCTCGGCAACTGGAGGGCATGTGAC--GGAAGCAGAGGCCAGTTCAGCTGACTGGAAGAGAAGA 205
Cavia   AGAATGCTAGAAGCAAGGCCAGGACCAAGTAGGAGAGCCAGGCAAGCA---------GGGAAGGACTAGGAAGGAAAAGCTGTTCTACCTGAATTACCC---AAAGATGA 190
Homo    GGCATGCTAGAAACGGGGCAGGGACCAAGAGAGAAGGAGAGCGAAGTGGCAGGGCAGGGTGTGGACGAGGAAGAAGATGCTGGCGCAGCCTGGGCTGGCTGGGGAGGTGA 173

Mus     AGCCTAAA-------------------------------------GCAAGTGGGAGACCGT--------------------------------------------CAGGA 231
Rattus  AGCCTAAA-------------------------------------GCAAGCAGATGACCAT--------------------------------------------CAGGA 234
Cavia   GGCCTGGGGCTGGGGATTTAGCTCAGCGGCATAAGCACCTGCCTTGCAAGCAGGCGGTCGTGAGTTCGATCCCCGGTACGGATAAAAGGTTAAAAAGTTAAAAAACAAAA 300
Homo    GGCCAGAG-------------------------------------GGAGGATGGAGCCCA----------------------------------------------GAGA 200

Mus     AGAAAGGTAAGCAGGAAGGTCAAGCTAGGTCCCGGGGATCCA---------CCCAA-----------ACTGCTTTCCACAG 292
Rattus  AGAAAGGTAGGCAGGAAGGTCAAGCCAGGTCCCAGGAACCCA---------CCCAA-----------ACTGCTTTCCACAG 295
Cavia   ACAAAGATGAGGCCTAAGGCCCAGGAAGAGCCAAGAGGGCGGGTAGGTAATAACAGGAGCATGCCCGACTGCCCCACACAG 381
Homo    GGGAATGTGGGTGATGAGGTTGGGCCAGGCCCCTAGACCCCA------AGCCCCAGAGGCCCACTCCAGTGCCCCCCACAG 275
```

ENSMUSG00000025733 intron 5

Description: Mitochondrial Rho GTPase 2 (Rhot2)
Intron number: 5
Mouse chromosome: 17
Upstream exon length: 54
Downstream exon length: 53
Mouse intron length: 688
Intron alignment length: 966
Total murinae branch length: 0.27959
K_score: 0.08942
Scaling factor: 0.57345

ENSMUSG00000025733 exon 5 (ORF 0)

```
Mus     GCAAATGTAGTGTGTGTGGTGTACGATGTGTCTGAGGAGACCACCATTGAGAAG 54
Rattus  GCAAACGTGGTGTGTGTGGTGTACGATGTGTCTGAGGAAGCCACCATTGAGAAG 54
Cavia   GCAAATGTGGTGTGTGTGGTATATGATGTGTCTGAAGAGGCCACCATTGAGAAG 54
Homo    GCAAACGTGGTGTGTGTGGTGTATGACGTCTCTGAGGAGGCCACCATTGAGAAG 54
```

ENSMUSG00000025733 exon 6 (ORF 0)

```
Mus     ATCCGAACCAAGTGGATCCCGCTTGTGAATGGCAGGACGGCGACAGGGCCCAG 53
Rattus  ATCCGAACCAAGTGGATTCCCCTCGTGAATGGCAGGACGGCAACAGGGCCCAG 53
Cavia   ATCCGAACCAAGTGGATCCCACTGGTGAATGGCAGAACTGGAAGTGGGCCCAG 53
Homo    ATTCGAACTAAGTGGATCCCACTGGTGAATGGGGGGACCACGCAGGGGCCCAG 53
```

ENSMUSG00000025733_intron_5

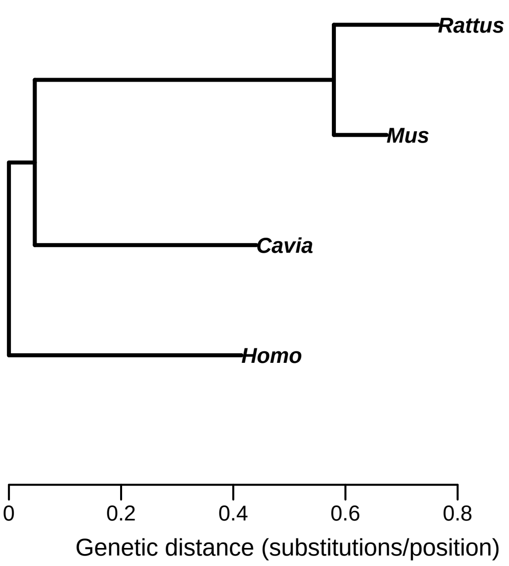

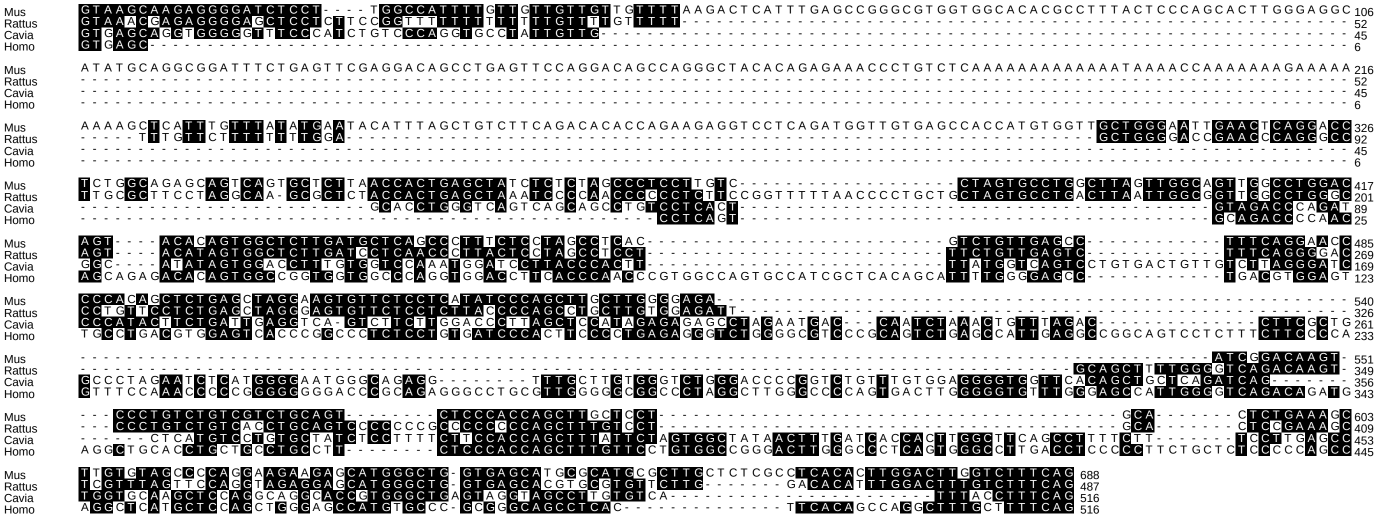
Mus
Rattus
Cavia
Homo

ENSMUSG00000008140 intron 1

Description: UPF0510 protein C19orf63 homolog Precursor (2310044H10Rik)
Intron number: 1
Mouse chromosome: 7
Upstream exon length: 114
Downstream exon length: 73
Mouse intron length: 1375
Intron alignment length: 1729
Total murinae branch length: 0.18755
K_score: 0.07851
Scaling factor: 0.58215

ENSMUSG00000008140 exon 1 (ORF 0)

```
Mus     ATGGTGGCCGCGGGTGCCGGGGTGACCCGGCTGCTAGTGCTCTTGCTGATGGTAGCCGCGGCTCCTAGCAGAGCCCGAGG 80
Rattus  ATGGTGGCCGCGGGTGCCGGCGTGACCCAGCTGCTAGTGCTCTTGTTGATGGTAGCCGCGGTTCCTAGCAGAGCCCGGGG 80
Cavia   ATGGCGGCGGCCGGCGCGGGGGCACCCCGGTTGCTCCTCCTTTTACTGATAGTGGCAGCAGCGCCCAGCCGGGCCAGGGG 80
Homo    ATGGCGGCAGCCAGCGCTGGGGCAACCCGGCTGCTCCTGCTCTTGCTGATGGCGGTAGCAGCGCCCAGTCGAGCCCGGGG 80

Mus     CAGCGGCTGCCGGGTCGGGGCCTCCGCGCGTGGG 114
Rattus  CAGCGGCTGCCGGGTCGGAGCCGCCGCGCGCGGG 114
Cavia   CAGCAGCTGCCGGGCTGGAGCCGCTACGCGGGGG 114
Homo    CAGCGGCTGCCGGGCCGGGACTGGTGCGCGAGGG 114
```

ENSMUSG00000008140 exon 2 (ORF 0)

```
Mus     ACCGGGGCCGATGGCCGTGAAGCTGAGGGCTGTGGCACCGTGGCTTTGCTGCTGGAGCATTCATTTGAGCTCG 73
Rattus  GTGGGAGCTGATGGCCATGAAGCTGAGGGCTGTGGCACTGTGGCATTGCTGCTGGAACATTCATTTGAGATTG 73
Cavia   GTTGGGGCCGAAGGCCGTGAGGGCGAGAGCTGCGGTACGGTGGGGCTGCTGCTGGAGCACTCCTTTGAGATCG 73
Homo    GCTGGGGCGGAAGGTCGAGAGGGCGAGGCCTGTGGCACGGTGGGGCTGCTGCTGGAGCACTCATTTGAGATCG 73
```

ENSMUSG00000008140_intron_1

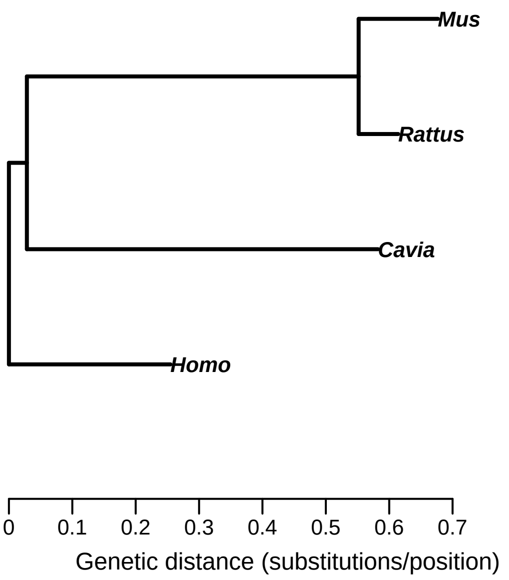

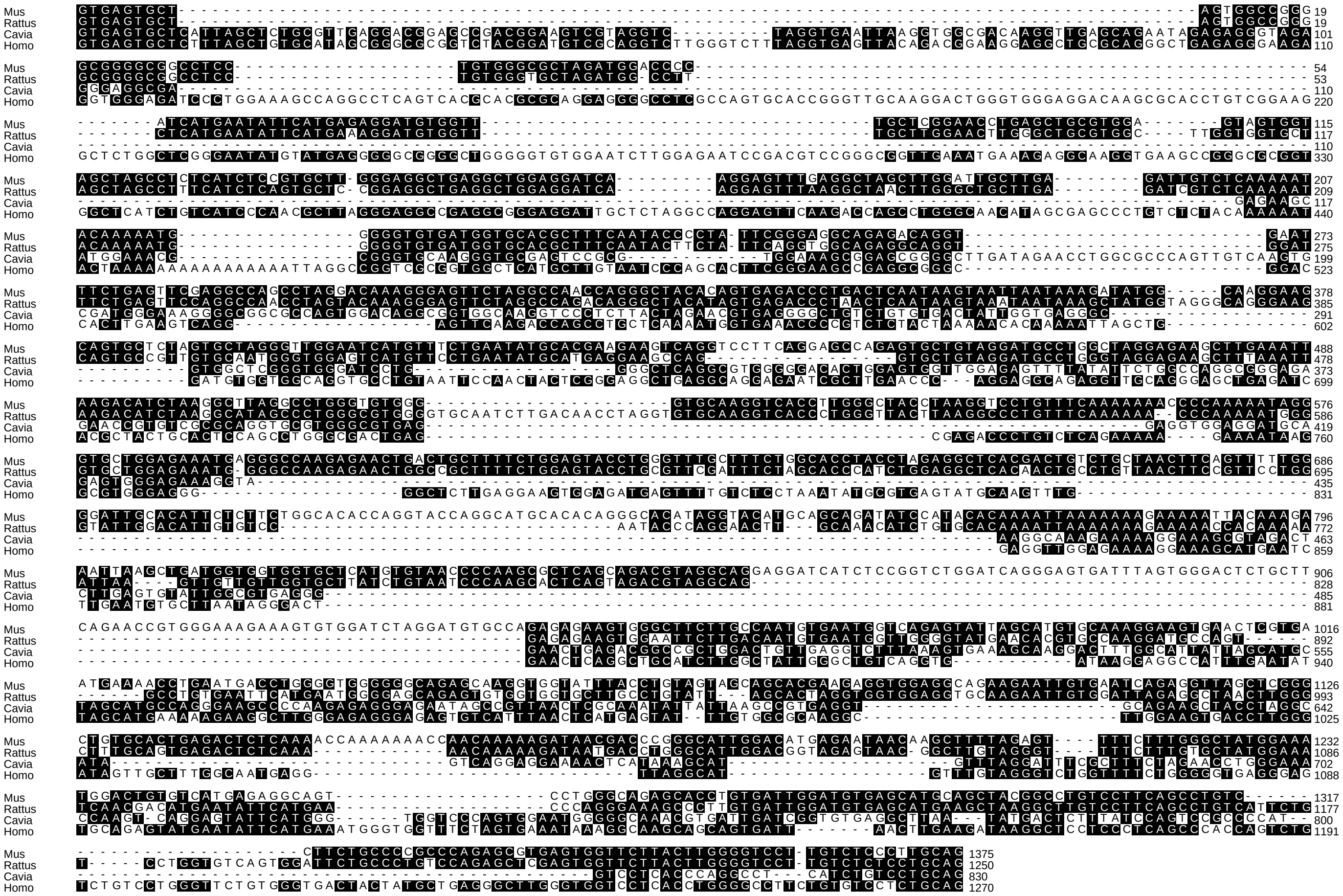

ENSMUSG00000026281 intron 1

Description: Thymidylate kinase (Dtymk)
Intron number: 1
Mouse chromosome: 1
Upstream exon length: 130
Downstream exon length: 109
Mouse intron length: 787
Intron alignment length: 1273
Total murinae branch length: 0.17899
K_score: 0.08402
Scaling factor: 0.58328

ENSMUSG00000026281 exon 1 (ORF 0)

```
Mus     ATGGCGTCGCGTCGGGGAGCGCTCATCGTGCTGGAGGGTGTGGACCGTGCTGGCAAGACCACGCAGGGCCTCAAGCTGGT 80
Rattus  ATGGCGTCGCGTCGCGGGGCGCTCATCGTGCTGGAGGGTGTGGACCGTGCTGGCAAGACCACGCAGTGCCTCAGGCTGGT 80
Cavia   ATGGCGGGCAGGCGGGGGGCTCTCATCGTGCTGGAGGGTGTGGACCGCGCGGGCAAGAGTACGCAGAGTCGCAAGTTGGT 80
Homo    ATGGCGGCCCGGCGCGGGGCTCTCATAGTGCTGGAGGGCGTGGACCGCGCCGGGAAGAGCACGCAGAGCCGCAAGCTGGT 80

Mus     GACCGCGCTGTGCGCCTCGGGCCACAGAGCGGAGCTGCTGCGTTTCCCCG 130
Rattus  GACCGCGCTGTGCGCCTCGGGCCACAGAGCGGAGCTGCTGCGTTTCCCTG 130
Cavia   GTCTGCTCTGTGCGCCGCCGGCCACCGCGCCGAACTTCTGCGCTTTCCTG 130
Homo    GGAAGCGCTGTGCGCCGCGGGCCACCGCGCCGAACTGCTCCGGTTCCCGG 130
```

ENSMUSG00000026281 exon 2 (ORF 2)

```
Mus     AAAGATCAACGGAAATCGGCAAGCTTCTGAATTCCTACTTGGAAAAGAAAACGGAACTAGAGGATCACTCCGTGCACCTG 80
Rattus  AAAGATCAACAGAAATCGGCAAGCTTCTGAGTTCCTACTTAGAAAAGAAAACCGAGCTCGAGGATCATTCCGTGCACCTG 80
Cavia   ATCGATCAACGGAAATTGGCAAACTTCTTAGTTCTTACTTGGAAAAGAAAAGTGAACTTGAGGATCACTCAGTGCACCTA 80
Homo    AAAGATCAACTGAAATCGGCAAACTTCTGAGTTCCTACTTGCAAAAGAAAAGTGACGTGGAGGATCACTCGGTGCACCTG 80

Mus     CTCTTCTCTGCAAACCGCTGGGAACAAGT 109
Rattus  CTCTTCTCTGCCAACCGCTGGGAACAAGT 109
Cavia   CTTTTTTCTGCCAACCGCTGGGAACAAGT 109
Homo    CTTTTTTCTGCAAATCGCTGGGAACAAGT 109
```

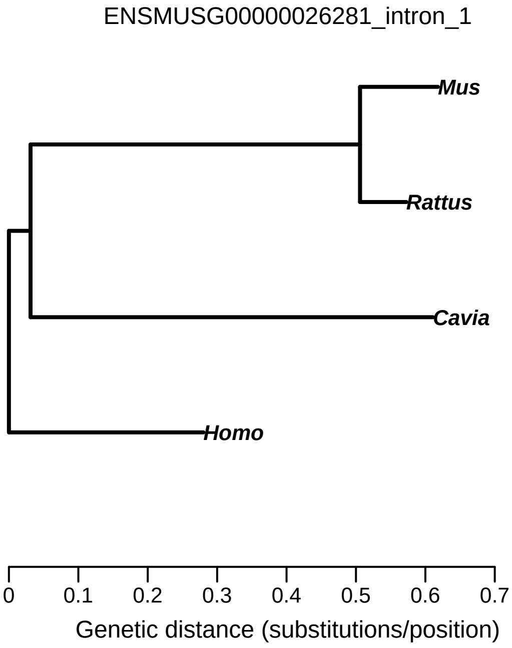

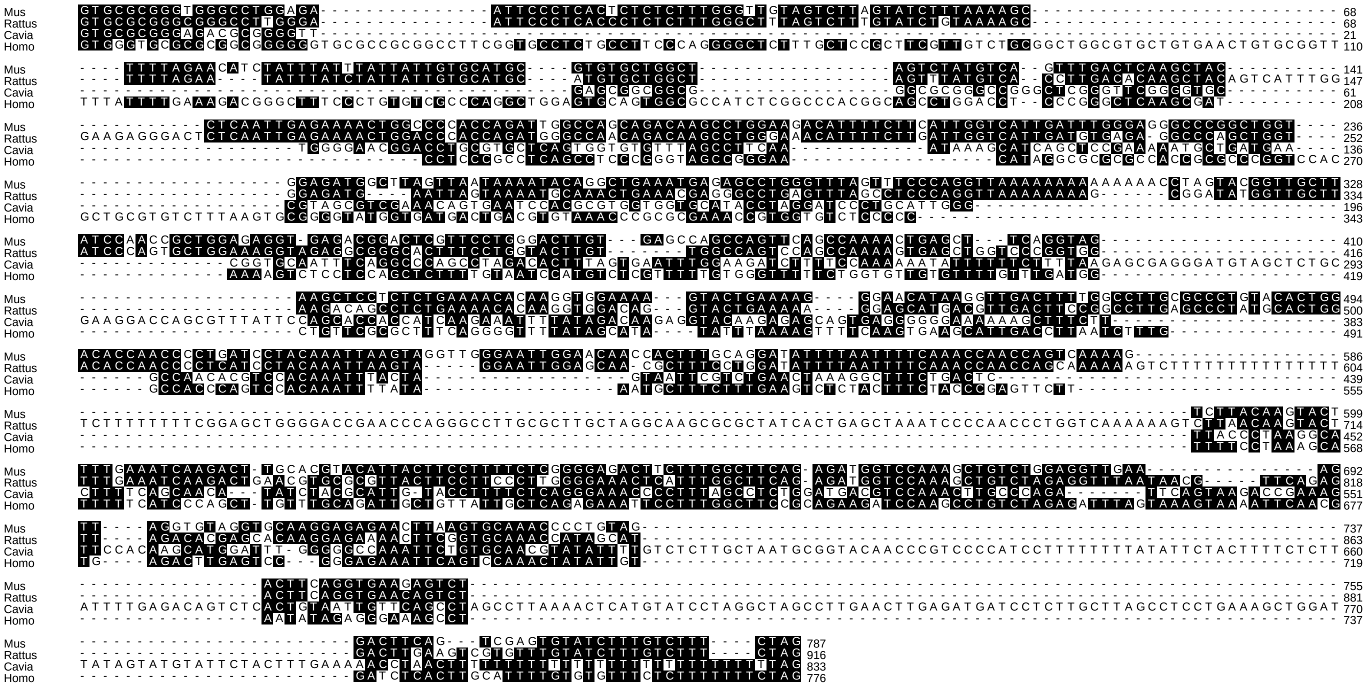

ENSMUSG00000027217 intron 3

Description: Tetraspanin-18 (Tspan18)
Intron number: 3
Mouse chromosome: 2
Upstream exon length: 75
Downstream exon length: 99
Mouse intron length: 895
Intron alignment length: 1264
Total murinae branch length: 0.16449
K_score: 0.06838
Scaling factor: 0.58936

ENSMUSG00000027217 exon 3 (ORF 0)

```
Mus     TTCTTCCTGTTTATCCTGATCATCTTCCTGGTAGAGCTCTCAGCAGCCATCCTGGCCTTCATCTTCAGAGAACAC 75
Rattus  TTTTTCCTGTTTATCCTGATCATCTTCCTGGTAGAGCTCTCAGCAGCCATCCTGGCCTTCATCTTCCGGGAACAC 75
Cavia   TTTTTCCTGTTCATCTTGGTCATCTTCCTGGCGGAGCTCTCAGCAGCCATCCTGGCCTTCATCTTCAGGGAAAAC 75
Homo    TTCTTCCTGTTCATCCTGATCATCTTCCTGGCAGAGCTCTCAGCAGCCATCCTGGCCTTCATCTTCAGGGAAAAT 75
```

ENSMUSG00000027217 exon 4 (ORF 0)

```
Mus     CTCACCCGAGAGTTCTTCACCAAGGAGCTTACCAAGCACTACCAAGGCGATAACGATACGGATGTTTTCTCTGCCACCTG 80
Rattus  CTCACCCGAGAGTTCTTCACCAAGGAGCTTACCAAGCACTACCAAGGCGATAATGACACGGACGTTTTCTCTGCCACCTG 80
Cavia   CTCACCCGTGAGTTCTTCACCAAGGAGCTCACCAAGCACTACCAGGGCAGTAACGAGACAGATGTCTTCTCCGCCACCTG 80
Homo    CTCACCCGAGAATTCTTCACCAAGGAGCTCACCAAGCACTACCAGGGCAATAACGACACAGACGTCTTCTCTGCCACCTG 80

Mus     GAATTCAGTCATGATCACA 99
Rattus  GAATTCCGTCATGATCACA 99
Cavia   GAACTCTGTCATGATCACA 99
Homo    GAACTCGGTCATGATCACA 99
```

ENSMUSG00000027217_intron_3

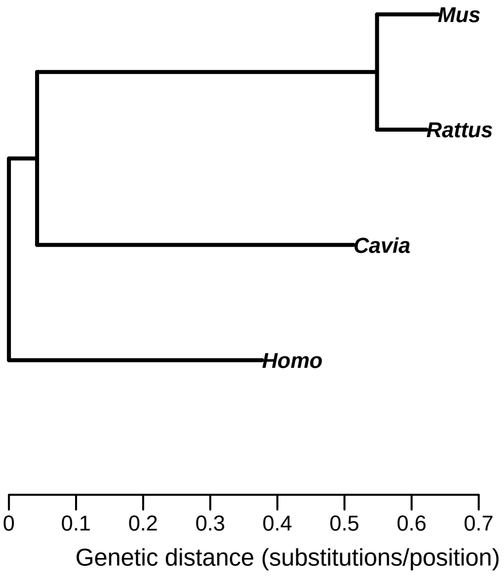

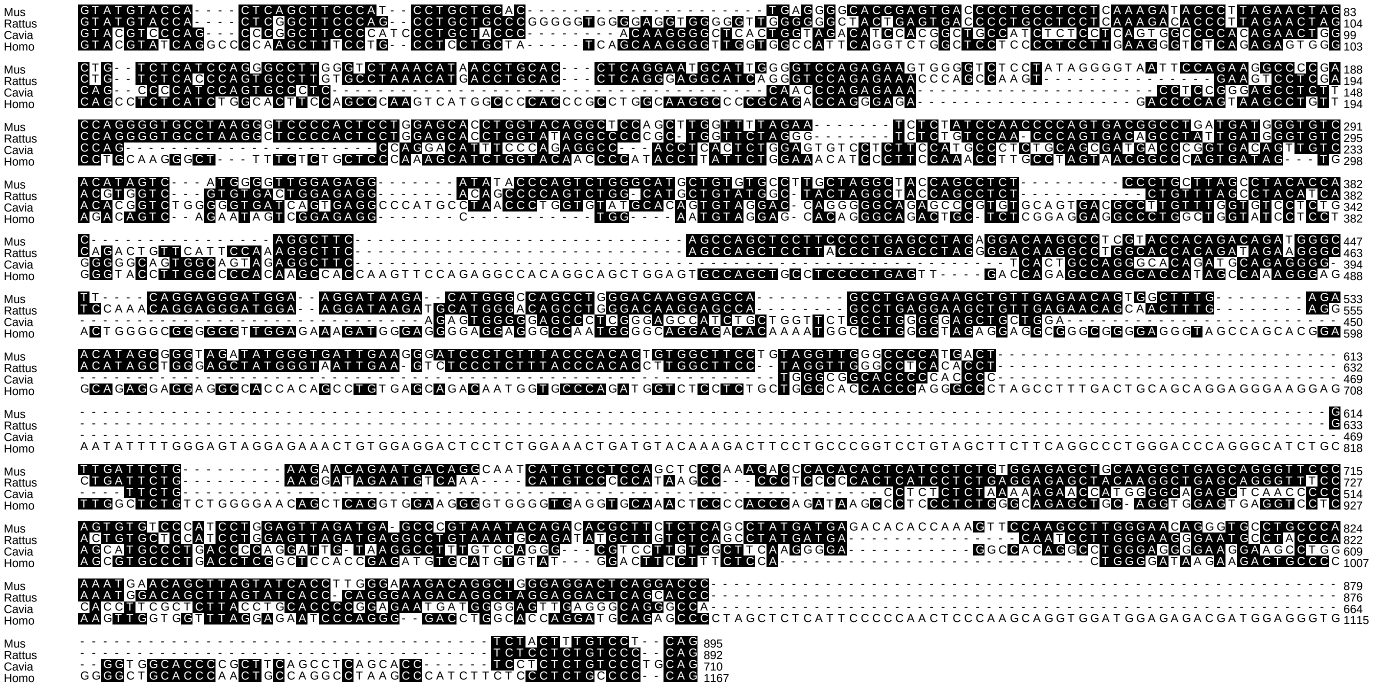

ENSMUSG00000022598 intron 2

Description: Prostate stem cell antigen Precursor (Psca)
Intron number: 2
Mouse chromosome: 15
Upstream exon length: 108
Downstream exon length: 212
Mouse intron length: 257
Intron alignment length: 506
Total murinae branch length: 0.24668
K_score: 0.04827
Scaling factor: 0.58956

ENSMUSG00000022598 exon 2 (ORF 2)

```
Mus     ---GTGCTGCTCTGCAGTGCTATTCATGCACAGCACAGATGAACAACAGAGACTGTCTGAATGTACAGAACTGCAGCCTG 77
Rattus  ---GTGCCGCTCTGCAGTGCTATTCATGCACCGCACAGGTGAGCAACAGAGATTGTCTGAATGTACAGAACTGCACCCTG 77
Cavia   CAGGTGCTGCTCTGCAGTGCTACTCCTGCACTGCCCAGGTGAACAACCGCGACTGCCAGAAGGTACAGAACTGCAGCCAC 80
Homo    ---GCACTGCCCTGCTGTGCTACTCCTGCAAAGCCCAGGTGAGCAACGAGGACTGCCTGCAGGTGGAGAACTGCACCCAG 77

Mus     GACCAGCACAGTTGCTTTACATCGCGCATCC 108
Rattus  GACCAGAACAGCTGTTTTACATCTCGTGTCC 108
Cavia   ACGGACACCTACTGCTGGACTTCACGTGTCG 111
Homo    CTGGGGGAGCAGTGCTGGACCGCGCGCATCC 108
```

ENSMUSG00000022598 exon 3 (ORF 2)

```
Mus     GGGCCATTGGACTCGTGACAGTTATCAGTAAGGGCTGCAGCTCACAGTGTGAGGATGACTCGGAGAACTACTATTTGGGC 80
Rattus  GGACCATTGGACTCCTGACATTTATCAGTAAGGGCTGCAGCTCAAAGTGTGAGGATGACACGGAGAACTACTATGTGGGC 80
Cavia   GTGCCATCGGGGTCCTGGAGTTCATCAGCAAGGGTTGCACCTCTGATTGTGTGGATGACTCAGACAACTACTACTTTGGC 80
Homo    GCGCAGTTGGCCTCCTGACCGTCATCAGCAAAGGCTGCAGCTTGAACTGCGTGGATGACTCACAGGACTACTACGTGGGC 80

Mus     AAGAAGAACATCACGTGCTGCTACTCTGACCTGTGCAATGTCAACGGGGCCCACACCCTGAAGCCA---CCCACCACCCT 157
Rattus  AAGAAGAATATCACATGCTGCTCCTCTGACCTGTGCAATGTCAACGGGGCCCACACCCTGAAGCCA---CCCACCACCCT 157
Cavia   AAGAAAAACATCACCTGCTGCTCTACTGACCTGTGCAATGCCAGCAGAGCCTACGCCCTGAAGCCA---ACCACCACTCT 157
Homo    AAGAAGAACATCACGTGCTGTGACACCGACTTGTGCAACGCCAGCGGGGCCCATGCCCTGCAGCCGGCTGCTGCCATCCT 160
```

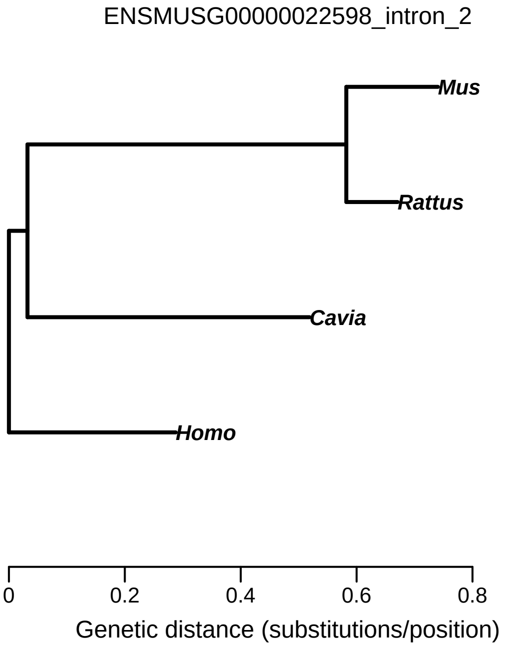

```
Mus     GTGAGTG-------------------------------------------------------------------------------------------------------   7
Rattus  GTGAGTG-------------------------------------------------------------------------------------------------------   7
Cavia   GTGAGTG---------------------------------------------------------------------TCAACCTGCTGTGTTCCTTC--------------  27
Homo    GTGAGTGGGGGGACGACAGCCGCCAGGCCTAGGTCTCTGCCACTGAACTATTAATCTTTCTGGCCATCTGTCCGCATCTGTGTGCTGTTTTCCTTCCACCTGTCCCCGAC 110

Mus     -------------------------------------------------------------------------------------CTCTGCCGCCCCCCACCCCCAGTGC  32
Rattus  -----------------------------------------------------------------------------------------------CCCCAAC--------  14
Cavia   ---TTCTCCACTGGCAGCTGCTGTAGAACATGGGCCTTGTGTCTGTTATT------------------------------------TTTCTCTCTGCCCATCTTCTCCCT  98
Homo    CCGTCCCGCACCTGCACCCCC-----AACAATCACCCAGCATCTGTCCCTCCAGCCATCCTCCTCCATCTGCCACTCCTCCACTCATCTGTCCCTCCCCATCCTCCATCT 215

Mus     TCTGCTGC------------------CCCATCCCCGGTCCTCACTGCCTGCTGC---------------CCCACCGGGCGGCCTCCCTCCCCACTCCAGCCAGACAT--- 106
Rattus  --------------------------CCCCACCCCTGTCCCCACTGCCTGCTGC---------------CCTGCCATGCAGCCTCCCTCCCCACTGCAGCTAGACAG---  80
Cavia   TACCCATCTATCCCCACATGCCCGCTCACCTTCCTGCCCACCTCTCCATGCTGCACTCAGATTGTTTCGCTCCA-----GGCCCCATTTCCTTAGGCACTTGGGTGTGGG 203
Homo    TCCACTCCTCCACCCATCTGTCCCTCCCCATCCCTGAGCTCACTTACTCACT-CACCCCATTTCTGACGCTCAGCGGGTGGTCCATCTGCCTCGGACATCTGGATAGGGC 324

Mus     ------------------CAAAGCTTTGCTCACCCATTCACCAACCATTTCTGGTCTTTGTGGAGTGGGTCCAGAAG-TTTAGCATGTTCCGGAAGGGGC------CCTG 191
Rattus  ------------------CGAAGAAACTGTCATTCACCAA------ATTTCTGGTCTTTGTGGGGTGGGTCTAGAGATTTTATCATGCTCAGGAATGGGA------CCTG 160
Cavia   CTGGACCAGACTCCTGGTTTTGGGCAGCTTTTC-------------TTTCATGGCCAG---GGGAGGGGACTTGAGCTCCTATTTAGAGGAGGCATGGGGTGGGCAGCTG 297
Homo    TGAGACCAG------GGCCGAGACCAGGCCCTCGCACTGCTTGCAATCCTGAGGCCAGCCCAGGGGGACTCTAGAGCATTAGGCAGGGTGGGACAGGAGGAGG---CCTG 425

Mus     AGCCAGGGGCAGAGAGGTGGCTATCAGGACCTGGCTTTAATCCCAAGTGTCACTCTCTCTTCCCAG 257
Rattus  AGGCAGGGGCAGAGAGGTGAATACCAAGACTTGACTTTAATCCCAAGCATCACTCTGTCTTCCCAG 226
Cavia   GGGCAGGCCTGGACAAGTAAGTTCCAGGACCTGTCCTCAGCCAGTGGTCACCAC---GCCCTCCAG 360
Homo    GGGCAGGTC-AGGCAGGTGAGCACACAGGGCAG-CCCCATCCCCGGATCCCGCT---GCTCCCCAG 486
```

ENSMUSG00000022469 intron 16

Description: Rap guanine nucleotide exchange factor 3 (Rapgef3)
Intron number: 16
Mouse chromosome: 15
Upstream exon length: 82
Downstream exon length: 147
Mouse intron length: 314
Intron alignment length: 361
Total murinae branch length: 0.24713
K_score: 0.03549
Scaling factor: 0.60024

ENSMUSG00000022469 exon 16 (ORF 0)

```
Mus     GCCCGGAATGCACCTGTTTGGCTCCCTAACCAGGAGGAACCCCTCCCAAGCAGCGCGGGTGCCATCCGAGTTGGGGACAA 80
Rattus  GCCCGGAATGCACCTGTTTGGTTTCCTAACCATGAGGAACCCCTCCCAAGCAGCGCTGGGGCCATCCGAGTCGGGGACAA 80
Cavia   GCCCGGAACATACCTGTTTGGCTCCCCAGCCAGGATGAGCCTCTCCCAAGCAGCAACTGTGCCATCCGAGTTGGGGACAA 80
Homo    GCCCGGAACTTGCCTGTTTGGCTCCCCAACCAGGACGAGCCCCTTCCTGGCAGCAGCTGTGCCATCCAAGTTGGGGATAA 80

Mus     AG 82
Rattus  AG 82
Cavia   AG 82
Homo    AG 82
```

ENSMUSG00000022469 exon 17 (ORF 2)

```
Mus     TCCCCTACGACATCTGCAGACCTGACCACTCAGTGCTGACCCTGCACCTGCCGGTGACGGCCTCGGTGAGAGAAGTGATG 80
Rattus  TCCCCTATGATATCTGCAGACCCGACCACTCGGTGCTGACCCTGCACCTGCCGGTGACAGCCTCCGTGAGGGAAGTGATG 80
Cavia   TGCCCTATGACATCTGCCGGCCCGACCACTCAGTGCTGACCCTGCAGCTGCCTGTGACAGCCTCGGTGAGAGAAGTAATG 80
Homo    TCCCCTATGACATCTGCCGGCCAGACCACTCAGTGTTGACCCTGCAGCTGCCTGTGACAGCCTCCGTGAGAGAGGTGATG 80

Mus     GCAGCTTTGGCCCATGAGGACCACTGGACCAAGGGACAGGTGTTGGTGAAGGTCAATTCTGCCGGTG 147
Rattus  GCAGCTTTGGCCCATGAGGACCACTGGACCAAGGGGCAGGTGCTGGTAAAGGTCAATTCTGCCGGTG 147
Cavia   GCAGCACTGGCCCAGGAAGATGGCTGGACCAAAGGGCAGGTGCTGGTGAAAGTCAACTCTGCAGGCG 147
Homo    GCAGCGTTGGCCCAGGAGGATGGCTGGACCAAGGGGCAGGTGCTGGTGAAGGTCAATTCTGCAGGTG 147
```

ENSMUSG00000022469_intron_16

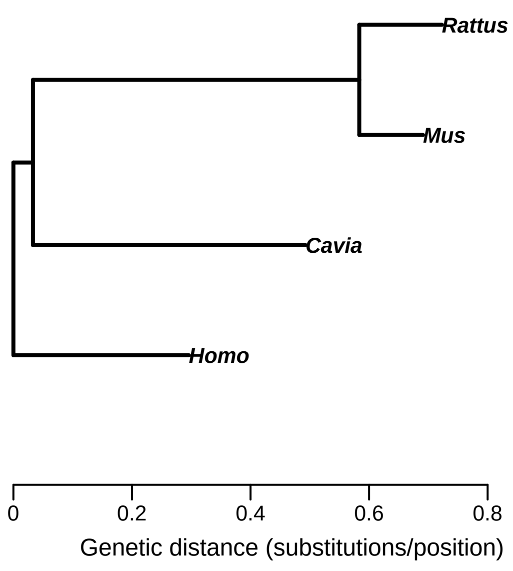

```
Mus     GTGGGTGGGGTAACTGAGCAGGTCAGGCA-------------------TGCTGTCTCTGTCCCTCTTGCCTGG---CTGCTGTTGTTGT----------------GGGCT 72
Rattus  GTGGGTGGGATAAATAAGCAAGCTGGGCGTAGACTG---TGTCAGCATTGCCATCTCTGTCTCCCCTGCCTGG---CTGGTGTCGCTCT----------------GGGCT 88
Cavia   GTAAGTGGTCCCAGCCAGAAGGACAAACAGAGCCTGGCTTCTGCACTCAGCTGTTTCTGCCCCTCTTGTGAAGAGTCAGCAGATGTCCTCTGCTGGGGATACCTGGGTCT 110
Homo    GTTGGTGGTCTGTGCC----GGCTGGGCACAGCCCGACATCTGCACCCTGCTGTCTCTGCCTCTCTTGTCCTG---CTGTCGTCACTCTTGACCTCGGTGTGCCTGGGCC 103

Mus     AGGGGAAGACTTGGCCCTCCTCAGGGTCTTTACTGCAG-CTGCCAAGCAGGTTTTCAGCCACCCGTGCTGCTTCCTCAGGTCCCGGACTCCCCACTGGGCCTTGATATCA 181
Rattus  AGGGGAGGACATG----TCCCCACAGCCTTTACTGTAG-CTGCCAAGCGGG---TCAGCCACCTGTGCTCCTTCCCTA------GGACTCCCCACTGGGCTTTGGCACCA 184
Cavia   GGGGCAGCACTCTGTGCTCTCAGAGGTCTCTGTTCCAGGATGCTAGGCAG----TTGGCCTCCTGTGCCCCTGTCCTG-GCTCAGGGTTCCTCTGGCCACCTGGCTCTGC 215
Homo    AAGGTGGCACGCTGTACTCCTCAGGGTCTGTGTTCCAGGCTGCTGGCTAGA---TCTGCCTCCTACCTGCCTCTTTTTTGCTCAGGGCCTCCCCACCTGC---------- 200

Mus     ACCCACTTTGTTGTGCCTTGTAT--CCTGTTTCTGGCCTATGGGAGTCTTCTTG----CTGCTGAGCTAGGGCCCTGCTCCTGGTGGGGAGGGGAGACCAGGCCTCTGCT 285
Rattus  ACCTACTTTGCTTCTCCTTGTATCCCCCTTTCCTGCCCTATGGTTGTCCTTTGG--------------------CTGCTCCTCCTAGGGAGGGGAGACCAGGCACCTGCT 274
Cavia   GCAGCCTGCAGGTTTAACCTTTGCGCCTGTTCCCTGTTCCTGGGGGACCCTTGC----TTTGTGTAGTTGGTACCCTCCCTCCCTGAGCTGGGGAGACCAGGCAGCTCCT 321
Homo    ----------------------------AGCTCAGCCTCTTGGGGATCCCTCCCACCATTACTGCAATAGCCGCCCTCTCCCTCCTGGG-GTGGAGATCAGGCAGCCCTT 281

Mus     TC--CAGCTCATCTCCACCCCTCCTCTCCAG 314
Rattus  TC--CAGCTCATCCCCTCC--TCCTCTCCAG 301
Cavia   CCGACCACTCAATCCCGTC-CTCCTCCCCAG 351
Homo    CCCACAGCTCAGTCTGGTC--TCCTCTTCAG 310
```

ENSMUSG00000027332 intron 8

Description: Isovaleryl-CoA dehydrogenase, mitochondrial Precursor (Ivd)
Intron number: 8
Mouse chromosome: 2
Upstream exon length: 94
Downstream exon length: 82
Mouse intron length: 478
Intron alignment length: 818
Total murinae branch length: 0.15541
K_score: 0.07661
Scaling factor: 0.6038

ENSMUSG00000027332 exon 8 (ORF 2)

Mus	CCGCTAACGTCCTGAGCCAGGAGAGTAAGGGGGTCTACGTATTGATGAGCGGGCTGGACCTAGAGCGCCTGGTGCTAGCA	80
Rattus	CTGCTAACATCCTGAGCCAAGAGAGTAAGGGTGTCTACGTACTGATGAGCGGGCTGGACCTAGAACGCCTGGTGCTGGCA	80
Cavia	CTGCCAACACCCTGGGCCAGGAGAGCAAGGGCGTCTATGTGCTGATGAGTGGGCTGGACCTGGAACGCCTGGTGCTGGCA	80
Homo	CTGCCAACATCCTGGGCCATGAGAATAAGGGTGTCTACGTGCTGATGAGTGGGCTGGACCTGGAGCGGCTGGTGCTGGCC	80

Mus	GGTGGGCCCCTTGG	94
Rattus	GGTGGACCCCTTGG	94
Cavia	GGTGGGCCCCTTGG	94
Homo	GGGGGGCCTCTTGG	94

ENSMUSG00000027332 exon 9 (ORF 1)

Mus	GATCATGCAGGCTGTCCTGGACCACACCATTCCCTACTTGCATGTGAGGGAGGCCTTTGGCCAGAAGATCGGCCAATTCC	80
Rattus	GATCATGCAGGCTGTCCTGGACCACACCATTCCCTATTTGCATGTGAGGGAAGCCTTTGGCCAGAAGATCGGCCAATTCC	80
Cavia	GTTGATGCAAGCCGTCCTTGATCACACCATTCCTTACCTGCACATGAGGGAAGCCTTTGGCCAGAAGATCGGGCACTTTC	80
Homo	GCTCATGCAAGCGGTCCTGGACCACACCATTCCCTACCTGCACGTGAGGGAAGCCTTTGGCCAGAAGATCGGCCACTTCC	80

Mus	AG	82
Rattus	AG	82
Cavia	AG	82
Homo	AG	82

ENSMUSG00000027332_intron_8

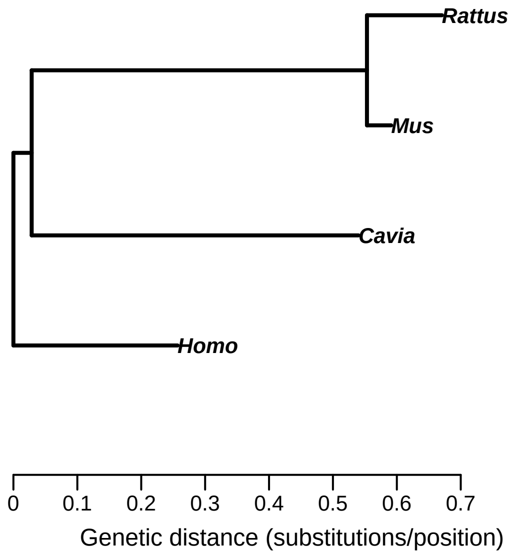

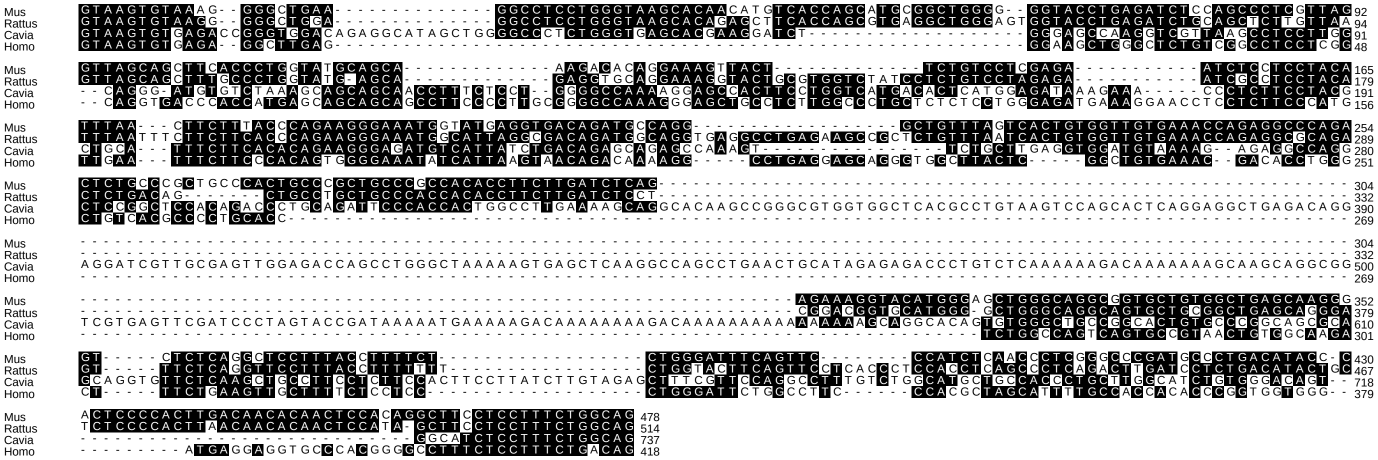

Mus
Rattus
Cavia
Homo
92
94
91
48
165
179
191
156
254
289
280
251
304
332
390
269
304
332
500
269
352
379
610
301
430
467
718
379
478
514
737
418

ENSMUSG00000041945 intron 2

Description: Major facilitator superfamily domain-containing protein 9 (Mfsd9)
Intron number: 2
Mouse chromosome: 1
Upstream exon length: 88
Downstream exon length: 44
Mouse intron length: 1319
Intron alignment length: 1526
Total murinae branch length: 0.26634
K_score: 0.03286
Scaling factor: 0.60485

ENSMUSG00000041945 exon 2 (ORF 0)

```
Mus     GACCTGTTTGGTGTCAGCATGGTGGTCCCATTGCTGAATCTTCATGTCAGATCCCTCGGAGCAAGTCCCGTAGTGGCTGG 80
Rattus  GATCTGTTTGGTGTCAGCATGACTGTCCCATTGTTGAATCTTCATGTCAGATCCCTTGGAGTAAGTCCCGTCGTTGCTGG 80
Cavia   GATTTGCTTGGGGTCAGCATGGTCATCCCGTTGTTGAGCCTTCACGCCAAGTCACTTGGAACAAGTCCTACTGTGGCTGG 80
Homo    GATTTGTTTGGTGTCAGCATGGTTGTGCCTTTATTGAGCCTTCATGTCAAGTCCCTTGGAGCAAGTCCAACAGTTGCTGG 80

Mus     AATAGTAG 88
Rattus  AATAGTAG 88
Cavia   CATCATCG 88
Homo    AATAGTAG 88
```

ENSMUSG00000041945 exon 3 (ORF 2)

```
Mus     GCTCCTCATACGGCGTTTTGCAACTCTTCTCCAGCACCTTTGTG 44
Rattus  GCTCCTCCTATGGCATTTTGCAACTCTTCTCCAGCACCTTTGTG 44
Cavia   GTTCCTCCTACGGAATTTTGCAGCTCTTCTCCAGCACGCTGGTG 44
Homo    GCTCCTCCTATGGCATTTTGCAACTCTTTTCTAGCACATTGGTG 44
```

ENSMUSG00000041945_intron_2

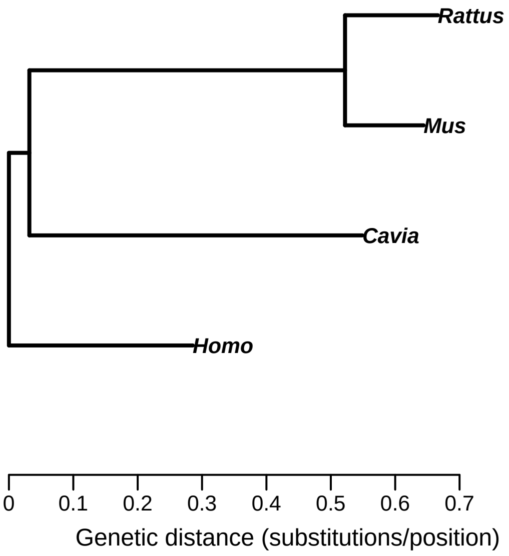

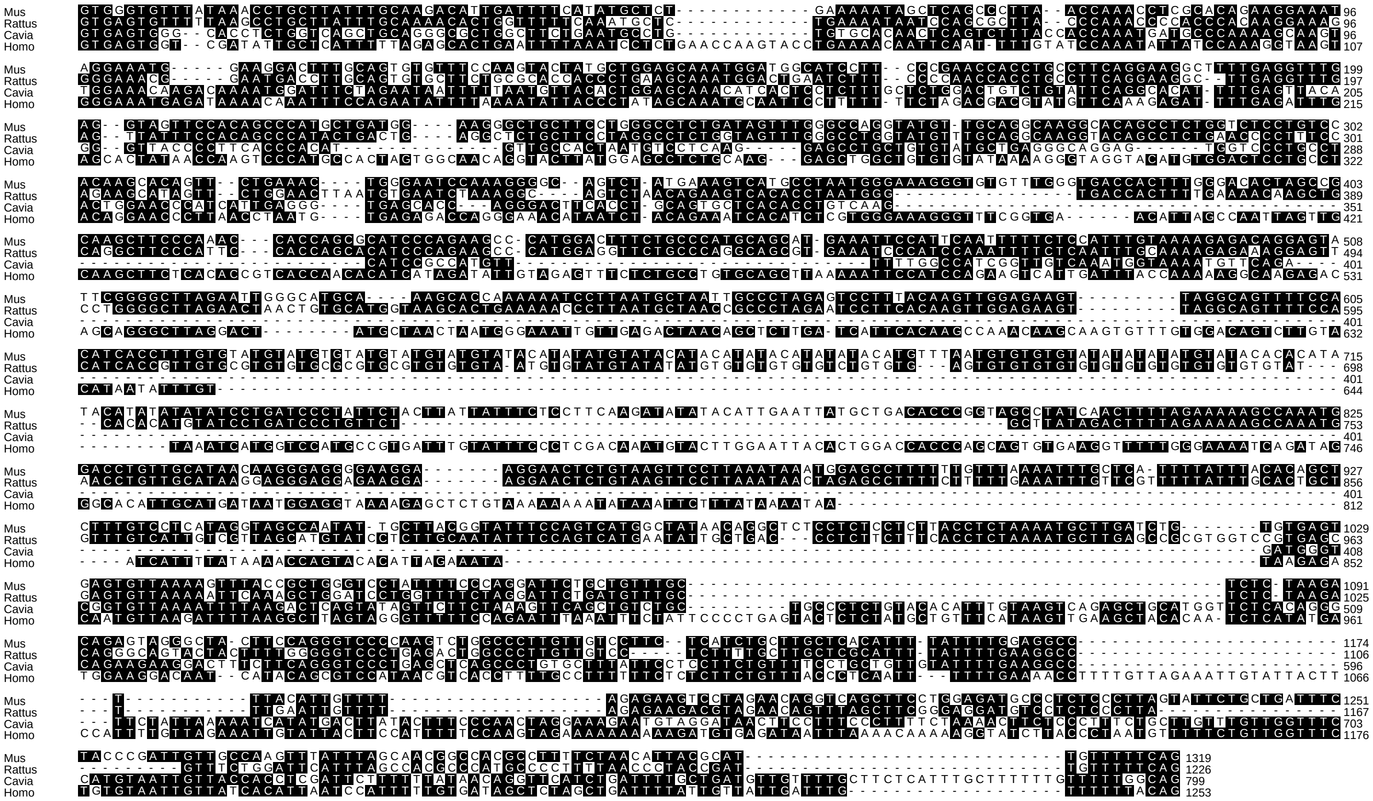

ENSMUSG00000029408 intron 2

Description: ATP-binding cassette sub-family B member 9 Precursor (Abcb9)
Intron number: 2
Mouse chromosome: 5
Upstream exon length: 115
Downstream exon length: 131
Mouse intron length: 423
Intron alignment length: 548
Total murinae branch length: 0.11990
K_score: 0.08984
Scaling factor: 0.60705

ENSMUSG00000029408 exon 2 (ORF 2)

```
Mus     GTGAGACCTTCCTGCCCTACTACACTGGCCGGGCCATTGACAGCATTGTGATCCAGAAAAGCATGGATCAGTTCACCACA 80
Rattus  GAGAGACCTTCCTGCCCTACTACACTGGCCGGGCCATCGACAGCATCGTCATCCAGAAAAGCATGGACCAGTTCACCACG 80
Cavia   GCGAGACCTTCCTGCCCTACTACACGGGCCGGGCCATCGATGGCATCGTGATCCAGAAGAGCACGGAGCAGTTCAGCACG 80
Homo    GAGAGACCTTCCTGCCCTACTACACGGGCCGCGCCATTGATGGCATCGTCATCCAGAAAAGCATGGATCAGTTCAGCACG 80

Mus     GCCGTTGTCGTCGTTTGCCTGCTGGCCATCGGCAG 115
Rattus  GCCGTCGTCGTCGTCTGCCTGCTGGCCATCGGCAG 115
Cavia   GCCGTGGTTGTCGTGTGCCTGCTGGCCGTCGGCAG 115
Homo    GCTGTCGTCATCGTGTGCCTGCTGGCCATTGGCAG 115
```

ENSMUSG00000029408 exon 3 (ORF 1)

```
Mus     CTCATTGGCCGCAGGTATTCGGGGCGGTATTTTCACCCTCGTATTTGCCAGACTGAACATTCGCCTCCGGAACTGTCTCT 80
Rattus  CTCATTGGCCGCAGGTATTCGGGGCGGCATTTTCACCCTCGTATTTGCCAGACTGAACATTCGCCTTCGCAACTGTCTCT 80
Cavia   CTCATTTGCCGCAGGTATTCGGGGCGGCATTTTTACCCTCATATTTGCCAGACTGAACATCCGCCTGCGAAACTGCCTCT 80
Homo    CTCATTTGCCGCAGGTATTCGGGGCGGCATTTTTACCCTCATATTTGCCAGACTGAACATTCGCCTTCGAAACTGTCTCT 80

Mus     TCCGCTCACTGGTGTCACAGGAGACAAGCTTCTTTGATGAGAACCGCACAG 131
Rattus  TCCGCTCCCTGGTGTCACAGGAGACGAGCTTCTTTGACGAGAATCGCACAG 131
Cavia   TCCGCTCGCTGGTGTCGCAGGAGACCAGCTTCTTTGATGAGAACCGCACAG 131
Homo    TCCGCTCACTGGTGTCCCAGGAGACAAGCTTCTTTGATGAGAACCGCACAG 131
```

ENSMUSG00000029408_intron_2

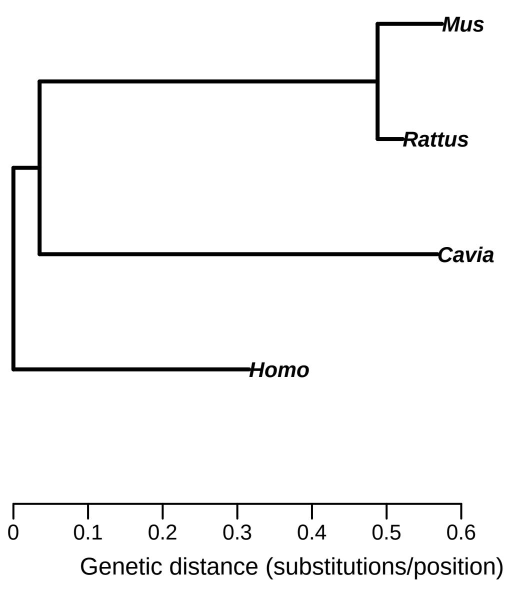

```
Mus     GTAGCCA-CCCAACCCATACACACCACT-------------CCTGCAGGAGGGAGGGCAGAAGGGAGCAGGGC------------------------------------- 59
Rattus  GTAGCCA-CCCAACCCATACATGGCACT-------------CCTGCAGGAGGGAGGGCAGGAGGGTGCAGGAC------------------------------------- 59
Cavia   GTAGCCG----------------------------------CCCGCGCCTG----------------------------------------------------------- 17
Homo    GTAGCTGGCTCAACCCCCACGCCCTGCCCCGCACACCCCGACCTGCACCAGGGAGGGAAGGGAGGGCCTGTTCCTAAGGAAGACAGGGGCCAGGGAGGCCCTGGAAATGC 110

Mus     -------------------CAGCCCAGCACTGCAGCAACCG---CCCAGACTGACGTGGAACCCTTTGGTTGGGCCACATCCCAGAGCTGTACCCTCTTGCCTCTGAAGA 147
Rattus  -------------------CGGCCCAGCACCGCGGCAACCT---CCCAAACTGACTTGGAACCCTTTGGTTGGGCCACATCCTAGAGCCATACCCTCTTGACTCTGAAAA 147
Cavia   CCCTGGGGGCCCTGTCCCTCGGCCCGTCACCTCAGTGACCGGTCCTGAGCCAGCC--------------TAGCACCTCATCCCTGCAGCAGGCAGGCCACGTCCTAAATC 113
Homo    CCCTGGTGGGCCTGTCCCTCAGCTGGTCACCCTGGGAACCGGCTTCCACACTGTC--------------TTAGA--GCACCCTAGAACCACACCCTCTGGTCCCTGAAAA 204

Mus     TGGCTCCAGTCCCCTGAAGATAGCTTTCTCTCTCCCTGGCTGGTTGGTGAATATGTAGCC---------CAAAGGATTGGTTTTCTATACTGTTCTAGA------AGGGT 242
Rattus  TGGCTCCTG-----------------------TCCCTGGCTGTTTTATGAATATATAGCC---------CAAAGGACTGGCTTTCTATACTGTTCTAGA------AGGGT 219
Cavia   GAGGCTTCAGCCCCTGAGGGTCCCCTCCATCCTGTCTGGCTGGTTT---------------------------------------------------------------- 159
Homo    GGGCTTCAGTCCTCAGGGTTCCCTGCTCACCCTCCCTGGCTGTTTTCCAAGTAGGAATCTGGTCCCAAACAGAACCGGCTTTGTCTGAGCTGTTCTGGAAGGAAGAGGGT 314

Mus     CTGGATGCCAGTGTTAAGT--------------------------CCTTTACCCTTGCTGTAGGACTTACTGGGGAGTGAGGGGTCTAGACCTGGATGTCATCCCTACAG 326
Rattus  CTGGCTGACAGTGTTGAGT--------------------------CCCTTACCCC----------------------TGGGGAGTCTAGCCCCAGATGCCATCCCTACAG 281
Cavia   ----CAGGCAGCTCTGGGTCCCCTGTCCCCA--------------CCCCTGCCCTCATGGAGGGGCCCGAGGTGG----------------TGGGGCGCCACCCTGGCAG 235
Homo    CTGGCTGGCACATTTGGGTCTCTTGTCCCACAACTGTCCCCTTTGCCCTCACCTTGAGGGGGGCTCCCACTGGGAATGAGGGGGACCCCGCAGGGATGCCAGCCCTGGAG 424

Mus     AGTGGCCTGTCCTCTGTCCT---------TGGGGAGGA--GGGGCTGCTGGTGGGTGATGCTGCCTGCCGGAAGGACACCGGCTATACCGTCTCTCATCTTCAATCAG 423
Rattus  AGTGGCCTGTCCTCTGTCCC---------TGGGGAGGAAGGGGGCTGTTGGTGGGTGGCGCTGCCTGCCGGAAGGACACCGGCTATACCGTCTCTCATCTGCAATCAG 380
Cavia   TGGGA------------------------GGAAGGATGGGAGGGCGGGCGGGTGGTGGCTGCCGCTCCTCGGAGGCCGCGCGCTATAACATCTGTTATCTGCAATTAG 319
Homo    TGGGGCTGCGGTGGGGCTCCCAGGCCTGCCGGCAGCGGGAGGGGGCAGCCGGGTGTGGTCACTGGTCTGGAAGGACATCGTGCTATAATATCTCTTATCTGCAATTAG 532
```

ENSMUSG00000039849 intron 7

Description: Phosphorylated CTD-interacting factor 1 (Pcif1)
Intron number: 7
Mouse chromosome: 2
Upstream exon length: 84
Downstream exon length: 100
Mouse intron length: 251
Intron alignment length: 299
Total murinae branch length: 0.28345
K_score: 0.04691
Scaling factor: 0.61008

ENSMUSG00000039849 exon 7 (ORF 1)

```
Mus     GTTATCCCGAATCAAGTTCCGGGAGGAAGCCAAACGCCTGCTCTTTAAATACGCAGAAGCTGCCAGGCGGCTCATTGAGT 80
Rattus  GTTATCTCGAATCAAGTTCCGGGAGGAAGCCAAACGCCTGCTCTTTAAATATGCAGAAGCTGCCAGGCGGCTCATTGAGT 80
Cavia   ATTATCCCGAATCAAGTTCCGGGAAGAAGCCAAGCGCCTGCTTTTTAAATATGCAGAGGCTGCCAGACGGCTCATCGAGT 80
Homo    GTTATCCCGAATCAAGTTCCGGGAGGAAGCCAAGCGCCTGCTCTTTAAATATGCGGAGGCCGCCAGGCGGCTCATCGAGT 80

Mus     CCAG 84
Rattus  CCAG 84
Cavia   CCAG 84
Homo    CCAG 84
```

ENSMUSG00000039849 exon 8 (ORF 1)

```
Mus     GAGTGCATCCCCTGACAGCAGAAAAGTGGTCAAATGGAACGTGGAGGACACCTTCAGCTGGCTGCGGAAAGAGCACTCGG 80
Rattus  GAGTGCATCCCCTGACAGCAGAAAAGTGGTCAAGTGGAATGTGGAGGACACCTTTAGCTGGCTGCGGAAGGAGCACTCAG 80
Cavia   GAGTGCTTCCCCTGACAGCAGGAAGGTGGTCAAGTGGAACGTGGAAGATACCTTCAGCTGGCTTCGGAAGGACCACTCGG 80
Homo    GAGTGCATCCCCTGACAGTAGGAAGGTGGTCAAATGGAATGTGGAAGACACCTTTAGCTGGCTTCGGAAGGACCACTCAG 80

Mus     CCTCCAAGGAGGATTACATG 100
Rattus  CCTCGAAGGAGGACTACATG 100
Cavia   CCTCCAAGGAGGACTACATG 100
Homo    CCTCCAAGGAGGACTACATG 100
```

ENSMUSG00000039849_intron_7

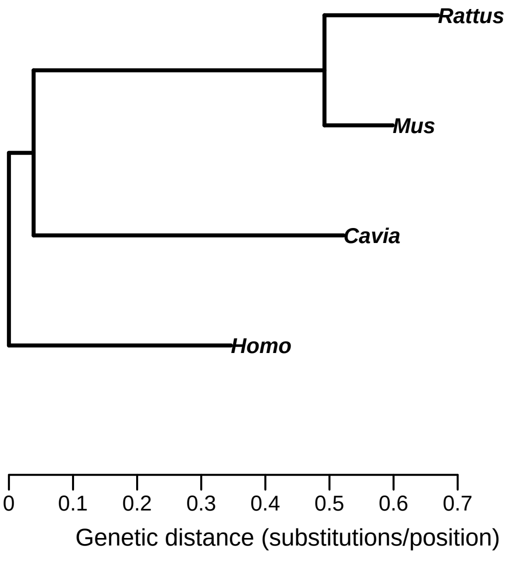

```
Mus     GTTTGGCT-TTTTCTGCCCCACACAATGAAAATGGTGG------GTGGGGATGTGTGTGTTCGCACATGTATACATGAAACATACACGCTGTCATCTGGCTAATGCTCAC 103
Rattus  GTTTGGCT-TTTTCTGCCCCACACAATGAAAATGGTGT------GTGCGTGTGCGTGTGCGTGTGTGTGTGCTCGCG---------------CATTTGCCTAATGCTCAC 88
Cavia   GTTTGGCT-CTCTCTGCCCCACATAAGACTGATCCACG------GGGGGAGATTGTAGGCC-------------------------------TGCTCTCCTTACCCACTC 72
Homo    GTTTGCCTGTCTTCTGCCCCAGGCGAGATGGGTCTGTGATTAAAGTGGCAGGTCATAGGC--------------------------------CATCTTGCCCAGTCTCAT 78

Mus     GCAGTTTTGCCAGTGTTACCAAAGACAGATGGGAGGTAAGC--------------------------------GTAGAGGCTAGTGCCTGAGGTGGCAGTGAGGCAGGAG 181
Rattus  ACAGGTGTGTCAATGCTT---AAGACAGATGGGACGTAAGC--------------------------------TTAGAGGCTAGTGTCTGAGATGGTAGTCTTATCGGGA 163
Cavia   AGCACTGCATCTGTGCCCCCAGATACAGAACTCAGGCTAAGTGCATGC-------------------------CTAGGAGTTGACACCCAAGACACCAGTGAAGGGGAAT 157
Homo    GCAGGGCTGTCATTGCTCTCGGTGGCAGAAGTGGGGCCAGCTCCCAAAGGCAGAACAAGGGCTGTGATGGAAGCTAGGGGTTGATACCCAGCGAGCCCATGGAATTGGGA 188

Mus     GAGGGGA----GCAGCAAGACACCTAACAGCTGGGCCC--CAAGAACCACAGCTTTCT---CCTGCTCACAACCTGCAG 251
Rattus  CAAGGAA--------CAAGACACCTAACAGCTGAGCCCCACACAAAACACAGTGTTCT---CTTGCTATCAACCTGCAG 231
Cavia   GGGAAGAGGGTGGGGCAGACCCCTCAGTGGCTGGGTCCTGTGCACGAACGAGCCCTCTTTCCTTGCCTTTGACTGGCAG 236
Homo    TGAGGAGGGTGGAGCCAAGCCATTCCTT-------------------------TCTTCTGCCCTCCCCTTGACTGGCAG 242
```

ENSMUSG00000006021 intron 9

Description: Kaptin (Kptn)
Intron number: 9
Mouse chromosome: 7
Upstream exon length: 76
Downstream exon length: 136
Mouse intron length: 920
Intron alignment length: 1532
Total murinae branch length: 0.18335
K_score: 0.0796
Scaling factor: 0.61043

ENSMUSG00000006021 exon 9 (ORF 2)

```
Mus     CAACACAGGACAGCCCCCAGCAGGAAGGATACAGTCTGCTTGTGGCCAGCATGCTGGAGCCAGCTGTGGTGTACTG 76
Rattus  CAACCAAGGACAGCCCCCAGCAGGAAGGATACAGTCTGCTTGTGGCCAGCATGTTGGAGCCGGCTGTGGTATACTG 76
Cavia   AGGCTGAGGACAGGCCCCAGCAGGAGGAGTACAGTGTGCTCGTGGCCAGTATGGTGGAGCCAGCTGTGGTGTACCG 76
Homo    AGACCAAGGACAGGCCACTACAAGATGAGTACAGCGTGCTCGTGGCCAGCATGTTGGAGCCAGCAGTGGTGTATCG 76
```

ENSMUSG00000006021 exon 10 (ORF 1)

```
Mus     GGATCTTCTGAACAAGGGTCTGGATGACCAACTTCTCCTGCCTGGCAGTGACCAGTTTGACAGCGTCCTCTGTGGCCTGG 80
Rattus  GGACCTTCTGAACAAAGGCCTGGATGACCAACTTCTCCTGCCTGGCAGTGACCAGTTTGACAGCGTACTCTGCGGGCTGG 80
Cavia   GGACCTACTCAATCAAGGCCTTGAGAACCAGCTTCTCCTGCCCGGCAGTGACCAATTTGACAGCATCCTCTGTGGCCTGG 80
Homo    GGACCTGCTGAACCGGGGTCTTGAAGACCAGCTTCTCCTGCCCGGCAGTGACCAGTTTGACAGCGTCCTCTGCAGCCTGG 80

Mus     TCACTGACGTGGACCTAGATGGACAACCGGAAGTCCTGGTGGCCACCTATGGACAG 136
Rattus  TCACTGACGTGGACCTAGATGGACAACCGGAAGTCCTGGTGGCCACCTATGGACAG 136
Cavia   TCACTGATGTGGACTTGGATGGACAGCCAGAAGTCCTGGTGGCCACCTACGGCCAG 136
Homo    TCACCGATGTGGATTTGGATGGGCGGCCAGAAGTCCTGGTGGCCACCTATGGACAG 136
```

ENSMUSG00000006021_intron_9

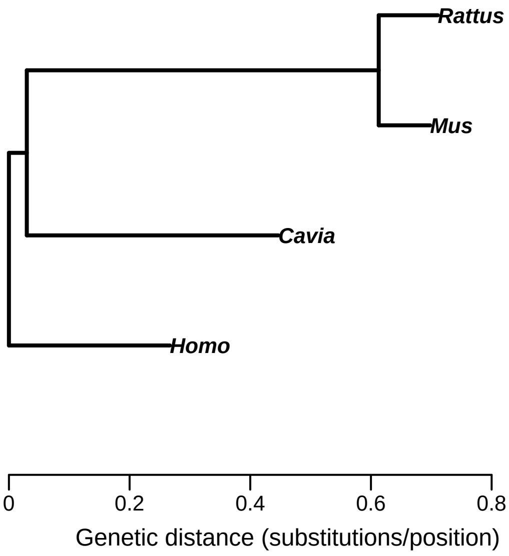

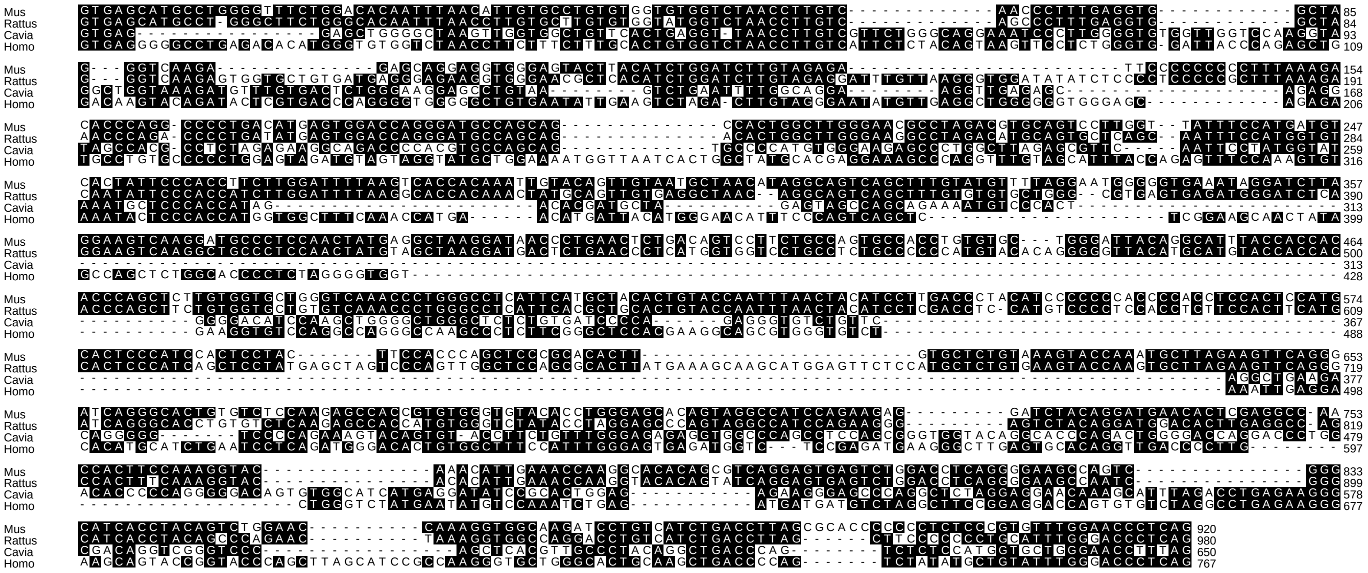
Mus
Rattus
Cavia
Homo
85
84
93
109
154
191
168
206
247
284
259
316
357
390
313
399
464
500
313
428
574
609
367
488
653
719
377
498
753
819
479
597
833
899
578
677
920
980
650
767

ENSMUSG00000014453 intron 3

Description: Tyrosine-protein kinase BLK (Blk)
Intron number: 3
Mouse chromosome: 14
Upstream exon length: 94
Downstream exon length: 99
Mouse intron length: 811
Intron alignment length: 1056
Total murinae branch length: 0.16558
K_score: 0.06904
Scaling factor: 0.61055

ENSMUSG00000014453 exon 3 (ORF 2)

```
Mus     AGGAGCGTTTTGTGGTGGCTCTGTTTGACTATGCCGCTGTGAATGACAGGGACCTTCAGGTGCTGAAGGGTGAGAAGCTC 80
Rattus  AGGAGCGTTTTGTGGTGGCCCTGTTTGACTATGCCGCTGTGAATGACAGGGACCTGCAAGTGCTAAAGGGAGAGAAGCTC 80
Cavia   AGGACCATTACGTGGTGGCCCTGTACGACTACGCTGCTGTGAACGACCGGGACCTGCAAATGTGCAAGGGGGAGAAGCTG 80
Homo    ACAAGCATTTCGTGGTGGCTCTGTATGACTACACCGCTATGAATGATCGGGACCTGCAGATGCTGAAGGGGGAGAAGCTA 80

Mus     CAGGTCTTGAGGAG 94
Rattus  CAAGTCTTGAAGAG 94
Cavia   CAGGTCTTGAAGGA 94
Homo    CAGGTCCTGAAGGG 94
```

ENSMUSG00000014453 exon 4 (ORF 1)

```
Mus     CACTGGAGACTGGTGGTTGGCCAGGTCACTCGTCACAGGAAGAGAAGGTTATGTGCCCAGCAACTTTGTGGCCCCAGTAG 80
Rattus  CACTGGAGACTGGTGGTTGGCCAGGTCACTCGTCACAGGAAGAGAAGGCTATGTGCCCAGCAACTTTGTGGCCCCAGTAG 80
Cavia   GACTGGGGACTGGTGGTTGGCCAAATCACTCATCACCGGAAGAGAGGGCTACGTGCCCAGCAACTTCGTGGCCCCTGTGG 80
Homo    AACTGGAGACTGGTGGCTGGCCAGGTCACTCGTCACAGGAAGAGAAGGCTATGTGCCCAGTAACTTTGTGGCCCGAGTGG 80

Mus     AGACTCTGGAAGTAGAAAA 99
Rattus  AGACCCTGGAAGTAGAAAA 99
Cavia   AGACCCTGGAAGTGGAAAA 99
Homo    AGAGCCTGGAAATGGAAAG 99
```

ENSMUSG00000014453_intron_3

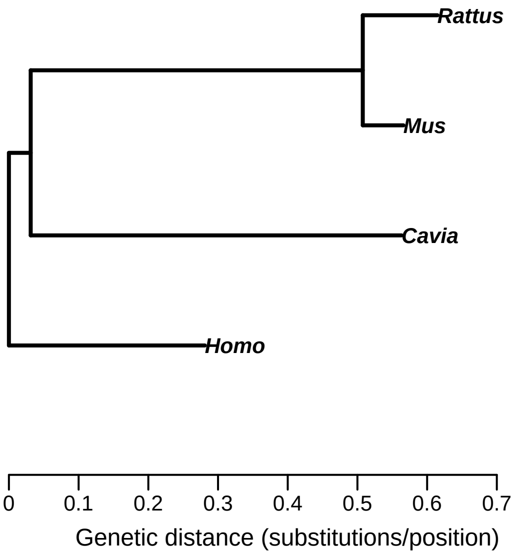

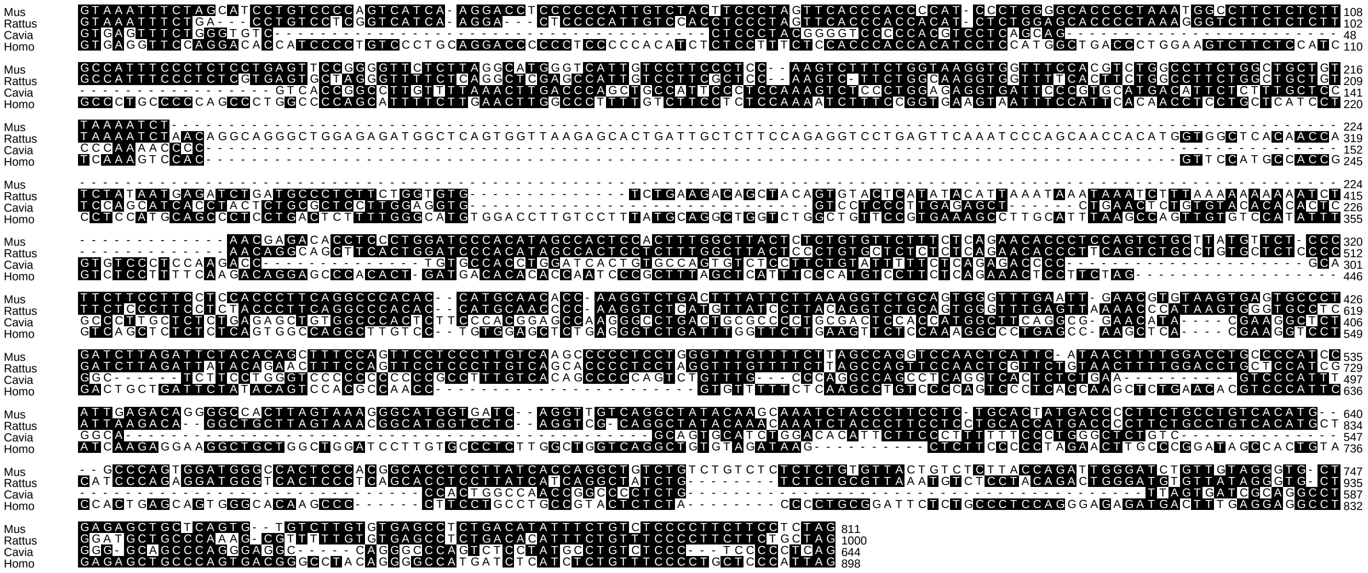
Mus
Rattus
Cavia
Homo

ENSMUSG00000037706 intron 5

Description: CD81 antigen (Cd81)
Intron number: 5
Mouse chromosome: 7
Upstream exon length: 105
Downstream exon length: 102
Mouse intron length: 308
Intron alignment length: 376
Total murinae branch length: 0.16972
K_score: 0.06904
Scaling factor: 0.61246

ENSMUSG00000037706 exon 5 (ORF 0)

```
Mus     ATCGCCAAGGATGTGAAGCAGTTCTATGACCAGGCCCTTCAGCAAGCTGTGATGGATGATGATGCCAACAATGCCAAGGC 80
Rattus  ATCGCCAAGGATGTGAAGCAGTTCTACGACCAGGCCCTTCAGCAGGCTGTGATGGATGATGATGCCAACAATGCCAAGGC 80
Cavia   ATTGCCAAGGACGTGAAGCAGTTCTACGACCAGGCTTTGCAGCAGGCTGTGGTAGATGACGATGCTAACAACGCCAAGGC 80
Homo    ATCGCCAAGGATGTGAAGCAGTTCTATGACCAGGCCCTACAGCAGGCCGTGGTGGATGATGACGCCAACAACGCCAAGGC 80

Mus     TGTGGTGAAGACTTTCCATGAGACG 105
Rattus  AGTGGTGAAGACCTTCCATGAGACG 105
Cavia   TGTGGTGAAGACTTTCCATGAGACG 105
Homo    TGTGGTGAAGACCTTCCACGAGACG 105
```

ENSMUSG00000037706 exon 6 (ORF 0)

```
Mus     CTCAACTGTTGTGGCTCCAACGCACTGACCACACTGACTACCACCATACTGAGGAACAGCCTGTGTCCCTCAGGCGGCAA 80
Rattus  CTCAACTGTTGTGGCTCCAATACGCTGACCACACTGACCACCACCGTGCTGAGGAACAGCCTGTGTCCCTCAAGCAGCAA 80
Cavia   CTCAACTGCTGTGGCTCCAACACATTGATTGCGCTGTCCAATTCGGTGCTGAGGAACAACCTGTGTCCATCGGGCACCGC 80
Homo    CTTGACTGCTGTGGCTCCAGCACACTGACTGCTTTGACCACCTCAGTGCTCAAGAACAATTTGTGTCCCTCGGGCAGCAA 80

Mus     CATACTCACCCCCTTACTGCAG 102
Rattus  CTCATTCACTCAGCTTCTGAAG 102
Cavia   CTTCATAAATACCTTATGGAAG 102
Homo    CATCATCAGCAACCTCTTCAAG 102
```

ENSMUSG00000037706_intron_5

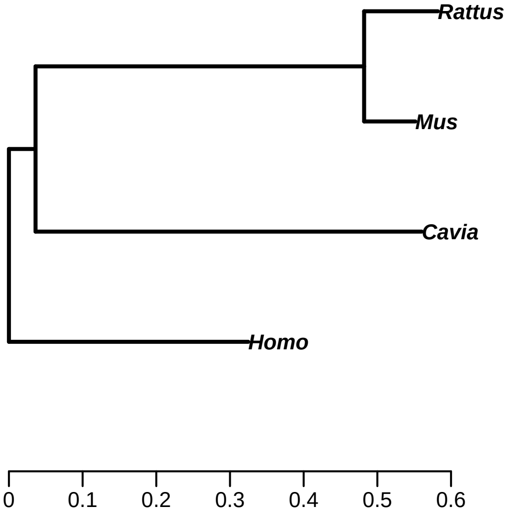

Genetic distance (substitutions/position)

```
Mus     GTATAGC----TAGGCTCAAGCAG-GAGGGGAGGCTGTGGGGTTCCTAGTGTGTGAGGGCATGGGTCTCCTTTC-------TTTCCAACACAACAGGGCCTAGGAA---- 94
Rattus  GTACAGC----TAAGCTGGAGCAG-GAGGGCAGGTTGTGGG-------GTGTGTGGGGGCATGAGTCCCAGATC-------TCT-----GTAACAAGACCTGGGAA---- 82
Cavia   GTGTGGCTGGCTGGGCTAGGCCAGCAGGGATGGGCTACAGGACCCAGGTGGGGTGGGGTGGGGTGTGCCTTCTTGGAGGAGTCCTGCCTCAGCCCAGACCTCAGGATCTG 110
Homo    GTGCGGC--------CCCGGGGGGCGAGGGCGGGGAGCAGGGCCCCGGGAACCCGGCGGGGTGTGTCTCGTCCTGGATGAATCCTGCCTACGCCCAGACCTCAGGAGCAG 102

Mus     -----------AGGGTCTCC----------TCTATGATTTGTGGGTCCTCAGGTAGGAATCTAATGGGCCTCATTTTCCCAGGGGTTTGAAGGAACAGGGCTCCTGTGCA 183
Rattus  -----------AGGTTCTCC----------TCTATGATT--AGATTCCCCAGGTAGGAATCTAATGGGCCTCGTTTTCCCAGGGGTTTGGAGGAATGGGACTTGTGTGCA 169
Cavia   TCTATGGTAGAGGGGTCTCC----------TCTATGACT----TAGTTCCTAGTGGGAACCTGTTGAACCTGGCCA---------GTGGGTTT--------GTCTGAGCA 189
Homo    GAGGTGCCCTTGGGACCTCCAGGACCCCTGGTCTCAACT----GGTCCTCGGGTGGGAACCTAGTGGGCCAGGGTGGCCCAGGGTGCGGAAAG--------CTCTGAGCA 200

Mus     GGGTGACAAACAGGGCAGAAG----GACAACTCCCAAGTG--TATTTTGGGGTGACGGTGTGCTGTGTGCC--TGATCTTTCCCTT-----------------------G 262
Rattus  GGGTGACCAACCAGGCAGAAGGACAGACAGCTCCCAAGTGTATATTTTGGGGTGATGCTGTGCTGTGGGCT--TGATCTTTCCCT------------------------- 252
Cavia   GTGCCTCTGGCTGGGGAGGAA----GGCTGGCCCTGGGTG--CACGCGGGATAG-CAGTGTAATGCACATCTGTGGCCTTTTCTTC-TCCATTGCCTGG----------- 280
Homo    GCGCAGCTGAGGAGGAAGAAG----GCTGGCCCCTGGATG--CATTCTGCAGTG-GGGAGCGCTGCGTACCCCTGGCCACCTCCCCATGGGTTCCCTAGAGCCACCGTCC 303

Mus     TCTCCTGAAGCTCCAAAGCTGACAGTGTTGTTCTTGGTCCCTGCAG 308
Rattus  -------------------TGACAGTGTTGTTCTTGGTCTCTGCAG 279
Cavia   --------------TGAGATGACCACAGTGCTC-TTATCTCTACAG 311
Homo    CCCTGGGCACATCCAGGGCTGACCTTGCACCCC-TGCTCTCTGCAG 348
```

ENSMUSG00000034853 intron 10

Description: Acyl-coenzyme A thioesterase 11 (Acot11)
Intron number: 10
Mouse chromosome: 4
Upstream exon length: 56
Downstream exon length: 67
Mouse intron length: 1165
Intron alignment length: 1472
Total murinae branch length: 0.17427
K_score: 0.08549
Scaling factor: 0.61374

ENSMUSG00000034853 exon 10 (ORF 0)

```
Mus     GAGGGTGAACGGCGATACCGAGAAGCCAGTGCCAGGAAGAAGATCCGCCTGGACAG---- 56
Rattus  GATGGTGAACGGCGATACCGAGAGGCCAGTGCCAGGAAGAAGATCCGCCTGGACAGGCGA 60
Cavia   GATGGTGAGCGGCGCTACAGAGAAGCCCAAGCCAGGAAGAAGATCCGGCTGGACAG---- 56
Homo    GATGGTGAGCGGCGGTACCGAGAGGCCAGTGCCAGAAAGAAGATCCGCCTGGACAG---- 56
```

ENSMUSG00000034853 exon 11 (ORF 1)

```
Mus     GAAATACCTTGTGTCCTGTAAGCAGGCAGAAGTGGCCCTGTCTGTCCCCTGGGACCCTAGCAACCAG 67
Rattus  ----TACCTTGTGTCCTGTAAGCAGGCGGAAATGGCCCTGTCTGTTCCCTGGGATCCTAGCAACCAG 63
Cavia   GAAGTACATTGTGTCCTGCAAGCAGGCGGAAGTGCCGCTCTCTGTGCCCTGGGACCCTAGCAACCAG 67
Homo    GAAGTACATCGTGTCCTGTAAGCAGACAGAGGTGCCCCTCTCCGTCCCCTGGGACCCTAGCAACCAG 67
```

ENSMUSG00000034853_intron_10

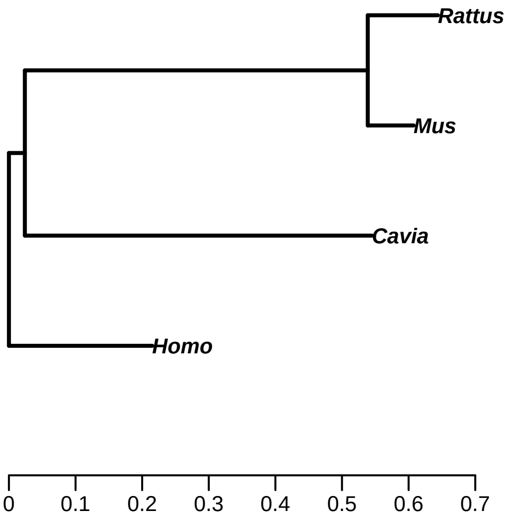

Genetic distance (substitutions/position)

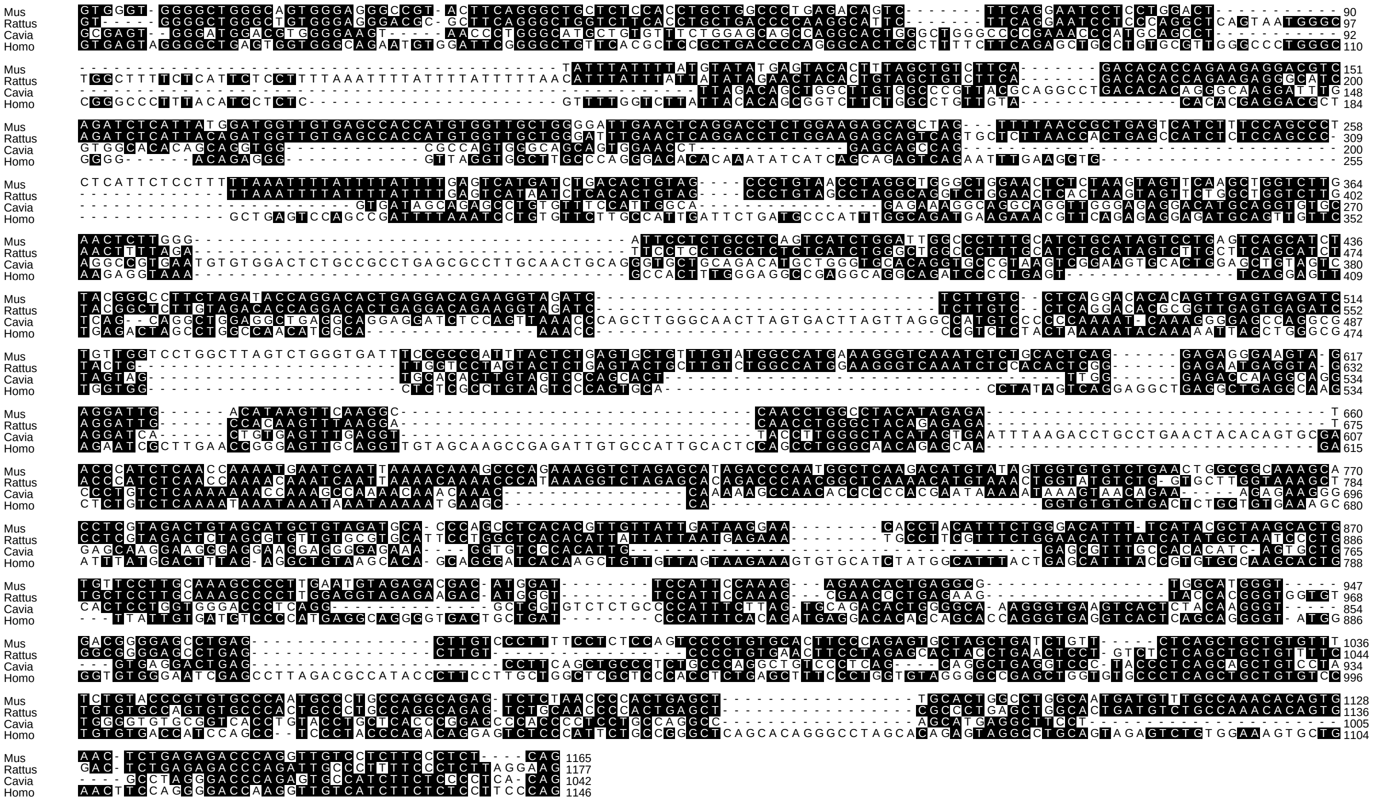
Mus
Rattus
Cavia
Homo

ENSMUSG00000028582 intron 17

Description: Coiled-coil and C2 domain-containing protein 1B (Cc2d1b)
Intron number: 17
Mouse chromosome: 4
Upstream exon length: 117
Downstream exon length: 74
Mouse intron length: 258
Intron alignment length: 345
Total murinae branch length: 0.25564
K_score: 0.05116
Scaling factor: 0.61914

ENSMUSG00000028582 exon 17 (ORF 1)

```
Mus     GTTTGAGAGGCTTGCGGAGGACCGAAAGAAACAGCTTGAGATCCTGCAGCTAGCCCAAGCCCAGGGCCTTGACCCTCCTA 80
Rattus  GTTTGAGAAGCTTGCTGAGGACCGAAAGAAACAGCTTGAGATCCTGCAGCTGGCCCAAGCCCAGGGCCTGGACCCTCCCA 80
Cavia   GTTTGAGAAGCTTGCTCATGACCGCAAGAAACAGCTGGAGATCTTGCAGCTGGCCCAGGCCCAGGGCCTCGACCCTCCCA 80
Homo    ATTTGAGAAGCTTGCTCAGGACCGCAAGAAACAGCTGGAGATCCTGCAGCTGGCCCAGGCTCAGGGCCTCGACCCTCCCA 80

Mus     GTCATCACTTTGAGCTGAAGACATTCCAGACTGTGAG 117
Rattus  GTCATCACTTTGAATTGAAGACATTGCAGACTGTGAG 117
Cavia   GCCACCACTTTGAGCTGAAGACATTTCAGACTGTGAG 117
Homo    CCCACCACTTTGAGTTGAAGACATTCCAGACTGTGAG 117
```

ENSMUSG00000028582 exon 18 (ORF 1)

```
Mus     GATCTTCTCAGAACTCAACAGCACAGAAATGCATCTGATCATCGTGCGGGGAATGAACCTCCCAGCCCCGCCAG 74
Rattus  GATCTTCTCAGAACTCAATAGCACAGAAATGCATCTGATCATCGTCCGGGGAATGAACCTCCCAGCCCCACCAG 74
Cavia   GATCTTCTCAGAACTCAACAGCACAGAAATGCATCTGATCGTTGTCCGGGGAATGAACCTGCCAGCCCCTCCAG 74
Homo    GATCTTCTCAGAACTCAACAGCACAGAAATGCATCTGATCATTGTCCGGGGAATGAACCTCCCAGCCCCTCCAG 74
```

ENSMUSG00000028582_intron_17

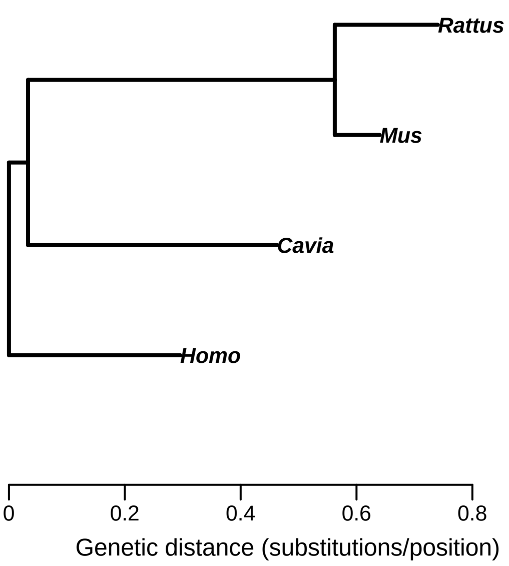

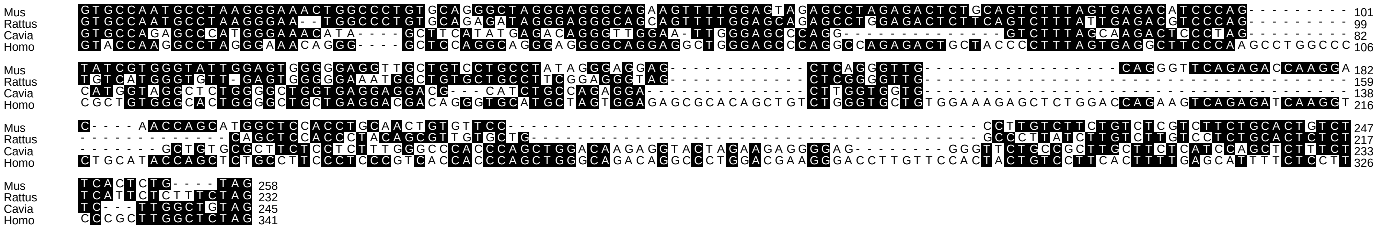

ENSMUSG00000030693 intron 1

Description: kallikrein related-peptidase 10 (Klk10)
Intron number: 1
Mouse chromosome: 7
Upstream exon length: 91
Downstream exon length: 184
Mouse intron length: 1110
Intron alignment length: 1852
Total murinae branch length: 0.18863
K_score: 0.04681
Scaling factor: 0.61939

ENSMUSG00000030693 exon 1 (ORF 0)

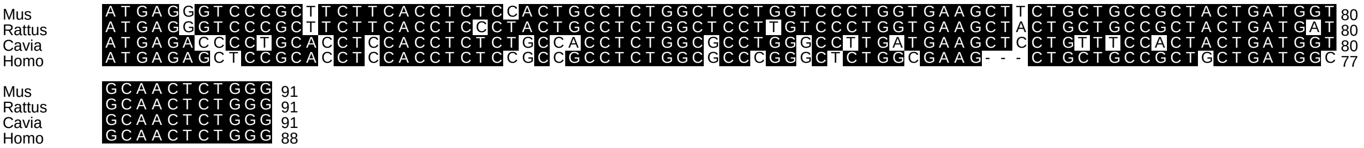

ENSMUSG00000030693 exon 2 (ORF 2)

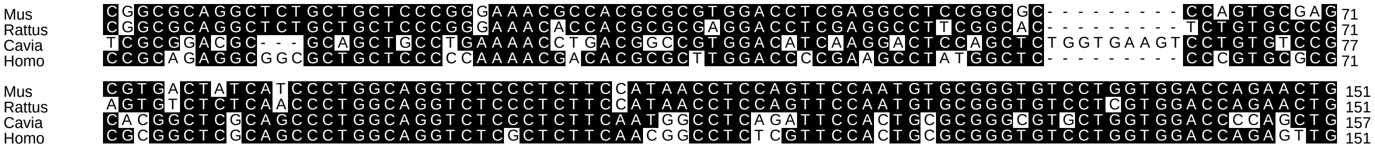

ENSMUSG00000030693_intron_1

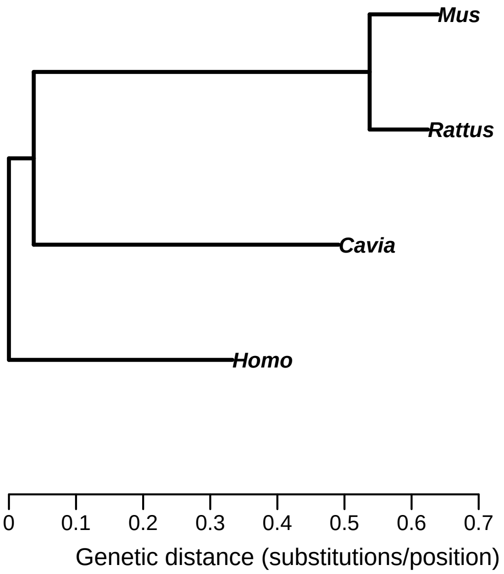

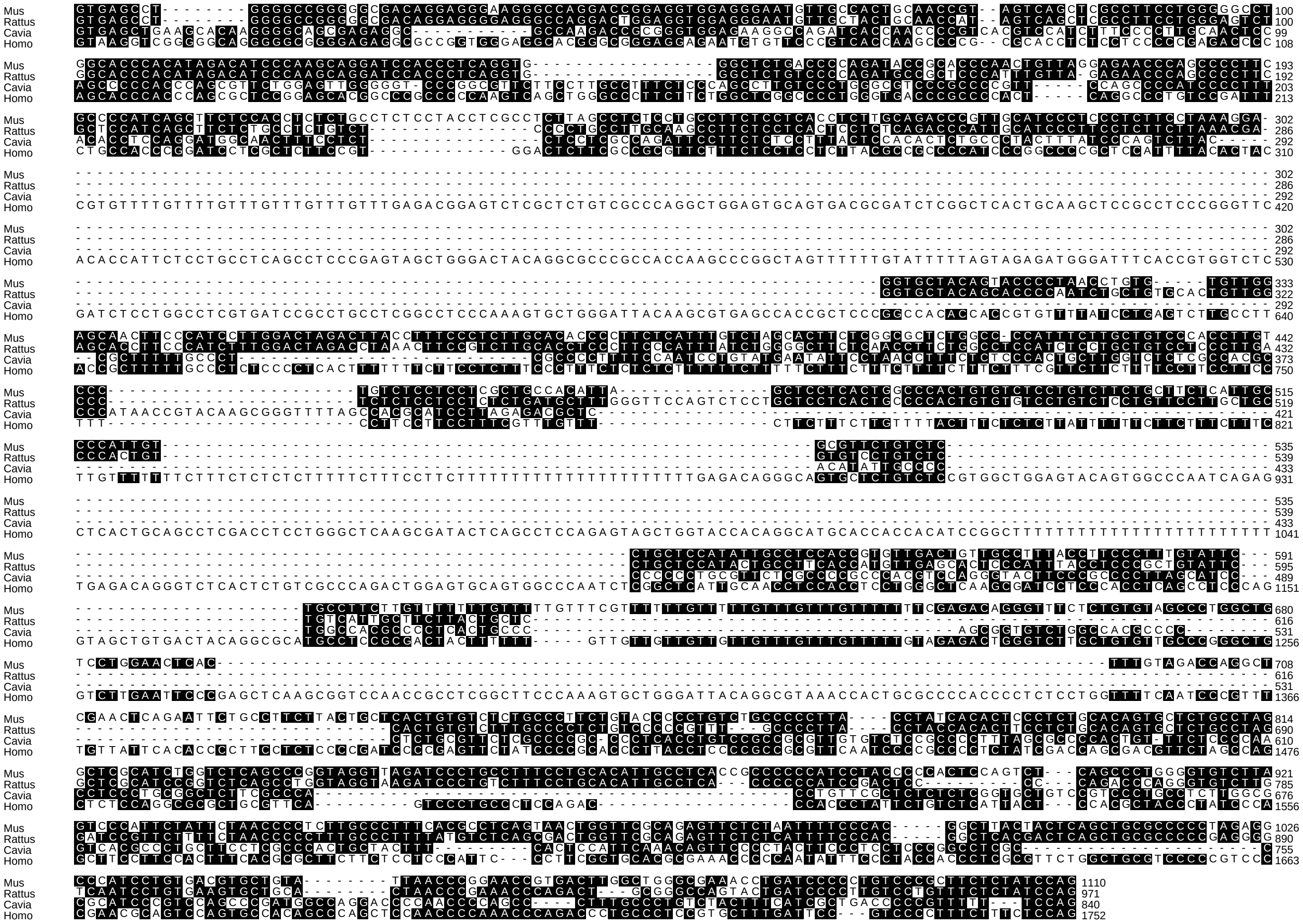
Mus
Rattus
Cavia
Homo

ENSMUSG00000041523 intron 3

Description: Uroplakin-2 Precursor (Upk2)
Intron number: 3
Mouse chromosome: 9
Upstream exon length: 139
Downstream exon length: 71
Mouse intron length: 241
Intron alignment length: 267
Total murinae branch length: 0.22255
K_score: 0.07575
Scaling factor: 0.62148

ENSMUSG00000041523 exon 3 (ORF 2)

```
Mus     TGGTTAAGTCAGACTTTGTGGTGCCTCCATGTCGCGGGCGCAGGGAGCTTGTGAGCGTGGTGGACAGTGGGTCTGGCTAC 80
Rattus  TGGTTAAGTCGGGCTTTGTGGTGCCTCCGTGCCGTGGGCGCAGGGAGCTTGTGAGCGTGGTGGACAGCGGGGCAGGCTTC 80
Cavia   TGGTGAACTCCAGCTTTGTGGTCCCTCCGTGCCGAGGACGCAGGGACCTGGTGAGTGTGGTTGACAGCGGGGCTGGCTTC 80
Homo    TGGTGACGTCCAGCTTTGTGGTGCCTCCGTGCCGTGGGCGCAGGGAACTGGTGAGTGTGGTGGACAGTGGTGCTGGCTTC 80

Mus     ACCGTCACAAGGCTCAGCGCATATCAGGTGACAAACCTAACACCAGGAACCAAATACTA 139
Rattus  ACCGTCACAAGGCTCAGCGCATATCAGGTGACAAACCTCACACCAGGAACCAAATACTA 139
Cavia   ACGGTCACGAGGCTCAGTGCGTACCAGGTGATGAACCTCACACCAGGAACCAAATACTA 139
Homo    ACAGTCACTCGGCTCAGTGCATACCAGGTGACAAACCTCGTGCCAGGAACCAAATTCTA 139
```

ENSMUSG00000041523 exon 4 (ORF 1)

```
Mus     CATATCCTACCGAGTACAGAAGGGGACATCCACCGAGTCCAGTCCAGAGACTCCCATGTCCACGCTTCCTC 71
Rattus  CATATCCTACCGAGTACAGAAGGGGATGTCCACTGAGTCCAGTCCAGAGACTCCCATGTCCACGCTTCCCC 71
Cavia   CATCTCCTACCGAGTACAGAAGGGGACGTCCACTGAGTCCAGTCGGGAGATCACAATGTCCACGCTTCCTC 71
Homo    CATTTCCTACCTAGTGAAGAAGGGGACAGCCACTGAGTCCAGCAGAGAGATCCCAATGTCCACACTCCCTC 71
```

ENSMUSG00000041523_intron_3

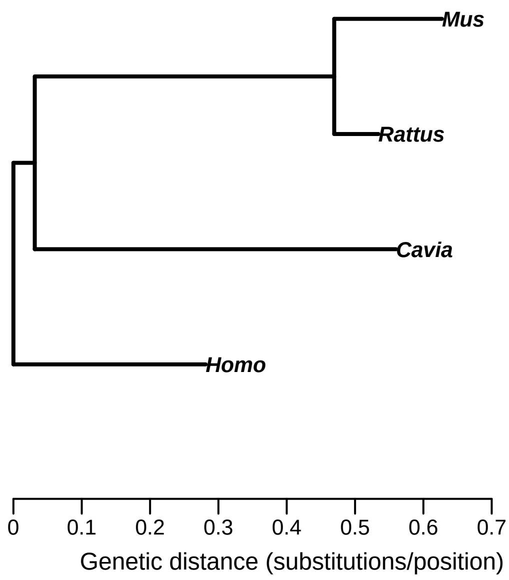

```
Mus     GTAGGTACCGA---------TGGACACCTGTGGAGGTGGGATGCAAAAAAGGGAAGTGGAGGTCCCGTGAGGGTGGGGAAGTGCCAGGAAGCATGAGT---TAGAGAGGG 98
Rattus  GTAGGTACCAA---------CGGACACCCGTGGGGGTGGGACACA-GAAAGGGGAGCGGAGGTGGGGTGAGAGTAGGGAGTTGCTGGTAAGCATGGGT---TAGGGAGGG 97
Cavia   GTAAGTACCTGACCCAGGCCTGGACACCTGTGCC-TTGGGATGCAGGAGGAAA----------------AGCTGGGGAGGGGACCGGTCAGCGTGGGGACATTGCGCAGA 93
Homo    GTAGGTACTGGGTCCAGCCTGAGGCACCTAGGGA-AGGGGGTGCAGGAAGAGAGAGAA--------GCTAGTGTGGGGGAGATCTGGTGGGCATGAGGAGAGGGTGAGGG 101

Mus     CACAGCTAAAGGGTAGGAAATGTGAACCTGGACCCCAGGAGGGCCCAGATGGGACACATAGCTA--------GAAGGTGGAGGCTGGAACCCCTCCTCCCGAGTG-CCAG 199
Rattus  TGCAGCTAAAGGGTATGAAATGTGAACCTCCACCCCAGGAGGGCACAAATGGGAGGCATAACTG--------GAAGATGGAGGCTGGAACCCCTCCTCCCACGTG-CCAG 198
Cavia   CCCAGC--------------------------CTGGGGCAGGGTACAA--CGGAGGCACAGCTGTGCCC------------------AGCTCCTCTTC--------CCTG 149
Homo    CACAGCAAAAGGGGGTGAAAGCTGGGCCTC--TTGGAGGAGTGCACATAGGGGAGACACAGCTGTGAGGGTAGGTGGCCCAGGCTGGAGCCCCTCTTCCTGTAAGTCCCA 209

Mus     ATACGTAC-----AATCTCTGCTTTCTCTCAACTCCTGCCTCTAAAG 241
Rattus  ATACCTACTGGGGGGTCTCTGCTTTCTCTCAACTCCCACCTCTAAAG 245
Cavia   ATACTCACTGAGG-----CCTCTGTTCCCCAAACACCACCCCAAAAG 191
Homo    ATACTCACTGAGGGTTCTCTACTCTCTCCCAA-----ACCACAAAAG 251
```

ENSMUSG00000026354 intron 16

Description: lactase (Lct)
Intron number: 16
Mouse chromosome: 1
Upstream exon length: 231
Downstream exon length: 218
Mouse intron length: 1119
Intron alignment length: 1614
Total murinae branch length: 0.22392
K_score: 0.03662
Scaling factor: 0.62672

ENSMUSG00000026354 exon 16 (ORF 2)

```
Mus     GGCTTCGCAGAGAGGTTCGGCGTGCACTTTGTGAACCGCTCTGACCCTTCTTTGCCAAGGATCCCCAAGGCGTCAGCCAA 80
Rattus  GGCTTCGCAGAGAGGTTCGGTGTGCACTTTGTGAACCGCTCTGACCCATCTTTGCCAAGGATCCCCAAGGCATCAGCCAA 80
Cavia   GGCTTCTCAGAGAGGTTTGGCTTGTACTATGTGAACCACACAGACCCTTCTCTGCCAAGAATCCCTAGAGCATCAGCTAA 80
Homo    GGCTTTTCAGAGAGATTTGGTCTGCATTTTGTGAACTACAGTGACCCTTCTCTGCCAAGGATCCCCAAAGCATCAGCGAA 80

Mus     GGTCTACGCCTCCATAGTCCGCTGCAATGGCTTTCCTGACCCTGCACAAGGACCTCATCCTTGTCTCCAGCAACCAGAAG 160
Rattus  GTTCTATGCCACCATCGTCCGCTGCAATGGCTTTCCCGACCCTGCACAAGGACCTCATCCTTGTCTCCAGCAACCAGAAG 160
Cavia   GTTCTATGCTTCCATAATCCGGTGCAATGGCTTCCCTGACCCGGCAGAGGGGCCTCACCCTTGTCTCCAGCAGCCAGAAG 160
Homo    GTTCTACGCCTCTGTGGTCCGATGCAATGGCTTCCCTGACCCCGCTACAGGGCCTCACGCTTGTCTCCACCAGCCAG--- 157
```

ENSMUSG00000026354 exon 17 (ORF 2)

```
Mus     ATGCTGGGCCCACTGCCAGCCCTGTGA---AGTCAGAGGTGCCCTTCCTGGGGCTGATGCTGGGCACGGCAGAAGCACAG 77
Rattus  ACGCTGCACCCACTGCCAGTCCCGTGC---AGTCGGAAGTGCCCTTCCTGGGGCTGATGCTGGGCATCGCAGAAGCACAG 77
Cavia   ATGCTGGTTCCACCATCAGCCCTGAGA---GAGAGGACGTGCCATTCCTGGGGTTGATGCTCGGCACCACAGAAGCACAA 77
Homo    ATGCTGGACCCACCATCAGCCCCGTGAGACAGGAGGAGGTGCAGTTCCTGGGGCTAATGCTCGGCACCACAGAAGCACAG 80

Mus     ACGGCTCTGTATGTCCTCTTCGCTCTTGTGCTGCTTGGAGTTTGCAGCGTGGCATTTCTGTTATACAAATACTGCAAGCG 157
Rattus  ACAGCGCTGTACGTGCTCTTCGCTCTGCTGCTGCTTGGAGCCTGCAGCCTGGCATTTCTGACCTACAAATACTGCCGGCG 157
Cavia   ACAGCTCTGTACACACTCTTTTCTCTCATGCTTCTTGGAGTCTGTGGCTTGGCATTTCTAACATACAAATACTGCAAGCT 157
Homo    ACAGCTTTGTACGTTCTCTTTTCTCTTGTGCTTCTTGGAGTCTGTGGCTTGGCATTTCTGTCATACAAGTACTGCAAGCG 160
```

ENSMUSG00000026354_intron_16

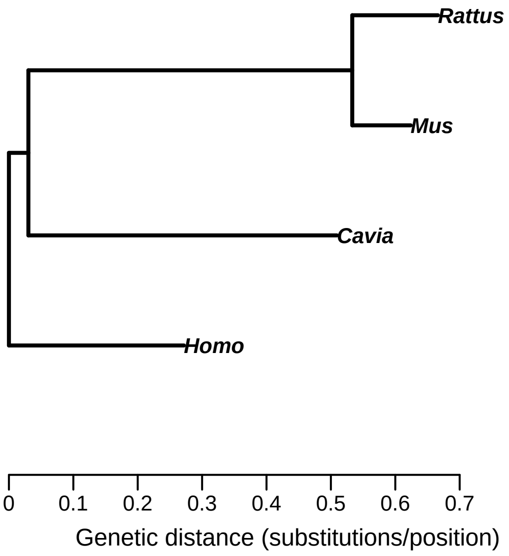

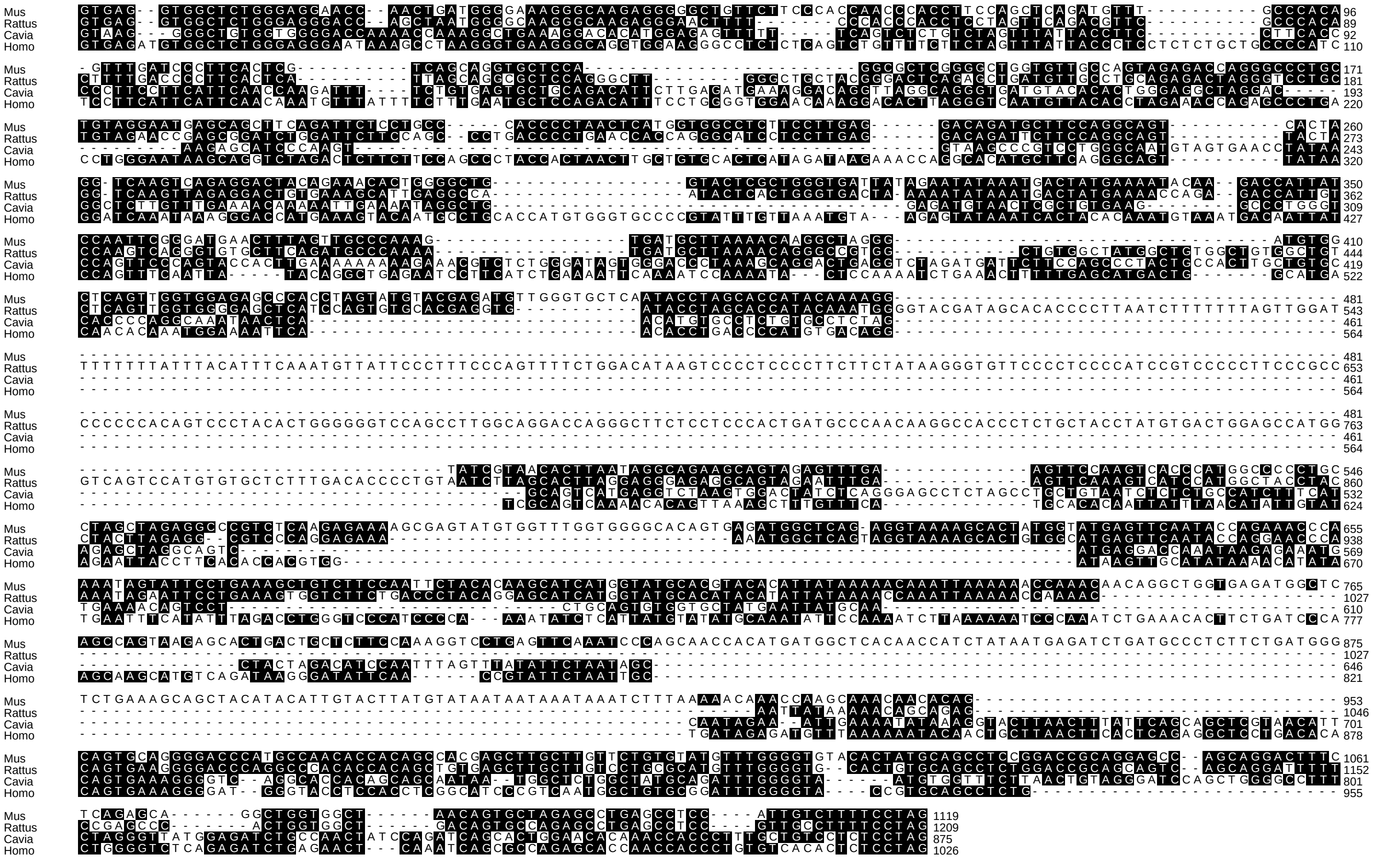

ENSMUSG00000037594 intron 4

Description: Uncharacterized protein C14orf79 homolog (BC022687)
Intron number: 4
Mouse chromosome: 12
Upstream exon length: 117
Downstream exon length: 162
Mouse intron length: 969
Intron alignment length: 1667
Total murinae branch length: 0.27715
K_score: 0.07873
Scaling factor: 0.62707

ENSMUSG00000037594 exon 4 (ORF 0)

```
Mus     AGCCCCCCTGGAGGCCAGGGTCATGTCCTGGAAGGTTCTGACTTGAGAAAACCTGAAGAGCTGCTGGCTGTCAGCGGTTT 80
Rattus  AGCCTTTCTGGAGACCAGGGTCATGATCTGGAAGGTTCTGACTGCAGAAAGCCTGAAGATCTGCTGGGAGTCAGTGGTTT 80
Cavia   GTCCCTTCTGGAAGCCAGGGCCACATCCTACAATGCCCCAGCCTCAGAGAGCCTGAGGAACTGCTGGCAGTCAGCAGCTT 80
Homo    AACCTTTCTGGAGGCCAGGGCCACATCATGGAAGATTGTGACCTCAAAGAGCCTGAAGGACTCCTCACTGTCAGCAGCTT 80

Mus     CCACCTGCACCACTGCAAAGCCCTGATTCAGACCAAG 117
Rattus  CCACCTACACCACTGCAAAGCCCTGATTCAGACCAAG 117
Cavia   CCATCTACACCCTTGCGAAGCCCTCATCCAGACCAAG 117
Homo    CTGTCTCCAGCATTGCAAAGCCCTGATCCAGACCAAG 117
```

ENSMUSG00000037594 exon 5 (ORF 0)

```
Mus     CTCTCAGGGACGTCCAGTAGCAGACAGGGCAGCCTGATTACCTACAGCCTGTTTCTGAAGACACCGCTCCAAGGGAACGG 80
Rattus  CTCTCAGGGACATCGGGCAGCAGACAGGGCAGCCTGATTACCTACAGTCTGTTTCTGAAGACACCACTCCAAGGGAATGG 80
Cavia   CCCTCAGGGACGCCAGGCAGCAGACAGGGGAGCCTGATTACCTACAGCCTCTTTCTGAAGTCGCCCCTTCATGGAAATGG 80
Homo    CTCTCGGGGCCGCCTGGCAGCAAACAGGGGAGGCTGATGACATGCAGCCGCTTCCTGAAGACCCCCTCATGCGGAGGTGG 80

Mus     GCGCTACATCACCATTCCAC---AAAAAAAGATCTTTACTCCACGCAACCTGAAAATGGCATTCTTTAATAATGATGTCT 157
Rattus  GCGCTACATCACCATCCCAC---AGAAGAAGATCTTTACTCCACGCAACCTGAAAATGGCATTCTTTAATAACAACGTCT 157
Cavia   ACAGTGTATTACCATCCCACAGAAAAAGAAGATTTTTGCTCCATGGAACCTAAAAGTGGCATTCTTTAATAATAATATTT 160
Homo    CCAGCACATCACTATTCCAA---GGAAAAGGATGTTCACTCCACGCAAGCTCAAACTGACACTCTTTAATAGCGACGTTT 157
```

ENSMUSG00000037594_intron_4

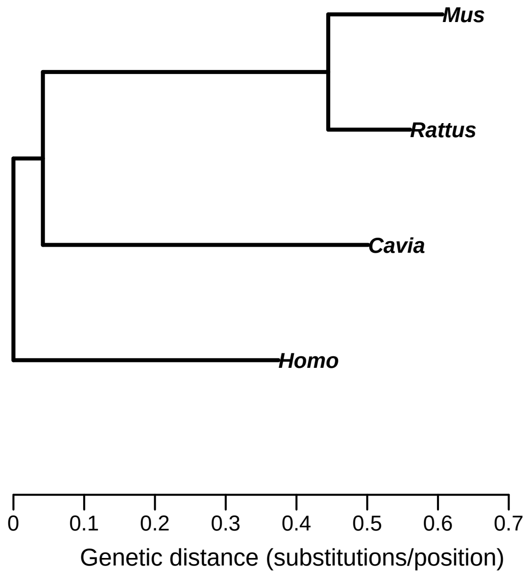

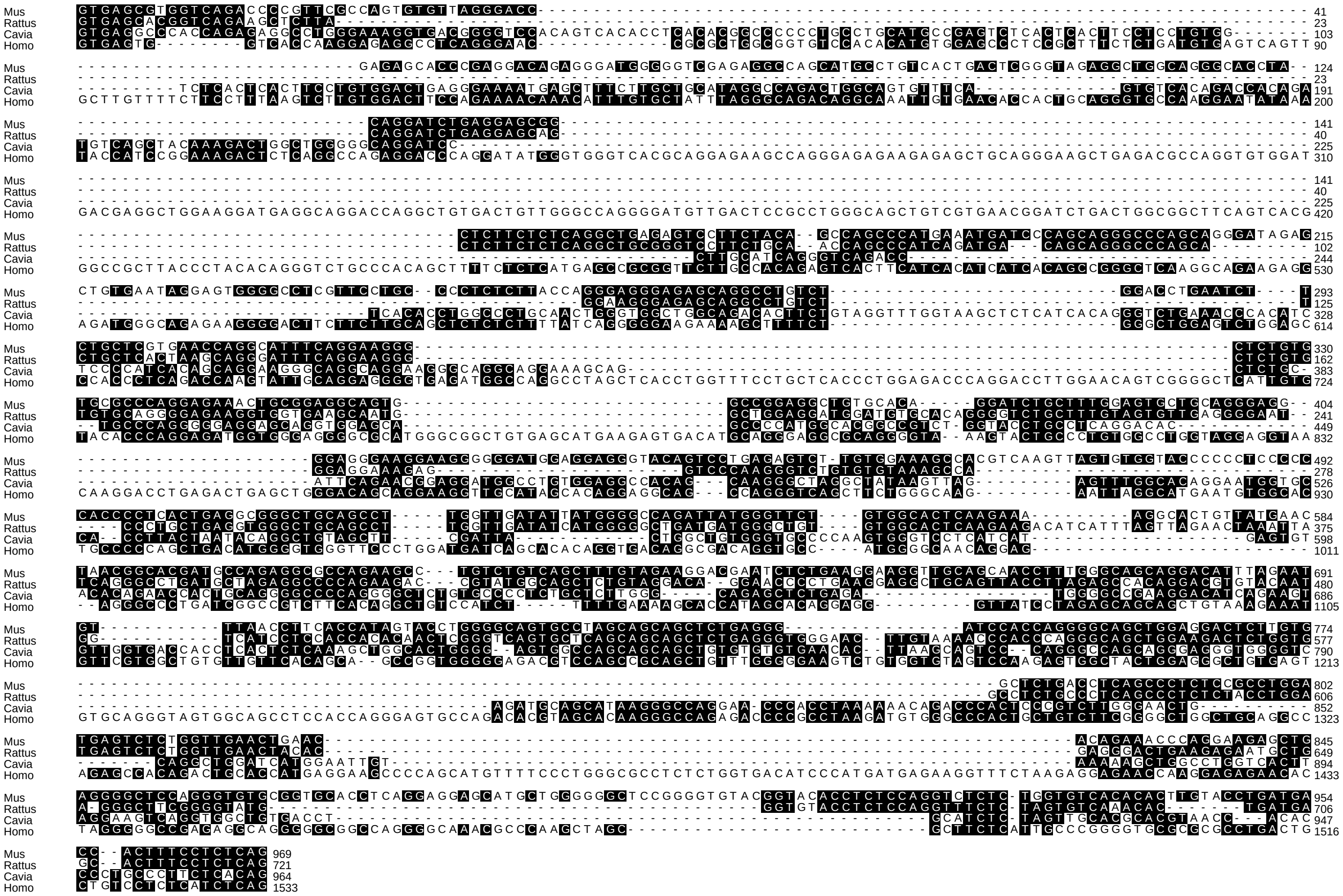
Mus
Rattus
Cavia
Homo

ENSMUSG00000040680 intron 2

Description: Kremen protein 2 Precursor (Kremen2)
Intron number: 2
Mouse chromosome: 17
Upstream exon length: 175
Downstream exon length: 92
Mouse intron length: 698
Intron alignment length: 1243
Total murinae branch length: 0.27735
K_score: 0.03133
Scaling factor: 0.62814

ENSMUSG00000040680 exon 2 (ORF 2)

```
Mus     TCCAGGTGAACGGCGCTGACTACCGAGGCCACCAGAACTACACCGGCCCACGCGGAGCTGGACGCCCTTGTCTTTTCTGG 80
Rattus  TCCAGGTGAACGGCGCTGACTACCGCGGCCACCAGAACTACACCGGCCCGCGCGGAGCTGGACGCCCTTGTCTTTTCTGG 80
Cavia   TCCAGGTGAATGGGGCCGACTATCGTGGCCACCAGAACCACACAGGCCCACGCGGGACAGGACGCCCGTGTCTCTTCTGG 80
Homo    TCCAGGTGAATGGGGCTGACTACCGCGGCCACCAGAACCGCACTGGCCCGCGCGGGGCGGGCCGCCCGTGCCTCTTCTGG 80

Mus     GACCAGACACAGCAGCACAGCTACAGCAGCGCCAGCGACCCTCAGGGCCGCTGGGGGTTGGGTGCGCATAACTTCTGTAG 160
Rattus  GACCAGACGCAGCAGCACAGCTACAGCAGCGCCAGCGATCCCCAGGGCCGCTGGGGGCTGGGTGCGCACAACTTCTGTAG 160
Cavia   GACCAGACACAGCAGCACAGCTACAGTAGCGCCAGTGACCCGCAGGGCCGCTGGGGGCTGGGCGCGCACAACTTCTGCCG 160
Homo    GACCAGACGCAGCAACACAGCTACAGCAGCGCCAGCGACCCCCACGGCCGCTGGGGGCTGGGCGCGCACAACTTCTGCCG 160
```

ENSMUSG00000040680 exon 3 (ORF 1)

```
Mus     GAACCCAGACGGTGATGTGCAGCCCTGGTGCTACGTGGCAGAGACAGAAGAGGGCATCTACTGGCGCTACTGTGATATCC 80
Rattus  GAACCCGGACGGCGATGTGCAGCCCTGGTGCTATGTGGCAGAGACAGAAGAAGGCATCTACTGGCGCTACTGTGATATCC 80
Cavia   GAACCCAGATGGTGATGTGCAACCATGGTGCTATGTGGCTGAGACGGAAGAGGGCATCTACTGGCGTTATTGTGACATCC 80
Homo    TAACCCAGACGGTGACGTGCAGCCGTGGTGCTACGTGGCTGAGACAGAGGAGGGCATCTACTGGCGCTACTGCGACATCC 80

Mus     CCACATGTCACA 92
Rattus  CCACGTGTCACA 92
Cavia   CCACATGTCACA 92
Homo    CCTCCTGTCACA 92
```

ENSMUSG00000040680_intron_2

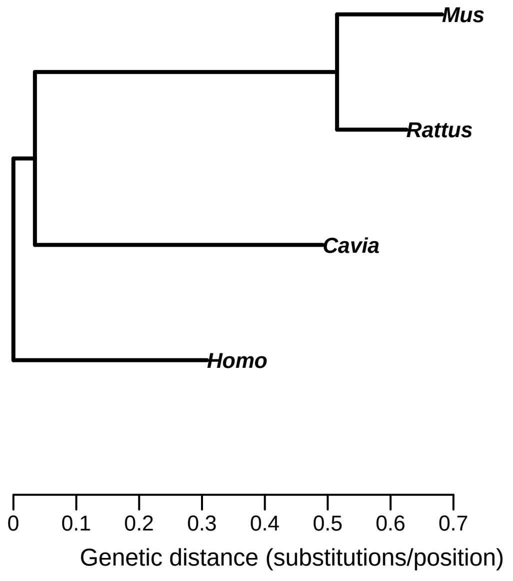

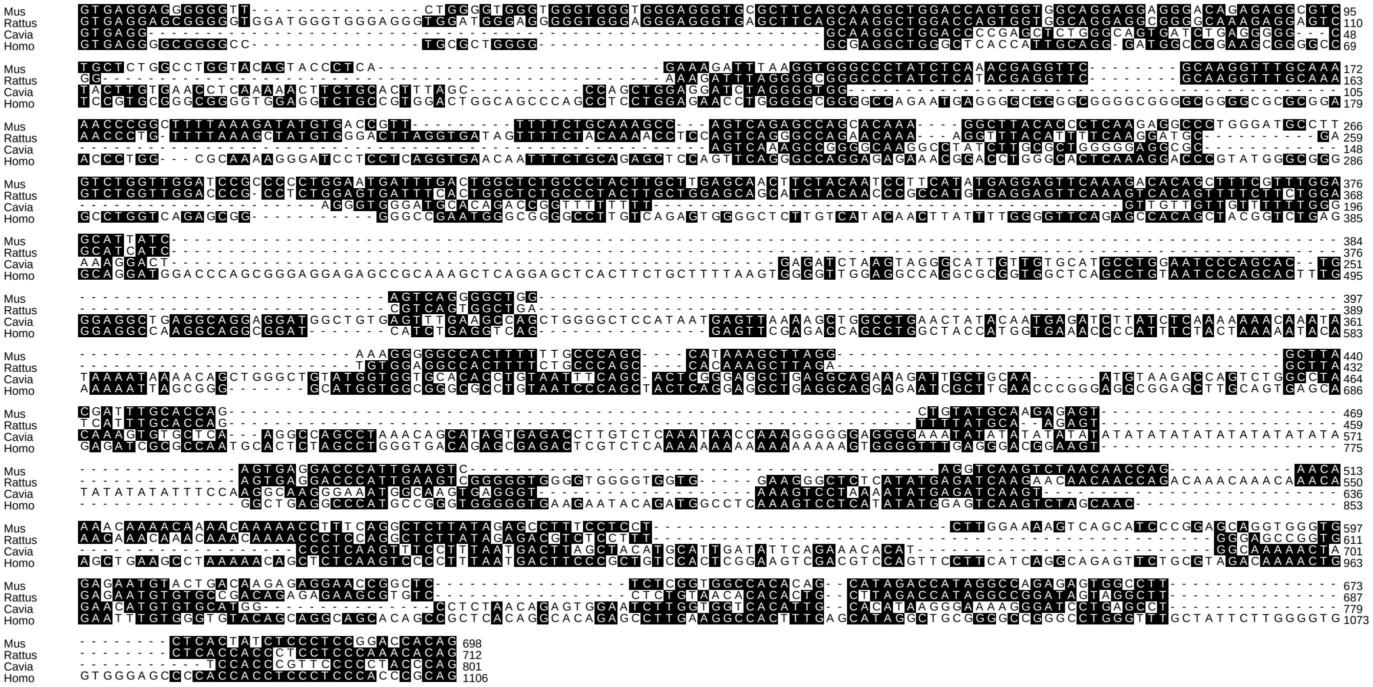

ENSMUSG00000027513 intron 1

Description: Phosphoenolpyruvate carboxykinase, cytosolic (Pck1)
Intron number: 1
Mouse chromosome: 2
Upstream exon length: 224
Downstream exon length: 182
Mouse intron length: 322
Intron alignment length: 443
Total murinae branch length: 0.21718
K_score: 0.04625
Scaling factor: 0.63141

ENSMUSG00000027513 exon 1 (ORF 0)

```
Mus     GCCTGCCCCAGGCAGTGAGGAAGTTCGTGGAAGGCAATGCTCAGCTGTGCCAGCCGGAGTATATCCACATCTGCGATGGC 80
Rattus  GCCTGCCCCAGGAAGTGAGGAAGTTTGTGGAAGGCAATGCCCAGCTGTGCCAGCCAGAGTATATTCACATCTGCGACGGC 80
Cavia   GCCTGCCTCGGGCAGTGAGGGAGTTCGTGGAGAGCAGCACGCAGCTGTGTGAGCCCGACCACATCCACATCTGCGATGGC 80
Homo    GCCTACCCCAGGCAGTGAGGGAGTTTCTCGAGAATAACGCTGAGCTGTGTCAGCCTGATCACATCCACATCTGTGACGGC 80

Mus     TCCGAGGAGGAGTACGGGCAGTTGCTGGCCCACATGCAGGAGGAGGGTGTCATCCGCAAGCTGAAGAAATATGACAACTG 160
Rattus  TCCGAGGAGGAGTACGGGCGGCTGCTGGCCCACATGCAGGAGGAGGGTGTCATCCGCAAGCTGAAGAAATATGACAACTG 160
Cavia   TCGGAGGCGGAGAACGGGCGGCTGCTGGCCCGCATGCAGGAGGACGGCGTCATCAGGAAGCTGGACAAGTATGATAATTG 160
Homo    TCTGAGGAGGAGAATGGGCGGCTTCTGGGCCAGATGGAGGAAGAGGGCATCCTCAGGCGGCTGAAGAAGTATGACAACTG 160
```

ENSMUSG00000027513 exon 2 (ORF 1)

```
Mus     TTGGCTGGCTCTCACTGACCCTCGAGATGTGGCCAGGATCGAAAGCAAGACAGTCATCATCACCCAAGAGCAGAGAGACA 80
Rattus  TTGGCTGGCTCTCACTGACCCCAGGGATGTGGCCAGGATCGAAAGCAAGACGGTCATCATTACCCAAGAGCAGAGAGACA 80
Cavia   TTGGTTGGCTCTCACTGACCCCAGGGATGTGGCCAGGATAGAAAGCAAGACCGTGATCATCACCCAGGAGCAAAGAGACA 80
Homo    CTGGTTGGCTCTCACTGACCCCAGGGATGTGGCCAGGATCGAAAGCAAGACGGTTATCGTCACCCAAGAGCAAAGAGACA 80

Mus     CAGTGCCCATCCCCAAAACTGGCCTCAGCCAGCTGGGCCGCTGGATGTCGGAAGAGGACTTTGAGAAAGCATTCAACGCC 160
Rattus  CCGTGCCCATCCCCAAAAGTGGGCAGAGCCAGCTGGGCCGCTGGATGTCAGAAGAGGACTTCGAGAAAGCATTCAACGCC 160
Cavia   CAGTACCCATCCCCAAAACCGGCCCCAGCCAGCTGGGTCGCTGGATGTCAGAAGAGGACTTTGAGAAAGCGTTCAATGCC 160
Homo    CAGTGCCCATCCCCAAAACAGGCCTCAGCCAGCTCGGTCGCTGGATGTCAGAGGAGGATTTTGAGAAAGCGTTCAATGCC 160
```

ENSMUSG00000027513_intron_1

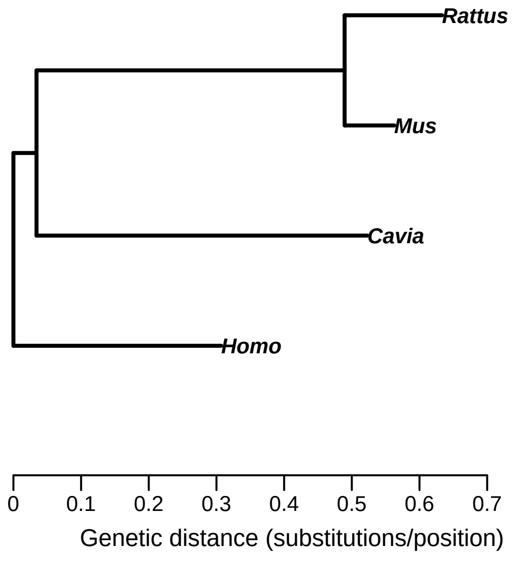

```
Mus     GTGAGTCCCCTCAGCCCTAACTCTCTGGCCCACAGCCTC----------CCTAACTCTATCTTCTTAGCGTT---------TGGAGAATGAAGACCTT---GCCGTAGCA 88
Rattus  GTGAGTCCCTTCAGCCCAAAGTCTCTGGCCTACAGGCTC----------CTCAGCTCTACCTTCTCGGAGTC---------TGGAGAATAAAGACCCTT--GCAGTAGCA 89
Cavia   GTGAGTTCC----------------------------------ACGGCGCCCGCACCCGGTTCCTGGGTGTTGGTGGA---GGGGTGATGGGGACACA-----------A 62
Homo    GTAAG----CTCGGCCCCCGCTGCCTGTCCCAGCACCCTGCAGGCAGGGCTCCCCTGCGTCTCCTGGGAGTTGGTGGAGAAAGGTGAATGAAGGCCTTCGGGTAGTTTCA 106

Mus     AACACCTGAGAAGCTTAAGAGAT-----------------------GCCCTGTGAGGTTAGCATAGCCAGCC-------------------------------------- 137
Rattus  AACGCCTGAGAAGCTTAGGAGATCAGTCAGAATCCAGAC-------ACCCTGTGAGGTTAGCATAGCCAGCT-------------------------------------- 154
Cavia   GCTGTTTGAAAACCACAAAG--------------------------ACGCCACAACGTTCACAGAGTTGCCCGTACTATCAACGGGATGCGCCTACAGAAACTCTTTGCA 146
Homo    GACTCTTGAGAAGATGAATGCAATGGTCAGAACCATACAGACTTGAATTTTGTGACATTAGTGGGCCAGCCCAAGCTTTAAATGAGGTGTGTGCACAAAAGCTC--TGCC 214

Mus     -ACTAGATTCTGGATAACTATACAAAGCAACTCCTTTCCCCAGGGCCAGGTCACAGTCTAGCTAATGTCCACCTGGTCAAGCTCCTGTTGTCCTTGTT-TC--------- 236
Rattus  -TCTAGATTCCAGATAGCCATGCAAGGCGGCCCCTTTCCCTAGGGCCAGCTCATGGCTTAGCAAATGTCTACCTGGTCAAGTTCCCGTTGGCTTGGCT-TCCATACCTAA 262
Cavia   AAGTTGATTCCTGATAAAAAGGAAGGGCAGTCCCTGTGCTCGGGGGCTGCTC-----GGGTTTGGTGACTCCCTGGTGCTGGCCCAGCTGGTTTGGCTATTTGCACTGTG 251
Homo    AACTAGATTCCTGATTA-AAAAAAAGGCAGCCCCTCTCCTACAGACCAGGTCCTAGTGGAGTAAATGTCCACCTGGCCATGTCTTAGATGGTCTGTGTGTTCACACTGAT 323

Mus     --------------------------CCGCAAGGCTT-GGAAGGACCTGGCGTGGGCAAGAGAGGTACATCCCCGAGGATACCTTGCTGAAGGAGTCCCTGTGACTTTTT 319
Rattus  GTAACACGGTCAACGTCCTTGTGT--CTGCAAGGCCG-GGAAGAGCCTGAGGTAGGCAAGAGAGGTACATCCCCAAGGGTACCTTGCTGAAGGCATCCCTGTGACTTTTT 369
Cavia   GCAGTGCCGC----------------CTGCCGGGCACAGGAAGGGCCTGATGACAAGGAGAGTGGTCCGTTCCTCATGACGCCCGGCTGAA--------TGTGGTTTTTG 337
Homo    GGACTGTTGTTAGCGTGCTCAGCACTCTGCTAGGCAT-GGAAAGCCACGGTACTGAAGGAGATGGTTCGCTGCCCGTGGTGCTTGGCTGAAAGGAAGCCTGTGATTTTTG 432

Mus     CAG 322
Rattus  CAG 372
Cavia   CAG 340
Homo    CAG 435
```

ENSMUSG00000021609 intron 12

Description: Sodium-dependent dopamine transporter (Slc6a3)
Intron number: 12
Mouse chromosome: 13
Upstream exon length: 168
Downstream exon length: 72
Mouse intron length: 1182
Intron alignment length: 2037
Total murinae branch length: 0.18049
K_score: 0.05343
Scaling factor: 0.63212

ENSMUSG00000021609 exon 12 (ORF 0)

```
Mus     CGTGGTCAGCATTGTGACCTTCAGACCCCCACACTATGGAGCCTACATCTTCCCAGACTGGGCCAATGCCCTGGGCTGGA 80
Rattus  CGTGGTCAGCATTGTGACCTTCAGACCCCCACACTATGGGGCCTACATCTTCCCAGACTGGGCCAATGCCCTGGGCTGGA 80
Cavia   TGTGGTCAGCGTTGTGACCTTCAGACCCCCCCACTACGGCTCCTACGTCTTCCCCGCCTGGGCCAACGCACTGGGCTGGG 80
Homo    CGTGGTCAGCATTGTGACCTTCAGACCCCCCCACTACGGAGCCTACATCTTCCCCGACTGGGCCAACGCGCTGGGCTGGG 80

Mus     TCATTGCCACATCCTCCATGGCCATGGTGCCCATTTATGCCACCTATAAGTTCTGCAGCCTGCCAGGGTCCTTCCGAGAG 160
Rattus  TCATCGCCACATCCTCCATGGCCATGGTGCCCATTTATGCGACCTACAAGTTCTGCAGCCTGCCGGGGTCCTTCCGGGAG 160
Cavia   CAGTTGCCGCCTCCTCCATGGCCATGGTGCCCATCTATGCGGCCTACAAGTTCTGCAGCCTCCCAGGCTCTTTCCGAGAG 160
Homo    TCATCGCCACATCCTCCATGGCCATGGTGCCCATCTATGCGGCCTACAAGTTCTGCAGCCTGCCTGGGTCCTTTCGAGAG 160
```

ENSMUSG00000021609 exon 13 (ORF 0)

```
Mus     AAACTGGCCTATGCCATCACACCTGAGAAAGACCGCCAGCTAGTGGACAGAGGGGAGGTGCGCCAATTCACG 72
Rattus  AAACTGGCCTATGCCATCACACCTGAGAAAGACCATCAGCTAGTGGACAGAGGGGAGGTGCGCCAATTCACG 72
Cavia   AAACTGGCCCAGGCCATCACACCCGAGAAGGACCGCGAGCTTGTGAACCGAGGAGAGGTGCGCCAGTTCACG 72
Homo    AAACTGGCCTACGCCATTGCACCCGAGAAGGACCGTGAGCTGGTGGACAGAGGGGAGGTGCGCCAGTTCACG 72
```

ENSMUSG00000021609_intron_12

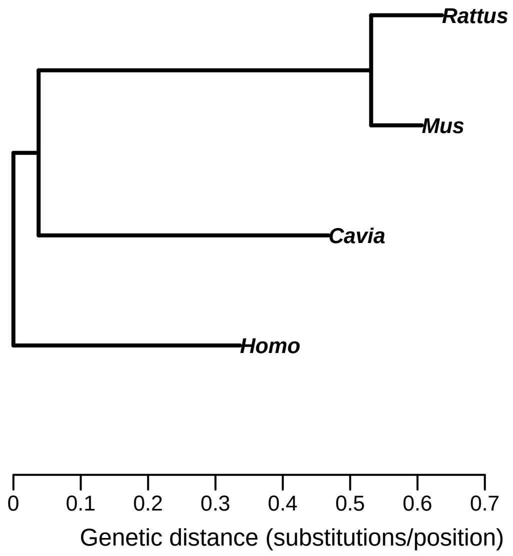

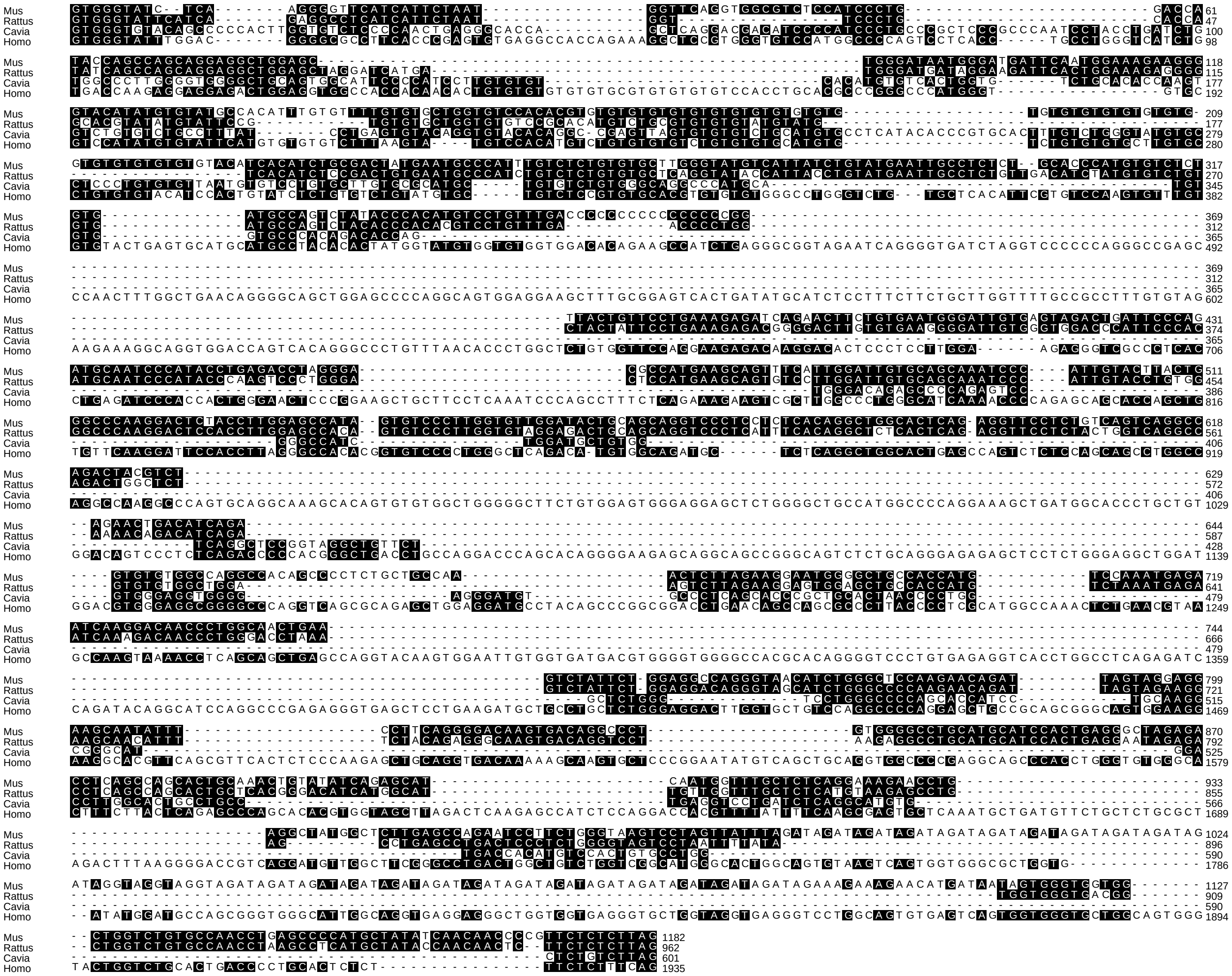
Mus
Rattus
Cavia
Homo

ENSMUSG00000031375 intron 3

Description: Biglycan Precursor (Bgn)
Intron number: 3
Mouse chromosome: X
Upstream exon length: 214
Downstream exon length: 111
Mouse intron length: 561
Intron alignment length: 1009
Total murinae branch length: 0.14172
K_score: 0.08245
Scaling factor: 0.63676

ENSMUSG00000031375 exon 3 (ORF 0)

```
Mus     CCTCTGCGGAAGCTGCAAAAACTCTACATCTCCAAGAACCACCTGGTGGAGATTCCTCCCAACCTGCCCAGCTCCCTGGT 80
Rattus  CCTCTGCGGAAGCTGCAGAAACTCTACATCTCCAAGAACCACCTGGTGGAGATTCCTCCCAACCTGCCCAGCTCCCTGGT 80
Cavia   CCGCTGCGGAAACTGCAGAAGCTGTACATCTCCAAGAACCACCTGGTAGAGATTCCGCCCAACCTTCCCAGCTCCCTGGT 80
Homo    CCACTGCGGAAGCTGCAGAAGCTCTACATCTCCAAGAACCACCTGGTGGAGATCCCGCCCAACCTACCCAGCTCCCTGGT 80

Mus     AGAACTACGAATCCATGACAACCGTATCCGCAAAGTGCCCAAGGGCGTGTTCAGCGGGCTCCGGAACATGAACTGCATTG 160
Rattus  AGAGCTACGAATCCATGACAACCGTATCCGCAAAGTGCCCAAGGGCGTGTTCAGTGGGCTCCGGAACATGAACTGCATTG 160
Cavia   GGAGCTGCGCATCCACGATAACCGGATCCGAAAAGTGCCTAAGGGCGTGTTCAGCGGGCTCCGGAACATGAACTGCATTG 160
Homo    GGAGCTCCGCATCCACGACAACCGCATCCGCAAGGTGCCCAAGGGAGTGTTCAGCGGGCTCCGGAACATGAACTGCATCG 160
```

ENSMUSG00000031375 exon 4 (ORF 2)

```
Mus     AGATGGGCGGGAATCCCCTGGAGAACAGTGGCTTTGAACCAGGAGCCTTTGATGGCCTGAAGCTCAATTACCTGCGCATC 80
Rattus  AGATGGGTGGGAATCCCCTGGAGAACAGTGGCTTTGAACCCGGAGCCTTTGATGGCCTGAAGCTCAACTACCTGCGCATC 80
Cavia   AGATGGGCGGGAACCCCCTGGAGAACAGCGGCTTTGAACCTGGAGCCTTCGATGGCCTGAAGCTCAACTACCTGCGCATC 80
Homo    AGATGGGCGGGAACCCACTGGAGAACAGTGGCTTTGAACCTGGAGCCTTCGATGGCCTGAAGCTCAACTACCTGCGCATC 80

Mus     TCAGAGGCCAAGCTCACTGGCATCCCCAAAG 111
Rattus  TCAGAGGCCAAGCTCACTGGCATCCCCAAAG 111
Cavia   TCCGAGGCCAAGCTCACTGGTATCCCCAAAG 111
Homo    TCAGAGGCCAAGCTGACTGGCATCCCCAAAG 111
```

ENSMUSG00000031375_intron_3

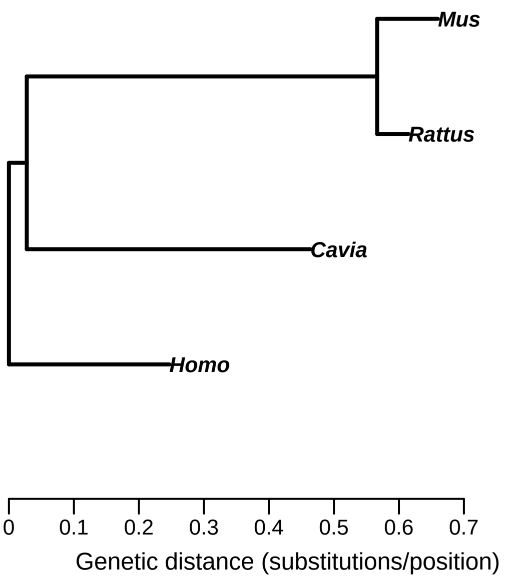

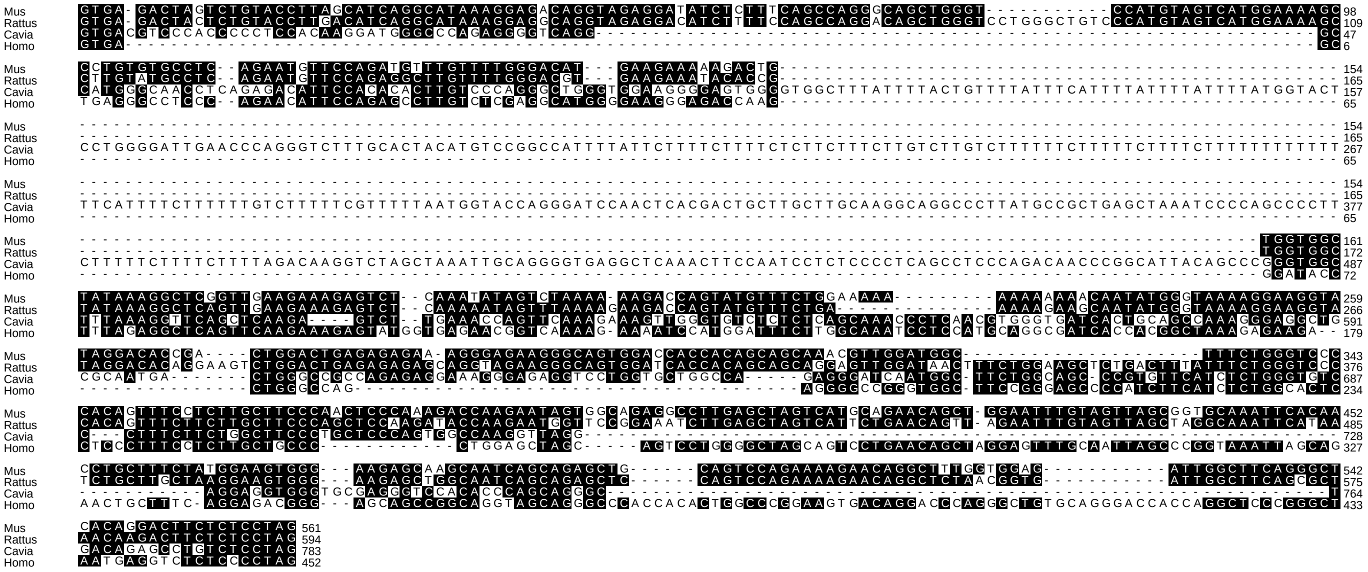
Mus
Rattus
Cavia
Homo
98
109
47
6
154
165
157
65
154
165
267
65
154
165
377
65
161
172
487
72
259
266
591
179
343
376
687
234
452
485
728
327
542
575
764
433
561
594
783
452

ENSMUSG00000041313 intron 10

Description: High affinity cationic amino acid transporter 1 (Slc7a1)
Intron number: 10
Mouse chromosome: 5
Upstream exon length: 109
Downstream exon length: 104
Mouse intron length: 1346
Intron alignment length: 1783
Total murinae branch length: 0.22903
K_score: 0.03872
Scaling factor: 0.6375

ENSMUSG00000041313 exon 10 (ORF 0)

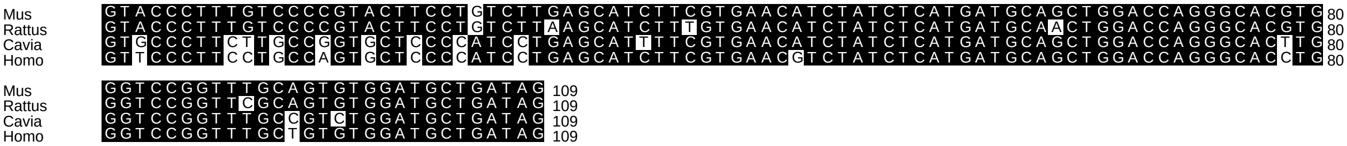

ENSMUSG00000041313 exon 11 (ORF 2)

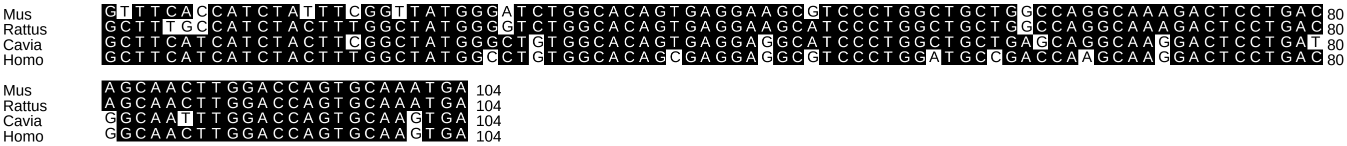

ENSMUSG00000041313_intron_10

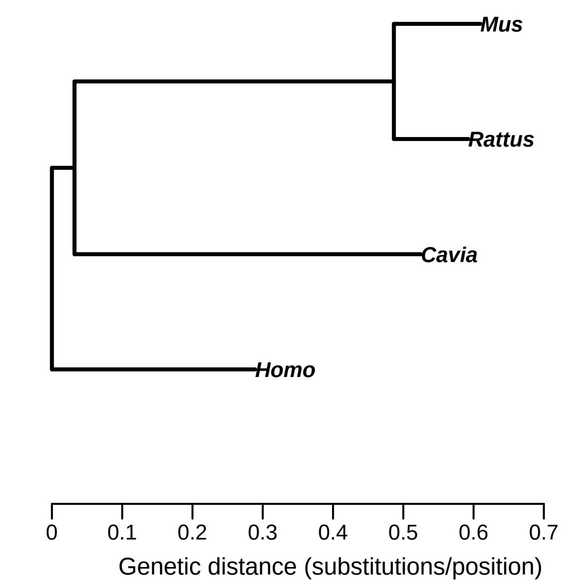

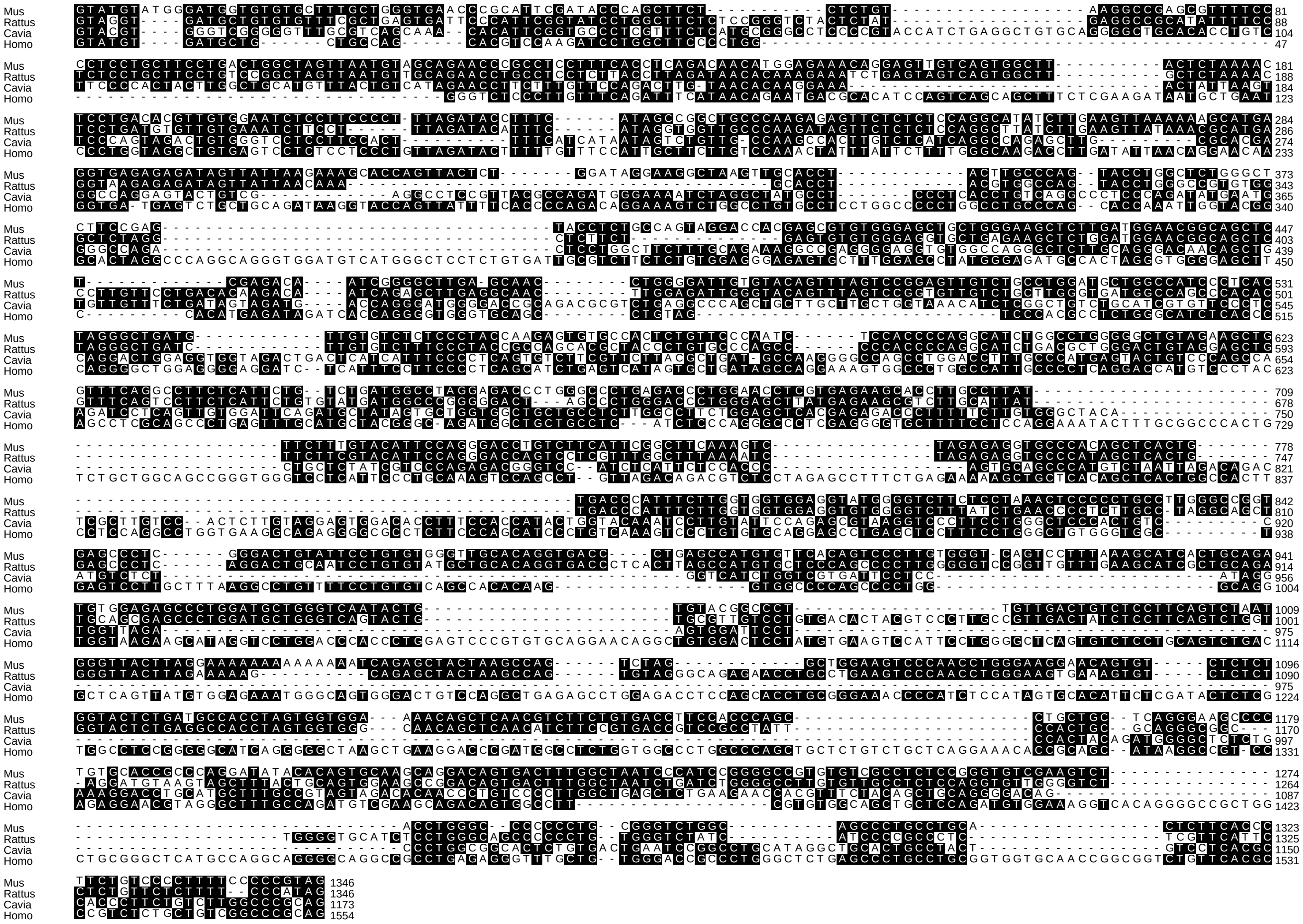

ENSMUSG00000020198 intron 22

Description: AP-3 complex subunit delta-1 (Ap3d1)
Intron number: 22
Mouse chromosome: 10
Upstream exon length: 72
Downstream exon length: 96
Mouse intron length: 388
Intron alignment length: 674
Total murinae branch length: 0.19484
K_score: 0.08845
Scaling factor: 0.63902

ENSMUSG00000020198 exon 22 (ORF 0)

```
Mus     GGTGAGGACTTAGACTTCTGGC--------------TGTCCACC-------------ACCCCACCACCTGCTGCTGCCCC 53
Rattus  GGTGAGGACTTAGACTTCTGGT--------------TGTCCACC-------------ACCCCGCCACCTGCTGCTGCCCC 53
Cavia   G-------------CCTCTGGCAGATAGCGAGAAGCTGCCCATCCAGAAACACAGAAACACCGAGACCTCCAA------- 60
Homo    GCTGAGGACCTGGACTTCTGGC--------------TGTCTACC-------------ACCCCACCGCCTGCCCCCGCCCC 53

Mus     CATTCCTGCCCC------GTCCACG 72
Rattus  CGTCCCTGCCCC------ATCTACG 72
Cavia   ------------------GTCCCCT 67
Homo    CGCCCCCGCCCCCGTTCCATCCACG 78
```

ENSMUSG00000020198 exon 23 (ORF 0)

```
Mus     GAAGAACTTGCTGCAAGCACTATCACCTCCCCTAAGGATGAGTGTGAGGTTCTCAAAGGGGAA------------GAGGA 68
Rattus  GAAGGACTTGCTGCGAGCATTGTCACCCCCCCTAAGGGTGAGTGTGAGGTTCTCAAAGGGGAAGAGCCCCAGGACGAGGA 80
Cavia   ---TCTACCACCCCGCCGCTTGTCACTGCCCCT----------------------------------------------- 30
Homo    ------------------------------------GACGAGTGTGAGGACGCCAAGACGGAGGCGCAGGGCGAGGAGGA 44

Mus     GGACCACGTGGATCATGACCAAGAAAGG 96
Rattus  GGACCACATGGATCATGACCAAGAAAGG 108
Cavia   ----------GCACCCGCCCCGTCCACG 48
Homo    CGATGCCGAGGGGCAAGACCAGGACAAG 72
```

ENSMUSG00000020198_intron_22

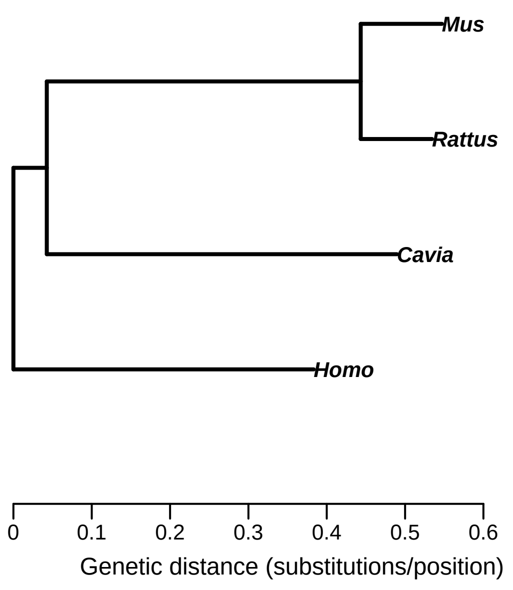

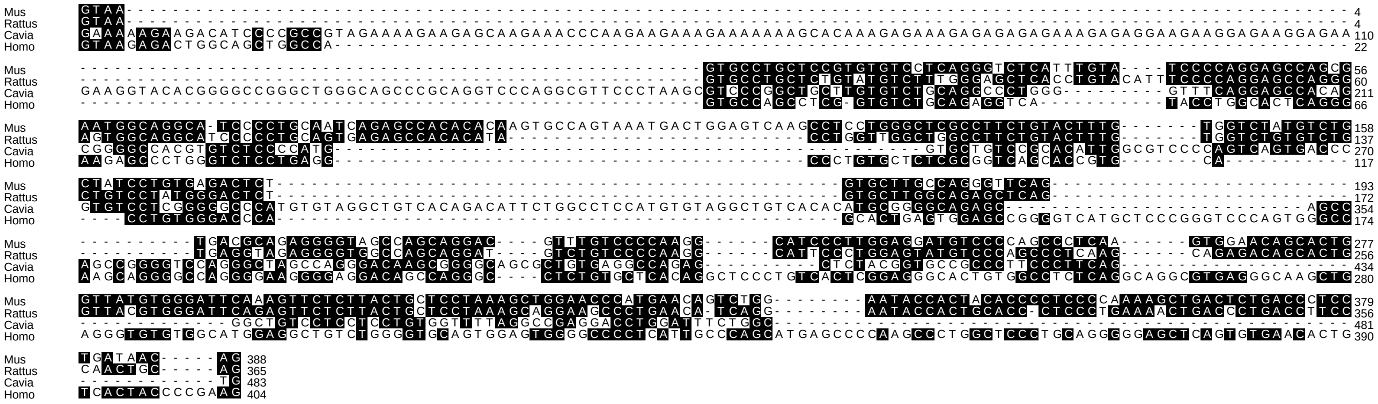

Mus
Rattus
Cavia
Homo
4
4
110
22
56
60
211
66
158
137
270
117
193
172
354
174
277
256
434
280
379
356
481
390
388
365
483
404

ENSMUSG00000038644 intron 18

Description: DNA polymerase delta catalytic subunit (Pold1)
Intron number: 18
Mouse chromosome: 7
Upstream exon length: 138
Downstream exon length: 176
Mouse intron length: 506
Intron alignment length: 999
Total murinae branch length: 0.24106
K_score: 0.04255
Scaling factor: 0.64265

ENSMUSG00000038644 exon 18 (ORF 0)

```
Mus     GTAGTCTACGGTGACACGGACTCTGTGATGTGCCGGTTTGGCGTCTCCTCTGTGGCTGAAGCAATGTCTCTGGGGCGGGA 80
Rattus  GTGGTCTACGGTGACACTGACTCTGTGATGTGCCGATTTGGTGTCTCCTCTGTGGCTGAGGCAATGTCTCTGGGGCGGGA 80
Cavia   GTGGTGTACGGTGACACTGACTCTGTCATGTGCCGGTTCGGCGTCTCCTCCGTGGCTGAGGCAATGGACCTGGGGCGTGA 80
Homo    GTGGTGTATGGTGACACTGACTCCGTCATGTGCCGATTCGGCGTGTCCTCGGTGGCTGAGGCGATGGCCCTGGGGCGGGA 80

Mus     GGCTGCAAACTGGGTATCCAGTCACTTCCCATCACCCATCCGGCTGGAGTTCGAGAAG 138
Rattus  GGCTGCAAACTGGGTATCCAGTCACTTCCCATCACCCATCCGGCTGGAGTTCGAGAAG 138
Cavia   GGCCGCGAGCTGGGTGTCCAGCCACTTCCCCCCGCCCATCCGGCTGGAGTTCGAGAAG 138
Homo    GGCCGCGGACTGGGTGTCAGGTCACTTCCCGTCGCCCATCCGGCTGGAGTTTGAGAAG 138
```

ENSMUSG00000038644 exon 19 (ORF 0)

```
Mus     GTTTACTTCCCATACCTGCTCATCAGCAAGAAGCGCTATGCTGGCCTGCTCTTCTCCTCCCGCTCTGATGCCCATGACAA 80
Rattus  GTTTACTTTCCCTACCTGCTCATCAGCAAGAAGCGCTATGCCGGCCTACTCTTCTCCTCCCGCTCTGATGCCCATGACAG 80
Cavia   GTCTACTTCCCCTACCTGCTCATCAGCAAGAAGCGCTATGCCGGCCTGCTCTTCTCCTCCCGGCCCGACAGCCACGACCG 80
Homo    GTCTACTTCCCATACCTGCTTATCAGCAAGAAGCGCTACGCGGGCCTGCTCTTCTCCTCCCGGCCCGACGCCCACGACCG 80

Mus     AATGGACTGCAAGGGCCTGGAGGCTGTGCGCAGGGACAACTGTCCCCTGGTGGCCAACCTCGTTACATCCTCTCTGCGCC 160
Rattus  AATGGACTGCAAGGGCCTGGAGGCTGTGCGTAGGGACAACTGTCCCCTGGTGGCCAACCTTGTCACATCCTCTCTGCGCA 160
Cavia   CATGGACTGCAAGGGCCTGGAGGCTGTGCGCAGGGACAACTGCCCGCTCGTGGCCAACCTGGTCACAGCCTCATTGCGTC 160
Homo    CATGGACTGCAAGGGCCTGGAGGCCGTGCGCAGGGACAACTGCCCCCTCGTGGCCAACCTGGTCACTGCCTCACTGCGCC 160
```

ENSMUSG00000038644_intron_18

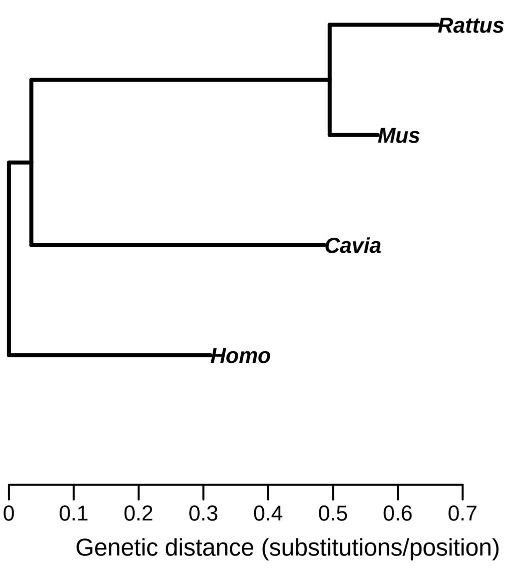

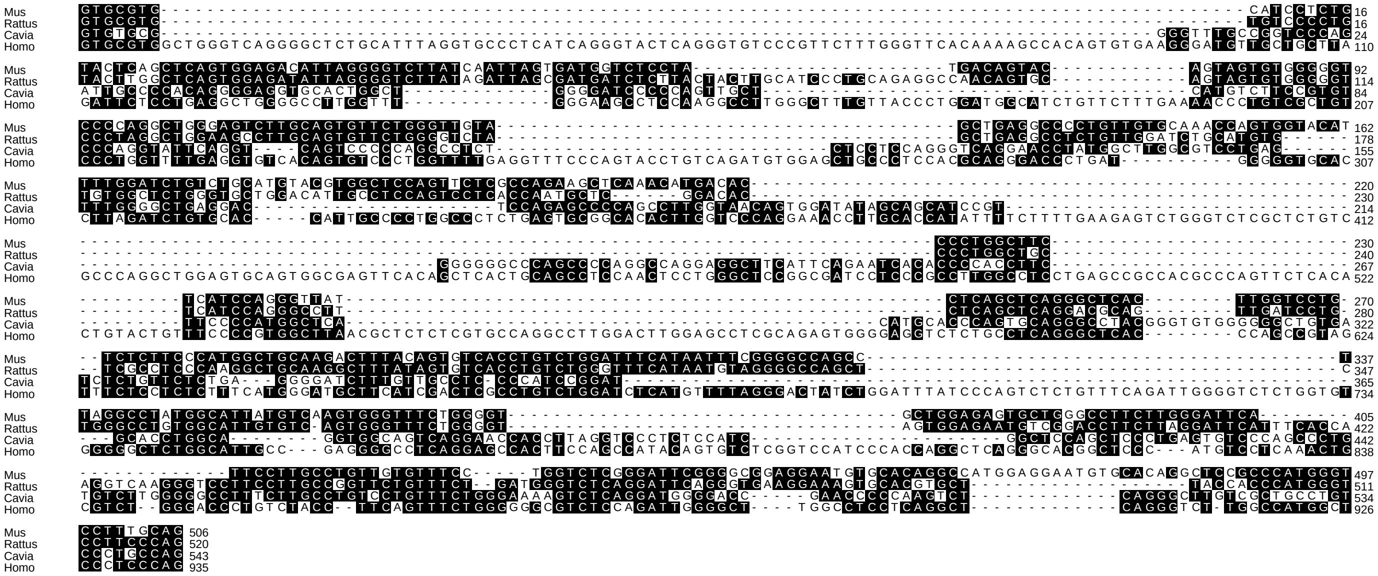

ENSMUSG00000035051 intron 19

Description: Putative ATP-dependent RNA helicase DHX57 (Dhx57)
Intron number: 19
Mouse chromosome: 17
Upstream exon length: 135
Downstream exon length: 75
Mouse intron length: 1087
Intron alignment length: 2221
Total murinae branch length: 0.29213
K_score: 0.06477
Scaling factor: 0.6434

ENSMUSG00000035051 exon 19 (ORF 0)

```
Mus     GAAATGGCCAGCCTCAAACGACAATTCACTGAACTGCTGTCCGACATAGGGTTTGTGAAAGAAGGACTCCGAGCAAAAGA 80
Rattus  GAAATGGCCAGCCTCAAACGGCAGTTCACTGAGCTGCTTTCCGATATAGGGTTTGTCAAAGAGGGACTCAGAGCAAAAGA 80
Cavia   GAAATGGCCAGCCTCAAACGACAATTCACTGAACTGTTATCAGATATAGGGTTTGTAAGGGAGGGACTCAGAGCAAGAGA 80
Homo    GAAATGGCCAGCCTCAAACGACAATTCACGGAACTGTTATCGGATATAGGGTTTGCAAGGGAAGGGCTCAGAGCAAGGGA 80

Mus     AATTGAAAAACGAGCCCAGGGAGGAGATGGTGTCCTGGATGCCACAGGAGAGGAG 135
Rattus  GATTGAAAAGAGAGCCCAAGGGGGAGATGGTGTCCTGGATGCCACAGGAGAGGAG 135
Cavia   AATTGAGAAAAGGGCCCAAGGAGGAGATGGTGTCCTGGATGCCACAGGAGAGGAG 135
Homo    AATTGAGAAAAGGGCCCAAGGAGGAGATGGTGTCTTAGATGCCACAGGAGAAGAG 135
```

ENSMUSG00000035051 exon 20 (ORF 0)

```
Mus     GCTAACACAAATGCTGAGAACCCCAAGCTGATATCAGCAGTGCTGTGTGCCGCTCTGTATCCAAACGTAGTACAG 75
Rattus  GCTAACACAAATGCTGAGAACCCCAAGCTGATATCAGCAGTGCTGTGTGCCGCTCTGTATCCAAATGTAGTACAG 75
Cavia   GCAAACTCTAATGCAGAGAACCCCAAGCTGATATCAGCCGTGCTGTGTGCTGCTCTGTATCCAAATGTAGTGCAG 75
Homo    GCAAACTCAAATGCTGAGAACCCCAAGCTGATATCAGCAATGCTGTGTGCTGCTTTGTATCCAAATGTAGTGCAG 75
```

ENSMUSG00000035051_intron_19

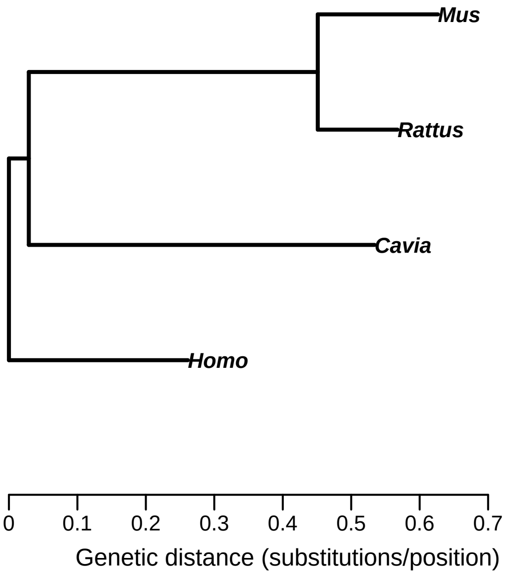

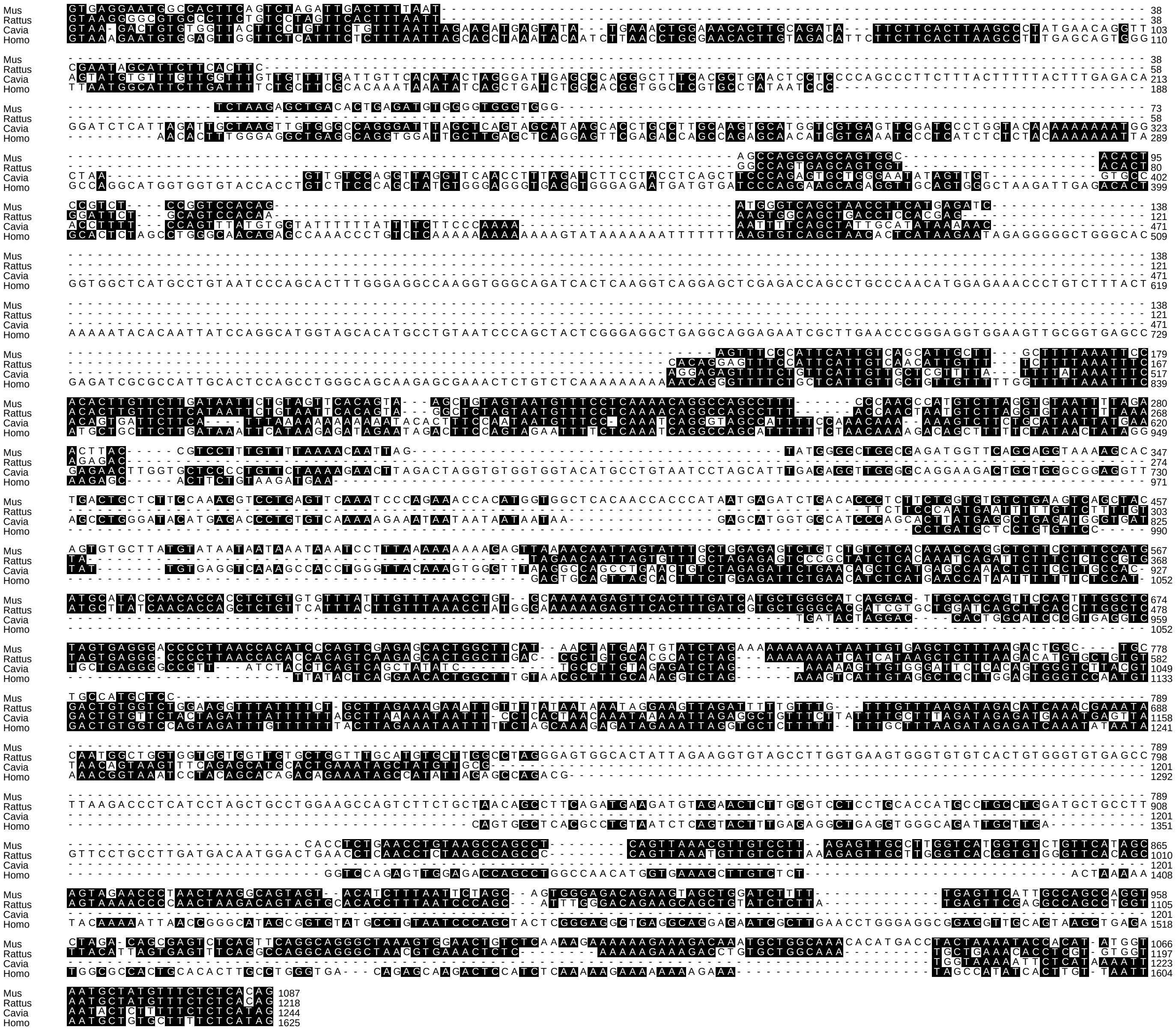

ENSMUSG00000026272 intron 10

Description: Serine--pyruvate aminotransferase, mitochondrial Precursor (Agxt)
Intron number: 10
Mouse chromosome: 1
Upstream exon length: 129
Downstream exon length: 108
Mouse intron length: 420
Intron alignment length: 721
Total murinae branch length: 0.15391
K_score: 0.0732
Scaling factor: 0.64431

ENSMUSG00000026272 exon 10 (ORF 0)

```
Mus     GAAATCCGGCTACCCACAATCACCACCGTGACTGTGCCTGCCGGCTACAACTGGAGGGACATCGTCAGCTATGTGCTGGA 80
Rattus  GAAATCCGGCTACCTACAATCACCACCGTGACCGTGCCTGCCGGCTACAACTGGAGGGACATCGTCAGCTACGTGCTGGA 80
Cavia   GCCAGCCGTCTACCTACAGTCACCACCGTGGCTGTGCCCGCTGGCTACAACTGGAGGGACATCGTGGACTACGTCATGGA 80
Homo    GCGCTCCGGCTTCCCACAGTCACCACTGTGGCTGTACCCGCTGGCTATGACTGGAGAGACATCGTCAGCTACGTCATAGA 80

Mus     CCACTTCAGCATTGAAATCTCTGGGGGTCTTGGGCCCACTGAGGAGAGG 129
Rattus  CCACTTCAACATTGAAATCTCTGGTGGTCTTGGGCCCTCTGAGGATAAG 129
Cavia   CTCCTTCAGCATTGAGATCTCGGGTGGGCTTGGACCTTCCCTGGGGAAG 129
Homo    CCACTTCGACATTGAGATCATGGGTGGCCTTGGGCCCTCCACGGGGAAG 129
```

ENSMUSG00000026272 exon 11 (ORF 0)

```
Mus     GTGCTACGGATTGGCCTCCTGGGCTACAACGCCACCACTGAGAATGTGGACCGTGTGGCCGAGGCCCTGAGGGAGGCCCT 80
Rattus  GTGCTGCGGATTGGCCTCCTGGGCTACAACGCCACCACAGAGAATGCGGACCGTGTAGCGGAGGCCCTGAGGGAGGCCCT 80
Cavia   GTGCTGCGGATCGGCCTGATGGGCTGTAATGCCAGCCGGGAGAACGTGGACCGCCTGGCCCAGGCCCTGAAGGAGGCCCT 80
Homo    GTGCTGCGGATCGGCCTGCTGGGCTGCAATGCCACCCGCGAGAATGTGGACCGCGTGACGGAGGCCCTGAGGGCGGCCCT 80

Mus     GCAGCATTGTCCTAAGAACAAGTTGTGA 108
Rattus  GCAACATTGTCCTAAGAATAAATTGTGA 108
Cavia   GCAGCGCTGCCCCCGTCACAGGCTGTAA 108
Homo    GCAGCACTGCCCCAAGAAGAAGCTGTGA 108
```

ENSMUSG00000026272_intron_10

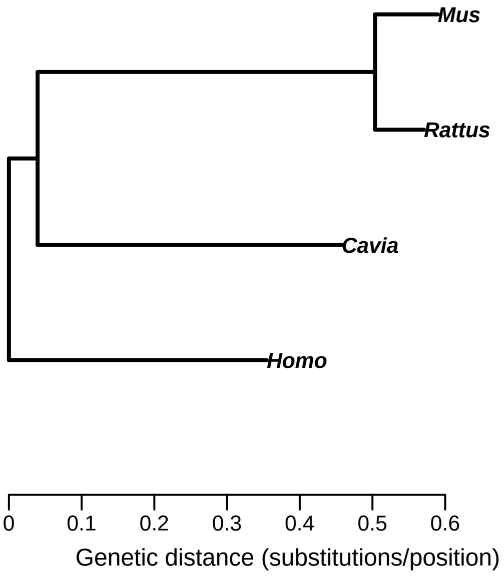

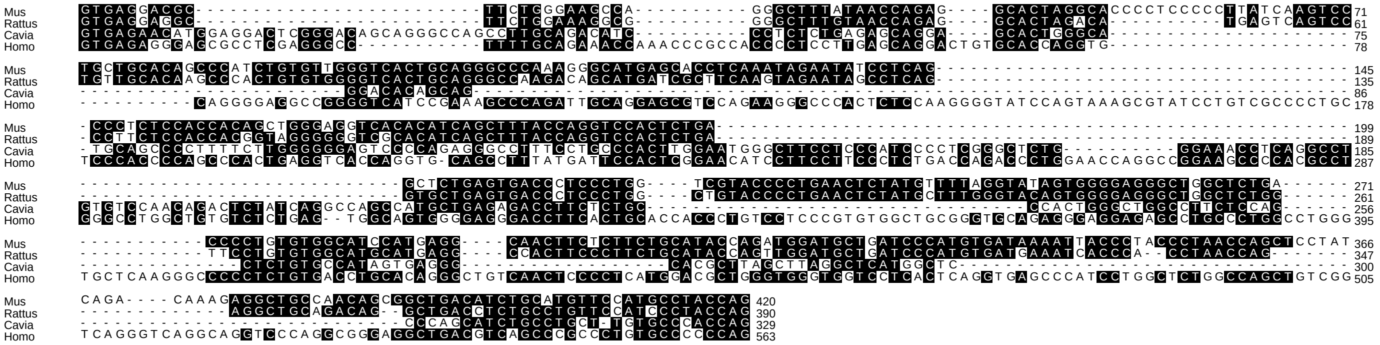
Mus
Rattus
Cavia
Homo

ENSMUSG00000014453 intron 4

Description: Tyrosine-protein kinase BLK (Blk)
Intron number: 4
Mouse chromosome: 14
Upstream exon length: 99
Downstream exon length: 104
Mouse intron length: 1189
Intron alignment length: 1370
Total murinae branch length: 0.19167
K_score: 0.05927
Scaling factor: 0.64588

ENSMUSG00000014453 exon 4 (ORF 1)

```
Mus     CACTGGAGACTGGTGGTTGGCCAGGTCACTCGTCACAGGAAGAGAAGGTTATGTGCCCAGCAACTTTGTGGCCCCAGTAG 80
Rattus  CACTGGAGACTGGTGGTTGGCCAGGTCACTCGTCACAGGAAGAGAAGGCTATGTGCCCAGCAACTTTGTGGCCCCAGTAG 80
Cavia   GACTGGGGACTGGTGGTTGGCCAAATCACTCATCACCGGAAGAGAGGGCTACGTGCCCAGCAACTTCGTGGCCCCTGTGG 80
Homo    AACTGGAGACTGGTGGCTGGCCAGGTCACTCGTCACAGGAAGAGAAGGCTATGTGCCCAGTAACTTTGTGGCCCGAGTGG 80

Mus     AGACTCTGGAAGTAGAAAA 99
Rattus  AGACCCTGGAAGTAGAAAA 99
Cavia   AGACCCTGGAAGTGGAAAA 99
Homo    AGAGCCTGGAAATGGAAAG 99
```

ENSMUSG00000014453 exon 5 (ORF 1)

```
Mus     ATGGTTCTTCAGGACCATCAGCCGGAAGGATGCTGAGAGGCAGTTGCTGGCTCCGATGAACAAGGCCGGCTCCTTTCTCA 80
Rattus  ATGGTTCTTCAGGACCATTAGCCGGAAGGATGCTGAGAGGCAGTTGCTGGCTCCAATGAACAAAGCCGGCTCCTTTCTCA 80
Cavia   GTGGTTCTTCAGGTCCATCAGCCGGAAGGAGGCCGAACGGCAGCTGCTGGCCCCGCTGAACAAGGTCGGCTCCTTCCTCA 80
Homo    GTGGTTCTTTAGATCACAGGGTCGGAAGGAGGCTGAGAGGCAGCTTCTTGCTCCAATCAACAAGGCCGGCTCCTTTCTTA 80

Mus     TCAGAGAGAGTGAGAGCAATAAAG 104
Rattus  TCAGAGAGAGTGAGAGCAATAAAG 104
Cavia   TCAGAGAAAGTGAGACCACCAAAG 104
Homo    TCAGAGAGAGTGAAACCAACAAAG 104
```

ENSMUSG00000014453_intron_4

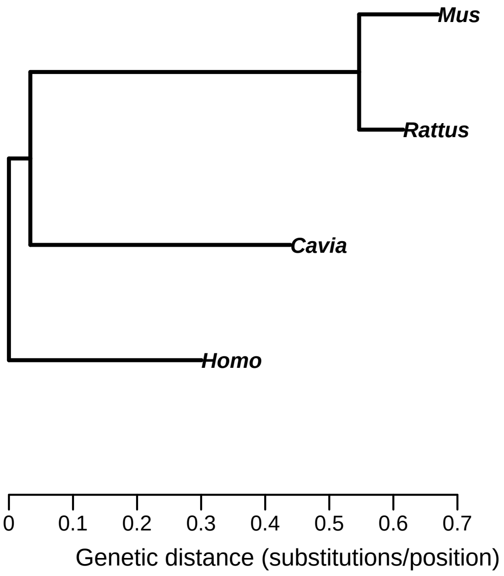

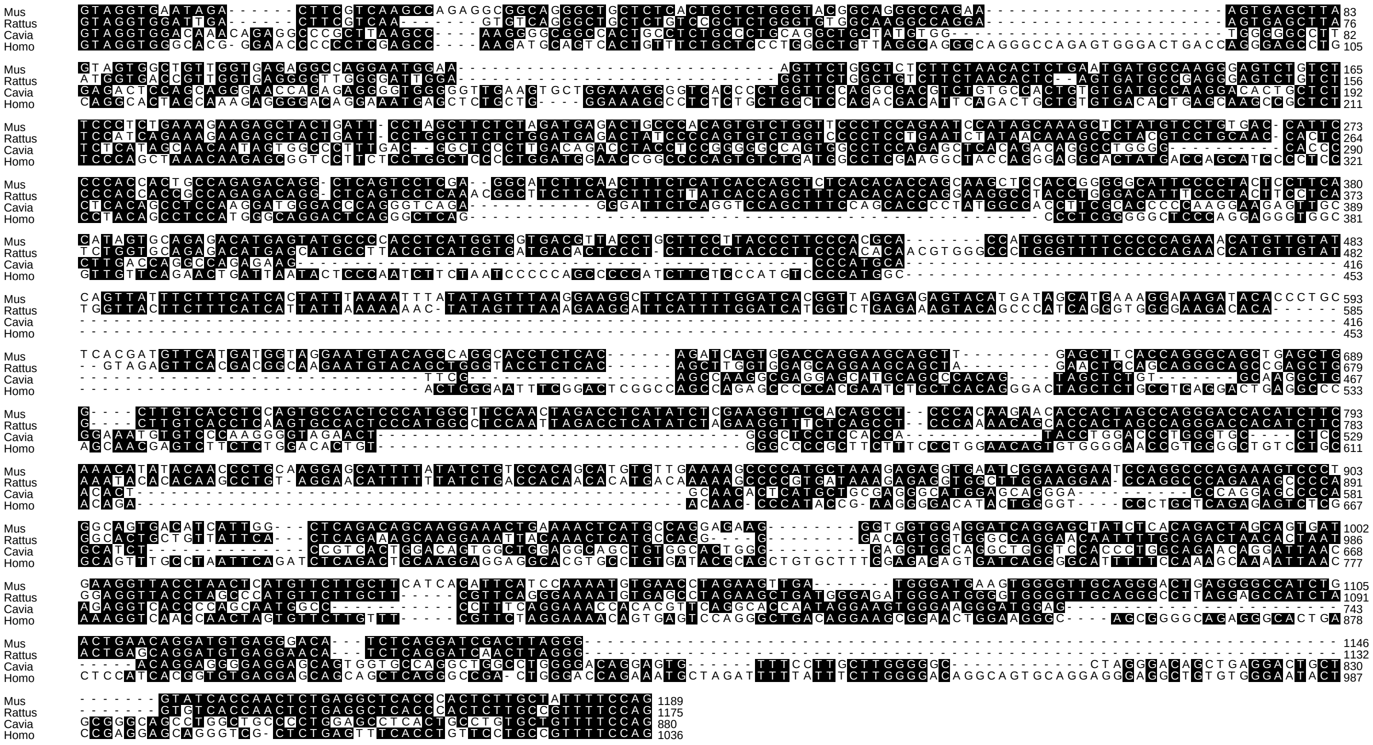
Mus
Rattus
Cavia
Homo

ENSMUSG00000034634 intron 1

Description: Lymphocyte antigen 6D Precursor (Ly6d)
Intron number: 1
Mouse chromosome: 15
Upstream exon length: 52
Downstream exon length: 99
Mouse intron length: 659
Intron alignment length: 882
Total murinae branch length: 0.18082
K_score: 0.08033
Scaling factor: 0.65137

ENSMUSG00000034634 exon 1 (ORF 0)

```
Mus     ATGAAGACAGCTCTGCTCGTCCTCCTTGTCTTGGCTGTGGCCACTAGCCCAG 52
Rattus  ATGAAGACAGCTCTGCTCCTCCTTTTGGCCTTGGCTGTGGCCACTAGCCCAG 52
Cavia   ATGAAGACAGTCCTGCTGCTCCTTATTGCCCTGACTGTGGCCCTGAGCCCAG 52
Homo    ATGAGGACAGCATTGCTGCTCCTTGCAGCCCTGGCTGTGGCTACAGGGCCAG 52
```

ENSMUSG00000034634 exon 2 (ORF 2)

```
Mus     CCTGGGCACTTCGATGTCACGTGTGCACCAACAGTGCCAACTGTAAGAACCCTCAGGTCTGCCCGTCCAACTTCTACTTC 80
Rattus  CCCAGGCATTCCGATGTCATGTGTGCATCAGCAGTGACAACTGTAGGAATATTCAGACCTGTCCACCCGACTTCTTCTAC 80
Cavia   CCTACACACTCCGCTGTCATGTGTGCAGAGACACTGGCAACTGCAAGAATCCCCAGAACTGCCCTAGCAGCTCCCGCTAC 80
Homo    CCCTTACCCTGCGCTGCCACGTGTGCACCAGCTCCAGCAACTGCAAGCATTCTGTGGTCTGCCCGGCCAGCTCTCGCTTC 80

Mus     TGCAAAACCGTCACCTCAG 99
Rattus  TGCAGAACAATCACCAAAG 99
Cavia   TGCAAGACTGTGATTTCAG 99
Homo    TGCAAGACCACGAACACAG 99
```

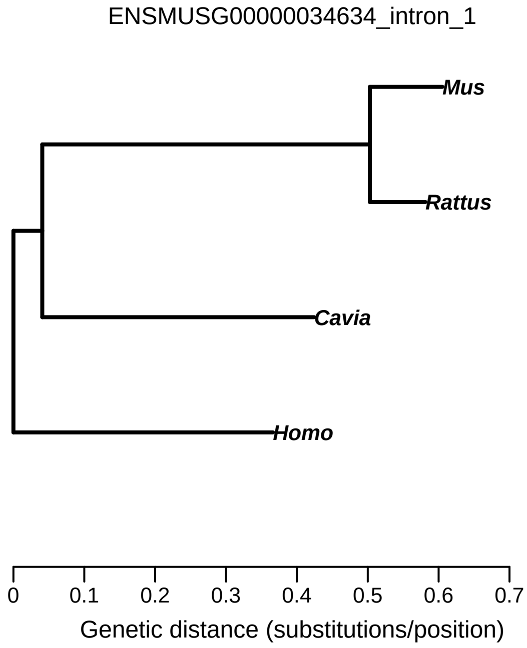

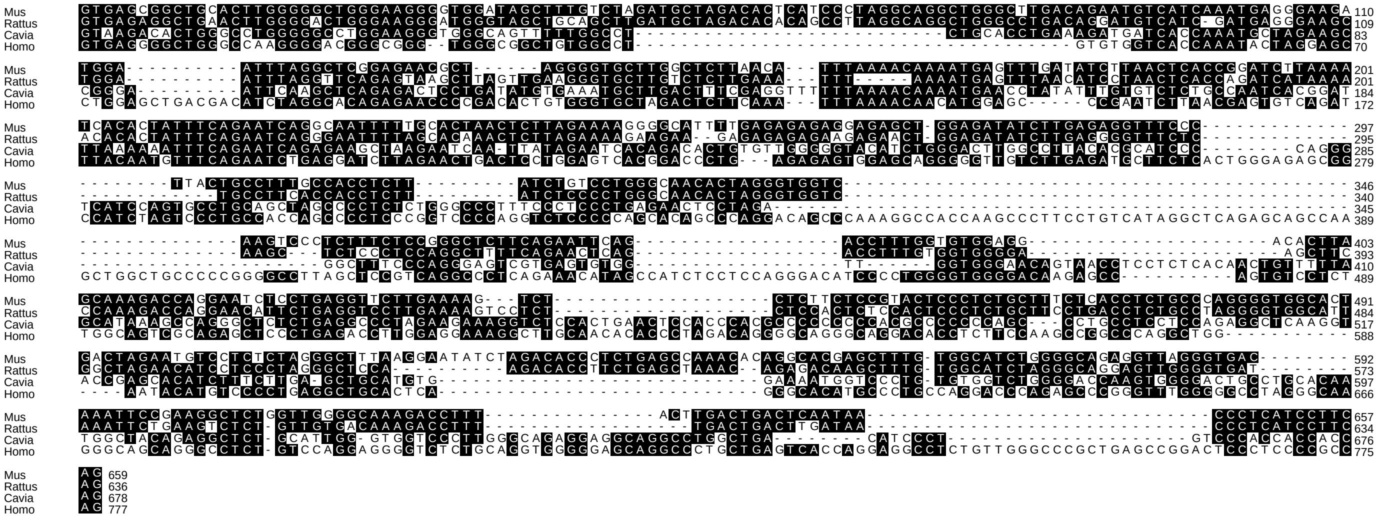
Mus
Rattus
Cavia
Homo

ENSMUSG00000038567 intron 5

Description: 1,25-dihydroxyvitamin D (Cyp24a1)
Intron number: 5
Mouse chromosome: 2
Upstream exon length: 92
Downstream exon length: 112
Mouse intron length: 933
Intron alignment length: 1460
Total murinae branch length: 0.19854
K_score: 0.07871
Scaling factor: 0.65165

ENSMUSG00000038567 exon 5 (ORF 2)

```
Mus     GCATCTGCCTTGTGTTATATGAGAAGAGATTCGGGCTCCTTCAAAAGGACACAGAGGAAGAAGCCCTGACCTTCATTGCG 80
Rattus  GCATCTGCCTTGTGTTATATGAGAAGAGATTTGGGCTCCTTCAAAAGGAGACAGAAGAAGAAGCGCTGACCTTCATTACG 80
Cavia   GTATCTGCCTCATACTGTACGAGAAGAGATTCGGGCTCCTTCAGAAGAATGTGAGCGAGGAATCCCTGAACTTCATCACA 80
Homo    GTATCTGCCTCGTGTTGTATGAGAAGAGATTTGGGCTTCTCCAGAAGAATGCAGGGGATGAAGCTGTGAACTTCATCATG 80

Mus     GCCATCAAAACA 92
Rattus  GCCATCAAAACG 92
Cavia   GCCATCAAAACA 92
Homo    GCCATCAAAACA 92
```

ENSMUSG00000038567 exon 6 (ORF 0)

```
Mus     ATGATGAGCACATTTGGGAAGATGATGGTGACCCCCGTGGAGCTGCACAAGCGCCTCAACACCAAAGTGTGGCAAGCGCA 80
Rattus  ATGATGAGCACGTTTGGGAAGATGATGGTGACCCCCGTGGAGTTGCACAAGCGCCTCAACACCAAAGTGTGGCAGGCACA 80
Cavia   ATGATGGGTACCTTTGGGAAGATGATGGTCACCCCGGTCGAGCTGCACAAGAGCCTCAACACCAGAGTCTGGCAGGCGCA 80
Homo    ATGATGAGCACGTTTGGGAGGATGATGGTCACTCCAGTCGAGCTGCACAAGAGCCTCAACACCAAGGTCTGGCAGGACCA 80

Mus     CACGCTGGCCTGGGACACCATTTTCAAATCAG 112
Rattus  TACGCTGGCTTGGGACACCATTTTCAAATCAG 112
Cavia   CACCCTGGCCTGGGACACCATCTTCAACTCAG 112
Homo    CACTCTGGCCTGGGACACCATTTTCAAATCAG 112
```

ENSMUSG00000038567_intron_5

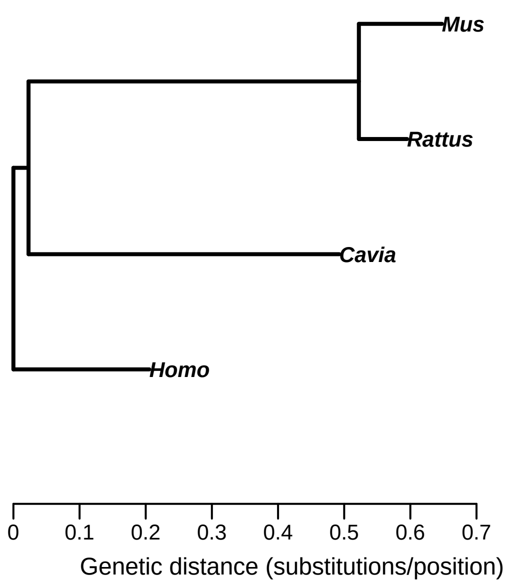

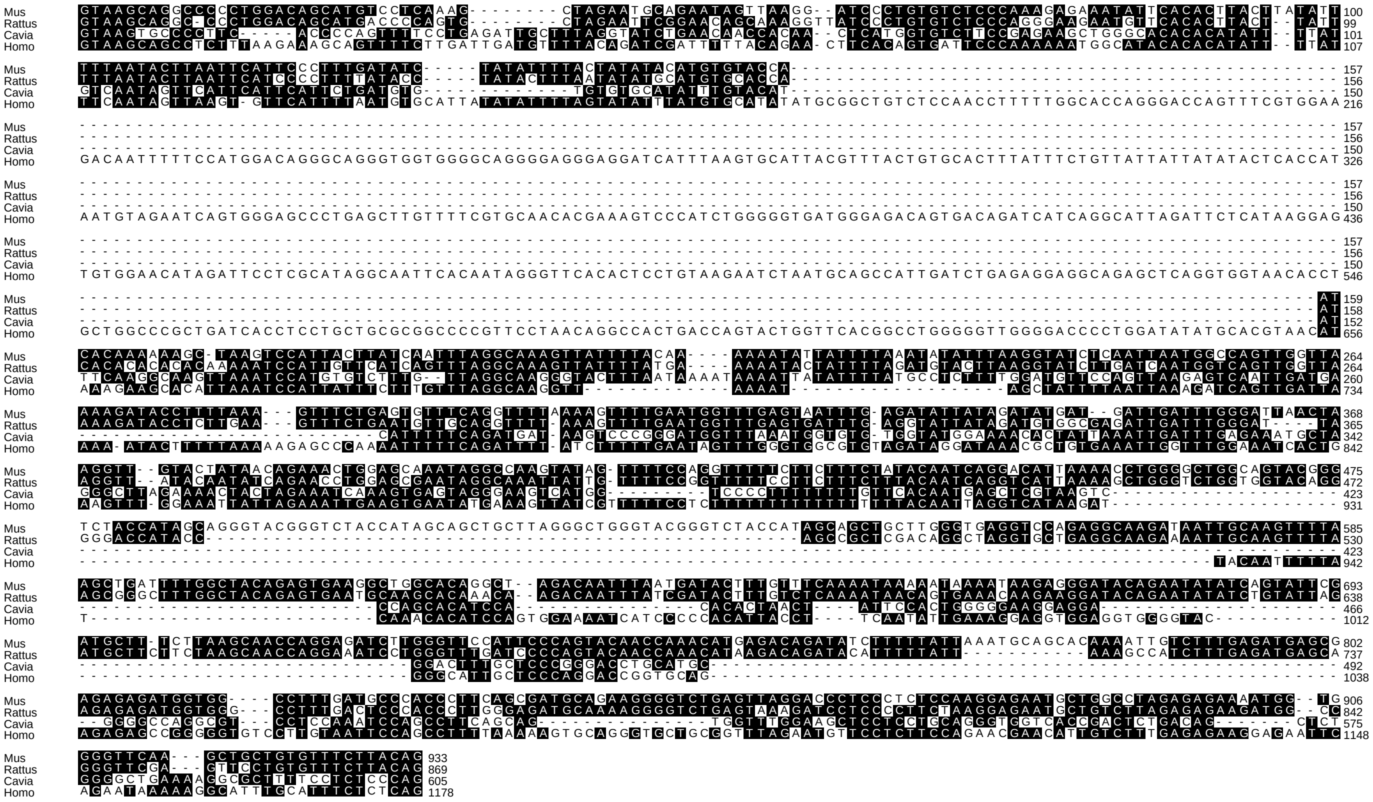
Mus
Rattus
Cavia
Homo

ENSMUSG00000007783 intron 14

Description: Carnitine O-palmitoyltransferase 1, brain isoform (Cpt1c)
Intron number: 14
Mouse chromosome: 7
Upstream exon length: 132
Downstream exon length: 153
Mouse intron length: 608
Intron alignment length: 970
Total murinae branch length: 0.14865
K_score: 0.07037
Scaling factor: 0.65235

ENSMUSG00000007783 exon 14 (ORF 0)

```
Mus     GACAGGGGTCAATTCTGCCTGACTTATGAGTCAGCCATGACCCGACTGTTCCTGGAAGGCAGGACAGAGACAGTGAGGTC 80
Rattus  GACAGGGGTCAATTCTGCCTGACTTATGAATCAGCCATGACCCGACTGTTTCTGGAAGGCAGGACGGAGACGGTGAGGTC 80
Cavia   GACAGGGGTCAATTCTGCCTGACTTACGAGTCGGCCATGACTCGCTTATTCCTGGAGGGCCGGACGGAGACAGTGCGCTC 80
Homo    GACAGGGGTCAATTCTGCCTGACTTATGAGTCGGCCATGACTCGCTTATTCCTGGAAGGCCGGACGGAGACGGTGCGGTC 80

Mus     TTGCACCAGAGAAGCCTGCCAGTTTGTGAGAGCCATGGACAACAAGGAGA---CA 132
Rattus  TTGCACTAGAGAGGCCTGCCAATTTGTGAGAGCCATGGAAAACAAGGAGAGGACA 135
Cavia   TTGCACCAGGGAGGCCTGCGACTTTGTGAGCGCCATGGAGGACCCGGAGAAGACA 135
Homo    TTGCACGAGGGAGGCCTGCAACTTTGTCAGGGCCATGGAGGACAAAGAGAAGACG 135
```

ENSMUSG00000007783 exon 15 (ORF 0)

```
Mus     GACCAACATTGCCTCGCCCTGTTCCGAGTGGCCGTGGACAAGCACCAGGCTCTACTGAAGGCGGCGATGAGTGGCCAGGG 80
Rattus  GACCAACAGTGCCTTGCCCTGTTCCGGGAAGCCGTGGACAAGCACCAGGCCCTGCTGAAGGCAGCAATGAGTGGGCAAGG 80
Cavia   GACACCACAGTGCCTCGCCCTGTTCCGTGCGGCGGTGGACAAGCACCAGGCTCTGCTCAAGGCGGCCCTGAGCGGCCAGGG 80
Homo    GACCCACAGTGCCTCGCCCTGTTCCGCGTGGCAGTGGACAAGCACCAGGCTCTGCTGAAGGCAGCCATGAGCGGGCAGGG 80

Mus     GATTGACCGTCACCTCTTTGCACTGTACATCATGTCCCGGCTCCTCCATATGCAGTCGCCCTTCCTGACCCAG 153
Rattus  GATCGACCGTCACCTCTTCGCACTGTACATCATGTCCCGGCTTCTCCACATGCAGTCGCCCTTCCTGACCCAG 153
Cavia   CGTCGACCGCCACCTCTTCGCGCTCTACATCGTGTCGCGACTCCTCCGCATGCAGTCGCCCTTCCTGGACCAG 153
Homo    AGTTGACCGCCACCTGTTTGCGCTGTACATCGTGTCCCGATTCCTCCACCTGCAGTCGCCCTTCCTGACCCAG 153
```

ENSMUSG00000007783_intron_14

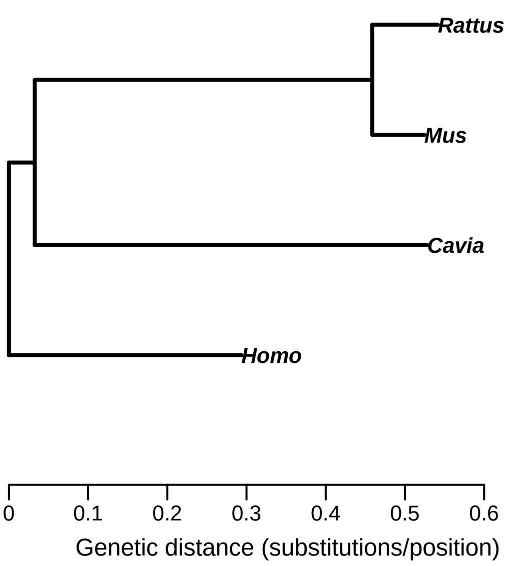

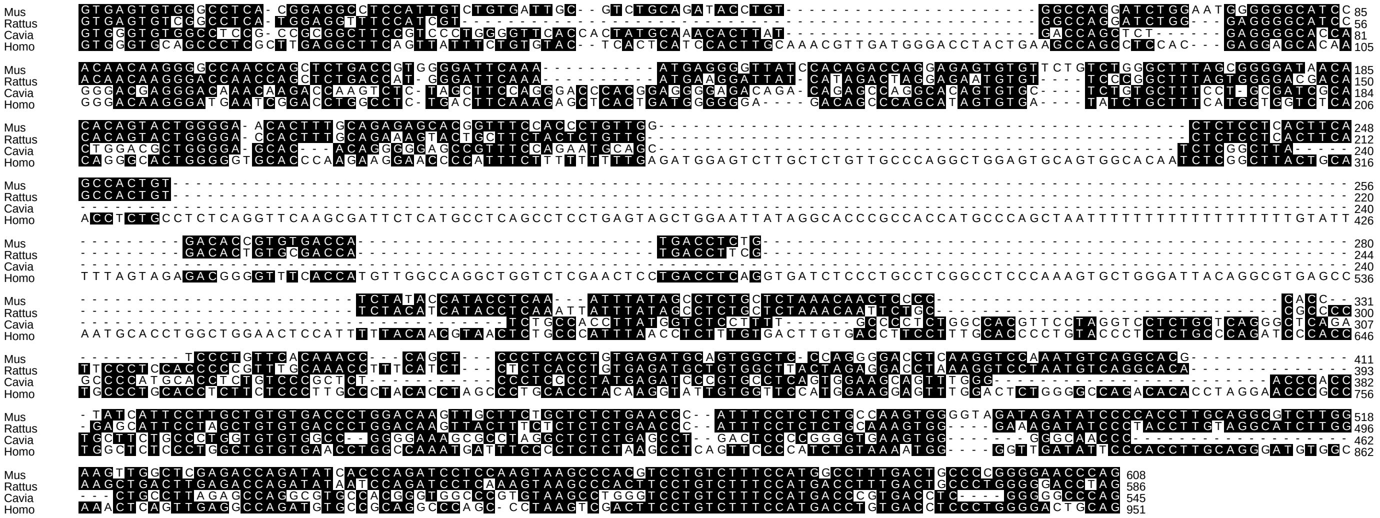
Mus
Rattus
Cavia
Homo

ENSMUSG00000034918 intron 11

Description: protocadherin 24 (Cdhr2)
Intron number: 11
Mouse chromosome: 13
Upstream exon length: 234
Downstream exon length: 156
Mouse intron length: 787
Intron alignment length: 1665
Total murinae branch length: 0.29201
K_score: 0.06777
Scaling factor: 0.65423

ENSMUSG00000034918 exon 11 (ORF 0)

```
Mus     TGTCAACGACCACAAGCCGGAATTTTACAACTGCAGCCTCCCTGGCTGCTCCTTTAGCCCCCAGGAGGCCCAAGTCAACT 80
Rattus  TGTCAACGACCACAAGCCGGAATTTTACAACTGCAGCCTCCAAAACTGCACCTTCAGCCCCCAGGAGGCCCAGGACAACT 80
Cavia   TGTCAATGACCACAAACCGGAGTTTTACAACTGCGTCCTCGAAGACTGCTCCTTCACCCCACAAGAGGCCCAGGACACTT 80
Homo    CGTCAATGACCACAAACCTGAGTTTTACAACTGCAGCCTCCCAGCCTGCACCTTCACCCCCGAAGAGGCCCAAGTGAACT 80

Mus     TCATAGGCTACGTGGACGAGCATGCCTCTGCCCGAATCTCCATCGATGGTCTGACCATGGTGGCCTATGATCCAGACCAG 160
Rattus  TCACAGGCTATGTGGATGAGCATGCCTCTGCCAGAATCTCCATCGATGGTCTGACCATGGTGGCCTACGACCCAGATCAG 160
Cavia   TCACTGCCTATGTGGACGAGCATGTCTCCGCCCGCATCCCAATTGACAATCTCACCATGGTGGCCTACGACCCAGACAAG 160
Homo    TCACTGGCTACGTGGACGAGCATGCCTCCCCCCGCATCCCCATCGATGACCTCACCATGGTGGTCTACGACCCGGACAAG 160
```

ENSMUSG00000034918 exon 12 (ORF 0)

```
Mus     GGCGACAATGGTACCTTCCTGCTGTCCCTGAATGGCCAGGATGCTGAAGCCTTCAATGTGTCCCCAGAGCGAGCAGCAGG 80
Rattus  GGTGCCAATGGCACGTTCCTGCTTTCCCTGAATGGCACAGATGCTGAAGCCTTCAACGTGTCCCCAGAGCGAGCGGCCGT 80
Cavia   GGCAACAACGGCACCTTCCTGCTGTCCCTGGGGGGCCCCGATGCTGGAGCCTTCAGCGTGTCCCCTGGACGGGCAGCAGG 80
Homo    GGCAGCAATGGCACCTTCCTGTTGTCGCTGGGGGGCCCCGATGCAGAAGCCTTCAGCGTCTCCCCGGAGCGGGCAGTGGG 80

Mus     GTCTGTCAGTGTGCAGGTGGGTGGTGAGAAACTCAGAGATGGTGGACTATGAGAAGGAGACAGTGATGGTTGTGGAG 156
Rattus  GTCTGTCAGTGTGCAGGTGGGTGGTGAAAAATTCAGAGATGGTGGACTACGAAAAGAAGACGGAGATGCAAGTGCAG 156
Cavia   CTCAGCCGACGTGCAGGTGCTGGTGAGAAATTCAGAGATGGTGGACTATGAGAACAAGAACGTGATGCTGGTGCAG 156
Homo    CTCAGCCTCCGTTCAGGTGCTGGTGAGAGTATCCGCGCTGGTGGACTACGAGAGGCAGACGGCGATGGCGGTGCAG 156
```

ENSMUSG00000034918_intron_11

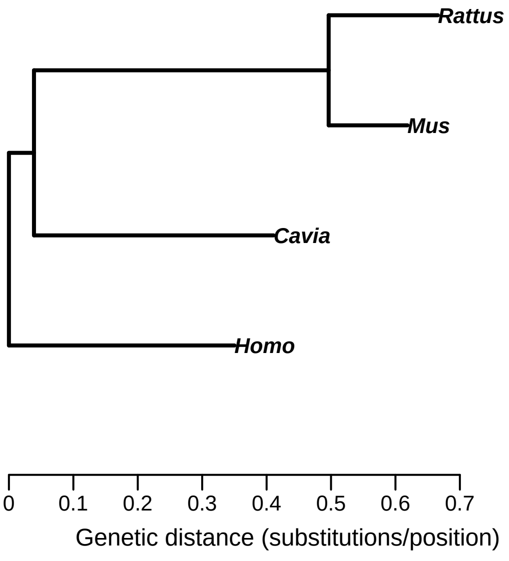

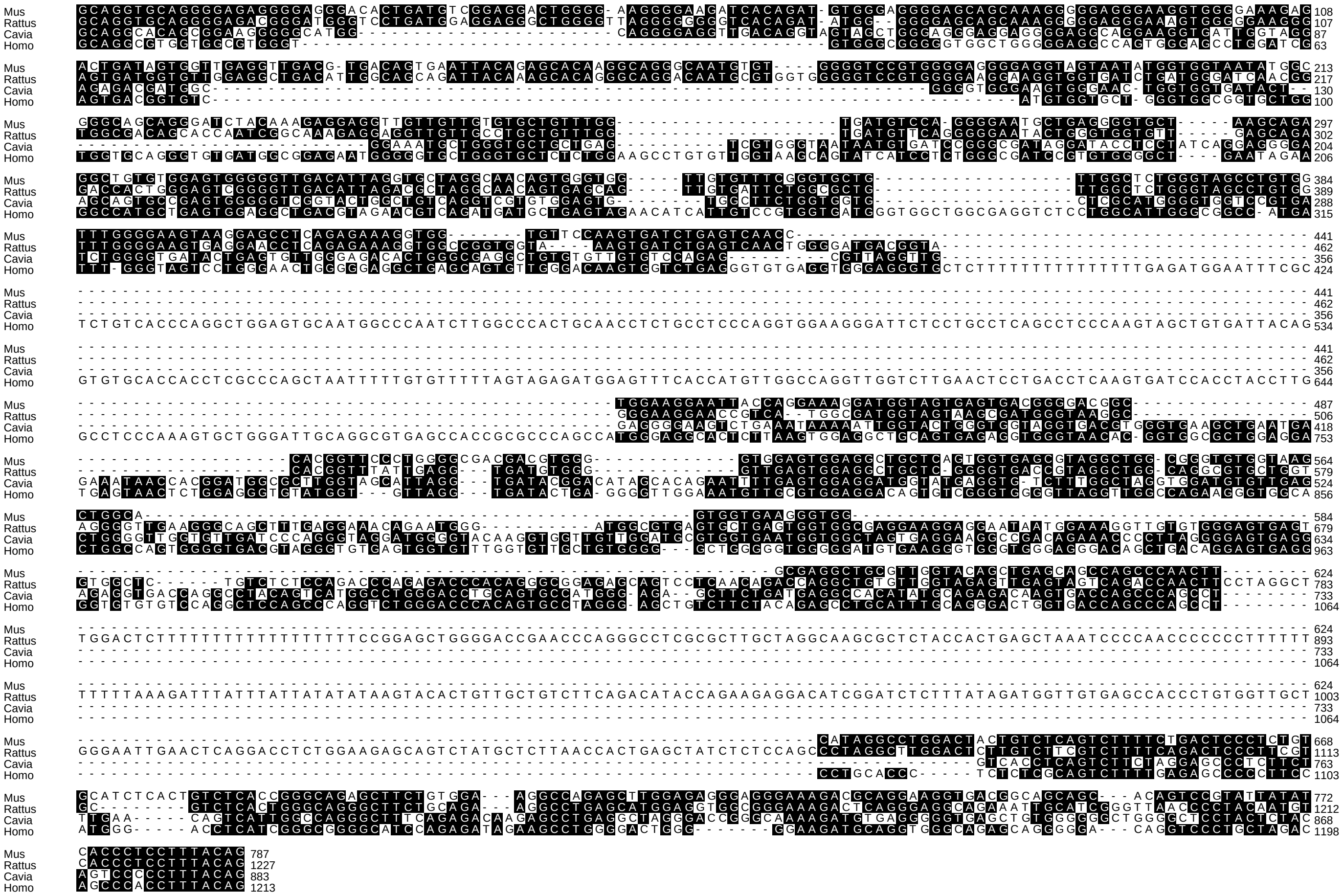

ENSMUSG00000042185 intron 23

Description: Nuclear factor related to kappa-B-binding protein (Nfrkb)
Intron number: 23
Mouse chromosome: 9
Upstream exon length: 107
Downstream exon length: 136
Mouse intron length: 731
Intron alignment length: 1075
Total murinae branch length: 0.20758
K_score: 0.07054
Scaling factor: 0.6551

ENSMUSG00000042185 exon 23 (ORF 0)

Mus	GGGAAACTGCCTACCAGAATCACAGTTCCTCTTTCTGTGATTAGCCAGCCAATGAAGGGCAAGAGCGTGGTCACAGCCCC	80
Rattus	GGGAAGCTGCCTACCAGGATCACAGTTCCTCTCTCTGTGATTAGCCAGCCAATGAAGGGCAAGAGCGTGGTCACAGCCCC	80
Cavia	GGGAAGCTGCCTACCCGGATCACAGTGCCGCTGTCGGTGATTAGCCAGCCAATGAAGGGCAAGAGCGTGGTCACGGCCCC	80
Homo	GGGAAGTTGCCTACACGGATCACAGTTCCCCTCTCTGTGATCAGCCAGCCAATGAAGGGCAAGAGCGTGGTCACAGCCCC	80

Mus	CATCATCAAAGGCAACCTTGGAGCCAA	107
Rattus	CATCATCAAAGGCAACCTTGGAGCCAA	107
Cavia	CATCATCAAAGGCAACCTTGGAGCCAA	107
Homo	CATCATCAAAGGCAACCTTGGAGCCAA	107

ENSMUSG00000042185 exon 24 (ORF 1)

Mus	TCTCAGTGGGCTGGGTCGCAACATCATCCTCACGACCATGCCAGCAGGTACCAAACTCATTGCTGGCAATAAGCCAGTGA	80
Rattus	TCTCAGTGGGCTGGGTCGCAACATCATCCTCACAACCATGCCAGCGGGTGCTAAGCTCATTGCTGGCAATAAGCCAGTGA	80
Cavia	CCTCAGTGGCTTGGGCCGCAATATCATTCTCACAACCATGCCAGCAGGTACCAAGCTCATCGCTGGCAACAAACCTGTCA	80
Homo	CCTCAGTGGGTTGGGCCGCAACATCATCCTCACAACTATGCCAGCAGGCACTAAGCTCATTGCTGGCAATAAGCCTGTTA	80

Mus	GTTTCCTCACTGCTCAGCAGTTGCAGCAGCTTCAGCAACAAGGTCAGGCTACACAG	136
Rattus	GTTTCCTCACCGCCCAGCAGTTGCAGCAGCTTCAGCAGCAAGGTCAGGCTACACAG	136
Cavia	GCTTCCTCACTGCACAGCAGCTACAGCAGCTTCAGCAGCAAGGTCAGGCCACACAG	136
Homo	GTTTCCTCACTGCTCAGCAGTTGCAGCAGCTTCAGCAGCAAGGTCAGGCCACACAG	136

ENSMUSG00000042185_intron_23

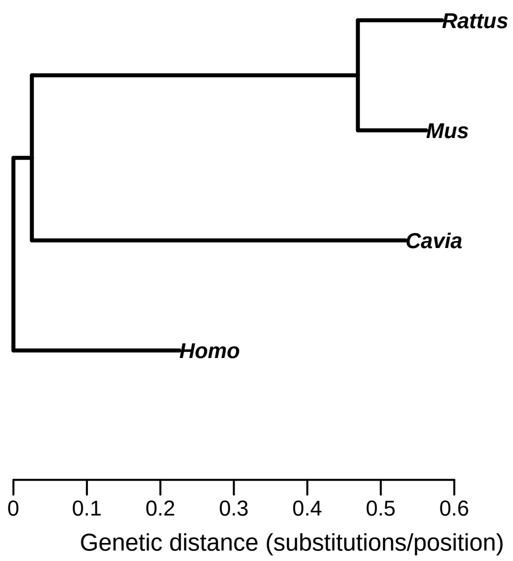

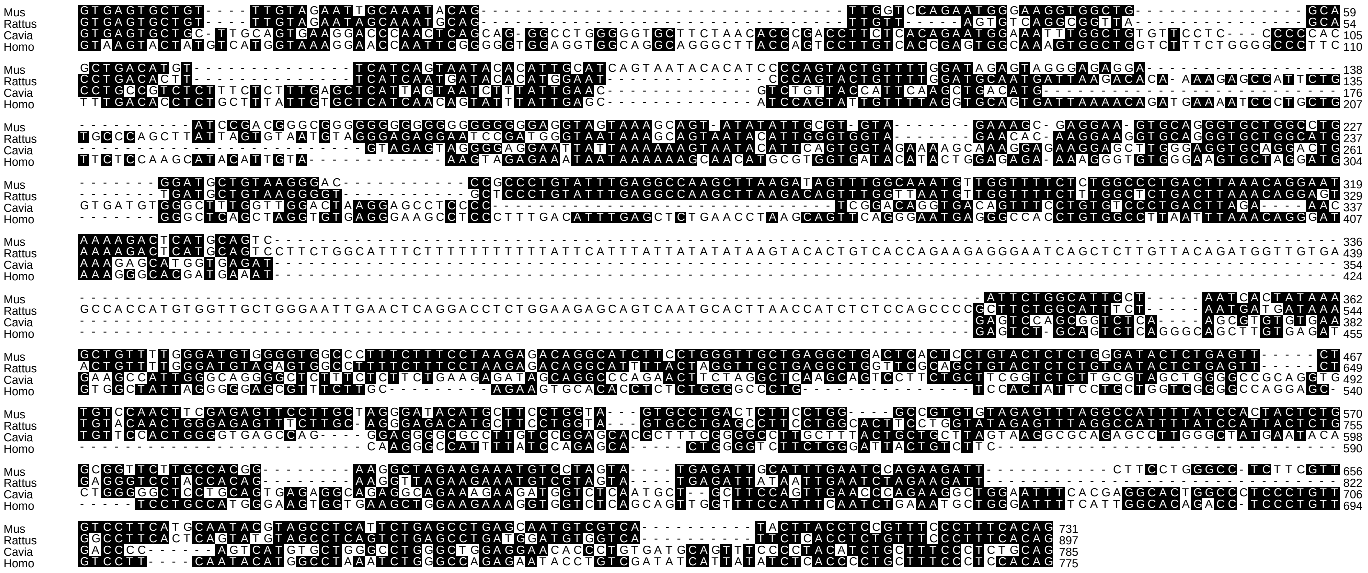

ENSMUSG00000030352 intron 4

Description: Tetraspanin-9 (Tspan9)
Intron number: 4
Mouse chromosome: 6
Upstream exon length: 102
Downstream exon length: 132
Mouse intron length: 551
Intron alignment length: 743
Total murinae branch length: 0.18979
K_score: 0.07622
Scaling factor: 0.65599

ENSMUSG00000030352 exon 4 (ORF 0)

```
Mus     GTGAACGAGAATGCCAAGCAGGACCTGAAAGAGGGGCTGCTGCTCTACAACACAGAGAACAACGTGGGGCTCAAGAACGC 80
Rattus  GTGAACGAGAATGCCAAGCAGGACCTGAAAGAGGGGCTACTGCTGTACAACACTGAGAACAACGTGGGGCTCAAGAATGC 80
Cavia   GTGAATGAGAATGCCAAGCAGGACCTGAAGGAAGGACTGCTTCTCTATAACACTGAGAACAACGTGGGGCTCAAGAATGC 80
Homo    GTGAACGAGAACGCCAAGAAGGACCTGAAGGAAGGCCTGCTGCTGTACCACACCGAGAACAACGTGGGGCTGAAGAACGC 80

Mus     CTGGAACATCATCCAGGCCGAG 102
Rattus  CTGGAATATCATCCAGGCCGAG 102
Cavia   CTGGAACATTATCCAGGCTGAG 102
Homo    CTGGAACATCATCCAGGCTGAG 102
```

ENSMUSG00000030352 exon 5 (ORF 0)

```
Mus     ATGCGGTGTTGTGGGGTCACTGACTACACAGACTGGTATCCGGTGCTCGGGGAGAACACAGTTCCTGACAGATGCTGCAT 80
Rattus  ATGCGGTGTTGTGGGGTCACTGACTATACAGACTGGTATCCAGTGCTCGGGGAGAACACAGTTCCTGACCGCTGCTGCAT 80
Cavia   ATGCGATGCTGTGGGGTCACTGACTACTCAGACTGGTACCCAGTGCTGGGGGAGAACACAGTGCCTGACCGTTGCTGCAT 80
Homo    ATGCGATGCTGTGGTGTCACTGACTACACAGACTGGTACCCAGTGCTGGGGGAGAACACGGTTCCCGACCGCTGCTGCAT 80

Mus     GGAGAATTCCCAGGGCTGTGGGCGCAACAGCACCACCCCCTTGTGGAGAACG 132
Rattus  GGAGAATTCCCAGGGTTGCGGGCGCAACAGCACCACCCCCTTGTGGAAAACG 132
Cavia   GGAGAATTCCCAGGGCTGTGGGCGAAACAGCACTACCCTTGTGTGGAAAACG 132
Homo    GGAGAACTCCCAGGGCTGCGGGCGCAACGCCACCACGCCTTTGTGGAGAACG 132
```

ENSMUSG00000030352_intron_4

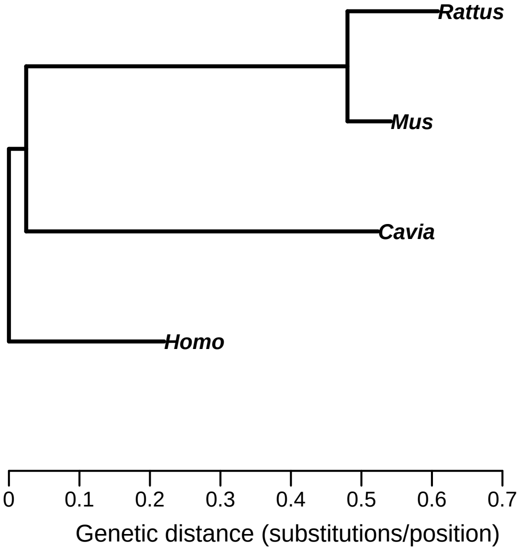

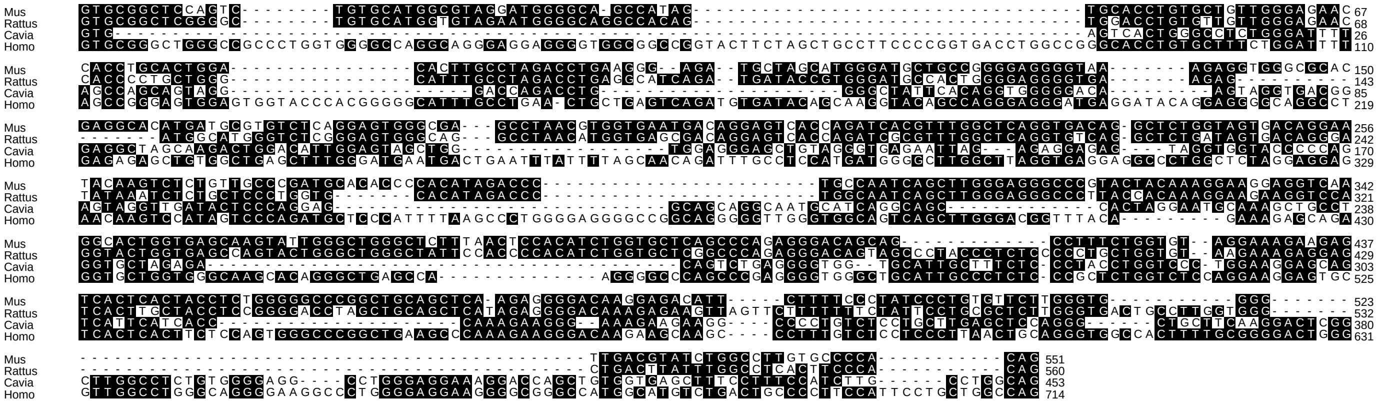
Mus
Rattus
Cavia
Homo

ENSMUSG00000006021 intron 11

Description: Kaptin (Kptn)
Intron number: 11
Mouse chromosome: 7
Upstream exon length: 171
Downstream exon length: 126
Mouse intron length: 1010
Intron alignment length: 1087
Total murinae branch length: 0.10397
K_score: 0.08895
Scaling factor: 0.65898

ENSMUSG00000006021 exon 11 (ORF 0)

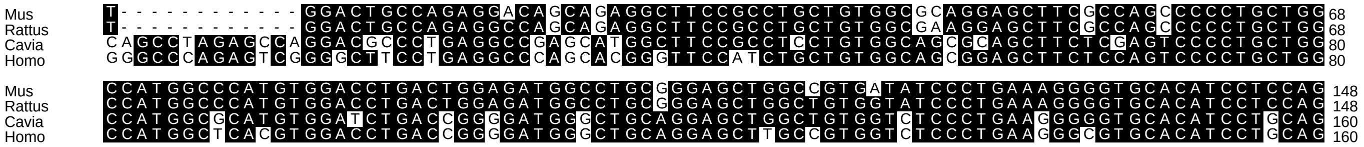

ENSMUSG00000006021 exon 12 (ORF 0)

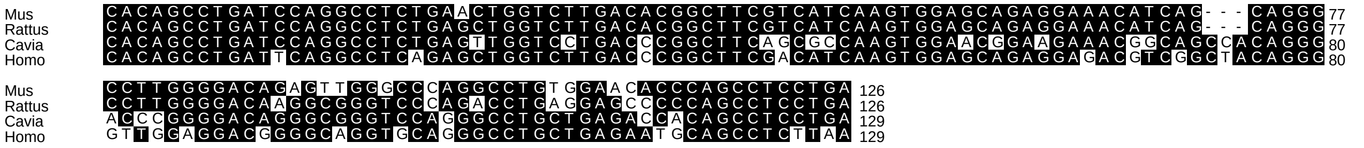

ENSMUSG00000006021_intron_11

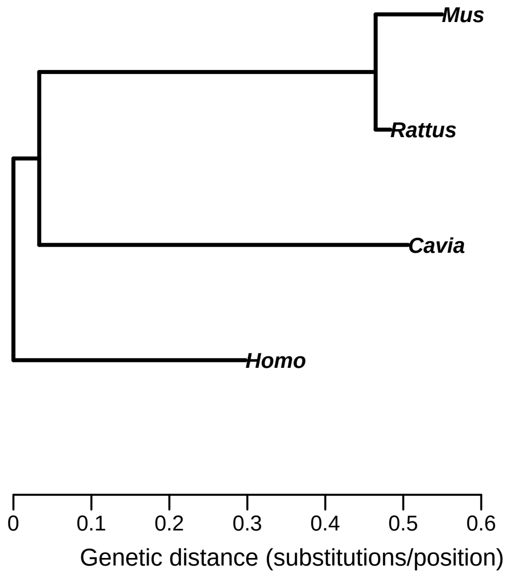

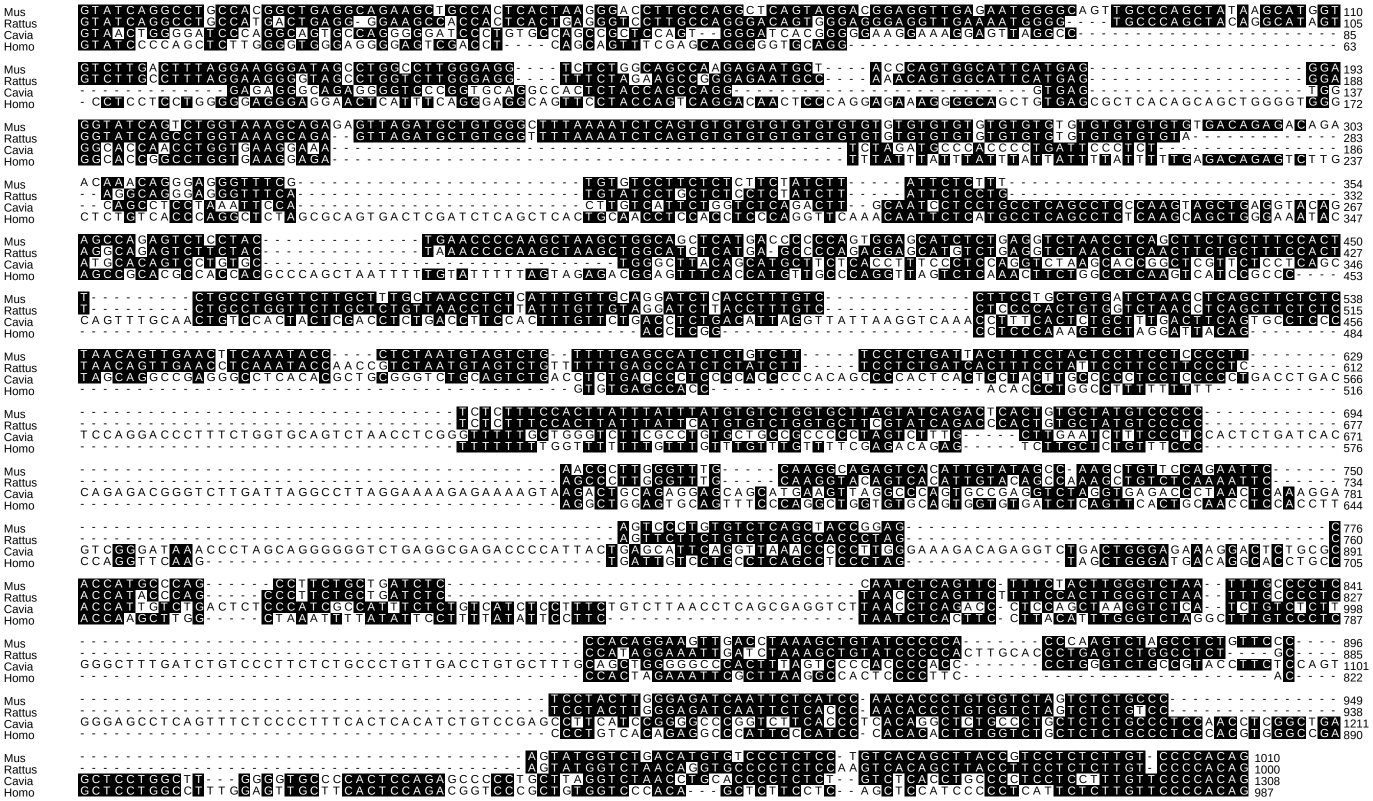

ENSMUSG00000033105 intron 15

Description: Lanosterol synthase (Lss)
Intron number: 15
Mouse chromosome: 10
Upstream exon length: 150
Downstream exon length: 97
Mouse intron length: 551
Intron alignment length: 437
Total murinae branch length: 0.25776
K_score: 0.07351
Scaling factor: 0.66046

ENSMUSG00000033105 exon 15 (ORF 0)

```
Mus     GGTGGTTTCTCCTTCAGCACACTGGACTGTGGCTGGATCGTTGCTGACTGCACAGCCGAGGGTTTGAAGGCTGTGCTGCT 80
Rattus  GGTGGTTTCCCCTTCAGCACACTGGACTGTGGCTGGATCGTTGCTGACTGCACGGCCGAGGCTTTGAAGGCTGTGCTGCT 80
Cavia   GGCGGCTTCCCCTTCAGTACCCTGGACTGTGGCTGGATCGTGGCTGACTGCACAGCTGAGGGCCTGAAGGCTGTCTTGCT 80
Homo    GGTGGCTTCTCCTTCAGTACGCTGGACTGCGGCTGGATCGTTTCTGACTGCACGGCTGAGGCCTTGAAGGCTGTGCTGCT 80

Mus     CCTGCAGAATCAGTGTCCGTCCATCACTGAGCATATCCCCCGAGAGCGGCTGTGCGATGCTGTGGATGTG 150
Rattus  CCTGCAGGAGCGGTGTCCCTCAATCACCGAGCATGTCCCCCAAGAGCGACTCTACAATGCTGTGGCTGTG 150
Cavia   CCTACAAGAGAAGTGTCCCTGTATTGCTGAACGCATCCCTCAAGAACGGCTCTGTGATGCCGTGGCTGTG 150
Homo    CCTGCAGGAGAAGTGTCCCCATGTCACCGAGCACATCCCCAGAGAACGGCTCTGCGATGCTGTGGCTGTG 150
```

ENSMUSG00000033105 exon 16 (ORF 0)

```
Mus     TTACTGAGCTTGAGGAATGCTGATGGCGGGTTCGCTACCTATGAGAAGAAGCGTGGGGGTTACTTGCTGGAGCTGCTGAA 80
Rattus  TTGTTGAGCATGAGGAATTCCGATGGAGGGTTTGCTACCTATGAGACTAAGCGTGGCGGGTATTTGCTGGAGCTGCTGAA 80
Cavia   TTACTGAACATGAGGAATCCTGATGGAGGGTTTGCCACCTATGAGACTACACGTGGGGGGCGCTTGCTGGAATTGCTTAA 80
Homo    CTGCTGAACATGAGAAATCCAGATGGAGGGTTCGCCACCTATGAGACCAAGCGTGGGGGGCACTTGCTGGAGCTGCTGAA 80

Mus     CCCCTCAGAGGTCTTTG 97
Rattus  CCCCTCAGAGGTCTTTG 97
Cavia   TCCCTCGGAGGTCTTTG 97
Homo    CCCCTCGGAGGTCTTCG 97
```

ENSMUSG00000033105_intron_15

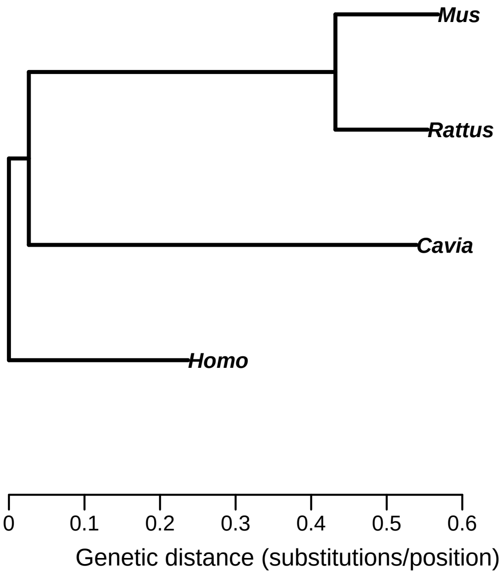

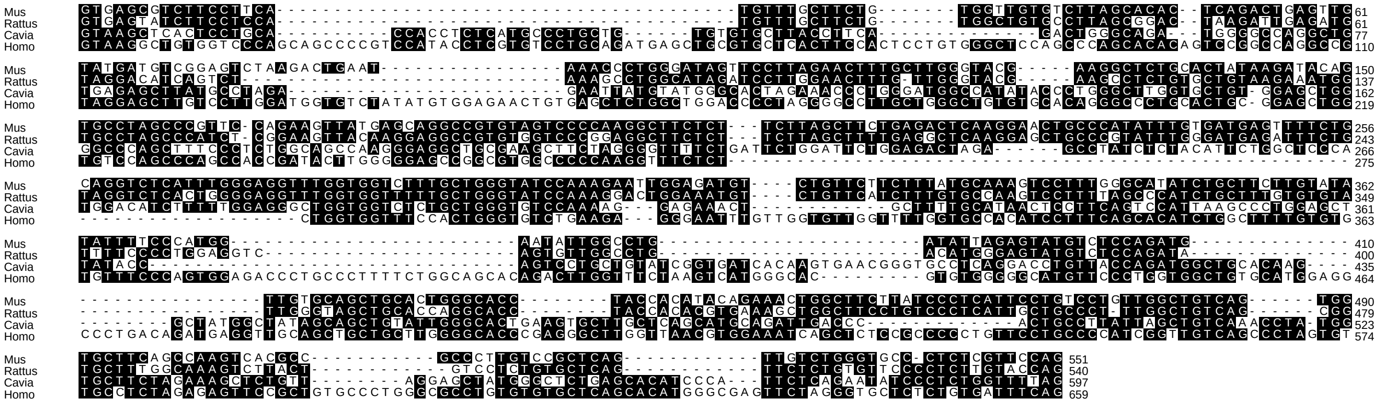
Mus
Rattus
Cavia
Homo

ENSMUSG00000003549 intron 6

Description: DNA excision repair protein ERCC-1   (Ercc1)
Intron number: 6
Mouse chromosome: 7
Upstream exon length: 100
Downstream exon length: 72
Mouse intron length: 675
Intron alignment length: 1238
Total murinae branch length: 0.21467
K_score: 0.04806
Scaling factor: 0.66201

ENSMUSG00000003549 exon 6 (ORF 1)

```
Mus     TGCAGAGGAAGCAGGGCGGTACCTGGAGACCTACAAGGCGTATGAGCAGAAGCCAGCCGACCTCCTTATGGAAAAGCTGG 80
Rattus  TGCAGAGGAAGCAGGGCGGTACCTGGAAACCTACAAGGCGTATGAGCAGAAGCCTGCTGACCTCCTTATGGAAAAGCTGG 80
Cavia   CCCCGAGGAAGCTGGACGGTACCTGGAGACCTACAAGGCCTATGAGCAGAAGCCCGCCGACCTCCTGATGGAGGAAGCTGG 80
Homo    CCCCGAGGAAGCTGGGCGGTACCTGGAGACCTACAAGGCCTATGAGCAGAAACCAGCGGACCTCCTGATGGAGGAAGCTAG 80

Mus     AGCAGAACTTCCTATCACGG 100
Rattus  AACAGAACTTCCTATCACGG 100
Cavia   AACAGAACTTTGTGTCTCGG 100
Homo    AGCAGGACTTCGTCTCCCGG 100
```

ENSMUSG00000003549 exon 7 (ORF 0)

```
Mus     GCCACTGAGTGTCTGACCACCGTGAAATCTGTGAACAAGACCGACAGCCAGACCCTCCTGGCTACATTTGGA 72
Rattus  GCCACTGAGTGTCTGACCACCGTGAAATCTGTGAACAAGACTGACAGCCAGACCCTCCTCACTACATTTGGA 72
Cavia   ATCACTGAATGTCTGACCACAGTGAAGTCAGTCAACAAAACAGACAGTCAAACACTCTTGTCCACTTTTGGG 72
Homo    GTGACTGAATGTCTGACCACCGTGAAGTCAGTCAACAAAACGGACAGTCAGACCCTCCTGACCACATTTGGA 72
```

ENSMUSG00000003549_intron_6

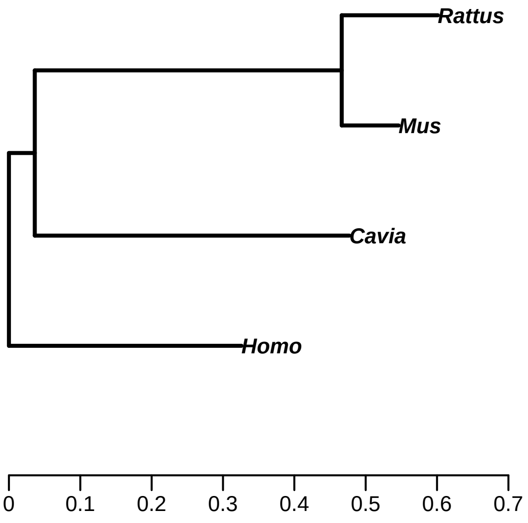

Genetic distance (substitutions/position)

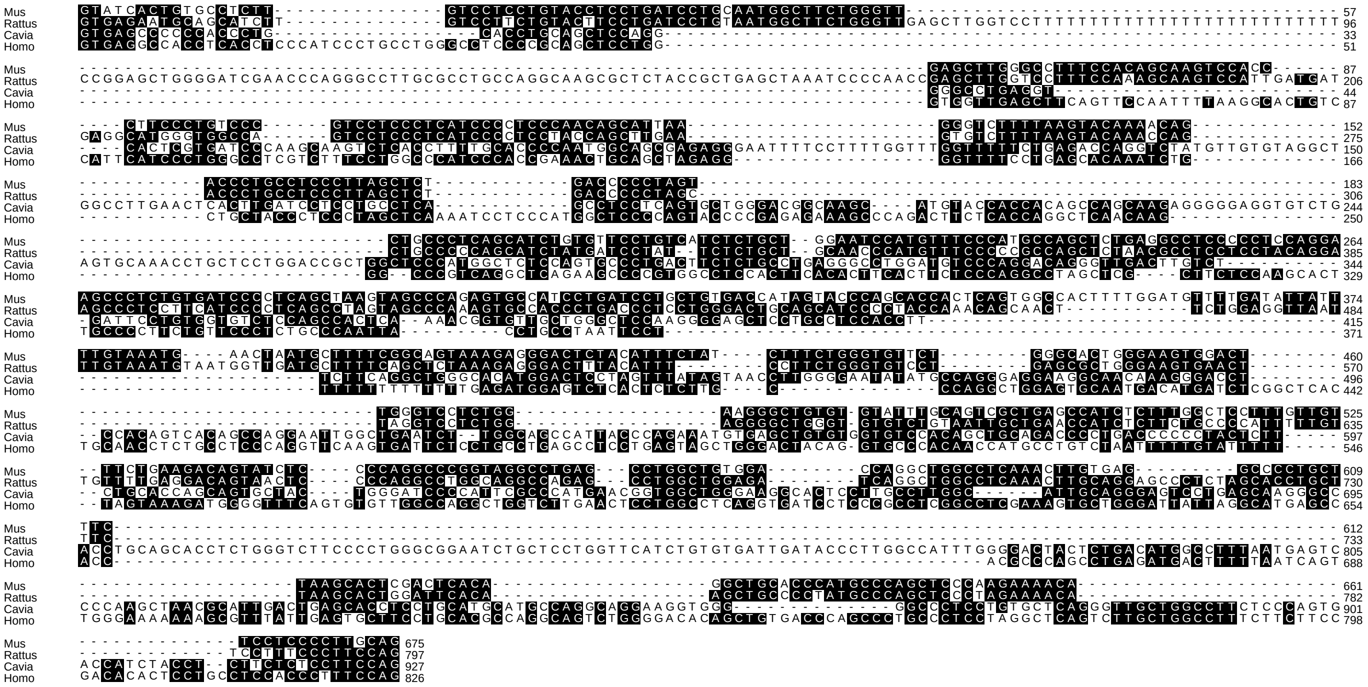
Mus
Rattus
Cavia
Homo

ENSMUSG00000009292 intron 14

Description: Transient receptor potential cation channel subfamily M memb (Trpm2)
Intron number: 14
Mouse chromosome: 10
Upstream exon length: 146
Downstream exon length: 113
Mouse intron length: 994
Intron alignment length: 1141
Total murinae branch length: 0.20666
K_score: 0.05854
Scaling factor: 0.66372

ENSMUSG00000009292 exon 14 (ORF 2)

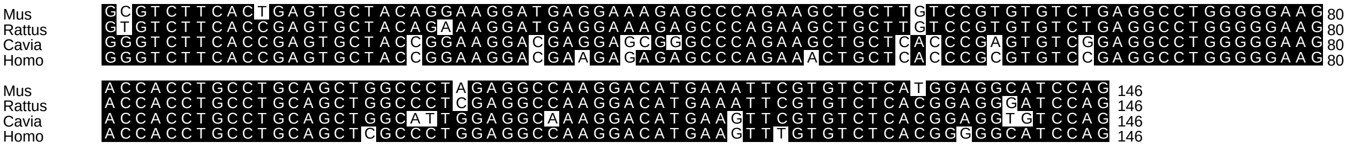

ENSMUSG00000009292 exon 15 (ORF 0)

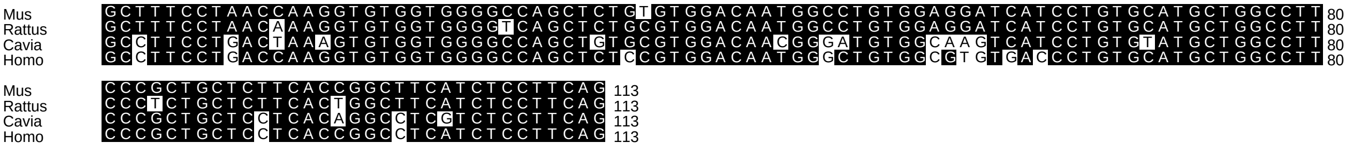

ENSMUSG00000009292_intron_14

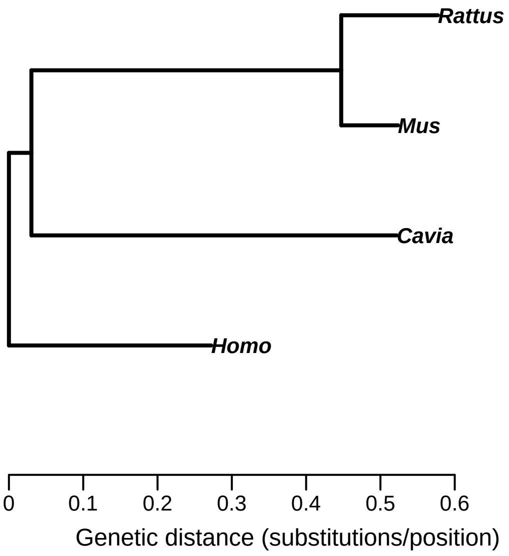

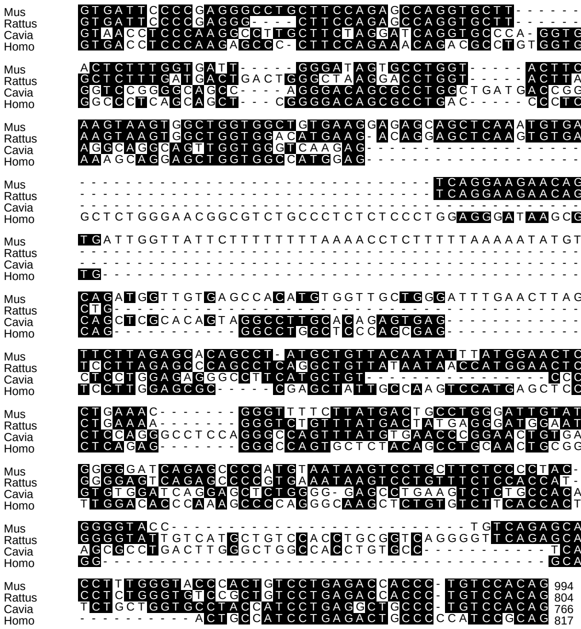
Mus
Rattus
Cavia
Homo

ENSMUSG00000039662 intron 1

Description: Protein-S-isoprenylcysteine O-methyltransferase (Icmt)
Intron number: 1
Mouse chromosome: 4
Upstream exon length: 195
Downstream exon length: 89
Mouse intron length: 1116
Intron alignment length: 1263
Total murinae branch length: 0.16615
K_score: 0.0639
Scaling factor: 0.66729

ENSMUSG00000039662 exon 1 (ORF 0)

```
Mus     CGAGGCGCGCCTCAGCCTCGCTACATTCCTCCTGGGCGCCTCGGTGCTCGCTCTGCCGCTGCTCACGCGCGCCGGCCTGC 80
Rattus  CGAGGCGCGCCTCAGCCTCGCTACCTTCCTCCTTGGTGCCTCGGTGCTCGCTCTGCCTCTGCTCACGCGCGCCGGCCTGC 80
Cavia   CGAGGCGCGCCTCAGCCTGGCCACCTTTCTGCTGGGCGCCTCGGTGCTCGCGCTGCCGCTGCTCACGCGCGCCGGCCTGC 80
Homo    TGAGGCGCGTCTCAGCCTCGCCACCTTCCTGCTGGGCGCCTCGGTGCTCGCGCTGCCGCTGCTCACGCGCGCCGGCCTGC 80

Mus     AGGGCCGCACCGGGCTGGCGCTCTACGTGGCCGGACTCAACGCGCTGCTGCTGCTACTCTACCGGCCGCCGCGCTACCAG 160
Rattus  AGGGCCGCACCGGGCTGGCGCTGTACGTGGCCGGACTCAACGCGCTGTTGCTGCTTCTCTACCGGCCGCCGCGCTACCAG 160
Cavia   AGGGCCGCACCGGGCTGGCGCTCTATGTGGCGGGGCTGAACGCGCTGCTGCTCCTGCTCTACCGGCCGCCGCGCTACCAG 160
Homo    AGGGCCGCACCGGGCTGGCGCTCTACGTGGCCGGGCTCAACGCGCTGCTGCTGCTGCTCTATCGGCCGCCTCGCTACCAG 160
```

ENSMUSG00000039662 exon 2 (ORF 0)

```
Mus     ATAGCCATCAGAGCTTGTTTCCTTGGCTTTGTGTTTGGCTGTGGTGTGCTGCTAAGTTTCAGCCAGTCCTCTTGGAATCA 80
Rattus  ATAGCCATCCGAGCTTGTTTCCTTGGCTTTGTGTTTGGCTGCGGCGTGCTGCTAAGTTTCAGCCAGTCCTCCTGGAATCA 80
Cavia   ATAGCCATCCGAGCTTGCTTCCTTGGCTTCGTGTTCGGCTGCGGCGTGCTGCTCAGCTTTAGCCAGTCCTCTTGGAATCA 80
Homo    ATAGCCATCCGAGCTTGTTTCCTGGGGTTTGTGTTCGGCTGCGGCACGCTGCTAAGTTTTAGCCAGTCTTCTTGGAGTCA 80

Mus     CTTTGGCTG 89
Rattus  CTTCGGCTG 89
Cavia   CTTTGGCTG 89
Homo    CTTTGGCTG 89
```

ENSMUSG00000039662_intron_1

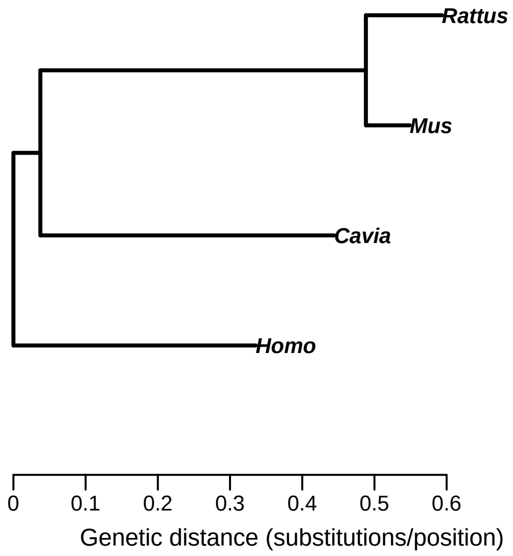

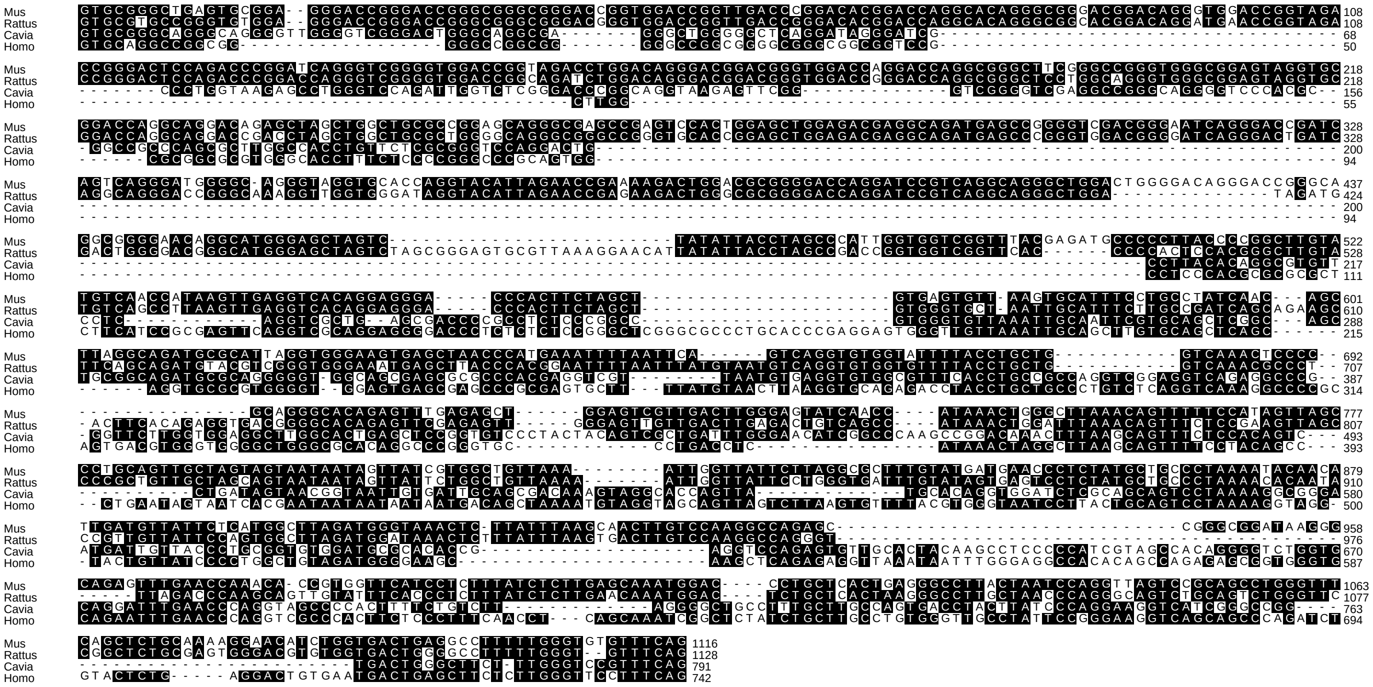
Mus
Rattus
Cavia
Homo

ENSMUSG00000032079 intron 2

Description: Apolipoprotein A-V Precursor (Apoa5)
Intron number: 2
Mouse chromosome: 9
Upstream exon length: 100
Downstream exon length: 967
Mouse intron length: 442
Intron alignment length: 564
Total murinae branch length: 0.23879
K_score: 0.05467
Scaling factor: 0.66747

ENSMUSG00000032079 exon 2 (ORF 2)

```
Mus     TGTTCGCAAGCACTCAGGCACGGAAGAGCCTCTGGGACTACTTCAGCCAAAACAGTTGGAGCAAAGGCGTGATGGG---- 76
Rattus  TGTTTGCAACTGTACAGGCGAGGAAGAGCTTCTGGGAGTACTTCGGCCAGAACAGCCAGGGCAAAGGCATGATGGG---- 76
Cavia   CATGCTCAGCCACCCAGGCACAGAAAGGCTTCTGGGATTACTTCAGCCAGAGCAGCAGGGAGAAGAGTGTGGTGGGGCCG 80
Homo    CGTTTTCGGCCACCCAGGCACGGAAAGGCTTCTGGGACTACTTCAGCCAGACCAGCGGGGACAAAGGCAGGGTGGAGCAG 80

Mus     --CCAGCCACAGAAGCTGGCACAGGA------ 100
Rattus  --CCAGCAGCAGAAGCTGGCACAGGA------ 100
Cavia   ATCCAGCAGCAGAAGCAGGCCCAGGACCACGG 112
Homo    ATCCATCAGCAGAAGATGGCTCGCGAGCCCGC 112
```

ENSMUSG00000032079 exon 3 (ORF 1)

```
Mus     GAACCTGAAAGGCAGCTTCGAGCAAGACCTCTACAATATGAACAATTACCTAGAAAAGCTGGGACCCTTGAGAGGGCCTG 80
Rattus  GAGCCTGAAAGGTAGCTTGGAGCAAGACCTCTACAATATGAACAATTTCCTAGAAAAGCTGGGACCCTTGAGAGAGCCTG 80
Cavia   GAGGCTGAAAGACAGCCTAGAGCAGGACCTCAGCGATATGAACAAATTCCTGGAGAAGCTGGGCCCCCTGAGTGGGCAGG 80
Homo    GACCCTGAAAGACAGCCTTGAGCAAGACCTCAACAATATGAACAAGTTCCTGGAAAAGCTGAGGCCTCTGAG------TG 74

Mus     GGAAGGAGCCTCCTCTGCTGGCCCAGGACCCAGAAGGCATTCGGAAACAGCTGCAGCAGGAGCTGGGGGAAGTGAGCTCG 160
Rattus  GGAAGGAGCCTCCTCGGCTGGCACAGGATCCAGAAGGCATTCGGAAGCAGTTGCAGCAAGAGCTGGAGGAAGTGAGCACA 160
Cavia   GGAAACAGCCTCCTCCTCTGACCCAG------GAGGACATGCGGCAGCAGCTGCAGGAGGAGCTAGAGCAGGTGCGGGCT 154
Homo    GGAGCGAGGCTCCTCGGCTCCCACAGGACCCGGTGGGCATGCGGCGGCAGCTGCAGGAGGAGTTGGAGGAGGTGAAGGCT 154
```

ENSMUSG00000032079_intron_2

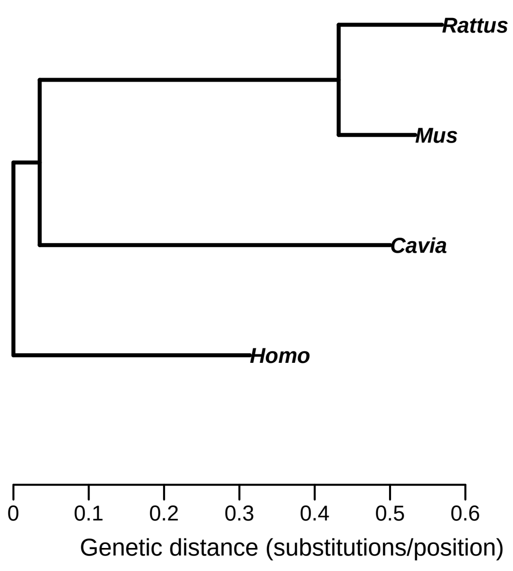

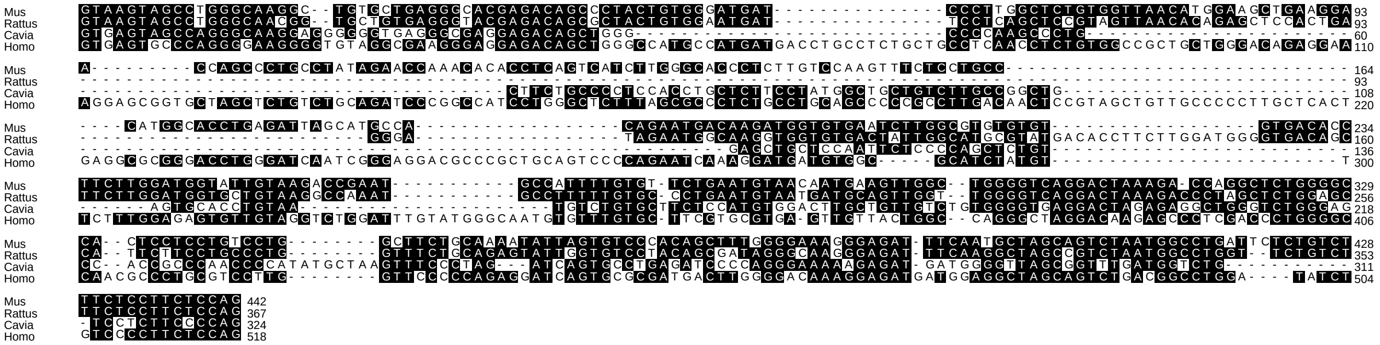
Mus
Rattus
Cavia
Homo

ENSMUSG00000053024 intron 5

Description: Contactin-2 Precursor (Cntn2)
Intron number: 5
Mouse chromosome: 1
Upstream exon length: 210
Downstream exon length: 100
Mouse intron length: 281
Intron alignment length: 338
Total murinae branch length: 0.20933
K_score: 0.07051
Scaling factor: 0.66776

ENSMUSG00000053024 exon 5 (ORF 2)

```
Mus     GATGGGCGTCACTTCGTGTCCCAGACCACAGGGAACCTGTACATCGCCCGCACCAATGCCTCGGACCTGGGCAACTACTC 80
Rattus  GATGGGCGACACTTTGTGTCCCAGACTACAGGAAACCTGTACATCGCCCGGACCAATGCCTCAGACCTGGGCAACTACTC 80
Cavia   GATGGGCGTCACTTCGTGTCCCAGACCACAGGGAACCTATACATTGCCCGGACCAATGCCTCTGACCTGGGCAACTACTC 80
Homo    GACGGGCGTCACTTCGTGTCCCAGACCACAGGGAACCTGTACATTGCCCGAACCAATGCCTCAGACCTGGGCAACTACTC 80

Mus     TTGCCTGGCTACCAGCCACCTGGACTTCTCCACCAAGAGCGTCTTCAGCAAATTCGCGCAGCTCAACCTGGCTGCTGAAG 160
Rattus  TTGTTTGGCTACCAGCCACATGGACTTTTCCACCAAGAGTGTCTTCAGCAAATTTGCGCAGCTCAACCTGGCTGCGGAAG 160
Cavia   CTGCCTGGCCACAAGCCACATGGACTTCTCCACCAAGAGTGTCTTCAGCAAATTCGCACAGCTTAACCTGGCTGCAGAAG 160
Homo    CTGTTTGGCCACCAGCCACATGGACTTCTCCACCAAGAGCGTCTTCAGCAAGTTTGCTCAGCTCAACCTGGCTGCTGAAG 160
```

ENSMUSG00000053024 exon 6 (ORF 2)

```
Mus     ATCCTCGACTCTTTGCTCCCAGTATCAAAGCCCGGTTCCCCCCAGAGACGTACGCACTGGTTGGGCAGCAGGTCACCCTG 80
Rattus  ATCCCCGACTCTTCGCTCCCAGTATCAAAGCTCGGTTCCCCCCGGAGACCTACGCACTAGTTGGGCAGCAAGTCACCCTG 80
Cavia   ATCCCCGGCTTTTTGCACCCAGCATCAAGGCCCGGTTCCCTGCAGAGACCTATGCACTGGTGGGACAGCAGGTCACCCTG 80
Homo    ATACCCGGCTCTTTGCACCCAGCATCAAGGCCCGGTTCCCAGCAGAGACCTATGCACTGGTGGGGCAGCAGGTCACCCTG 80

Mus     GAGTGCTTTGCCTTTGGGAA 100
Rattus  GAGTGCTTTGCCTTTGGGAA 100
Cavia   GAATGCTTTGCCTTTGGGAA 100
Homo    GAGTGCTTCGCCTTTGGGAA 100
```

ENSMUSG00000053024_intron_5

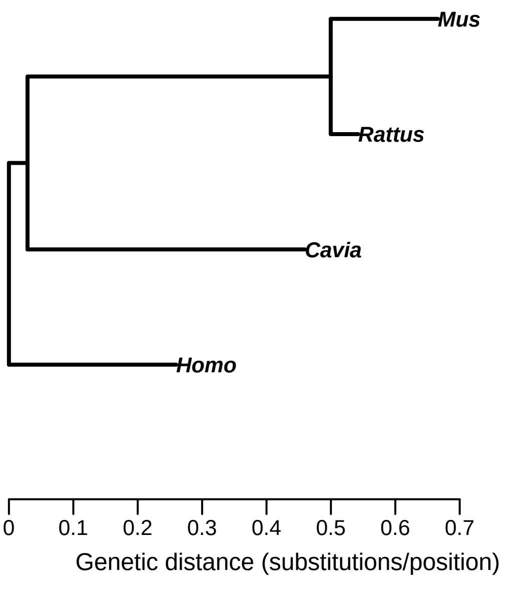

```
Mus     GTCAGGATC------------TAGGGTAATGGA---GGCTGAAAGGGTCAATAGGCCTTGGAGAAATGGTATTCTACC--AAGCTTAAGAATTGCAGCTCAGAATATAT- 92
Rattus  GTCAGGATC------------TAGGGTAATGGAGGGGGCTGAAAGAGTTCACAGGCTTTGGGGAAATGGTCTTCCACC--AAGCCTAAGAACCGCAGCTCA------AT- 89
Cavia   GTCAGGGTCTCTGGGCGAGCATGGCAGGAGGGG-------------------------TGGGAAAACGGGATTCCCCTTGGTAATGGTTAGGAGCAGCTCACAGGGCAT- 84
Homo    GTCAGGCTT---GGCCAGGCGTGGCTGGAGGGAGGGAACTGGAAGGGTCAGCGGGCATTAGGAAAAGGGTTTTTCCTTTGGAGATTG-GAAGATCAGCTTGCAGGGCACT 106

Mus     -----------------------GAGGTCCTGCTGCCGGGTCTCAGATCCTTGCCCCAGGCCCCCC-AGAAAAAGAGAACATTTGGAGATTCCAAATATTTATGTGGTGA 178
Rattus  -----------------------ACGGCCCGGCTGCCAGGTCTCAGACCCTTGCCCCAGGCCCCCA-CGGGAAAGGGAATGTTTGGAGATTCCAAACATTTATTTGGAGA 175
Cavia   --------------GGCACCCTGGGCCTCCTGCAGACAGGCCTTGGACCCTGTGCCTGGCCCAAGAATAGGCATGAGAGACTCCTGAGATCCCACCCGACTGCGCAGCAG 180
Homo    GATTCCAGGCCCTGGACCCCCAGATCCTCCTGCTTCAAATCCTGAGGCCCTTGCCCCAGGCCTCAA------------------GGAGATTCCACACAGCTGCCTAGACA 198

Mus     TCTGTGAACTTTGGAAGGCAT-----------GGGCCCTAATACAAAG----CACCTCCCTGGAGCCCTTTGCATGTGGTTCACACAGTCAATCTCTCTCTCCCCTCCAC 273
Rattus  TTCACGAGCTTTGGAAGGCAC-----------GGGCCCTA-CACAAAG----CACCTCCCTGGAGTCCTTTGTGTATGATTCACACAACCTCTCTCTCTGTCCCC----- 264
Cavia   --CACAAGCTCCAACAAGCGCTGTGGTTCTGAAATCCTGAGCACCAGAGCCACGCCTCCCTGGAGTCA-CCGCATGTGATTTGCAAGGCCCTTCTCTCCC---------- 277
Homo    -GAGTTGGCTCTGAAAGGTGCTGAGATCCATGCACGGGAGCACCTGA-----------CCTGGAGTCATCTGCATCTGATTTGTAAAACCCTCTCTCCC----------- 286

Mus     CCCCCCAG 281
Rattus  -----CAG 267
Cavia   -----CAG 280
Homo    -----CAG 289
```

ENSMUSG00000015971 intron 2

Description: Actin-related protein 8 (Actr8)
Intron number: 2
Mouse chromosome: 14
Upstream exon length: 171
Downstream exon length: 111
Mouse intron length: 1260
Intron alignment length: 1772
Total murinae branch length: 0.18658
K_score: 0.08644
Scaling factor: 0.66793

ENSMUSG00000015971 exon 2 (ORF 0)

```
Mus     CAACTTCATTGTTGTCATACACCCAGGTTCAACAACTCTAAGACTTGGCCGAGCCACAGATACACTTCCGGTCAGTGTTC 80
Rattus  CAACTTTATTGTCGTCGTACACCCAGGCTCAACAACGTTAAGAATCGGCCGAGCCACAGACACGCTCCCAGCCAGCATTC 80
Cavia   CAACTTTATTGTGGTCATACATCCAGGCTCAACAACTTTAAGAATCGGACGAGCTACAGACACACTCCCCGCCAGTGTTC 80
Homo    CAACTTCATCATTGTCATACATCCAGGTTCAACAACTTTAAGGATTGGTCGAGCCACAGACACTCTTCCTGCCAGCATTC 80

Mus     CTCACGTCATTGCACGAAGACACAAACAACAAGGGCAGCCCCTGTACAAGGACAACTGGCTCCTAAGGGAAGGACTAAAT 160
Rattus  CTCACGTCATTGCTCGGAGACACAAACAGCAAGGGCAGCCCCTGTACAAGGACAACTGGCTCCTAAGGGAAGGACTAAAT 160
Cavia   CCCATGTCATTGCCCGAAGACACAAACAACAAGGGCAACCTCTGTACAAGGACAACTGGCTCCTAAGGGAAGGATTAAAC 160
Homo    CTCACGTCATTGCCCGAAGACACAAACAACAAGGGCAGCCCCTATACAAGGACAGTTGGCTCCTAAGGGAGGGACTAAAT 160
```

ENSMUSG00000015971 exon 3 (ORF 0)

```
Mus     AAACCTGAAAGTAATGAACAAAGACAAAATGGCCTTAAAATGGTGGACCAGGCAATATGGTCTAAAAAGATGTCAAACGG 80
Rattus  AAACCTGAAAGTAATGAACAAAGACAAAATGGCCTTAAAATGGTGGACCAGGCAATATGGTCTAAAAAAATGTCAAACGG 80
Cavia   AAACCTGAGAGTAACGAACAAAGACAAAATGGCCTTAAAATGGTGGATCAAGCAATATGGTCTAAAAAGATGTCAAATGG 80
Homo    AAACCAGAAAGTAATGAACAAAGACAAAATGGCCTTAAAATGGTGGATCAAGCAATATGGTCTAAAAAGATGTCCAATGG 80

Mus     TACAAGGCGGATCCCTGTGTCACCAGAACAG 111
Rattus  TACAAGGCGGATCCCTGTGTCACCAGAACAG 111
Cavia   TACAAGACGGATTCCTGTGTCCCCAGAACAG 111
Homo    TACAAGACGCATTCCTGTGTCCCCTGAACAG 111
```

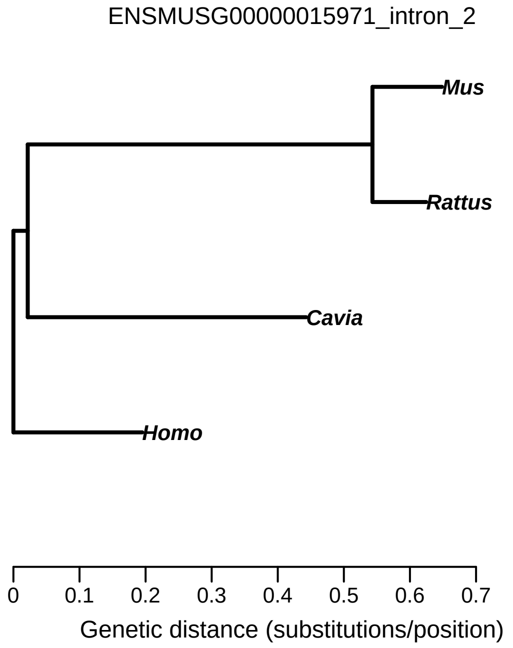

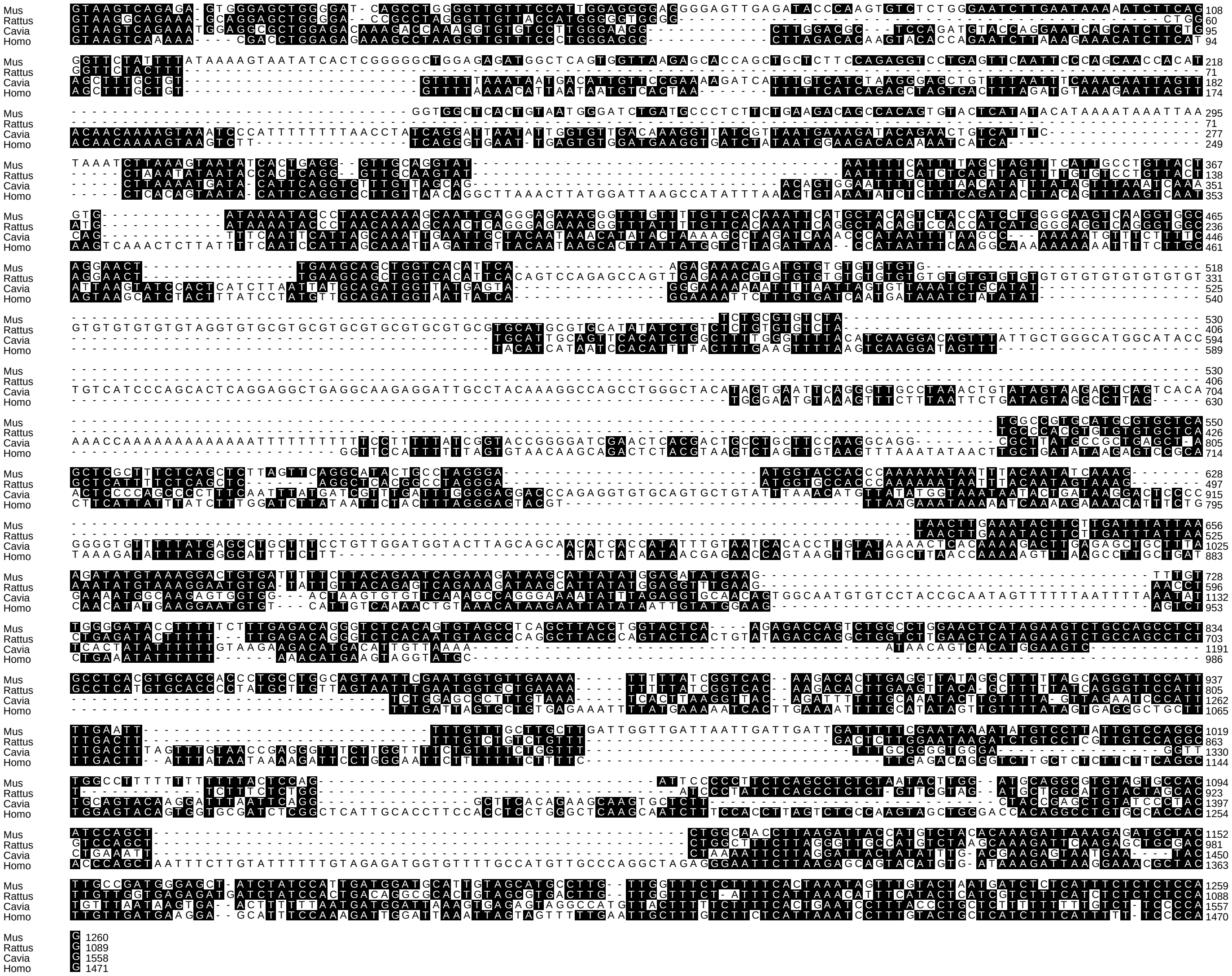
Mus
Rattus
Cavia
Homo

ENSMUSG00000030872 intron 10

Description: ADP-ribosylation factor-binding protein GGA2 (Gga2)
Intron number: 10
Mouse chromosome: 7
Upstream exon length: 126
Downstream exon length: 90
Mouse intron length: 705
Intron alignment length: 1237
Total murinae branch length: 0.17685
K_score: 0.06964
Scaling factor: 0.66861

ENSMUSG00000030872 exon 10 (ORF 2)

```
Mus     CGGAGATTCTTCAAGCAAATGACCTTCTCACACAGGGGGTTCGGCTGTACAAACAGGTGGTGGAGGGCCGAGTTAGTGCT 80
Rattus  CGGAGATTCTTCAAGCAAATGACCTTCTCACCCAAGGGGTTCTGCTGTACAAACAGGTGGTGGAAGGCCGAGTCAATGCC 80
Cavia   CCGAGATTCTCCAGGCAAATGACCTCCTTACTCAGGGAGTTCTGCTGTACAAACAGGTCATGGAGGGTCGTGTCACCTTT 80
Homo    CGGAAATTCTCCAGGCAAATGACCTCCTCACCCAAGGAGTTCTGCTGTACAAACAGGTGATGGAGGGCCGGGTCACCTTT 80

Mus     GGGAACGCAGTGCCTGCCGCAGTGGGAGCCATTCCCGCCCCCAGAG 126
Rattus  GGGAATGCAGTGCCCGCCGCAGTGGGAGCCATTCCTGCTTCCAGAG 126
Cavia   GGGAGCACAGTGACCAGCTCAGTGAAAGACACACCTGTCTCCAGAG 126
Homo    GGAAACAGAGTGACCAGCTCATTGGGAGACATCCCTGTCTCCAGAG 126
```

ENSMUSG00000030872 exon 11 (ORF 2)

```
Mus     CATTCCCGAATCCAGAACCCTGTGGGTTGAACTGTCCACTCATTGACCTGGA---------------------------- 52
Rattus  TGTTCCCGAGTCCAGAACCCTGTGGGAAGAACTGCCCCCTCATTGACCTGGA---------------------------- 52
Cavia   TCTTCCAGAATCCAGCAGACAGCATGAAGAATTGCCCCTTGATTGACCTGGAGGTGGACAGTGGCCCTGAGCAGGCTGGG 80
Homo    TCTTTCAGAATCCAGCAGGCTGCATGAAGACCTGCCCCCTGATTGACTTGGAGGTGGACAATGGACCTGCGCAGATGGGG 80

Mus     -----GACGCCATCTTTGCTTCATCAGGACCTGGCAGCCTTAG 90
Rattus  -----GGCACCATCTTTGCTTCATCAGGACCTGGCGGCCTTAG 90
Cavia   TCTGTGGCACCATCTTTGCTTCATCAGGACTTGGCAGCCTTAG 123
Homo    ACTGTGGTGCCATCTTTGCTTCATCAGGACCTGGCAGCCTTGG 123
```

ENSMUSG00000030872_intron_10

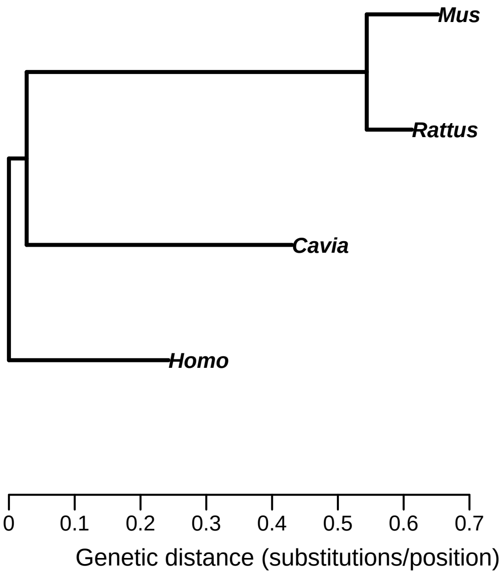

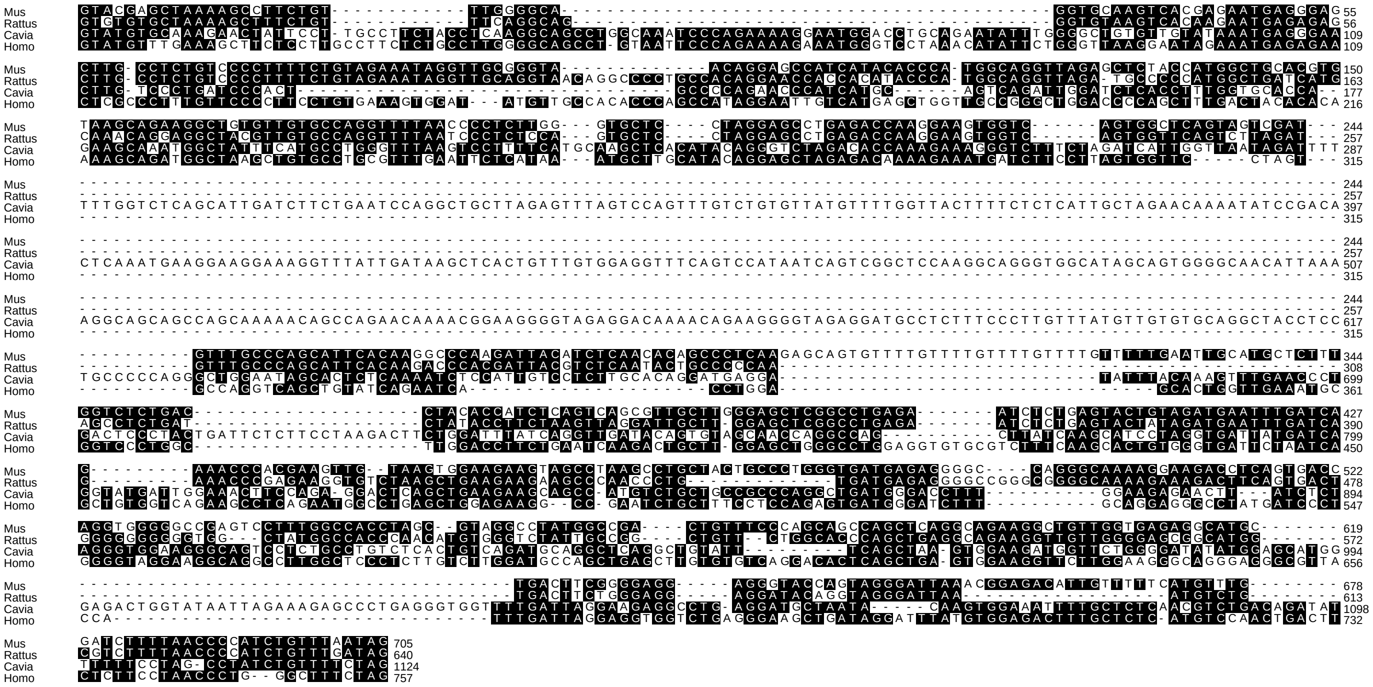
Mus
Rattus
Cavia
Homo

ENSMUSG00000030374 intron 16

Description: Striatin-4 (Strn4)
Intron number: 16
Mouse chromosome: 7
Upstream exon length: 87
Downstream exon length: 170
Mouse intron length: 1091
Intron alignment length: 1532
Total murinae branch length: 0.17999
K_score: 0.06173
Scaling factor: 0.67261

ENSMUSG00000030374 exon 16 (ORF 2)

```
Mus     GTAAATCTGTGCATTCCATGGTGGCCCACCTGGATGCAGTCACCTGCCTAGCTGTGGACCCAAATGGCGTGTTCTTGATG 80
Rattus  GTAAATCCGTGCATTCCATGGTGGCCCACCTGGACGCAGTCACCTGCCTAGCTGTGGACCCAAATGGCGTGTTCTTGATG 80
Cavia   GTAAATCGGTGCACTCCATGGTTGCCCACCTGGATGCAGTCACCTGCCTAGCTGTGGACCCCAATGGCGTGTTCCTAATG 80
Homo    GTAAGCCGGTGCACTCCATGGTTGCACACCTGGACGCAGTCACCTGCCTAGCCGTGGACCCCAACGGCGCATTCCTGATG 80

Mus     TCAGGAA 87
Rattus  TCAGGAA 87
Cavia   TCAGGAA 87
Homo    TCAGGAA 87
```

ENSMUSG00000030374 exon 17 (ORF 2)

```
Mus     GCCACGACTGTTCTCTGCGTTTATGGAGCCTAGACAACAAGACATGTGTGCAGGAGATCACGGCCCACCGCAAGAAGCAT 80
Rattus  GCCATGACTGTTCTCTGCGTTTGTGGAGCCTAGACAATAAGACATGTGTGCAGGAGATCACGGCCCACCGGAAGAAGCAC 80
Cavia   GCCATGACTGCTCCCTGCGCCTGTGGAGCCTGGACAACAAGACCTGCGTACAGGAGATCACAGCCCATCGCAAGAAGCAT 80
Homo    GCCATGACTGCTCCCTGCGTCTCTGGAGCCTGGACAACAAAACGTGCGTGCAGGAGATCACGGCCCACCGCAAGAAGCAC 80

Mus     GAGGAGGCCATCCATGCAGTGGCCTGCCATCCCAGCAAGGCACTTATTGCCAGTGCTGGTGCTGATGCCCTAGCCAAGGT 160
Rattus  GAAGAGGCCATCCATGCGGTGGCCTGCCATCCCAGCAAGGCACTCATTGCCAGTGCTGGTGCTGATGCCCTAGCCAAGGT 160
Cavia   GAGGAGGCCATCCATGCTGTCGCCTGCCACCCCAGCAAGGCCCTTATTGCCAGCGCTGGTGCCGACGCCCTGGCCAAGGT 160
Homo    GAGGAGGCCATCCACGCTGTTGCCTGCCACCCCAGCAAGGCCCTCATTGCCAGTGCTGGCGCTGATGCCCTGGCCAAGGT 160
```

ENSMUSG00000030374_intron_16

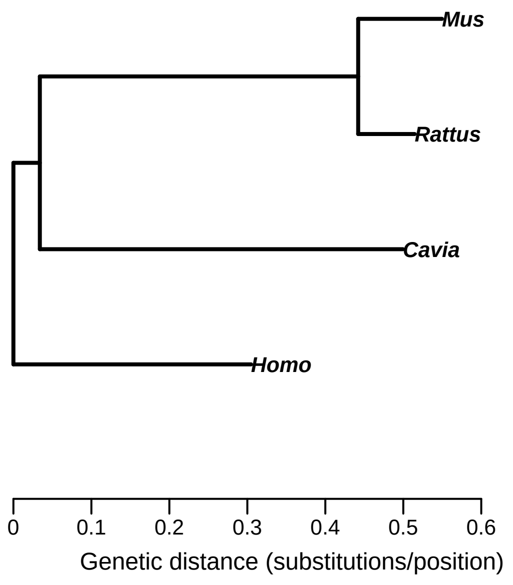

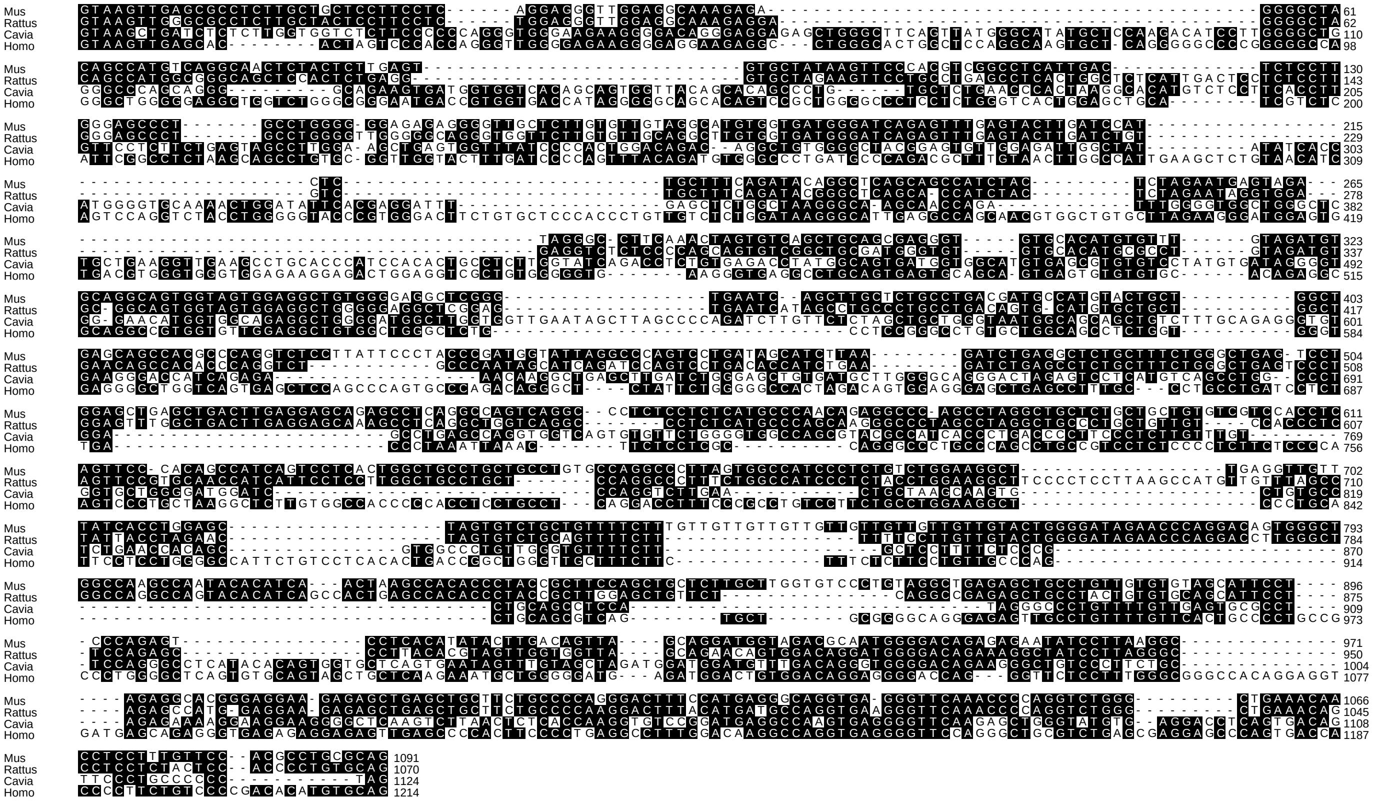
Mus
Rattus
Cavia
Homo
1091
1070
1124
1214

ENSMUSG00000027340 intron 14

Description: Solute carrier family 23 member 2 (Slc23a2)
Intron number: 14
Mouse chromosome: 2
Upstream exon length: 96
Downstream exon length: 233
Mouse intron length: 1298
Intron alignment length: 2615
Total murinae branch length: 0.17163
K_score: 0.07946
Scaling factor: 0.67333

ENSMUSG00000027340 exon 14 (ORF 2)

```
Mus     GAATAACAGGAATCGACCAAATCCTGAATGTCCTTCTCACCACCGCTATGTTTGTAGGAGGCTGTGTGGCTTTCATTTTG 80
Rattus  GCATAACAGGCATTGATCAAGTTCTGAATGTCCTTCTCACCACTGCTATGTTCGTCGGAGGCTGTGTGGCTTTTATTTTG 80
Cavia   GCATAACAGGAATTGATCAAGTCTTAAACGTTCTTCTCACCACTGCTATGTTTGTAGGAGGCTGTGTGGCTTTTATTTTG 80
Homo    GGATAACAGGAATCGATCAAGTGTTGAACGTCCTTCTCACAACTGCTATGTTTGTAGGGGGCTGTGTGGCTTTTATCCTG 80

Mus     GACAACACCATCCCAG 96
Rattus  GACAACACCATCCCAG 96
Cavia   GACAACACCATCCCAG 96
Homo    GATAACACCATCCCAG 96
```

ENSMUSG00000027340 exon 15 (ORF 2)

```
Mus     GTACCCCAGAGGAAAGAGGAATCAAGAAATGGAAGAAGGGTGTGAGCAAAGGAAGCAAGTCTCTGGACGGCATGGAGTCC 80
Rattus  GAACCCCAGAGGAAAGAGGAATCAAGAAATGGAAGAAGGGTGTGAGCAAAGGAAACAAGTCTCTTGACGGCATGGAGTCC 80
Cavia   GTACCCCAGAGGAAAGAGGGATCAGGAAATGGAAGAAAGGTGTGAGCAAAGGGAACAAGTCGCTTGATGGCATGGAATCC 80
Homo    GCACTCCAGAGGAAAGAGGAATCCGGAAATGGAAGAAGGGTGTGGGCAAAGGGAACAAATCACTCGACGGCATGGAGTCG 80

Mus     TACAATCTGCCATTTGGCATGAACATTATTAAAAAGTACCGATGCTTCAGCTACCTGCCCATCAGCCCCACCTTTGCAGG 160
Rattus  TATAATTTGCCATTTGGCATGAACATTATTAAAAAATACAGATGCTTCAGCTACCTGCCTATCAGCCCAACCTTTGCAGG 160
Cavia   TACAACTTGCCGTTCGGCATGAACATTATTAAAAAGTACAGATGCTTCAGCTACCTGCCCATCAGCCCAACCTTTGCAGG 160
Homo    TACAATTTGCCATTTGGCATGAACATTATAAAAAAATACAGATGCTTCAGCTACTTACCCATCAGCCCAACCTTTGTGGG 160
```

ENSMUSG00000027340_intron_14

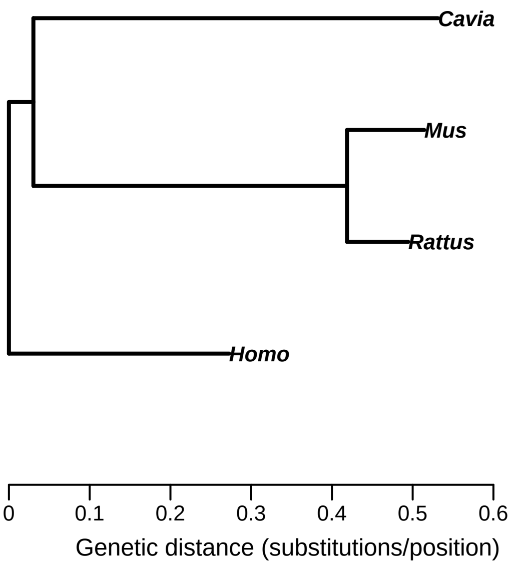

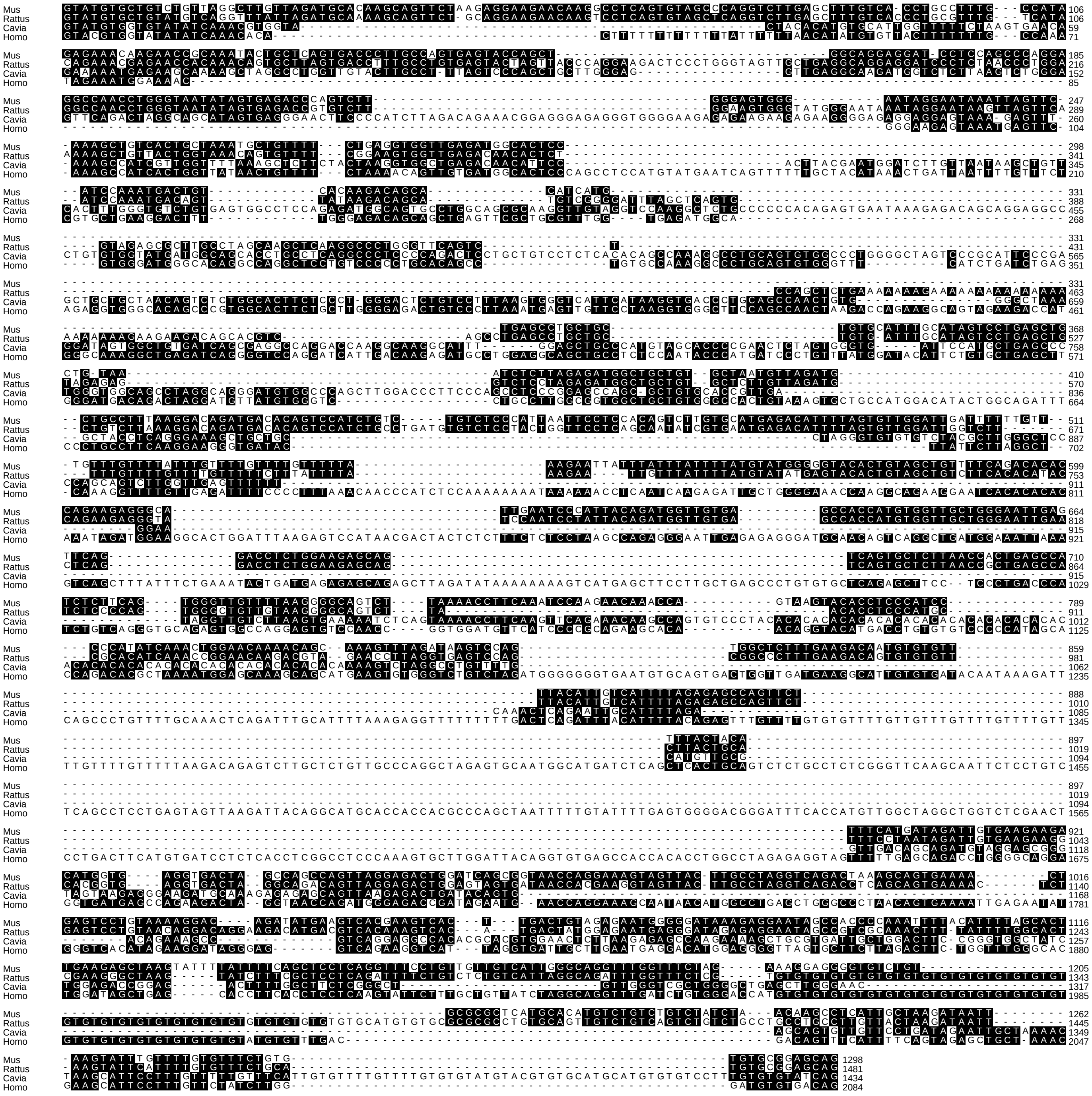

ENSMUSG00000030653 intron 27

Description: cGMP-dependent 3’,5’-cyclic phosphodiesterase (Pde2a)
Intron number: 27
Mouse chromosome: 7
Upstream exon length: 100
Downstream exon length: 113
Mouse intron length: 271
Intron alignment length: 299
Total murinae branch length: 0.15961
K_score: 0.08321
Scaling factor: 0.67428

ENSMUSG00000030653 exon 27 (ORF 0)

```
Mus     GACTATCAGCGCATGCTGGACCTGATGAGGGACATCATCTTGGCTACAGACCTGGCACACCACCTCCGCATCTTCAAGGA 80
Rattus  GACTATCAGCGAATGCTGGACCTGATGAGGGACATCATCTTGGCCACGGACCTGGCACACCACCTCCGCATCTTCAAGGA 80
Cavia   GACTATCAGCGCATGCTAGATCTGATGAGGGACATCATCTTGGCCACGGACCTGGCCCACCACCTCCGTATCTTCAAGGA 80
Homo    GACTATCAGCGCATGCTGGATCTGATGCGGGACATCATCTTGGCCACAGACCTGGCCCACCATCTCCGCATCTTCAAGGA 80

Mus     CCTGCAGAAGATGGCTGAAG 100
Rattus  CCTGCAGAAGATGGCTGAAG 100
Cavia   CCTCCAGAAGATGGCTGAAG 100
Homo    CCTCCAGAAGATGGCTGAGG 100
```

ENSMUSG00000030653 exon 28 (ORF 2)

```
Mus     TGGGTTATGACCGAAACAACAGGCAACACCACAGGCTTCTTCTGTGCCTCCTCATGACCTCCTGTGACCTCTCTGACCAG 80
Rattus  TGGGTTATGACCGAAACAACAAGCAACACCACAGGCTTCTTCTATGCCTCCTTATGACCTCCTGTGACCTCTCTGACCAG 80
Cavia   TGGGCTATGACCCAACCAACAAGCAGCACCACAGACTCCTCCTTTGCCTCCTCATGACCTCCTGTGACCTCTCTGACCAG 80
Homo    TGGGCTACGACCGAAACAACAAGCAGCACCACAGACTTCTCCTCTGCCTCCTCATGACCTCCTGTGACCTCTCTGACCAG 80

Mus     ACAAAGGGCTGGAAGACCACCAGAAAGATTGCA 113
Rattus  ACAAAGGGCTGGAAGACCACCAGAAAGATTGCG 113
Cavia   ACCAAGGGCTGGAAGACCACCAGAAAGATTGCT 113
Homo    ACCAAGGGCTGGAAGACTACGAGAAAGATCGCG 113
```

ENSMUSG00000030653_intron_27

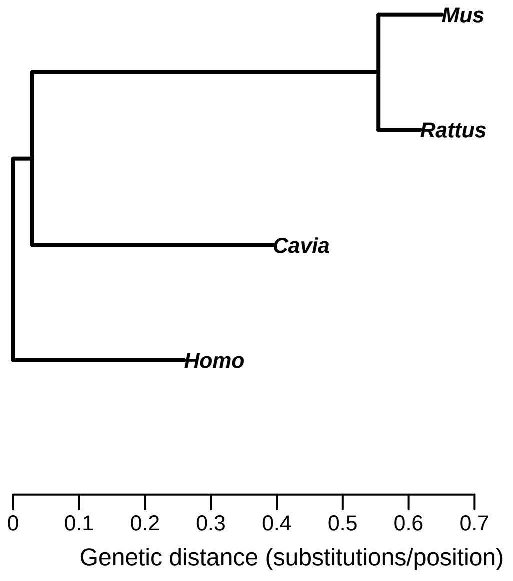

```
Mus     GTG-------------CCGCA-CACATTGCTGGGGAGAGATGGGTGGTTTTGGTTTAACATGGAGATCTGCCCACCTGAACATCTGAAGTGGCTTTGTCCTGTCCTT--- 93
Rattus  GTG-------------CCACAGCTCACTGCTGGGGAGGGATGGG------TGGTTTAGCATGGAGATCTGAACATCTGGACATCTGGAGTGCTTCTGTCCTGTCCTT--- 88
Cavia   GTGCCCACCCTTAGCCCCAGC-CTTCTGGTTGGAGAGGGAGGG--------------TCTTCAGGATCTGCCCG-CGGTGCATACTCTGTACTTCAG------------- 81
Homo    GTGACTGCTGTTAGCCCCAGTCCTTGGGGCTGGGGAGGAACAA-----------CCAGGGGAAGGATTTGCCAG-GGGAGCATTCCCAGGGTGCAGACCCATCCCCTGCA 98

Mus     -----GGCCCATTCCTGGCTATG-GTGTCCCTGCAGGCAGACCTACTACTGGTCACCT----TCCATTGGGTGTGCTTTGTTCCCATGGGATGCTGTAGACCCACTTTGG 193
Rattus  -----AGCCCCTTCCTGGCTATG-GTGTCCCTCCAGGCAGACCTAGTACTGGCCATCT----TCCATTGCATG-----GTTCCCCATGGGATGCTGTAGACCTACTTCAG 183
Cavia   -----ATTCTTCCCCTTCCAGTATCAGCCCCTCCAGGCAGA-CCATCATCAGCCCCTGGA--CCTAACAGGACTGTCCAGTCCCC------------TGGGCAACTC--- 168
Homo    ACATCAACCCTTCTCTGGCTGCACGGCCCCCCCCAGGCAGACCCAGCACTGGCCCCTTGGCTCCCATCAAGGGTGCCCAATTCCC------------TGGACCGCTCTGG 196

Mus     GACAGGCTGTGGGGTCCTCGTTCTCAGAAGGGACAAGGGACCTGGGCCCTCCTGCTTGACTCCATTGTCCCCC-AACAG 271
Rattus  GACGGGCTGCGGGGCCCTTGTTCTCAGAAGGGACAAGGAACCTGGGTCCTCCTGCTTGACTCCGTTGTCCCCC-AACAG 261
Cavia   ------------AGGCCTTGTCCTCAGAGGGGA-AAGGAGGCTGGGTCCCTCTCCTTGACCCCATCATTTCCCCAACAG 234
Homo    GTTGGGCCCTGGGAGCCTTGTCCTCAGAAGGGC-AAAGAGGCTGGGCCCCGCTCCTTGACCCCATCCTCCCCTCAACAG 274
```

ENSMUSG00000034575 intron 1

Description: DNA polymerase sigma (Papd7)
Intron number: 1
Mouse chromosome: 13
Upstream exon length: 90
Downstream exon length: 47
Mouse intron length: 768
Intron alignment length: 1223
Total murinae branch length: 0.16595
K_score: 0.05011
Scaling factor: 0.67578

ENSMUSG00000034575 exon 1 (ORF 0)

```
Mus     ATGTCCCCTTGTCCTGAAGAAGCAGCCATGAGAAGGGAGGTGGTGAAACGGATCGAAACTGTGGTGAAAGACCTCTGGCC 80
Rattus  ATGTCCCCTTGTCCTGAAGAAGCAGCCATGAGAAGGGAGGTGGTGAAGCGCATCGAAACTGTGGTGAAAGACCTCTGGCC 80
Cavia   ATGTCCCCCTGTCCTGAGGAAGCAGCCATGAGGAGGGAGGTGGTGACGCGGATCGAGACCGTGGTGAAGGACCTGTGGCC 80
Homo    ATGTCCCCTTGTCCTGAAGAAGCAGCTATGAGAAGAGAGGTGGTGAAACGGATCGAAACTGTGGTGAAAGACCTTTGGCC 80

Mus     CACAGCTGAT 90
Rattus  CTCGGCTGAC 90
Cavia   TACGGCTGAC 90
Homo    GACGGCTGAT 90
```

ENSMUSG00000034575 exon 2 (ORF 0)

```
Mus     GTGCAGATATTTGGCAGCTTTAGTACAGGCCTCTATCTTCCAACAAG 47
Rattus  GTGCAGATATTTGGCAGCTTTAGCACAGGCCTCTATCTTCCAACAAG 47
Cavia   GTGCAGATATTCGGCAGCTTCAGCACAGGTCTCTACCTTCCCACCAG 47
Homo    GTACAGATATTTGGCAGCTTTAGTACAGGTCTTTATCTTCCAACTAG 47
```

ENSMUSG00000034575_intron_1

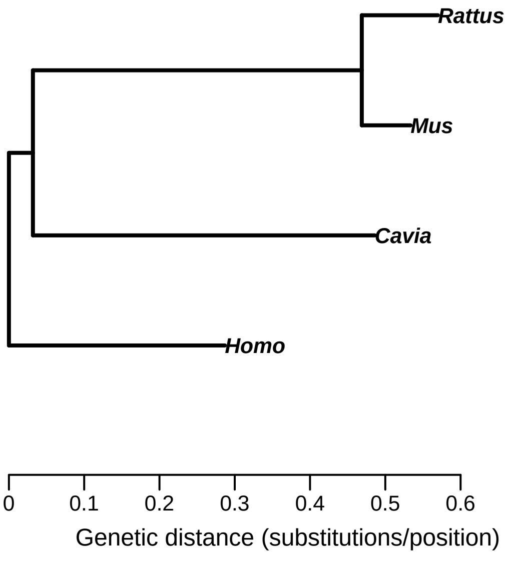

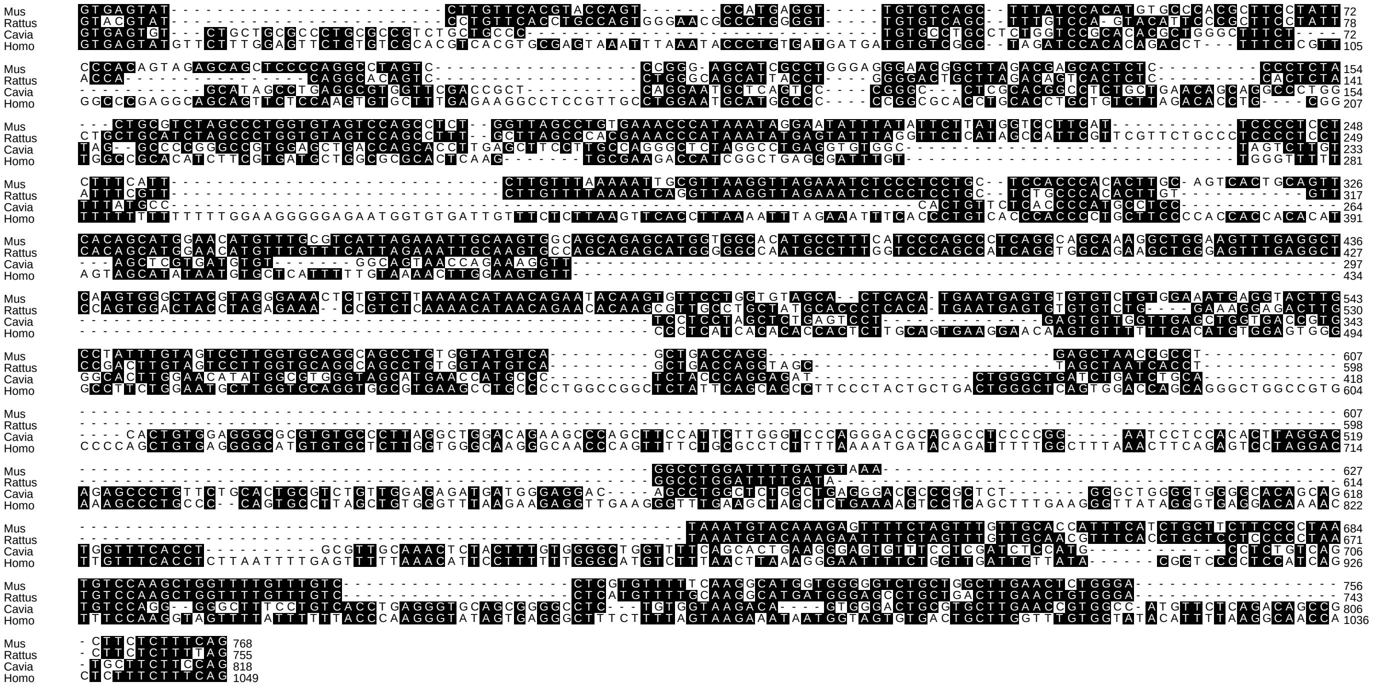
Mus
Rattus
Cavia
Homo

ENSMUSG00000051768 intron 14

Description: DNA repair protein XRCC1 (Xrcc1)
Intron number: 14
Mouse chromosome: 7
Upstream exon length: 137
Downstream exon length: 91
Mouse intron length: 1057
Intron alignment length: 2202
Total murinae branch length: 0.17517
K_score: 0.06743
Scaling factor: 0.67933

ENSMUSG00000051768 exon 14 (ORF 1)

```
Mus     GGTGGCCAAACAGAGGGAACAAAGGCAGCCCCCAGCCCCAGAGGAGAATGGCGAGGACCCGTATGCAGGCTCCACAGATG 80
Rattus  GGTGGCCAAGCAGAGGGAGCAAAGGCAGCCCCCAGCCCCAGAGGAGAATGGCGAGGACCCGTATGCAGGCTCCACAGATG 80
Cavia   GGTGGCCGAGCAGAGGGAGCACCAGGCCCGCCCCTGGCC---AGGAGAACGGTGAGGACCCGTATGCAGGCTCCACAGATG 77
Homo    GGTGGCAGAGCAGAAGGAACACCAGACTGCCCCCTGGCCAGGAGGAGAATGGGGAAGACCCGTATGCAGGCTCCACGGATG 80

Mus     AGAACACAGACAGTGAGACCCCCTCAGAGGCT---GACCTGCCAATCCCAGAGCTCCCAG 137
Rattus  AGAACACAGACAGCGAGGCTCCCTCAGAGGCT---GACCTGCCAATTCCAGAGCTCCCAG 137
Cavia   AGAACACCGACAACGAGGATCGCCCAGAGTCTCCCGACCAGCCAATCCCTGAGCTCCCAG 137
Homo    AGAACACGGACAGTGAGGAACACCAGGAGCCTCCTGATCTGCCAGTCCCTGAGCTCCCAG 140
```

ENSMUSG00000051768 exon 15 (ORF 2)

```
Mus     ACTTCTTCGAGGGCAAACACTTCTTCCTGTATGGCGAGTTCCCTGGGGATGAGAGGAGGAGGCTCATCCGCTACGTGACC 80
Rattus  ACTTCTTCCAGGGCAAGCACTTCTTCCTGTACGGCGAGTTCCCTGGGGATGAGAGGAGGAAGCTCATCCGATATGTGACA 80
Cavia   ACTTCTTCCAGGGCAAGCACTTCTTCCTGTATGGGGAGTTCCCTGGTGACGAGCGGCGGAAGCTCATCCGTTATGTGACA 80
Homo    ATTTCTTCCAGGGCAAGCACTTCTTTCTTTACGGGGAGTTCCCTGGGGACGAGCGGCGGAAACTCATCCGATACGTCACA 80

Mus     GCTTTCAATGG 91
Rattus  GCTTTCAATGG 91
Cavia   GCCTTCAATGG 91
Homo    GCCTTCAATGG 91
```

ENSMUSG00000051768_intron_14

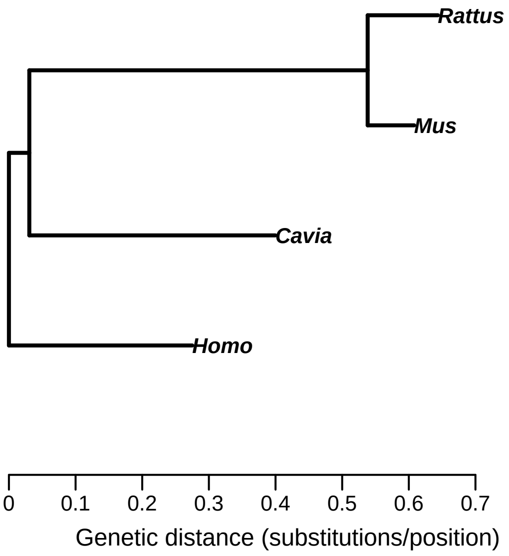

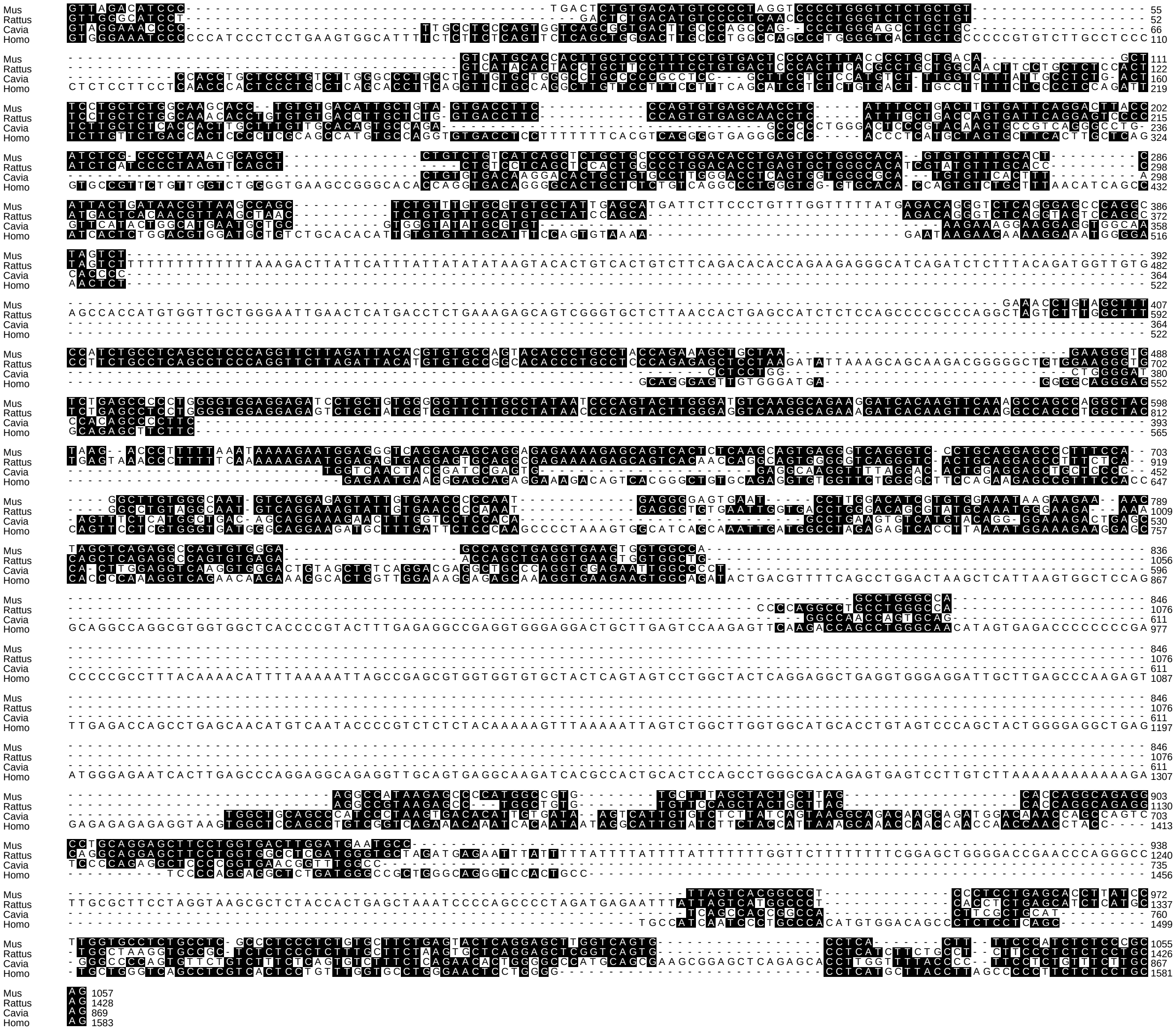

ENSMUSG00000036244 intron 7

Description: TBC1 domain family, member 21 (Tbc1d21)
Intron number: 7
Mouse chromosome: 9
Upstream exon length: 97
Downstream exon length: 101
Mouse intron length: 252
Intron alignment length: 347
Total murinae branch length: 0.17005
K_score: 0.05567
Scaling factor: 0.67976

ENSMUSG00000036244 exon 7 (ORF 0)

```
Mus     GAGCACAGCTGTGTCATCAACATCGGTGTAGGCAAGAATCTGGACATGCTCAACAGCCTGATCACTCTACTGGACCCTGA 80
Rattus  GAGCACAGTTGTGTCATCAACATCGGTGTAGGCAAGAACCTGGACATGCTCAACAACCTGATCACTTTACTGGACCCTCA 80
Cavia   GAGAACAGCTGTGTCATCACCATTGGGGTGGACAAGAACCTGGACATGCTCAACCACCTCATCGCCCTCCTGGACCCCAC 80
Homo    GAACACAGCTGTGTCATCAACATTGGCGTGGCCAAGAACCTAGACATGCTCAGCACCCTGATCACCTTCCTGGACCCCGT 80

Mus     ATTTGCTGAACACCTCA 97
Rattus  GTTTGCTGACCACCTCA 97
Cavia   ATTTGCTGAGCACCTGA 97
Homo    GTTTGCTGAGCACCTAA 97
```

ENSMUSG00000036244 exon 8 (ORF 2)

```
Mus     AAGGGAAGGGCTCAGGGGCTGTGCAGTCCCTCTTCCCGTGGTTCTGCCTTTGCTTCCAGCGTGCCTTCAAAACCTTCGAT 80
Rattus  AAGGAAAGGGCTCGGGAGCTGTGCAGTCCCTCTTCCCATGGTTCTGCCTTTGCTTCCAGCGTGCCTTCAAATCCTTCGAT 80
Cavia   AAGGGAAGGGCGCGGGGACAGTGCAGGCCCTCTTCCCTTGGTTCTGCCTCTGCTTCCAGCGTGCCTTCAAAACCTTTGAT 80
Homo    AAGGGAAGGGTGCAGGGGCTGTGCAGTCCCTCTTCCCCTGGTTCTGCTTCTGCTTCCAGCGTGCCTTCAAGTCCTTCGAT 80

Mus     GATGTCTGGAGGCTCTGGGAG 101
Rattus  GATGTCTGGAGGCTCTGGGAG 101
Cavia   GACGTCTGGCGGCTCTGGGAG 101
Homo    GATGTCTGGAGGCTCTGGGAG 101
```

ENSMUSG00000036244_intron_7

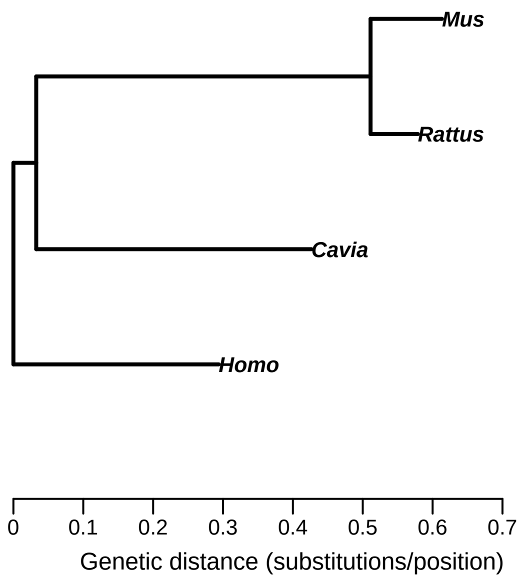

```
Mus     GTGAGTGGTCCACAGTTACTATTACTTCTCACACCAGCAAG-----------------------------------TAGGCTGTGGAAGGGTCCCGACCTAGC------- 68
Rattus  GTGAGTGGTCCACAGTTACTATTACTATTCACACCAGCAGG-----------------------------------TAGGCCATGGGAGGGCCAGGGCCCAGC------- 68
Cavia   GTGCGTGGACTCCATCTCCTGACCCCTAAGGCCCTGGCCAGACAAGGATGGGGGAGGGGG-------CAGCTGTCCTTAGTCACAGGAAGA----GGGGCAGT------- 92
Homo    GTGAGTGGTTCCCACCTCCTGCCACCTCACGCACCCCCCAGGCATGGAAGGGGGCTGGGACCCAACTGTCAGTCCCTAATCATGGGGGGGGCTGGAGAGCAGCAGAATCA 110

Mus     CTGACAGACATATTCAGG-----AGGGGAGGCCCAACGTGAAGGAGCCTGGCTGGGTCTGTGATGCTAAACCTAAGCTCCAAATGGACTCCATGGCTTGTTGAGAAAGTA 173
Rattus  CTAGCAGATATCCTCTGG-----AGGGGAGGAGCAACACGGAGGAGC----CTGGGTCTGTGGTGCTAAACCTATGCTCCAAATGGACTCCATGGCTTGTTGGGAAAGTA 169
Cavia   GCTGGGGGCCTGTGCAGGCACTAGGAGGAG------------AGGGC----TGGGGTCTGCACTGGTGACCACAGCCCC-----AGGCTCCAGGGACAGTTG-CACAGTC 180
Homo    TTTGATTGCTTCTCCAGGCCCTGGGAAGAGGGTTATCTGGATGGGGT----TGGAGCCTGGACAGTTGAAAGTTGACTCTAAACCATCTCCCAGGGCAGCTG-GAAAAGG 215

Mus     GCAGGTGACATTCTCATGTTTCTTATGTCTCCCAT--GCTCTGTCATGTGGTGGCTTAAGCA----------------------------------------------CC 235
Rattus  GCAGGTGAAATTTTCATGTTTCCTATGTCTGCTGT--GCTCCGTTGTGTGGTGGCTTAAGCA----------------------------------------------CC 231
Cavia   GCAGCCAGAGTTACCTCATGTCCTAAGTGTCCCGCAGGCTCTGTGCATAGGCTTTGTTGG-----------------GTGTGTAGTCTTCCCAGGCCCCCAACATTGGCC 273
Homo    GGCGGCAGGCTTTTTGTATTTCCCAAGTGTTTTGCAGGCTCTGTAGTTTGGCCTCATCTGCATGTGTTTGCTGCATCATATGTGTTTTCCCCTGACCACAGCC-----TC 320

Mus     TGCTTTTCTTCCCACAG 252
Rattus  TGCTTCTCTCCCAACAG 248
Cavia   TGCTTCTCTCCTCACAG 290
Homo    ACCTTCTCTCCCCACAG 337
```

ENSMUSG00000028173 intron 7

Description: Integral membrane protein GPR177 Precursor (Wls)
Intron number: 7
Mouse chromosome: 3
Upstream exon length: 98
Downstream exon length: 64
Mouse intron length: 364
Intron alignment length: 475
Total murinae branch length: 0.12969
K_score: 0.087
Scaling factor: 0.68014

ENSMUSG00000028173 exon 7 (ORF 0)

```
Mus     GATCAACATGAACGGAATCACATTGCAGGGTACTGGAAGCAAGTTGGACCAATTGCTGTTGGCTCCTTCTGCCTCTTCAT 80
Rattus  GATCAACATGAGCGGAATCACATTGCAGGGTATTGGAAGCAAGTTGGACCAATTGCTGTTGGCTCTTTCTGCCTCTTCAT 80
Cavia   GATCAGCACGAGCGGAACCACATCGCCGGCTACTGGAAGCAGGTTGGCCCAATCGCTGTTGGCTCCTTCTGCCTCTTCGT 80
Homo    GATCAGCACGAGCGGAACCACATCGCAGGGTATTGGAAGCAAGTCGGACCCATTGCCGTTGGCTCCTTCTGCCTCTTCAT 80

Mus     ATTTGACATGTGTGAAAG 98
Rattus  ATTTGACATGTGTGAGAG 98
Cavia   ATTTGACATGTGTGAGAG 98
Homo    ATTTGACATGTGTGAGAG 98
```

ENSMUSG00000028173 exon 8 (ORF 1)

```
Mus     AGGAGTACAACTCACAAATCCTTTCTACAGTATCTGGACTACAGATGTTGGAACAGAACTGGCT 64
Rattus  AGGAGTGCAACTCACAAATCCTTTCTACAGTATCTGGACTACAGATGTTGGAACAGAACTGGCT 64
Cavia   AGGCGTGCAGCTCACCAACCCTTTCTACAGTATCTGGACCACGGACGTTGGCACGGAGCTGGCT 64
Homo    AGGGGTACAACTCACGAATCCCTTCTACAGTATCTGGACTACAGACATTGGAACAGAGCTGGCC 64
```

ENSMUSG00000028173_intron_7

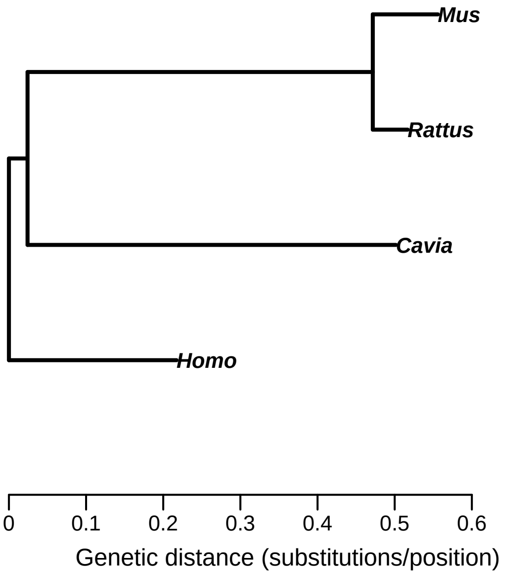

```
Mus      GTGAGCAGATATCAGCATCACTCCAGGATGAAATGTACCTTTCTATGTCTACTTCTATCTTTCTAGGAAGAAT-TTTGAAATGAAGCATGGTGGTTGGGGATTCATGGGA 109
Rattus   GTGAGCAGGTACCGGAGTCATTCCAGGATGAAATGTGCCTTTCTATGTCTACTTCTATCTTTCTAGGAAGAAT-TTTAAAATGAAGCATGGTGGTTGGGGATCCATGGGA 109
Cavia    GTGAGCAGGTGCCCCAG--GCCTCAGCACAAAAGGTGCATGAC-----CTCCCCCAATCTCTTCCAAGGGTCAGTTTGAGAA-------GGTGGTTGGGAGCCTTTAG-- 94
Homo     GTGAGCAAGTGTTTGTGGGACACTGAGCCAAACTGTGCATCACCACGCCCACTCCTGTCATTTTAGGAGGAGTGTTTGAAAAGGAGAATGGTGGTCGGGAACCTTTGGTT 110

Mus      TGGAA-TATTGGAGAGGTCAATATCCACAGATGACATATGGGACTAAGTCAAAGGTTTAGTCCTTTAT------------------------------------------ 176
Rattus   TGGAA-TATTGGAGAGGTCAACATCTTCAGATGAAGC-TGGTACTAAGTCAAAGGTTTAGTCCTTTAT------------------------------------------ 175
Cavia    -GAAGTCACTGGGTGTGGTCATGTCTTCAGGTCCAGACTGGGCTTCAGTCCAAGCTGTGGGCCTTTATTTGTTTTTTCCCAGCACTGGGGATCAACCCAGGTCCTGTTGC 203
Homo     TGGAAGCACTGGACGTGGTCACATCCTCAGACAAGGTCTGGGATCAAATCAAAGGCACAGCTCTTTAC------------------------------------------ 178

Mus      ----------------------------ATATGCAAAGTGCTTG------CCATATTCTAAAGCTCTGCATCTCCTTA----TACTCTCTGAAGTTTATTTTCTAAATAT 248
Rattus   ----------------------------ACATGCAAAGCACTTGGCCTCTTCATATTTTAAAGTTCTGCATC-CCTTA----TACTCTGTAAAGTTTATTTTCTAAATAA 252
Cavia    AGCCCCAGGTCCATGCATAGCCCCTTAGAGTTGCAAAACTTCTGGGCCATCAAGCTCTCTCCAGCTTGTTTCACTGTGGATTTGTTCTCTGTGATGCTG---CTGGTCAG 310
Homo     ----------------------------AGTTGAAAAATATTTGGGCCGATGTATTCTACAAGCTTCATTTCTCTTTA----GGATTTCTGTAATGTTTT--TTAGCCTA 254

Mus      AGTACACTCACACTCCTCCCATACTCACCATGTCTCACTTCATTGTTGCAACACTAAGAGAGGCT------------CTGTTCTGCCTTTGTGTTA-----------TCT 335
Rattus   AGTATACTCCTACCCCTACCACATTCCCC--GTCTCACTCCACTGTTGCAATGTTAAGAGAAGCT------------CTGTTCCGCCTTTGTGTTA-----------TCT 337
Cavia    GGCACAGCTCCTCCTCCCCCA-----------------GCTAGAGATGGAAAGCAAACAAAGTCCAG----------CTGCTTCGCCTTAGCAGCAGTTACACCCCGGCT 393
Homo     AATACAGCCCTCACCCCTGCA-----------------------GCTGAAAAGCTAAGAATATGTGAAAAGGAAAGTTCCCGTTGCCTTTGTATTAATTGTGTCTTGCCT 341

Mus      CTTCATCTTG------TTTGGTTTTGTTTTTATAG 364
Rattus   CTTCATCTTG------TTTGTTTTTGTTTTTATAG 366
Cavia    TTCCCTCTTGGTTCTTTTCCCTTTTTTCTCTGTAG 428
Homo     TTCCATCTCA-------------TTTTCTTTGTAG 363
```

ENSMUSG00000024187 intron 4

Description: Protein ITFG3 (Itfg3)
Intron number: 4
Mouse chromosome: 17
Upstream exon length: 137
Downstream exon length: 133
Mouse intron length: 602
Intron alignment length: 667
Total murinae branch length: 0.25732
K_score: 0.05686
Scaling factor: 0.6818

ENSMUSG00000024187 exon 4 (ORF 2)

```
Mus     GAGAAACCCTGTGGAGCCATCCCAGCAGCTTTAGTGGGAACGTTTCCATCCTGAGCCCTCTGCTCCAGGTGCCTGACATT 80
Rattus  GAGAAACGCTGTGGAGCCATCCCAGAAGCTTTAGTGGGAATGCGTCCATCTTGAGTCCTCTGCTCCAGGTGCCTGACATC 80
Cavia   GGGAAACCCTGTGGAACCACCCCAGTAGCTTTGGTGCGAACACGTCTATCCTGAGCCCTCTGCTCCAAGTACCTGACATC 80
Homo    GGGAAACCCTGTGGAACCACAGCAGCAGCTTCAGCGGGAATGCGTCCATCCTGAGCCCTCTGCTGCAGGTGCCTGATGTG 80

Mus     GATGGTGATGGTGACGGTACTCCAGACCTCCTGATCCTCGCCCAGGAGGGACAGGAG 137
Rattus  GACGGTGACGGTG------CTCCAGACCTGCTGATGCTCTCCCGGGAGGGACAGGAG 131
Cavia   GACAGTGATGGTG------CCCCAGACCTGCTGATCCTCACCCAAGAGGGAAAGGAG 131
Homo    GACGGCGATGGGG------CCCCAGACCTGCTGGTTCTCACCCAGGAGCGGAGGAG 131
```

ENSMUSG00000024187 exon 5 (ORF 0)

```
Mus     GTCAGTGGAGCTCTCTATTCAGGTAGCACTGGGTACCAGATTGGCCACAGAGGCAGCCTTGGTGTGGATGGAGATGGTGT 80
Rattus  GTCAGTGGCGCTCTCTATTCAGGTAGCACTGGGTACCAGATTGGCCACAGAGGCAGCCTTGGTGTGGATGGAGATGGTGT 80
Cavia   GTCAGCGGGTCCATCTATTCAGGCAGCACTGGGCACCAGATTGGCAACAGAGGCAGCCTCGGTATGGATGGAAACAGCAG 80
Homo    GTTAGTGGCCACCTCTACTCCGGCAGCACCGGGCACCAGATTGGCCTCAGAGGCAGCCTTGGTGTGGACGGGGAAAGTGG 80

Mus     CGCCCTCCTTCATGTGACCAGAACTGGTGCTCAGTATATTCTCCTGCCCTGTG 133
Rattus  CGCCCTCCTTCACGTGACCAGAACGGGTGCTCAGTATATTCTCCTGCCCTGTG 133
Cavia   CCCCCTTCTTCACATCACCAGGACGGGTGCACACTACATTCTCTTGCCCTGTG 133
Homo    CTTCCTCCTTCACGTCACCAGGACAGGTGCCCACTACATCCTCTTTCCCTGCG 133
```

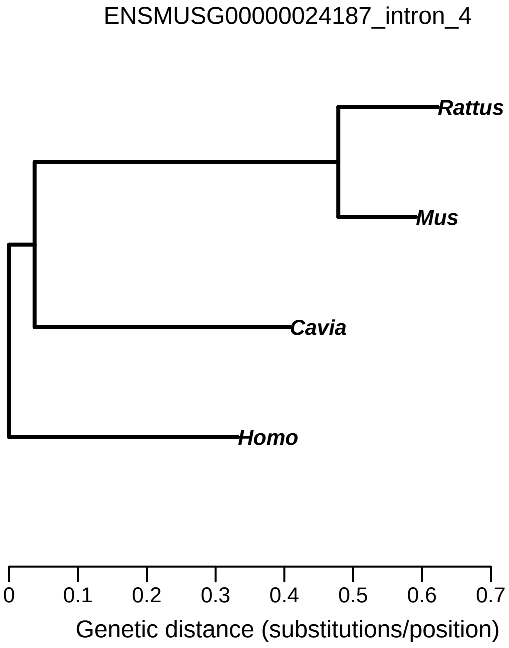

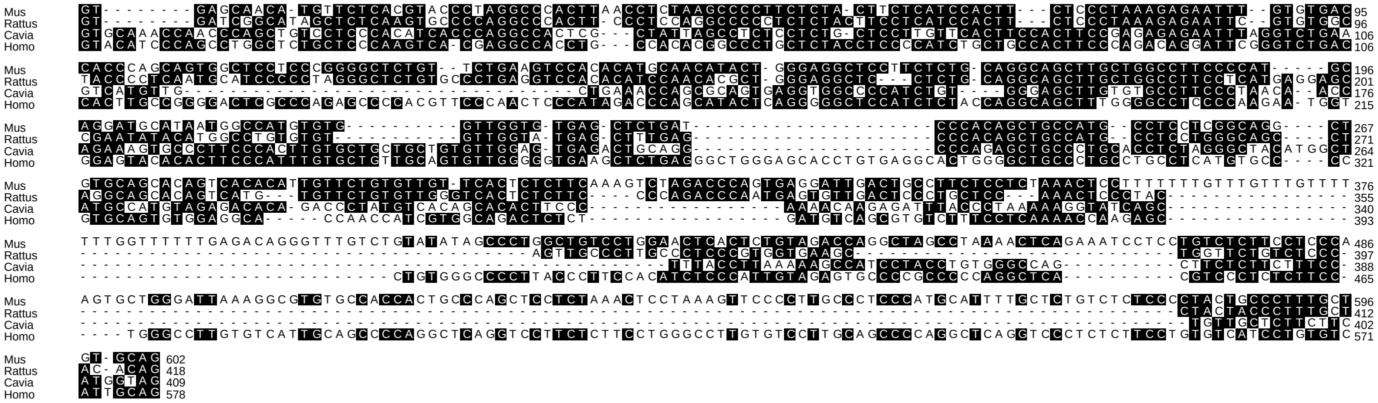
Mus
Rattus
Cavia
Homo
95
96
106
106
196
201
176
215
267
271
264
321
376
355
340
393
486
397
388
465
596
412
402
571
602
418
409
578

ENSMUSG00000009292 intron 17

Description: Transient receptor potential cation channel subfamily M memb (Trpm2)
Intron number: 17
Mouse chromosome: 10
Upstream exon length: 119
Downstream exon length: 133
Mouse intron length: 574
Intron alignment length: 676
Total murinae branch length: 0.18154
K_score: 0.06535
Scaling factor: 0.6819

ENSMUSG00000009292 exon 17 (ORF 0)

Mus	CTATTCTATGATCCTGATGGCTGTGGACTAATGAAGATGGCGTCCCTGTACTTCAGTGACTTCTGGAACAAACTGGACGT	80
Rattus	CTATTCTACGATCCCGATGGCTGCGGCTCATGAAGATGGCGTCCCTGTACTTCAGTGACTTCTGGAACAAACTGGACGT	80
Cavia	CTCTTCTGTGTCCCTGATGAGTGCCGGCTGATGAAGATGGTGTCCCTGTACTTCAGCGACTTCTGGAATAAGGTGGACGT	80
Homo	CTCTTCTATGACCCTGACGAGTGCGGGCTGATGAAGAAGGCAGCCTTGTACTTCAGTGACTTCTGGAATAAGCTGGACGT	80
Mus	TGGGGCCATTCTGCTCTTCATAGTAGGACTGACCTGTCG	119
Rattus	TGGGGCCATTCTGCTCTTTATAGCAGGACTGACCTGCCG	119
Cavia	TGGGGCCATCTTGCTCTTCATCGCGGGGCTGACGTGCCG	119
Homo	CGGCGCAATCTTGCTCTTCGTGGCAGGGCTGACCTGCAG	119

ENSMUSG00000009292 exon 18 (ORF 1)

Mus	GCTCATCCCAGCGACGCTGTACCCTGGGCGCATCATCCTGTCTTTGGACTTCATCATGTTCTGTCTCCGTCTCATGCACA	80
Rattus	ACTCATCCCAGCGACGCTGTACCCTGGGCGCATCATCCTGTCTTTGGACTTCATTATGTTCTGCCTCCGCCTCATGCACA	80
Cavia	GCTCATCCCGGCGACGCTGTACCCGGGCCGCGTGATCCTCTCCCTGGACTTCATCATCTTCTGCCTCCGCCTGATGCACA	80
Homo	GCTCATCCCGGCGACGCTGTACCCCGGGCGCGTCATCCTCTCTCTGGACTTCATCCTGTTCTGCCTCCGGCTCATGCACA	80
Mus	TCTTCACTATTAGCAAGACACTGGGGCCCAAGATAATCATCGTGAAGCGGATG	133
Rattus	TCTTCACCATTAGCAAGACACTGGGGCCCAAGATAATCATCGTGAAGCGGATG	133
Cavia	TTTTCACCATCAGCAAGACTCTGGGGCCCAAGATCATCATTGTGAAGCGGATG	133
Homo	TTTTTACCATCAGTAAGACGCTGGGGCCCAAGATCATCATTGTGAAGCGGATG	133

ENSMUSG00000009292_intron_17

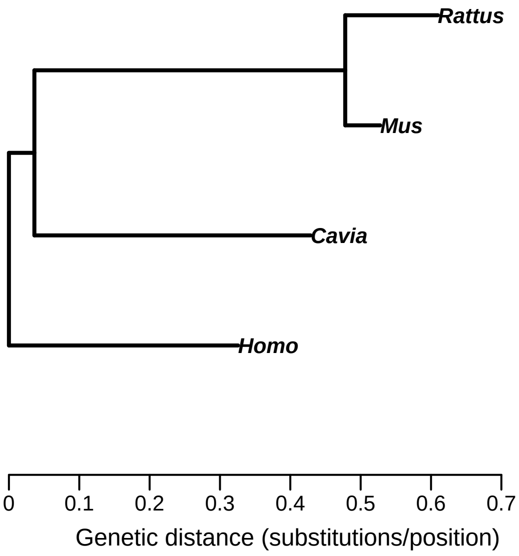

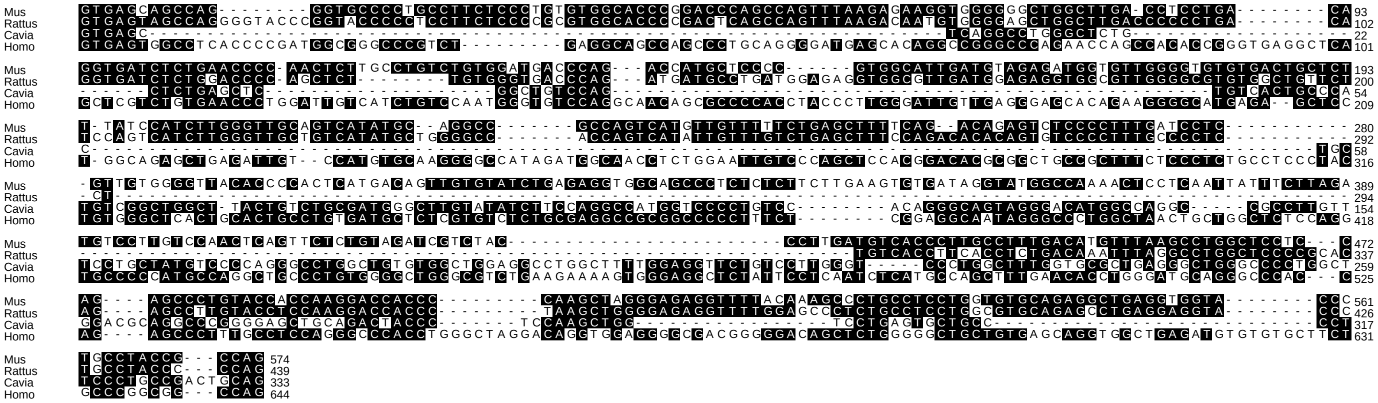
Mus
Rattus
Cavia
Homo

ENSMUSG00000020325 intron 2

Description: Follistatin-related protein 3 Precursor (Fstl3)
Intron number: 2
Mouse chromosome: 10
Upstream exon length: 186
Downstream exon length: 216
Mouse intron length: 1216
Intron alignment length: 2474
Total murinae branch length: 0.21315
K_score: 0.04431
Scaling factor: 0.68302

ENSMUSG00000020325 exon 2 (ORF 2)

```
Mus     AGAGAGGCCACCTGCAGTCTGGTGCTGAAGACTCGTGTCAGCCGGGAGGAGTGCTGTGCTTCCGGCAACATCAACACCGC 80
Rattus  AAGGAGGCCACCTGCAGTCTGGTGCTGAAGACACAGGTCAGCCGGGAGGAGTGCTGCGCTTCCGGCAACATCAACACAGC 80
Cavia   CCCGAGGCCACCTGCAGCCTGGTGCTGAGGACGGACGTGAGCCAGGCTGAGTGTTGCGCCTCCAGCAACATAGACACCGC 80
Homo    CAGGAGGCCACCTGCAGCCTGGTGCTCCAGACTGATGTCACCCGGGCCGAGTGCTGTGCCTCCGGCAACATTGACACCGC 80

Mus     CTGGTCCAACTTCACCCACCCAGGCAATAAAATCAGCCTGCTAGGGTTCCTGGGCCTCGTCCACTGCCTCCCCTGCAAAG 160
Rattus  CTGGTCCAACTTCACCCACCCGGGCAATAAAATCAGCCTGCTAGGATTCCTGGGCCTTGTCCACTGCCTACCATGCAAAG 160
Cavia   CTGGTCCAATTACACACAGCCCGAGAACAAGATCAACCTCCTGGGCCTCTTGGGCCTGGTCCACTGCCTGCCTTGCAAAG 160
Homo    CTGGTCCAACCTCACCCACCCGGGGAACAAGATCAACCTCCTCGGCTTCTTGGGCCTTGTCCACTGCCTTCCCTGCAAAG 160
```

ENSMUSG00000020325 exon 3 (ORF 2)

```
Mus     ATTCCTGCGACGGAGTGGAGTGCGGCCCCGGCAAGGCGTGCCGCATGCTGGGGGGCCGTCCACACTGCGAGTGCGTGCCC 80
Rattus  ATTCTTGCGACGGAGTGGAGTGCGGCCCCGGCAAGGCGTGCCGCATGTTGGGGGGCCGTCCACACTGCGAGTGCGTGTCC 80
Cavia   ATTCCTGTGACGGCGTGGAATGCGGCCCCGGCAAGACGTGCCGTATGCTGGGGGGTCGCCCGCGCTGCGAGTGCACGCCC 80
Homo    ATTCGTGCGACGGCGTGGAGTGCGGCCCGGGCAAGGCGTGCCGCATGCTGGGGGGCCGCCCGCGCTGCGAGTGCGCGCCC 80

Mus     AACTGCGAGGGGCTTCCCGCGGGCTTCCAGGTCTGCGGCTCTGATGGCGCCACCTACCGGGACGAATGCGAACTGCGCAC 160
Rattus  AACTGCGAGGGGGTTCCCGCGGGCTTCCAGGTCTGCGGCTCTGATGGCGCCACCTACCGGGACGAATGCGAACTGCGCAC 160
Cavia   GACTGCGCGGGGCTCCCGGAGCGCATGGAGGTCTGCGGCTCGGACGGCGCCACCTACCGCGACGAGTGTGAGCTGCGCAC 160
Homo    GACTGCTCGGGGCTCCCGGCGCGGCTGCAGGTCTGCGGCTCAGACGGCGCCACCTACCGCGACGAGTGCGAGCTGCGCGC 160
```

ENSMUSG00000020325_intron_2

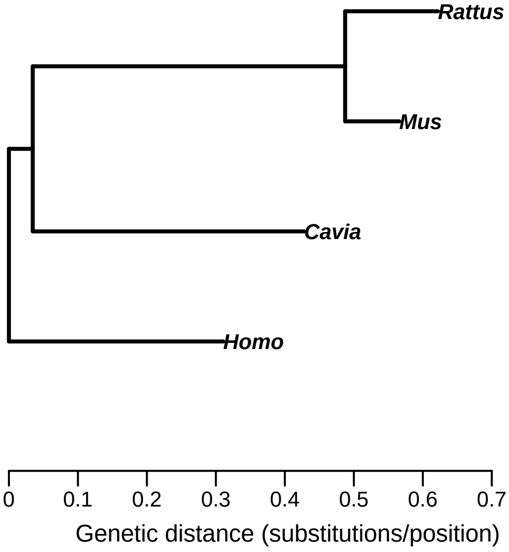

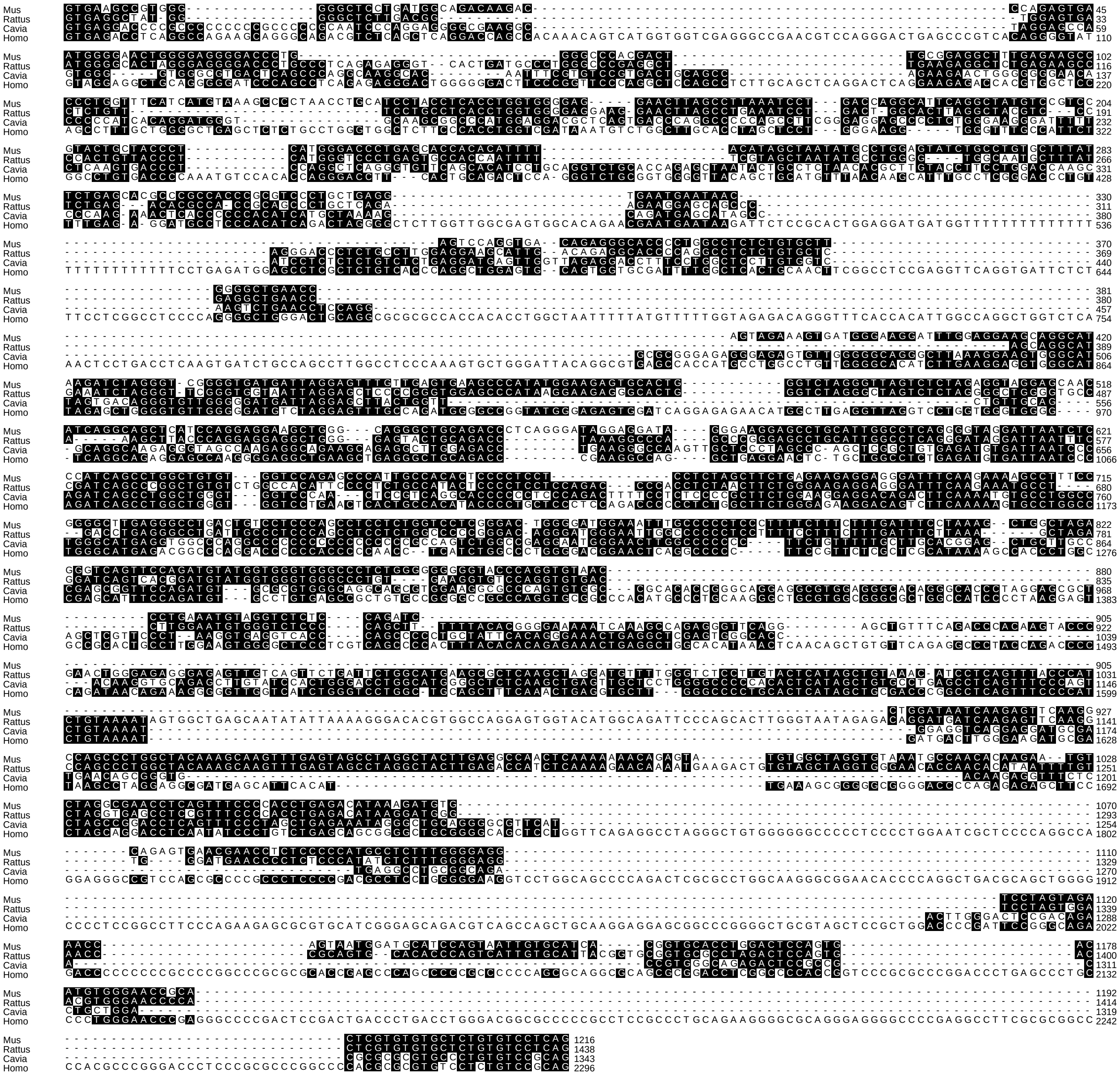

ENSMUSG00000026113 intron 19

Description: Type I inositol-3,4-bisphosphate 4-phosphatase (Inpp4a)
Intron number: 19
Mouse chromosome: 1
Upstream exon length: 127
Downstream exon length: 141
Mouse intron length: 272
Intron alignment length: 302
Total murinae branch length: 0.29315
K_score: 0.04116
Scaling factor: 0.68333

ENSMUSG00000026113 exon 19 (ORF 2)

```
Mus     GAGAGGAGTTGGCCATGTTGGAGGACATGAGCCTTGGAATCATGGACCTGAGGAATGTGACCTTTAAAGTCACTCAGGCC 80
Rattus  GAGAGGAGTTGGCCATGTTGGAGGACATGAGCCTTGGGATCATGGACCTGAGGAATGTGACCTTTAAAGTCACTCAGGCC 80
Cavia   GTGAGGAGCTGGCCATGCTGGAGGACATGAGCCTTGGGATCATGGACTTGAGGAATGTGACGTTTAAAGTCACGCAGGCC 80
Homo    GGGAGGAGCTGGCAATGCTGGAGGACATGAGCCTTGGGATCATGGACTTGAGGAACGTGACCTTCAAAGTCACTCAGGCC 80

Mus     ACTTCGAATGCATCAAATGACATGCTGCCGGTCATCACAGGAAACCG 127
Rattus  ACTTCGAATGCTTCTAGTGACATGCTGCCAGTCATCACAGGAAACCG 127
Cavia   ACTTCCAGTGCATCAGCTGACATGCTGCCCGTCATCACAGGAAATCG 127
Homo    ACTTCCAGCGCCTCCGCAGACATGCTGCCCGTCATCACAGGAAATCG 127
```

ENSMUSG00000026113 exon 20 (ORF 1)

```
Mus     GGATGGCTTTAACGTGCGGATCCCTCTGCCAGGCCCACTGTTTGACTCCCTCCCCAGAGAGATCCAGAGTGGCATGCTGC 80
Rattus  GGATGGCTTTAACGTGCGGATTCCTCTGCCAGGCCCACTGTTTGACTCTCTCCCCAGAGAGATCCAGAGCGGCATGCTGC 80
Cavia   TGATGGGTTTAATGTGCGGATCCCCCTGCCAGGCCCGCTCTTCGACGCCCTGCCCCGAGAGATCCAGAGCGGCATGCTGC 80
Homo    CGACGGGTTTAACGTGCGGGTCCCTCTGCCGGGCCCGCTGTTTGACGCCTTGCCCCGGGAGATCCAGAGTGGCATGCTGC 80

Mus     TGCGGGTGCAGCCTGTCCTCTTCAACGTGGGCATCAATGAGCAACAGACACTGGCTGAGAG 141
Rattus  TGCGGGTGCAGCCCGTCCTCTTCAACGTGGGCATCAATGAGCAACAGACACTGGCCGAGAG 141
Cavia   TGCGCGTGCAGCCAGTCCTCTTCAATGTGGGCATCAACGAGCAGCAGACCCTGGCCGAGAG 141
Homo    TGCGAGTGCAGCCCGTCCTCTTCAACGTGGGCATCAATGAGCAGCAGACACTGGCCGAGAG 141
```

ENSMUSG00000026113_intron_19

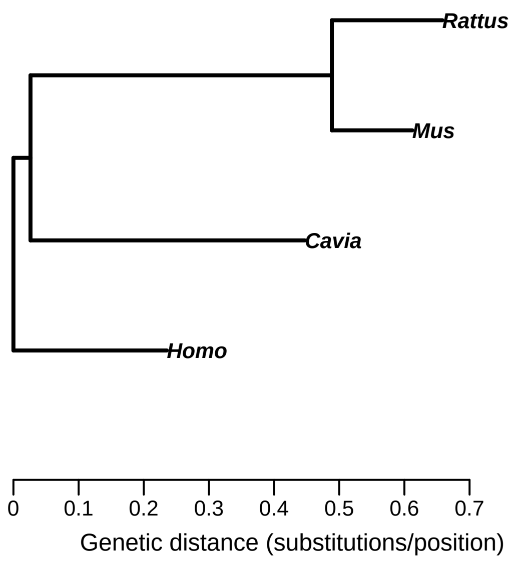

```
Mus     GTAAGAT----CATCTAACACTCTGCCAGCAGCCAGGCATCACT-----GTGTCTGTCTTGTGGGAC--------ACACACCTCTCTGTTCTTTGCTTTTACTC-----C 88
Rattus  GTAAGAT-TGTCATCTAACATTCCGTCAACAGCGGGGCTTCCCT-----GTCTCCATCCACAGTGGGGCCTCACTGTGCCTGTCTCTGTTCTTTGCTTTTATTTTTACTC 104
Cavia   GTAGAACAGTTTCTCTCAAAACCATGCACCAGAGCAGTGTTCTTCATTAATGTCCCTCTCATGGGTG-------CATGCTTCTGTTTCATTTTTACTCCATTTCTTTCTG 103
Homo    GTAGAGT-GTTGGTTTAAAATTCTCTGAGCCAGAGAGCGGTTTTCATTTTTGTTTATCTCAGGGAAG--------GTACTCTTCTGAATTTTTTATCCTATTTCATTTTG 101

Mus     TTTAGAGCATTGCATATAGCCTTCCCAGACCCATACCAATCCCATAATTT--CTCTTTAAAGGTCTGAG---TGGGCATTGAACCACACC-TGCCAGGGCATCACCCAGG 192
Rattus  TTTGGAACATGGCCTACAGCCTTCCCAGACCCATGTCAATTTTGTAATGTAAGGTGTTAAATGTCTGAG---TGGGCATTGAACGGCCCT-AGCCAGAGCATCACCCAGG 210
Cavia   TTAAAACCACTGGCTACAAGCC-------CTTACTGTGCTTCTA--------CCGCTTAAAGGTCTGGC---TGGGGTTTGGACGTCATAGACGCCCAGGGCTGCCTGGA 195
Homo    TTAAATGCTCTGATTACAGCCC-------CCTAGGTTAGTGCCAC-------TCCCTTAAAGGTCTGTGGTTGGCAGTTTGGCCATCACTAAGCCAGAGGGCCAGCCTGG 197

Mus     GCTCTGAAGTATGCTTTGGGTCTGCCCTCTACTCTCAGCGTCCTGACCTCACCCCA-CTTTGCATTTC-CTCTCTTCACTAG 272
Rattus  GCTCTAAAGTGTGCTTTGGGTGTGTCCTCCACCACCAGCGTCCTGACCTCACCCCGTCTTTGCGTTTT-CTCTCTCCACCAG 291
Cavia   CGCCCACCCTGCCCTCTGTA---------------------TTGGGGCTCACGCTGCCCTTCCTGTCT-CACCCTCCTCCAG 255
Homo    GCACTGCAGCCTGCTCGGTG-----------------GCCCTCTGGCCTCACACTGCTCTCCCTCTCTCCACCTTTCTCCAG 262
```

ENSMUSG00000021610 intron 4

Description: Cleft lip and palate transmembrane protein 1-like protein (Clptm1l)
Intron number: 4
Mouse chromosome: 13
Upstream exon length: 149
Downstream exon length: 79
Mouse intron length: 1035
Intron alignment length: 1346
Total murinae branch length: 0.14894
K_score: 0.07618
Scaling factor: 0.68789

ENSMUSG00000021610 exon 4 (ORF 0)

```
Mus     CAACAGATAGAAGCCGAGAAGAAGCCATCAAACGCCCTGGATGAACCCGTCTCTCACTGGCGACCAAGGCTGACCCTGAA 80
Rattus  CAGCAGATAGAAGCAGAGAGGAAGCCATCGAATGCCCTAGATGAGCCCGTCTCTCACTGGCGACCACGGCTGACCCTGAA 80
Cavia   ---CAGATGGAGGCTGAGAAGAAGCCATCGAGTGCCCTGGACGAGCCCATCTCTCACTGGAGACCCAGGCTAACGCTGAA 77
Homo    ---CAGATCGAGGCGGAGAAGAAGCCGACGAGTGCCCTGGATGAGCCAGTGTCCCACTGGCGACCGCGGCTGGCGCTGAA 77

Mus     TGTGATGGTGGATGACTTTGTCTTTGATGGTTCCTCCCTGCCTGCAGATGTGCATCGGTACATGAAGAT 149
Rattus  TGTGATGGTGGATGACTTCGTCTTTGATGGTTCCTCCCTCCCTGCAGATGTGCATCGATACATGAAGAT 149
Cavia   CGTGATGGTGGACGACTTTGTCTTCGATGGATCCTCGCTGCCTGCCGATGTGCACCGGTACATGAAAAT 146
Homo    CGTGATGGCGGACAACTTTGTCTTTGACGGGTCCTCCCTGCCTGCCGATGTGCATCGGTACATGAAGAT 146
```

ENSMUSG00000021610 exon 5 (ORF 1)

```
Mus     GATTCAGCTTGGGAAGACAGTGCACTACCTCCCCATCCTGTTCATTGACCAGCTGAGCAACCGGGTGAAAGACCTGATG 79
Rattus  GATTCAGCTTGGGAAGACAGTTCACTACCTCCCCATCCTGTTCATTGACCAGCTGAGCAACCGGGTGAAAGACCTGATG 79
Cavia   GATCCAGCTGGGGAAGACGGTGCACTACCTTCCCATCCTGTTCATTGACCAGCTGAGCAACCGCGTGAAGGACTTGATG 79
Homo    GATCCAGCTGGGGAAAACCGTGCATTACCTGCCCATCCTGTTCATCGACCAGCTCAGCAACCGCGTGAAGGACCTGATG 79
```

ENSMUSG00000021610_intron_4

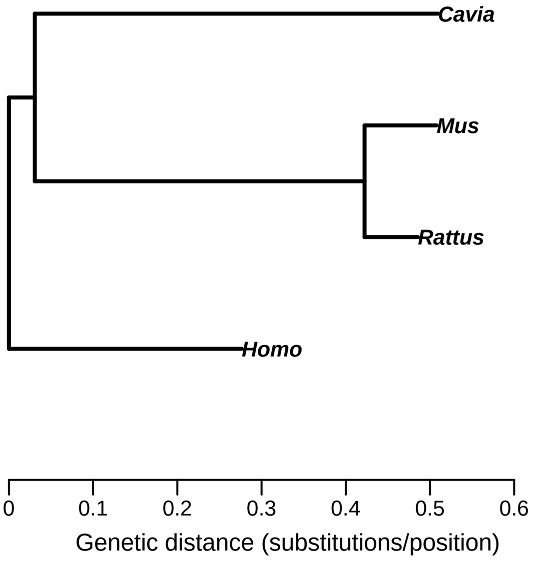

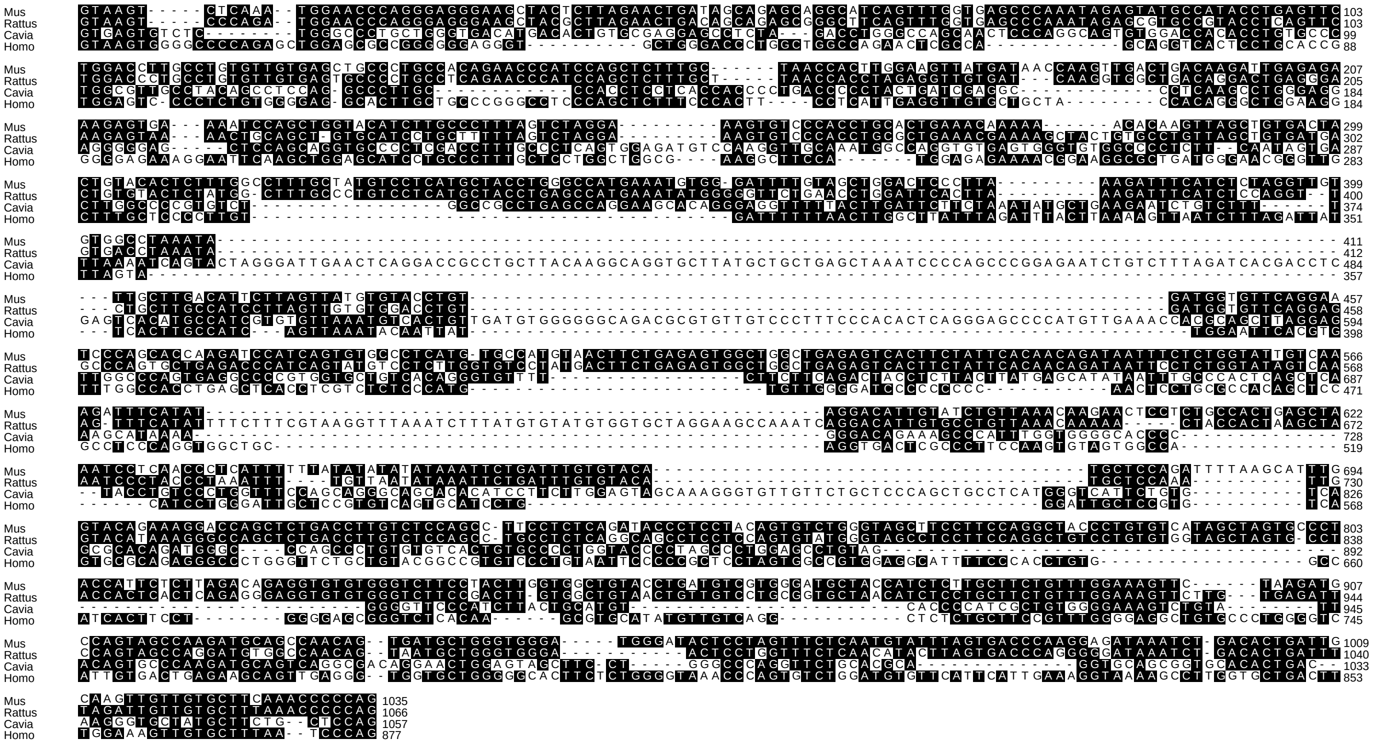

ENSMUSG00000031090 intron 4

Description: Glutamine-dependent NAD (Nadsyn1)
Intron number: 4
Mouse chromosome: 7
Upstream exon length: 54
Downstream exon length: 90
Mouse intron length: 498
Intron alignment length: 688
Total murinae branch length: 0.13169
K_score: 0.06118
Scaling factor: 0.688

ENSMUSG00000031090 exon 4 (ORF 1)

```
Mus     GCCTATAATGCACCGGAATGTTCGCTACAACTGCAGAGTCATCTTTCTCAACAG 54
Rattus  GCCTATAATGCACCGGAATGTTCGTTACAACTGCAGAGTCATCTTTCTCAACAG 54
Cavia   GGCTGTGATGCACCGAAATGTCCGCTACAATTGCAGAGTCATCTTTCTTAACAA 54
Homo    GCCTGTAATGCACCGAAACGTCCGCTACAACTGCAGAGTGATATTCCTCAACAG 54
```

ENSMUSG00000031090 exon 5 (ORF 1)

```
Mus     GAAGATCCTGCTCATCAGACCCAAGATGGCCTTGGCCAACGAAGGCAACTACCGGGAACTGCGCTGGTTTACCCCCTGGA 80
Rattus  GAAGATTCTGCTCATCAGACCCAAGATGGCCTTGGCCAATGAAGGCAACTACAGGGAACTGCGCTGGTTCACCCCCTGGG 80
Cavia   GAGGATTCTGCTCATCAGGCCCAAGATGGCCCTGGCAAATGAAGCCAACTACCGTGAGCTGCGCTGGTTTACCCCATGGT 80
Homo    GAAGATCCTGCTCATCAGACCCAAGATGGCCTTGGCCAATGAAGGCAACTACCGCGAGCTGCGCTGGTTCACCCCGTGGT 80

Mus     CCAGGAGCCG 90
Rattus  CCAGGAGCCG 90
Cavia   GCAGGAGCCG 90
Homo    CGAGGAGTCG 90
```

ENSMUSG00000031090_intron_4

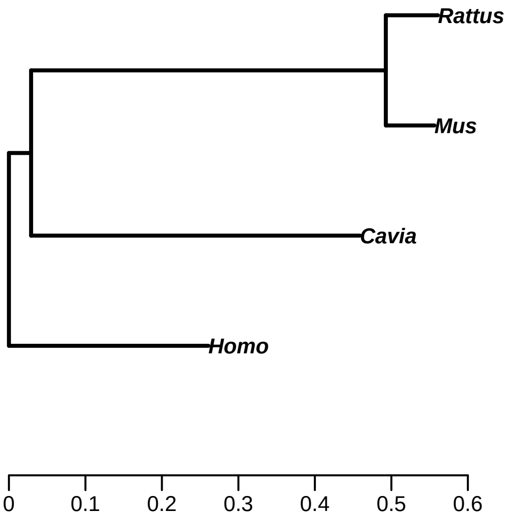

Genetic distance (substitutions/position)

```
Mus     GTAGG----------------------------------------------------TAAGGGCAGCTGGCATACCTGTGCCTGGCTCTTGTTG-------------GGG 45
Rattus  GTAGG----------------------------------------------------TGAGGGCAGCTAGCATACCTGTGCCTGGCCCTTGTCG-------------GGG 45
Cavia   GTAGG----------------------------------------TGAGCCCTGGGCTGGAGGCTGTGAG--GACCTGTGTGAGGCCTGAGGAG---------------- 52
Homo    GTAGGCCCCCTGCCCCCACCCCGGGAGGGTGACTGGGGCCTCTCCCTGGCTCTCAGCTGAGGGCTGCCAG--GACCCGTGCTGGTGCCCTGGGGACCCGTTGCTGGGAGG 108

Mus     ATACCTTGAGAGGTGCC----CCTTAGTGAGCACAGCATACTGGCCCAGCAATTCCTATGACTCTCCTTCTCTGGGACTCAGCCACCCTCACTCAGGCTAG--------- 142
Rattus  AGACCTTGTGGGGT---------------------GCACACTGGCCCAGCAATTCCTATGAATCTCCTTCTCTGGGGCTCAGCCACCCTCACTCAGTCTAG--------- 125
Cavia   -----------------------------------------------------CCATGTGCCACAGCCCTTTTCAGG---AGATATCCTCAATGAGGACACCAAACCAGA 106
Homo    CTGCTTCTGGAAGTGCCCGGGCTTAGTGAGGGCCGATGCACCAGGCAGGTGATCCGGGTGCCTTTGCTTTTCTGGGTCTCAGCCACCACCGAGACAGGCATCATCAGGTA 218

Mus     ------AGGAGGAGACTCAAGGTTGGGTTTGTTTGGGCTTTTTATCCTTGCCACCTGGACAATAGATAAGGTGGGGGAAGTCTCCTTGCCATGCCCCGCCAAACCACCCT 246
Rattus  ------AGGAGGAGACTCAAGGTTGGGTTTCTTTGGGTTTCTTATCCTTGCTACCTGGACAATAGACAAGGTGAGGGAAGCTTCCTTGCCACAACC--CCAAACCACCCT 227
Cavia   AAGTTTGGAGAGACACCCAGAGGG-GGTGGGGCTTGGAGGCGCGATCTTTCAGCCTGAATGTTAATCTAGGTGAGGGAGAACCCCTTT-----------------CAGTC 198
Homo    TCAGAAGGAGAGAGACGCAGAGGGTGGGGGTGTGGGGACACAGGTCCTTGTGGCCTGGAAGCCAGTCCAGGTGAGCAAGTTCCCTTGG-----------------AGCGC 311

Mus     CCACACACCAGGCTACAGCAAAGTGCACATGTT------------------------------------GGGTGGATAGAGCCATGGAGTATTTCTTCCCCCACCCCAAA 320
Rattus  CCACACACCAGCCTACAGCAAAGTGCATGTGCT------------------------------------GGGTGGATGGAACCATGA--------------------AAA 281
Cavia   CTTTTCCTTAGCTGCCTGTGAGGTACAC---TTTTAGGCCTTCGCCCTTCTGTCCTTGTGGACTGTCAGTCATGGATGGAGCTGTGGAG---------ACCTGTTTTGCA 296
Homo    CTGGTCACCAGCTGCTGGGGAGGCGCACACCTTCTGGGGCTTAGCTGCTTCATC---GTTGACGGTCACGAATGGATGCAGCCATGGGG---------ACCCGAGTCATA 409

Mus     CAGAAACCTTTAGCCTAGAGA---------------AAGGGAGGAAGGAAGGGAGGGAGGGAGGGAGGGAGGAAGGTAGGTAGGT------------------------- 390
Rattus  CAGAAAACTTAAGCCTAGAGAAAGAAAGAAAGAAGGAAGGAAGGAAGGAAGGAAGGAAGGAAGGAAGGAAGGAAAAAAGGTAGGTGAGAGAGAGAGAGAGAGAGAGAGAG 391
Cavia   GACAAAGCTGAAGCTCAGAGG----------------------------------------------------------------------------------------- 317
Homo    G--AAAGCGGAAGCCCAGAGA----------------------------------------------------------------------------------------A 429

Mus     ------AGGTTTGCTCAGGGCCCTGGTCTTGAGCCAGCTCAGTA--GCCTTATCTTATCCTATGGACAGATTTTGTG--------------------GCAGAAGTGTGGT 472
Rattus  AGAGAGGGGTTTGGTCAGAGCCCTAGTCTTGAACCAGCTCAGTA--GCCTTACCATATCCTATGGACAGATTGTGTG---GCAGTGGCCTGCTTGTCCCAGGAGTTTGGT 496
Cavia   AGGAAGAGATTTATTCAGGGCTTGGTTCTTGAACCGACTCCAT---GTATAACCTGG--CTTGGTTGGTGATGACTGTGCAAACTGGCTCCCTGGTTTCAGCAGCTTGTC 422
Homo    GGAGAGAGATTTGCTCGGGGCCCCATTCTCAAGCTGACTCTGCATGGCATCACCTCGTCACTTGGTGGCACTGACCACCGCCAGTGGCACGTTCATCTCAGGAGCCCGGT 539

Mus     GTGAACAGTTTTTTCACCT--GCTGTAG 498
Rattus  GTGAACAGTTTTTTCACTT--GCCATAG 522
Cavia   CTGACCTGCCCTGTTTCCT--GCTGTAG 448
Homo    GTGCACAGCCCTGTTCCCCTGTCTGCAG 567
```

ENSMUSG00000010205 intron 10

Description: Ribonucleoprotein PTB-binding 1 (Raver1)
Intron number: 10
Mouse chromosome: 9
Upstream exon length: 75
Downstream exon length: 144
Mouse intron length: 459
Intron alignment length: 880
Total murinae branch length: 0.16701
K_score: 0.08305
Scaling factor: 0.68806

ENSMUSG00000010205 exon 10 (ORF 0)

```
Mus     GATGTGGGACCTAGACGACTCTTCTCCCACCCTCGGGAACCAACCTTAGGGGCTCATGGCCCCAGCCGACACAAG 75
Rattus  GATGTGGGACCTAGACGGCTCTTCTCCCACCCTCGGGAATCAAACTTAGGGGCTCATGGCCCCAGCCGACACAAG 75
Cavia   GATGTGGGACCTCGGCGGCTCTTCTCCCAGCCCCGGGAGCCAAGCCTAGGGCCTCACGGACCCAGTCGACACAAG 75
Homo    GACATGGGACCTCGGCGGCTCTTCTCCCACCCACGGGAACCAGCCCTTGGGCCTCACGGACCCAGCCGACACAAG 75
```

ENSMUSG00000010205 exon 11 (ORF 0)

```
Mus     ATGTCCCCCCCACCTAGCAGCTTCAATGAGCCCCGAAGTGGTGGAGGCAGTGGGGGACCCCTCTCTCATTTTTACTCTGG 80
Rattus  ATGTCCCCCCCACCTAGCAGCTTCAGTGAGCCTCGCAGTGGTGGAGGCAGTGGGGGACCCCTCTCTCATTTCTATTCTGG 80
Cavia   ATGTCCCCCCCACCCAGCGGCTTCAGTGAGC---GCGGTGGCGGAGGCGGAGGGGGGCCGCTCTCCCACTTCTACTCCGG 77
Homo    ATGTCCCCCCCGCCCAGTGGCTTCGGCGAACGGAGCAGCGGTGGGAGTGGCGGGGGCCCCCTGTCTCACTTCTATTCAGG 80

Mus     CTCACCCACCTCCTACTTCACCAGTGGCCTACAGGCTGGCCTCAAGCAGAGCCACCTGAACAAG 144
Rattus  CTCACCCACCTCCTACTTCACCAGTGGCCTACAGGCTGGCCTCAAGCAGAGCCACCTGAACAAG 144
Cavia   CTCACCCACCTCCTACTTCACCAGTGGCCTGCAGGCTGGCCTCAAGCAGAGCCACCTCAGTAAG 141
Homo    CTCGCCCACTTCCTACTTCACCAGCGGCCTGCAGGCTGGCCTCAAGCAGAGCCACCTCAGTAAG 144
```

ENSMUSG00000010205_intron_10

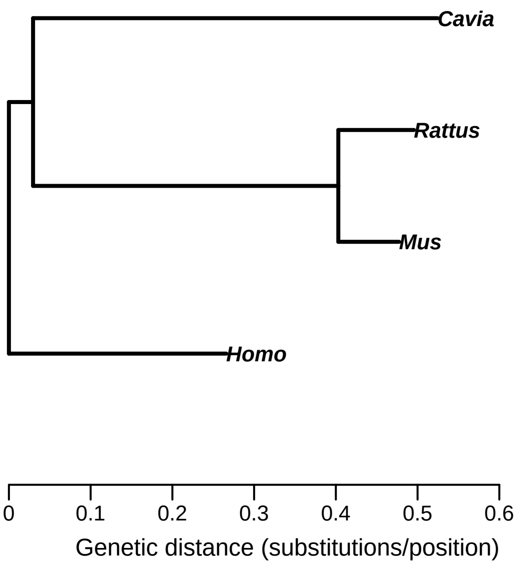

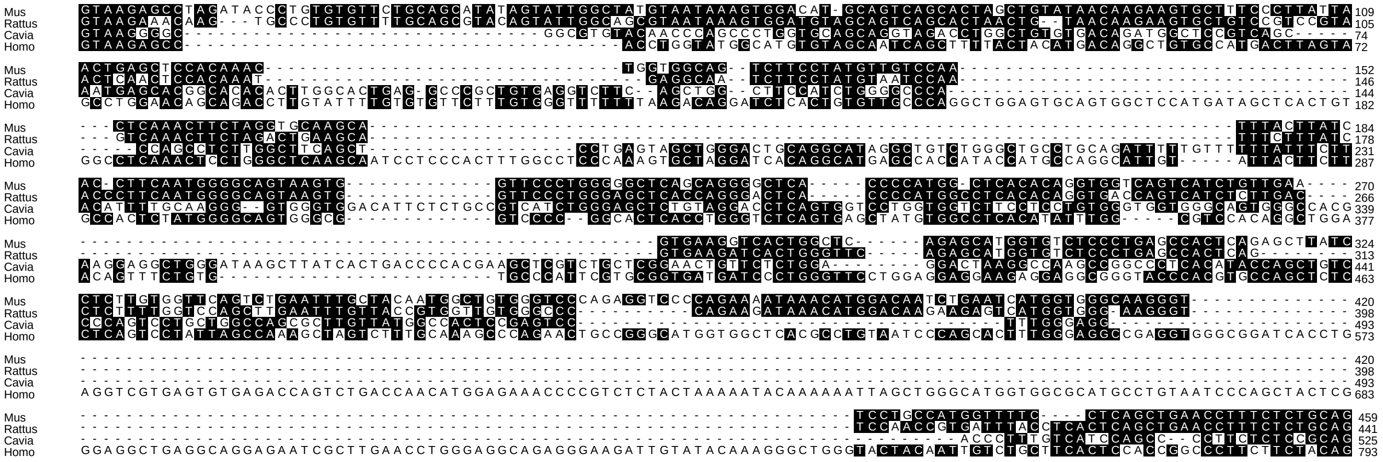
Mus
Rattus
Cavia
Homo

ENSMUSG00000035078 intron 7

Description: Myotubularin-related protein 9 (Mtmr9)
Intron number: 7
Mouse chromosome: 14
Upstream exon length: 142
Downstream exon length: 221
Mouse intron length: 1393
Intron alignment length: 2256
Total murinae branch length: 0.18768
K_score: 0.07183
Scaling factor: 0.689

ENSMUSG00000035078 exon 7 (ORF 1)

```
Mus     GGAAGGTGCATCAGTGTTGATTCATGGGACAGAAGGAACTGATTCCACACTGCAGGTGACTTCTCTGGCCCAGATCATCC 80
Rattus  GGAAGGTGCATCAGTGTTGATCCATGGGACAGAAGGAACTGATTCCACACTGCAAGTGACTTCTCTGGCCCAGATCATCC 80
Cavia   GGAAGGTGCATCCGTGCTGGTCCATGGGACAGAAGGAACCGACTCCACACTGCAGGTGACCTCCCTGGCCCAGATCATCT 80
Homo    GGAAGGAGCATCAATATTGATTCACGGAACAGAAGGAACTGATTCCACACTCCAGGTGACCTCCTTGGCCCAGATCATCT 80

Mus     TGGAACCAAGAAGCAGGACCATCCGAGGCTTTGAGGCCCTGATAGAGAGGGAGTGGCTGCAG 142
Rattus  TGGAACCGAGAAGCAGAACCATCCGAGGCTTTGAAGCCCTGATAGAGCGAGAGTGGCTGCAG 142
Cavia   TGGAACCGAGGAGCAGGACCATCCGGGGCTTTGAGGCACTGATAGAAAGGGAGTGGCTCCAG 142
Homo    TAGAGCCAAGAAGCAGGACCATTCGTGGTTTTGAGGCCCTGATTGAAAGAGAGTGGCTGCAG 142
```

ENSMUSG00000035078 exon 8 (ORF 0)

```
Mus     GCTGGTCACCCATTCCAACAGCGCTGTGCACAGTCAGCCTATTGCAGCAGCAAGCAGAAGTGGGAGGCCCCCGTGTTTCT 80
Rattus  GCTGGTCACCCGTTCCAGCAGCGCTGTGCACAGTCAGCCTATTGCAGCAGCAAGCAGAAGTGGGAGGCCCCCGTGTTTCT 80
Cavia   GCGGGTCACCCGTTCCAGCAGCGCTGCGCACAGTCAGCCTACTGCAGCAGCAAGCAGAAGTGGGAGGCGCCGGTGTTTCT 80
Homo    GCTGGTCACCCATTCCAGCAGCGCTGTGCACAGTCAGCCTACTGTAACACCAAGCAGAAGTGGGAGGCTCCTGTATTTCT 80

Mus     TCTCTTCCTGGACTGCGTGTGGCAGATCCTCCGGCAGTTCCCTTGCTCCTTCGAGTTTAATGAGCATTTTCTCATCATGC 160
Rattus  TCTCTTCCTGGACTGTGTGTGGCAGATCCTGCGGCAGTTCCCCTGCTCCTTTGAGTTTAATGAGCATTTTCTCATCATGC 160
Cavia   GCTCTTCCTGGACTGCGTGTGGCAGATACTTCGTCAGTTCCCCTGCTCTTTTGAATTCAATGAGCATTTCCTCATCGTGC 160
Homo    TCTCTTCTTGGACTGCGTGTGGCAGATCCTTCGTCAGTTTCCCTGTTCTTTTGAGTTTAATGAGAATTTCCTCATCATGC 160
```

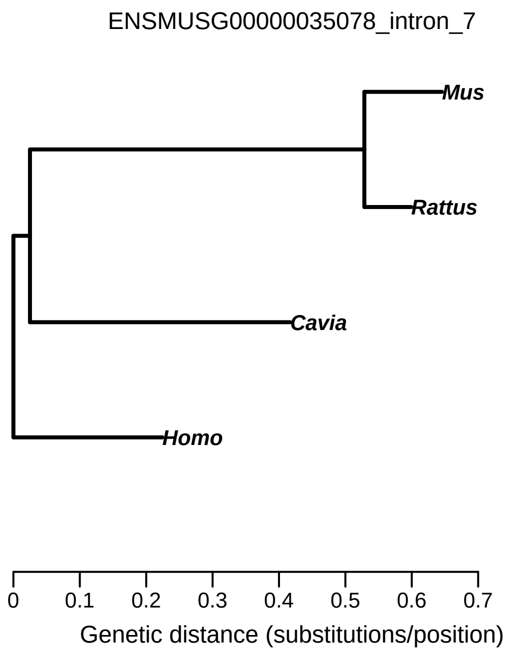

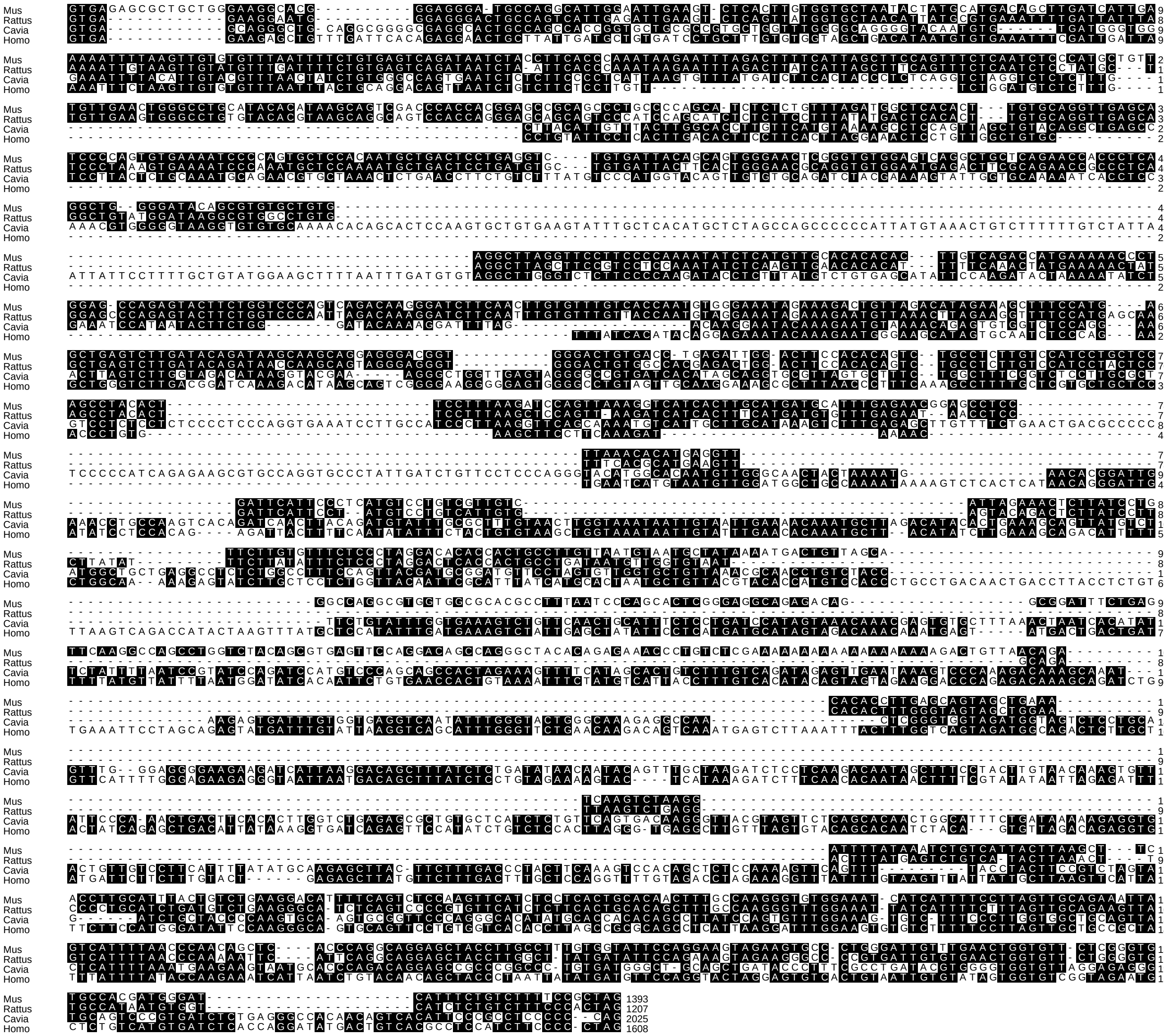
Mus
Rattus
Cavia
Homo

ENSMUSG00000038567 intron 2

Description: 1,25-dihydroxyvitamin D (Cyp24a1)
Intron number: 2
Mouse chromosome: 2
Upstream exon length: 191
Downstream exon length: 94
Mouse intron length: 756
Intron alignment length: 1579
Total murinae branch length: 0.16771
K_score: 0.08597
Scaling factor: 0.69088

ENSMUSG00000038567 exon 2 (ORF 0)

```
Mus    TCCGGATGAAGCTGGGCTCCTTCGACTCGGTGCATCTGGGCTCGCCGAGCCTGCTGGAAGCTCTGTACCGCACAGAGAGC 80
Rattus TCCGGATGAAGCTGGGCTCCTTCGACTCCGTGCATCTGGGCTCGCCGAGCCTGCTGGAAGCTCTGTACCGCACAGAGAGC 80
Cavia  TCCGGATGAAACTGGGTTCCTTCGACTCGGTGCACCTCGGCTCGCCGAGCCTGCTGGAAGGGCTGTACCGCACCGAGAGC 80
Homo   TCCGCATGAAGTTGGGTTCCTTTGAGTCGGTGCACCTGGGCTCGCCATGCCTGCTGGAAGCGCTGTACCGCACCGAGAGC 80

Mus    GCGCATCCCCAGCGGCTAGAGATCAAACCCTGGAAAGCCTATCGGGACCATCGCAACGAAGCCTACGGGCTGATGATCCT 160
Rattus GCGCATCCCCAGCGACTGGAGATCAAACCTTGGAAAGCCTATCGCGACCACAGGAACGAAGCCTACGGGTTGATGATCCT 160
Cavia  GCTTATCCGCAGCGGCTGGAGATCAAGCCGTGGAAAGCCTACCGCGACTACCGCGGGGAGGGCTACGGGCTGATGATCCT 160
Homo   GCGTACCCGCAGCGGCTGGAGATCAAACCGTGGAAGGCCTATCGCGACTACCGCAAAGAAGGCTACGGGCTGCTGATCCT 160
```

ENSMUSG00000038567 exon 3 (ORF 1)

```
Mus    GGAAGGACAGGAGTGGCAGCGGGTCCGCAGCGCCTTCCAAAAGAAACTCATGAAGCCCGTGGAAATCATGAAGCTGGACA 80
Rattus GGAAGGACAGGAGTGGCAAAGGGTCCGCAGCGCCTTCCAGAAGAAACTCATGAAGCCCGTGGAGATCATGAAGCTGGACA 80
Cavia  GGAAGGAGAAGACTGGCAGAGGGTCCGGAGCGTTTTTCAAAAGAAATTAATGAAACCAGTGGAAATTATGAAGCTGGACA 80
Homo   GGAAGGGGAAGACTGGCAGCGGGTCCGGAGTGCCTTTCAAAAGAAACTAATGAAACCAGGGGAAGTGATGAAGCTGGACA 80

Mus    AGAAAATCAATGAG 94
Rattus AGAAAATCAATGAG 94
Cavia  GCAAAATCAATGAG 94
Homo   ACAAAATCAATGAG 94
```

ENSMUSG00000038567_intron_2

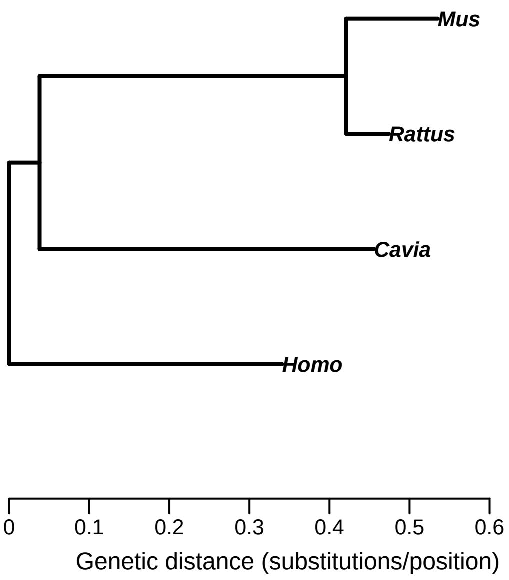

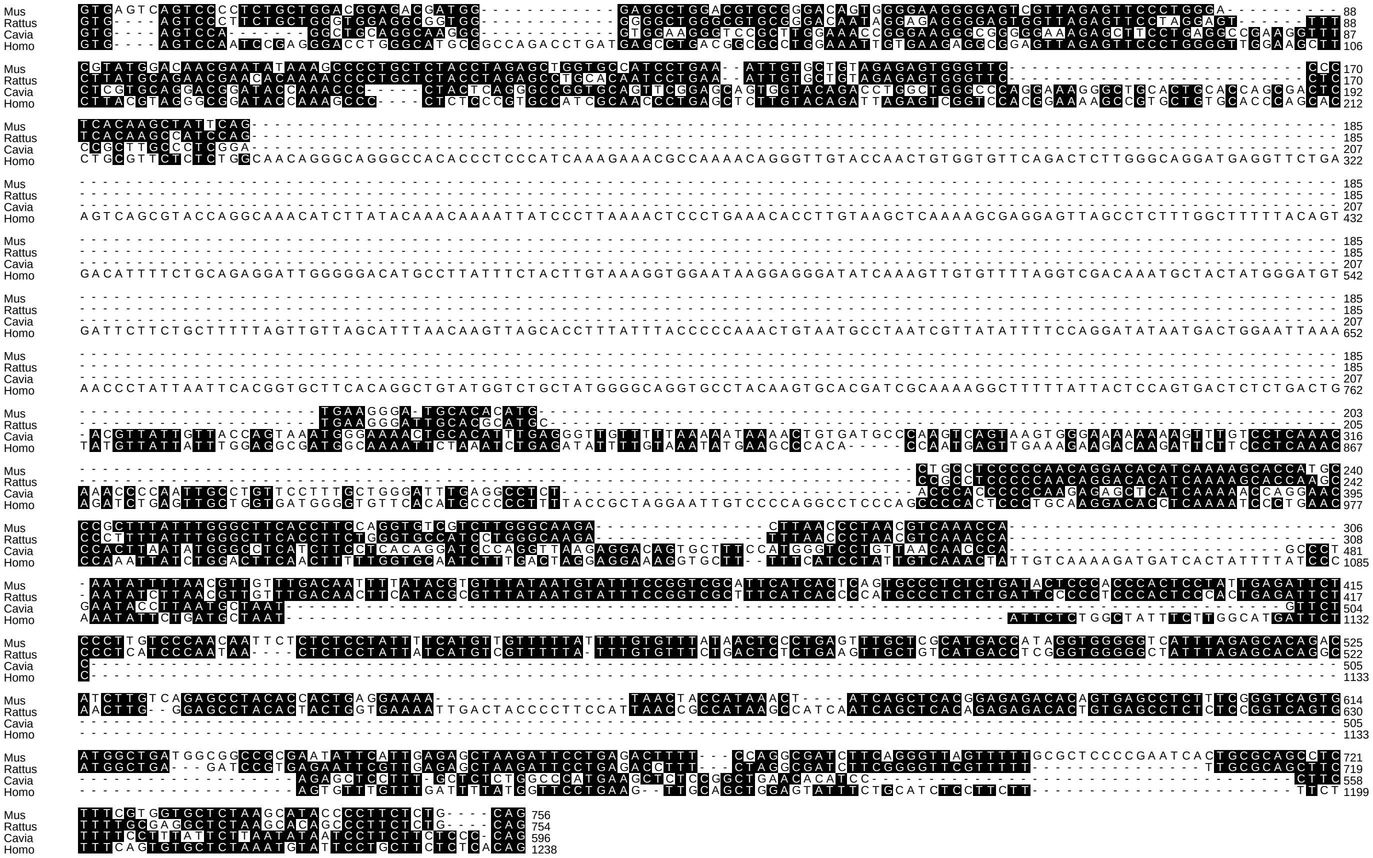
Mus
Rattus
Cavia
Homo

ENSMUSG00000039959 intron 16

Description: Huntingtin-interacting protein 1 (Hip1)
Intron number: 16
Mouse chromosome: 5
Upstream exon length: 93
Downstream exon length: 102
Mouse intron length: 1302
Intron alignment length: 1778
Total murinae branch length: 0.23324
K_score: 0.08951
Scaling factor: 0.69124

ENSMUSG00000039959 exon 16 (ORF 0)

```
Mus     AACGCAGAGGTGACCAAACAGGTGTCCGTGGCCCGGCAAGCCCAGGTGGATTTGGAAAGAGAGAAAAAAGAGCTAGCAGA 80
Rattus  AATGCAGAGGTGACCAAGCAGGTGTCTGTGGCCCGGCAAGCCCAGGTGGATTTGGAAAGAGAGAAAAAAGAACTAGCGGA 80
Cavia   AACGCAGAGGTGACCAAACAGGTCTCCATGGCCAGGCAAGCCCAGGCAGATTTGGAACGAGAGAAAAAAGAGCTCGAAGA 80
Homo    AATGCAGAGGTGACCAAACAGGTGTCCATGGCCAGACAAGCCCAGGTAGATTTGGAACGAGAGAAAAAAGAGCTGGAGGA 80

Mus     TTCCTTTGCACG------------------------G 93
Rattus  TTCCTTTGCACG------------------------T 93
Cavia   TTCCTTTGAGCGAGTCAGTGACCAGGCCCAGCGGAAG 117
Homo    TTCGTTGGAGCGCATCAGTGACCAGGGCCAGCGGAAG 117
```

ENSMUSG00000039959 exon 17 (ORF 0)

```
Mus     ACTCAAGAGCAACAGGATGTTCTAGAGAACCTGAAGCATGAACTGGCCACCAGCAGACAGGAGCTGCAGGTCCTCCACAG 80
Rattus  ACTCAAGAGCAACAGGATGTTCTAGAGACCCTGAAGCACGAACTGGCCACCAGCAGGCAAGAGCTGCAGGTCCTCCATGG 80
Cavia   ACCCAGGAACAGCAGGAAATTCTAGAGAGCTTAAAGCAGGAACTTGCTACCAGCAGGCAGGAGCTGCAGCTCCTCCACAA 80
Homo    ACTCAAGAACAGCTGGAAGTTCTAGAGAGCTTGAAGCAGGAACTTGCCACAAGCCAACGGGAGCTTCAGGTTCTGCAAGG 80

Mus     CAACCTGGAAACCTCTGCCCAG 102
Rattus  CAACCTGGAAACCTCTGCCCAG 102
Cavia   CAACCTGGAAACTTCTGCCCAG 102
Homo    CAGCCTGGAAACTTCTGCCCAG 102
```

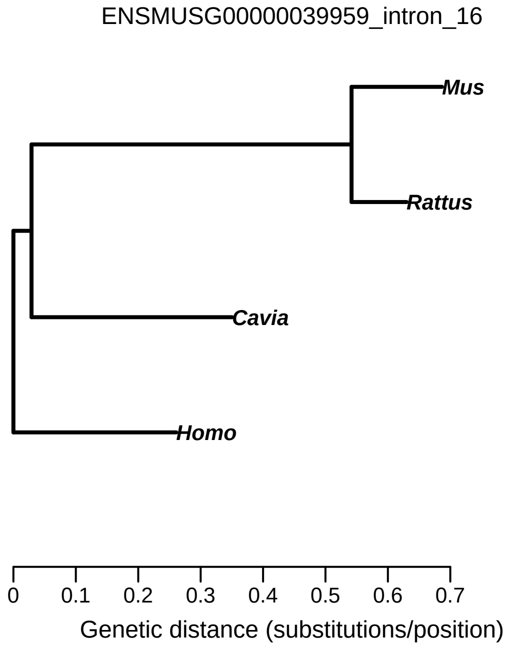

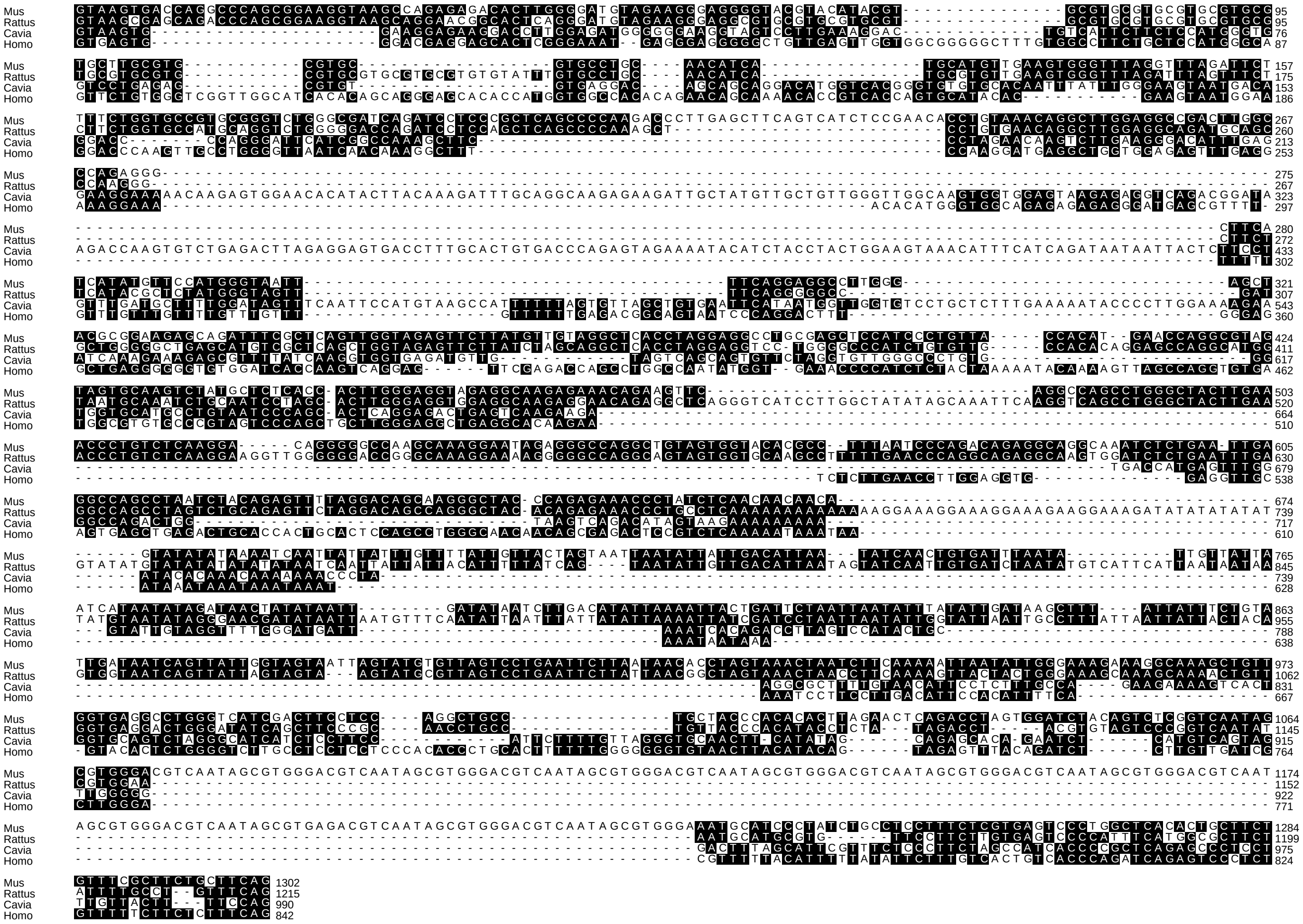
Mus
Rattus
Cavia
Homo

ENSMUSG00000030374 intron 9

Description: Striatin-4 (Strn4)
Intron number: 9
Mouse chromosome: 7
Upstream exon length: 95
Downstream exon length: 176
Mouse intron length: 1345
Intron alignment length: 1457
Total murinae branch length: 0.17228
K_score: 0.04523
Scaling factor: 0.69218

ENSMUSG00000030374 exon 9 (ORF 2)

```
Mus     ---------------------ACGTCTTCATCATGGACACTATCGGGGGCGGGGAGGTGAGCCTGGGGGACTTGGCAGAT 59
Rattus  GTTCCTTTGGCTTCTCCTCAGACGTCTTCATCATGGACACTATCGGGGGCGGGGAGGTGAGCCTGGGGGACTTGGCAGAT 80
Cavia   GTTCCTTTGGCTTCTCCTCAGACGTATTCATCATGGACACTATCGGGGGCGGGGAGGTGAGCCTGGGGGACTTGGCAGAT 80
Homo    GTTCCTTTGGCTTCTCCTCAGACGTCTTCATCATGGACACTATCGGGGGCGGGGAGGTGAGCCTGGGGGACTTGGCAGAT 80

Mus     CTCACCGTCACCAATGACAACGACCTCAGCTGTGAT 95
Rattus  CTCACCGTCACCAATGACAACGACCTCAGCTGTGAT 116
Cavia   CTCACCGTCACCAACGACAACGACCTCAGCTGTGAT 116
Homo    CTCACCGTCACCAACGACAACGACCTCAGCTGCGAT 116
```

ENSMUSG00000030374 exon 10 (ORF 0)

```
Mus     CTGTCTGACAGCAAAGACGCTTTCAAGAAGACATGGAACCCCAAGTTTACCCTCCGCTCACACTACGATGGCATCCGCTC 80
Rattus  CTGTCTGACAGCAAAGACGCTTTCAAGAAGACATGGAACCCCAAGTTTACTCTCCGCTCACACTACGATGGCATCCGTTC 80
Cavia   CTGTCTGACAGCAAAGATGCCTTCAAGAAGACGTGGAACTCCAAGTTCACCCTCCGCTCACACTACGATGGCATCCGCTC 80
Homo    CTGTCTGACAGCAAAGATGCTTTTAAGAAGACGTGGAACCCCAAGTTCACCCTGCGCTCGCACTACGACGGCATTCGTTC 80

Mus     CCTGGCCTTCCACCACAGCCAGTCAGCACTGCTTACTGCCTCTGAGGATGGCACACTCAAACTGTGGAACCTGCAGAAGG 160
Rattus  CCTGGCCTTCCACCACAGCCAGTCAGCACTGCTTACTGCCTCTGAGGATGGCACACTCAAACTGTGGAACCTGCAGAAGG 160
Cavia   CCTGGCTTTTCATCACAGCCAATCGGCGCTTCTCACTGCCTCTGAGGACGGCACTCTCAAGCTCTGGAACCTGCAGAAGG 160
Homo    CCTGGCCTTCCACCACAGCCAGTCGGCTCTGCTCACCGCCTCCGAGGACGGCACGCTCAAGCTCTGGAACCTGCAGAAGG 160
```

ENSMUSG00000030374_intron_9

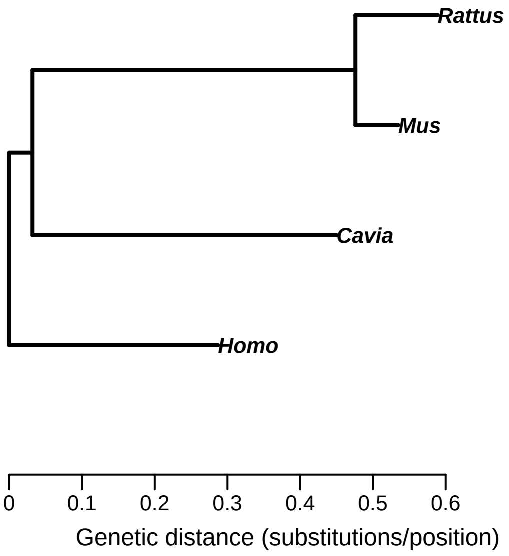

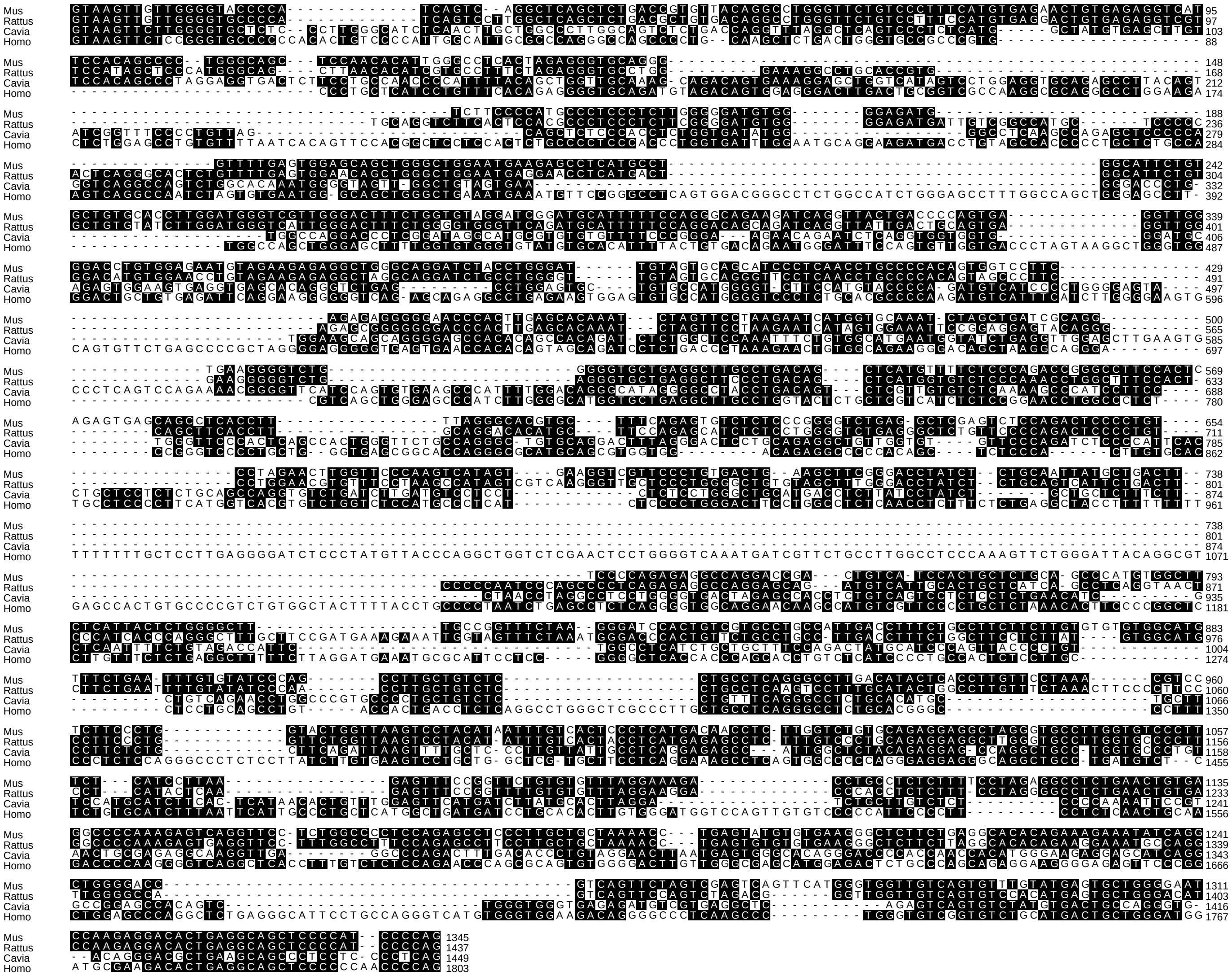

ENSMUSG00000021559 intron 3

Description: Death-associated protein kinase 1 (Dapk1)
Intron number: 3
Mouse chromosome: 13
Upstream exon length: 139
Downstream exon length: 130
Mouse intron length: 1197
Intron alignment length: 1525
Total murinae branch length: 0.17118
K_score: 0.05749
Scaling factor: 0.69278

ENSMUSG00000021559 exon 3 (ORF 1)

```
Mus     TGTTGCAGGAGGTGAGCTGTTTGACTTCCTGGCTGAGAAGGAATCTCTGACTGAAGAGGAGGCAACGGAATTCCTTAAGC 80
Rattus  TGTTGCAGGAGGCGAGCTGTTTGACTTCCTGGCTGAGAAGGAATCTCTCACTGAAGAAGAAGCAACCGAGTTCCTCAAGC 80
Cavia   CGTTGCAGGTGGCGAGCTGTTTGACTTCTTAGCTGAAAAGGAATCTCTGACTGAAGAGGAAGCGACTGAATTTCTCAAGC 80
Homo    CGTTGCAGGTGGCGAGCTGTTTGACTTCTTAGCTGAAAAGGAATCTTTAACTGAAGAGGAAGCAACTGAATTTCTCAAAC 80

Mus     AGATTCTCAGCGGCGTTTACTACCTGCACTCACTGCAGATCGCTCACTTTGACCTGAAG 139
Rattus  AGATCCTCAGCGGCGTTTACTACCTGCACTCGCTTCAAATCGCCCACTTTGACCTGAAG 139
Cavia   AGATTCTTAATGGTGTTTACTACCTGCACTCGCTTCAGATTGCGCACTTCGATCTCAAG 139
Homo    AAATTCTTAATGGTGTTTACTACCTGCACTCCCTTCAAATCGCCCACTTTGATCTTAAG 139
```

ENSMUSG00000021559 exon 4 (ORF 0)

```
Mus     CCGGAAAACATAATGCTTCTGGATAGAAATGTGCCCAAACCTCGGATCAAGATCATAGACTTTGGCTTGGCCCATAAAAT 80
Rattus  CCGGAAAACATAATGCTTCTGGATAGAAATGTGCCCAAACCTCGGATCAAGATCATCGACTTCGGCCTGGCCCATAAAAT 80
Cavia   CCTGAGAACATAATGCTTTTGGATAGAAATGTTCCCAAACCTCGGATCAAAATCATCGACTTCGGCTTGGCCCATAAAAT 80
Homo    CCTGAGAACATAATGCTTTTGGATAGAAATGTCCCCAAACCTCGGATCAAGATCATTGACTTTGGGTTGGCCCATAAAAT 80

Mus     TGACTTTGGAAATGAATTCAAAAACATATTTGGGACACCAGAGTTTGTGG 130
Rattus  TGACTTTGGAAATGAATTCAAAAACATATTTGGGACACCAGAGTTTGTGG 130
Cavia   TGACTTTGGAAATGAATTCAAGAACATATTTGGAACACCAGAGTTTGTCG 130
Homo    TGACTTTGGAAATGAATTTAAAAACATATTTGGGACTCCAGAGTTTGTCG 130
```

ENSMUSG00000021559_intron_3

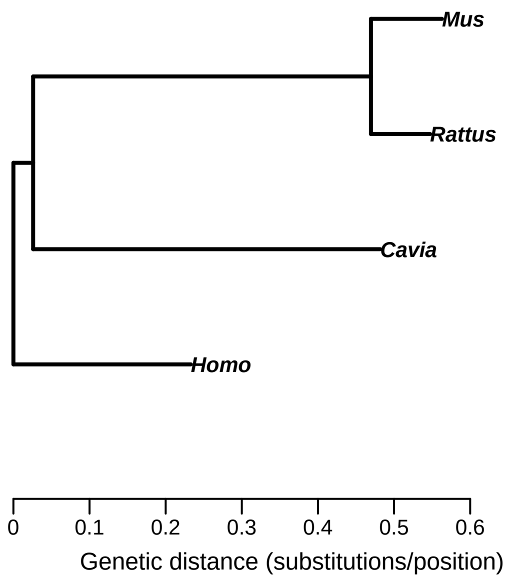

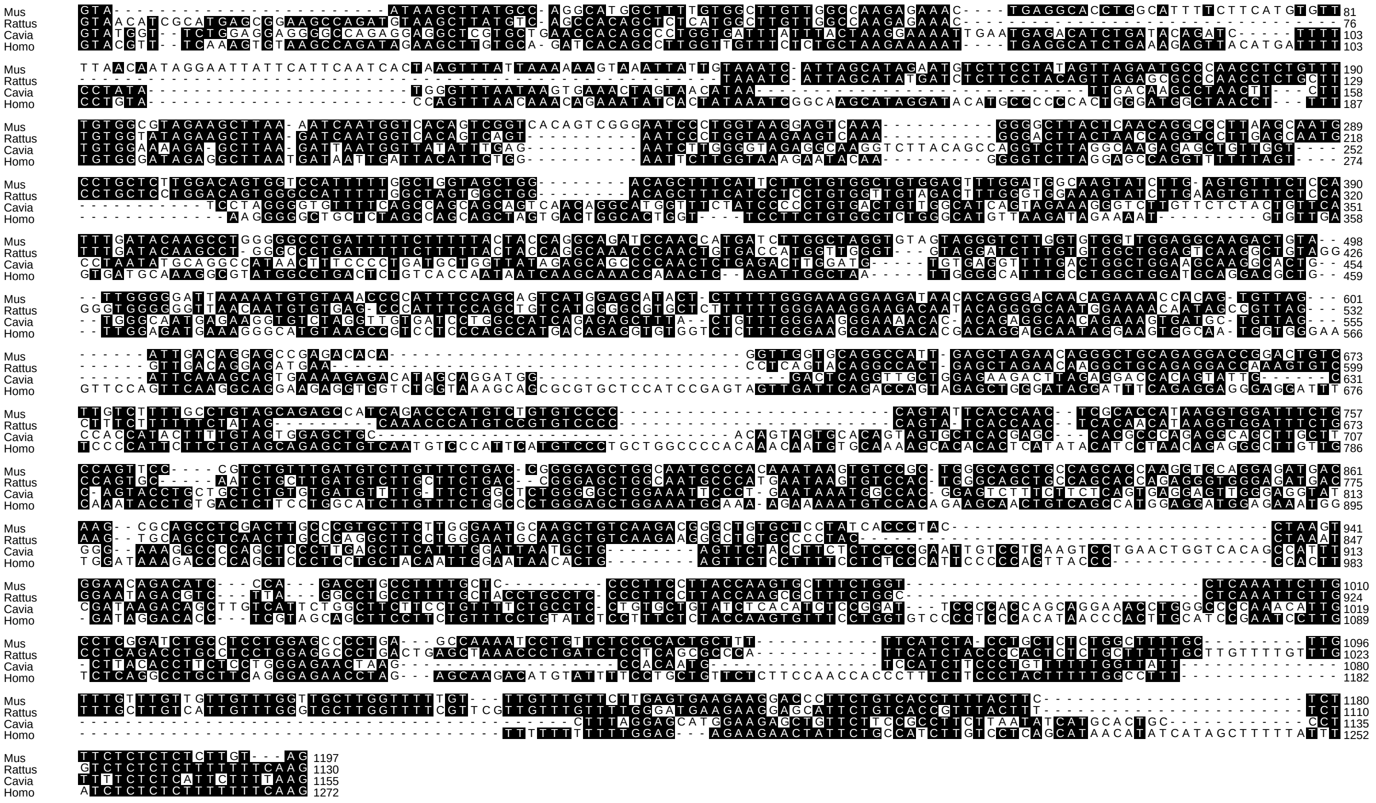
Mus
Rattus
Cavia
Homo

ENSMUSG00000021559 intron 12

Description: Death-associated protein kinase 1 (Dapk1)
Intron number: 12
Mouse chromosome: 13
Upstream exon length: 99
Downstream exon length: 99
Mouse intron length: 647
Intron alignment length: 1450
Total murinae branch length: 0.20007
K_score: 0.04469
Scaling factor: 0.69365

ENSMUSG00000021559 exon 12 (ORF 0)

```
Mus     CATGGGACACCTCCATTACTGATTGCCGCAGGCTGTGGCAACATCCAGATGTTACAGTTACTCATAAAACGAGGCTCAAG 80
Rattus  CACGGGACACCTCCATTACTGATTGCTGCAGGCTGTGGGAATATTCAGATGTTACAGTTACTCATAAAACGGGGCTCAAG 80
Cavia   CATGGGACACCTCCATTACTCATTGCTGCTGGTTGTGGGAATATTCAAATACTACAGCTGCTCCTAAAAAGAGGCTCAAG 80
Homo    CACGGGACACCTCCATTACTCATTGCTGCTGGCTGTGGGAATATTCAAATACTACAGTTGCTCATTAAAAGAGGCTCGAG 80

Mus     GATTGACGTCCAGGATAAG 99
Rattus  GATCGACGTCCAGGATAAG 99
Cavia   AATTGATGTCCAGGATAAG 99
Homo    AATCGATGTCCAGGATAAG 99
```

ENSMUSG00000021559 exon 13 (ORF 0)

```
Mus     GGAGGATCCAATGCCATCTACTGGGCCTCTCGGCATGGCCATGTGGATACTTTGAAATTTCTCAATGAGAACAAATGCCC 80
Rattus  GGAGGATCCAATGCTATCTACTGGGCCTCTCGGCATGGCCATGTGGATACTTTGAAGTTTCTCAATGAGAACAAATGCCC 80
Cavia   GGCGGATCCAATGCTGTGTACTGGGCAGCTCGGCATGGCCACGTGGACACTTTGAAATTTCTCAGTGAGAATAAGTGCCC 80
Homo    GGCGGGTCCAATGCCGTCTACTGGGCTGCTCGGCATGGCCACGTCGATACCTTGAAATTTCTCAGTGAGAACAAATGCCC 80

Mus     TTTGGATGTTAAAGACAAG 99
Rattus  TTTGGATGTTAAAGACAAG 99
Cavia   TTTGGATGTGAAGGACAAG 99
Homo    TTTGGATGTGAAAGACAAG 99
```

ENSMUSG00000021559_intron_12

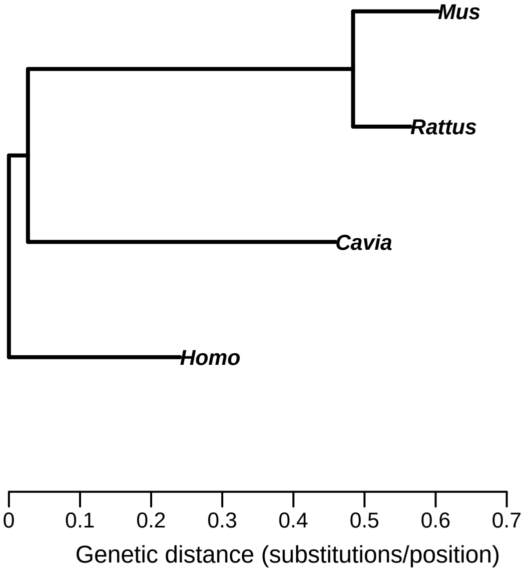

```
Mus     GTCATAATGCTATATCATTA-AAAACGAATTTA--TTTCCTGTTTTCTCAAATGTCTTCCCAATTGTAAGGGCAGTATGTGGGAGAGTATGA-------------CGAGT 94
Rattus  GTCATAATGCTGTGTCATTA-AGAATGAATTAA--TTTTATGTATTCTCAAAGGTCTT-CTAAGTGTAAGGGCAGGAGGTGGGAGAGTACGG-------------CCATT 93
Cavia   GTCATAATTCTATTTTATCATATAATGAATGCA-----------------------TTTTTAGTTGTAGAGGTACCAGGAAAAAAAAAATGTATGTTCAAGTGTCTTAAT 87
Homo    GTCATAAT--TGTTTTATT--GAAATGAATTGAATTTTAAAGTATTTTCAAGTGTCTC-TTAATCATAGGGGTAACAGAGGAAAAAAATTTATGTCCTAA-----CTTGT 100

Mus     TTCATGATTTCCTATGA---AAAGTGAGCTGGAC----AAAGGGCCAGTGCCATCTG---------AAATGCT-CCGGGAAGGGTGCACACTTAACCCC----------- 176
Rattus  TTCATGAGTCCGTACAA---AAAGCAAGCTGGACAAAGAAAGGGCCTCTGCCGTCTG---------ACATGCA-CTGGGAAAGGTGCACACTTAACTCTTTCATGTGCTC 190
Cavia   TCACCAGTTTCCTATAAAGGAAAATGAATTGCGT----AAATATCCCACATAATCGGTTTCTAAACATGTATA-AGAAAAAAAGCAAATGTTTGTCTCTTGTG------- 185
Homo    TTCTCAGTTTCCTGTAAAGGAAGATGAGCTGCTTTTA-AACAGCTTGGTACAGCCTG---------ACACATGGGTTAAAGAGGCAAACATTTATCATTTATCCTCCCCT 200

Mus     -------------------------------------------------------------------------------------------------------------- 176
Rattus  TGCTCTGTTTTTGTGCCTTATGTGCTGCAGATCACATTATTCCACAGTGCTACTGGTCTTACTTGGAAAAGTTTTCCTTGTGTAGTTACTCTAATCAGAAATTCATTAGC 300
Cavia   -------------------------------------------------------------------------------------------------------------- 185
Homo    TGG----------------------------------------------------------------------------------------------------------- 203

Mus     -----------CTGTACGC------------------------------------------------------------------------------------------- 184
Rattus  CTGAATATTTACTGTGCATCCAGGTATGGGTGTATGAGCAGGTGAAGGTGGCATTAGAAAGGCAGACAGTCATATGACAGCTGATTAGTGGATCTGTGATCAACTACCAC 410
Cavia   -------------------------------------------------------------------------------------------------------------- 185
Homo    -------------------------------------------------------------------------------------------------------------- 203

Mus     -------------------------------------------------------------------------------------------------------------- 184
Rattus  AAAAACGCTAATCTGTCCCTGAAATTCCATGAACTCAATCCTTGACCCAATCAAATGTGTACTAGAGAGCTTGTGGGGGAAAAAGATCTAGAATGTAGGTCTTCTTATGA 520
Cavia   -------------------------------------------------------------------------------------------------------------- 185
Homo    -------------------------------------------------------------------------------------------------------------- 203

Mus     --------------------------------------------------------TAATTCACAGGTGATTTAGGGCGCCCGTTCTGTGCAAGCTGCATCATTTT---- 234
Rattus  TTCTCTAGACGATGAAAAATAACAACTGTTCCCATGGTATTTCATCAGGTTATGAATAATTCGCAGGTG-TTTAGGGTACCAGTTCTATGCAGGCTGCATCATTTT---- 625
Cavia   --------------------------------------------------------------------GCTCAGTCTCAGCATTTCTTTGTACCTACC-CAGTTTT---- 222
Homo    -------------------------------------------------------------CGCTGCTGCTTGACATCAGCATTTCTTTGTACACTACCCAGATTTTACT 252

Mus     -------ATAAAAGCGT----------CTTGAA---CACTCA-----------TGTTTGTTTGTTTT---------------------GTACCTTTATAAAGGCTTGAAT 292
Rattus  -------ATAAAAGTGT----------CTTGAA---CACTCATGTGGTTTTTTTGTTTGTTTGTTTTGTTGCTGTTGTTTGGTTTTTGGTACCTTTATAGAGGCTTGAAT 715
Cavia   -------GTAAAAGTTTTCATTGTCTAGTTAAATTACTCAGAAAAAAAAATTTTGCTTATAAGTGTT-AGGGCACCCAAGTATGAATGATACCTTTTGAGGAGTGCCCTC 324
Homo    TCTTACCGATCACATTTACTCAACAATGTTAGATGTCACACATGAAAAAGTTTTGTTATCTAATTAT------------------------TCTCATCAGAAATTTGTTT 338

Mus     TGGAA----------------------------------------------TCAGTTCCTACAGATATCTAGGGTACTCTTA---------------------------- 328
Rattus  TTGAA----------------------------------------------TCCGTTCTTACAAATACTAAGGGGACTCTTA---------------------------- 751
Cavia   CAGAAGTATAGACAAAATTAACATCTCCTAATCTTTACAGTGAATATGTCCTCAGCTCCCAAAGATGCCAGTAGAGCTGTCAGTGGATTAAATCTGCCTCTTCCCAGTAC 434
Homo    G--------------------------------------------------CCTGCTTCTGCAGACGTCAGCAGAGCTGTAAGTGGAGTAGATCTGGCCCTCTGCAGTAA 398

Mus     -------------------------------------------------------------------------------------------------------------- 328
Rattus  -------------------------------------------------------------------------------------------------------------- 751
Cavia   AGCCAGCCCTGCACAGCCAGGTGTCTGCATCCCTGTGTGCACCCAACTGTGGATCAAAAATATCTCTGTTTTTCATCTCACTCTCTAAACAGCATACTATAATAACTATT 544
Homo    G------------------------------------------------------------------------------------------------------------- 399

Mus     -------------------------------------------------------------------------------------------------------------- 328
Rattus  -------------------------------------------------------------------------------------------------------------- 751
Cavia   CATATAGCATGTCCATTCTGTTGGGTGTTCTGATTAATTTAGATGTGATTTAAAGCATAAAAGAGGATATGCACTGCATCTACACAGATTCCACATTTTGTAGAAGGTGT 654
Homo    -------------------------------------------------------------------------------------------------------------- 399

Mus     -----------------------------------------------------------------------GGCATTCTAGACTGAG-GTTAATATGAGGACTTCAAAAA 366
Rattus  -----------------------------------------------------------------------GGAATTCTAGACTGAG-GTTAATATGAGGACTTCAAAAA 789
Cavia   TTGAGTGTCCATGGTCACAGGTGTCCACTAGAGAGCCTGGTGCTATTGCAGGTACTGAGGGCTACTGGATCGAGAGTTCAACTAGAG-GTGGAAATGAGGTTTAAAGAGA 763
Homo    -----------------------------------------------------------------------GGAATCCTAACCAGAGAGGGGAGATGAGGGTTTACAAAC 438

Mus     AAGAAAAACAAAACATTTTGAC------------TTTATATTGAAATACTTACACATTT----ACAAAAAAAGTAAAGATAATACAGAAAACCCCCTTCTGCTCCCTGGT 460
Rattus  AGAAGAAAGGAAA-GTTTTGTC------------TTTACATTTG---------CCCCTC----AAAAAAGTTGTAAAGATAACATAGAAAA---CCTTCTGCTCCCTAGT 870
Cavia   GAAAAGCAT-----------CT------------TTTGCTTTGAAATAGATGCAGATAT-CCCAAGGAACTTACAAAGATGTTATAAATAATTGCCTTACACCCAACACG 849
Homo    ATAAAATAA--------TTGTTTATAAGCAATTGTTTATTTTGAAATCATTTTTCATTTACAAAAAAAAGTTGGAAAAGTGGTACAGAGAGTTCCATCATTCGCTTCACC 540

Mus     GAGCATCCCCTGT-------------CATGACCATGAGCTAATTATCAAAACCTTGAAGCCAAGTG-GGCTTTTCTTCCTCTTGGAAGAGGCTCCCTTAGGGTCTGTCAC 556
Rattus  GAGCCTCACCTGTTATTAACATAGTACACGACCGTGGTGTAATTATCAAAGCCTTGAAGCTGAATG-GGCTTTTCTTCCTCTTGGGAGAGGCTACCTT-GGGTTTGTTAC 978
Cavia   TAATGTTCCCTGT----------------------GGTGCAATTGCTAAAACTATTAATGTTGAAT-GAGTTTTAGTCCTTTTGGAAGTGGCTTCTTTGGGATTTGTCAC 936
Homo    CAGGTGCCCCTAATGCTAGCATCTTGTGTAACCACGGTACATTTGTCAAAGGTGTAAAATAGAATGAGGGTTTTATTCCTCTGGGAAGTTGCTCCTCTGGGGTTTGTCAT 650

Mus     CCTATGAGAAGGAAAAC-ACTGTGGTTCTAAGGGGAGCTTG-ATCCGGGTCCATTATAGTTCTAA--ATGTGACTCTACCGTATTTTCCTTGCAG 647
Rattus  CCCACAAGAAGGGAAAC-ACTGTGGTTCTAAGGGGTGCATG-GTCCAGTTCCATTATAGTTCTAG--ATGTGACTCTATGGTGCTTTCCTTGCAG 1069
Cavia   TGCATAGGAAGGGGACT-TGAGTGATTCTAAGGGAAATATGTGTTTAGCTCTGTCGG----------TTGTGACTCCACACGGGTTTTCACGCAG 1020
Homo    CACACAGGAAGGGAAATAACAGTGAGTCTGAGGAATGCATGCATCTGGTGCTGTCAGCTCCCTAGAAATGTGACCCCAGGTTGATTTTCCTGCAG 745
```

ENSMUSG00000021499 intron 5

Description: Cation channel sperm-associated protein 3 (Catsper3)
Intron number: 5
Mouse chromosome: 13
Upstream exon length: 141
Downstream exon length: 120
Mouse intron length: 382
Intron alignment length: 444
Total murinae branch length: 0.19633
K_score: 0.06139
Scaling factor: 0.69488

ENSMUSG00000021499 exon 5 (ORF 0)

```
Mus     GTTGATGGCTGGACTGACCTGCAGGAAGAGCTGGACAAGAGGAAGTTTACTGTGAGCCGGGCGTTTACTATCCTCTTCAT 80
Rattus  GTTGATGGCTGGACAAACCTACAGGAGGAACTGGACAAGCGGAAGTTTACTGTGAGCCGGGCATTTACCATCCTCTTCAT 80
Cavia   GTGGAAGGCTGGACAGATGTTCAGGAGCAGCTGGAGAAGCACAATTTTATTGTGAGCCGAATATTCACCATTGTGTTCAT 80
Homo    GTTGATGGCTGGACAGACCTGCAGAAGCAGTTGGACAATCGGGAATTTGCTTTGAGCCGGGCATTCACCATCATCTTCAT 80

Mus     CTTGCTTGCATCCTTCATCTTCCTCAACATGTTTGTGGGTGTGATGATCATGCACACGGAG 141
Rattus  CCTGCTTGCCTCCTTCATCTTCCTCAACATGTTTGTCGGCGTGATGATCATGCACACAGAG 141
Cavia   CTTGCTTGCCTCGTTCATCTTCCTCAGCGTGTTCGTGGGTGTCATAATCATGCCAACCGAG 141
Homo    CTTGCTCGCCTCTTTCATCTTCCTCAACATGTTCGTGGGTGTGATGATCATGCACACAGAG 141
```

ENSMUSG00000021499 exon 6 (ORF 0)

```
Mus     GATTCCATGAAAAAGTTTGAGCGGGATCTGACGTTGGAGAGGAACCTTGCGATTATGGAGGAGAAGCAAATAATCCTGAA 80
Rattus  GACTCCATCAAAAAGTTCGAGCGGGAGATGACATTGGAGAGGAACTTGGCACTTATGGAGGAGAAGCAAATGATTCTGAG 80
Cavia   GTCTCCATCAAGAAGTTTAAGAAAGACCAGGTTCTGAAGAAGCGCA------TAGAGGAGAAGAAGCAGATGATCCTGAA 74
Homo    GACTCCATCAGAAAGTTTGAGCGAGAGCTGATGTTGGAGCAGCAGGAGATGCTCATGGGAGAGAAGCAGGTGATTCTGCA 80

Mus     ACGCCAGCAAGAGGAGGTCAACAGGCTGATGAACACCACAG 120
Rattus  ACGACAGCAAGATGAGGTCAACAGGCTGATGAACACCACAG 120
Cavia   ACGACAAAAGGAGGAGGTCAACAGGCAGATGCAAATCCGG 114
Homo    GCGGCAGCAGGAGGAGATCAGCAGGCTGATGCACATACAG 120
```

ENSMUSG00000021499_intron_5

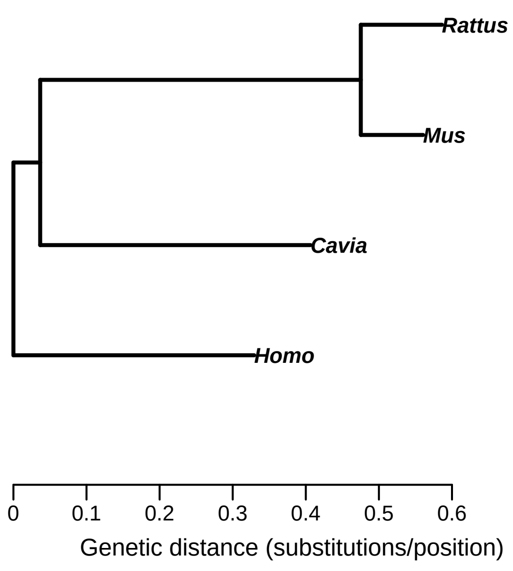

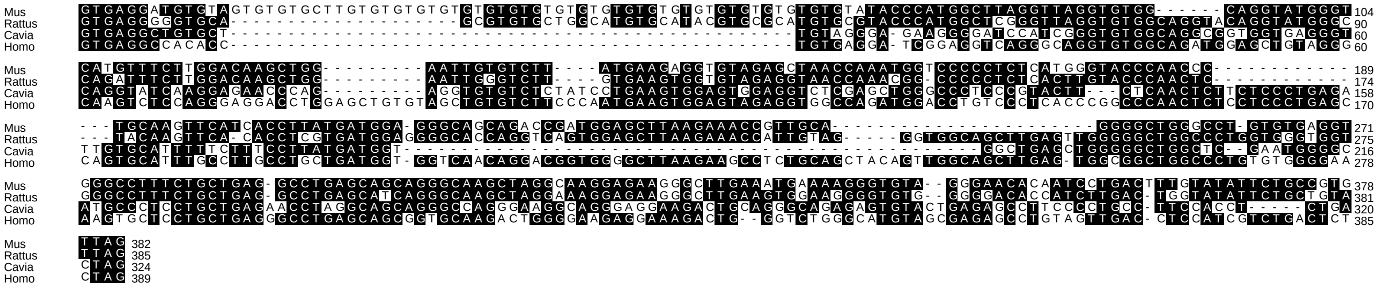
Mus
Rattus
Cavia
Homo

ENSMUSG00000048677 intron 11

Description: Two pore calcium channel protein 2 (Tpcn2)
Intron number: 11
Mouse chromosome: 7
Upstream exon length: 101
Downstream exon length: 82
Mouse intron length: 443
Intron alignment length: 742
Total murinae branch length: 0.20397
K_score: 0.08783
Scaling factor: 0.69557

ENSMUSG00000048677 exon 11 (ORF 0)

```
Mus     AAATCTCTACAGACCTCACTGTTCCGGCGGCGACTGGGGGCCCGTGCAGCCTACGAAGTCCTCGCCTCCAGGGCCGGGCC 80
Rattus  AAATCTCTACAGACTTCACTGTTCCGGCGGCGACTAGGGGCCCGTGCGGCCTATGAAGTCCTCACCCTTCAGGCAGGGCC 80
Cavia   AAATCTCTGCAGACCTCGCTGTTCCGGCGGCGCCTGGGCACCCGTGCTGCCTATGAGGTCCTCTCCTCCATGCAGGCG-- 78
Homo    AAATCTCTCCAGACCTCGCTGTTTCGGAGGCGGCTGGGAACCCGGGCTGCCTTTGAAGTCCTATCCTCCATGGTGGGGGA 80

Mus     AGCTGGAACCACCCCTGAGTT 101
Rattus  AAATGGAACCACCTCTGAGTT 101
Cavia   ----GGAGCCGCTCCTGAGAC 95
Homo    GGGAGGAGCCTTCCCTCAGGC 101
```

ENSMUSG00000048677 exon 12 (ORF 1)

```
Mus     AGTTGGGGTGAATCCTGAGACCTTCCTGCCAGTGCTTCAGAAAACGCAGCTGAACAAAACCCACAAACAAGCCATTATGC 80
Rattus  AGTCGGGGTAAATCCCGAGAACTTCCTGCGAGTGCTTCAGAAAACCCAGCTGAACAAAATCCACAAACAAGCCATCATAC 80
Cavia   AGCTGGAGTGCGGCCTCACACCTTACTGCAGGTGCTCCAGAAAGTGCAGATGAGCGGTTCTCACAAACAGGCCATCATGG 80
Homo    AGTTGGGGTGAAGCCCCAGAACTTGCTGCAGGTGCTTCAGAAGGTCCAGCTGGACAGCTCCCACAAACAGGCCATGATGG 80

Mus     AG 82
Rattus  AG 82
Cavia   AG 82
Homo    AG 82
```

ENSMUSG00000048677_intron_11

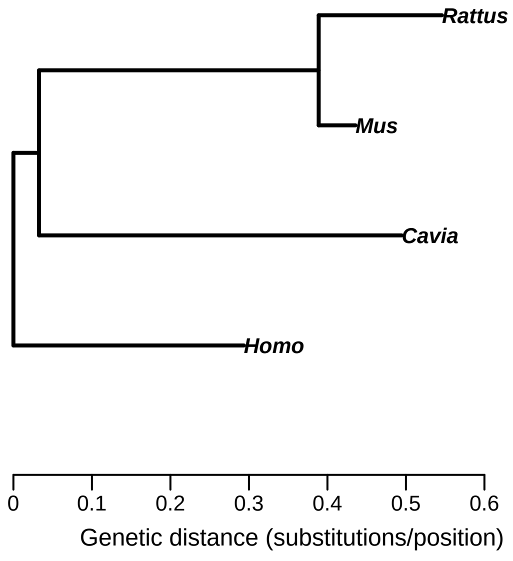

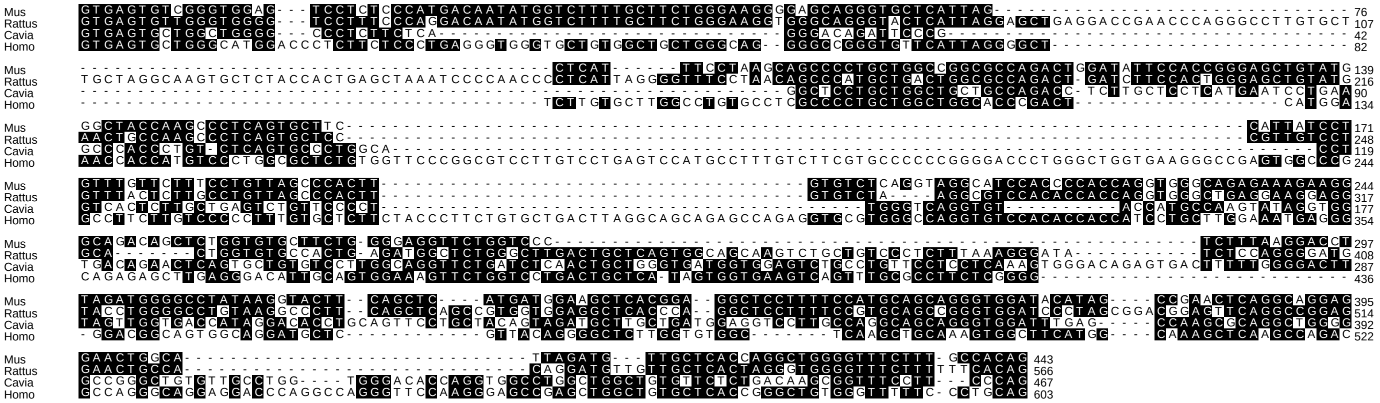
Mus
Rattus
Cavia
Homo

ENSMUSG00000026272 intron 3

Description: Serine--pyruvate aminotransferase, mitochondrial Precursor (Agxt)
Intron number: 3
Mouse chromosome: 1
Upstream exon length: 65
Downstream exon length: 101
Mouse intron length: 540
Intron alignment length: 608
Total murinae branch length: 0.14773
K_score: 0.08125
Scaling factor: 0.69612

ENSMUSG00000026272 exon 3 (ORF 2)

```
Mus     GAGCCCGTGTGCACCAGATGATCAAGAAGCCTGGAGAACATTATACACTGCAGGAGGTGGAGGAG 65
Rattus  GAGCCCGTGTGCACCAGATGATCAAGAAGCCTGGAGAACATTACACACTGCAGGAGGTGGAGGAG 65
Cavia   GAGCCCATGTGCACGCCATGATCAAGGAGCCTGGAAGCTACTACACACTGCAGGAGGTGGAAGAG 65
Homo    GAGCCCGAGTGCACCCGATGACCAAGGACCCTGGAGGCCACTACACACTGCAGGAGGTGGAGGAG 65
```

ENSMUSG00000026272 exon 4 (ORF 0)

```
Mus     GGCCTGGCCCAACACAAACCGGTATTGCTGTTCCTGGTCCACGGGGAGTCATCCACTGGCGTGGTGCAGCCCCTGGATGG 80
Rattus  GGCCTGGCTCAGCATAAACCAGTGTTGCTGTTCCTGACCCACGGGGAGTCATCCACTGGTGTGCTGCAGCCCCTGGATGG 80
Cavia   GGCTTGGCCCAGCACAAGCCGGTGCTGCTGTTTCTGACCCAGGGGGAGTCCTCCACCGGGGTGCTGCAGCCCTTGGATGG 80
Homo    GGCCTGGCCCAGCACAAGCCAGTGCTGCTGTTCTTAACCCACGGGGAGTCGTCCACCGGCGTGCTGCAGCCCCTTGATGG 80

Mus     TTTTGGGGAGCTCTGCCATAG 101
Rattus  TTTCGGGGAGCTCTGCCACAG 101
Cavia   CTATGGGGAGCTGTGTCACAG 101
Homo    CTTCGGGGAACTCTGCCACAG 101
```

ENSMUSG00000026272_intron_3

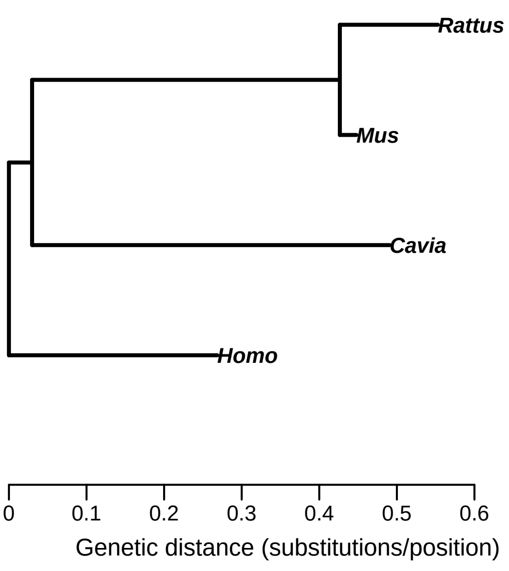

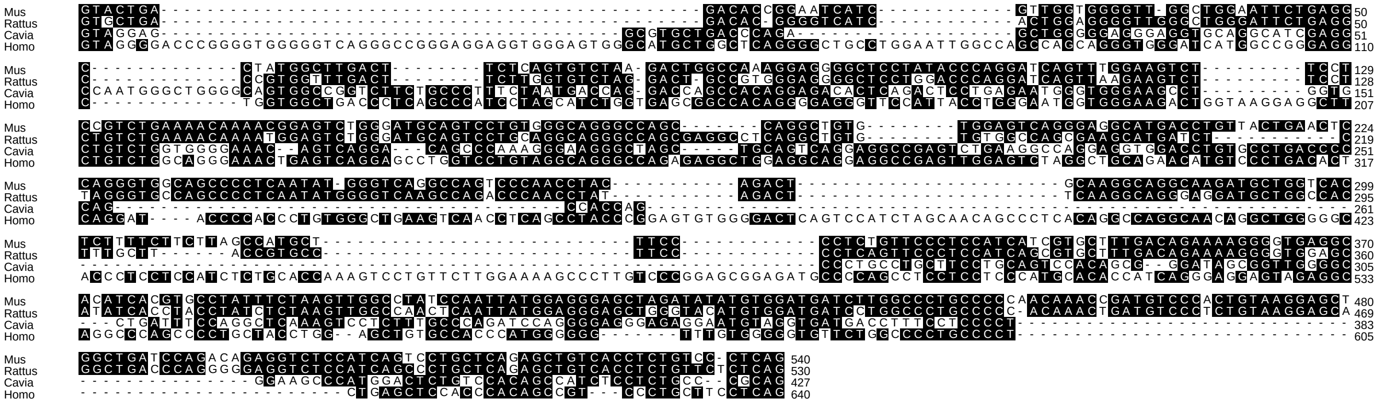
Mus
Rattus
Cavia
Homo

ENSMUSG00000023945 intron 7

Description: High affinity choline transporter 1 (Slc5a7)
Intron number: 7
Mouse chromosome: 17
Upstream exon length: 218
Downstream exon length: 630
Mouse intron length: 1527
Intron alignment length: 2150
Total murinae branch length: 0.24380
K_score: 0.08861
Scaling factor: 0.69778

ENSMUSG00000023945 exon 7 (ORF 2)

```
Mus     GATTCTCCCGATCGTTCTGCAGTACCTCTGCCCTGTGTACATCTCCTTCTTTGGGCTTGGTGCTGTTTCAGCTGCTGTCA 80
Rattus  GATTCTCCCGATTGTTCTACAGTACCTCTGCCCTGTGTACATTTCCTTCTTTGGGCTTGGTGCTGTTTCTGCTGCTGTCA 80
Cavia   GATCCTCCCGATCGTGCTGCAGCACCTCTGTCCCGTGTACGTGTCCTTCTTCGGCCTGGGCGCGGTGTCCGCTGCTGTCA 80
Homo    GATTTTACCAATTGTTCTGCAGTATCTCTGCCCTGTGTATATTTCTTTCTTTGGTCTTGGTGCAGTTTCTGCTGCTGTTA 80

Mus     TGTCCTCAGCTGACTCGTCCATCCTGTCGGCGAGTTCTATGTTTGCTCGGAATATCTACCAGCTTTCCTTCAGACAAAAT 160
Rattus  TGTCCTCGGCTGACTCATCCATCCTATCAGCAAGTTCCATGTTTGCTCGGAATATCTACCAGCTTTCCTTCAGACAAAAT 160
Cavia   TGTCCTCGGCCGACTCATCTATCCTGTCGGCCAGCTCCATGTTCGCACGCAACATCTACCAGCTCTCCTTCCGGCAAAAT 160
Homo    TGTCATCAGCAGATTCTTCCATCTTGTCAGCAAGTTCCATGTTTGCACGGAACATCTACCAGCTTTCCTTCAGACAAAAT 160
```

ENSMUSG00000023945 exon 8 (ORF 0)

```
Mus     GCATCAGACAAGGAAATTGTGTGGGTCATGAGGATCACTGTGCTTGTGTTCGGAGCATCTGCAACAGCCATGGCTTTGCT 80
Rattus  GCATCAGACAAGGAAATTGTGTGGGTCATGAGGATCACTGTGTTTGTGTTTGGAGCATCTGCAACAGCCATGGCCTTGCT 80
Cavia   GCATCAGACAAAGAAATTGTCTGGGTCATGCGAATCATGGTGTTTGTGTTTGGAGCCTCTGCCACGGCCATGGCGCTGCT 80
Homo    GCTTCGGACAAAGAAATCGTTTGGGTTATGCGAATCACAGTGTTTGTGTTTGGAGCATCTGCAACAGCCATGGCCTTGCT 80

Mus     GACGAAGACTGTGTATGGGCTCTGGTACCTGAGCTCTGACCTTGTCTACATCATCATCTTCCCACAGCTGCTCTGTGTAC 160
Rattus  CACGAAGACTGTGTATGGGCTCTGGTACCTGAGCTCTGACCTTGTCTACATCATCATCTTCCCACAGCTGCTCTGTGTAC 160
Cavia   GACCAAGACAGTGTACGGGCTCTGGTACCTCAGCTCTGACCTGGTCTACATCATCATCTTCCCACAGCTCCTGTGTGTGC 160
Homo    GACGAAAACTGTGTATGGGCTCTGGTACCTCAGTTCTGACCTTGTTTACATCGTTATCTTCCCCCAGCTGCTTTGTGTAC 160
```

ENSMUSG00000023945_intron_7

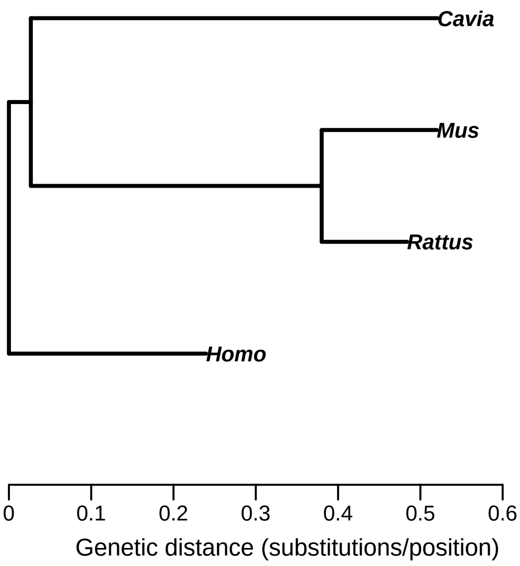

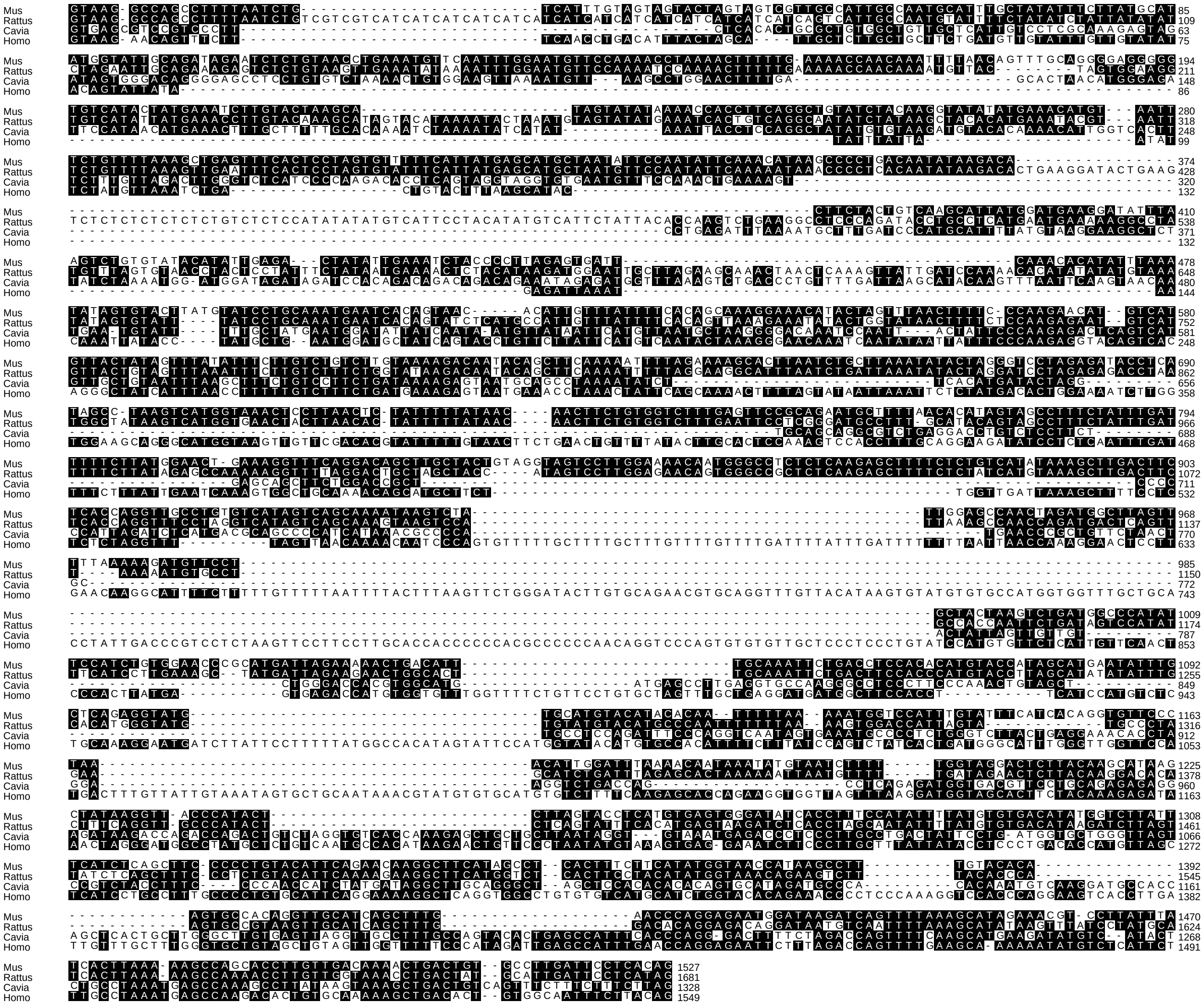
Mus
Rattus
Cavia
Homo

ENSMUSG00000038567 intron 6

Description: 1,25-dihydroxyvitamin D (Cyp24a1)
Intron number: 6
Mouse chromosome: 2
Upstream exon length: 112
Downstream exon length: 146
Mouse intron length: 1327
Intron alignment length: 1903
Total murinae branch length: 0.19803
K_score: 0.08693
Scaling factor: 0.69847

ENSMUSG00000038567 exon 6 (ORF 0)

```
Mus     ATGATGAGCACATTTGGGAAGATGATGGTGACCCCCGTGGAGCTGCACAAGCGCCTCAACACCAAAGTGTGGCAAGCGCA 80
Rattus  ATGATGAGCACGTTTGGGAAGATGATGGTGACCCCCGTGGAGTTGCACAAGCGCCTCAACACCAAAGTGTGGCAGGCACA 80
Cavia   ATGATGGGTACCTTTGGGAAGATGATGGTCACCCCGGTCGAGCTGCACAAGAGCCTCAACACCAGAGTCTGGCAGGCGCA 80
Homo    ATGATGAGCACGTTTGGGAGGATGATGGTCACTCCAGTCGAGCTGCACAAGAGCCTCAACACCAAGGTCTGGCAGGACCA 80

Mus     CACGCTGGCCTGGGACACCATTTTCAAATCAG 112
Rattus  TACGCTGGCTTGGGACACCATTTTCAAATCAG 112
Cavia   CACCCTGGCCTGGGACACCATCTTCAACTCAG 112
Homo    CACTCTGGCCTGGGACACCATTTTCAAATCAG 112
```

ENSMUSG00000038567 exon 7 (ORF 2)

```
Mus     TCAAGCCCTGCATCGACCACCGCCTAGAGAGATATTCCCAGCAGCCTGGTGCGGATTTCCTTTGTGATATTTATCAGCAA 80
Rattus  TCAAACCCTGCATCGACAACCGCCTACAGAGATATTCCCAGCAGCCCGGGGCAGATTTCCTCTGTGATATTTATCAGCAA 80
Cavia   TCAAGTCTTGCGTTAATAGCCGATTAGAGAAATATTCTCAGCAGCCTGGCATGGATTTCCTTGGAGATATTTATGACCAC 80
Homo    TCAAAGCTTGTATCGACAACCGGTTAGAGAAGTATTCTCAGCAGCCTAGTGCAGATTTCCTTTGTGACATTTATCACCAG 80

Mus     GATCATCTTTCCAAGAAAGAACTGTACGCTGCTGTCACGGAGCTCCAGCTGGCTGCAGTGGAGACG 146
Rattus  GATCACCTTTCCAAGAAGGAACTGTACGCCGCTGTCACGGAGCTGCAGCTCGCTGCAGTGGAGACG 146
Cavia   AATCAGCTTTCCAAGAAAGAATTGTACGCTGCCATCACGGAGATCCAGCTGGGTGCCATAGAAACT 146
Homo    AATCGGCTTTCAAAGAAAGAATTGTATGCTGCTGTCACAGAGCTCCAGCTGGCTGCGGTGGAAACG 146
```

ENSMUSG00000038567_intron_6

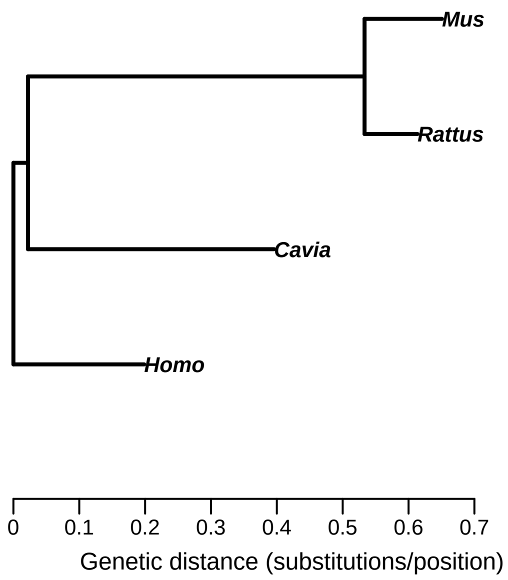

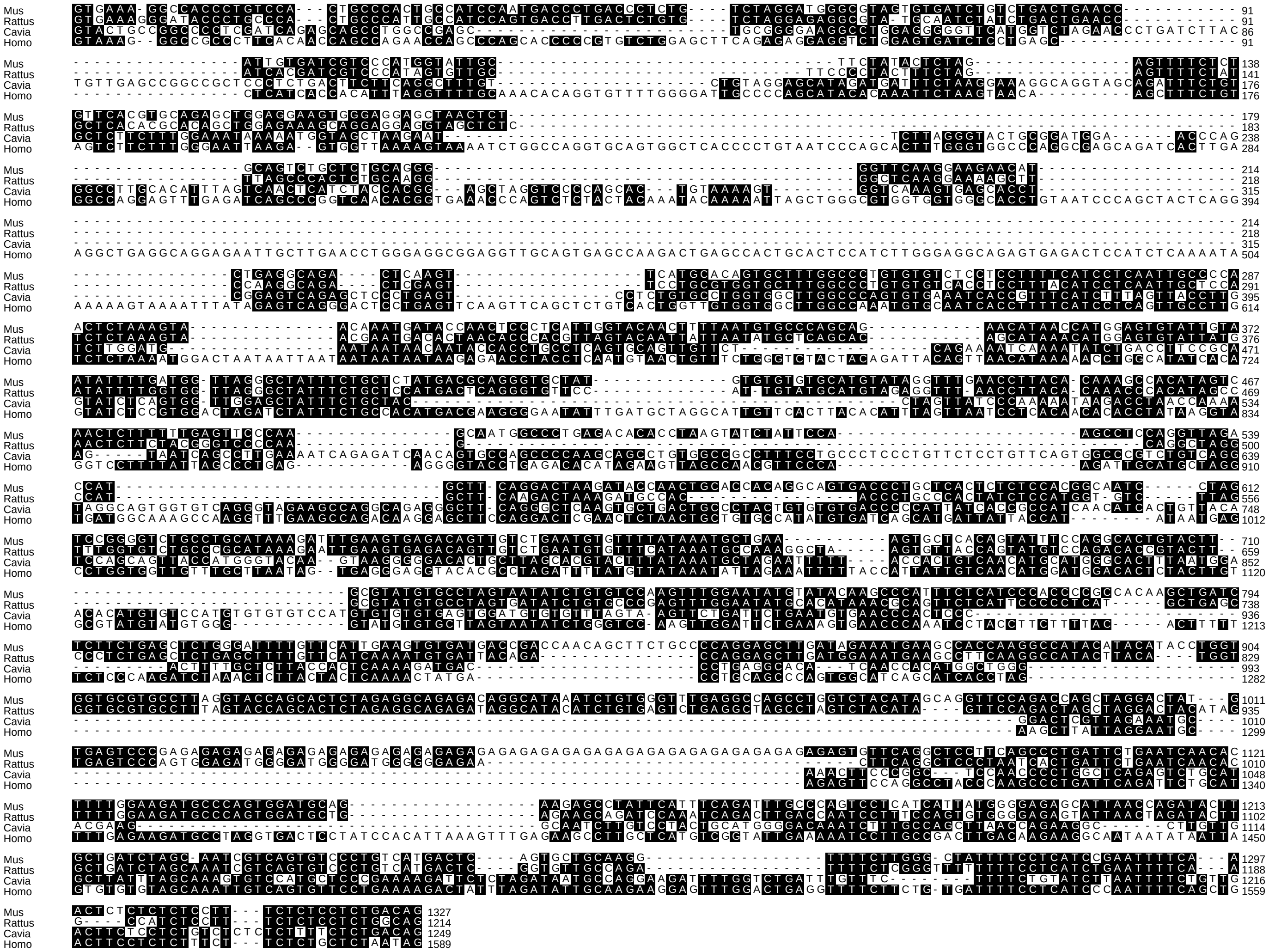
Mus
Rattus
Cavia
Homo

ENSMUSG00000029545 intron 1

Description: Short-chain specific acyl-CoA dehydrogenase, mitochondrial P (Acads)
Intron number: 1
Mouse chromosome: 5
Upstream exon length: 46
Downstream exon length: 164
Mouse intron length: 1496
Intron alignment length: 1828
Total murinae branch length: 0.24276
K_score: 0.06899
Scaling factor: 0.6996

ENSMUSG00000029545 exon 1 (ORF 0)

```
Mus     ------ATGGCTGCCGCCTTGCTCGCCCGGGCCCGTGGCCCTCTCCGTAGAG 46
Rattus  ATGGCCATGGCCGCTGCGTTGCTCGCCCGGGCCGGTGGCTCTCTCGGCAGAG 52
Cavia   CCGCCCATGGCCGCTACGCTGCTCGCCCGGGCCTGCGGCCCCCTCCGCGGAG 52
Homo    ------ATGGCCGCCGCGCTGCTCGCCCGGGCCTCGGGCCCTGCCCGCAGAG 46
```

ENSMUSG00000029545 exon 2 (ORF 2)

```
Mus     CTCTCGGTGTTCGGGACTGGCGACGGTTACACACTGTTTACCAGTCTGTGGAGCTGCCTGAGACACACCAGATGTTGCGT 80
Rattus  CTCTCCGCGCTCGAGATTGGCGAAGATTACATACTGTTTACCAGTCTGTGGAACTACCTGAGACACATCAGATGTTGCGC 80
Cavia   TTCGCCGTCCTCGGGACTGGCAGCAGTTACACACTGTCTACAATTCTGTGGAGCTGCCAGAGACGCACCAGATGTTGCGT 80
Homo    CTCTCTGTCCTAGGGCCTGGCGGCAGTTACACACCATCTACCAGTCTGTGGAACTGCCCGAGACACACCAGATGTTGCTC 80

Mus     CAGACATGCCGTGACTTTGCCGAGAAGGAGTTGGTCCCCATTGCGGCCCAGCTGGACAGGGAGCATCTCTTCCCCACAGC 160
Rattus  CAGACATGCCGGGACTTTGCTGAGAAGGAGCTGGTCCCCATCGCTGCCCAGCTGGACAAGGAACATCTCTTCCCCACATC 160
Cavia   CAGACCTGTCGGGACTTTGCTGAGAAGGAGCTGGTTCCCATCGCGGCTCAGGTGGACAGGGAGCATCTCTTCCCAGCGGC 160
Homo    CAGACATGCCGGGACTTTGCCGAGAAGGAGTTGTTTCCCATTGCAGCCCAGGTGGATAAGGAACATCTCTTCCCAGCGGC 160
```

ENSMUSG00000029545_intron_1

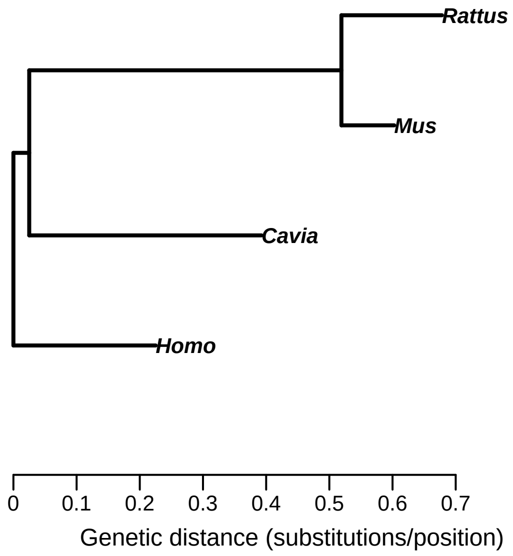

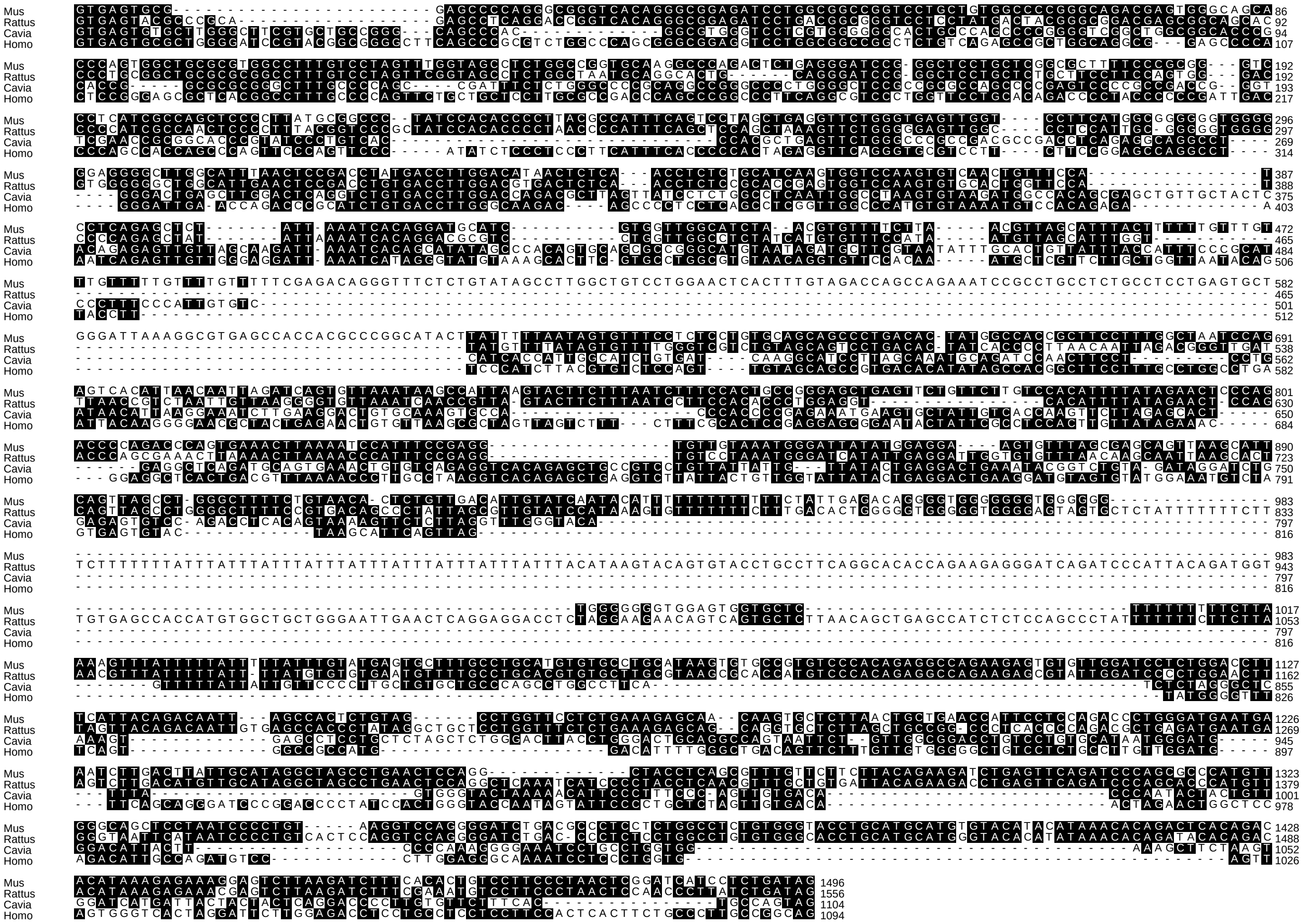

ENSMUSG00000039686 intron 10

Description: Protein zer-1 homolog (Zer1)
Intron number: 10
Mouse chromosome: 2
Upstream exon length: 120
Downstream exon length: 116
Mouse intron length: 472
Intron alignment length: 656
Total murinae branch length: 0.23724
K_score: 0.08848
Scaling factor: 0.70058

ENSMUSG00000039686 exon 10 (ORF 0)

```
Mus     TGTGACCAGGTCATGGAGTTCTCCTGGAGTGCTCTGTGGAACATCACAGATGAGACACCTGACAACTGCGAGATGTTCCT 80
Rattus  TGTGACCAGGTCATGGAGTTCTCCTGGAGCGCTCTGTGGAACATCACGGATGAGACTCCAGACAACTGTGAGATGTTCCT 80
Cavia   TGTGACCAGGTCATGGAGTTCTCCTGGAGTGCCCTGTGGAACATCACAGATGAGACACCCGACAACTGCGAGATGTTCCT 80
Homo    TGTGACCAGGTCATGGAGTTCTCCTGGAGTGCCCTGTGGAACATCACAGATGAAACTCCTGACAACTGCGAGATGTTCCT 80

Mus     CAACTTCAATGGCATGAAGCTCTTTCTCGACTGCCTGAAG 120
Rattus  TAACTTCAACGGCATGAAGCTCTTTCTCGACTGCCTGAAG 120
Cavia   CAACTACAGCGGCATGAAGCTCTTCCTGGACTGCCTGAAG 120
Homo    CAATTTCAACGGCATGAAGCTCTTCCTGGACTGCCTGAAG 120
```

ENSMUSG00000039686 exon 11 (ORF 0)

```
Mus     GAGTTCCCAGAGAAGCAGGAGCTACACCGTAACATGCTAGGGCTCTTGGGGAATGTGGCAGAGGTAAAGGAGCTGCGACC 80
Rattus  GAGTTCCCAGAGAAGCAGGAGCTACACCGGAACATGCTCGGGCTCTTGGGGAATGTGGCAGAGGTGAAGGGGCTGCGGCC 80
Cavia   GAATTCCCAGAGAAGCAGGAACTGCACCGGAACATGCTGGGACTCCTGGGGAACGTGGCAGAAGTGAAGGAGCTGCGGCC 80
Homo    GAATTCCCAGAGAAGCAGGAACTGCATAGGAATATGCTAGGACTTTTGGGGAATGTGGCAGAAGTGAAGGAGCTGAGGCC 80

Mus     TCAGTTGATGACCTCCCAGTTCATCAGCGTCTTCAG 116
Rattus  TCAGCTGATGACCTCTCAGTTCATCAGCGTCTTCAG 116
Cavia   TCAGCTGATGACTTCCCAGTTCATCAGTGTCTTCAG 116
Homo    TCAACTAATGACTTCCCAGTTCATCAGCGTCTTCAG 116
```

ENSMUSG00000039686_intron_10

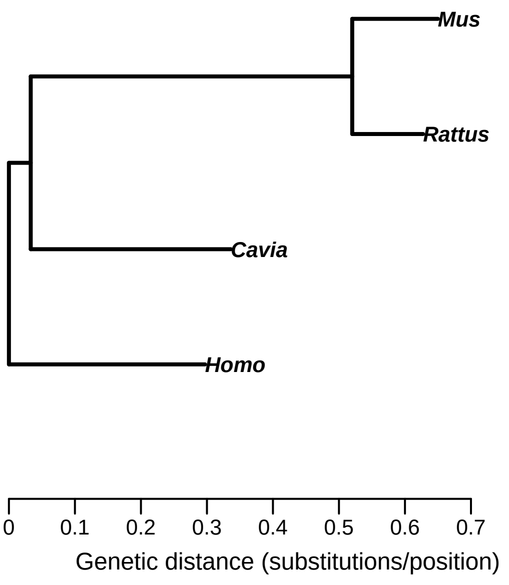

```
Mus     GTAGGCAGTTCCC-----GGCTGCCAC-------------CATCCCACAGGTGCCATCCCACACAAGCCTCCCATGGGC------------------------------- 61
Rattus  GTAGGCAGTGCCCGCACGGGCTGCCA--------------CACACCTAGCATGCCATCCCGCACAAGCTTCCCATGGCC------------------------------- 65
Cavia   GTAGGA----------------------------------CGCTCGGCCCGGCTCACTGCTCCAAGGCTCCCCGTGGCC------------------------------- 45
Homo    GTAGTGTCCACCTCCTGCAGCAGCAGCTGCCTCAGCTGCCCACATGGCCCCTCTT-CTGTCCCTAGGCTCCCCACCAACCAAATCCGCAGCACCCCTCACCCATGCTTCC 109

Mus     ----------------ACACAGGGCCAGCCACACACAGACAATGTCTGCTGCTGAGAGACGTGTCA-----------------GGAACCTCCTCTCAGCCATGCCGTCCA 138
Rattus  ----------------ACACA-GGCCAGCCACACAC--CCCATGTCTGCTGCTGGAAGAAGTGTCA-----------------GGAAGCTTTTCTCAGCTTCACCTCCTG 139
Cavia   -------------------------------------------------------------------------------------------------------------- 45
Homo    TGGAGAAGGGCAGTGGGGACAGACTCAGGCCCACCCACCCAGGATTTGCTGCTGACAGAAGCTCCAGACCCAGATGGCCTGGTGGGGCCTCCTGAGAGCCCCCACTCCCA 219

Mus     GCTGTGCCCCC----------------TGTTCTGTTCTGCCTGCCCACAGACCATTCCCTGCTAACAAGATAAA-----------------ATTCTGCCCAGAATGTAGG 215
Rattus  AGAGTCCTTCTGCCAGGGAACCC----CAACCTGTTCTGCCTGCCCAGAGACCGTCCTCTGCTAACAAGATACA-----------------GTTCTGCCCAGAATATAGG 228
Cavia   ---------------------------CGCTGGGCCCCGACTGCTCA------GTCCTCACCCAGCAGGCTGCT---------------------TCCCTAGGCTACGTC 101
Homo    GGGAAAGCCCCTCCCTGTCTCCTCGGCCGTTGTTCTCAGGCTGCCCACAGCTGGCCAGAGCCCACTACGCTACGAGCTGGACAGCGGGTCTGTTCATCCTTAAACACATG 329

Mus     ACTCTGTTT-TGTTGGTAGCTTCTTAGGGGCTCCCCTAAAAGGACTCTTCTGAGCTGTGGCAAGTGGAGCCACAATCCTCCC--AAGTCAGCTCTCTTCGGGGC------ 316
Rattus  ACTCGGCTTGTTTCTGTAGCTTCTTAGGGGCTCCCCAGAAAGGACTCCTCTGAGCTGTGGCAAATGGAGCTGTAATCATCCCCAAAGTCAGCTCTCTTCCCGGC------ 332
Cavia   CTC---------------GTTCCCGCTGGGACTCCCAGAAGCCAGGCCCTGGTGCT-TGGTAACTAGATCCGAG------CTGGAGCTCAGCTCCCTCC----------- 178
Homo    CTCTGTTTTCCTGCTTTAATTTCTAGGAGGGT-CCCTTAAACTAGTATTTTGAGCTTTGGTGAACAGAACCACGTCTGTCCCATAGGTCAGATCTCTAATCAACCTTTGT 438

Mus     --------------------------------------------------CTCTCTCCTGGTAGCACTGGAGAGTTGGGCTTGGCTTACTGTAC--CTCTCCTTTGTCTA 374
Rattus  --------------------------------------------------CTCTGTCCTGGGAGCACTAGAAAGTCGAGGCCGGCCTACTGTAT--CTCTCCCTTGTCTA 390
Cavia   -------------------------------------TGTGTGCACCAAGCTCTCCC----GGGCCACAGAATCCAGGGGGCAGCCTGTGTGCTGCCTCCCACTATCTA 247
Homo    CTTTGTGGCAAGCAATCCCACCCCTTTAATTCCTTAGTGTGTGTACCCAGCACTGACCTAGGAGCATTGGAGGGTAGGAGGGCAGCATATATGC--CCCCTCTTTGTCTA 546

Mus     GAAGGTCCCTTCACACAGGTGCCCACAAAACCTGCAGGGTAACAGTATCAGACAT-GAAGGGACCAAGTATGAA------GGCCTTGCTGGTTCTCGT-TCCCCAG 472
Rattus  GAAGGGCCCTCCACACAGGCGCCCAC-AAATCCGCAGGATAACAGTATCACGT---GAAGTGA-TGAGTGTGAA------GGCCTTGCCTGCTCTTGT---CCCAG 482
Cavia   ATTGGAAGGGCCAGACAGAAGCCTATAAAACCTCCAGGTTAACAGCACCAGACACTTAAGTGTCTCGGTACTAACTGAGCAGCCCTGTTTGGTCTCGTGTTGCCAG 353
Homo    GTTGGCATGGCCAGAT----GCCCATGAAGCTTTTTGGTTAACAATACCAGATAC-TAAGTCATTAAGTACTAATTGATGCGGCCCATTGGCTCTCCTTTTCCCAG 647
```

ENSMUSG00000033105 intron 16

Description: Lanosterol synthase (Lss)
Intron number: 16
Mouse chromosome: 10
Upstream exon length: 97
Downstream exon length: 106
Mouse intron length: 575
Intron alignment length: 745
Total murinae branch length: 0.14774
K_score: 0.08418
Scaling factor: 0.70173

ENSMUSG00000033105 exon 16 (ORF 0)

```
Mus     TTACTGAGCTTGAGGAATGCTGATGGCGGGTTCGCTACCTATGAGAAGAAGCGTGGGGGTTACTTGCTGGAGCTGCTGAA 80
Rattus  TTGTTGAGCATGAGGAATTCCGATGGAGGGTTTGCTACCTATGAGACTAAGCGTGGCGGGTATTTGCTGGAGCTGCTGAA 80
Cavia   TTACTGAACATGAGGAATCCTGATGGAGGGTTTGCCACCTATGAGACTACACGTGGGGGGCGCTTGCTGGAATTGCTTAA 80
Homo    CTGCTGAACATGAGAAATCCAGATGGAGGGTTCGCCACCTATGAGACCAAGCGTGGGGGGCACTTGCTGGAGCTGCTGAA 80

Mus     CCCCTCAGAGGTCTTTG 97
Rattus  CCCCTCAGAGGTCTTTG 97
Cavia   TCCCTCGGAGGTCTTTG 97
Homo    CCCCTCGGAGGTCTTCG 97
```

ENSMUSG00000033105 exon 17 (ORF 2)

```
Mus     GAGACATCATGATTGACTACACGTATGTAGAGTGTACCTCCGCTGTGATGCAGGCACTGAAGCATTTCCATGAACACTTC 80
Rattus  GAGACATCATGATTGACTACACGTATGTAGAGTGTACCTCCGCAGTGATGCAGGCCCTGAGGCACTTCCGCGAGTACTTC 80
Cavia   GGGACATCATGATCGACTATACATATGTGGAGTGCACCTCAGCAGTGATGCAGGCACTGAAGTGTTTCCACAAGAATTAC 80
Homo    GGGACATCATGATTGACTACACCTATGTGGAGTGCACCTCAGCCGTGATGCAGGCGCTTAAGTATTTCCACAAGCGTTTC 80

Mus     CCAGACTACAGGGCAGCAGAGGTCAG 106
Rattus  CCAGACCACAGGGCTACAGAGATCAG 106
Cavia   CCAGACCACAGGGCGGCAGAGATCAA 106
Homo    CCGGAGCACAGGGCAGCGGAGATCCG 106
```

ENSMUSG00000033105_intron_16

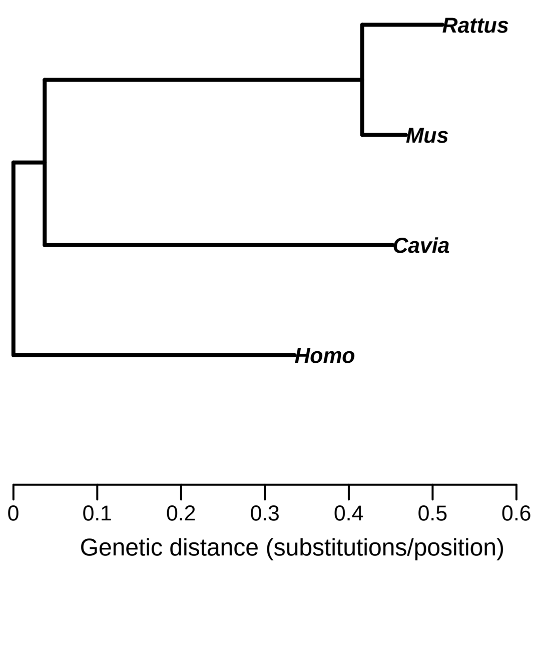

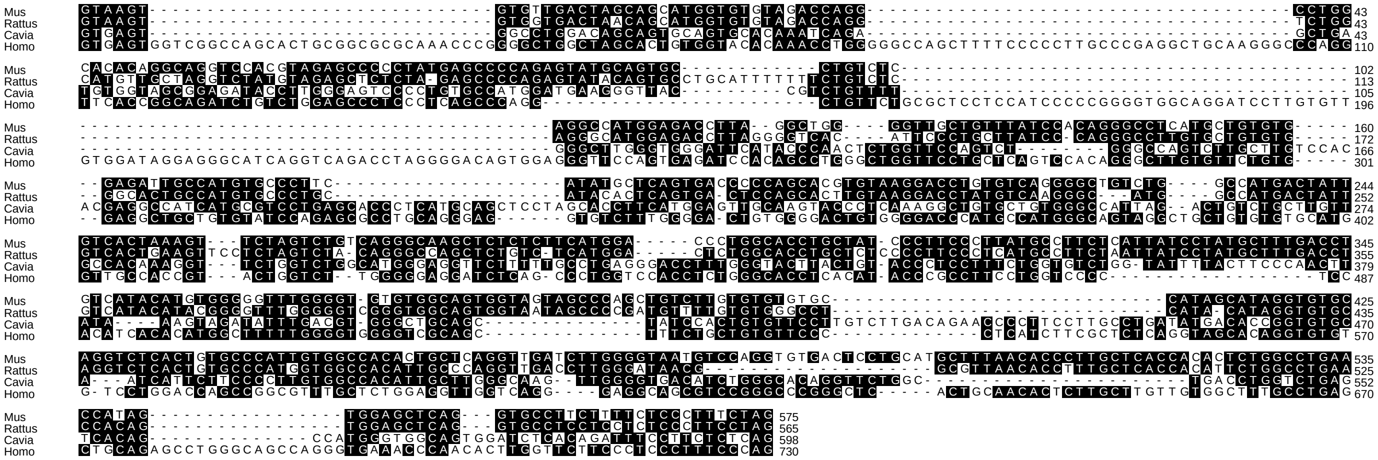

ENSMUSG00000044056 intron 2

Description: EF-hand calcium-binding domain-containing protein 9 (Efcab9)
Intron number: 2
Mouse chromosome: 11
Upstream exon length: 149
Downstream exon length: 177
Mouse intron length: 689
Intron alignment length: 1106
Total murinae branch length: 0.22557
K_score: 0.05093
Scaling factor: 0.70324

ENSMUSG00000044056 exon 2 (ORF 2)

```
Mus     ATGTGCTCTTCTTTCATTTCCTCCAGCACGTGACTAACCTGAACAAGTCACAGATCGGGATGATATTTGACCTCCTGGAC 80
Rattus  ATGTGTTGTTCTTTCATTTCCTCCGCCACGTGACTAACCTGAGAAGCTCACAGATCAAGATGATATTCGACCTCCTAGAC 80
Cavia   ATGTGCTCTTCTATCACTTCCTCAGTTACGTGACTAACTTGAAATCCAGACAGATCAGGATGGTGTTTGACATGTTCGAC 80
Homo    ATGTGCTGTTCTATCACTTCCTTCATCATGTGACTGACTTGAAAAAGGCACAGATCAACATTGTGTTTGACATGCTGGAC 80

Mus     TGGACAGCTGTAGGAGAGATTGGTTTTGACCAGTTCTATGTGTTGATTTGCATACTGCTGGCACATCAG 149
Rattus  TGGACGGCTGTAGGAGAGATCGGTTTTGACCAGTTCTACGTGCTGGTTTGCATACTGCTGGCACATCAG 149
Cavia   TGCAATGCTGTGGGTGAAATTGGCTTCGAGGAATTCTACTTGTTGGTCTGCATCCTGCTGTCACATCAG 149
Homo    TGGAACGCTGTGGGCGAGATCGACTTTGAGAAGTTCTACATGCTGGTGTGCATGCTGCTGGCCCACCAG 149
```

ENSMUSG00000044056 exon 3 (ORF 0)

```
Mus     GACCATCTGGAAGACCACTTTATGTACCGCCATTCCCGGCCAGTCTTTGAGCTGCTTGATCTGGACGGGGAGATGAATAT 80
Rattus  AACCATCTGGAAGATCACTTTATGTACCGCCATTCCCGGCCTGTTTTCGATCTGCTTGATTTGGACGGGGAGTTGAATAT 80
Cavia   AATCATTTGGAAGAACAATTTATGTACTGCCATTCGCGGCACGTCTTTAGACTGCTTGACATAAACGGAGAGCAGAGAAT 80
Homo    AACCATTTGGAAGGACAGTTTATGTATCGTCATTCCCGGCCTGTCTTTGACCTGCTTGACCTGAAAGGGGATCTGAGAAT 80

Mus     AGGTGCGGCCAATTTCCAAAACTACAGATTTCTCTTCAATATTAAAAAGCAGGAACTCCGAGACCTTTTCCATGACTTTG 160
Rattus  AGGTGTGACCAATTTCCAAAACTACAGGTTTCTCTTCAATATTAAAAAGCAGGAACTCCGAGACCTTTTCCATGATTTTG 160
Cavia   TGGTAAAGTCAACTTCCACTTGTACAGATTTCTCTTCAACATTAGAAACGAGCGACTCAAAGAGTTCTTCCAAGATTTTC 160
Homo    TGGTGCAAAAAACTTCGAAATGTACAGATTTCTCTTCAATATTCAAAAACAGGAACTCAAAGATCTCTTCCGTGACTTTG 160
```

ENSMUSG00000044056_intron_2

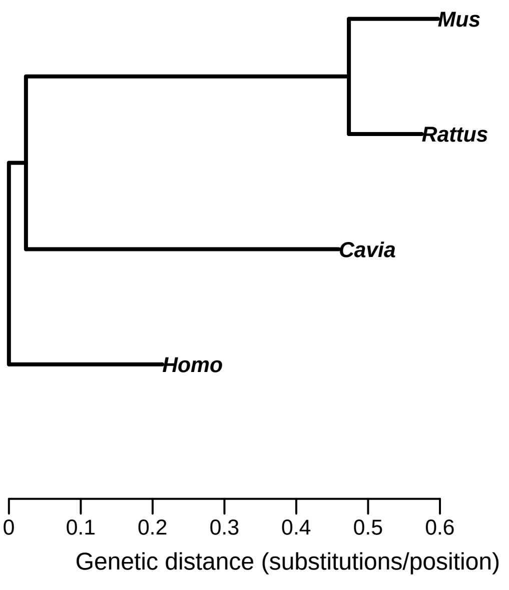

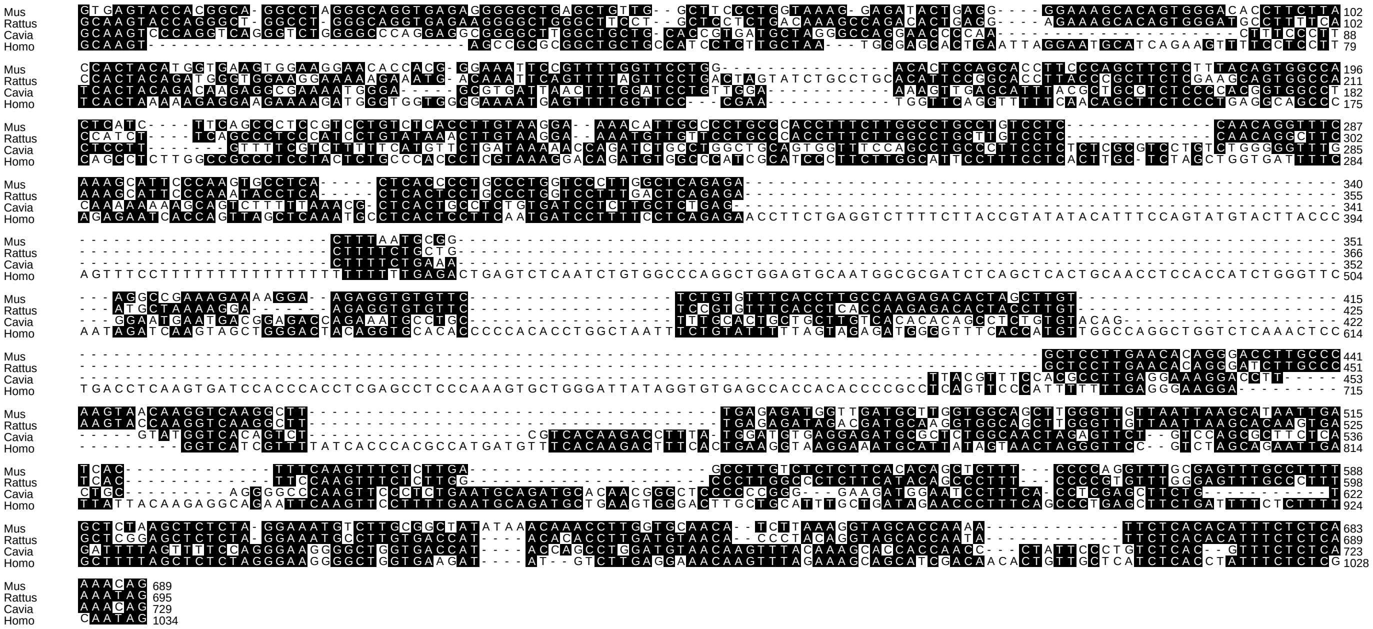
Mus
Rattus
Cavia
Homo

ENSMUSG00000038095 intron 9

Description: Protein strawberry notch homolog 1 (Sbno1)
Intron number: 9
Mouse chromosome: 5
Upstream exon length: 162
Downstream exon length: 136
Mouse intron length: 785
Intron alignment length: 2004
Total murinae branch length: 0.20731
K_score: 0.02838
Scaling factor: 0.7067

ENSMUSG00000038095 exon 9 (ORF 0)

```
Mus     TAAATATGGTAAGATTTCTTCCAAACACAATGGGAGTGTGAAAAAAGGTGTTATTTTTGCTACCTATTCTTCCCTTATTG 80
Rattus  TAAATATGGAAAGATTTCTTCTAAACACAATGGGAGTGTGAAAAAGGGTGTTATTTTTGCTACCTATTCTTCCCTTATTG 80
Cavia   TAAATATGGAAAAATTTCTTCCAAACATAACGGGAGTGTGAAAAAGGGTGTTATTTTTGCTACATATTCTTCTCTTATTG 80
Homo    TAAATACGGAAAAATTTCTTCCAAACATAATGGGAGTGTGAAAAAGGGTGTTATTTTTGCTACTTACTCTTCACTTATTG 80

Mus     GTGAAAGCCAGTCTGGTGGTAAATATAAGACCAGGTTAAAACAGCTTCTACACTGGTGTGGTGACGACTTCGATGGAGTG 160
Rattus  GTGAAAGCCAGTCTGGAGGTAAATATAAGACCAGGTTAAAACAGCTTCTGCACTGGTGTGGTGACGACTTCGATGGAGTG 160
Cavia   GGGAAAGCCAGTCTGGTGGCAAATATAAAACTCGGTTGAAACAACTTCTGCATTGGTGCGGTGACGACTTCGATGGAGTG 160
Homo    GTGAAAGCCAGTCTGGCGGCAAGTATAAAACTAGGTTAAAACAACTTCTGCATTGGTGCGGTGATGACTTCGATGGAGTG 160
```

ENSMUSG00000038095 exon 10 (ORF 0)

```
Mus     ATAGTGTTTGATGAATGTCACAAAGCAAAAAACTTATGTCCTGTTGGTTCTTCAAAGCCAACCAAGACAGGCTTGGCTGT 80
Rattus  ATAGTGTTTGATGAATGTCACAAAGCAAAAAACTTATGTCCTGTTGGTTCTTCAAAGCCAACCAAGACAGGCTTGGCTGT 80
Cavia   ATAGTGTTTGATGAGTGTCATAAAGCAAAAAACTTATGTCCTGTTGGTTCTTCAAAACCCACCAAGACAGGCTTAGCAGT 80
Homo    ATAGTGTTTGATGAGTGTCATAAAGCCAAAAACTTATGTCCTGTTGGTTCTTCAAAGCCAACCAAGACAGGCTTAGCAGT 80

Mus     TTTAGAGCTTCAGAACAAATTGCCAAAAGCCAGAGTTGTTTATGCCAGTGCCACTG 136
Rattus  TTTAGAACTTCAAAACAAATTGCCAAAAGCCAGAGTCGTGTATGCCAGTGCCACCG 136
Cavia   TTTGGAGCTTCAGAACAAATTGCCAAAAGCCAGAGTTGTTTATGCAAGTGCCACTG 136
Homo    TTTAGAGCTTCAGAACAAATTGCCAAAAGCCAGAGTTGTTTATGCTAGTGCAACTG 136
```

ENSMUSG00000038095_intron_9

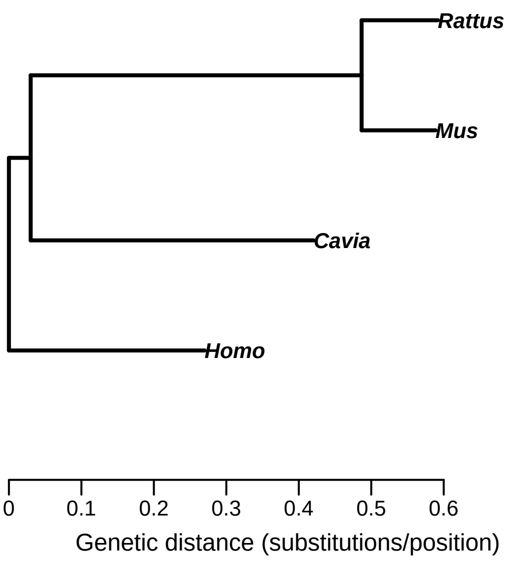

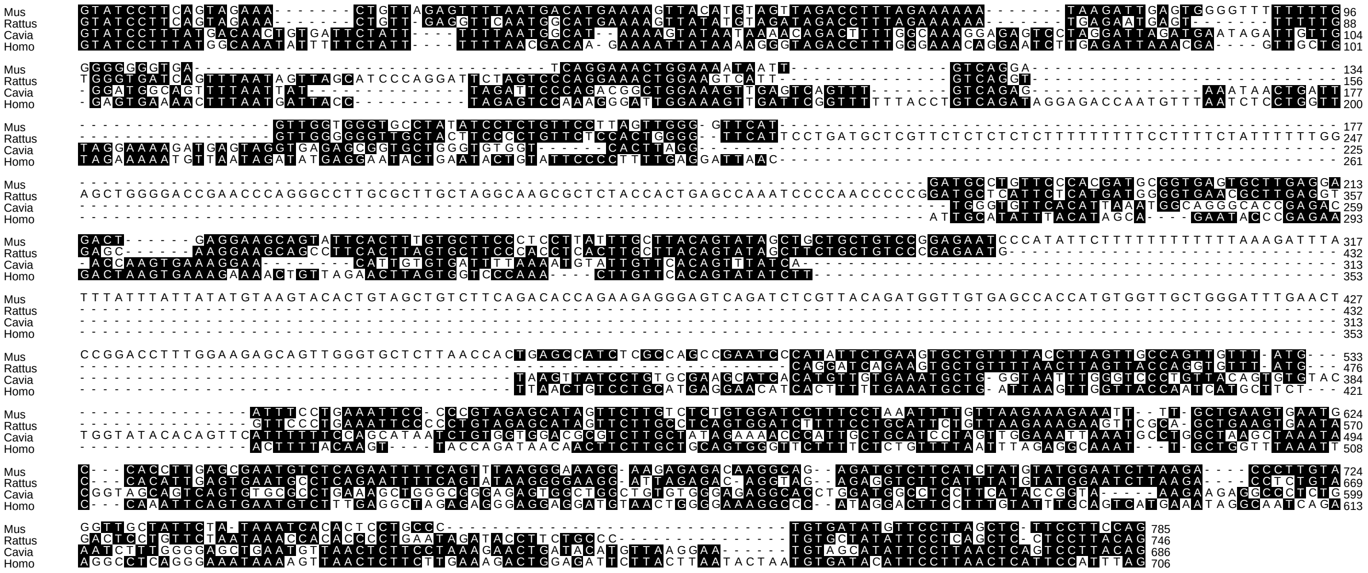
Mus
Rattus
Cavia
Homo

ENSMUSG00000027217 intron 6

Description: Tetraspanin-18 (Tspan18)
Intron number: 6
Mouse chromosome: 2
Upstream exon length: 84
Downstream exon length: 48
Mouse intron length: 1277
Intron alignment length: 2508
Total murinae branch length: 0.19833
K_score: 0.06426
Scaling factor: 0.70827

ENSMUSG00000027217 exon 6 (ORF 0)

```
Mus     GGCTGTTATACAGTGATCCTCAACACCTTCGAAACCTATGTCTACCTGGCCGGAGCCTTTGCCATTGGAGTACTGGCTAT 80
Rattus  GGCTGTTACACTGTGATCCTCAACACCTTCGAAACCTATGTCTACCTGGCTGGAGCCTTTGCCATTGGGGTGCTGGCTAT 80
Cavia   GGCTGCTACACGGTGATCCTCAACGCCTTCGAAACGTACGTCTACCTGGCAGGAGCGCTGGCCATAGGGGTGCTGGCCAT 80
Homo    GGCTGTTACACGGTGATCCTCAACACCTTCGAGACCTACGTCTACTTGGCCGGAGCCCTTGCCATCGGGGTACTGGCCAT 80

Mus     TGAG 84
Rattus  TGAG 84
Cavia   CGAG 84
Homo    CGAG 84
```

ENSMUSG00000027217 exon 7 (ORF 0)

```
Mus     CTCTTCCTCATGGTCTTTGCCATGTGCCTCTTCCGGGGCATCCAGTAG 48
Rattus  CTCTTCCTCATGGTCTTTGCCATGTGCCTCTTCCGGGGCATCCAGTAG 48
Cavia   CTTTTTGTCATGATCTTCGCCATGTGCCTCTTCCGGGGCATCCAGTAG 48
Homo    CTTTTCGCCATGATCTTTGCCATGTGCCTCTTCCGGGGCATCCAGTAG 48
```

ENSMUSG00000027217_intron_6

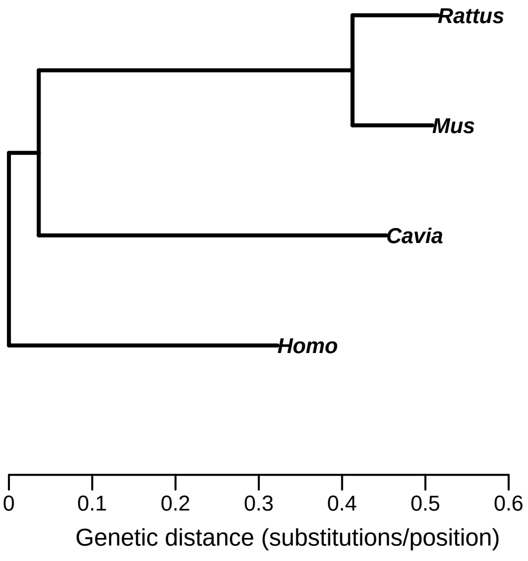

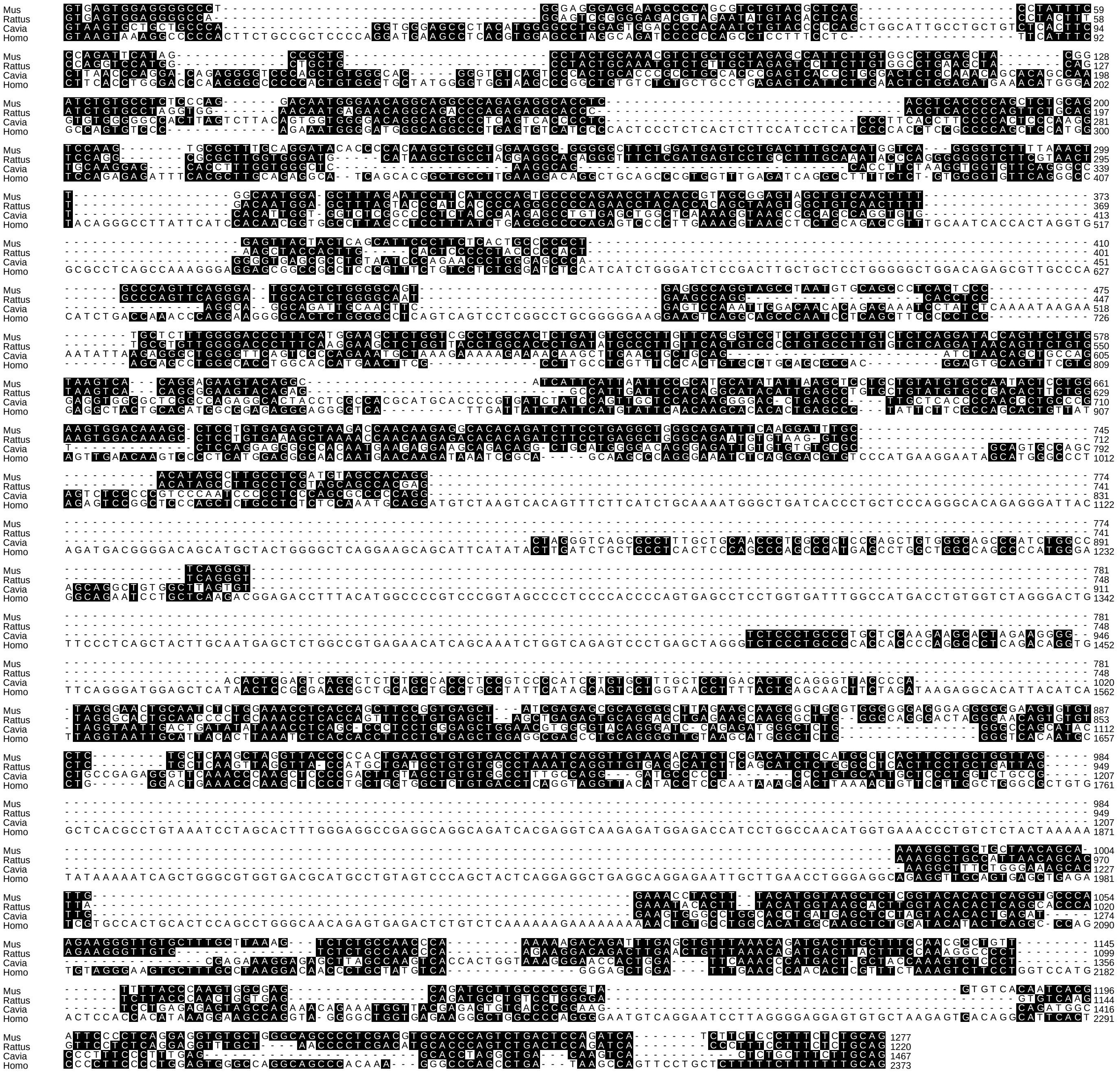

ENSMUSG00000028356 intron 1

Description: Protein AMBP Precursor (Ambp)
Intron number: 1
Mouse chromosome: 4
Upstream exon length: 114
Downstream exon length: 143
Mouse intron length: 1215
Intron alignment length: 1801
Total murinae branch length: 0.14975
K_score: 0.07321
Scaling factor: 0.71127

ENSMUSG00000028356 exon 1 (ORF 0)

```
Mus     ATGCAGGGTCTCAGGACCCTGTTCCTGCTGCTGACTGCCTGCCTCGCTTCGAGGGCTGACCCTGCGTCAACACTGCCAG- 79
Rattus  ATGCAGGGTCTCGGGGCCCTGTTCTTGTTGCTGACTGCCTGCCTCACTTTGAAGGCTGACAATGTGCCAACACTGCCAG- 79
Cavia   ATGTGGGGCGCTGAGGCCCTGCTGTTGCTGCTGGGCAGCTGCCTGGCCATAAGTGTGAATCCTGTGCTGACGCTGCCAGA 80
Homo    ATGAGGAGCCTCGGGGCCCTGCTCTTGCTGCTGAGCGCCTGCCTGGCGGTGAGCGCTGGCCCTGTGCCAACGCCGCCCGA 80

Mus     --ATATCCAGGTTCAGGAGAACTTCAGTGAGTCCCGG 114
Rattus  --ATATCCAGGTTCAGGAGAACTTCAATGAGGCCCGG 114
Cavia   CGACATCCAAGTGCAGGAGAACTTTGACGAGTCCCGG 117
Homo    CAACATCCAAGTGCAGGAAAACTTCAATATCTCTCGG 117
```

ENSMUSG00000028356 exon 2 (ORF 0)

```
Mus     ATCTATGGAAAATGGTACAACCTGGCGGTGGGATCCACCTGCCCGTGGCTGAGCCGCATTAAGGACAAGATGAGCGTGAG 80
Rattus  ATCTATGGAAAATGGTTCAACCTGGCGGTGGGCTCCACCTGCCCGTGGCTGAGGCGGATTAAGAACAAGATGAGCGTGAG 80
Cavia   ATGTACGGGAAGTGGTACAGCCTGGCTATCGGCTCCACGTGTCCCTGGCTGAAGAGGATCAAGGACAGGCTGAGCGTGGA 80
Homo    ATCTATGGGAAGTGGTACAACCTGGCCATCGGTTCCACCTGCCCCTGGCTGAAGAAGATCATGGACAGGATGACAGTGAG 80

Mus     CACGCTGGTGCTGCAGGAGGGGGCGACAGAAACAGAGATCAGCATGACCAGTACTCGATGGCG 143
Rattus  CACGCTGGTGCTGCAGGAGGGGGCGACAGAAGCCGAGATCAGCGTGACCAGTACTCAATGGCG 143
Cavia   CACCATGGTGCTGGGCAAGGGGGCCACGGAGGCGCAGATGAGCACCACCCGCACCCACTGGCG 143
Homo    CACGCTGGTGCTGGGAGAGGGCGCTACAGAGGCGGAGATCAGCATGACCAGCACTCGTTGGCG 143
```

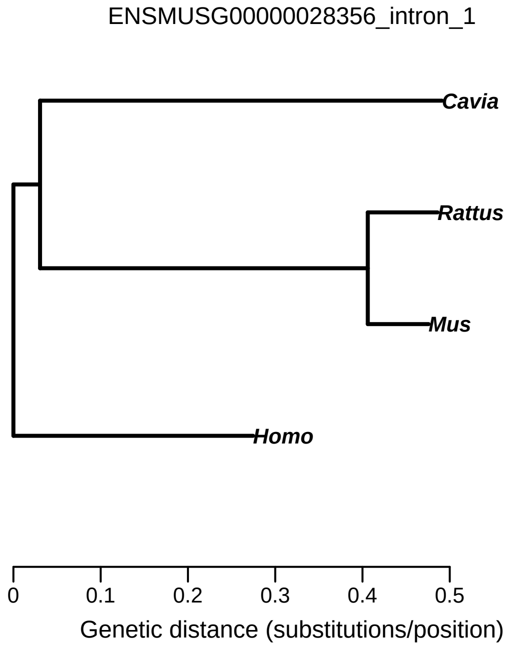

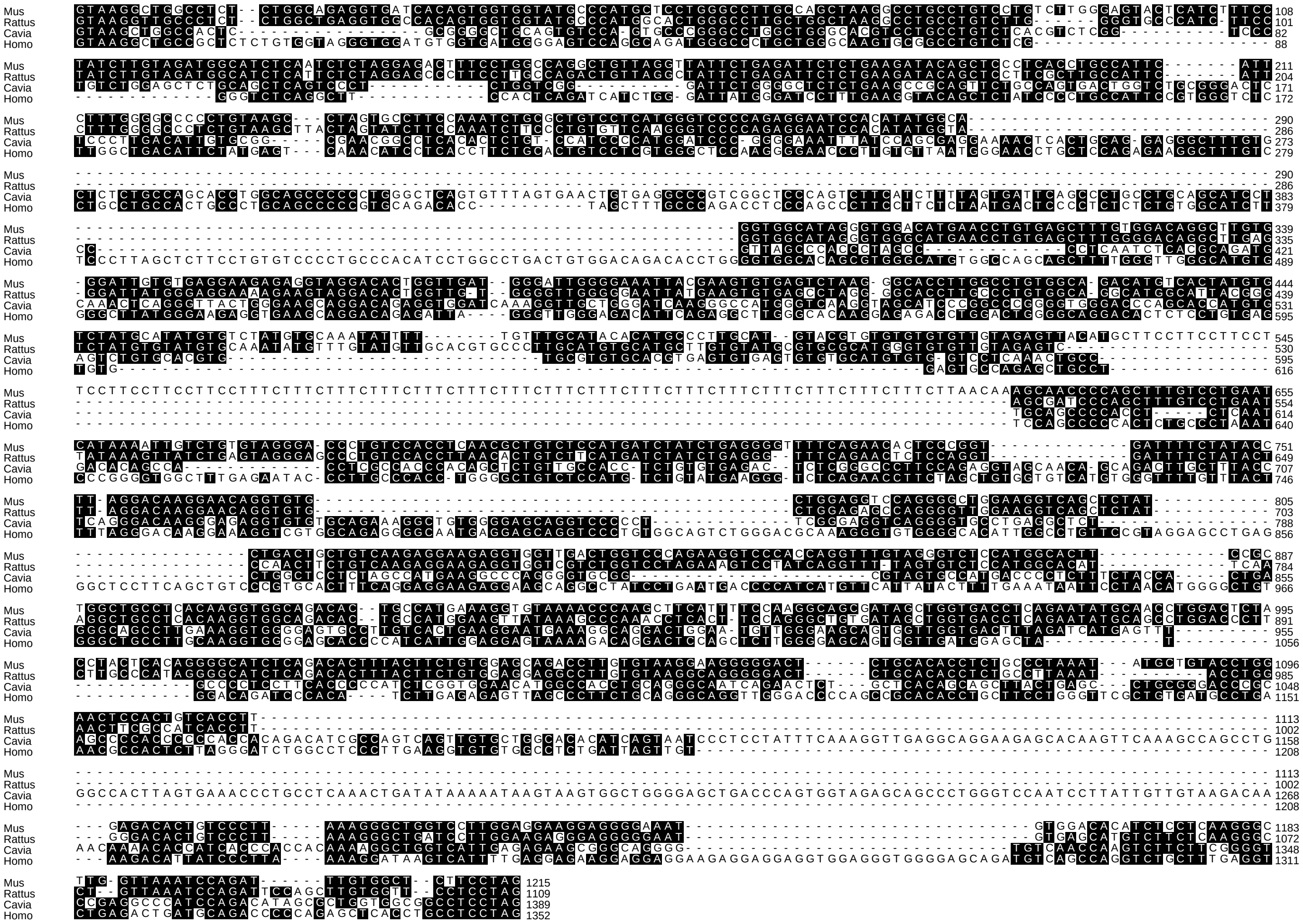
Mus
Rattus
Cavia
Homo

ENSMUSG00000048170 intron 12

Description: UPF0557 protein C10orf119 homolog (Mcmbp)
Intron number: 12
Mouse chromosome: 7
Upstream exon length: 166
Downstream exon length: 134
Mouse intron length: 850
Intron alignment length: 1889
Total murinae branch length: 0.21984
K_score: 0.07074
Scaling factor: 0.71143

ENSMUSG00000048170 exon 12 (ORF 0)

```
Mus     CGTCTACAGATGACAATAGAAAACATGAACCAGTTGAAGCTCATTCCCCACAAAGACTACACAGCCAACCGACTGGTCAG 80
Rattus  CGTCTACAGATGACAATAGAAAACATGAACCAGTTGAAACTCATTCCCCACAAAGACTATACAGCCAACCGACTGGTCAG 80
Cavia   CGCCTACAGATGACTATAGAGAACATGAACCATTTGAAGTTCATTCCCCATAAAGACTACACAGCCAACCGCCTGGTCAG 80
Homo    CGTCTGCAGATGACTATAGAGAACATGAACCATTTGAAATTCATTCCCCACAAAGACTACACAGCCAATCGCTTGGTCAG 80

Mus     TGGCCTCCTTCAGCTGCCCAACAATACTTCCCTGGTCATTGACGAGACTCTTCTGGAGCAAGGGCAGTTGGACACGCCAG 160
Rattus  CGGCCTCCTTCAGCTGCCCAACAACACTTCCCTGGTCATTGATGAGACTCTTCTGGAGCAAGGGCAATTGGACACGCCAG 160
Cavia   CGGGCTCCTCCAGTTGCCCAACAACACTTCGCTCGTTGTTGACGAGACCCTGCTGGAGCAGGGACAGCTGGACTCCCCAG 160
Homo    TGGGCTCCTCCAGCTGCCCAGCAATACTTCCCTTGTAATCGATGAGACTCTCCTGGAACAGGGGCAGCTGGATACCCCAG 160
```

ENSMUSG00000048170 exon 13 (ORF 2)

```
Mus     GTGTTCATAACGTAACTGCCCTGAGCAACCTGATAACCTGGCAGAAGGTGGATTACGACTTCAGCTATCACCAGATGGAG 80
Rattus  GTGTTCATAACGTAGCCGCCCTGAGCAATCTCATAACCTGGCAGAAGGTGGACTATGACTTCAGTTACCACCAGATGGAG 80
Cavia   GTGTTCATAATGTGACTGCCCTGAGCAACCTGATAACCTGGCAGAAGGTGGATTATGACTTCAGCTATCACCAGATGGAA 80
Homo    GTGTTCATAATGTGACAGCCCTGAGCAACCTCATAACGTGGCAGAAGGTGGATTATGACTTCAGCTACCATCAGATGGAA 80

Mus     TTCCCATGCAATATCAATGTTCTCATCACATCTGAGGGAAGGTCACTACTTCCG 134
Rattus  TTCCCATGTAACATCAACGTTCTCATCACATCCGAGGGGAGGTCACTACTTCCG 134
Cavia   TTCCCCTGCAGTATCAATGTTCTCATCACCTCAGAGGGGCGGTCACTCCTCCCG 134
Homo    TTCCCCTGCAATATTAACGTTTTCATTACTTCGGAGGGGAGGTCACTCCTCCCG 134
```

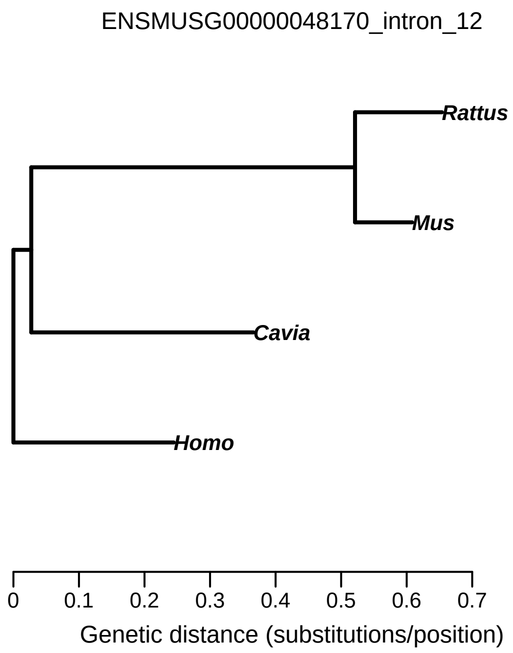

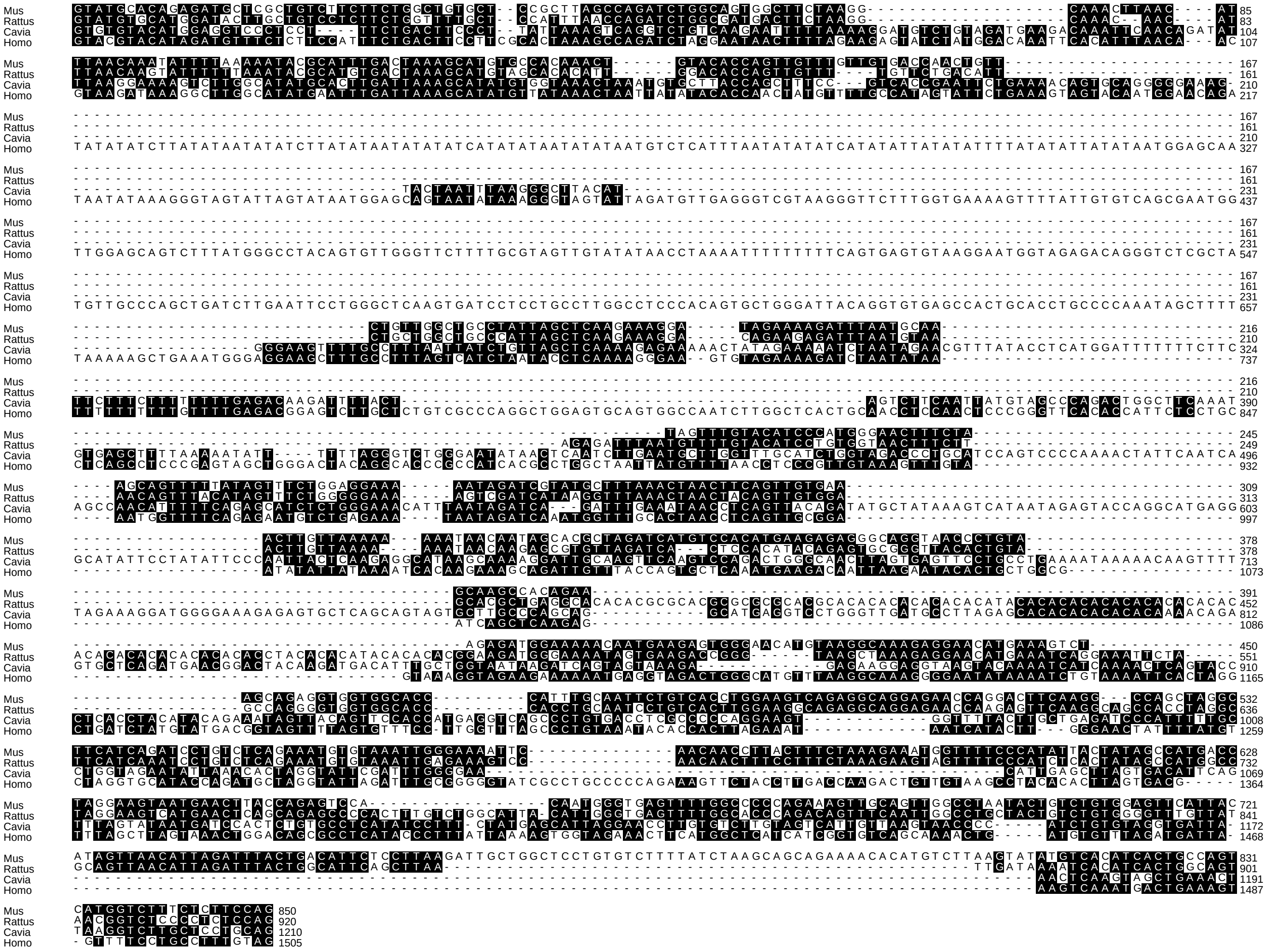
Mus
Rattus
Cavia
Homo

ENSMUSG00000003235 intron 14

Description: Translation initiation factor eIF-2B subunit epsilon (Eif2b5)
Intron number: 14
Mouse chromosome: 16
Upstream exon length: 126
Downstream exon length: 111
Mouse intron length: 555
Intron alignment length: 622
Total murinae branch length: 0.20942
K_score: 0.08515
Scaling factor: 0.7128

ENSMUSG00000003235 exon 14 (ORF 0)

```
Mus     CTGCTCAAAGCCTGGAGCCCTGTTTTAAGGAACTACATAAAGCGTGCAGCTGACCACTTGGAAGCATTGGCAGCCATTGA 80
Rattus  CTGCTCAAAGCCTGGAGCCCTGTTTTTCGGAACTACATAAAGCGCGCAGCTGACCATTTGGAAGCATTGGCGGCCATTGA 80
Cavia   CTGCTCAAAAGCTGGAGCCCCGTTTTTAGGAACTATGTAAAGCGTGCAGCTGACCATTTGGAAGCACTGGCGGCCATTGA 80
Homo    CTGCTAAAGGCCTGGAGCCCTGTTTTTAGGAACTACATAAAGCGCGCAGCCGACCATTTGGAAGCGTTAGCAGCCATTGA 80

Mus     GGACTTCTTCTTGGAACATGAAACTCTCGTTACTTCCATGGCCAAG 126
Rattus  GGACTTCTTCTTGGAACATGAAACTCTTGTTCCTTCCCTGGCCAAG 126
Cavia   AGACTTCTTCCTGGGGCATGAAGCGCTCGGAACTTGCATGGCCAAG 126
Homo    GGACTTCTTCCTAGAGCATGAAGCTCTTGGTATTTCCATGGCCAAG 126
```

ENSMUSG00000003235 exon 15 (ORF 0)

```
Mus     GTCCTGATGGCTTTCTACCAGCTGGAGATCTTGGCTGAGGAAACAATCTTGAGCTGGTTCAGCCAAAGAGACACAACTGA 80
Rattus  GTCCTGATGGCTTTCTACCAGCTGGAGATCTTGGCTGAGGAAACAATCTTGAGCTGGTTCAGCCAAAGAGACATAACTGA 80
Cavia   GTGCTGATGGCTTTCTACCAGCTGGAGATCCTGGCCGAGGAAACCATCCTGAGCTGGTTCAGCAGCAGGGGCATAACTGA 80
Homo    GTACTGATGGCTTTCTACCAGCTGGAGATCCTGGCTGAGGAAACAATTCTGAGCTGGTTCAGCCAAAGAGATACAACTGA 80

Mus     CGAAGGCCAGCAGTTACGGAAGAATCAACAG 111
Rattus  CAAAGGCCAGCAGTTACGTAAGAATCAACAG 111
Cavia   CAGAGGCCAGCAGCTGCGCAAGAATCAGCAG 111
Homo    CAAGGGCCAGCAGTTGCGCAAGAATCAACAG 111
```

ENSMUSG00000003235_intron_14

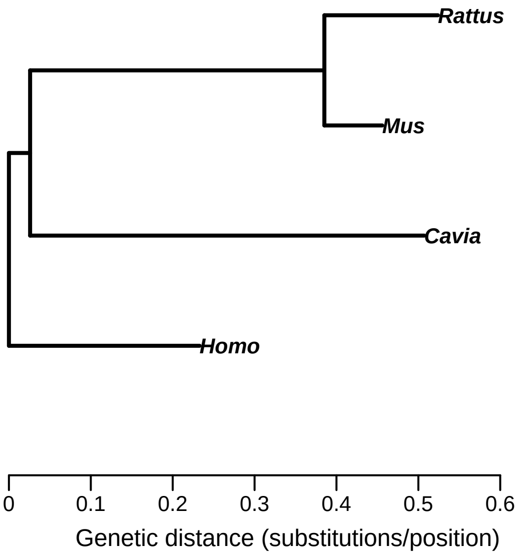

Genetic distance (substitutions/position)

```
Mus     GTGAATTCCATCTCAAGCTTTGTA-TGCTGTGCTTGCCCTGAAACAGGCCTCCATGACCCCCC---ACAGACACAAAACTGAGGTTAACTTGAGCCTAGCATAG-------T 100
Rattus  GTGAACACCATCTCCATCTTCCTA-TCCTGTGTTTGCCTTGACACAGGCCTTCATGACCCCCC---ACAGAGACAAAACCGAGCTTAACCTGAGCCTAGCATAG-------A 100
Cavia   GTGAAAACCACATCAGACCTCATGCTCCTGCATTTGCTTTCAAACCGGCCTAGGTGACCTCC-AGGGAGACTCGAAAGCAAGGACCCCATAACCCCAGCCAAGCTCCATG 109
Homo    GTGAATATGACCTCAAGCCCCATTCTTCTGCACTTGCTTTCAAACGAGGGTTGGTGACCCCCTTGGGAGACATAAAATAGAAGCTAATGTGAATCCAGTATGG-------- 103

Mus     GTGTGTTCACTGCACCGTCTGTCCTTAGGGTT-TACTTACTGCTGGAGTAATGCAGTGCAGAGCAGGCTGATGCTAGTGAGGCAGCCAT---GGGGCAGGAGGCGGCCTG 206
Rattus  GTGTGTTCACTACAGCCTCTGTCCCTGGGATTGTGCTTACTGTTGGAGTAATTCAGTGCAGACCAGGCTGATGCTGGTGAGGCAGCCGTCAGAGGGGGGTGGGGGAGCTG 210
Cavia   CTGGGCTCACAGCCTCATCTGCCCT----ATTGCGCTTATTG-------AATAGGACCCAAGATGGT-----AGGAGCAGGCCGGCTCTTGGGC------ACTGGCCTG 197
Homo    -------------ACCATCCACTCCC---AATATGCTTATTGCTAGAATAGTACAGCTTGGAGCCGG-----ACTAATAAGGTAGCCATCTGGGG-------GGTGGTCTG 186

Mus     AGGATGCACTAAACAGACACTCGGCAGGAGGTCCGTGAGCCATGCACTCCTGGTACTGCAGAGAGTGGTCCACTCCTTTCTTTGTTTTTGGGGGACAGTGATGATACA-- 314
Rattus  AGGATGCACTAAGCAGACACCCAGCAGGAGGTCCACGAGTCATGTGTTCCCAGTACC-----------TCCACTCATGTCTTTGTTTTGGGTGCACAGTGATGATATGAT 309
Cavia   GTGATGTGTGGTGCGCTC-CCCAGCAGTAGTGGCCACAGT------------------------------------TCTCCCTGACCCGTG------------------- 251
Homo    GAAATGTGCAAAGCTCTC-CCCAGCAATAGTTCCAGAAGT------------------------------------TAGACCTGCCCCAGGAGCAGAGCGAACA------ 253

Mus     -------------------GTTTTTGGTTTTTCCCAAATTGGCCTTAAACTTGTTAAGTAGTTCAGTTCGGATTCTTGACCCTC-CGCCTCCACCTCCCAAGTGCTGGAA 404
Rattus  GTTATGACTTTTGGTATATATGTTTGGTTTTTCCCAAACTGGCCTTAAACTTGTTAGGTAGTTCAGTTTGGATTCCCAAGCCTCATGCCTCCACCTCCCAAGTGCTGGAA 419
Cavia   -------------------------------------------------------------------------------------------------------------- 251
Homo    -------------------------------------------------------------------------------------------------------------- 253

Mus     TTACAAGCATGTACCACTATGCCCACTCTCTTGTCTGTCTTGAA-GAGGCTTCTTCATATGTGGTAGG---------------------AAGATGATAAGGTCGT---CT 489
Rattus  TTAGAAGCAGGTA---------CCACTCTCTTGTCTGTTTTGAA-GAGGCTTCTTAATATGTGTGTGGTAGGAAGCTGAGAGCCTGGTAGAGATGCTAAGGTCTT---CT 516
Cavia   -------------------------------TGTACCTGGAGGACACGGCTGCTCCATGCATGAGATG-----TGCTGAGGGCCCAGT-GTGGTACCAGGGTCCTGCCAC 324
Homo    ----------------------GCTCACTCTTGTCCGTTTTGGAGGAGGCTACTCAGTGCATAGAGGG-----ATCTGAGGGCCTGGT-AGAGTAT-------------- 321

Mus     CTTACATTATCCATCAT----CCTTGCTTAGATAGAGGGATCCTGAGACTCCCCTTTGCTGAC--TCTGTAG 555
Rattus  CTTATCCATTATACGATTGTATCCTTGTTAGATAGAGGGATCTTGAGACTACCTTTTGCTGACTCTCTGTAG 588
Cavia   CTCTCCCCATTAGCCATTGTTCCTCCCGCACTCGGCAGAGCCCTGAG--TCCCTCTTTGCGGCCGTCTGCAG 394
Homo    ---------------------CCTGCTGGACTTTCCAAGGCCCCTGAGGTCCCCTTTATTGGCCTTTTGCAG 372
```

ENSMUSG00000036244 intron 8

Description: TBC1 domain family, member 21 (Tbc1d21)
Intron number: 8
Mouse chromosome: 9
Upstream exon length: 101
Downstream exon length: 117
Mouse intron length: 727
Intron alignment length: 1059
Total murinae branch length: 0.15875
K_score: 0.07153
Scaling factor: 0.71304

ENSMUSG00000036244 exon 8 (ORF 2)

```
Mus     AAGGGAAGGGCTCAGGGGCTGTGCAGTCCCTCTTCCCGTGGTTCTGCCTTTGCTTCCAGCGTGCCTTCAAAACCTTCGAT 80
Rattus  AAGGAAAGGGCTCGGGAGCTGTGCAGTCCCTCTTCCCATGGTTCTGCCTTTGCTTCCAGCGTGCCTTCAAATCCTTCGAT 80
Cavia   AAGGGAAGGGCGCGGGGACAGTGCAGGCCCTCTTCCCTTGGTTCTGCCTCTGCTTCCAGCGTGCCTTCAAAACCTTTGAT 80
Homo    AAGGGAAGGGTGCAGGGGCTGTGCAGTCCCTCTTCCCCTGGTTCTGCTTCTGCTTCCAGCGTGCCTTCAAGTCCTTCGAT 80

Mus     GATGTCTGGAGGCTCTGGGAG 101
Rattus  GATGTCTGGAGGCTCTGGGAG 101
Cavia   GACGTCTGGCGGCTCTGGGAG 101
Homo    GATGTCTGGAGGCTCTGGGAG 101
```

ENSMUSG00000036244 exon 9 (ORF 0)

```
Mus     GTCCTGCTGACGGGGAAGCCCTGCAGAAACTTCCAGGTGCTGGTGGCCTACAGCATGCTACAGATGGTGCGCGAGCAGGC 80
Rattus  GTCCTGCTGACAGGGAAGCCCTGCAGGAACTTCCAGGTGCTGGTGGCCTACAGCATGCTACAGATGGTGCGGGAGCAGGC 80
Cavia   GTCCTGCTGACCGGGAAGCCCTGCCGGAACCTGCAGGTACTCGTGGCCTATAGCATGCTGCAGATGGTGCGAGAGCAGGC 80
Homo    GTTCTGCTGACGGGGAAGCCCTGCAGGAACTTCCAGGTGCTGGTGGCCTACAGCATGCTGCAGATGGTGCGGGAGCAGGT 80

Mus     GCTTCTGGAGTGTATGAGCGGTGACGCCATTCTCATG 117
Rattus  GCTGCTGGAGTGTATGAGCGGTGACGCCATTCTCATG 117
Cavia   ACTGCTGGAGAGCATGAGTAGTGATGCCATTCTCCTG 117
Homo    GCTGCAGGAAAGCATGGGCGGGGATGACATCCTCCTG 117
```

ENSMUSG00000036244_intron_8

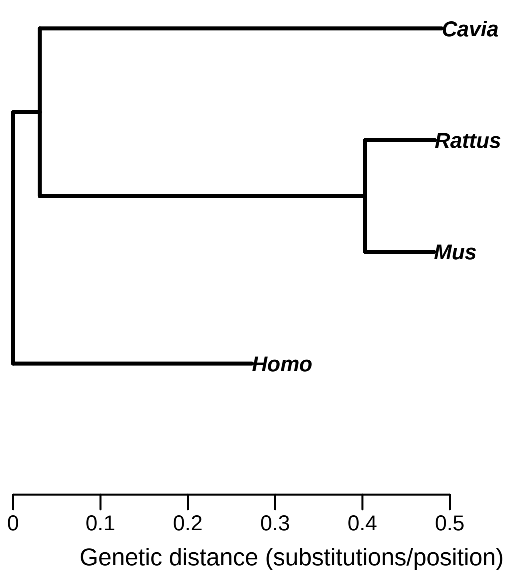

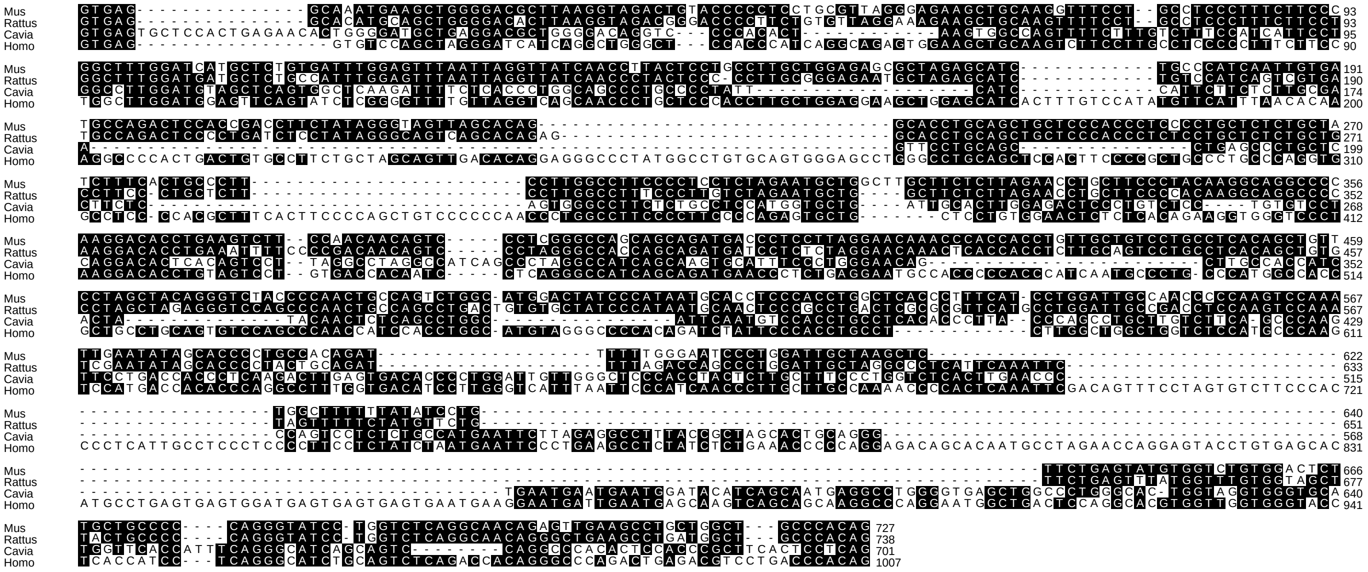

Mus
Rattus
Cavia
Homo

ENSMUSG00000034617 intron 5

Description: 5-methyltetrahydrofolate-homocysteine methyltransferase redu (Mtrr)
Intron number: 5
Mouse chromosome: 13
Upstream exon length: 123
Downstream exon length: 154
Mouse intron length: 1354
Intron alignment length: 2767
Total murinae branch length: 0.20088
K_score: 0.07083
Scaling factor: 0.71361

ENSMUSG00000034617 exon 5 (ORF 0)

```
Mus     GAGGAAAACCAAGCATCTGTGCCTTCA---GGGGATCCAAGTTTTCAAGTTCCAATTTCAAAAGCCATTCGGCTGACTAC 77
Rattus  GATGAAAACCAAGCATCTGTGCCACCATCAGTCGATCCAATTTTTCAAGTTCCAATTTCAAAAGCTGTTGAGCTGACTAC 80
Cavia   GAGCAAGGTCAAGCACCTGCGACTTCAGCCGGGGAGCCAGTCTTTCGAGTTCCAGTTTCAAAGGCCGTTCAGCTTACTGC 80
Homo    GAGGAAAGCCAAGTATCTGTGACTTCA---GCAGATCCAGTTTTTCAAGTGCCAATTTCAAAGGCAGTTCAACTTACTAC 77

Mus     AAATGATGCCGTAAAGAGCACTCTGCTGTTGGAACTGGACATTTCA 123
Rattus  AAACGATGCCATAAAGACCACTCTGCTGTTGGAATTGGACATCTCA 126
Cavia   GAGCGATGCCATCAAAACCACTCTGCTGCTGGAACTGGACATTTCT 126
Homo    GAATGATGCCATAAAAACCACTCTGCTGGTAGAATTGGACATTTCA 123
```

ENSMUSG00000034617 exon 6 (ORF 0)

```
Mus     AAAATAGAGTTTTCCCATCAACCTGGAGACTCCTTCAATGTGACCTGTCCAAACAGTGATCGTGAGGTAGAAGAACTGCT 80
Rattus  AAAGTAGAGTTTTCCCATCAACCTGGAGATTCCTTCAATGTGATCTGTCCAAACAGTGGTTCTGAAGTAGAAGACTTGCT 80
Cavia   AAAACAGATTTCTCCTATCAGCCGGGCGATGCCTTCCATGTGATCTGTCCGAACAACGACTCAGAGGTGCAGGCCCTACT 80
Homo    AATACAGACTTTTCCTATCAGCCTGGAGATGCCTTCAGCGTGATCTGCCCTAACAGTGATTCTGAGGTACAAAGCCTACT 80

Mus     CCAAAGGCTGCAGCTGGCTGATAAACGAGCTCACCGTGTCATCTTGAAAATTAAGACGGACACTAAGAAGAAAG 154
Rattus  GCAAAGGCTGCAGCTGGCTGACAAACAAGCACACCGGGTCATCCTGAAAATTAAGATGGACACCAAGAAGAAAG 154
Cavia   CCAGAGACTGCAGCTCGCTGAGCAGCGAGACCACCACGTCCTCTTGCAGGTCAAGGCGGACACCAAGAAGAAAG 154
Homo    CCAAAGACTGCAGCTTGAAGATAAAAGAGAGCACTGCGTCCTTTTGAAAATAAAGGCAGACACAAAGAAGAAAG 154
```

ENSMUSG00000034617_intron_5

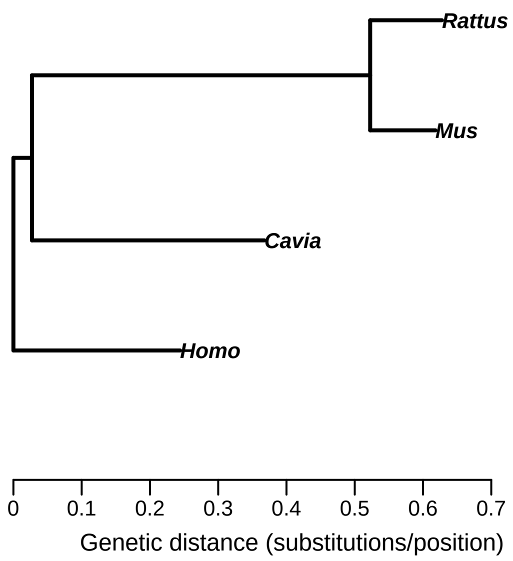

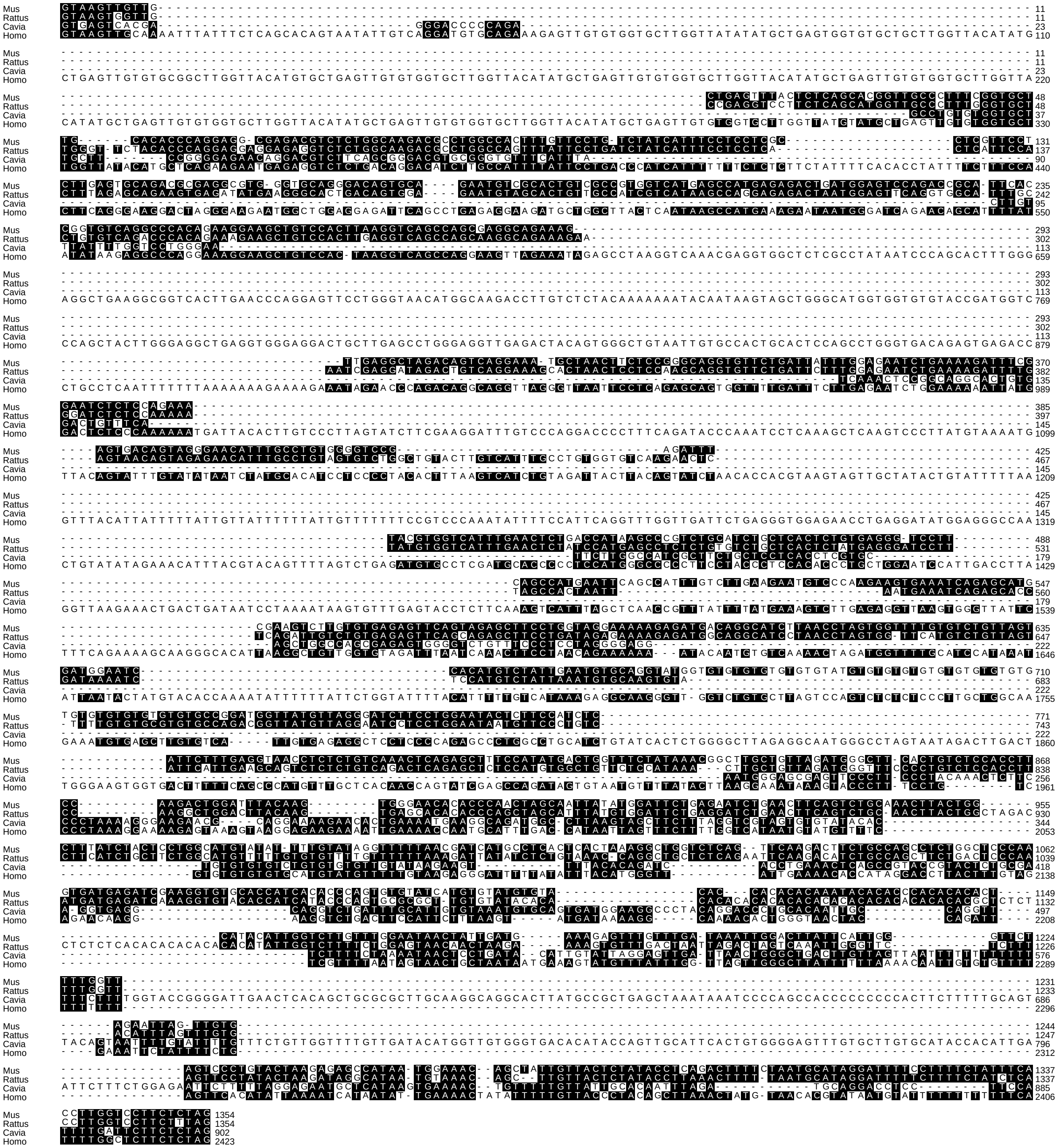

Mus
Rattus
Cavia
Homo
1354
1354
902
2423

ENSMUSG00000042115 intron 3

Description: Kelch domain-containing protein 8A (Klhdc8a)
Intron number: 3
Mouse chromosome: 1
Upstream exon length: 216
Downstream exon length: 102
Mouse intron length: 573
Intron alignment length: 668
Total murinae branch length: 0.12898
K_score: 0.08447
Scaling factor: 0.71475

ENSMUSG00000042115 exon 3 (ORF 2)

```
Mus     CGGTCCTGGACCAAGTTCCCCAACATTCCCTGTAAACGGGCTTTCTCCAGCTTTGTGACCCTGGACAACCACTTATACAG 80
Rattus  CGGTCCTGGACCAAGTTCCCCAACATTCCCTGTAAGCGGGCCTTCTCCAGCTTTGTGACCCTGGACAACCACTTGTACAG 80
Cavia   CGCTCCTGGACCAAGTTTCCCAACATCCCCTGCAAGCGGGCCTTCTCCAGCTTTGTGACCCTGGACAATCACTTGTACAG 80
Homo    CGCTCCTGGACCAAGTTTCCCAACATTCCCTATAAGCGGGCCTTCTCCAGCTTTGTGACCCTGGACAACCACTTGTACAG 80

Mus     CCTAGGAGGCCTGCGCCAGGGCCGTCTGTACCGGCAGCCCAAGTTCCTCCGGACAATGGATGTGTTCGACATGGAACAGG 160
Rattus  CCTGGGAGGCCTGCGGCAGGGTCGTCTGTACCGGCAGCCCAAGTTCCTCCGGACAATGGATGTGTTCGACATGGAGCAGG 160
Cavia   CTTGGGAGGCCTACGACAGGGCCGGCTTTACCGGCAGCCCAAGTTCCTGCGAACAATGGATGTGTTTGACATGGAACAGG 160
Homo    CCTAGGAGGCCTGCGGCAAGGTCGCCTCTACCGGCAGCCCAAGTTCCTGCGGACGATGGACGTGTTCGACATGGAACAGG 160
```

ENSMUSG00000042115 exon 4 (ORF 2)

```
Mus     GGGGATGGCTGAAGATGGAACGCTCATTCTTCCTCAAGAAACGCCGGGCAGACTTTGTGGCTGGTGGTCTGAGTGGAAGG 80
Rattus  GGGGATGGCTGAAGATGGAACGCTCATTTTTCCTCAAGAAGCGCCGGGCAGACTTTGTGGCTGGTGGCCTGAGCGGACGG 80
Cavia   GGGGATGGCTGAAGATGGAACGTTCATTCTTTCTCAAGAAGAGGCGGGCAGACTTTGTGGCCGGCTCTCTGAGTGGACGG 80
Homo    GGGGATGGCTGAAGATGGAACGATCGTTCTTCCTCAAGAAGCGGCGGGCAGATTTTGTGGCTGGCTCTCTGAGTGGACGG 80

Mus     GTCATAGTGGCTGGGGGCCTTG 102
Rattus  GTCATAGTGGCCGGGGGCCTTG 102
Cavia   GTCATAGTAGCAGGAGGACTTG 102
Homo    GTCATAGTGGCTGGGGGACTTG 102
```

ENSMUSG00000042115_intron_3

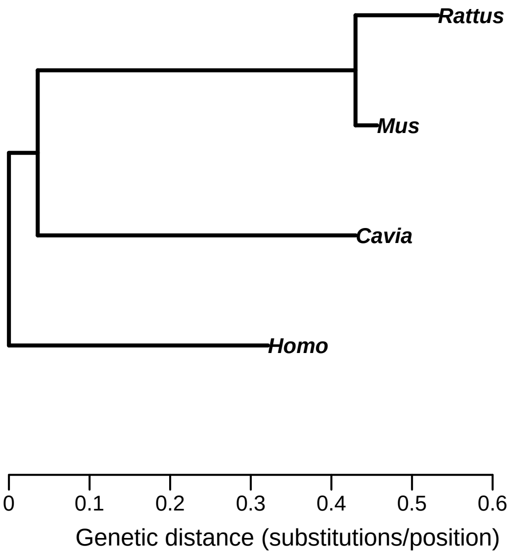

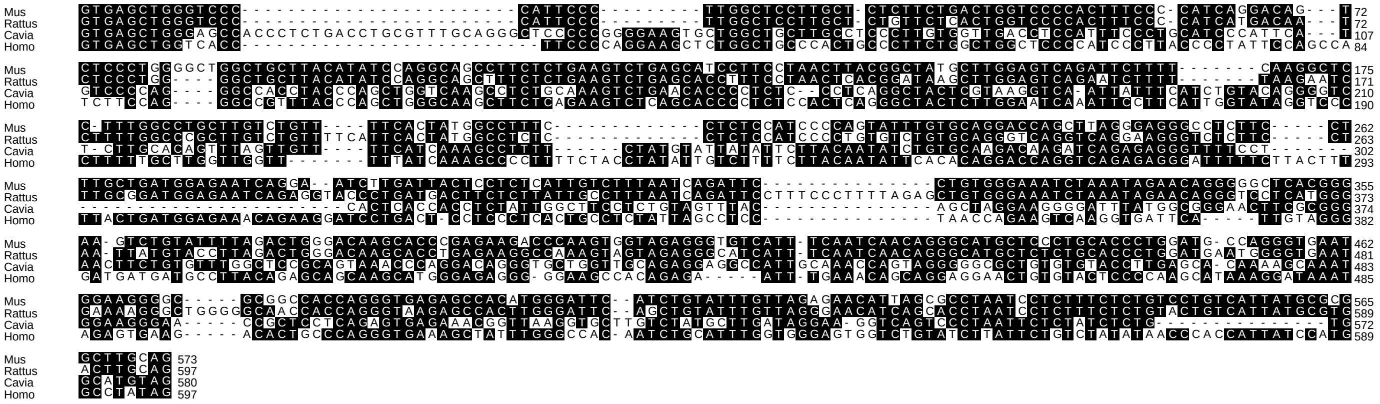
Mus
Rattus
Cavia
Homo

ENSMUSG00000041660 intron 1

Description: Gamma-butyrobetaine dioxygenase (Bbox1)
Intron number: 1
Mouse chromosome: 2
Upstream exon length: 219
Downstream exon length: 115
Mouse intron length: 1557
Intron alignment length: 2500
Total murinae branch length: 0.20839
K_score: 0.03032
Scaling factor: 0.7153

ENSMUSG00000041660 exon 1 (ORF 0)

```
Mus     TTGGCACGATGGGGCAGAGTCCCTCTACCCAGCAGTATGGCTGAGAGACAACTGTCAGTGTTCAGACTGCTACCTACATT 80
Rattus  TTGGCACGATGGGGCAGAGTCCCTCTACCCAGCAGTATGGCTCAGAGACAACTGTCAGTGTTCGGACTGCTATCTACATT 80
Cavia   CTGGGATGACGGTGGAGAGTCCCTGTACCCCGCCGTGTGGCTGAGGGACAACTGCCAGTGCTCCGACTGCTACCTGGATT 80
Homo    CTGGTATGATGAGGAAGAGTCTCTCTACCCAGCTGTATGGTTGAGAGACAACTGTCCGTGCTCTGATTGCTACCTGGATT 80

Mus     CTGCAAAAGCTCGGAAACTTCTCCTGGAAGCTCTTGATGTAAACATTAGAATCGATGATTTGACATTTGACCGGAAAAAG 160
Rattus  CTGCAAAAGCTCGGAAACTTCTCCTGGAAGCTCTCGATGTGAACATTAGAATGGATGACTTGACATTTGACCAGAAAAAG 160
Cavia   CTGCCAAAGCCCGCAAACTTCTCCTGGAAGCCCTCGATGTGAACATCTATATCAAAGGCTTGACGTTTGACAGGAGAAAG 160
Homo    CCGCAAAAGCACGGAAACTTCTAGTGGAAGCTCTTGATGTGAACATTGGAATTAAAGGCTTGATATTTGACAGAAAAAAG 160
```

ENSMUSG00000041660 exon 2 (ORF 0)

```
Mus     GTTTATATCACATGGCCCAATGACCATTACAGTGAATTCGAAGCTAACTGGCTGAAGAAAAGATGCTTTTCTCAACAGGC 80
Rattus  GTTTATATCACTTGGCCCAATGGCCATTACAGCGAATTTGAAGCTAATTGGCTGAAGAAAAGATGCTTTTCTCAGGAGGC 80
Cavia   GTTTACATCACGTGGCCGCACGAGCATTACAGCGAGTTTGAAGCTGATTGGCTGAAGAAAAGATGCTTCTCCCGGCAGGC 80
Homo    GTGTACATCACATGGCCCGATGAGCATTACAGTGAATTCCAGGCTGATTGGCTGAAGAAAAGATGCTTTTCCAAGCAGGC 80

Mus     CAGAGCAAGGCTCCAAGGAGAATTGTTTTTACCAG 115
Rattus  CAGAGCAGGACTCCAAGGAGAATTATTTTTACCAG 115
Cavia   CAGAGCAAAGCTGCAAGGAGAGTTGTTTTTGCCAG 115
Homo    CAGAGCAAAGCTCCAAAGAGAATTGTTTTTTCCAG 115
```

ENSMUSG00000041660_intron_1

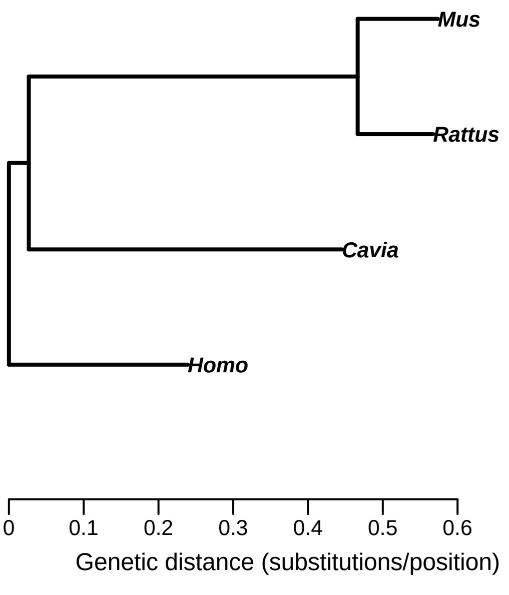

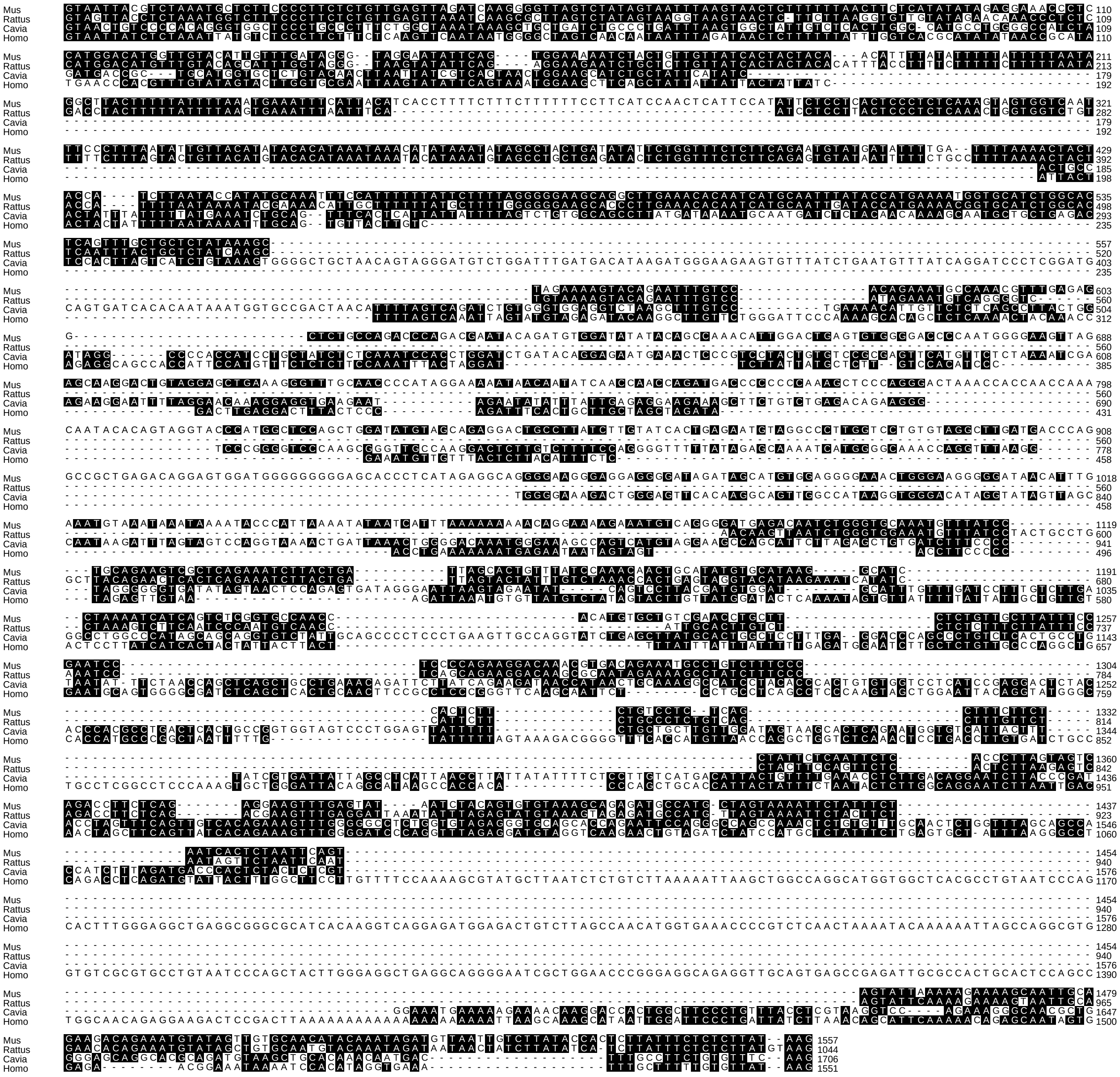
Mus
Rattus
Cavia
Homo
