# Supplementary material for: Development of Rapidly Evolving Intron Markers to Estimate Multilocus Species Trees of Rodents
Source: PLoS One. 2014 May 7;9(5):e96032. doi: 10.1371/journal.pone.0096032 (PMC4012946; doi:10.1371/journal.pone.0096032)
Supplement: Appendix S2 — Final set of 208 introns selected for the phylogeny of closely related mammalian species (part b). (PDF) [file pone.0096032.s007.pdf]

ENSMUSG00000048677 intron 23

Description: Two pore calcium channel protein 2 (Tpcn2)
Intron number: 23
Mouse chromosome: 7
Upstream exon length: 82
Downstream exon length: 83
Mouse intron length: 346
Intron alignment length: 524
Total murinae branch length: 0.11900
K_score: 0.07745
Scaling factor: 0.7166

ENSMUSG00000048677 exon 23 (ORF 1)

```
Mus     GTGGTCAATGGTGTATTTTGTGCTATGGTGGCTGGTGTCCTCTGTTATCTGGATCAACCTGTTTCTGGCTCTTCTTCTGG 80
Rattus  GTGGTCGATGGTGTATTTTGTGCTGTGGTGGTTGGTGTCCTCTGTCATCTGGATCAACCTGTTTCTGGCTCTGCTTCTGG 80
Cavia   GTGGTCCAAGGTCTACTTCGTGCTGTGGTGGCTAGTGTCATCCGTCATCTGGGTCAACCTCTTTCTGGCTCTGCTCCTGG 80
Homo    GTGGTCCAAGATCTATTTTGTATTGTGGTGGCTGGTGTCGTCTGTCATCTGGGTCAACCTGTTTCTGGCCCTGATTCTGG 80

Mus     AG 82
Rattus  AG 82
Cavia   AG 82
Homo    AG 82
```

ENSMUSG00000048677 exon 24 (ORF 0)

```
Mus     AACTTTCTCCACAGATGGGACCCTCAAGGTCATAAGCAGCTCCTTGTTGGGACCAAGC------------AGATGTCTGT 68
Rattus  AACTTTCTCCACAGATGGGACCCTCAAGGTCATAAGCAGCTCCTGGTTGGGACCAAGCAGATCACCTACGAGATGTCTGT 80
Cavia   AACTTCCTCCACAGGTGGGACCCCCAGAATCATGAGCAGCCCTCCGCGGGGACCCCAGAGGCTGTCTACCTGGTGTCGGA 80
Homo    AACTTCCTTCACAAGTGGGACCCCCGCAGCCACCTGCAGCCCCTTGCTGGGACCCCAGAGGCCACCTACCAGATGACTGT 80

Mus     GGAGCTCATGTTCAG 83
Rattus  GGAGCTCATGTTCAG 95
Cavia   GGAGCTCATGTTCAG 95
Homo    GGAGCTCCTGTTCAG 95
```

ENSMUSG00000048677_intron_23

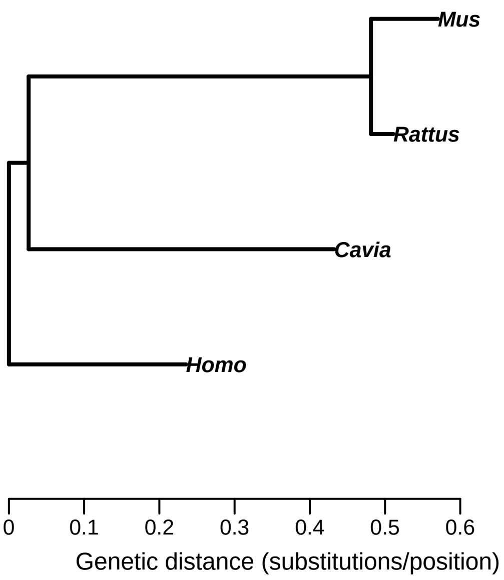

```
Mus     GTACCAGCCGTCCCTGTCCTGTCCAGAC------------------------------------------CTGGGATTGCCTACTGAGTTGTT--GAGAGTTTTGGTGGG 66
Rattus  GTACCAGCTGCC-----CCTGTTCAGGC------------------------------------------CTGAGATTGC-TACTGATTTGTTTATAGAGTTTTGGTGGA 62
Cavia   GTATCAGCAGTC-----CCTTCCTAGGC---------------------------------------------------CTTTCTGGGGCCTCCATGAGGCTCAGGCCAG 54
Homo    GTATCAGAGATC-----CCCACCCAGGCTGTTCTCCATGGTCGTTTGTTTCTAATTCACTCTCGATGGGCCTGAGGCCACCGGCCGGGCCTGCC-TGGAACTTTGATGGG 104

Mus     TGAGC-------CCTGCTAGCCAAGTCTAC---------AGGCTAAAGGCAGCCTGATTGGGGACCTCTGAGCTGCTTGCTCTG-------------------------- 134
Rattus  TAAGC-------CATGCTAGCCAAGACTAC---------AGGCTAAA------------GGGAGCCTCTGAGCTGCTTGCTCTG-------------------------- 118
Cavia   GACATCTGGGTGCCTGGTGGGGGAGGAGCTGGCTGAGTGTGGGTGGGAAGCCCCCTGCCCTGGGGCTTGGTGCTGCTT-TCCTGGCTGCTGGGTCC-------------- 149
Homo    AGAGT-------CGTGCTGGGTTAGGCGGGTCCTGAGTGAGGCTGAG----CCCCTCCCGTGGGGCTCAGTGCTGCCCGCCCTGGCTGCTGGGGCCTTGGGGAGTGCTCC 203

Mus     -------------------------------------------------------------------------------CTGGTCTGTGGGGCTCTCAGGACCCCAAGCT 165
Rattus  -------------------------------------------------------------------------------CTGGTCTTTGGGGTTCTCACGACCCCAACCT 149
Cavia   -------------------------------------------------------------------------------CTTGCATGGGGG------AGGACTCCTGTCT 174
Homo    TGACATCTGGGCTGCTGGGGCCTTGGGGGGTGCTCCTGACATCTGGGCTGCTGGGGCCTTGGGGGGTGCTCCTGACATCTGGGCTGTGGGGATTCTCAGGCCCCCAGGTC 313

Mus     GGCTTAAGGGTGAAGCTGGGCATCTTGCTGTTTCAGGGGTGTG-GCTCTCACAGGCTCTGCATCCAGATGTCCAGGTTGGGCTGTC----CCCCACAGTGACTGTACTGT 270
Rattus  GGCTTAAGGGTGAAGTTGGGCATCTTGCTGTTTCAGGGGCCTG-GATCTCATGGGCTCTGCATCCAGATGTCTGGGGTGGGCTGCT----CCCCACAGTGACTGTACTGT 254
Cavia   TGCTTGTAGGGAGGGA----------------------------GCTGTATTGGGCCCTGT---------------------------------------GGAGACCTGG 217
Homo    GGGCTGAAGGAGGGACTTGGACATTGGCTCCAGGAGGAGCCAGCAGCCTCGTGGGGCCTGTGCCTGGGTGTCCAGGGTGAATGGCTCAGCCCCCTCAGCGGGTGCCCTGT 423

Mus     GACTG---CAGACTGGGAAATGGGGGCTCTGAAGCTGGTATCACATGGG---TACTTACACCCTTTTTCCCTCCTGCT--CCAG 346
Rattus  GACTG---CAGACTGGGCAGTGGGGGCTCTGAAG---------CATGGG---TACTTACACCTTCTCTTCTTCCTGCT--CCAG 321
Cavia   GGCTG--------------GAGAGGGGGGCGAGGTTGATTCCCCATGG----TGTGTCCACTAACTTCCTTTCCTGCTTGCCAG 283
Homo    GGCTGACCCTGGAGGGGCCGGGGATGGGGCAAGGCTGCTTTCATTCGGGGCCCACACTCACTGGCCACTCCTCCTGCTTGCCAG 507
```

ENSMUSG00000030976 intron 2

Description: hypothetical protein LOC73808   (4930404H21Rik)
Intron number: 2
Mouse chromosome: 7
Upstream exon length: 132
Downstream exon length: 81
Mouse intron length: 717
Intron alignment length: 1166
Total murinae branch length: 0.16482
K_score: 0.06807
Scaling factor: 0.71661

ENSMUSG00000030976 exon 2 (ORF 0)

```
Mus     TTCACTCACACTGGTCTGACGGAGAAGACACCAGAATCCATCACGTGTACTTCCCTGAAGGAAACCCACTGCCCATACTT 80
Rattus  TTCACTCACATTGGTGTGATCGAGAAAATACCGGAATCCATCACAAGTACTTCCCTGAAGGAAATCCACTGTCCATACTT 80
Cavia   TTCCCTCATATTGGACTTACACAAAAGACACCAGAATCCCTCATGAGTTCTGTGTTAAAAGAGCCTTATTGTCCGAAGTT 80
Homo    TTCCCTCACATCGGGCTAACGCAAAAGACACCAGAATCCATCACCAGTGCTACGTCAAAAGAGCCCCAGAGTCCACACTT 80

Mus     GTCTGAGCAGGTGGAGAGGAGGCTGCCACCAATCTACAAAATTCGAGAGAAG 132
Rattus  GTCTGAGCAGGTGGAGAAGAGGCTGCCACCAATCTACAAGATCCGAGAGAAG 132
Cavia   GATTCAGCAAGCAGAGGGGAAGCTACCACCAATATACAAAGTCCGGGAGAAG 132
Homo    GCCTCGGCAAGCGGAGGGGAAGCTGCCGCCCATATACAAAGTCCGGGAGAAG 132
```

ENSMUSG00000030976 exon 3 (ORF 0)

```
Mus     CAAGCAGCAAACAAAAACTTCCCTTTCTCTGTGCATGACAACCGCCACAGTTTCCAGAACTCTGGATACTATTTTGACTC 80
Rattus  CAAGCAGCAAACAAAAACTTCCCTTTCTCTGTGCATGACAACCGCCACTGTTTTGAGAACTCCGGATACTACTTTGACTC 80
Cavia   CAAGTAGTGAGTAACAACTTTCCCTTCTCTGGGCATGACAATCGCCACAGCTTAGAGAACTTTGGATGCCATCTTGCCTC 80
Homo    CAAGCAGTGAATAACCAGTTCCCCTTCTCCGTGCATGACAATCGGCACAGCTTGGAGAACTCTGGATGCTACCTTGACTC 80

Mus     T 81
Rattus  T 81
Cavia   T 81
Homo    C 81
```

ENSMUSG00000030976_intron_2

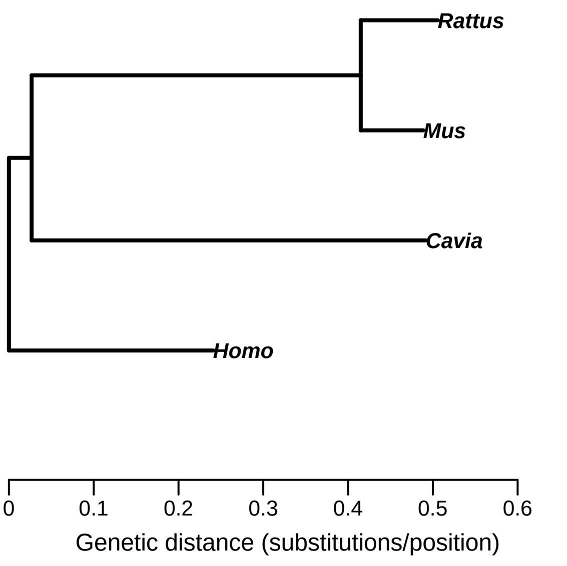

```
Mus     GTGA--CATGAGTATGGGGCCCAGATATAGCATCT-CAGGGCTCTCTGAGGAGGTGCCATGTTGGCCAGATAGCTAACCCATTT-----------------GCCCATCTG 90
Rattus  GTAA--GATGAGTATGGGGCCCAGATATAGGATTTACAGGGCTCTCTGAGGAGGTGCCATTTTGACAAGA---CTAACCCATCT-----------------GTCTATCTG 88
Cavia   GTGAGTCCAGGGCCCTGGCCCCAGCTGCAAACCCCACGGGGACCCTAAGGGGTTAGGACTGTGGTGTCAGTTCTAGAATCAGTTATTCCTTTTAAGGGATGTCTCATTTA 110
Homo    GTGAGTCCTGAACACTGGCCACTGCCATGTGCTCCTGTGGACCTCCCAGGGGGCAGCGCCGCTGGCACGTTGGGCCAAACAATTCTTCTTTGTAAGAAACTGCGCATTTA 110

Mus     CCATACCCA--------------------GGTTCCCAGTGATAGATCCATGTCCTCTGCAGCTACAACTGCCCCCTGATCCAGACAATGAGCTGGGATTCTCCTC----- 175
Rattus  CTATACCCA--------------------GGATCACAGTCATAGACCCACGTCCTCTGCAACTACAGCTGCTTCCTTGTCCAGACAATGATCTGGGATTCTCCACAGTAA 178
Cavia   GCAGCCCTGATGGCATGTACTGATTTGCAGT-TCCCTGCGACAAGCTCAGGCATTCTATAG---AAGCCCTGTCTGGTTCCAGACAATCATCAGGGAGGCTCCTTTACAA 216
Homo    CTATCCTTG--GACCCCTAAGGGTTGGTAGTATCCCCACCCCATACCCTGGTGCTCTGCGGCAGGAACTACTTCCTGCTATGGACAATCA--AGTTAGGCTCCTCCACGA 216

Mus     -------------------------------GTCATTAATAGCCATGGTCTGCCTCCCTTGCCAGCTCCTGAGCTGCTGCTCCAG--CCGTGGTAGTGTGAGGGAGGAGG 252
Rattus  -------------------------CCTTCAGTCATTAACAGCCACGGTCTGCCTCCCTCATCAGCTCCTGGGCAACT-CCCCAG--CCTCTGTGGTGTGAAGGAGGAGG 260
Cavia   GAACTGTGGGTCACAC---------TCTCAGGTCACTGCTAGTTACATTCTGCCTCCCGACCCAGTTCATGGTGAACTTCAGGGGTACTGCAGTGGCTTGACAGAGGAGC 317
Homo    ACAAGAAGCAACACCCCCTTCCAGCCCACAGCTCATTAACATGCACCTCCTGCCTCCCCGGCCAATTCCTGAGCAACT-TCCAGGCCCTGCAGTGGCACAAAGGAGAAGC 325

Mus     CGGAATGGTTTTAGTATCAATATCAACCACACTCACTGTGGGTCTTGACTGAGGCGCTAGGCTTCTGGGAACCCAGCCTCAGGGCTTCTAGGATCAGGCCAGGTTTCCAG 362
Rattus  CAG-ATGGTTTTAATATCAATATCAACCACACTCACTGCAAGCCTTGACTCAGGCTTCAGGCCTCTGGAAACCCAGCCTCAAGATTTTTAGGATCAGGCCAGGTTTCCAG 369
Cavia   AGGTGCGCACCCTGTG-------------------------------------------------------------------------------AGGACAGGGCTC--- 345
Homo    CAGGGTCCATTTCATACCTGCCAGGGTTCCCCCCA------------------------------------------------------GGCCTTGGGCCAGGGCTC--- 378

Mus     GATTTGCTTTCTGCCATCCTTATTAGTCAGATAAGAG-GCTGGCCCCCATGCTGTCAAAAGGTCCTTGCCAGCATTTGGTTCTGTGCATATATGCAGTTTTGCATTCTTG 471
Rattus  GATTTGGTTTCTGCCATCCTTATTAGTCAGATGAG---GTTGGCCCCCATGCTGTC-AAAGGTCCTTGCCAGCATTTTGTTCTGTGCCTATATGCACTTTTGCATCCTTG 475
Cavia   ----TGGTCTCTTTGATCTTTACTACTCAAAGAAGATGGT-----CCCATGGCCTC-AGGAGTCCTTTTTGGC------TTCCAAGAGCCTGTACCCTTTTCCACCCTAT 439
Homo    ---TCATCTCCTGGGACCCTTACTGGCCAGATGACAGAGTTGGTCTACACGGCCTC-AAAAGTCCTTCCCAGTTTTTGGCTCCATGAGCCTGTACACTTTTCCATCCTTG 484

Mus     CCTCCA---AAGACATTTTGAAAGAGATTTTTTTTTTT---------CTTCAGAGATTTGGTA----------------------------------------------- 522
Rattus  CCTCCA---AAGACATTTTGAAAGAGATGTTTTGTTTTTTTTTCCAATTTCAGAGGTTTGGTA----------------------------------------------- 535
Cavia   CCTACAGAGAAGGTGTTCTGAAAGAGAATCTTTTCGAT---------TTTCAGCAAGTTGGTATCTTAGTTACTGCCTTTGTTTCTGGGACAAAATAACCAACACCCAAA 540
Homo    CATCCAAATAAGGTGTTTTGAAAGATAATTTTTATGAC---------TTTGGGGAAGCTGGAA----------------------------------------------- 538

Mus     -------------------------------------------------------------------------------------------------------------- 522
Rattus  -------------------------------------------------------------------------------------------------------------- 535
Cavia   ATTTAAAGAGGAAAGGTTTATTTCCACTAAACTCATAGTTCATAGAAGTTTCAGTATGTATACAGATGGCTGCAAGGCAGGGTGGCGCAGCAGGTGGACATATGCAGGAA 650
Homo    -------------------------------------------------------------------------------------------------------------- 538

Mus     -------------------------------------------------------------------------------------------------------------- 522
Rattus  -------------------------------------------------------------------------------------------------------------- 535
Cavia   AGCTGCTCGTACCGAGTGGACTGGGAGCAGACAGAGTGAAGGAAGGAGCTAGGAAGGGACATAAACCCTCCCAGGTCACACAGAGCTGGTGACCCACCTCTTCTGACTAG 760
Homo    -------------------------------------------------------------------------------------------------------------- 538

Mus     -------------------------------------------------------ACAGGAT--TGTTTGGAAGCCCCGCC--------CATGCTTGCACCCAC------ 561
Rattus  -------------------------------------------------------ATAGGAT--AGTTTGGAAGCCCTGCC--------CATGCCCGCATCTAT------ 574
Cavia   ACCTCACGTCCCAGCAGCAAGTCAGCTATAAGCCCCATAAATCTATTCCCCTCTAATCATATGAGATTTTGGAAGCCAGTTTAGATTTACACCATAACAGTTGTCAGTTA 870
Homo    -------------------------------------------------------ATAAGGTGGGGTTTCAAAGCCCGGCGCTGATGAGCACGTGGATATCCTC------ 587

Mus     ---------------ATCCTGTGCTATTAACTCCA-TGCTAGTGATAGACTTCATTAGCCTCCCTGAGAGTTCCCTTTGTCCCACGCCTCTCCCTTCCCCATCACCGTGC 655
Rattus  ---------------ATCCTGTGCTGTTCACTCCTGTGCTGCTGATAGACTTCATTCGCCACACTGAGAGTTTCCTTTGTCCAGTGCCTCTCCCTTCCCTATCGCTGTGC 669
Cavia   GGTTGTGTTTCAAAGCCCTTAGGTGATACACCCCACGGCTATGTCCTGTGCATAACCATCTTAATAGGAGGTGGCTTGACCCCAC-TGTCTTCTCTCTGGATTGCT---- 975
Homo    ---------------CCCCTCCTGTGTCTGCTCCATCTCTGGGGCCAGGTTTCATT-GTCTTTCTTAGAGGTCGCTTTGCCTCATCCATCTTCCTTCCACATCACCCGAT 681

Mus     TCCGGTTCTGAGGACATCTCGCAGTTCTGACGTGTGCTTATCATCTCTTCATT----CTTCCCCAG 717
Rattus  CCCAGTTCTGAGGACATTTCACAGTTCTGACATGTGCTTTTCATGTCTCTCCT---CATTCTCCAG 732
Cavia   --------------------AGAGTTCTAATGTGTGTCTTCTCATCTCTTTCTTGCTCTTCTCCAG 1021
Homo    CCCAGTTCTGAAAGCAT-GCAGGGTTCTAATGTGTGCTCTTTCCCTTCCATCC--CACTTTTCCAG 744
```

ENSMUSG00000038987 intron 6

Description: Uncharacterized protein C9orf117 homolog (1700019L03Rik)
Intron number: 6
Mouse chromosome: 2
Upstream exon length: 151
Downstream exon length: 167
Mouse intron length: 663
Intron alignment length: 757
Total murinae branch length: 0.25269
K_score: 0.05633
Scaling factor: 0.71742

ENSMUSG00000038987 exon 6 (ORF 1)

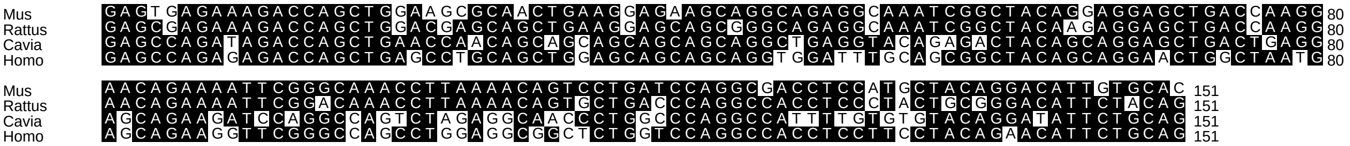

ENSMUSG00000038987 exon 7 (ORF 0)

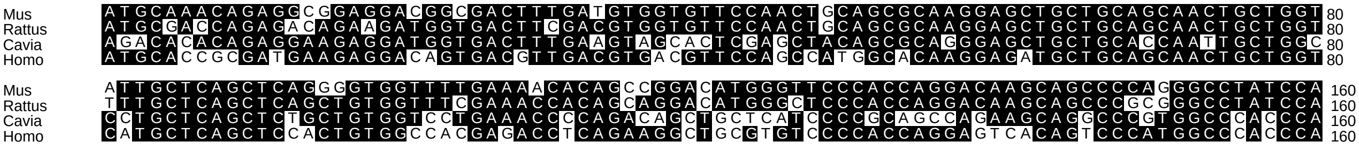

ENSMUSG00000038987_intron_6

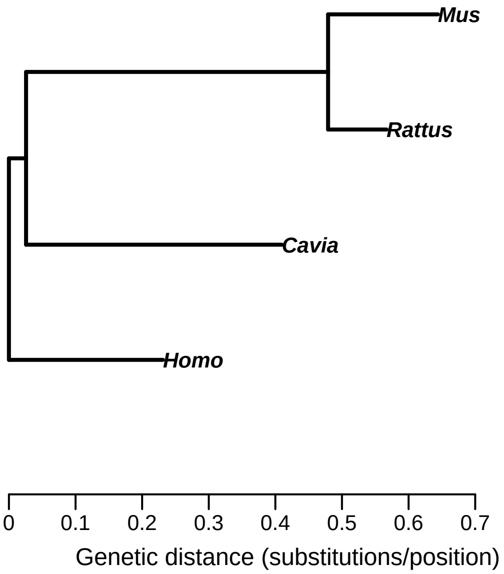

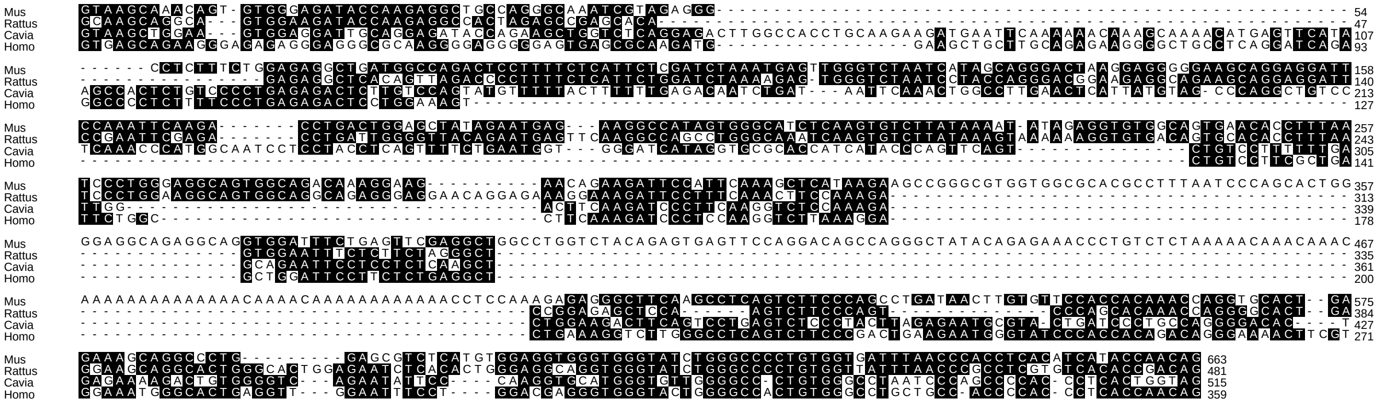

Mus
Rattus
Cavia
Homo

ENSMUSG00000025198 intron 9

Description: Erlin-1 (Erlin1)
Intron number: 9
Mouse chromosome: 19
Upstream exon length: 90
Downstream exon length: 80
Mouse intron length: 1494
Intron alignment length: 1821
Total murinae branch length: 0.22067
K_score: 0.07871
Scaling factor: 0.71836

ENSMUSG00000025198 exon 9 (ORF 2)

```
Mus     AAGCAGAGAAGATTGCACAAGTAGCAAAAATTCGATTTCAACAGAAAGTGATGGAGAAAGAAACTGAAAAACGCATTTCT 80
Rattus  AAGCAGAGAAGATTGCACAAGTGGCAAAAATTCGATTTCAACAGAAAGTGATGGAGAAAGAAACTGAAAAACGCATTTCT 80
Cavia   AAGCAGAAAAGATTGCACAAGTGGCCAAAATTCGGTTTCAGCAGAAGGTGATGGAGAAAGAAACTGAAAAGCGCATTTCT 80
Homo    AAGCAGAGAAGATTGCACAAGTGGCAAAAATTCGGTTTCAGCAGAAAGTGATGGAAAAAGAAACTGAAAAGCGCATTTCT 80

Mus     GAGATTGAAG 90
Rattus  GAGATTGAAG 90
Cavia   GAAATTGAAG 90
Homo    GAAATCGAAG 90
```

ENSMUSG00000025198 exon 10 (ORF 2)

```
Mus     ATGCTGCGTTCCTGGCCCGAGAGAAGGCAAAAGCAGATGCCGAGTATTACGCTGCACACAAATACGCCACCTCAAACAAG 80
Rattus  ATGCTGCGTTCCTGGCCCGAGAGAAGGCGAAGGCAGATGCCGAGTATTATGCTGCGCACAAATATGCCACCTCAAACAAG 80
Cavia   ATGCTGCCTTTTTGGCACGAGAGAAGGCAAAAGCAGATGCTGAGTATTACGCTGCACACAAATATGCCACCTCAAACAAA 80
Homo    ATGCTGCATTCCTGGCCCGAGAGAAAGCGAAAGCAGATGCTGAATATTATGCTGCACACAAATATGCCACCTCAAACAAG 80
```

ENSMUSG00000025198_intron_9

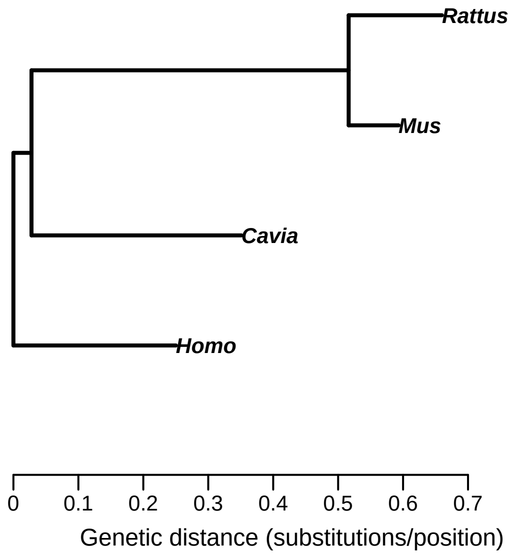

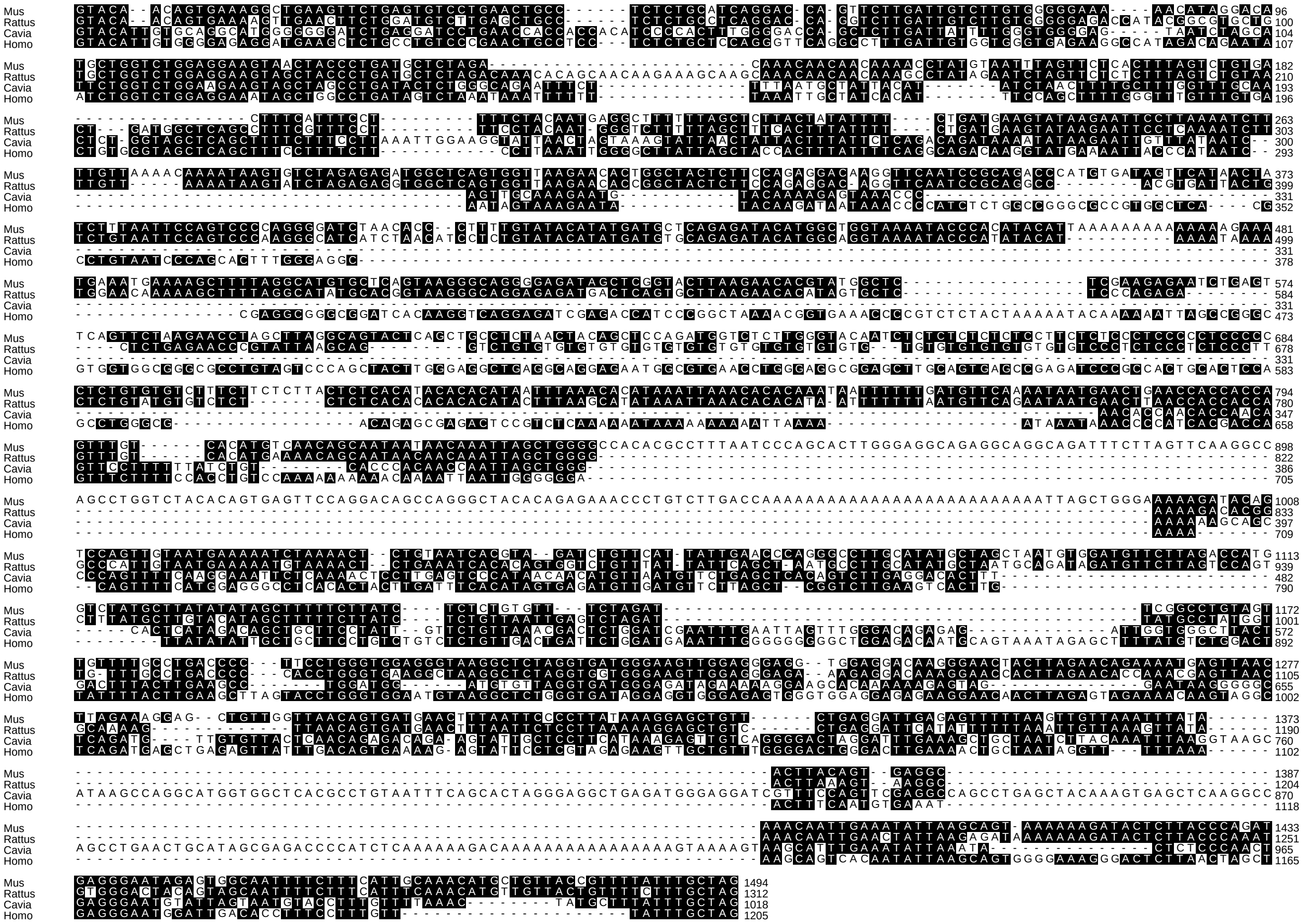
Mus
Rattus
Cavia
Homo

ENSMUSG00000030324 intron 2

Description: Rhodopsin (Rho)
Intron number: 2
Mouse chromosome: 6
Upstream exon length: 169
Downstream exon length: 166
Mouse intron length: 1083
Intron alignment length: 1301
Total murinae branch length: 0.21765
K_score: 0.03375
Scaling factor: 0.71858

ENSMUSG00000030324 exon 2 (ORF 2)

```
Mus     CCCTGTGGTCCCTGGTGGTCCTGGCCATTGAGCGCTACGTGGTGGTCTGCAAGCCGATGAGCAACTTCCGCTTCGGGGAG 80
Rattus  GCCTGTGGTCCCTGGTAGTCCTGGCCATTGAGCGCTACGTGGTGGTCTGCAAGCCCATGAGCAACTTCCGCTTTGGGGAG 80
Cavia   CTCTGTGGTCTCTGGTGGTGCTGGCCATTGAGCGCTATGTTGTCGTGTGCAAGCCCATGAGCAACTTCCGCTTCGGTGAA 80
Homo    CCCTGTGGTCCTTGGTGGTCCTGGCCATCGAGCGGTACGTGGTGGTGTGTAAGCCCATGAGCAACTTCCGCTTCGGGGAG 80

Mus     AATCACGCTATCATGGGTGTGGTCTTCACCTGGATCATGGCGTTGGCCTGTGCTGCTCCCCCACTCGTTGGCTGGTCCAG 160
Rattus  AATCATGCCATTATGGGTGTGGCCTTCACCTGGGTCATGGCGTTGGCCTGTGCTGCTCCCCCACTGGTTGGCTGGTCCAG 160
Cavia   AACCATGCCATCATGGGTGTTGTCTTCACCTGGATCATGGCGCTGGCCTGCGCTGCACCACCCCTTGTCGGCTGGTCCAG 160
Homo    AACCATGCCATCATGGGCGTTGCCTTCACCTGGGTCATGGCGCTGGCCTGCGCCGCACCCCCACTCGCCGGCTGGTCCAG 160
```

ENSMUSG00000030324 exon 3 (ORF 1)

```
Mus     GTACATCCCTGAGGGCATGCAATGTTCATGCGGGATTGACTACTACACACTCAAGCCTGAGGTCAACAACGAATCCTTTG 80
Rattus  GTACATCCCCGAGGGCATGCAGTGTTCATGTGGGATTGACTACTATACACTCAAGCCTGAGGTCAACAATGAGTCCTTCG 80
Cavia   GTACATCCCCGAGGGTATGCAGTGCTCATGTGGGATCGACTACTACACACTCAAGCCTGAGGTCAACAATGAGTCCTTTG 80
Homo    GTACATCCCCGAGGGCCTGCAGTGCTCGTGTGGAATCGACTACTACACGCTCAAGCCGGAGGTCAACAACGAGTCTTTTG 80

Mus     TCATCTACATGTTCGTGGTCCACTTCACCATTCCTATGATCGTCATCTTCTTCTGCTATGGGCAGCTGGTCTTCACAGTC 160
Rattus  TCATCTACATGTTCGTGGTCCACTTCACCATCCCCATGATCGTCATCTTCTTCTGCTACGGGCAGCTGGTCTTCACCGTC 160
Cavia   TCATCTACATGTTCGTGGTCCACTTCACCATCCCCATGATCATCATCTTCTTTTGCTATGGACAGCTGGTCTTCACCGTC 160
Homo    TCATCTACATGTTCGTGGTCCACTTCACCATCCCCATGATTATCATCTTTTTCTGCTATGGGCAGCTCGTCTTCACCGTC 160
```

ENSMUSG00000030324_intron_2

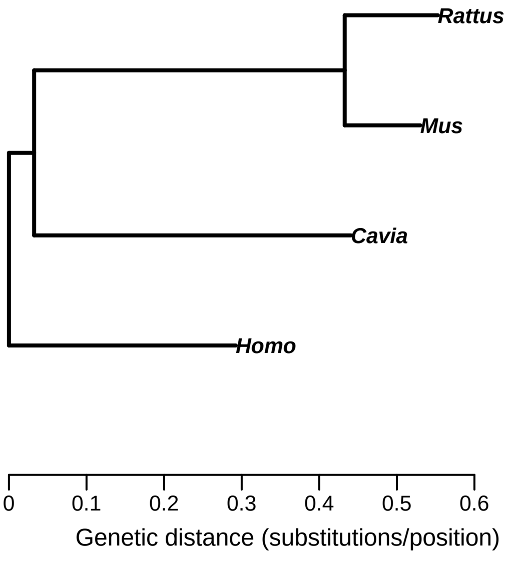

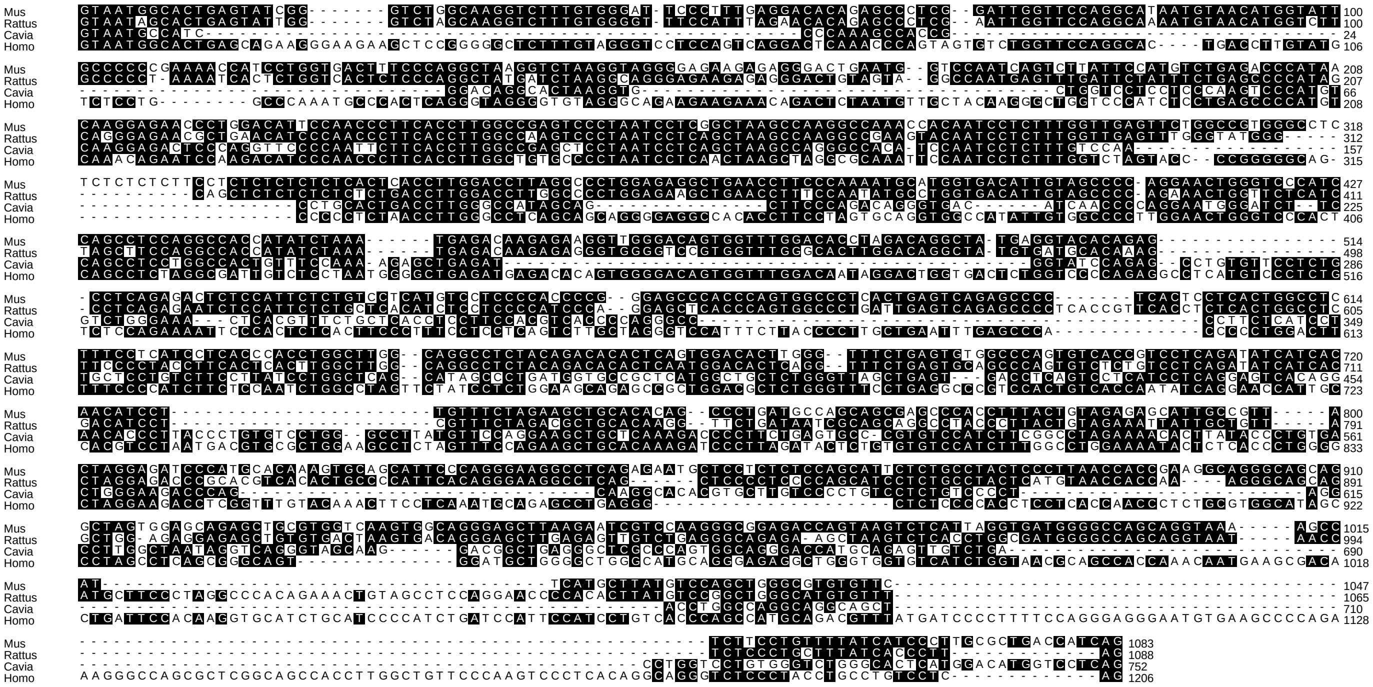
Mus
Rattus
Cavia
Homo

ENSMUSG00000020315 intron 32

Description: Spectrin beta chain, brain 1 (Spnb2)
Intron number: 32
Mouse chromosome: 11
Upstream exon length: 239
Downstream exon length: 174
Mouse intron length: 1014
Intron alignment length: 1299
Total murinae branch length: 0.25878
K_score: 0.0249
Scaling factor: 0.72198

ENSMUSG00000020315 exon 32 (ORF 0)

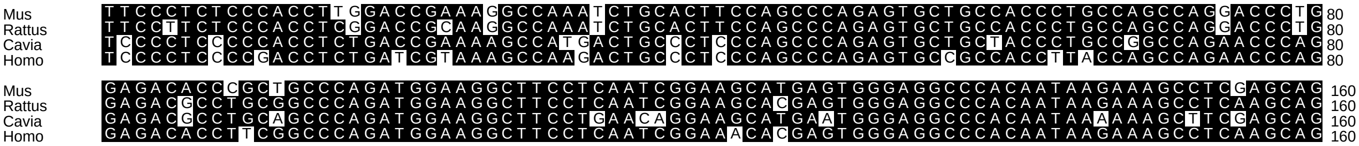

ENSMUSG00000020315 exon 33 (ORF 1)

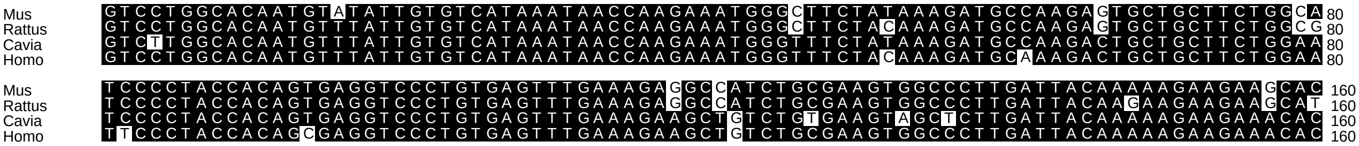

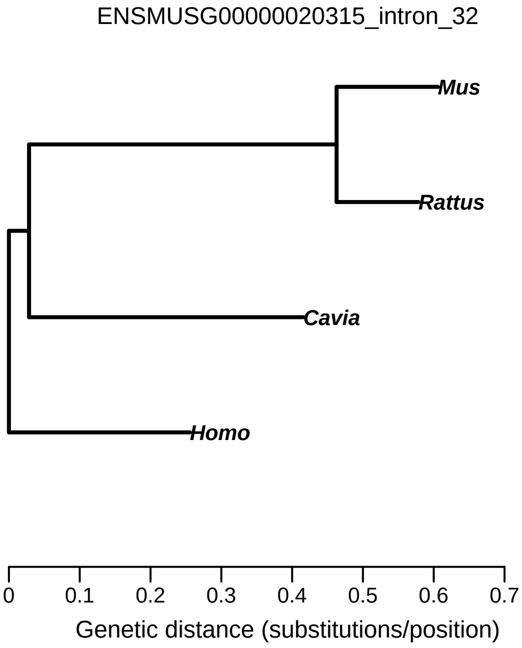

```
Mus    GTAAAGGCAGCA--CGTGAGGAAAGTGGGGAGGGAAATCA-CCCGTGGGAAGGTGGCCCAGGGTCTCGCAGGATTG------------GCATTCAGAAGG---------- 85
Rattus GTAAAGACAGCAGCACAGGGGAAAGAGGGGAGGGAAGTCA-CCCGTGTGGAGGGGCCCAGCGTCATGCAGGATTG------------GCATTCAGAAGG---------- 87
Cavia  GTAACAGCAGCA-CGGGTGGGGCAGAGTGGTGGAAGGTCAGCCTGTACAAAGCCAGGA---------GCCGCACTG------------GGGTCTGGGAGCGTGTGCTTCT 88
Homo   GTAACAGCAGCAGAGGCTGCCACAGTAAGATGGGAAGTCAGCCTGTGAAGGGATAAGGCGGGCCACTCTTGAATTGGAAGAGAAGTATGTGCTCATGTAGTTTATTCCT 110

Mus    ----------TCAGAGCTTGTCATTCTTGTGTGTACAGTGTGTTGAGAATCGGAGTGAGAGTGACACTCAGCTCAGGAGATGTAG-----------CTGGCAGGAATCGC 174
Rattus ----------TCAGAGCTTTTCATTC---TGCGACCAGTGTGTCGAGAAGTGCTCAGTGAGGAG----------AGGAGATGTAG-----------TGGGCCAGAATCGC 163
Cavia  GTGGGTGCATCTGGGGCTTTGCGTTCT--TAGGCCTG--ACATTAGAAATTGTGGGTAGAGTGCTATTTATTCACTGAGAAAAAGA-AGAGAGGCCTGGGTGCGAGCAGC 193
Homo   TTGGTAGCTTCCTGGACATTGCATTCTTCTTGGGCTG--ACGCTGGAAAGCAGGAGTAGAATGCTGGTTATTCACTGAGAGAAGAAGAGTTGAGTTTGGATGGGAGTAGC 218

Mus    AGGAGTGGCCT---GAGGCTTGTCCTGAGGAGGCGCTCACTAGAGCAAAGTTGAACAGTCTCCTGAAGT-----GAAGTGACCTCATTGTAGGGTTCCTAGTAGGAT--- 273
Rattus AGGAATGGCCT---GAGGCTTATCCTGAGGAGGAACTCACTATAGCGAAGTTGAACAATCTCCTACAGT-----GAAGTGACTTCTTTATAGGGTTCCTTATGGGAT--- 262
Cavia  AAGAAGAGACCTTCGTAGTTGATCTCGAGGAGGTGTTCACTAGGCTGAGCCTGAGGGGCTTCCTTGGT-----GAAGAGATTTCACAGTG-------------------- 278
Homo   TAGAAGGGGCTTTAGTAGTTCATCTAGAGAAGGAATTTGCTAGATTGAGACTGAAGAGTCTTCTTTACTTTAAGTGATTGATTTCATT-TAGGACTCCTTGTGGGTTTTT 327

Mus    ------------CCGGCTTTCACAGGTACTCACTCCTCCATATGAGTCTTGAGATG-----ACCATCTCCCACCAGTC-TCTAAGTTTATTATTTAGCTGATGAGATAGT 365
Rattus ------------CCAGCTTTCACAGATGCTCACTTCTCTGTGTGAGTCTCGAGATG-----ACTGTCTCCCACCAGTCTTTTAAGTTTATTATTTAGCTGATGAGAAAGT 355
Cavia  ----------------------------TGCCCGCTTGACCATGTTGATC------------------------------------------------------------ 300
Homo   TGTAAAAAGCCATTAACCATTTCCCCTGCCCTGATCTCCATATCAGCCTTTATATATATTCATTGTCTCTCAACAGCC--CCAATTTTTTATTTAACAGATGGGG-AAC 434

Mus    AGTGACTCTGAGAGGTTGTAAAAAAGCATACTTTTTTAGACATCTAGTAAATAGTAAAACTGAGATGAAAACCACATCTAATTCCAAAGCTGGTACAACTTAGCTGGTCT 475
Rattus AGGTGCTCTGAGAGGTTGT------------------------------------AAACTGAGATGAAAATCACATCTGATTCCAGAGC-------------------- 408
Cavia  ------------GGATCGT-------------------------------------------------------------------------------------------- 307
Homo   AGAGGCTCTGAGCGGTTAA-CAAAGCTTTCTTTCTGTGGAAACCTAGTAAATGGCAAAGCTGGAATATGAA---------ACTGCAAAGC-------------------- 514

Mus    GCCATATGTCTAGATTA-----CTGTACATGTGGAGTGTCTACTGTACA-----AAGGACTTGTGTCTGTGTTGGATAAGG------------------------------ 546
Rattus -CTGTGTGCTTAAATTA-----CTATACACGTGGAGCATCCACCGTACATGTTGGAGGTGTCCACTGTACAAAGGGCTTGG------------------------------ 483
Cavia  -------------------------------------------------------------------------------------------------------------- 307
Homo   -CTATGTGCTTAACTAGTAAACCCAACCACCTGGGACCACCATATCTTACTGTGCAGTTTGTTGCTGTGTGAGGGGATCAGAACATCTAAGAACTGCCATTCACGTTGAG 623

Mus    -------------------------------------TAACGGTCTGATGTTCTCATTAAGTTATCGTATACTGACAGTTTGCCCCTTGGGTATTGTTGGCACA---- 613
Rattus -------------------------------------TTACAGTCTGATGTTCTCGTTAAGTTAACGTATTCTAACGGTATGCCCCTTGCATACTGTTGGCGCA---- 550
Cavia  ----------------------------------------------------------------------------------------------------GGAGAAAGGC 317
Homo   GACAACATAGGGATTTATATATTAATACATGCTGGTGTAATACAGGCCGGTGTTCTGTTAAAATTAGTATATTAGGACAGTTTGCC------ATACCATAGGGGAAAAGC 727

Mus    CACCTGAGTGTAGCTC-CCCAAAGGGATACCATAAATACCAATGGCCCTGGATCTACCCTCTTGTC-AGTAGCTGAACCCCAGAAAGAATGTCACTAAGTCTGGGGGAGA 721
Rattus CACCTGAGTCTAGCTCCCCCGAAGGGATTCCACAAACACTAATGACCCTGGATCTACCCTCTTGTC-AGTA-CTGCACCACAAAAAAACAGTCACTAAGGCTGGGGGAGA 658
Cavia  TGCCTTAGTCTGATTCTCTTACATGAAGA-----------------------------------------ATGAGATGCGTAAAGAATAACTTAAGGC---AAGGAGC 381
Homo   CACTTTATTCTAATTCACCCAAAGG--TACTGTAAGTGCTGATGGCTCTGGGTCTGTCTTTCTGTCAAGTTGCCAAGTCACATAAGTAATGAATCCGCAGCTGGAGGAGC 835

Mus    TTGTGCTTGAGAATA---------------AGGAGCCTTGTCTGT-GTTCCTGAACATG------------AGTGAAAAGCTTCAAAGGCCGCATGT------------- 790
Rattus TTGTGCTTGAGAATA---------------AGGAGCCTTGCCTGT-GTTCCTGAACAGG------------AGTGAAAAGCTTCAAGGGCGACATGT------------- 727
Cavia  TTTTGCTTGAGAACAGGTATGTTTTAAATAAGGAGCC-AGCTTGT-ACCCATCATAGCA-AAGTCCTACACACTAGTGAAATCTAAGAGCTAAGCAGGCCTCTGTAAAAG 488
Homo   TGCTGCTGGAGGACAGGCATGTTTTCAGACAGGAGCATGGCCTTTAGCTCATGACCATAAGAGGGCGCTACAGCAAAGAGTACTAAATACCAATCAA------------- 932

Mus    ----------GATAGAAGGACAGCAGCTCACGTGTTCTCTG-----ATGCAGAGCCGTATCCCTCCCCAGCTGCGCCTCAGGCATATGCGCTGTTCCAAAGAGGCTGAG 885
Rattus ----------TGCGGAAGGACGGCAGCTCACGTGTCCTCCA-----ACACAGCGCTGCGTCCCTCCCCAGGTGCTCCTTAGCCGTATGCACTGTTCCAAGGGGGTTGGG 822
Cavia  CAAAAGTGCCTTAATACAAGAAGGAAGCCC----------------AAGTAGAAACACAACCCTCGAAAGGCTGCCGCTGTAATAATGTGCTGTGTCCTGGTGAACTTC 581
Homo   -AGTGAGCTCAGAGTACAAGAGGGTAACTTGCTTTTCTCCGAAGTGAAAGAGAAGACATAGTCCTCAAACACTT-------GCCCTGTGCCCTGTCCCTGAACTCTCCAT 1034

Mus    TTCAGGG------CTAATATTTGGTATAGGTTCAGTTTAGAGAGGCTTTGAATAAGGGGTAGC--------------------------CTGTTTGTGAAGATCTTTC---- 961
Rattus GCAGGGG------CTGTTCCTCAGCATATGTTCATTTTA-------TGTTGGAGAGGCTTCGT--------------------------TTTCTTGTGAAGTTCTTCT---- 891
Cavia  GGAGGGA------CTTTCTACCACTGGTAACAGAATTTATGAAGGTCTTTTAAGAAGG-------------------GGTAACAGTGTGTTTATGAATACCATTTTTGT 666
Homo   GGAGGAATTTTTTCTGTTGTTTAGTGTTAGCAGAATTTAGGAAGGGCTTGTAATAAGGAAGGTTGAGGGTTGGGTGGGGTCACACTGTGTTGTGTGAGGATCATTTTCAT 1144

Mus    ----------------------AGCAAAGGTGAGCGCAGAGCCTTAATACTTCTTTCTCTGCTGTACCCACT--------------CAG 1014
Rattus ----------------------GGGAAGGGTAAGCACAGAGCCTTGATACTCCTCTCTTTACTCCTCTCTCTCTGCTGTACCCACTCAG 958
Cavia  GTCA----------------------------AATTCTTGAATCTTGACATTTCTTTCTCTACTATTCC------------------CAG 710
Homo   GTTAATGAAATGTTTAGTTCTTTAAGGGGAATGTGGTAGAGCCTTTA----TCTCCCCTGCCCTTTTTTTAAACTGCA------CAG 1223
```

ENSMUSG00000032513 intron 6

Description: Golgi reassembly-stacking protein 1 (Gorasp1)
Intron number: 6
Mouse chromosome: 9
Upstream exon length: 211
Downstream exon length: 151
Mouse intron length: 587
Intron alignment length: 1121
Total murinae branch length: 0.19773
K_score: 0.07363
Scaling factor: 0.72481

ENSMUSG00000032513 exon 6 (ORF 1)

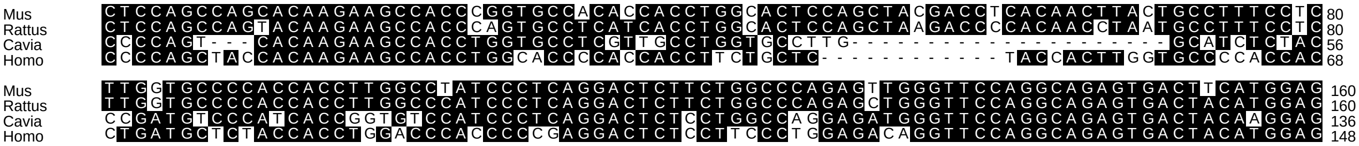

ENSMUSG00000032513 exon 7 (ORF 0)

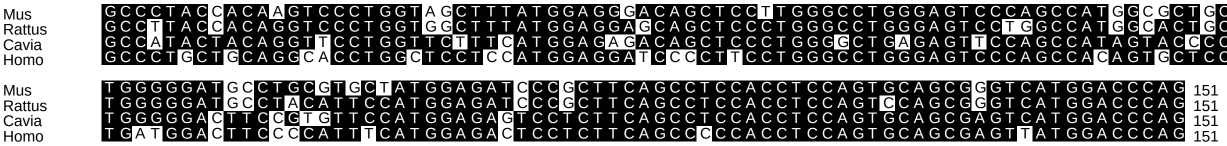

ENSMUSG00000032513_intron_6

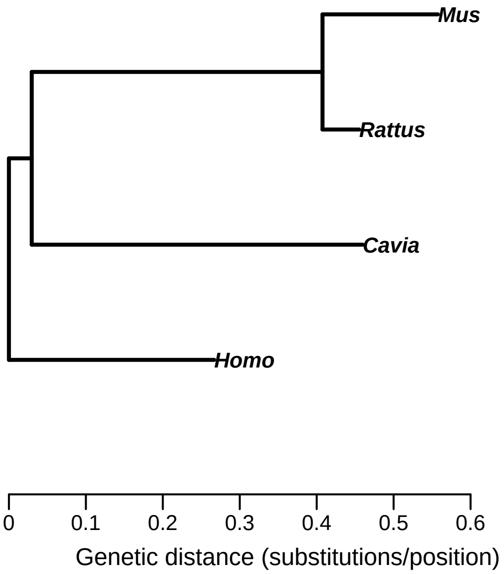

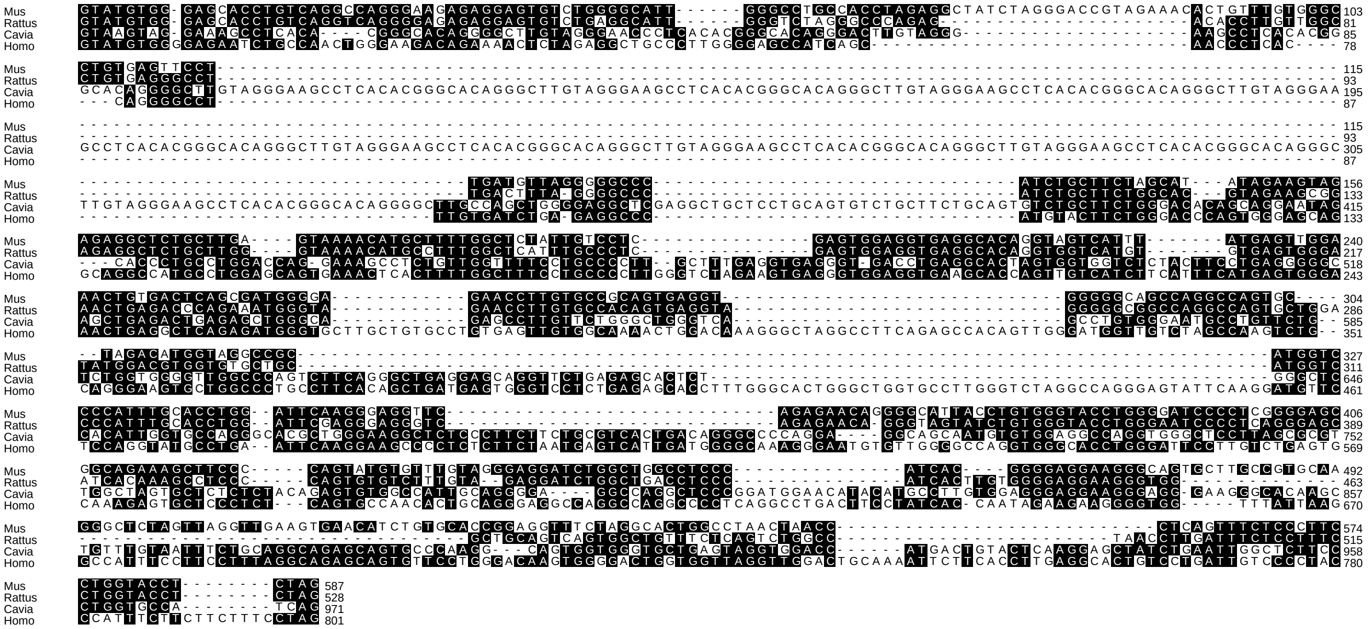

ENSMUSG00000024581 intron 10

Description: Gamma-soluble NSF attachment protein (Napg)
Intron number: 10
Mouse chromosome: 18
Upstream exon length: 80
Downstream exon length: 130
Mouse intron length: 571
Intron alignment length: 801
Total murinae branch length: 0.18878
K_score: 0.08827
Scaling factor: 0.72851

ENSMUSG00000024581 exon 10 (ORF 0)

```
Mus     AAAACAATTGCTCAGGTCTTAGTTCATCTACACAGAAATGACTATGTGGCTGCAGAGAGGTGTGTCAGGGAGAGCTACAG 80
Rattus  AAAACAATCGCTCAGGTCTTAGTTCATCTACACAGAAATGACTATGTGGCTGCAGAGAGGTGTGTCCGGGAGAGCTACAG 80
Cavia   AAAACCATCGCCCAGGTCCTGGTTCACCTGCACAGAAACGACTATGTGGCTGCGGAAAGGTGTGTCAGGGAGAGCTACAG 80
Homo    AAAACAATTGCTCAAGTCTTAGTTCATCTACACAGAAATGACTATGTAGCTGCAGAAAGATGTGTCCGGGAGAGCTATAG 80
```

ENSMUSG00000024581 exon 11 (ORF 1)

```
Mus     CATACCAGGCTTTAACGGCAGTGAGGACTGTGCGGCGCTGGAGCAGCTCCTGGAGGGCTACGACCAGCAGGACCAAGACC 80
Rattus  CATACCAGGGTTTAACGGCAGTGAAGACTGTGCCGCGCTGGAACAGCTCCTGGAGGGCTATGACCAGCAAGACCAAGATC 80
Cavia   CATCCCAGGCTTCAATGGTAGTGAGGACTGTGCGGCGCTAGAACAGCTCCTCGAGGGCTACGACCAGCAGGACCAGGACC 80
Homo    CATCCCTGGGTTCAATGGCAGTGAAGACTGTGCTGCCCTGGAACAGCTTCTTGAAGGTTATGACCAGCAAGACCAAGATC 80

Mus     AAGTGTCCGAGGTCTGCAACTCGCCCCTTTTCAAATACATGGACAATGAT 130
Rattus  AAGTGTCCGAGGTCTGCAACTCGCCCCTGTTCAAATACATGGACAATGAC 130
Cavia   AGGTGTCAGAGGTCTGCAACTCGCCACTCTTCAAGTACATGGACAACGAC 130
Homo    AGGTGTCAGATGTCTGCAACTCACCGCTTTTCAAGTACATGGACAATGAT 130
```

ENSMUSG00000024581_intron_10

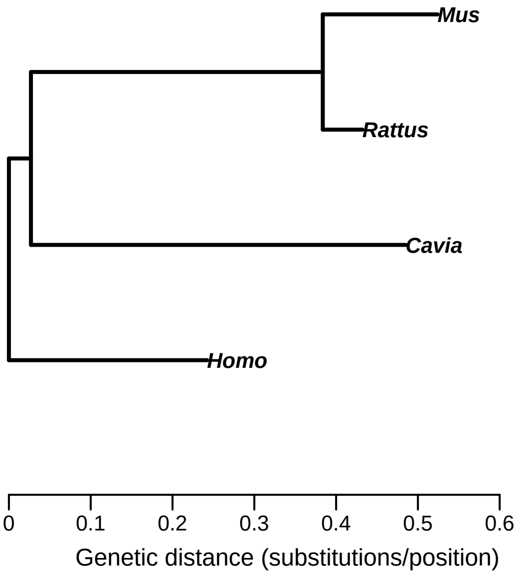

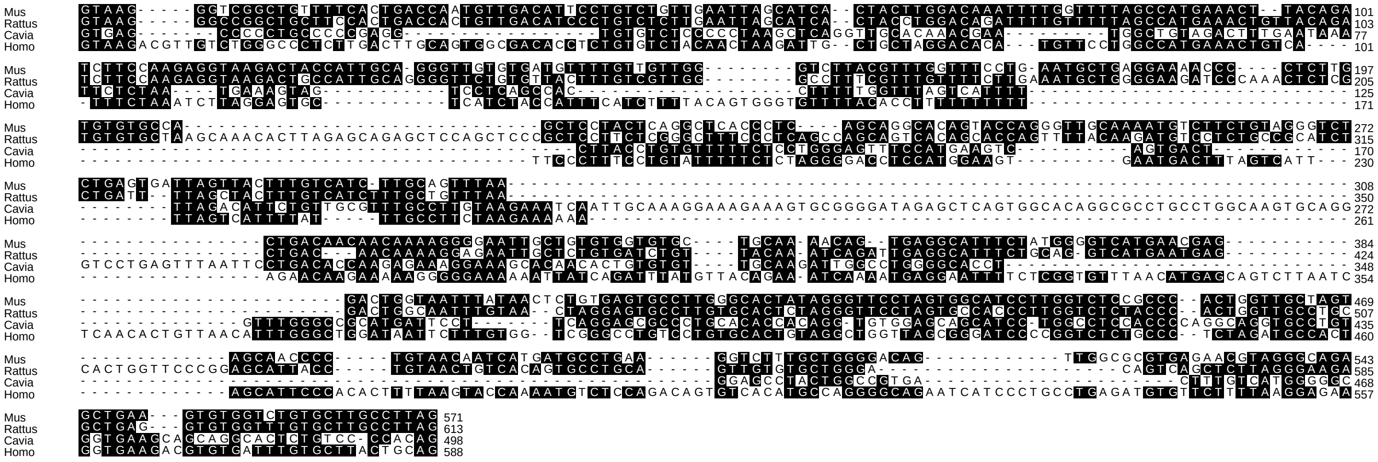
Mus
Rattus
Cavia
Homo

ENSMUSG00000034918 intron 13

Description: protocadherin 24 (Cdhr2)
Intron number: 13
Mouse chromosome: 13
Upstream exon length: 162
Downstream exon length: 62
Mouse intron length: 257
Intron alignment length: 292
Total murinae branch length: 0.18375
K_score: 0.04519
Scaling factor: 0.73134

ENSMUSG00000034918 exon 13 (ORF 0)

Mus	TGTGGCCACTGACTCAGTCAGCAACAACTACTCTGTTGCCACGGTGACCATCCACCTTAGAAACATCAATGACCACAGGC	80
Rattus	TGTGGCCACTGACTCAGTCAGCGGCAACTACTCAGTTGCCACGGTGACCATCCACCTTCGAAACATCAATGACCACCGGC	80
Cavia	TGTGGCCACTGACTCAGTCAGCAAGAAATCCTCCATTGCCACGGTGACCATCCATCTCACAGACATCAATGACCACAGGC	80
Homo	TGTGGCCACAGACTCCGTCAGCCAGAACTTCTCCGTCGCCATGGTGACCATCCACCTTAGAGACATTAATGACCACAGGC	80
Mus	CTGTGTTCTCTCAGAGCCTGTATGAACTCACTGTGCCAGAGCACTGTCCAACAGGTTATCTGGTCACTGACAAAATCCAG	160
Rattus	CCGTGTTCTCTAAGAGTTTGTATGAACTCACTGTGCCAGAGAACAGTCCAACAGGTTTTCTGGTCACCGACAGCATCGAG	160
Cavia	CCACCTTCCCTGAGAGGTCATACAACCTCAGCCTGCTGGAGGACAGTGCCACAGGCACTGTGGTCACCAGCAGCATTCAT	160
Homo	CCACGTTTCCCCAGAGCTTGTACGTCCTCACGGTGCCAGAGCACAGCGCCACCGGCTCTGTGGTCACCGACAGCATCCAC	160

ENSMUSG00000034918 exon 14 (ORF 0)

Mus	GCTACAGACCTGGATGGGGATGAATGGGGTCCTATCACCTACAGTCTGCTTCCAGGAAATGG	62
Rattus	GCTACAGACCTAGATGGGGACGAATGGGGTCCTATCACCTACAGTCTGCTTCCAGGAGCTGG	62
Cavia	GCTGAAGACCCAGACACGGGTGAATGGGGCCGCATCACCTACAGCCTGCTCTTAGGGAATGG	62
Homo	GCCACGGACCCAGACACGGGCGCGTGGGGCCAAATTACCTACAGCCTGCTCCCAGGAAATGG	62

ENSMUSG00000034918_intron_13

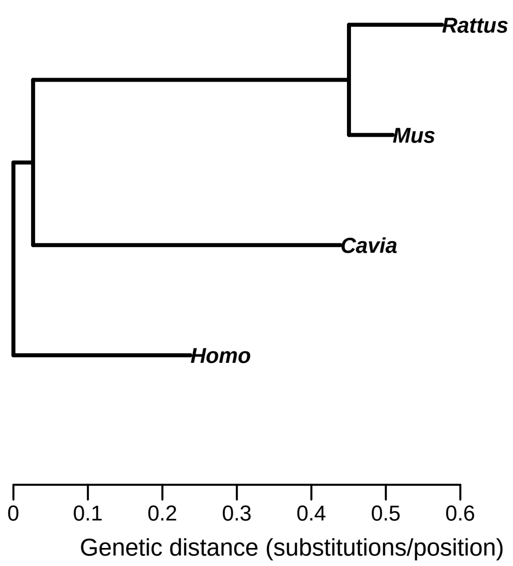

```
Mus     GTGAGTGATGTTGGCTGGATCATGCTGGG-GGAGAGCTGCTGAGCAGGCC----TGAGCTCAGACTGTCCCAGACTTTGTTTCATTCAGTGACACTGGGTCTCTGCGAGT 105
Rattus  GTGAGTGATGTTGGCTGGGTCCCTCTGGGAGAAAAGCTGCTGGGCAGGGCGGATCGATCTCAGACCGTCCCAGACCTTGTTTCATTCAGTATCACTGGGTCTCTGCGAGT 110
Cavia   GTGAGTGACAC-------------------GAGATGTGGCTGGCCAGGCT-------------------------------------------GGGAGGCTGCTGTGGAG 48
Homo    GTGAGTGATGTGGACACAGTGGGGCTGT--GGGGTCCCAGCAAGCGGGC-----------------------------------------------GTGCATGTGTGAGT 61

Mus     GTGCGTA-CGATGTATCTGGGT------GTGTGGAGTGTCTAGGTGTGAATGCAGACATGGTGAG-------CTTTGGAGCCAGGATGCACAGCAACTGTGTCAGATGGG 201
Rattus  GTGCATAGAAATGTATCTGGATGTG---GTGTGGGGTGTCTGGGTGTGAATGTAGATG----GGG-------CTTCAGAGTCGGGGTGCACAGCAGCTGT---------- 196
Cavia   AAGGGAA---------------------GATCTGGGAACTGTGGTGTGGATCCAGATGTG--AGAAGTCCTCCCTTTGTGCCAGGGGGCAGGACAGCCCTGTC------- 128
Homo    GTGAGAGTGTGTGTGTGTGTATGTGTGAGATGGGGGTGGCCATGAGTGAATCTGAGTGTGCTGGGGATTCTCCCTTTGTGTGAGGAAGCACGACAGCTCTGTC------- 164

Mus     TCTCTTGGTGACACAGATGG----------CCCTGTTTCCA----GTGTTCTGCCA--TCTTTGCCTTACAG 257
Rattus  -------GTGACACAGATGGC---------CCCCATATCCA----GTGTTCCGCCA--CCTTTCCCTCACAG 246
Cavia   TCCATGAACTGCACGGTAGGCAGCAGGTGCCATTGTGTCTAAACTGCCTCCTCTCAT-CCCCCGCTTCACAG 199
Homo    CCCACGAGCTGCATGGGTGGCGGTGCGTG-CATCGCCTCCACACAGCACCCTGCCATGTCTCTGCCTCACAG 235
```

ENSMUSG00000022096 intron 11

Description: Protein hairless (Hr)
Intron number: 11
Mouse chromosome: 14
Upstream exon length: 166
Downstream exon length: 70
Mouse intron length: 203
Intron alignment length: 212
Total murinae branch length: 0.17503
K_score: 0.07502
Scaling factor: 0.73222

ENSMUSG00000022096 exon 11 (ORF 0)

```
Mus     TTAGTGTCAGGCATCCAGAAGACATTGAGACTTAGCCTGTGGGGAATGGAAGCCCTTGGGACACTTGGTGGCCAGGTGCA 80
Rattus  TTAGTATCAGGCATCCAGAAGACATTGAGACTTAGCCTGTGGGGAATGGAAGCCCTTGGGACACTTGGTGGCCAGGTGCA 80
Cavia   TTGGTGTCAGGGATCCAGAGGACGCTGCAAGGTGGCCTGTGGGGGATGGAAGCTCTTGGGGCACTGGGAGGCCAGGTGCG 80
Homo    TTGGTGTCAGGGATCCAAAGGACATTGCAGGGCAACCTGTGGGGGACAGAAGCTCTTGGGGCACTTGGAGGCCAGGTGCA 80

Mus     GACACTGACTGCCCTTGGGCCTCCCCAGCCCACAAACCTGGACAGCACAGCATTCTGGGAGGGATTCTCTCATCCTGAGA 160
Rattus  GACACTGACTGCCCTTGGGCCGCCCCAACCCACCAGCCTGGACAGCACAGCATTCTGGAAGGGATTCTCTCATCCTGAGG 160
Cavia   GGCACTGACCGCCCTGGGGCCCCCACAGCCCACAAGCCTGGACACTGCAGTCTTTTGGGAGGGATTCTCCCGGCCTGAAA 160
Homo    GGCGCTGAGCCCCCTCGGACCTCCCCAGCCCAGCAGCCTGGGCAGCACAACATTCTGGGAGGGCTTCTCCTGGCCTGAGC 160
```

ENSMUSG00000022096 exon 12 (ORF 2)

```
Mus     CACGTCCAAAGTTAGATGAGGGCTCTGTCCTCCTGCTACACCGAACCCTGGGGGATAAGGACGCTAGCAG 70
Rattus  CACGTCCAAAGTTAGATGAGGGCTCTGTCCTCCTGCTACACCGACCCCTGGGGGATAAGGACGAGAGCAG 70
Cavia   GTCGCCCAAAGTCAGATGAGGGTTCCGTCCTTCTGCTGCACCGAACTCTGGGAGACGAGGATGCCAGCAG 70
Homo    TTCGCCCAAAGTCAGACGAGGGCTCTGTCCTCCTGCTGCACCGAGCTTTGGGGGATGAGGACACCAGCAG 70
```

ENSMUSG00000022096_intron_11

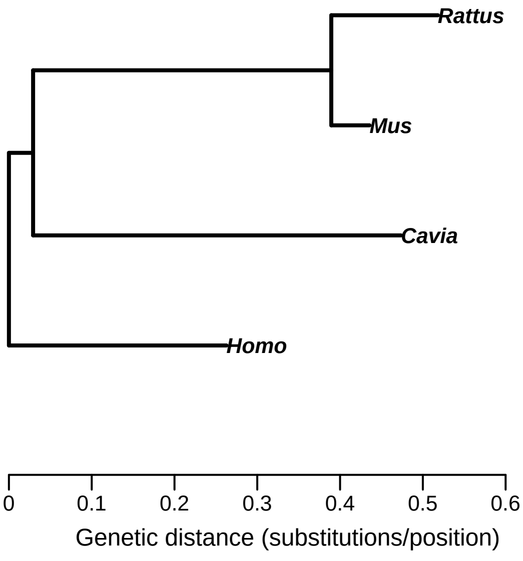

```
Mus     GTAAGTATTCCTGACATAGGATAGAGGGAGCTG---GGAGCCTAACCCCGAGGCTTCATGTATCAAGGGGCCCCTAGAATAGCCCTGGAGGC-----AGATGGGTTCAGG 102
Rattus  GTAAGTATTCCTGGCATAGGGTAGAGGGAGCTAACTGGGGAGCTATCCTGAGATTTCATGTATCTAGGGGCCCCTAGAATAGCTCTGGAGGC-----AGATGGGTTCAAG 105
Cavia   GTGAGTATCCCTGATGTGGACTACAGGGAGCTG---GGTGCATGAGTCTGAGGGTTGGGGTGACAAGGGAACCCTGGAGTGATCTAAGTGTCACTAGAAGCAGGACGAGA 107
Homo    GTAAGTGTCCCCAACACAGGGGAGAGGGAGCTG---GGAGCCCAAGCCCTAGGGATCGGGTGGCAGAGGAGTCCCAGGGTGACCCCGAGG------------GGCTGGAA 95

Mus     GGAGAAACTAAACTTAGGGATGGGGTGCCACAGGGACAAGAAAGAGGCTCTGGGCCAAGAGGCTCTACCTTGGGTCAATGA-CATCTGTCTTTTCCCCTTAG 203
Rattus  GTAGAAACTGAACACGGGTAT-GGGTGCCACAGGGACAAGAAAGAGTCTCTGGGCCAAGAGGCTTCATCATGGGTGAATGG-CATCTGTCTTTTCTCT-TAG 204
Cavia   GCAGGTACT-------------------------------------TCTGCCAGCCCTGGAGCTCCGCCAGGGGCCAAGGG-CATTCTGTGCCTTCCCCCAG 171
Homo    GCAGGTGTT--------------GCTGCCGCCGGGGCAGGGG----CTTCAGGGCTGAGGA-----------GGCCAATGGCCGTTCTGTCTCCTCCTGTAG 168
```

ENSMUSG00000040688 intron 1

Description: Transducin beta-like protein 3 (Tbl3)
Intron number: 1
Mouse chromosome: 17
Upstream exon length: 41
Downstream exon length: 52
Mouse intron length: 1357
Intron alignment length: 2152
Total murinae branch length: 0.16635
K_score: 0.08567
Scaling factor: 0.73271

ENSMUSG00000040688 exon 1 (ORF 0)

```
Mus     ---------ATGGCCGAGACTGCGGCGGGATTGTGCCGCTTCAAAGCCAA 41
Rattus  ---------ATGGCCGAGACTGCGGCGGGATTGTGCCGCTTCAAAGCAAA 41
Cavia   GGCAACAATATGGCTGAAACAGTTGCCGGAGTGGGCCGCTTTAAAGCTAA 50
Homo    ---------ATGGCAGAGACCGCGGCCGGAGTGGGCCGCTTCAAGACCAA 41
```

ENSMUSG00000040688 exon 2 (ORF 1)

```
Mus     CTATGCTGTTGAGCGAAAGATTGAACCTTTCTACAAGGGTGGGAAAGCACAG 52
Rattus  CTATGCTGTTGAGCGAAAGATCGAACCTTTCTACAAGGGTGGGAAAGCACAG 52
Cavia   CTATGCTGTGGAGCGCAAGATTGAGCCTTTCTACAAGGGCGGGAAAGTACAG 52
Homo    CTATGCTGTGGAGCGCAAAATTGAGCCTTTCTACAAGGGCGGAAAAGCACAG 52
```

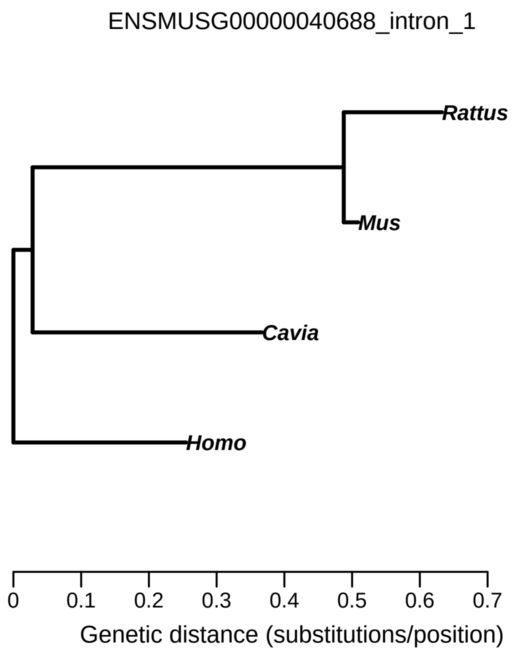

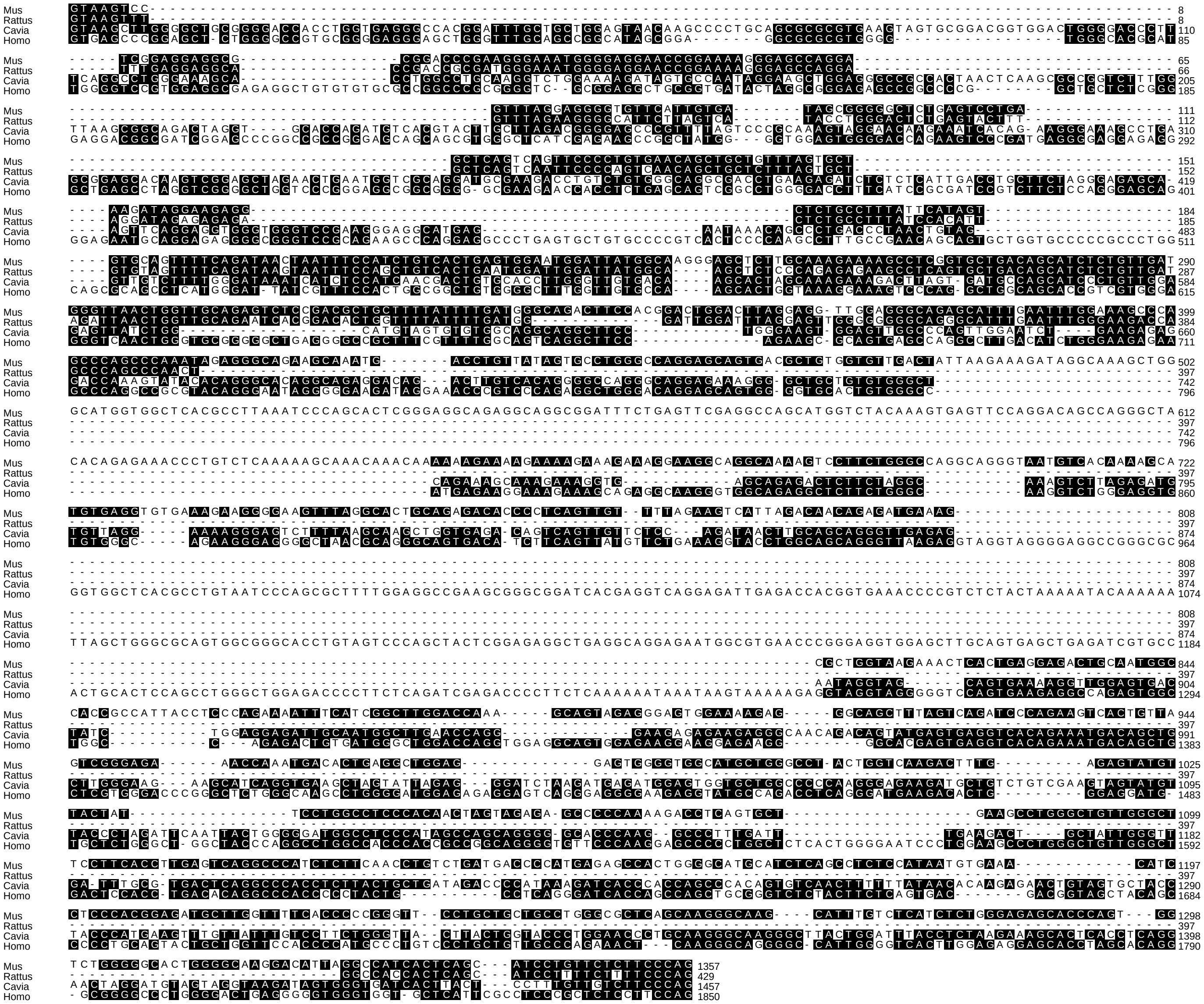

ENSMUSG00000014158 intron 7

Description: Transient receptor potential cation channel subfamily V memb (Trpv4)
Intron number: 7
Mouse chromosome: 5
Upstream exon length: 159
Downstream exon length: 93
Mouse intron length: 229
Intron alignment length: 318
Total murinae branch length: 0.28154
K_score: 0.07043
Scaling factor: 0.73431

ENSMUSG00000014158 exon 7 (ORF 0)

```
Mus     AACCGCCATGAGATGCTGGCTGTAGAGCCCATTAACGAACTGTTGAGAGACAAGTGGCGTAAGTTTGGGGCTGTGTCCTT 80
Rattus  AACCGCCATGAGATGCTGGCTGTGGAGCCCATTAACGAACTGCTGAGGGACAAGTGGCGTAAGTTCGGGGCCGTGTCCTT 80
Cavia   AACCGCCATGAGATGCTGGCAGTGGAGCCCATCAACGAGCTGCTGCGGGACAAGTGGCGGAAGTTTGGGGCTGTCTCCTT 80
Homo    AACCGCCACGAGATGCTGGCTGTGGAGCCCATCAATGAACTGCTGCGGGACAAGTGGCGCAAGTTCGGGGCCGTCTCCTT 80

Mus     CTACATCAACGTGGTCTCCTATCTGTGTGCCATGGTCATCTTCACCCTCACCGCCTACTATCAGCCACTGGAGGGCACG 159
Rattus  CTACATCAACGTTGTCTCCTATCTGTGTGCCATGGTCATCTTCACCCTCACAGCCTACTATCAGCCACTGGAGGGCACG 159
Cavia   CTACATCAACGTGGTCTCCTATCTGTGTGCCATGATCATCTTCACCCTCACTGCCTACTACCAGCCACTGGAGGGCACT 159
Homo    CTACATCAACGTGGTCTCCTACCTGTGTGCCATGGTCATCTTCACTCTCACCGCCTACTACCAGCCGCTGGAGGGCACA 159
```

ENSMUSG00000014158 exon 8 (ORF 0)

```
Mus     CCACCCTACCCTTACCGGACCACAGTGGACTACCTGAGGCTGGCTGGCGAGGTCATCACGCTCTTCACAGGAGTCCTGTT 80
Rattus  CCACCCTACCCTTACCGTACCACGGTGGACTACCTGAGGCTGGCTGGTGAGGTCATCACGCTCCTCACAGGAGTCCTGTT 80
Cavia   CCACCCTACCCTTACCGCACCACAGTGGACTACCTGAGGCTGGCCGGCGAGGTCATCACGCTCTTCACGGGGGTGCTGTT 80
Homo    CCGCCGTACCCTTACCGCACCACGGTGGACTACCTGCGGCTGGCTGGCGAGGTCATTACGCTCTTCACTGGGGTCCTGTT 80

Mus     CTTCTTTACCAGT 93
Rattus  CTTCTTTACCAGT 93
Cavia   CTTCTTCACCAAT 93
Homo    CTTCTTCACCAAC 93
```

ENSMUSG00000014158_intron_7

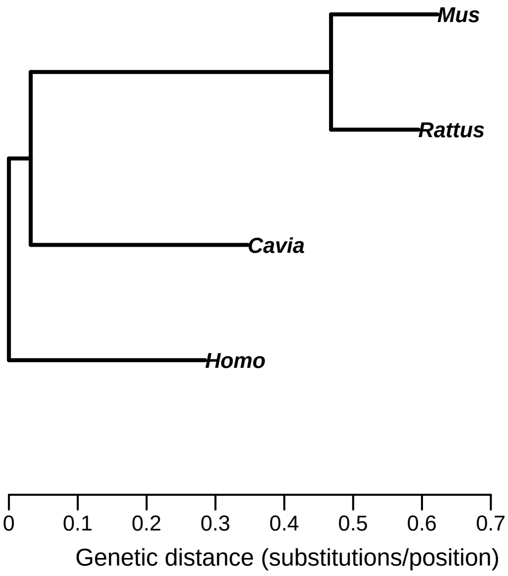

```
Mus     GTGAGTGCTCACTGGGGAGCC-GGCACAAGGACAG--------------------------------------------------CCTCTGCAGCGGGACA--GAAGACA 57
Rattus  GTGAGTGCTCGCTGGGGAGATGGGCATGGGAGCAG--------------------------------------------------CCTCTGTGGCAGGGGAGTGAAGACA 60
Cavia   GTGAGTGCTTGGAGCTGGGCAGAGC---GAGGCTAGTGCTGGGCTGGGGCCCTCAGGCCTGGGAC---AGCTGGTTCTCCTGGGTCCCCAAGGGAGGGA-ATAGACAGCC 103
Homo    GTGAGTGCCCGGGGACCGGGCAGGGGCTGGGGCAGGCACTGGGCTGAGCCATGCAGGACTGGGGCACAACCTCATCCTTCTGGGTCCCCTGTAGGGGGACCCGGAGAAGG 110

Mus     GCTTGGGGGTGG---GGAGGAAGATGCCCAGCACCCA--GATGGCTCA-------GCCTGGAGAGACTTTGGGGGAG----ACAGGAGAGGGCGCTGCTGAGGACGCAGA 151
Rattus  GCTTGGGGTGGG---GAGGAAGATACCCCAGCACCCT--GATGGCTG--------GGAGGAGGAGACTCTGGGGGAG----ACAGGAGAGGAAGTTGCTGAGGACACAGA 153
Cavia   CCTACGGACAGGT--GGAGGGGGTCTGCCGACATCCA-GGGTGGCCCT---------GGGAGGAAACCCAGGCGGAAGCTAAC-------AGGGCTTCAAAGGACCCAGA 194
Homo    TTTAGGAACAGGTTGGGGAGGCGCCCTCCAGCATCCACGGGTGGCCCTGAGCTGGGAGGAGGAAGACTCAGGAGGAAGAGAGTGAAGGAGGAGGCTCCATGGGATGCCGA 220

Mus     TGTTTGGGGCAGCAGGGAGC-------------------TGGTGTTGGGGGCAGGGGAGGACCTGT-TGCATCTGCTCTTCCCGCCCTGCCCCCTCAG 229
Rattus  TGTTTGGGGCAGCGAGGAAC-------------------TGGTGTTGAGGGCAGGTGAAGACCTGC-TGCATCTGCTCCCCCC----------CTCAG 221
Cavia   TGTTCGTGGCGCTGGGGAACATCTGGATTGAGGG-CAGAGGCTGGAGGGG-CAGATGAGGATCT---TGCCCCTCCTCCG---------------CAG 272
Homo    TGTTTCGGGCCTGGGGGAACATCTGGATTGGGGGCCAGATGTTGCAGGGGCTGGGTGACGACCTGTGTGCCCTTGCTGCTCCC------------CAG 306
```

ENSMUSG00000038801 intron 1

Description: Secretoglobin family 1C member 1 Precursor (Scgb1c1)
Intron number: 1
Mouse chromosome: 7
Upstream exon length: 55
Downstream exon length: 200
Mouse intron length: 416
Intron alignment length: 574
Total murinae branch length: 0.10638
K_score: 0.08509
Scaling factor: 0.73629

ENSMUSG00000038801 exon 1 (ORF 0)

```
Mus     ATGAAAGGGAGCAGCGCTCTTCTGCTGGTGGCCCTAAGCCTGCTCTGCGTCTGTG 55
Rattus  ATGAAAGGGAGCAGCGCTCTTCTGCTGGTGGCTCTAACCGTGCTCTGCATCTGTG 55
Cavia   ATGAAGGGAAGCAGCACCCTCCTGCTCGTGGTCCTCACTGTGCTCTGTACTTGTG 55
Homo    ATGAAGGGGAGCCGTGCCCTCCTGCTGGTGGCCCTCACCCTGTTCTGCATCTGCC 55
```

ENSMUSG00000038801 exon 2 (ORF 2)

```
Mus     GGCTGACTAGAGCAGAGGATGACAATGAGTTTTTCATGGAATTCCTGCAAACACTCCTGGTGGGGACCCCGGAAGAACTC 80
Rattus  GGCTGACTAGAGCAGAGGATGACAATGAGTTTTTCATGGAATTCCTGCAAACACTCCTGGTGGGGACCCCAGAAGAGCTC 80
Cavia   GAAGGGCCAGAGGGGAGGACAACAGTGAATTCTTCATGGAGTTCCTGCAAGTGCTGCTGGTTGGAAGCCCAGAGGAGCTC 80
Homo    GGATGGCCACAGGGGAGGACAACGATGAGTTTTTCATGGACTTCCTGCAAACACTACTGGTGGGGACCCCAGAGGAGCTC 80

Mus     TACGAAGGGCCCCTGGGCAAGTACAATGTCAATGACATGGCCAAGTCAGCGCTGAGCGAGCTCAAGTCCTGCATCGATGA 160
Rattus  TATGAAGGGCCCCTGGGCAAGTACAATGTCAATGACATGGCCAAGGCAGCGCTGACAGAGCTCAAGTCCTGCATTGATGA 160
Cavia   TATGAGGGGCCTCTGGGCAAGTACAATGTCAACCAGGATGCCAAGGAGGCCCTGACAGAGCTCAAATCCTGCATTGATGG 160
Homo    TATGAGGGGACCTTGGGCAAGTACAATGTCAACGAAGATGCCAAGGCAGCAATGACTGAACTCAAGTCCTGTATAGATGG 160
```

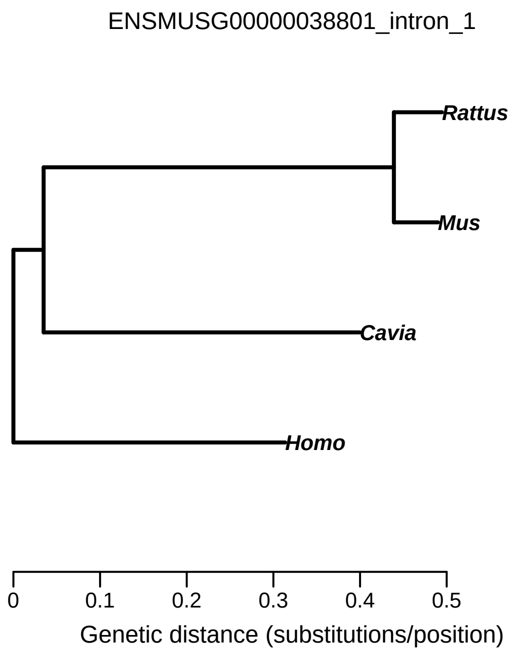

```
Mus     GTAAGTCTGGGCCT----TGTTTCTAGCACTTTCGG-----------TAGGTAAGGGGCCCCAAGAGGCTGAAATCCTGGGATCAGGT------------GGGCAATGCCTA 85
Rattus  GTAAGTCTGGGCCT----TATTTCTAGCACTTT-GG-----------TAGGTAAGGGGCACCAAGAGGCTGGAATCCTGGGACCAGGT------------GGACGGTGCCTA 84
Cavia   GTAAGTCTGGGTCA----GGTTTCCAGTGTATTCAG---------------------------------GGACAGGGTCCTGGGATATAGT------------GGATACCACCTG 66
Homo    GTGAGTCTGTGCCACTGGGGTTTCCAGAACATTCAGGGGTGGGGGGAGTGGGGGGTACCTGAGAAGGTAAAATCCTGGCATCGGGCCTCTGCAGTGAACACTCCACCTG 110

Mus     CCAGTGTCACAGACCCCACCCCTAAGTGCAGGAAATCTGTTGACACCAGTTCTCTCCCAACCCACCCTGACCTAACACTAACTAACCAC-------------CGAGGCC 182
Rattus  CCTGTGTCACTGACCCCACACCCAAAGCGCTGGGAATCTGTTGACACCAGTCCTATCCCAACCCACCCTGACCTAAC----ACCAACCAC-------------CAAGGCC 177
Cavia   CCTCCCTCACAAATCACTCACA-GAAGAGCACAGGCAATGCTAACCCTAATCCCAAACCAGGCCCACTCCAC------------------------------------TC 139
Homo    CTTCTGTCACAGA-----------AGTGGCAAGGACCCTGCAGAACCCGCGCCCACCCTACTCCACCCCAACCAAGG----ACAAAGCCCAGCTGACCCAACACCCAGCCC 206

Mus     AATGCCGCCCACCCTGGAGCAATCCGGTTTGACCTGACCCTCTGGATCC--------------------------AGTTAGCCCAG--GGGAAGAACTCCCCCTTCTCT 263
Rattus  AATGCTGTCCACCCTGGCTCAATCCAATCTGACCTGGCCCTCTGGATCC--------------------------AGTTAACCCAGGCGGGGGGAAATCCCCCTTTTTCT 260
Cavia   AGTGTCACCTACTCCAGCCCAATATAGCATGACCCCA-ACTCTGAACCTCACAGCAGTTCAGCTTCACAACAGGGAAAGAAATCAACTCATATCAAAGCTCAACTTTCCT 248
Homo    AGTGTCTCCTACCCCAGCCCAGTCCAGCCTGACCTGACCCTTCAAGCCTCTGGTCATAATAGCCTCTCATCAGGGGAAAATTTCCCCCACAC-CAGGAGCTTAACTTTCCT 315

Mus     TCCCTGGTGAGGTCAGGAAGT-CAGGGGGGAAACCACAGCAGCTCCCACTAGTGGTGATGGGGAAGGGATCTGC---AGGTTGACCTGGGA------------------ 350
Rattus  TCCCTGGTGAGGTCAGGAAGT-TAGAGGGGAAACCACAGCAGCTCCCACTAGTGGTGATGGGGAAGGGATCTGCAGAAGGTCACCCTGGGA------------------ 350
Cavia   TCCCTGGTGAGGCCAGGAT---CAGAGTGGAAATCAGA--TGCTGAGATTAGAAATGTGAGGAGAAAGGTCTATGGTATTTCGGGTT------------------------ 330
Homo    TCCCTGGTGATCCCAGGGAATGCTGAGTGGAAATTGGAGCAGCTAAGATTAAGGGGGATGTGGAGGGGTCTTCGGGCAAGGTGGAGTTGGAGGTGTTGTGGAGGAAGCCC 425

Mus     ---------------GTGCTAGAAAGACAAGGACAGGAGATCAAG---------------------------------------------------------TCCCTCCCTCGG 392
Rattus  ---------------GTG----------AAGGACAGGAGATCAAG---------------------------------------------------------TCCCTCCCTCGC 382
Cavia   ----------ACACTGTTATGGAGAAAAATATGGTGAAGACCACAGAGCTGAGAGGCCCAGGGAGG----------------------------------GCCCTCTCCTAG 398
Homo    TGGGGAGGAGAGGTGGGCATTGAAGGGGAAGGTCTGGAGACCATGGAGGTTGGAGCCCTGGGGCAGGTCCAGGGCTGTGGTTGCACCAGAGTCACCCTGTCCTGTCCTGG 535

Mus     CATTGTATCCCCTTTGCAACACAG 416
Rattus  CATTGTATCCCCTTTGCAATACAG 406
Cavia   CATCATCTCCCC-TTACAACACAG 421
Homo    CATCATCTCGC--TCGTGATGCAG 557
```

ENSMUSG00000026295 intron 5

Description: Secreted phosphoprotein 24 Precursor (Spp2)
Intron number: 5
Mouse chromosome: 1
Upstream exon length: 55
Downstream exon length: 51
Mouse intron length: 1094
Intron alignment length: 1547
Total murinae branch length: 0.21464
K_score: 0.08103
Scaling factor: 0.7365

ENSMUSG00000026295 exon 5 (ORF 0)

```
Mus     ATGATGTTTGGGGACATGGCAAGATCCCACAGACGAAGAAATGATTATCTACTTG 55
Rattus  ATGATTTTTGGGGACATGGCAAGATCCCACCGACGAAGAAATGATTATCTACTTG 55
Cavia   ATGATTTTTGGGGACATGGGAAGATCCTATAACTGGAGAAACGATTATCTACTTG 55
Homo    ATGATTTTTGGGGACATGTTGGGATCTCATAAATGGAGAAACAATTATCTATTTG 55
```

ENSMUSG00000026295 exon 6 (ORF 2)

```
Mus     GTTTTCTTTCTGATGAATCCAGAAGTGAACAATTCCGTGACCGGTCACTTG 51
Rattus  GCTTTCTTTATGATGAACCCAAAGGTGAGCAGTTCTATGACCGGTCGATTG 51
Cavia   GTCTCATTCCTGAGGAACCCAGAAGCCAGCGGTTTTATGATCGGTCACCTG 51
Homo    GTCTCATTTCAGACGAGTCCATAAGTGAACAATTTTATGATCGGTCACTTG 51
```

ENSMUSG00000026295_intron_5

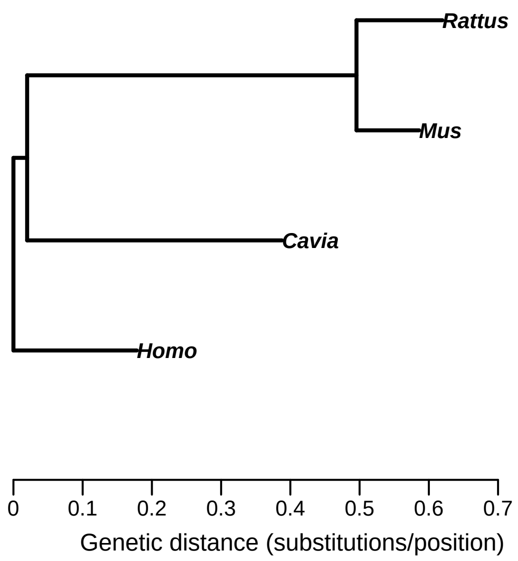

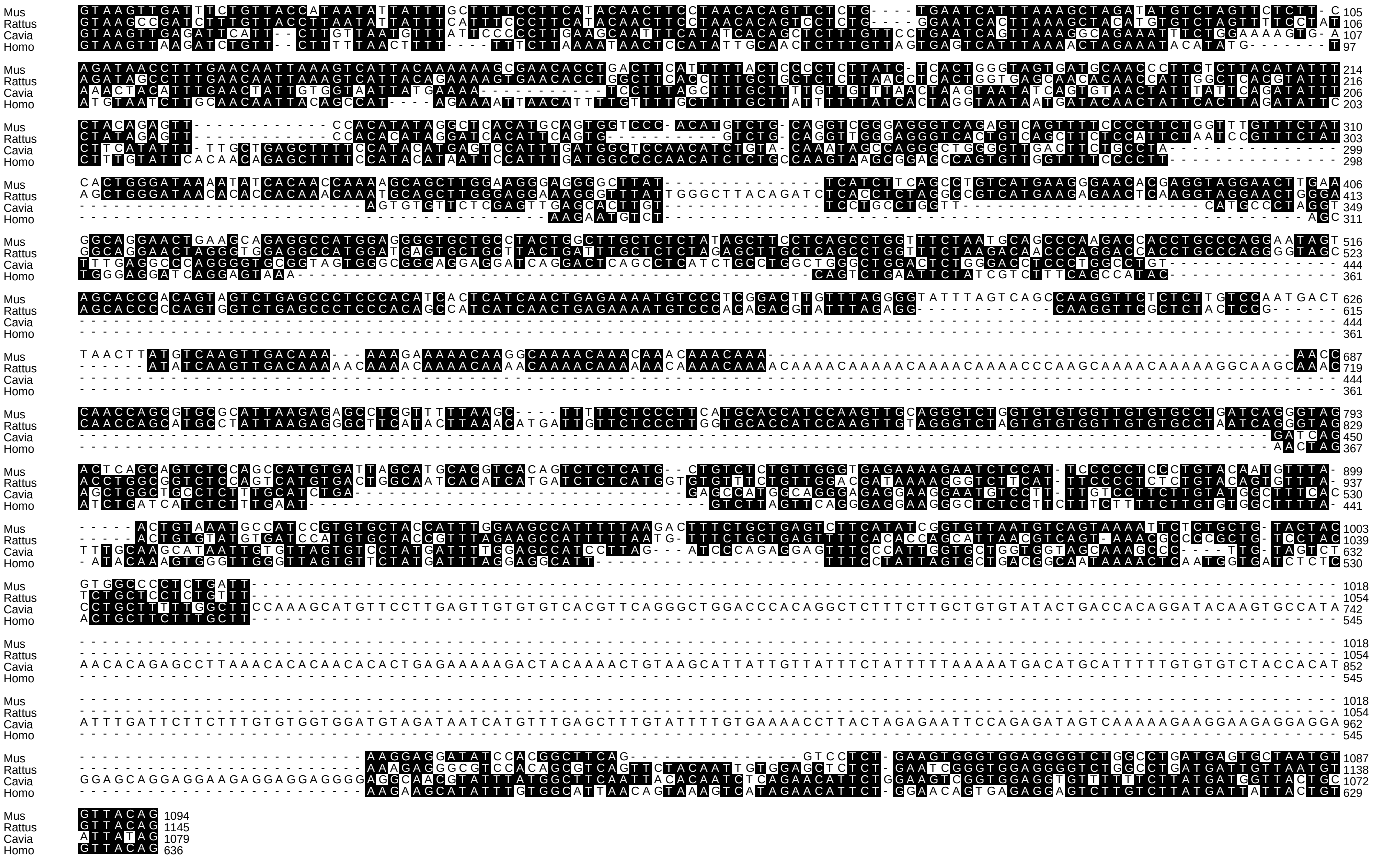
Mus
Rattus
Cavia
Homo

ENSMUSG00000026627 intron 6

Description: Transmembrane protein 206 (Tmem206)
Intron number: 6
Mouse chromosome: 1
Upstream exon length: 145
Downstream exon length: 108
Mouse intron length: 1463
Intron alignment length: 3178
Total murinae branch length: 0.21772
K_score: 0.07495
Scaling factor: 0.73791

ENSMUSG00000026627 exon 6 (ORF 1)

```
Mus     CCCGGACAAGGCGGGCTTCATGCAGGCCTGTGAGAGTGCTTACTCCAGTTGGAAGTTCTCCGGAGGCTTCCGCACCTGGG 80
Rattus  CCCGGACAAGGCGGGCTTCATGCAGGCCTGTGAGAGTGCCTACTCCAGCTGGAAGTTCTCCGGAGGCTTCCGCACCTGGG 80
Cavia   CCCAGACAAGGCGGGCTTCATGCAGGCCTGCGAGAGCGCCTACTCCAGCTGGAAGTTCTCGGGGGGCTTCCGCACCTGGG 80
Homo    CCCAAACAGGGTAGGCTTCATGCAGGCCTGTGAGAGTGCCTATTCCAGCTGGAAGTTCTCTGGGGGCTTCCGCACCTGGG 80

Mus     TCAAGATGTCACTGGTGAAGACCAAAGAAGAGGACGGGCGAGAAGCTGTTGAGTTCCGTCAAGAG 145
Rattus  TCAAGATGTCACTGGTGAAGACCAAAGAAGAGGATGGGCGAGAAGCCGTTGAGTTCCGGCAAGAG 145
Cavia   TCAAGATGTCACTGGTGAAGACCAAGGAGGAGGACGGGCGGGAGGCGGTAGAGTTCAGGCAAGAG 145
Homo    TCAAGATGTCACTGGTAAAGACCAAGGAGGAGGATGGGCGGGAAGCAGTGGAGTTCCGGCAGGAG 145
```

ENSMUSG00000026627 exon 7 (ORF 0)

```
Mus     ACCAGCGTGGTTAACTATATTGACCAGAGACCCGCAGCCGAGAGGAGTGCTCAGTTGTTCTTTGTGGTCTTCGAATGGAA 80
Rattus  ACCAGCGTGGTCAATTATATTGACCAGAGGCCGGCAGCCGAGAAGAGTACTCAGTTGTTCTTTGTGGTCTTTGAATGGAA 80
Cavia   ACCAGCGTGGTTAACTACAATGACCGGAGGCCGGCCGCTGAGAAGAGCGCCCAGCTGTTCTTTGTGGTCTTCGAATGGAA 80
Homo    ACAAGTGTGGTTAACTACATTGACCAGAGGCCAGCTGCCAAAAAAGTGCTCAATTGTTTTTTGTGGTCTTTGAATGGAA 80

Mus     AGATCCCTTCATCCAGAAGGTCCAGGAC 108
Rattus  GGATCCCTTCATCCAGAAAGTCCAGGAT 108
Cavia   AGATCCTTTCATCCAGAAAGTGCAAGAC 108
Homo    AGATCCTTTCATCCAGAAAGTCCAAGAT 108
```

ENSMUSG00000026627_intron_6

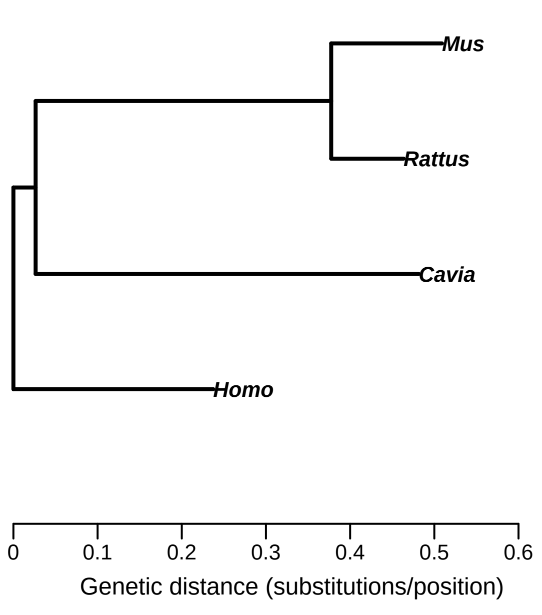

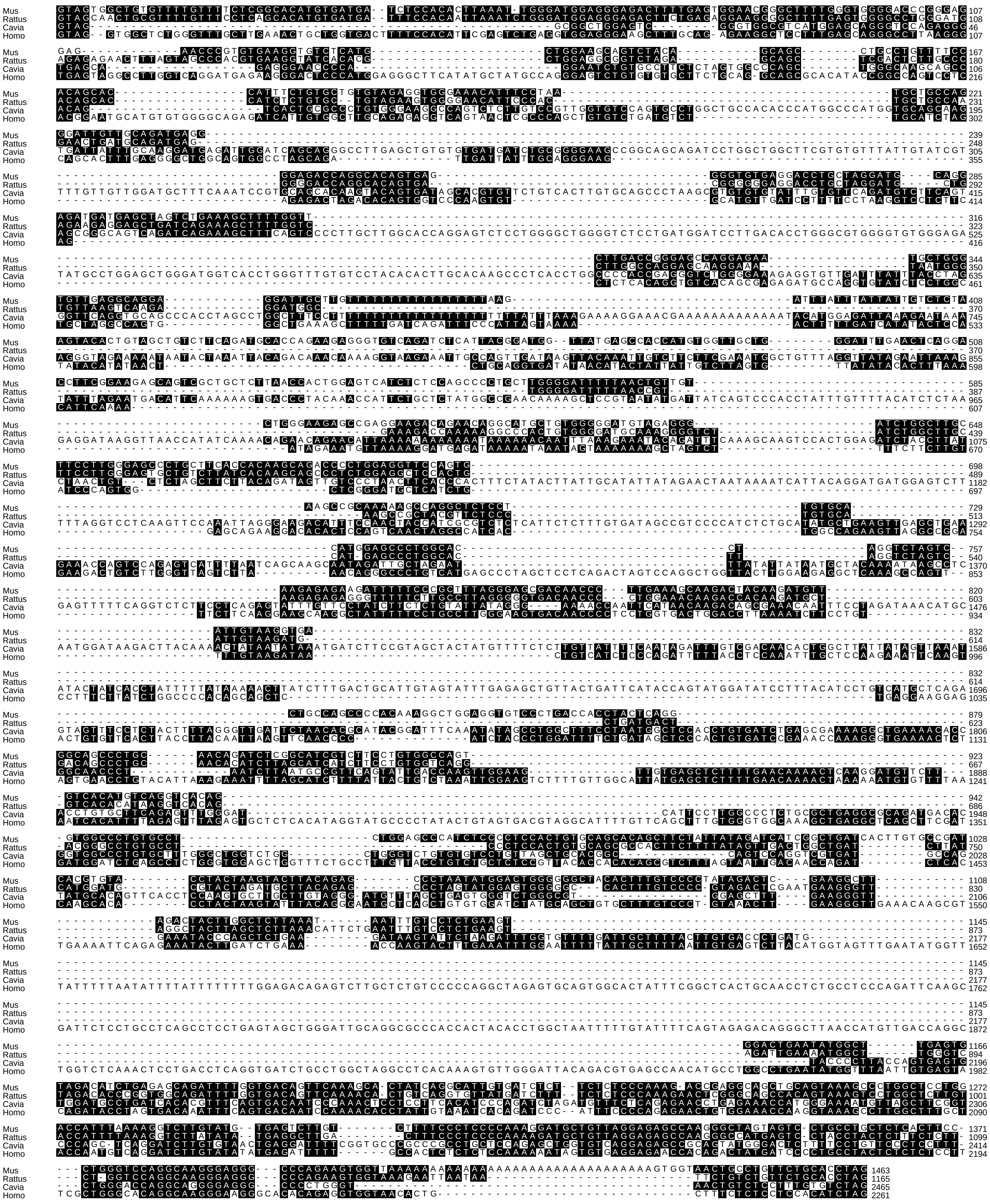

ENSMUSG00000022761 intron 7

Description: Leucine-zipper-like transcriptional regulator 1 (Lztr1)
Intron number: 7
Mouse chromosome: 16
Upstream exon length: 58
Downstream exon length: 140
Mouse intron length: 812
Intron alignment length: 1365
Total murinae branch length: 0.15077
K_score: 0.05286
Scaling factor: 0.73948

ENSMUSG00000022761 exon 7 (ORF 1)

```
Mus     GTTGAACGACATGTGGACAATTGGCCTCCAAGACCGAGAGCTCACATGCTGGGAGGAG 58
Rattus  GTTGAACGACATGTGGACAATTGGCCTCCAAGACCGAGAGCTCACATGCTGGGAGGAG 58
Cavia   GTTGAATGACATGTGGACGATTGGCCTGCAGGACCGGGAGCTCACATGCTGGGAGGAG 58
Homo    GTTGAATGACATGTGGACAATTGGCCTCCAGGACCGAGAGCTCACCTGCTGGGAGGAG 58
```

ENSMUSG00000022761 exon 8 (ORF 0)

```
Mus     GTGGCCCAGAGTGGTGAGATCCCCCCATCCTGTTGCAACTTCCCTGTGGCTGTGTGCCGGGATAAGATGTTCGTGTTCTC 80
Rattus  GTGGCCCAGAGCGGTGAGATACCCCCATCCTGCTGCAACTTCCCTGTGGCTGTGTGCCGGGATAAGATGTTTGTGTTCTC 80
Cavia   GTGGCCCAGAGTGGTGAGATCCCACCATCTTGCTGCAACTTCCCTGTGGCTGTGTGTCGGGACAAGATGTTTGTGTTCTC 80
Homo    GTGGCCCAGAGTGGCGAGATCCCCCCATCTTGCTGCAACTTCCCCGTGGCTGTGTGCCGGGACAAGATGTTTGTATTCTC 80

Mus     AGGACAGAGTGGAGCCAAGATAACTAACAACCTCTTCCAGTTTGAATTCAAAGACAAGAC 140
Rattus  AGGACAGAGTGGAGCCAAGATAACCAACAACCTCTTCCAGTTTGAATTCAAGGACAAGAC 140
Cavia   AGGACAGAGTGGAGCCAAGATAACCAACAACCTCTTCCAGTTTGAGTTCAAGGACAAGAC 140
Homo    TGGGCAAAGCGGAGCCAAAATAACCAACAACCTCTTCCAGTTTGAATTCAAGGACAAGAC 140
```

ENSMUSG00000022761_intron_7

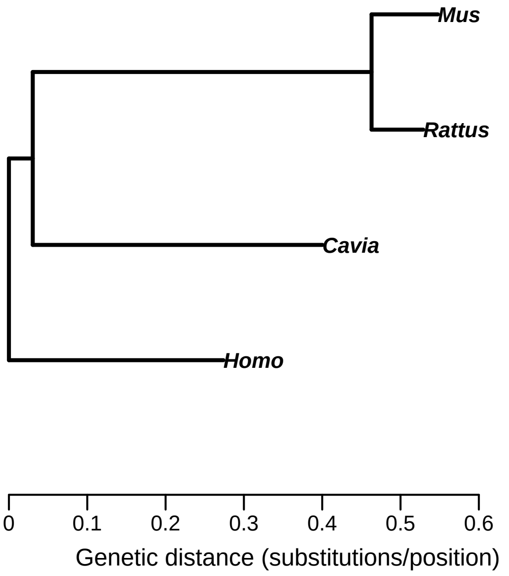

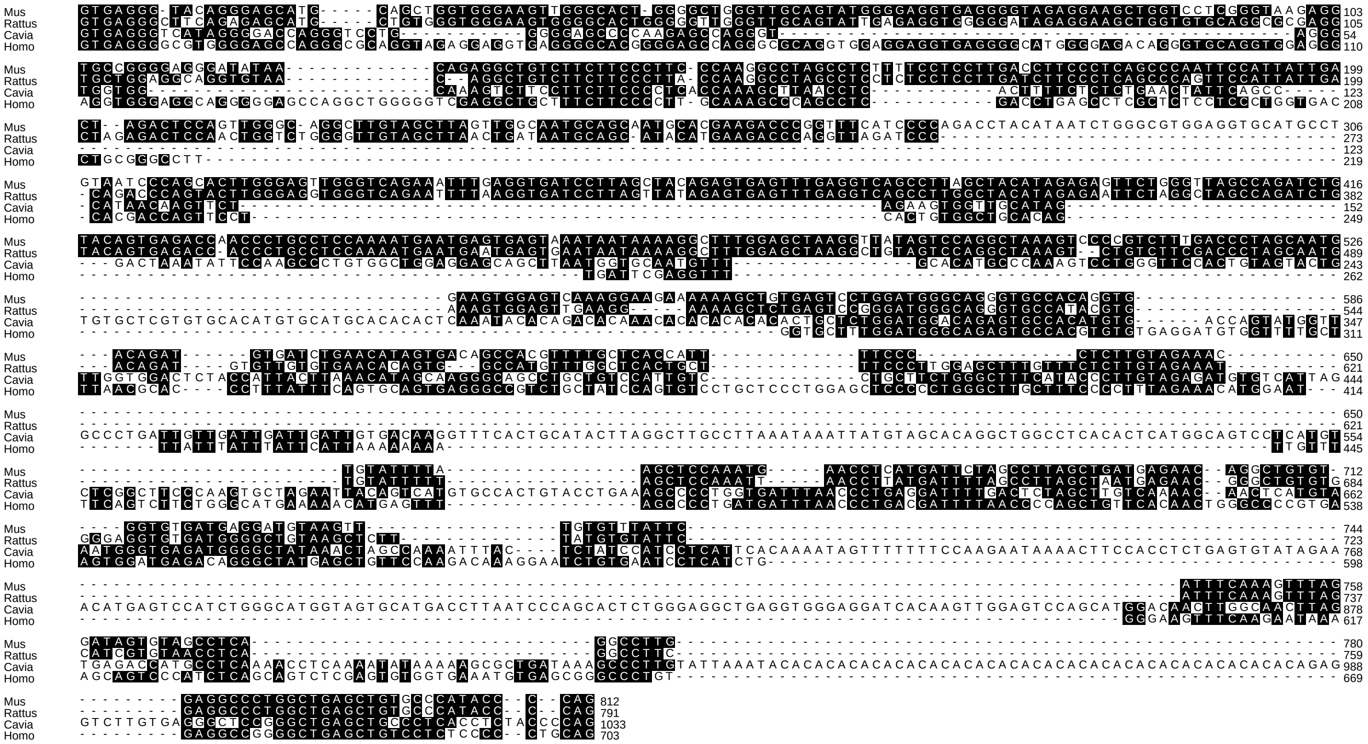

ENSMUSG00000015970 intron 1

Description: Choline dehydrogenase, mitochondrial Precursor (Chdh)
Intron number: 1
Mouse chromosome: 14
Upstream exon length: 709
Downstream exon length: 152
Mouse intron length: 899
Intron alignment length: 1078
Total murinae branch length: 0.19589
K_score: 0.05669
Scaling factor: 0.73965

ENSMUSG00000015970 exon 1 (ORF 0)

```
Mus     GGTGGGGATGGCCCACTGCATGTGTCTCGGGGCAAGACCAACCACCCGCTTCACCAGGCCTTCCTGCAGGCAGCACGTCA 80
Rattus  GGTGGGGATGGCCCACTGCATGTGTCTCGGGGCAAAACCAACCACCCACTCCACCAGGCCTTCCTGCAGGCAGCACGTCA 80
Cavia   GGCGGGACAGGCCCACTGCACGTGTCCCGGGGCCGGAGCGGCAACCCGCTGCACCCGGCCTTCCTGAGCGCCGCGCAGCA 80
Homo    GGCGCCGATGGCCCGCTGCGGGTGTCCCGGGGCAAGACCAACCACCCGCTGCACTGCGCATTCCTGGAGGCCACGCAGCA 80

Mus     GGCTGGCTACCCCTTCACTGAAGACATGAATGGCTTCCAACAGGAGGGCTTCGGCTGGATGGACATGACTGTCCACCAAG 160
Rattus  GGCTGGCTACCCCTTCACTGAAGACATGAATGGCTTCCAACAGGAGGGCTTCGGCTGGATGGACATGACCATCCACCAAG 160
Cavia   GGCGGGCTACCCGCTCACCGAGGGACATGAACGGCTTCCAGCAGGAGGGCTTCGGCTGGATGGACATGACCATCCATGAAG 160
Homo    GGCCGGCTACCCGCTCACCGAGGGACATGAATGGCTTCCAGCAGGAGGGCTTCGGCTGGATGGACATGACCATCCATGAAG 160
```

ENSMUSG00000015970 exon 2 (ORF 2)

```
Mus     GGAAGCGCTGGAGCACAGCCTGCGCCTACTTGCACCCGGTGCTGAGCCGCCCCAACCTCAGGGCCGAGGTCCAGACACTT 80
Rattus  GGAAGCGCTGGAGCACGGCCAGTGCCTACTTGCGCCCAGCGCTGAGCCGCCCCAACCTCAGGGCCGAGGTCCAGACACTT 80
Cavia   GCAAGCGATGGAGCTCAGCCTCGGCCTACCTGCACCCAGCGCTGAGCCGCCCCAACCTCAAGGCTGAGTCCCGGACGCTG 80
Homo    GCAAACGGTGGAGCGCGGCCTGTGCCTACCTGCACCCAGCACTGAGCCGCACCAACCTCAAGGCCGAGGCCGAGACGCTT 80

Mus     GTAAGCAGAGTGCTGTTTGAGGGCACCCGTGCAGTAGGTGTGGAGTACATCAAGGACGGCCAGAGACACAAG 152
Rattus  GTAAGCAGAGTGCTGTTTGAGGGCACGCGAGCAGTGGGCGTGGAGTACATCAAGGACGGCCAGAGCCACAAG 152
Cavia   GTGAGCAGGGTGCTGTTTGAGGGCACCCGTGCAGTGGGCGTGGAATATGTCAAGAACGGCCAGAGCCACAAG 152
Homo    GTGAGCAGGGTGCTATTTGAGGGCACCCGTGCAGTGGGCGTGGAGTATGTCAAGAATGGCCAGAGCCACAGG 152
```

ENSMUSG00000015970_intron_1

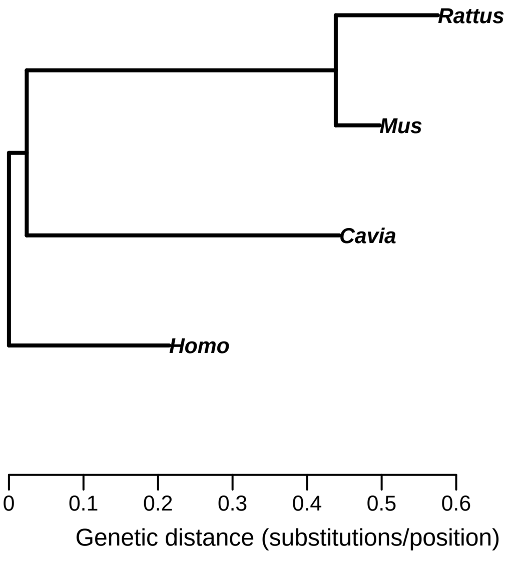

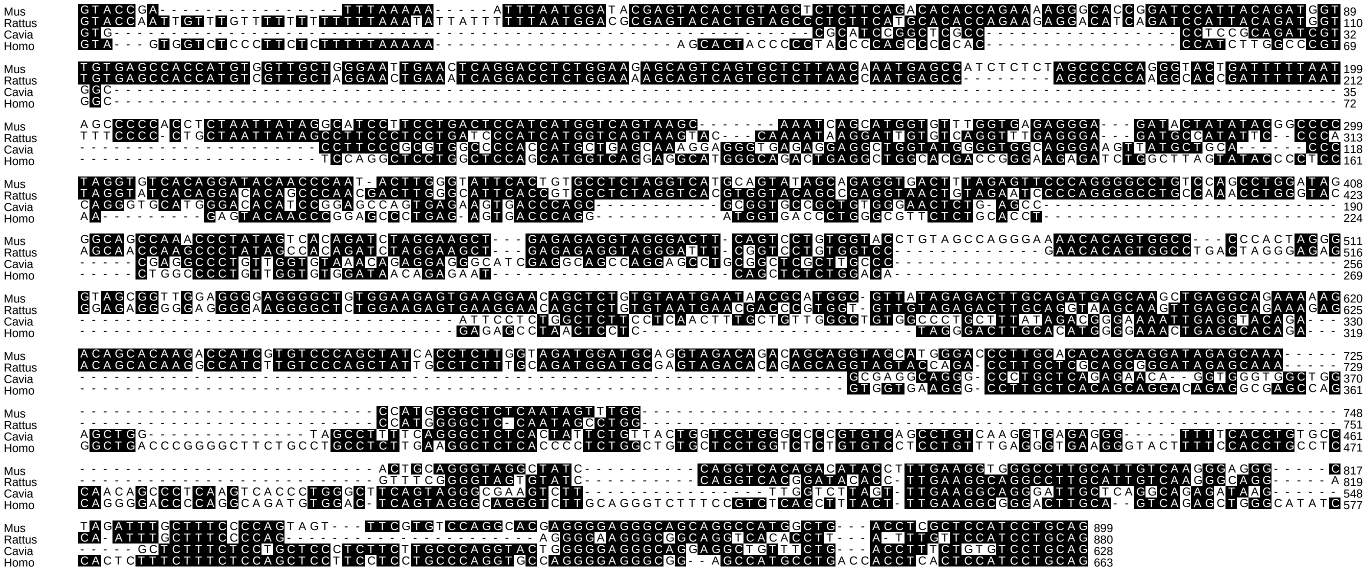
Mus
Rattus
Cavia
Homo

ENSMUSG00000026354 intron 15

Description: lactase (Lct)
Intron number: 15
Mouse chromosome: 1
Upstream exon length: 224
Downstream exon length: 231
Mouse intron length: 804
Intron alignment length: 964
Total murinae branch length: 0.21537
K_score: 0.0568
Scaling factor: 0.74025

ENSMUSG00000026354 exon 15 (ORF 1)

```
Mus     CCTTTTGGCTTCCGGAGGATCTTGAACTGGTTGAAGGAGGAGTACAACAACCCTCTAATTTATGTCACAGAGAATGGAGT 80
Rattus  CCTTTTGGCTTCCGGAGGATCTTGAACTGGCTGAAGGAGGAGTACAATAATCCTCCGATTTATGTCACCGAGAATGGTGT 80
Cavia   CCTTTTGGTTTCAGGAGAATCCTGAATTGGTTGAAGGAGGAGTACAACAACCCTCCAATTTATGTAACAGAGAATGGAGT 80
Homo    CCTTTTGGCTTCAGGAGGATCCTGAACTGGTTAAAGGAGGAATACAATGACCCTCCAATTTATGTCACAGAGAATGGAGT 80

Mus     GTCCCGACGAGGAGACCCAGAACTCAATGACACCGACAGGATCTACTACCTCCGCAGCTACATTAATGAAGCCCTCAAAG 160
Rattus  CTCCAGACGAGGAGAGCCGGAACTCAACGACACCGACAGGATCTACTACCTGCGCAGCTACATTAACGAAGCCCTCAAGG 160
Cavia   GTCCAAGCGTGGAGAGGTAGATCTCAATGACACTGAGAGGATTTATTATCTCCGGAGTTACATCAATGAGGCTCTGAAAG 160
Homo    GTCCCAGCGGGAAGAAACAGACCTCAATGACACTGCAAGGATCTACTACCTTCGGACTTACATCAATGAGGCCCTCAAAG 160
```

ENSMUSG00000026354 exon 16 (ORF 2)

```
Mus     CTGTACGA---GATAAGGTGGACCTTCGAGGGTACACGGTCTGGAGCATCATGGACAACTTTGAATGGGCCACAGGCTTC 77
Rattus  CTGTACAG---GACAAGGTGGACCTTCGAGGCTACACGGTATGGAGCATCATGGACAACTTTGAATGGGCCACAGGCTTC 77
Cavia   CTGTGCAGGATGATAATGTGGACCTCCGTGGATACACGCTTTGGACTCTGATGGACAACTTCGAGTGGGCCACAGGCTTC 80
Homo    CTGTGCAG---GACAAGGTGGACCTTCGAGGATACACAGTTTGGAGTGCGATGGACAATTTTGAGTGGGCCACAGGCTTT 77

Mus     GCAGAGAGGTTCGGCGTGCACTTTGTGAACCGCTCTGACCCTTCTTTGCCAAGGATCCCCAAGGCGTCAGCCAAGGTCTA 157
Rattus  GCAGAGAGGTTCGGTGTGCACTTTGTGAACCGCTCTGACCCATCTTTGCCAAGGATCCCCAAGGCATCAGCCAAGTTCTA 157
Cavia   TCAGAGAGGTTTGGCTTGTACTATGTGAACCACACAGACCCTTCTCTGCCAAGAATCCCTAGAGCATCAGCTAAGTTCTA 160
Homo    TCAGAGAGATTTGGTCTGCATTTTGTGAACTACAGTGACCCTTCTCTGCCAAGGATCCCCAAAGCATCAGCGAAGTTCTA 157
```

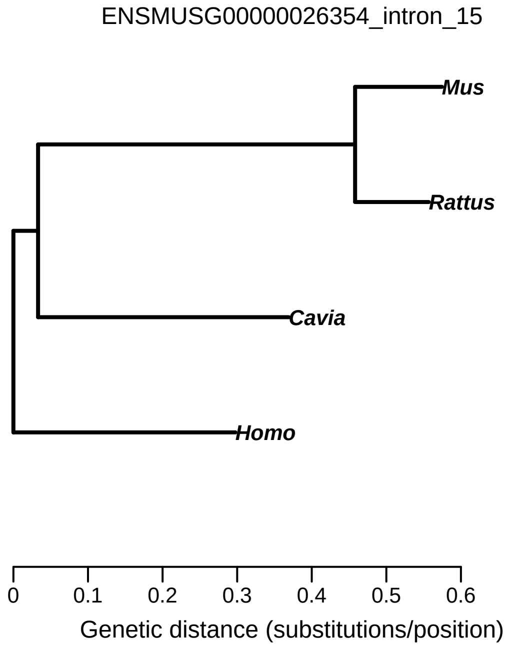

```
Mus     GT---AAGAGCTTTCGCATCCA---------------------------CTGTGTGAGCATCTCAGCATCCCTAGGAGGATGCTCTGGCATGCCAAGGC----------- 69
Rattus  GT---AAGAGCCCTGGCTTCCACCCTGCCTGGCCCAAATGAGCCCCTCGCTGCTGGAGCACCACAACGTCCGGAAGAGGACACTCTGGCAGCCCAAGGG----------- 96
Cavia   GT---GAGAGGCCACCCCTTTC-----------TCCACGGGGCCCCTCACCAATGGAG--------TGTCTACAGGAGGGGAGGCCTGCCCCATAGGAG----------- 77
Homo    GTACGACGGGCCCGCCCTTCCC-----------CAGCGTGCACCTTCCCCTGTTGGAAACATCTGCTATTTGCAGGAGTGTGATACAGCGTCCTTGGGCCACAGTAAGAA 99

Mus     -----------------CTTTGGCTCTTGAGTTTAAGCATTTGTTTGAAGCTAGAATCTAAGGTCTTTTCCCTTCCTAGATTTACGTT---CAAGTAAAACCAGTTGCCT 159
Rattus  -----------------CCTTTGCTCTTG-----AAGCATTTGTTTGAGGCTAGAATCTCAGGTCTTTTTCCTTCCTAGA------------AAGTAACACCCGTTGCCT 172
Cavia   ----------------------------------------------------------------------CTTTCCTAGATCCATG-TCAGCAAACAAATCCAGCTGCT- 115
Homo    GAGAACGATAAGCCTTCTTGCTCCTGTGGGGGTTAAGCTTTGCTTTAGGGCCGCACTCTGA-GTTTTTTTCCTTCCTATGTCTAAGCTCAGCAAGCAAGCCCAGTGGCCT 208

Mus     GCACTGGCAAGACCTTAGTGTCGGACTCGAGTCCTCTGAAACCCTCAGGTTTTCTTGCTTCTTGGTATCTCTGCACATTACTGTCCTGCAGCAGG------CCAAGAGTG 263
Rattus  GAACTGGCTAGACCTGGGTGTCCGACTCGAGTCCTCTGGAACCTTTAGGTTCTCTTGCTTCTTGGCATCT---------------CTACAGCAGG------CCGAGGGCA 261
Cavia   ------ACCAGACCTTAGTCCCTGGCCTGAGTTATCTGCTGCCCCTGACCATCCCTGGTGTCGTA----------------------ACAGCAGGGCCTTCTGGAGGAAG 197
Homo    GATGCCACCAAACCCTAGTTCCTGGCCTGAGTCACTTGCTCCCCTTGATCCTCCCTGATGTCGGA----------------------ACAGCAGTGCCCTCCTGAGAATG 296

Mus     GTCTGGGCTTTTGGTTTCTAAGGTGGTAGGACGGGGGAGATAGCTCAGTTAGTAAAGTGCTCAGTATACAGCCATAAGACCTAAATTAGAATTTAAAACACCCGAGGATC 373
Rattus  GCCTGGGCTTCTTGTTTCCAAGGCCGCAGG-CTTGGGAGAGCACTCAGCTGGTAAAGTGCTCATCGCATACCCATGAGACGTGAATTCAATTCCAAAACCACGGCGGATC 370
Cavia   GTCTAAG---------------------GGGGCTGGGCAGTGGTCTCCAGGATC---TGCTCTGCAGGCAGAAACTGGACA----------------------------- 254
Homo    GTCTGGGT-----AGGGCCCAGGAGGAGTAGGCTGTGTGGTTGCCTGTAGGGTCTCTTAGTCTCCGAGCAAAAGTTTGAGG----------------------------- 372

Mus     ATCTCTGGCTTCTACATACACCCACACATAGCACTCACATGAA--------TACAGACTATGTGTGTGATATCTATGTAGACCCTGGGCTCTACCACAGCCAAGAAAATG 475
Rattus  GTCTCTGGGCCCCACATGCACCCACAC--AGCACTCACACGAACACATGGCTGCACACTATGTGTGTGATACGTATGTAGACCCTCGGTTCTACTACAGCCAAGGGAATG 478
Cavia   --CCCTGAGCTCTATCCAAAT--------AACTTGGATGGGACTGACAGAGCAAAGAAAGTG------ATATTGAGTCAG----------CCACTCCAGCCAAGGAAATG 338
Homo    --CTCTTGGCTCAGCCCTAAC--------AACTTGAGATGGGACCATGAGGATAAGAGAATGGAACAAATGCTGAGTAAAAGAGCAAGCAATGCACCAGCAAAGGAAATG 472

Mus     GCCAGAGGTCCTATTA-CAGCTCAGATCTTTTGCTTCTAAAAGTTCCTGCCTCTCCATGGG--AGAG------TATTTCAGGGTCCTGACTCAGATCAGTCTCCTGAGCT 576
Rattus  GCCAGGGGTCCTATTA-CAGATCAGATCTTTTGCTTCTGGAAGCTCCTGTCTCTCTGTGGGGAAGAG------TATTTCAGGGCCCTGAC-----TCAGTCGCCTTAGCT 576
Cavia   CTTAGTGTTCTGCTAC-------------------------------TGATTTTCCATAGGCAGGAG--TTGTTGCTCTACGGCTCTGACCCTGACCC-TGACCCTGCTG 414
Homo    ATTAGAAAAATCCTACCCAGCTGAAATATATTCTATCTAAGGG----CTATTTTCTACAGGGAGAAGCATTAACATTCTAAGGCACTGACCAAGATCA-T---------- 567

Mus     ACAGAGGAAGGCAGTGGGGTTA------------------------------------------GCTCAGGCTATCTAAGTTCTAGAAAGACACTGATGGCCCTGCATAT 644
Rattus  ACAGAAGAATGCAGTAGGGTTG--------------------------------------TTCCACTCAGAACTGAGCCTTTCTAGAAAGATACCAGTGGCCCTGCA-GT 647
Cavia   GGGGAGGGGTAAGGTGGGGCAGTGGCACAGAGGTTAGCTGAGGGGTTCCACGTTCACAACTCCCACCAAGGCTT------------------------------------ 488
Homo    -----GGATAGAAGTGTAGGTGACCCAGGGAAATTAGTCCA-----CCCATGTTCCCGACTCCCATTCAGGGCTGAGCCTTTCT--AAAGATGCTGGTGGCTCTACATCT 665

Mus     CCA-CGATAGAGGACCTGCGAA-----------------GACACAGGATGGGCTCTCAGA---------GTTGAACTCACACTCTACTATTAGGACAGTGGGGACAACCA 727
Rattus  CCA-GTGTAGAGGACCTGCTAA-----------------GACACAGGGTGGGCTCTCAGA---------GCTGAACTCACACTCTACTATTCGGGCAGTGGGGACAGCCA 730
Cavia   ------------AGCCTGCCAACAGCCAT----------GAAAAGGGAGGGCTGCCCACA-----------------TTCACTATGCAAGTCGGGAGCACGGGAGAAGTG 559
Homo    CCATCCACAGAGGACCCGCTAACAGTCACTGCATGCAAAGAAAGGGGCAGCTTTCTCACAGAGCTAAAGCCAAAGCTTACCGCCATCTGTGAAGGCGGTGGGGAGAGCAG 775

Mus     CGA----GCCTGGCTTCAGGAGGCCTGGCACACAGGTGTCTTGATGTGAAGAAGACTCACACCAGG---CCTGTGTTTGAGCAG 804
Rattus  TG-------------------------------AGGTGTCCTGCTGTGAAGAACACTTACACCAGG---CCTGTGTTTGAGCAG 780
Cavia   TGGGCA-ATTGGGTTGCAGGAGGCCTGGTGGCAAGGTGTCCCGAGAC-----AAGTTCACACT------CTGGCCTGTGGGTAG 631
Homo    TAGGCAGACCTGGCTTCCGAAGGCCTGGCAGGAAGCTGCCTTATGAGATAGCAAACTTATGCCTAGTCCTCTGTGTTTGAGCAG 859
```

ENSMUSG00000021113 intron 5

Description: snRNA-activating protein complex subunit 1 (Snapc1)
Intron number: 5
Mouse chromosome: 12
Upstream exon length: 159
Downstream exon length: 69
Mouse intron length: 1545
Intron alignment length: 2618
Total murinae branch length: 0.29669
K_score: 0.08669
Scaling factor: 0.74132

ENSMUSG00000021113 exon 5 (ORF 0)

```
Mus     GAAATGCTGAATGTTCACGATCATTATCAAAATATGAAGCATGCTATTTCAGCAGACAAGTCCATGCCAGACAGAGCTCT 80
Rattus  GAGATGCTGAATGTTCACGACCATTATCAAAATATGAAGCATGCCATTTCGGCAGACAAGTCCACGCCAGACCGAGCTCT 80
Cavia   GAAATGCTGAATGTTCATGAGCATTATCAGAACATGAAACGTATAATTTCGACTGATAAGTCCAAACCAGACAAAACGCT 80
Homo    GAAATGCTGAATGTTCATGATCATTATCAGAACATGAAACATGTAATTTCAGTTGATAAGTCCAAGCCAGATAAAGCCCT 80

Mus     CAGTCTGGTAAAGGAGGACTTTTTTGAGAACATTAAGAACATAGTTTTGGAGCATCAGGAGTGGCACAAGGAACGGAAG 159
Rattus  CAGTCTAGTCAAGGAGGATTTTTTTGACAACATTAAGAACATAGTTTTGGAGCATCAGGATTGGCACAAAGAAAGGAAG 159
Cavia   CAGCTTGATAAAGGATGACTTTTTTGATAACATTAAGAACATAGTTTTGGAACATCAGCAGTGGCACAGAGACAGACAG 159
Homo    CAGCTTGATAAAGGATGATTTTTTTGACAATATTAAGAACATAGTTTTGGAGCATCAGCAGTGGCACAAAGACAGAAAG 159
```

ENSMUSG00000021113 exon 6 (ORF 0)

```
Mus     AATCCCTCCTTAAAACCCAAACTTAAAGATGGAGAAGAGAACGGTGAAGGCTCTTCAGAGGAGCCAGAG 69
Rattus  AATCCCTCCTTAAAACCGAAACTTAAAGATGGAGAGGAGGATGGCGAAGGCCCCTCAGAGGAGCCGGAG 69
Cavia   AATCCATCCTTAAAATCAAATCTTAAAGATGAAGAAGAACAGAGAGAAGGAGATACACGAGAATCACAG 69
Homo    AATCCATCCTTAAAGTCAAAAACTAATGATGGAGAAGAAAAAATGGAAGGAAATTCACAAGAAACGGAG 69
```

ENSMUSG00000021113_intron_5

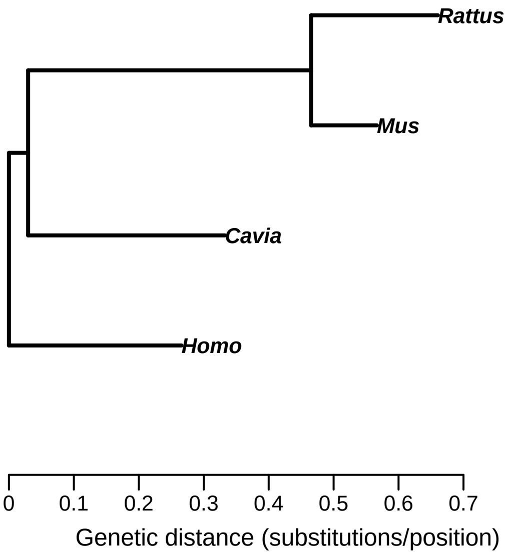

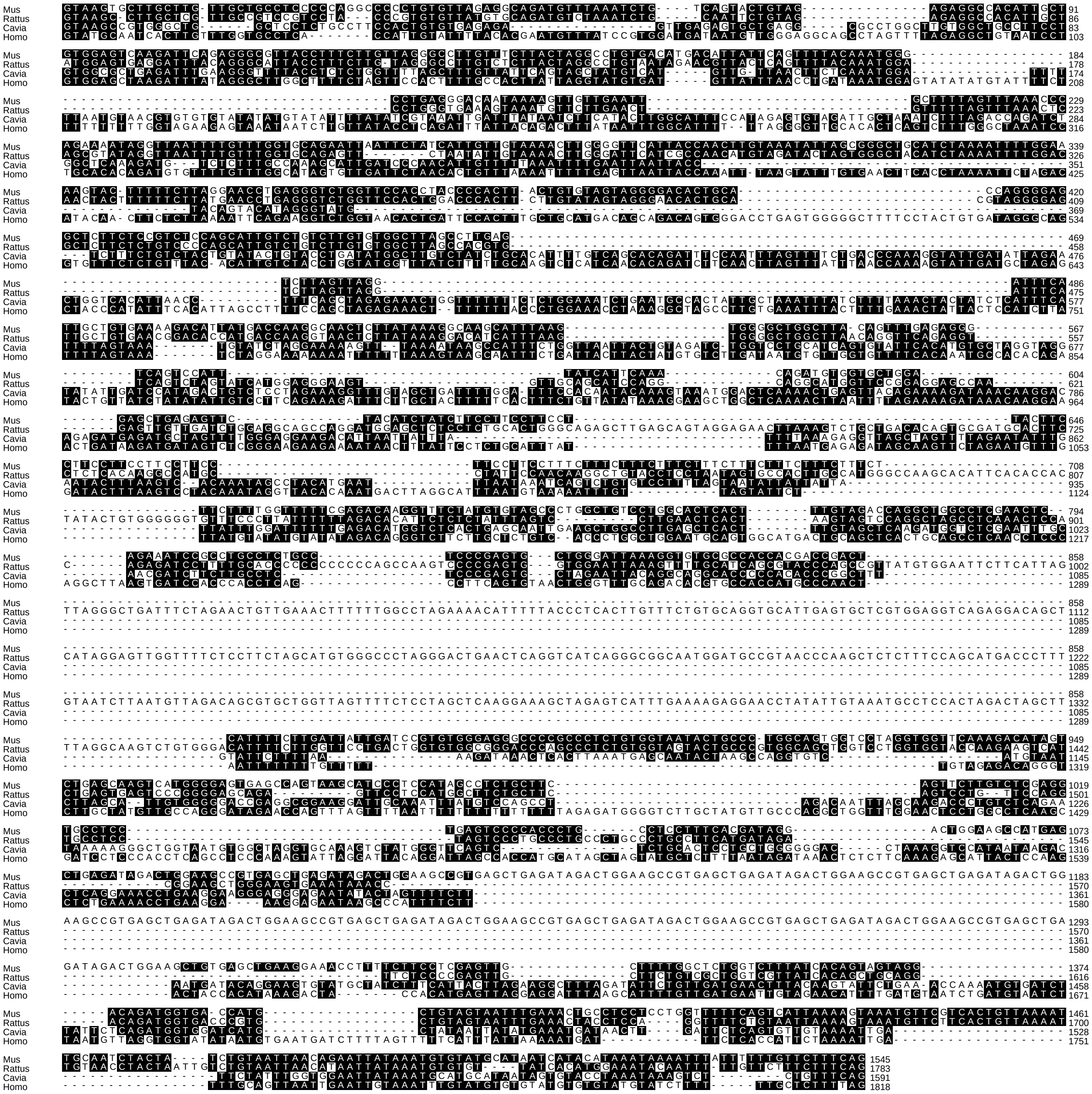
Mus
Rattus
Cavia
Homo

ENSMUSG00000052026 intron 10

Description: Sodium-dependent proline transporter (Slc6a7)
Intron number: 10
Mouse chromosome: 18
Upstream exon length: 132
Downstream exon length: 100
Mouse intron length: 453
Intron alignment length: 519
Total murinae branch length: 0.19567
K_score: 0.07255
Scaling factor: 0.74203

ENSMUSG00000052026 exon 10 (ORF 0)

```
Mus     TTTGCCTTTCTAGAAACCATAGTGACTGCAGTGACTGATGAGTTCCCATACTACCTGCGGCCCAAGAAGGCAGTGTTCTC 80
Rattus  TTTGCCTTTCTGGAAACCATAGTGACTGCAGTGACCGATGAGTTCCCATACTACCTACGGCCCAAGAAGGCAGTGTTCTC 80
Cavia   TTTGCCTTTCTGGAGACCATTGTGACAGCAGTGACAGATGAGTTCCCATACTACCTGCGGCCCAAGAAGGCGGTGTTCTC 80
Homo    TTTGCTTTTCTGGAGACCATTGTGACAGCTGTGACAGATGAGTTCCCATACTACCTGCGGCCCAAGAAGGCGGTGTTCTC 80

Mus     AGGACTCATCTGTGTGGCCATGTACCTGATGGGACTGATTCTCACCACTGAT 132
Rattus  AGGCCTCATCTGTGTAGCCATGTACCTGATGGGACTGATCCTCACCACCGAT 132
Cavia   GGGCCTCATCTGCGTATCCATGTACCTGATGGGGCTGATCCTCACCACTGAC 132
Homo    AGGGCTCATCTGCGTGGCCATGTACCTGATGGGGCTGATCCTCACCACTGAT 132
```

ENSMUSG00000052026 exon 11 (ORF 0)

```
Mus     GGGGGTATGTACTGGCTGGTCCTTTTGGATGACTACAGCGCCAGCTTCGGACTCATGGTGGTGGTGATCACCACGTGCCT 80
Rattus  GGGGGGATGTACTGGCTGGTCCTTCTGGATGACTACAGCGCCAGCTTCGGACTCATGGTGGTGGTGATTACCACATGCCT 80
Cavia   GGGGGCATGTACTGGCTGGTCCTTCTGGATGACTACAGCGCCAGCTTCGGGCTCATGGTGGTGGTGATCACCACGTGCCT 80
Homo    GGGGGCATGTACTGGCTGGTCCTTCTGGATGACTACAGCGCCAGCTTCGGGCTGATGGTGGTGGTTATCACCACGTGCCT 80

Mus     CGCTGTCACCCGGGTATATG 100
Rattus  CGCTGTCACCCGGGTATACG 100
Cavia   CGCTGTCACCCGGGTATACG 100
Homo    TGCCGTGACACGGGTGTATG 100
```

ENSMUSG00000052026_intron_10

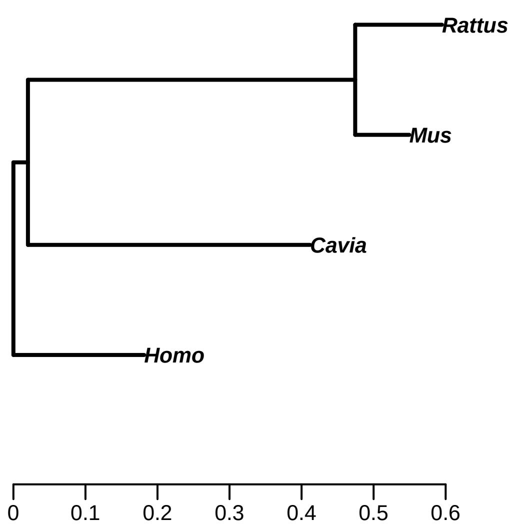

Genetic distance (substitutions/position)

```
Mus     GTGAGTGAC-CTTCAGGGAGGCGCCAAGCTGGGAAGCTGGGCAGGGTGTTGTTCTTTGC-----AGCAGTATTTTTGTCCAGCTGAGAAGTCACTGAATAGCTTCT----  100
Rattus  GTGAGTGGC-CTGCAAGG-GGCACCAAGTTGGGAAGCTGGGCAGGGTGTCGTTTACCACCT---GAGGGTATTTTGGGCTGGCTGAGAAGTCACCAAATAGCTTCC----  101
Cavia   GTGAGTGGCCTGACTGGA--------AGAAGGCAGGCTGGGCAGGGAGGGAC-GGTGCTTGCAGAA--------GTCCTGGCCGAGCAGTTTCTGAG--ACTTCTGCCT  91
Homo    GTGAGTGGCGCTACAGGG--------AGGATGGCAGGTGGGCGGGACAAGGGCAGACGCCTGCAACGAGATCTCTGGCCCAGCTGAGCAGTTGCTGGGCCCCCTCCATCT  102

Mus     --------------------GGTCCTACCAGGGCCTGTTTCCTGCGGCAGGCACCTTGGTCTATGG---TGGAGTTCTGCCTCTGTAG--TTAATTTGAAAGACCTGGGT  185
Rattus  --------------------GGTCCTACCAGGGCCTGTTTCCTGTGGCAGGCAACTTGGTC--------TTGAGTTCTGGCTCTGGAA--TCAGTTTGAAAGACCTGGGT  181
Cavia   TCCTCAGTGTGGAGGACCT-GTCCCT-------CCTGTCTCCAAAGGAGGGCAG-----------------AATCTCAGAGTGTGGAG-------------ACATGGGT  162
Homo    TCCTCTTTGCAAGGAACCCAGTCCCTCCCCGGCCCTATCTCCCAAGGGAGGCAGTTTGGCCTTGTGATCCAGAGTCCTGGCTGTGGAGATTTAGTCTGCAAGATCTGGGT  212

Mus     TCAAACCTCACTCCTCTACTTGCTGAGAAA--CTTCAGACAAGCGACTTCTCCTCTCTGAACCCATTTTTCCTCATGCATACATGGGTCCTGT--------GAGGCTGCT  285
Rattus  TCAAACCTCACTCCTCCATTCGCTGAGTAA--CTCCAGACAAGTGACTTCCCCTCTCTGAACCCATTTTTCCTCATGCATGCATGGGTCCTGTTAGGAGACTAGGCTGCT  289
Cavia   TCAAACCTCG-TCCTGTACATGCTGAGGGC---------------CTTGGCCCCCTGAGTCTG-TTCTCCTCTTTGGTAAATGGGGGTTCT-----------GCCATA  243
Homo    TCAAACCTGACTCCTGCACTTGCTGAGGGGGCTTCAGGATAAATGATTTCCCCTCCCTGAGATTGTTTTTCCTCATCAGTAAAAGGAGGGTTCTATAATAGACGGCTGTC  322

Mus     GGGAAGCAGGAAGGGACATTGTCTCAACAGCTCTGAGCAGGCAGACCGTGGCATGGTTTT------CTACTCCCTCA------------GCCCTGGCCACTTCCCGCTCT  377
Rattus  GGGTAGCAGAAAGGGACATCATCTCTACAGCACTGAGCAGGCTGACCATTCCATGGATTTTACCCCCAACCCCCTCAG------CATTTGCCCTGGCTGCTTCCCACTCA  393
Cavia   TGGCGCTTTTGGGAGGTGATGCAGGTGCTTTGGTGGGCAGGTGACCAGTG----------TGACGCTGTCATCATCTGGCTCTCCTGGCAGCCTGGCTACTTCCTGCTAA  343
Homo    TTGGGACTCAGATAAGACAAGGCCTAGAAGGGCTGGGCTGGCAGCCAGTGG---------TCACCCTGTCATCTTGTG------CTGTCAGCATGGCTGCTTCCTGCTCA  417

Mus     CCTCCTGCCAGAAGGAGATGGGC--AAACAGAGGGGGAGCCTGAGGGTGGAGGC-CCAGCGCTGTGCTTTGTGTTCTAG  453
Rattus  C-TCCTGACTGAAGGAGATGGGCTACAGCAGAGACAGAGACGGAGGCTGGAGGC-CCAGTGCTGTGCTTTGTGTCATAG  470
Cavia   CTTCCTGCTAAGAAAAG--GAGGTGTGGCAGAG------------GGTGGGGGA-CCAGCACTGCAACTTTTATTTCAG  407
Homo    CTTCTTGCCAGGAGAAG--GGGCTGTAGCAGAGAACGAGGCCCAGGAAGGGGACACCAGAACTGTGCTT-GTGTTTTAG  493
```

ENSMUSG00000068008 intron 9

Description: Long palate, lung and nasal epithelium carcinoma-associated (Bpifb3)
Intron number: 9
Mouse chromosome: 2
Upstream exon length: 54
Downstream exon length: 171
Mouse intron length: 1314
Intron alignment length: 2147
Total murinae branch length: 0.18895
K_score: 0.05991
Scaling factor: 0.74271

ENSMUSG00000068008 exon 9 (ORF 0)

```
Mus     GTTCCCAGGAATGTCCCTCTGACAACTACTGACCTGGCAGCTTTGGCTCCTGAG 54
Rattus  GTTCCCAGAAATATCCCGCTGACAACTACTGACCTGGCAGCTTTGGCCCCTGAG 54
Cavia   GTTCCCAAGAACATTCCGCTGACAACCACATACCTGGCAGTTTTGGTCCCAGAG 54
Homo    GTTCCCAGCGATGTCCCACTGACAACTACAGACCTGGCAGCTTTGCTCCCTGAG 54
```

ENSMUSG00000068008 exon 10 (ORF 0)

```
Mus     GCCCTGGGGAAACTGCCCCCTGCTCAGCACCTCCTGCTCTCGCTGCGGGTGACAAAATCGCCCATGGTCCTGCTGCAGAA 80
Rattus  GCCCTGGGGAAGCTGCCCCCTGGTCAGCACCTCCTGCTCTCGCTGCGGGTAATGAAGTCACCCATGATCCTGCTGCAGAA 80
Cavia   GCCCTGGGGAAGTTGCCCCCAGGCCAGCAGCTCCTACTGTCACTGAGGGTGAAGGAAGCACCCACTGTCACACTACAGAA 80
Homo    GCCCTGGGGAAGCTGCCCCTGCACCAGCAACTCCTACTGTTCCTGCGGGTGAGGGAAGCTCCCACGGTCACACTCCACAA 80

Mus     CAAGAAGGCCACAGTCTCCATCCCAGTCACCATCCATGTGCTGTCTTCTGTCCCTCAAGGAACTCCTGTAGCCCTTTTCC 160
Rattus  CAAGAAGGTCACAGTCTCTATCCCAGTCACCATCCACGTGCTGTCTTCTGTCCCTCAAGGAACTCCTGTAGCTCTTTTCC 160
Cavia   AAAGAAGGCCACAGTCTCCATCCCAGCCACCATCCATGTACTATCTTCTGTCCCTCAGGGGACTCCTACAGCCCTCTTCC 160
Homo    CAAGAAGGCCTTGGTCTCCCTCCCAGCCAACATCCATGTGCTGTTCTATGTCCCTAAGGGGACCCCTGAATCCCTCTTTG 160
```

ENSMUSG00000068008_intron_9

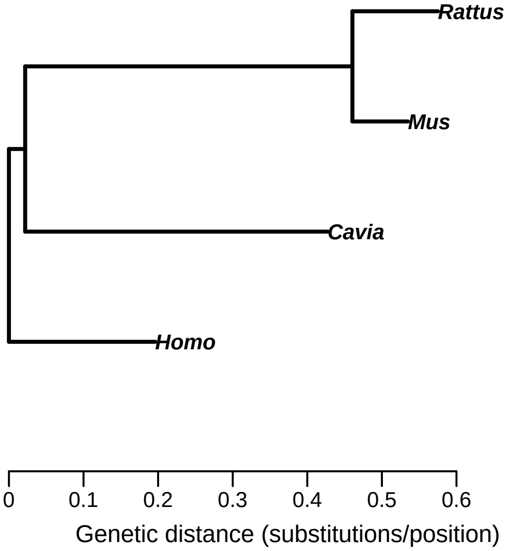

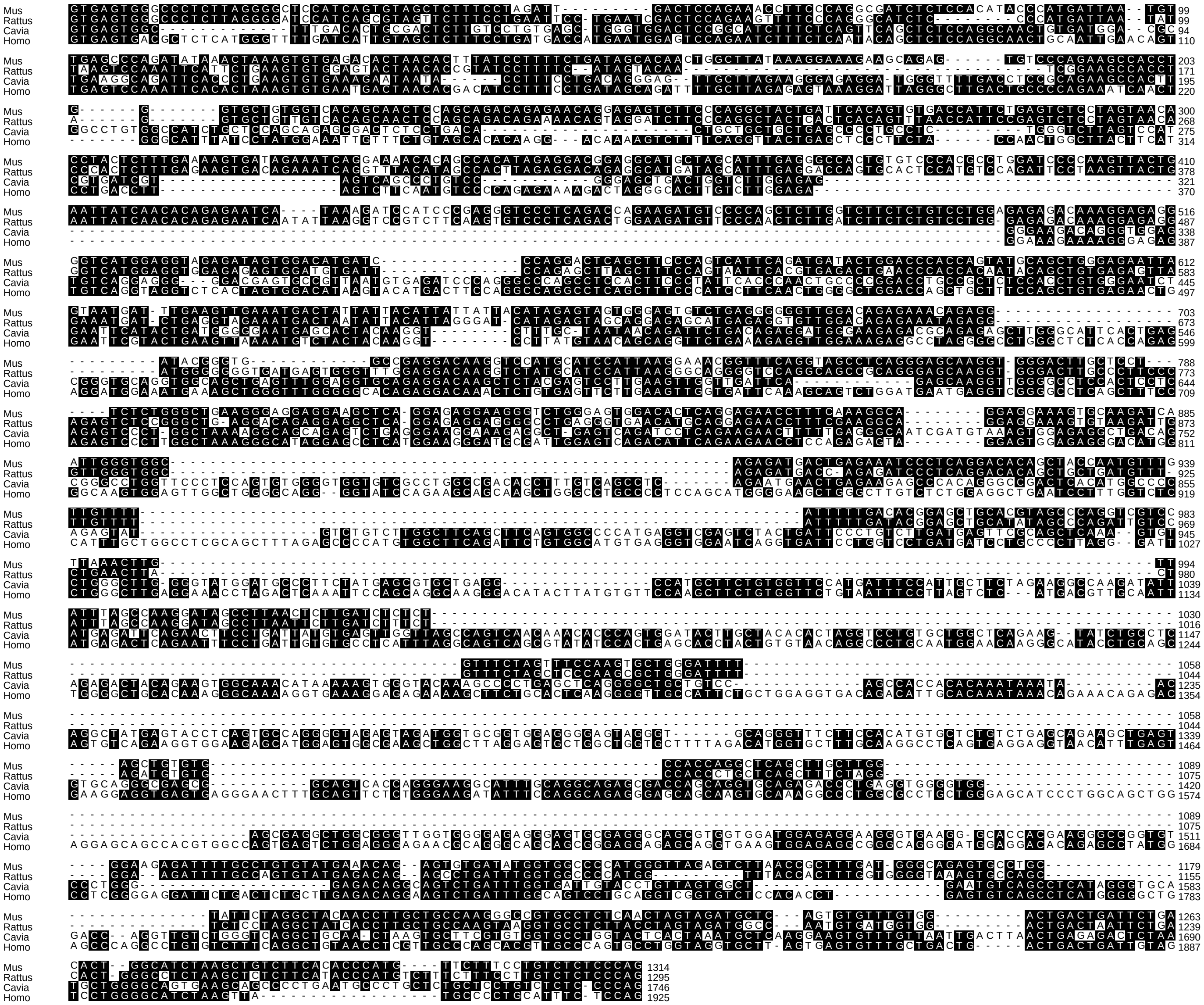
Mus
Rattus
Cavia
Homo

ENSMUSG00000032122 intron 5

Description: Sugar phosphate exchanger 2 (Slc37a2)
Intron number: 5
Mouse chromosome: 9
Upstream exon length: 136
Downstream exon length: 77
Mouse intron length: 411
Intron alignment length: 692
Total murinae branch length: 0.19731
K_score: 0.08329
Scaling factor: 0.74344

ENSMUSG00000032122 exon 5 (ORF 1)

```
Mus     TGGAATCTTTGGGGAGCGGCTGCCCCTGCGTTACTACCTCTCGGCTGGAATGGTGCTAAGTGGCCTATTCACCTCCCTCT 80
Rattus  TGGAATCTTTGGGGAGCGGCTGCCCCTGCGTTACTACCTCTCAGCTGGAATGGTGCTCAGTGGCCTATTCACCTCCCTCT 80
Cavia   TGGGATTTTTGGGGAGCGGCTCCCCCTCCGTTACTACCTTTCTGCCGGAATGTTGCTCAGTGGCCTATTCACCTCCCTCT 80
Homo    TGGGGTTTTTGGGGAGCGGCTTCCGCTCCGTTACTACCTCTCAGCTGGAATGCTGCTCAGTGGCCTTTTCACCTCGCTCT 80

Mus     TTGGCCTGGGGTACTTCTGGAATATCCACATGCTCTGGTACTTTGTGCTCATCCAG 136
Rattus  TCGGCCTGGGATACTTCTGGAATATCCACATGCTCTGGTACTTCGTGCTCATCCAG 136
Cavia   TGGGCCTGGCATATTTCTGGAACATCCACATGCTCTGGTACTTTGTGCTCATTCAG 136
Homo    TTGGCCTGGGATATTTCTGGAACATCCACGAGCTCTGGTACTTTGTGGTCATCCAG 136
```

ENSMUSG00000032122 exon 6 (ORF 0)

```
Mus     ATCTGCAACGGGCTTGTGCAGACTACAGGCTGGCCATCTGTGGTGACCTGTGTTGGCAACTGGTTTGGGAAGGGAAA 77
Rattus  ATCTGCAATGGCCTTGTTCAGACTACGGGCTGGCCGTCTGTGGTGACCTGTGTTGGCAACTGGTTTGGGAAGGGAAA 77
Cavia   ATCTGCAATGGACTTGTCCAGACCACCGGCTGGCCCTCCGTCGTGGCCTGTGTTGGCAACTGGTTCGGGAAGGGGAA 77
Homo    GTCTGTAATGGACTCGTCCAGACCACAGGCTGGCCCTCTGTGGTGACCTGTGTTGGCAACTGGTTCGGGAAGGGGAA 77
```

ENSMUSG00000032122_intron_5

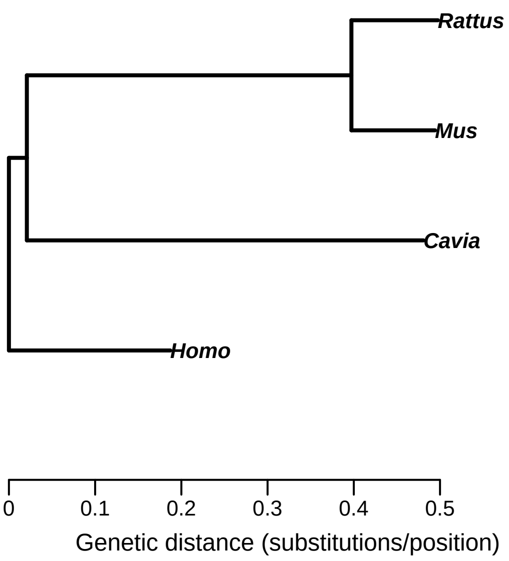

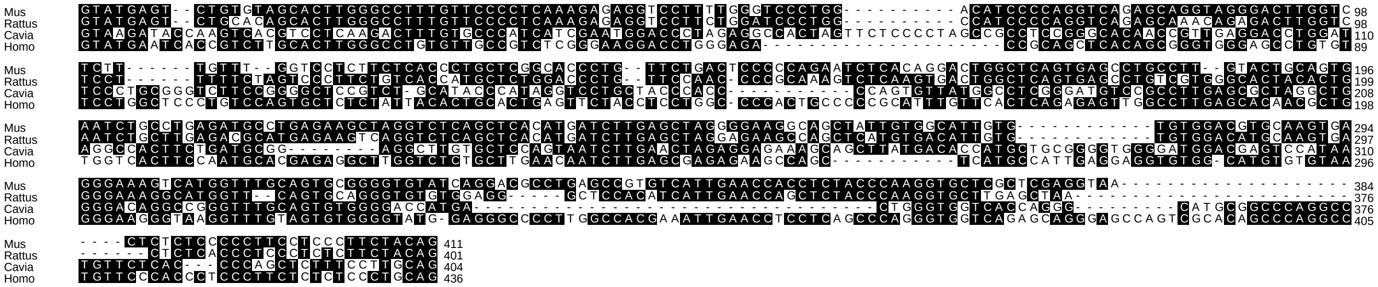
Mus
Rattus
Cavia
Homo

ENSMUSG00000030284 intron 3

Description: Cysteine-rich with EGF-like domain protein 1 Precursor (Creld1)
Intron number: 3
Mouse chromosome: 6
Upstream exon length: 111
Downstream exon length: 92
Mouse intron length: 297
Intron alignment length: 467
Total murinae branch length: 0.17921
K_score: 0.04268
Scaling factor: 0.74441

ENSMUSG00000030284 exon 3 (ORF 1)

```
Mus     TGAGACCCGCCTGGTGGAGGTGCTGGAGGGCGTGTGCAGCAGGTCAGACTTCGAGTGCCACCGCCTGCTCGAGCTGAGCG 80
Rattus  TGAGACCCGCCTGGTGGAGGTGCTGGAGGGTGTGTGCAGCAAGTCAGACTTCGAGTGCCACCGCCTGCTAGAGCTCAGCG 80
Cavia   CGAGACGCGCCTGGTGGAAGTGCTGGAGGCTGTGTGTGCCAAGTCGGACTTCCAGTGCCACCGCCTGCTGGAGCTGAGTG 80
Homo    TGAGACCCGCCTGGTAGAGGTGCTGGAGGGTGTGTGCAGCAAGTCAGACTTCGAGTGCCACCGCCTGCTGGAGCTGAGTG 80

Mus     AGGAGCTGGTGGAAAACTGGTGGTTTCACAG 111
Rattus  AGGAGCTGGTGGAAGCCTGGTGGTTCCACAG 111
Cavia   AGGAGCTGGTGGAGAGCTGGTGGTTTCACAA 111
Homo    AGGAGCTGGTGGAGAGCTGGTGGTTTCACAA 111
```

ENSMUSG00000030284 exon 4 (ORF 1)

```
Mus     GCAGCAGGAAGCCCCCGACCTCTTCCAGTGGCTCTGTTCCGATTCCCTGAAGCTCTGCTGCCCCTCTGGCACCTTTGGGC 80
Rattus  GCAGCAAGAAGCCCCTGACCTCTTCCAGTGGCTCTGTTCAGATTCCCTGAAGCTCTGCTGCCCCTCTGGGACTTTCGGTC 80
Cavia   GCAGCAGGAAGCCCCTGACCTCTTCCAGTGGCTCTGCTCAGACTCTCTGAAGCTCTGCTGCCCTGCTGGCACCTTCGGAC 80
Homo    GCAGCAGGAGGCCCCGGACCTCTTCCAGTGGCTGTGCTCAGATTCCCTGAAGCTCTGCTGCCCCGCAGGCACCTTCGGGC 80

Mus     CCTCCTGCCTGC 92
Rattus  CCTCCTGCCTGC 92
Cavia   CCTCCTGTCTCC 92
Homo    CCTCCTGCCTTC 92
```

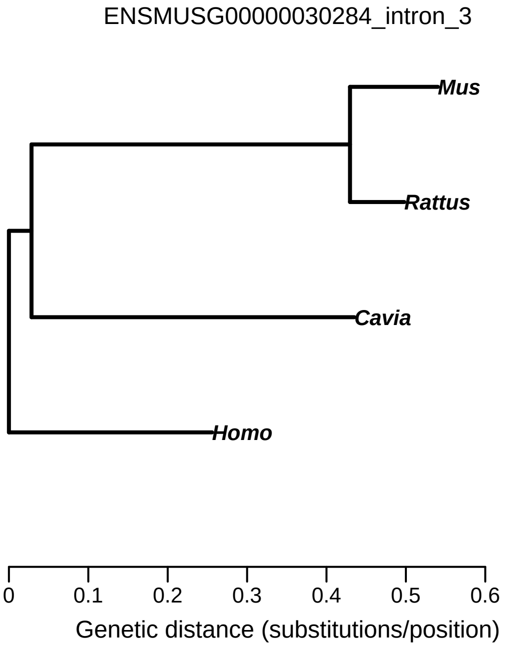

```
Mus     GTGAGGAGTGGGAGG---------------GGTTACAGATATGCCCCGGTGATGTGGCTTTGACTTGGCCAAGAAGCAGTTGCATG-------------GCAGCTTGGTT 82
Rattus  GTGAGGGGCGGGAGG--------------GGCTCACAGACAGGCCC--------TGGCTTTTACTTGACCAAGAAACAGTTGCATG-------------ACAGCCTAGTT 75
Cavia   GTGAGTGGCAAGGGGCCTCC--------T-------------------------GGGACCCAGCGTGGC--AAGAGCAGAGGGTTGCACAGTGAG--CCAGGGCCTGGTT 73
Homo    GTGAGTGGCAAAGGGCCTTCCCTGGAAGTGGGTCACAGGTGAGGCCTGGTGAT-AAGGCCTGATTTGGCCGAGAAGCAGGGGGGTGCATGCTGGGGCCCATGTCCTGGTT 109

Mus     TGGAGCCTTCCCAATCCAGATCTG---AGGAGTTGCCCCTGGGTACCAGGCCCTCACACTGAGCCTCTTTACTCCTCTGCC-------CAAAGGGCCTTCCTTGGTCAAG 182
Rattus  GAGAGCCTTCCAAATCTAGATCTG---AAGACTTGCCTCTGGGTACCAGGCCCACAGTCTGGGATTGTTTACTCCTCTGCC-------CAAAGGG----CCTTGATCAAC 171
Cavia   GGGCATGTGCTAAGCCCACATCTG---AGGACTTTCTGGGGGCATCTAAACTCCTGCTCTAAGTGTGTTTACCTGTTTGCCAGATGGGCAAAGGA----CA-TGGTCAGG 175
Homo    GTGCTCCTTCCAAACCCAGGTCTGCTAAGAACTTGCCGGGGGACTTGCGCTCCACTTTGAGCCTCAGTTTACCCTTCTGCCAAATGGGGAAAGGG----CATTGGTCAGA 215

Mus     GGG-TTCCCAGGACACATGCCCAAGTTGAGCTGAATT------------------------------CCAGCTTCAGGGAGGGCAGGAGCAGGCAGCCACTCCCA----- 256
Rattus  TGG-TTCCTGGGACATAGGCCCATGTTGAGCTGAATT------------------------------CCAGATTCGGGGAGAGTAGGAGAAGGCAGCCACTCCCGCACTG 250
Cavia   TGGCCCTCTGGGGTTTATGCTCCTGATAGGTTAAATTGGCAGGGCGTGGTGGCAAGCACCTATAATCCCAGCATTTGGGAGACTGAGGCAGGAGGATTGATGAGAGTTCA 285
Homo    TGGCCTTTTGGGTCTTATGTCCAAGCTGGGTTGAATC------------------------------ACAGATTCAGGCATGGGGGAATGGGAACAGCACTTATGACACT 295

Mus     -CAGCACCCT-----------------------------------------------------------------------------CGCCTTTCAGC------------ 276
Rattus  TCAGCACCCT-----------------------------------------------------------------------------CGCCTTTCTGC------------ 271
Cavia   AGACCAACCTGAACTATATCTTGAGACCCTGTCTCAAAAAAAAAAAAAGGGTCAAATGGCAGATTTCTTGGGAGGTAGGGTGGACAGCATCTCTGAACCAGGTCAGTGCT 395
Homo    ATCTCAGCAC-----------------------------------------------------------------------------CTCCTCCCCAC------------ 316

Mus     -CTTTCTGCCCCTTTCTC-----ACAG 297
Rattus  -CTTTCTGCCCCTTACTC-----ACAG 292
Cavia   TCTCCCTGTTACCTGCCTCTGCTTCAG 422
Homo    -CTCCCTCCACCCTGCCCCTGCCTCAG 342
```

ENSMUSG00000032737 intron 19

Description: Phosphatidylinositol-3,4,5-trisphosphate 5-phosphatase 2 (Inppl1)
Intron number: 19
Mouse chromosome: 7
Upstream exon length: 90
Downstream exon length: 114
Mouse intron length: 645
Intron alignment length: 748
Total murinae branch length: 0.19946
K_score: 0.07516
Scaling factor: 0.74488

ENSMUSG00000032737 exon 19 (ORF 2)

```
Mus     GTTGCACTGATGACATTGTTACCAGTGACCATTCTCCTGTGTTTGGGACATTTGAGGTTGGAGTTACTTCCCAGTTCATC 80
Rattus  GTTGCACTGATGACATTGTTACCAGTGACCATTCTCCTGTGTTTGGGACATTTGAGGTTGGAGTGACTTCCCAGTTCATC 80
Cavia   GTTGTACTGATGACATCGTCACCAGCGACCATTCCCCTGTGTTTGGAACATTTGAGGTTGGAGTTACCTCTCAGTTTATC 80
Homo    GTTGCACTGATGACATCGTCACCAGCGACCATTCCCCCGTGTTTGGGACATTTGAGGTTGGAGTTACCTCCCAGTTCATC 80

Mus     TCCAAGAAAG 90
Rattus  TCCAAGAAAG 90
Cavia   TCCAAGAAAG 90
Homo    TCCAAGAAAG 90
```

ENSMUSG00000032737 exon 20 (ORF 2)

```
Mus     GTCTCTCTAAGACCTCAGATCAAGCCTACATTGAGTTTGAGAGTATCGAAGCCATTGTGAAGACAGCCAGCCGCACCAAG 80
Rattus  GTCTCTCTAAGACCTCAGACCAGGCCTACATTGAGTTTGAGAGCATCGAGGCCATCGTGAAGACGGCCAGCCGCACCAAG 80
Cavia   GGCTCTCAAAGACTTCAGACCAGGCCTACATTGAGTTTGAGAGCATTGAAGCCATTGTGAAGACAGCCAGCCGCACTAAG 80
Homo    GGCTCTCAAAGACTTCAGACCAGGCCTACATTGAGTTTGAGAGCATCGAGGCCATTGTGAAGACAGCCAGCCGCACCAAG 80

Mus     TTCTTCATTGAGTTCTATTCTACCTGCTTGGAAG 114
Rattus  TTCTTCATTGAGTTCTATTCTACCTGCTTGGAAG 114
Cavia   TTCTTCATCGAGTTTTATTCCACTTGCCTGGAGG 114
Homo    TTCTTCATCGAGTTCTACTCTACCTGCCTGGAGG 114
```

ENSMUSG00000032737_intron_19

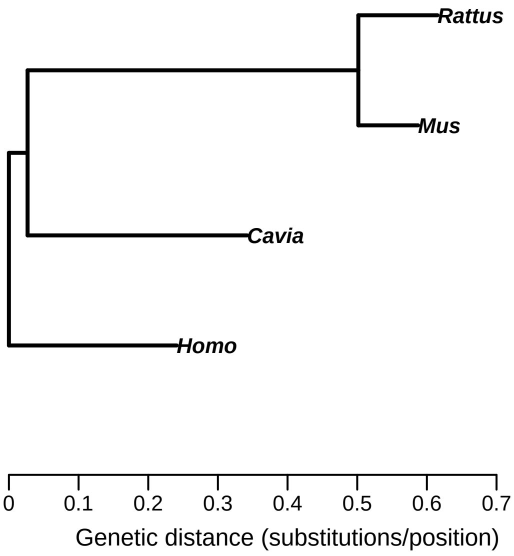

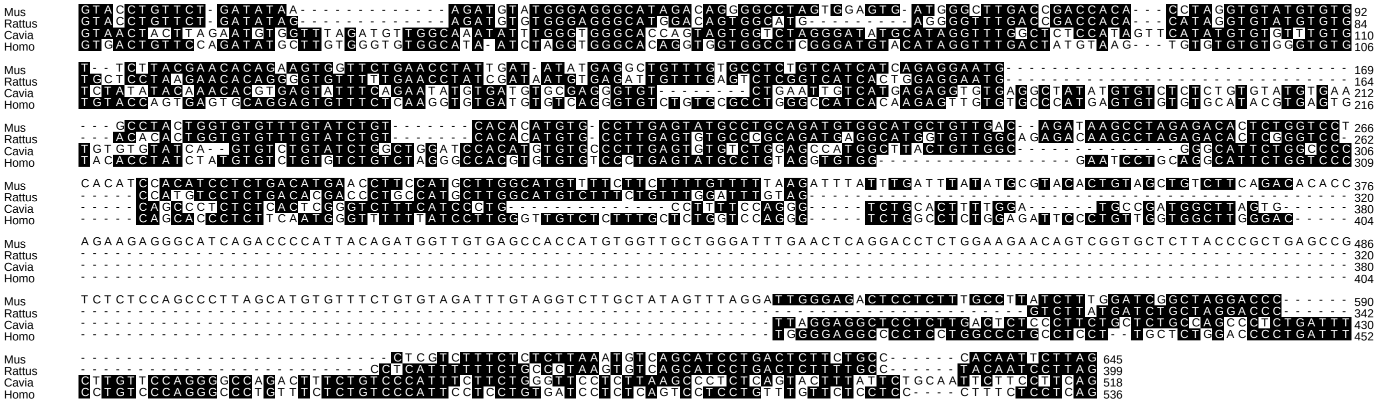
Mus
Rattus
Cavia
Homo

ENSMUSG00000003423 intron 5

Description: PIH1 domain-containing protein 1 (Pih1d1)
Intron number: 5
Mouse chromosome: 7
Upstream exon length: 82
Downstream exon length: 130
Mouse intron length: 321
Intron alignment length: 419
Total murinae branch length: 0.32665
K_score: 0.07299
Scaling factor: 0.74524

ENSMUSG00000003423 exon 5 (ORF 0)

```
Mus     AACAGTGATTTCTTGCGGGAACTGGTGGTCACCATCGCCAGGGAGGGCCTTGAGGACAAGTATGGCCTACAGCTAAATCC 80
Rattus  AACAGTGATTTCCTGAGGGAACTAGTGGTCACCATTGCCAGGGAGGGCCTTGAAGACAAGTATGGCCTACAACTAAACCC 80
Cavia   AACAGTGATTTCTTGCGGGAGCTCGTGATCACCATCGCCAGGGAGGGCCTTGAGGATAAGTACAACTTACAGCTGAATCC 80
Homo    AACAGCGATTTCTTGCGGGAGCTCGTGATCACCATCGCCAGGGAGGGCCTTGAGGACAAATACAACTTGCAGCTGAATCC 80

Mus     TG 82
Rattus  CG 82
Cavia   GG 82
Homo    GG 82
```

ENSMUSG00000003423 exon 6 (ORF 2)

```
Mus     AATGGCGCATGTTGAAGTACCGGTCTTTCCTGGGCTCCATCTCACAGCAGAACATCCGTTCTCAGCAGCGACCTCGGATC 80
Rattus  AATGGCGCATGCTGAAGTACCGGTCTTTCCTGGGCTCCATCTCACAGCAGAGCATCCGTTCCCAGCAGCGCCCTCGGATC 80
Cavia   AATGGCGCATGATGAAGAACCGGACTTTCCTGGGCTCCATTTCGCAGCAGAACATCCGCTCCAAGCAGCGTCCTCGGATC 80
Homo    AATGGCGCATGATGAAGAACCGGCCATTCATGGGCTCCATCTCGCAGCAGAACATCCGCTCGGAGCAGCGTCCTCGGATC 80

Mus     CAGGAGCTGGGGACCCTGGATGCCAGTGGCTCACTGGGAACCTGTCATGG 130
Rattus  CAGGAGCTGGGGACCCTGGATACCCATGACTCACTCGGAACCCGGCATGG 130
Cavia   CAGGAGCTGGGGAACCTACATGTGCCTGACTCCCGGGGCCCTGAGGGAAG 130
Homo    CAGGAGCTGGGGGACCTGTACACGCCCGCCCCCGGGAGAGCTGAGTCAGG 130
```

ENSMUSG00000003423_intron_5

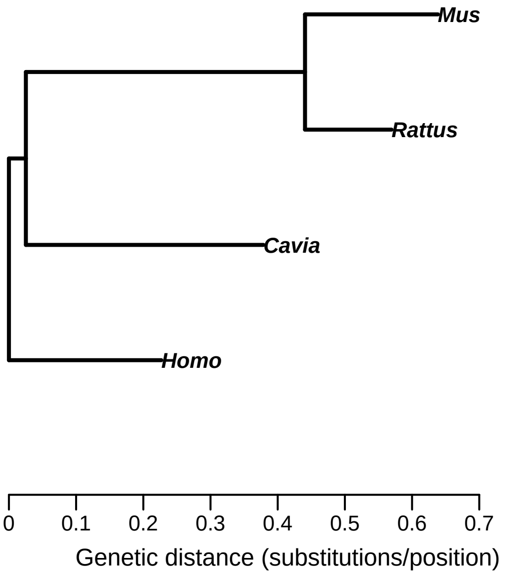

```
Mus     GTGAGGAACAGGA---------------------AGGAACAGGAATGGCTTGGGTGCTA-CACTGGGAGAGGTGAATCCAGTGTGTCGGGCATTTCCTTGACTAGCT--- 85
Rattus  GTGAGGAGCAAGA---------------------------------GGCTTGGGTGCCT-CACTTGGGAGAGGTGAATCAGTGTGTCAAGTGGTTCCTCCACTAGCT--- 73
Cavia   GTGAGAAGTGGGGCCTGGGCCTTCAGGCAGGAGTGGAGCCTGGCAAGGTTTAGGGGCCACCATTGGAAGACGA--GCTTGGTGTGGGCAGGGATTTTCTTACTTTCTGAA 108
Homo    GTGAGGGGCAGGTCCTGGGCCCTCGGGAAAGAGTGGAGCCAGGAGCGGTTTGGGGAACACCCCTAGGAGAGGAGGGGCTGGCTTGGGCAGGG---------TGCCCTGGA 101

Mus     ----------TTCAGGACGGGTGACGT-TTCTTAGAATGAGGGTGGAGCCAAG----------------AAGGTTCCAGGCCAGGAGCTTGCAGGGTGTG---------- 158
Rattus  ----------TTCAGGAGGGGTGAAGC-CTCTTAGAATAAGGGTGGAGCCAAG----------------ATGGTTCAAGGCCAAGAGCCTATAGCGTGTGCCTAGGGGAG 156
Cavia   GACTGGGTTACAGGGAAGGGATGGGTATCCCCAGAGTGAGGGGTGGTGCAAGGAGCCCGAATCCAGGTAGGGGACAGAGCCAGG-GCATCTAGAG------AAGTGATT 211
Homo    GTCTGTGCTACTAGGAGGGGGTGGAGT------------------------------CTGGACGCCCTGAGGGGGCGGGGCCAGGAACATCTCCAG------GGGTGGGT 175

Mus     ----TGAGTGTGTGTTTGTGTAACAGTAGAGCTAAGGTGGGTAGGGGCCGCTAGAGTCAAGCTT----GCTATGGTCAGCAGG--AGGTATCCAGGAAGGCGGCCCAGCC 258
Rattus  TTTCTTGGTGCATTCCCCAGGAAGGGTGGGGCTCAGGTAGGTGGGGCCCCTAGAATCAAGCTT----GCTATGGTGAG------------------------------- 231
Cavia   TTC---------CCACTTGGGAAGTGAGAGGCTATGTG-GGTGTGGCTTATTTAAGCCACGCCT-TGGGGCGTGGTCGGGGCT--GGTGTTCTCG-GAGAGGGCGGAGCC 307
Homo    TTTATTGGGGCATATCATGGGAGGGGCGTGACTAAGGT-GGGCGGGCTTCCTGGAGCCAAGCCTGCGGGGTGTGGTCAGACCCCAAGAGTCCTGGTGAGCCGGTGGAGCC 284

Mus     TCTGTGGACTTGGGAGCACTCCCT----------------GAG----GGTTCACCTCAGTGATCTTTGA-----CTCTGCACCTTCAG 321
Rattus  -----------GGGAGCACCACCT----------------GAGGTGAGCACTACCTTAGTGATCTTTGA-----CTCTGCACTGTCAG 287
Cavia   TCTAT------GGAAGCTCTCTAGACCCAACCACCTAAGAGTGAGAAGGACAGATCTTTGC-------------CTCTCTCACCTTAG 376
Homo    TATCC------CGGGAGGCTCTTGGCCCCCACA-------AAGGGAACCCTGACCTGTACGATGTTTGACGGCGCTCCTGACCCTCAG 359
```

ENSMUSG00000032470 intron 4

Description: Ras-related protein M-Ras Precursor (Mras)
Intron number: 4
Mouse chromosome: 9
Upstream exon length: 80
Downstream exon length: 100
Mouse intron length: 1228
Intron alignment length: 1670
Total murinae branch length: 0.19439
K_score: 0.08719
Scaling factor: 0.74803

ENSMUSG00000032470 exon 4 (ORF 0)

```
Mus     ATCCCATATATAGAGACCAGTGCCAAGGACCCGCCTCTCAACGTGGATAAAACCTTCCATGACCTAGTTAGAGTAATTAG 80
Rattus  ATCCCATATATAGAAACCAGTGCCAAGGACCCGCCTCTCAACGTGGACAAAACCTTCCATGACCTAGTTAGAGTAATTAG 80
Cavia   ATTCCTTACATAGAAACCAGTGCCAAGGACCCGCCTCTCAACGTGGACAAGGCCTTCCACGACCTCGTTAGAGTCATCAG 80
Homo    ATTCCGTACATAGAAACCAGTGCCAAGGACCCACCTCTCAATGTCGACAAAGCCTTCCATGACCTCGTTAGAGTAATTAG 80
```

ENSMUSG00000032470 exon 5 (ORF 1)

```
Mus     GCAACAGGTTCCAGAGAAAAACCAGAAGAAGAAAAAGAAGACAAAATGGCGAGGAGACAGGGCCACCGGCACTCACAAAC 80
Rattus  GCAACAGGTTCCAGAGAAAAACCAGAAGAAGAAAAAGAAGACAAAATGGCGAGGGGACAGGGCCACCGGCACTCACAAAC 80
Cavia   GCAACAGATTCCAGAAAAAAGCCAAAAGAAGAAGAAGAAAACCAAATGGCGAGGAGACCGTGCCACTGGCACCCACAAAC 80
Homo    GCAACAGATTCCGGAAAAAAGCCAGAAGAAGAAGAAGAAAACCAAATGGCGGGGAGACCGGGCCACAGGCACCCACAAAC 80

Mus     TGCAGTGTGTCATCTTGTGA 100
Rattus  TACAGTGTGTCATCTTGTGA 100
Cavia   TCCAGTGTGCGATACTG--- 97
Homo    TGCAATGTGTGATCTTGTGA 100
```

ENSMUSG00000032470_intron_4

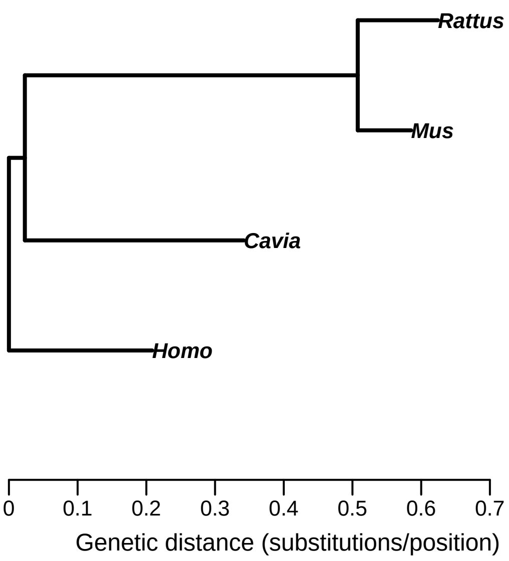

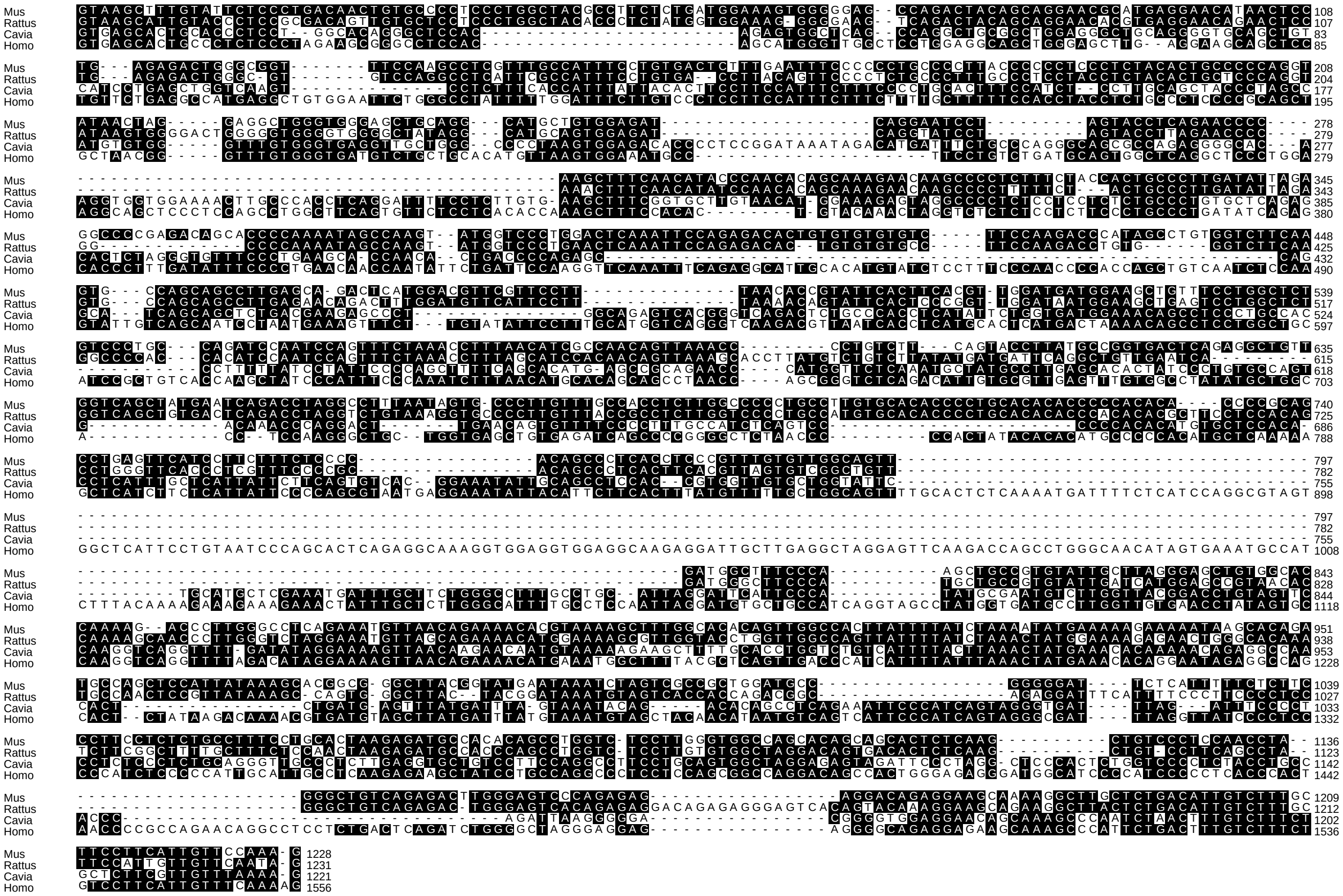

ENSMUSG00000027332 intron 6

Description: Isovaleryl-CoA dehydrogenase, mitochondrial Precursor (Ivd)
Intron number: 6
Mouse chromosome: 2
Upstream exon length: 137
Downstream exon length: 97
Mouse intron length: 1360
Intron alignment length: 1710
Total murinae branch length: 0.24560
K_score: 0.0546
Scaling factor: 0.74816

ENSMUSG00000027332 exon 6 (ORF 2)

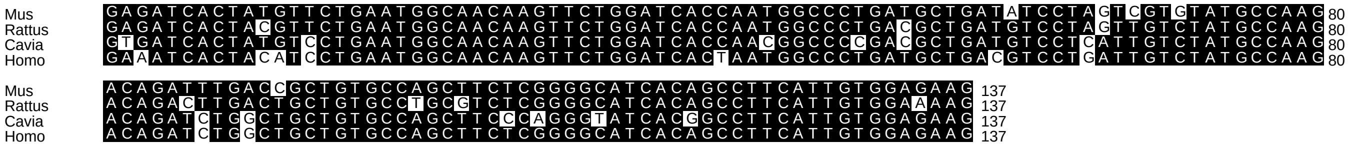

ENSMUSG00000027332 exon 7 (ORF 0)

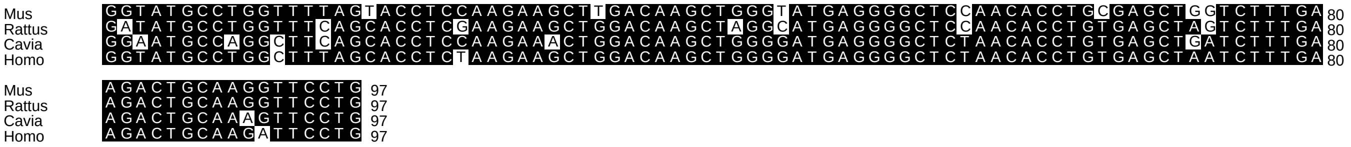

ENSMUSG00000027332_intron_6

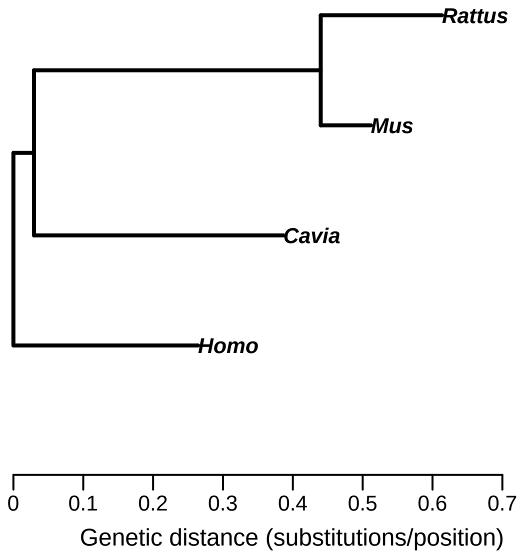

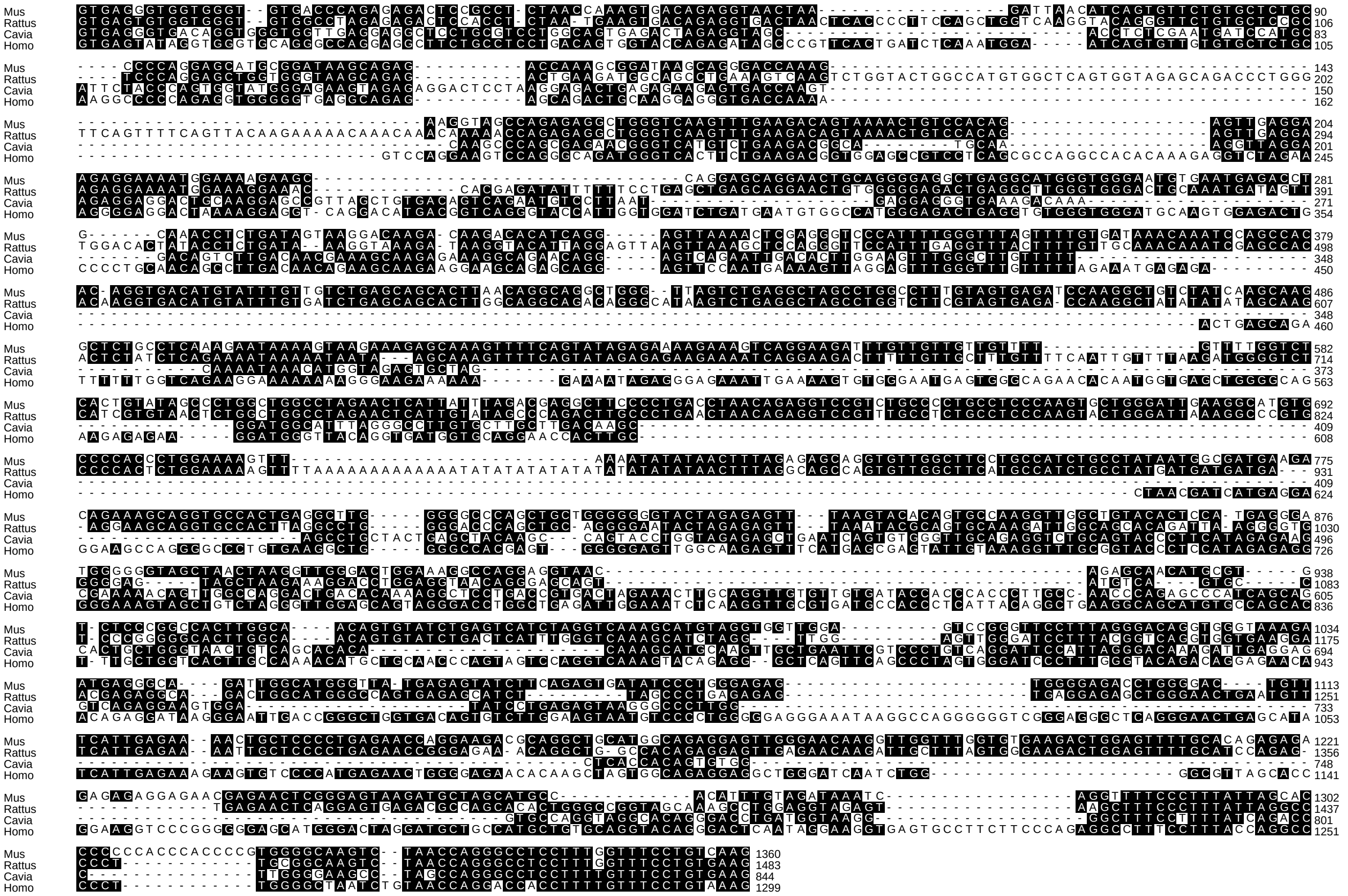
Mus
Rattus
Cavia
Homo

ENSMUSG00000042096 intron 5

Description: D-amino-acid oxidase (Dao)
Intron number: 5
Mouse chromosome: 5
Upstream exon length: 55
Downstream exon length: 102
Mouse intron length: 1301
Intron alignment length: 2199
Total murinae branch length: 0.21210
K_score: 0.0405
Scaling factor: 0.74934

ENSMUSG00000042096 exon 5 (ORF 1)

```
Mus     GTTAACTGAGAGGGGAGTGAAGCTTATCCATCGGAAGGTGGAGTCTCTCGAAGAG 55
Rattus  GTTAACTGAGAGGGGAGTGAAGTTCATCCATCGGAAGGTGGCATCTTTCGAAGAG 55
Cavia   GTTAACTGAGAGGGGAGTGAAATTCTTCCAGCGGAAGGTGGAGTCTTTGGAGGAG 55
Homo    GTTAACTGAGAGGGGAGTGAAGTTCTTCCAGCGGAAAGTGGAGTCTTTTGAGGAG 55
```

ENSMUSG00000042096 exon 6 (ORF 0)

```
Mus     ---GTGGCAAGAGGAGTGGATGTGATTATCAACTGCACCGGGGTGTGGGCCGGGGCCCTGCAAGCAGATGCCTCCCTGCA 77
Rattus  GTGGTGAGAGGAGGCGTGGATGTGATTATCAACTGCACCGGGGTGTGGGCCGGGGCTCTGCAAGCAGACGCCTCCCTGCA 80
Cavia   GTGGCCAGAGGAGGGGCGGATGTGATTATCAACTGCACGGGGGTGTGGGCTGGAGCACTACAGCCAGACCCACTGCTGCA 80
Homo    GTGGCAAGAGAAGGCGCAGACGTGATTGTCAACTGCACTGGGGTATGGGCTGGGGCGCTACAACGAGACCCCCTGCTGCA 80

Mus     GCCAGGCCGGGGCCAGATCATCCAG 102
Rattus  GCCAGGCCGGGGCCAGATCATCCAG 105
Cavia   GCCAGGCCGGGGCCAGATCATTAAG 105
Homo    GCCAGGCCGGGGGCAGATCATGAAG 105
```

ENSMUSG00000042096_intron_5

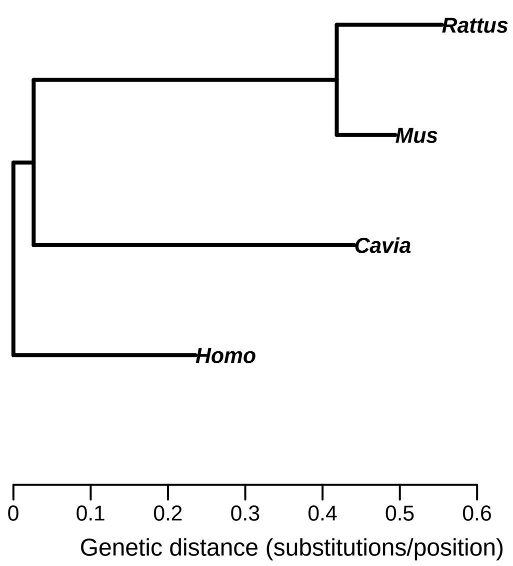

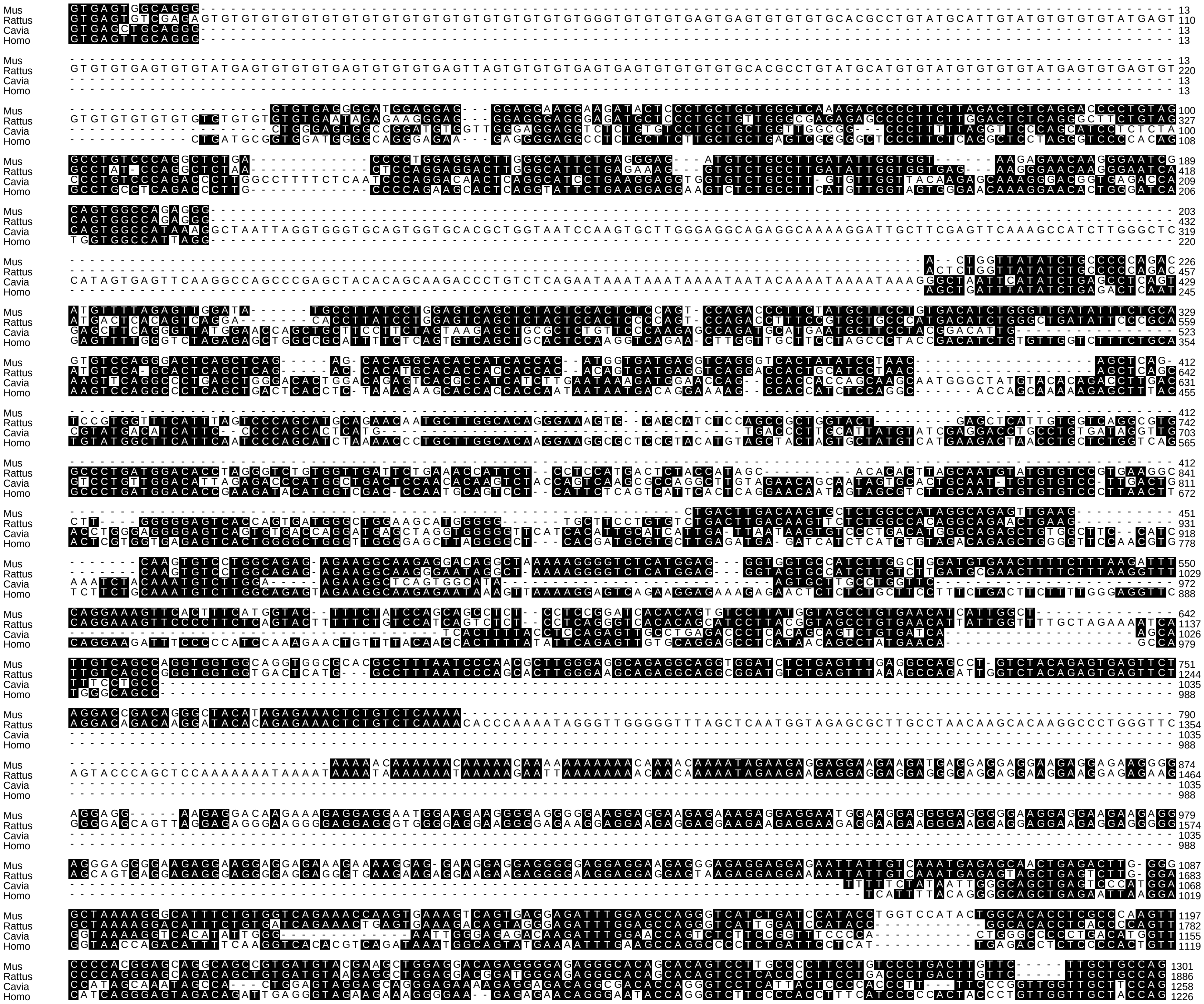

ENSMUSG00000020429 intron 3

Description: Insulin-like growth factor-binding protein 1 Precursor (Igfbp1)
Intron number: 3
Mouse chromosome: 11
Upstream exon length: 129
Downstream exon length: 132
Mouse intron length: 988
Intron alignment length: 1644
Total murinae branch length: 0.16837
K_score: 0.05517
Scaling factor: 0.74936

ENSMUSG00000020429 exon 3 (ORF 0)

```
Mus     GAGCCCTGCCAACGAGAACTCTATAAAGTGCTAGAGAGATTAGCTGCAGCCCAACAGAAAGCAGGAGATGAAATCTACAA 80
Rattus  GAGCCCTGCCAACGGGAACTCTATAAAGTGTTAGAGAGATTAGCTGCCGCTCAACAGAAAGCAGGAGATGAGATCTACAA 80
Cavia   CAGCCGTGCCAGCGAGAGCTCTACAGAGTGCTGGAGAGACTAGCCAATGGGCAACAGCAGGCCGGGGAGGAAATCTACAA 80
Homo    GAGCCCTGCCGAATAGAACTCTACAGAGTCGTAGAGAGTTTAGCCAAGGCACAGGAGACATCAGGAGAAGAAATTTCCAA 80

Mus     ATTTTATCTGCCAAACTGCAACAAGAATGGATTTTATCACAGCAAACAG 129
Rattus  ATTTTATCTGCCAAACTGCAACAAGAATGGATTTTATCACAGCAAACAG 129
Cavia   ATTTTATCTGCCTAACTGCAACAAGAATGGATTCTATCACAGCAAACAG 129
Homo    ATTTTACCTGCCAAACTGCAACAAGAATGGATTTTATCACAGCAGACAG 129
```

ENSMUSG00000020429 exon 4 (ORF 0)

```
Mus     TGTGAGACATCCCTGGATGGAGAAGCTGGACTCTGCTGGTGTGTCTACCCATGGAGTGGAAAGAAAATCCCTGGGTCTCT 80
Rattus  TGCGAGACATCTCTGGATGGAGAAGCTGGGCTCTGCTGGTGTGTCTACCCATGGAGTGGGAAGAAGATCCCTGGATCTCT 80
Cavia   TGCGAGACCTCCCTGGACGGCGAGGCGGGGCTGTGCTGGTGCGTCTACCCCTGGAGTGGGAAGAGGATCCCAGGGTCTCC 80
Homo    TGTGAGACATCCATGGATGGAGAGGCGGGACTCTGCTGGTGCGTCTACCCTTGGAATGGGAAGAGGATCCCTGGGTCTCC 80

Mus     GGAGACTAGAGGGGACCCCAACTGCCACCAGTATTTTAATGTACATAACTGA 132
Rattus  GGAGACCAGAGGGGACCCCAACTGCCACCAGTATTTTAATGTGCAAAACTGA 132
Cavia   AGAGACCAGAGGGGACCCCAACTGCCAGCTGTATTTTAACTTACAAAAC--- 129
Homo    AGAGATCAGGGGAGACCCCAACTGCCAGATATATTTTAATGTACAAAACTGA 132
```

ENSMUSG00000020429_intron_3

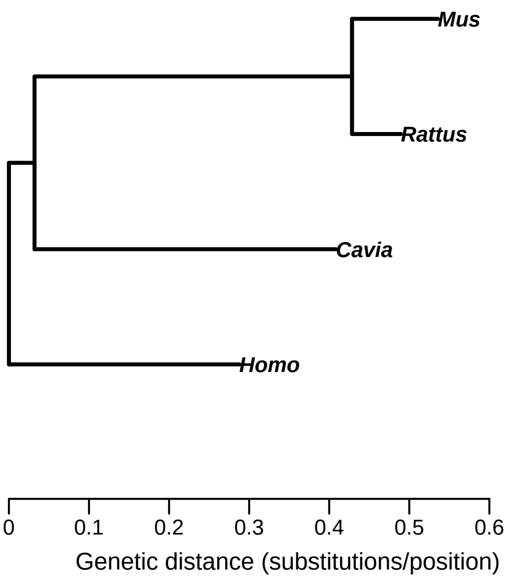

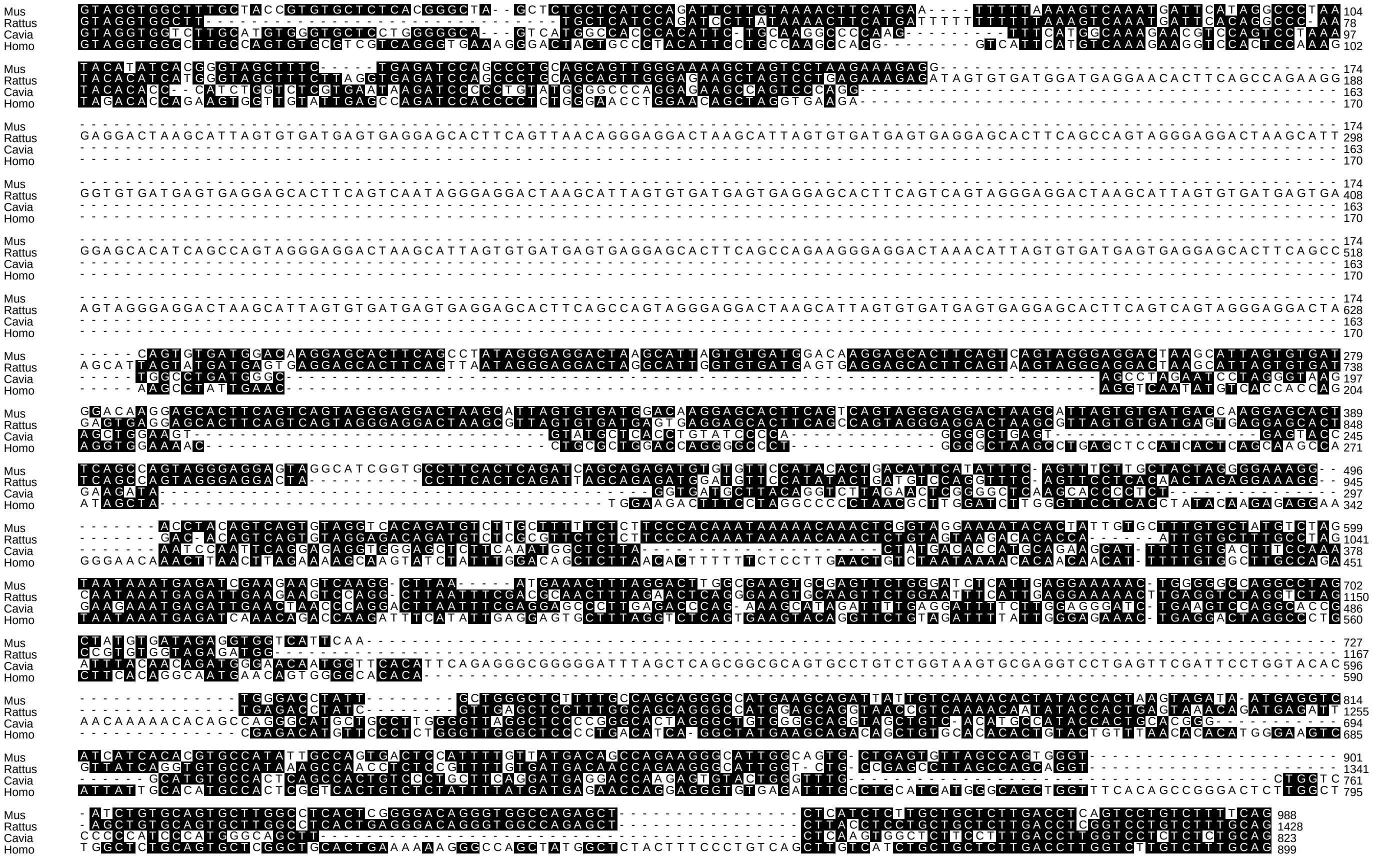
Mus
Rattus
Cavia
Homo

ENSMUSG00000037622 intron 14

Description: WD and tetratricopeptide repeats protein 1 (Wdtc1)
Intron number: 14
Mouse chromosome: 4
Upstream exon length: 193
Downstream exon length: 198
Mouse intron length: 812
Intron alignment length: 1241
Total murinae branch length: 0.24149
K_score: 0.06426
Scaling factor: 0.74946

ENSMUSG00000037622 exon 14 (ORF 1)

```
Mus     CGGCTCCTTCTTCATCTGGGAAAAGGAGACCACCAACCTCGTCCGAGTGCTGCAGGGGGATGAGTCCATTGTCAACTGCT 80
Rattus  TGGCTCCTTCTTCATCTGGGAAAAGGAGACCACCAACCTAGTCCGAGTGCTCCAGGGAGATGAATCCATTGTCAACTGCT 80
Cavia   TGGCTCCTTCTTCATCTGGGAAAAAGAAACCACCAACCTTGTCCGCGTGCTCCAGGGGGATGAGTCCATTGTCAACTGCC 80
Homo    TGGCTCCTTCTTCATCTGGGAAAAGGAGACCACCAACCTGGTCCGTGTGCTCCAAGGGGATGAGTCCATTGTCAACTGCC 80

Mus     TGCAGCCCCATCCCAGCTACTGCTTCCTGGCCACCAGTGGCATCGACCCTGTTGTGCGACTGTGGAACCCACGACCAGAG 160
Rattus  TGCAGCCACACCCCAGCTACTGCTTCCTGGCTACCAGTGGCATCGACCCTGTGGTGCGATTGTGGAACCCACGACCAGAG 160
Cavia   TGCAGCCACATCCCAGCTACTGCTTCCTGGCCACCAGTGGCATTGACCCCGTTGTGCGACTCTGGAACCCCCGACCAGAG 160
Homo    TGCAGCCACACCCCAGCTACTGCTTCCTGGCCACCAGTGGCATCGATCCTGTTGTGCGGCTCTGGAACCCCCGACCAGAG 160
```

ENSMUSG00000037622 exon 15 (ORF 0)

```
Mus     AGTGAAGACCTCACAGGCCGAGTTGTGGAAGATATGGAGGGTGCATCTCAGGCCAACCAGCGGCGCATGAATGCCAACCC 80
Rattus  AGTGAAGACCTCACGGGCCGAGTCGTGGAGGACATGGAGGGTGCCTCTCAGGCCAACCAGCGGCGCATGAATGCCAATCC 80
Cavia   AGTGAAGACCTCACAGGCCGAGTTGTGGAGGACATGGAGGGCGCCTCTCAGGCCAACCAAAGGCGCATGAATGCAGACCC 80
Homo    AGTGAAGACCTCACAGGCCGAGTCGTGGAAGATATGGAGGGTGCTTCACAGGCCAACCAGCGGCGCATGAATGCAGACCC 80

Mus     CCTGGAGGCGATGCTGCTGGACATGGGCTATCGGATCACAGGCCTGAGCAGCGGGGGCGCCGGGGCCTCTGATGACGAGG 160
Rattus  CCTGGAGGCGATGCTGCTGGACATGGGCTACCGGATCACAGGCCTGAGCAGTGGGGGCGCTGGGGCCTCCGATGACGAGG 160
Cavia   CTTGGAGGTGATGCTGCTCAACATGGGCTACCGGATCACAGGCCTGAGCAGTGGGGGCGCTGGGGCCTCCGATGATGAGG 160
Homo    GTTGGAGGTGATGCTGCTCAACATGGGCTACCGGATCACGGGCCTGAGCAGTGGGGGTGCCGGGGCCTCTGATGATGAGG 160
```

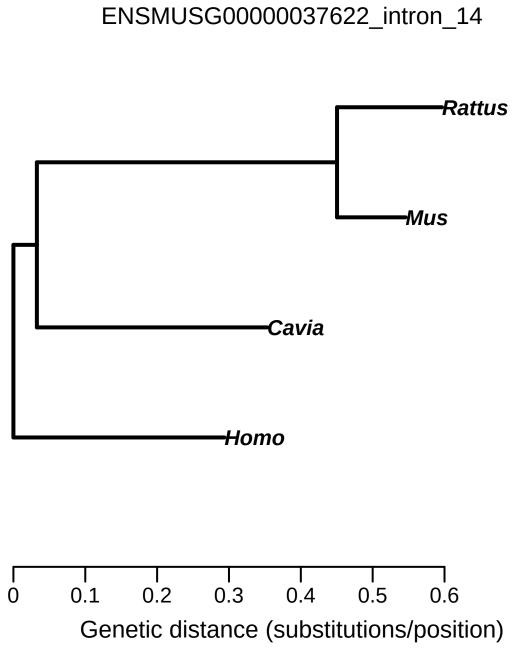

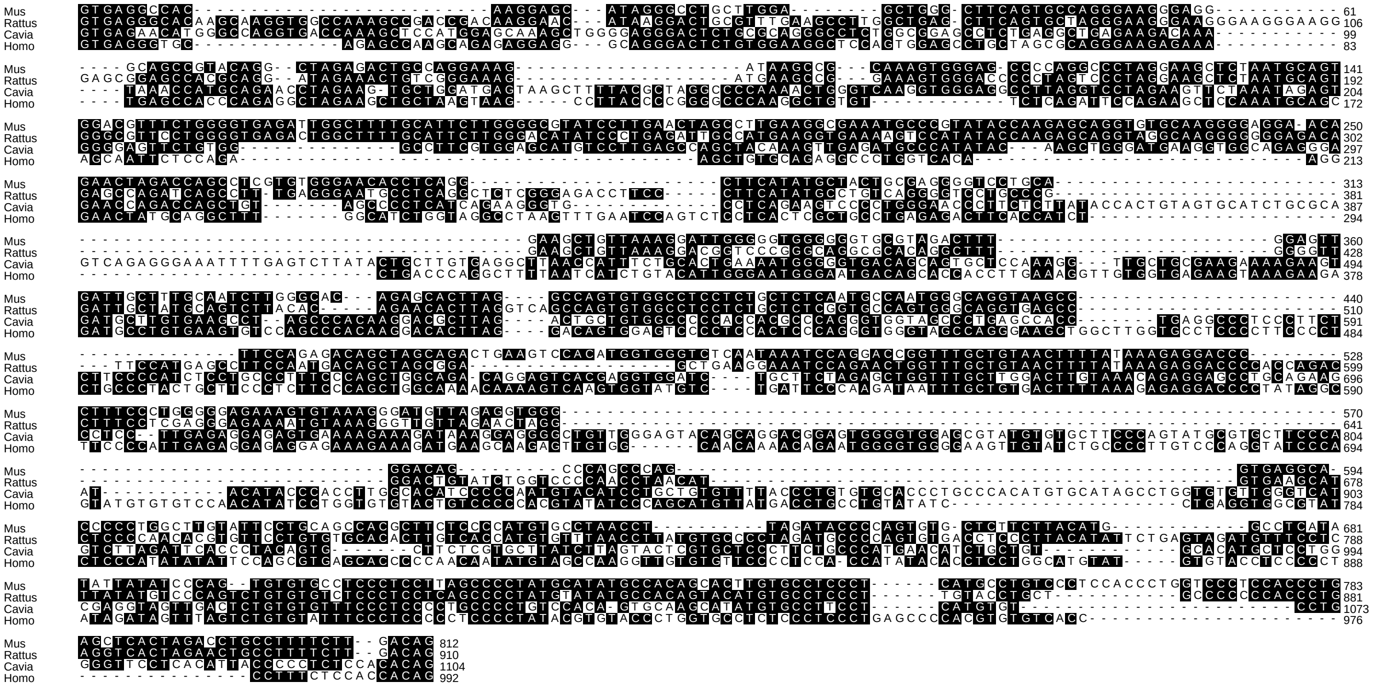

ENSMUSG00000075701 intron 1

Description: Selenoprotein S (H47)
Intron number: 1
Mouse chromosome: 7
Upstream exon length: 76
Downstream exon length: 135
Mouse intron length: 542
Intron alignment length: 797
Total murinae branch length: 0.12807
K_score: 0.06245
Scaling factor: 0.75055

ENSMUSG00000075701 exon 1 (ORF 0)

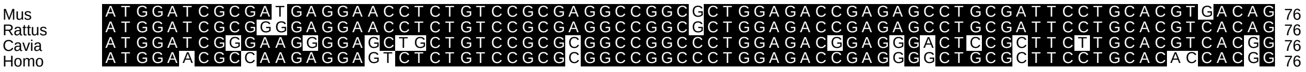

ENSMUSG00000075701 exon 2 (ORF 2)

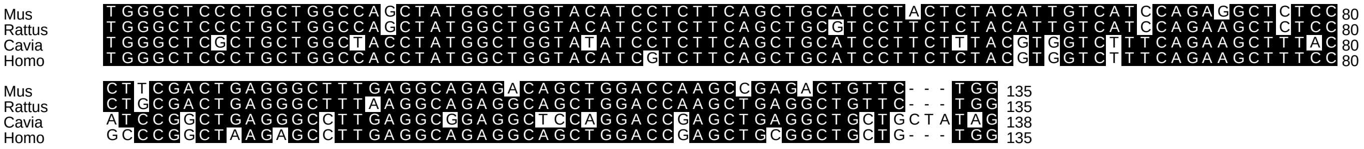

ENSMUSG00000075701_intron_1

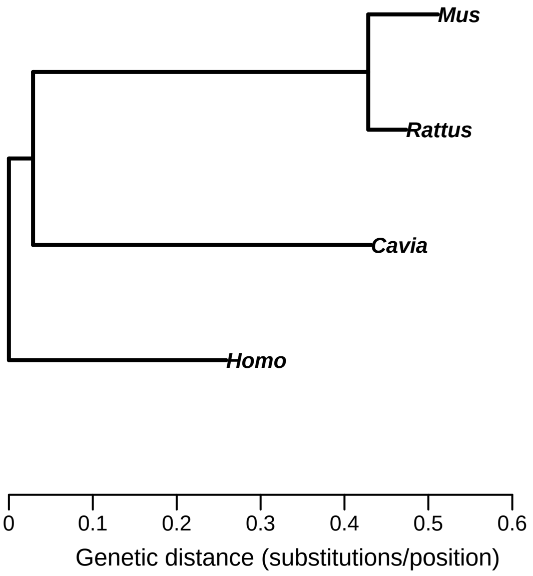

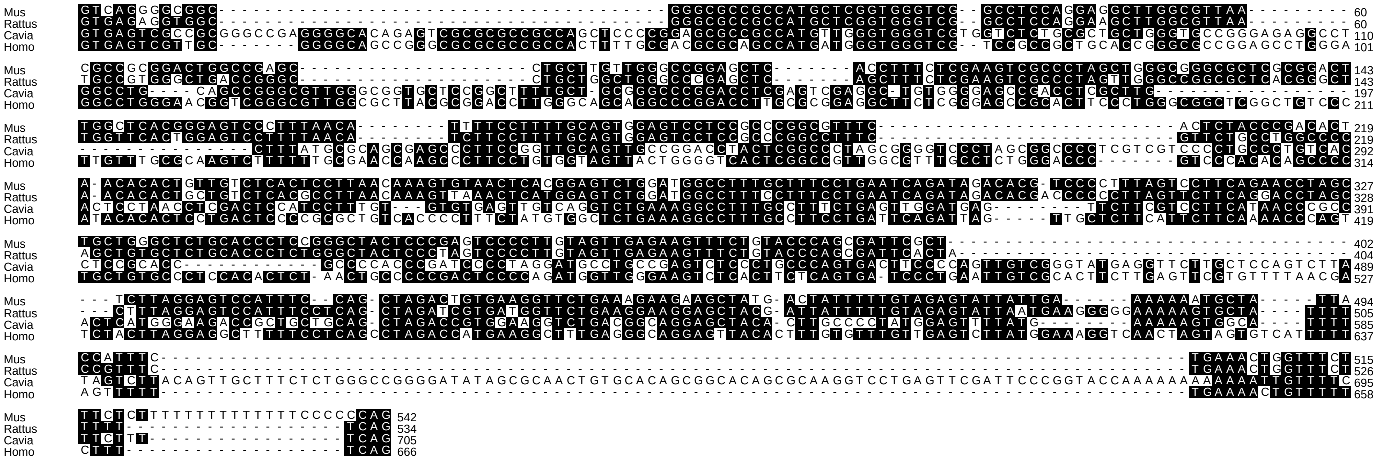

ENSMUSG00000027356 intron 10

Description: Fermitin family homolog 1 (Fermt1)
Intron number: 10
Mouse chromosome: 2
Upstream exon length: 107
Downstream exon length: 222
Mouse intron length: 1341
Intron alignment length: 3020
Total murinae branch length: 0.18115
K_score: 0.07177
Scaling factor: 0.75191

ENSMUSG00000027356 exon 10 (ORF 2)

```
Mus     GCTGCGAAATTGTGCCAGATGTGAATGTATCGGGGAGAAAATTTGGAATCAAGTTACTAATCCCCGTTGCTGATGGTATG 80
Rattus  GCTGTGAAATTGTGCCAGATGTGAATGTAGCAGGGAGAAAATTTGGAATCAAGTTACTAATCCCTGTTGCTGATGGAATG 80
Cavia   GCTGTGAAATTGTACCAGATGTGAATGTAGCAGGGAGAAAATTTGGAATCAAGTTACTAATCCCTGTCGCGGATGGTATG 80
Homo    GCTGCGAAGTTGTGCCCGATGTAAATGTAGCAGGAAGAAAATTTGGAATCAAGTTACTAATCCCTGTTGCCGATGGTATG 80

Mus     AATGAAGTGTACTTGAGATGTGACCAT 107
Rattus  AATGAAGTGTATTTGAGATGTGACCAT 107
Cavia   AACGAAGTGTACCTGAGATGTGACAAT 107
Homo    AATGAAATGTATTTGAGATGTGACCAT 107
```

ENSMUSG00000027356 exon 11 (ORF 0)

```
Mus     GAGGATCAATATGCCCGATGGATGGCTGCCTGCATCTTGGCATCGAAGGGTAAAACTATGGCAGACAGCTCCTATCAGCC 80
Rattus  GAGGATCAATATGCCCGGTGGATGGCTGCGTGCATCTTGGCCTCGAAGGGTAAAACCATGGCAGACAGCTCCTACCAGCC 80
Cavia   GAGAATCAGTATGCCCGGTGGATGGCTGCCTGTGTCTTGGCATCCAAGGGCAAAACCATGGCAGACAGCTCCTACCAGCC 80
Homo    GAGAATCAATACGCCCAATGGATGGCTGCCTGCATGTTGGCATCGAAGGGCAAAACCATGGCAGACAGCTCCTACCAGCC 80

Mus     AGAGGTCATCAGCATCCTTTCGTTTCTCAAGATGAAAAACCGGAACTCATCACCTCTGGTGGCTTCCAGTCTTGAAAACA 160
Rattus  AGAGGTCGTCAGCATCCTTTCATTTCTCAAGATGAAAAACCGGAACTCATCACCTCTGGTGGCTTCCAGTCTGGAAAGCA 160
Cavia   CGAGGTCCTCAACATCCTCTCATTTCTGAGGATGAAGAACCGCAACTCGTCGTCTGACGTGGCTTCCAGTCCCGAAAGCA 160
Homo    AGAGGTCCTCAACATCCTTTCATTTCTGAGGATGAAAAACAGGAACTCTGCATCTCAGGTGGCTTCCAGTCTCGAAAACA 160
```

ENSMUSG00000027356_intron_10

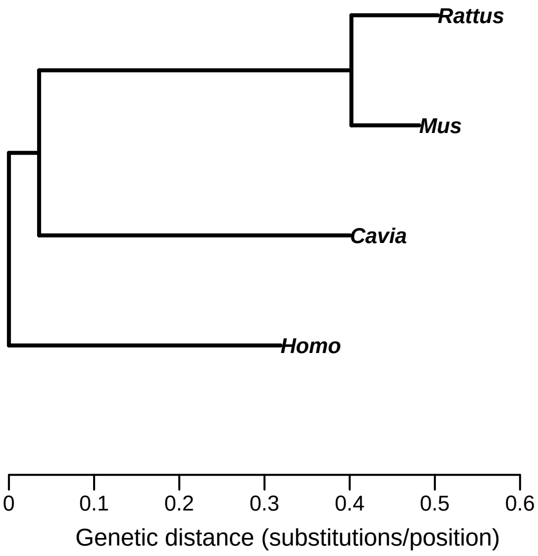

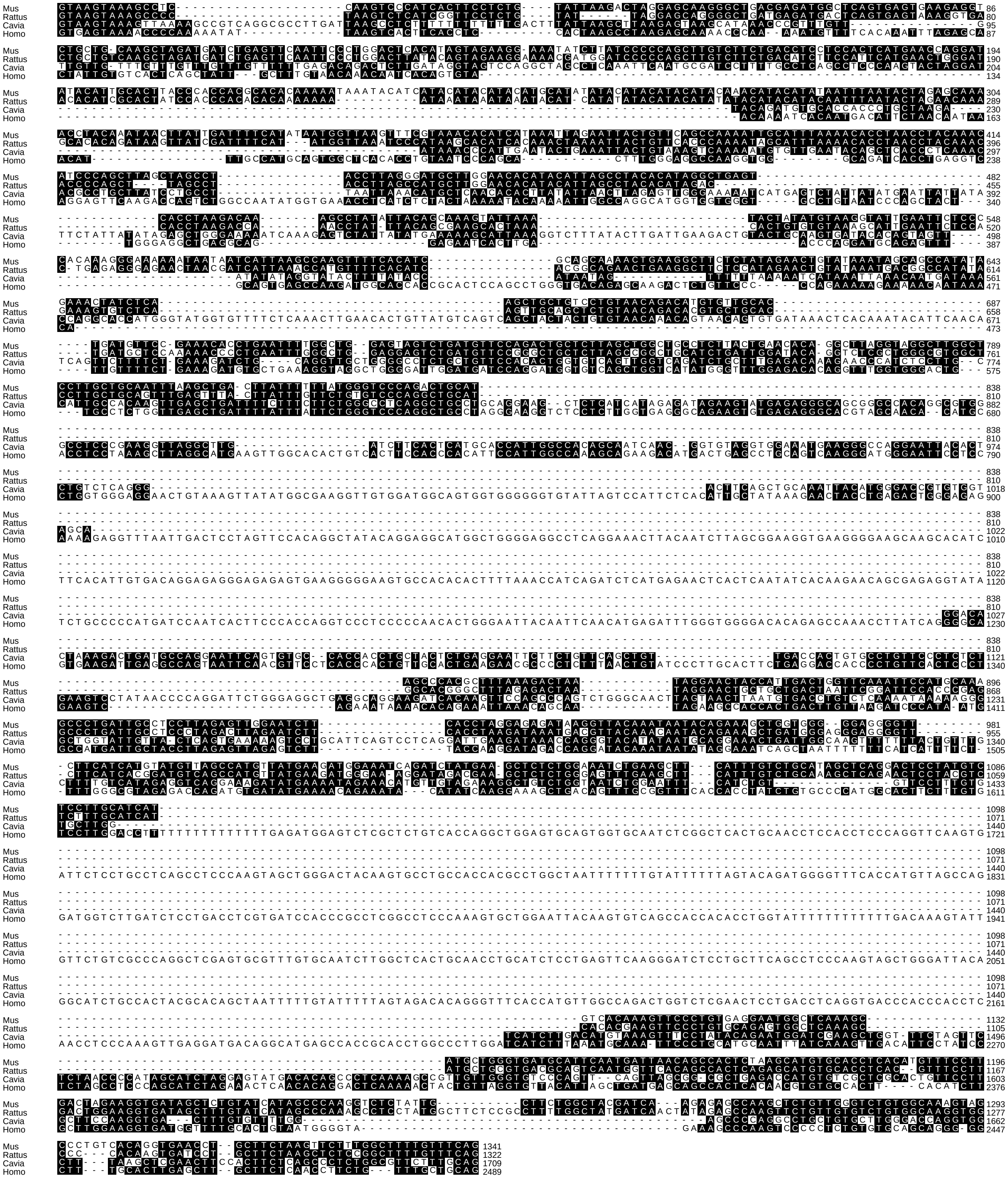
Mus
Rattus
Cavia
Homo

ENSMUSG00000015970 intron 6

Description: Choline dehydrogenase, mitochondrial Precursor (Chdh)
Intron number: 6
Mouse chromosome: 14
Upstream exon length: 103
Downstream exon length: 419
Mouse intron length: 559
Intron alignment length: 762
Total murinae branch length: 0.18254
K_score: 0.08378
Scaling factor: 0.75267

ENSMUSG00000015970 exon 6 (ORF 0)

```
Mus     GTGCACGTGGGAACCATGAGGGCCACAAGTGTGGGCTGGCTGAAACTGAGAAGTGCCAACCCCCGGGACCACCCTGTGAT 80
Rattus  GTACATGTGGGAACCATGAGGGCCACAAGTGTGGGCTGGCTGAAACTGAGAAGCACCAACCCTCAGGACCACCCAATGAT 80
Cavia   GTGCATGTGGGGCCCATGCGGGGCACGAGTGTGGGCTGGCTCAAACTGAGAAGTGCCGACCCCCAGGAGCACCCTGTGAT 80
Homo    GTACATGTGGGGCCCATGCGGGGCACGAGTGTGGGCTGGCTCAAACTGAGAAGTGCCAATCCCCAAGACCACCCTGTGAT 80

Mus     CCATCCCAACTACTTGTCAACAG 103
Rattus  CAATCCCAACTACCTGTCAACAG 103
Cavia   CCAACCCAACTACCTGTCAACAG 103
Homo    CCAGCCCAACTACTTGTCAACAG 103
```

ENSMUSG00000015970 exon 7 (ORF 2)

```
Mus     AAACCGATGTCGAGGACTTCCGTCAGTGTGTGAGGCTGTCACGAGAAATTTTTGCCCAGGAAGCCTTGGCTCCATTTCGG 80
Rattus  AAACCGATGTCGAGGACTTCCGTCAGTGTGTGAAGCTGACACGGGAAATTTTTGCACAGGAAGCCTTCGCTCCCTTTCGG 80
Cavia   AGGCCGATATCCAGGATTTCCGTCAGTGCGTGAAGCTGTCCAGAGAAATCTTTGCCCAGGAAGCCCTGGCTCCCTTCCGG 80
Homo    AAACTGATATTGAGGATTTCCGTCTGTGTGTGAAGCTCACCAGAGAAATTTTTGCACAGGAAGCCCTGGCTCCGTTCCGA 80

Mus     GGAAAAGAGCTGCAGCCCGGAAGCCACGTCCAGTCAGACAAAGAGATAGATGCCTTTGTGCGGGCAAAAGCAGACAGTGC 160
Rattus  GGCAAAGAGCTGCAGCCGGGAAGCCATGTCCAGTCAGACAAAGAGATAGATGCCTTTGTGCGGGCAAAAGCAGACAGTGC 160
Cavia   GGCAAGGAGCTGCAGCCAGGAAGCCACGTGCAGTCAGATAAGGAGATAGACGCCTTTGTGCGGGCAAAAGCTGACAGCGC 160
Homo    GGGAAAGAGCTCCAGCCAGGAAGCCACATTCAGTCAGATAAAGAGATAGATGCCTTTGTGCGGGCAAAAGCCGACAGCGC 160
```

ENSMUSG00000015970_intron_6

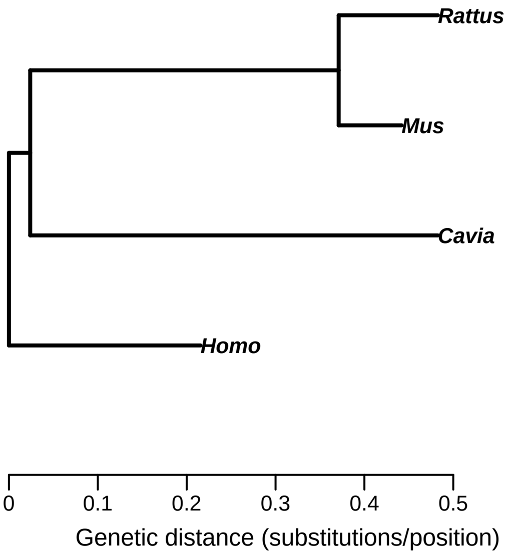

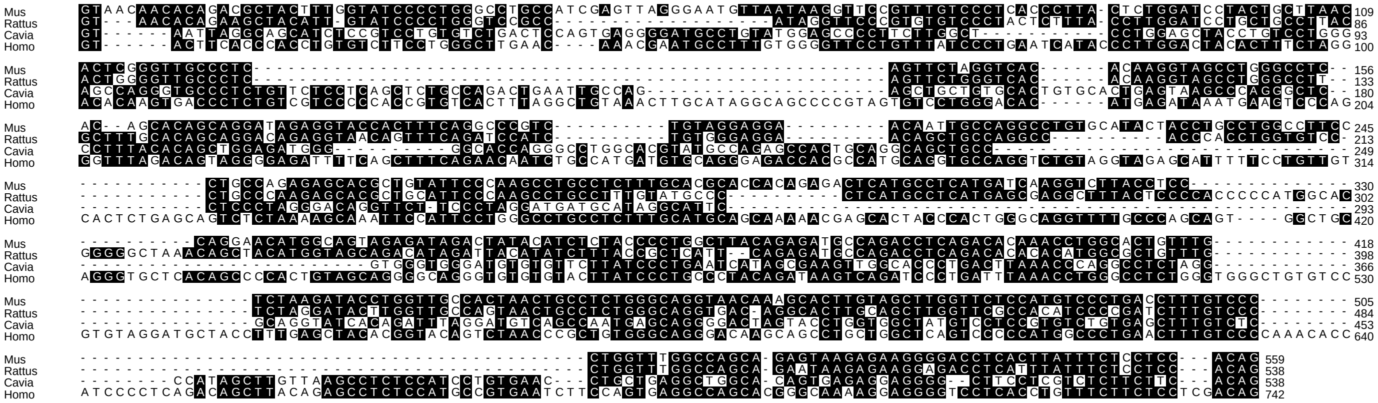
Mus
Rattus
Cavia
Homo

ENSMUSG00000026843 intron 4

Description: far upstream element (Fubp3)
Intron number: 4
Mouse chromosome: 2
Upstream exon length: 50
Downstream exon length: 72
Mouse intron length: 357
Intron alignment length: 474
Total murinae branch length: 0.25672
K_score: 0.02864
Scaling factor: 0.75417

ENSMUSG00000026843 exon 4 (ORF 1)

```
Mus     GGCAGTAATAACAGAAGAATTCAAAGTGCCTGATAAAATGGTTGGATTTA 50
Rattus  GGCAGTAATAACAGAAGAATTCAAGGTGCCTGATAAAATGGTTGGATTTA 50
Cavia   GGCGGTCATAACGGAAGAATTCAAAGTGCCGGATAAAATGGTTGGGTTCA 50
Homo    GACGGTAATAACGGAAGAATTCAAAGTGCCTGACAAAATGGTTGGATTTA 50
```

ENSMUSG00000026843 exon 5 (ORF 2)

```
Mus     TTATTGGCAGGGGAGGTGAGCAGATTTCACGAATTCAAGCAGAATCTGGGTGCAAAATTCAGATTGCCTCAG 72
Rattus  TTATTGGCAGGGGAGGTGAGCAGATTTCACGAATTCAAGCAGAATCTGGGTGCAAAATCCAGATTGCCTCAG 72
Cavia   TTATTGGCAGGGGAGGTGAGCAGATTTCACGGATTCAAGCAGAATCTGGTTGCAAAATTCAAATTGCTTCAG 72
Homo    TTATCGGCAGGGGAGGTGAGCAGATTTCACGGATTCAAGCAGAATCTGGTTGCAAAATTCAGATTGCTTCAG 72
```

ENSMUSG00000026843_intron_4

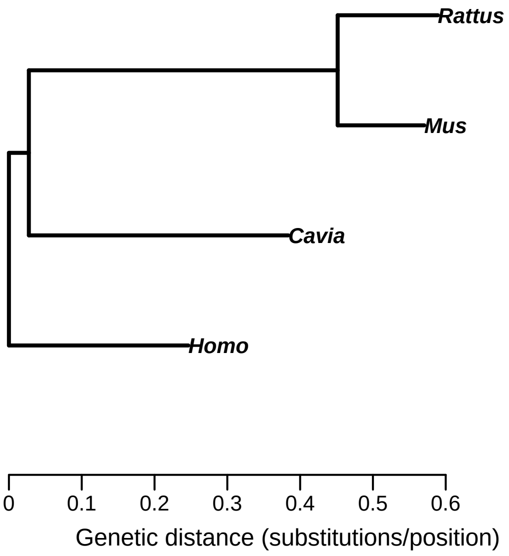

```
Mus     GTAAGTATTGC---------CTTTTTCCTTAACTCCTGTCTGT----------------------------------------TCTTGTCTGTTAGACTCTCTTATGAGC 61
Rattus  GTAAGTATCTGAATTG----CTTTGTGCTTAACTCTTGTCTGTTCTGTTTACCTTTTAAT-----------------------TCTTGTCTGTTCTATTCTCTTATGAGC 83
Cavia   GTAAGTATCTGCCTTACCCTCACTTTCCCTCGCTGCTGTCTG-----------------------------------------TCCTGTTTGCTCTGATCTCTCTCACGT 69
Homo    GTAAGTATCCATGTTGTCTACTTTTTCCCTGATTCCTGTCTCTTCTTTTTCTCTCTTTTTTTCTGAGCTGCTTTGCCAGGATGTTCTTTTTGTTTTAATCTCTCTTGTGA 110

Mus     ATGTC--------------ACCTGTGGTAGCCTGCTAGGGGGACTGTTGCAGGC------------------ATCGTGGTCAGTATCATCTGAACATCAGAGTACTTGG- 138
Rattus  GTGTC--------------ACCTGTGGTAGCATGCT-GGGGCAGTGCTGCAGGC------------------ATTGCAGT-AGCTGCGTCTGAACGGCAGGGCATCTGG- 158
Cavia   AAGCCTTAGCCATAGCCATAGCCATAGTCGGAAACT--------TCTCGCATGCATTTGCACAGCCCTG---AGTGCTGCAGGTCTC-TCTGGCCACAGGAGAGCCCTGA 167
Homo    GTATC--------------ACCTGTAGTAGAATGCT--------TTGCACATGCCATTCCCCACAGCTCTGAAATGCTGCAGGTCTCTTCAGGCTGAGGGAGAAACCAGA 198

Mus     -----------------------CCACCCCTAGCTATGCTTGCCAAGGCCGAAGTCGTTACCTGCTG-----TGTGTCACATGCATCGTCTGTTGCTGTGACGTCCATGA 220
Rattus  ------------------------CCACCCTAGCTATTCTTGACGCAGCTGAAGTAGTTACCTGCTG-----TGTGTCCCATGCAGCTTCTGTTGCTGTGGTGTCTGTGG 239
Cavia   GTGCAGGGCAAGGA-------TGCGCACCCCATCCTTTCCTTCCTC------GCTGGCCTCTGTCTGTCCACTGTGTTTCACGGGT--------------GCCTGTGGGG 250
Homo    GCACTGGACTGTGGATGTCCCTCCCTTCCCCACCTCTCCCCACCTCAGCTCAGATCCCCTCTGCCTGTAGGCTGTTTCACAAGGGCTTTCTGCTGCCATCATGCCCAAAG 308

Mus     GCTGAGA-GGACTCCCCAGAGG-----TTGGCTTATAGTGAGCTGTGAGCAGAGTGTAAATGTTTGCTGAGTTAATTAAGCTATCCATGTGTGCTTATGTGTGCAAAGGG 324
Rattus  GCTGACACGGGCTCCCCAGAGG-----ACGGCTTAGAGTGAGCTGTGAGC-GAGAGTGAATGTTTGCTGAGTTAGTTAAGCTA-----------GAGTGTGGAAAGGAGG 332
Cavia   AGTGCGTGTGGGCTTGCAGAGACCAGCTTGACTTGTTGTGAGCTGCGAGCCATGTGT-AATGTTTGCTGAATTCATTAAGCTATATGTGCGTGGTTTAGTGTAAGCCGGA 359
Homo    AGTGGAGATGGGCTCACGGGGGCCAACTCGATGTGTAGT--ATTGTGAGCCAAGTGTCACAGTTTGTGGAATTAATTCAGTCATTCCTGCGTGGTGAAATATGGAATAGG 416

Mus     GG-GCACCTTCTGACCCTGCTCGTTTCATTGCAG 357
Rattus  AG-GCATGGTCTGACCCTGCTCATTTCATTGCAG 365
Cavia   G--CTGTGTTCTGATCCTGTTCAATTTGTTTCAG 391
Homo    GGCGTGTATTCTGACCCTGTTCAATTTGTTTCAG 450
```

ENSMUSG00000037995 intron 5

Description: Protein turtle homolog A Precursor (Igsf9)
Intron number: 5
Mouse chromosome: 1
Upstream exon length: 118
Downstream exon length: 142
Mouse intron length: 842
Intron alignment length: 1651
Total murinae branch length: 0.24279
K_score: 0.0898
Scaling factor: 0.75533

ENSMUSG00000037995 exon 5 (ORF 0)

```
Mus     GTGCAGAATGGAACACTGTGGATCCGTCGGGTGGAGCGAGGCAGCGCTGGAGACTACACCTGCCAAGCCTCCAGCTCCGA 80
Rattus  GTGCGGAATGGAACACTGTGGATCCGTCGGGTGGAGCGAGGCAGCGCTGGAGACTACACCTGCCAAGCCTCCAGCACTGA 80
Cavia   GTGCAGAACGGGACCTTGTGGATCCGGCGGGTGCAACGAGGTAGCTCTGGAGTCTACACCTGCCAAGCCTCCAACACTGA 80
Homo    GTGCAGAACGGGACGCTGCGGATCCGCCGGGTAGAGCGAGGCAGCTCTGGGGTCTACACCTGCCAAGCCTCCAGCACTGA 80

Mus     GGGCAGCATCACCCACGCCACCCAGCTGTTGGTGCTAG 118
Rattus  GGGCAGCGTCACCCACACTACTCAGCTGCTGGTGCTAG 118
Cavia   GGGCAGCGCCACCCATGCCACTCAGCTGCTGGTGCTAG 118
Homo    GGGCAGCGCCACCCACGCCACCCAGCTGCTAGTGCTAG 118
```

ENSMUSG00000037995 exon 6 (ORF 2)

```
Mus     GACCCCCTGTCATTGTGGTGCCCCCCAGCAACAGTACAGTCAACTCCTCTCAGGATGTTTCCTTGGCCTGCCGGGCTGAG 80
Rattus  GACCCCCTGTCATTGTGGTGCCCCCCAACAACAACACAGTCAATGCCTCCCAGGATGTCTCTTTGGCTTGCCGGGCTGAG 80
Cavia   GACCCCCTATCATTGTGGTGCCCCCTAAGAACAGCACAGTCAATGCATCCCAGGATGTTTCCTTGGCCTGTCGGGCTGAG 80
Homo    GACCCCCAGTCATCGTGGTGCCCCCCAAGAACAGCACAGTCAATGCCTCCCAGGATGTTTCATTGGCCTGCCATGCTGAG 80

Mus     GCATACCCTGCTAACCTCACCTACAGCTGGTTCCAGGATGGTGTCAATGTCTTCCATATCAG 142
Rattus  GCATACCCTGCAAACCTCACCTATAGCTGGTTCCAGGATCGTATCAATGTCTTCCACATCAG 142
Cavia   GCGTACCCCACTAACCTCACCTACAGCTGGTTCCAGGATGGGATCAATGTCTTCCACATTAG 142
Homo    GCATACCCTGCTAACCTCACCTACAGCTGGTTCCAGGACAACATCAATGTCTTCCACATTAG 142
```

ENSMUSG00000037995_intron_5

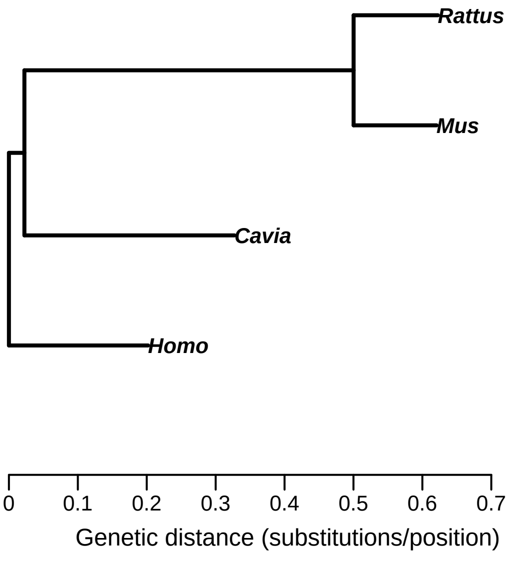

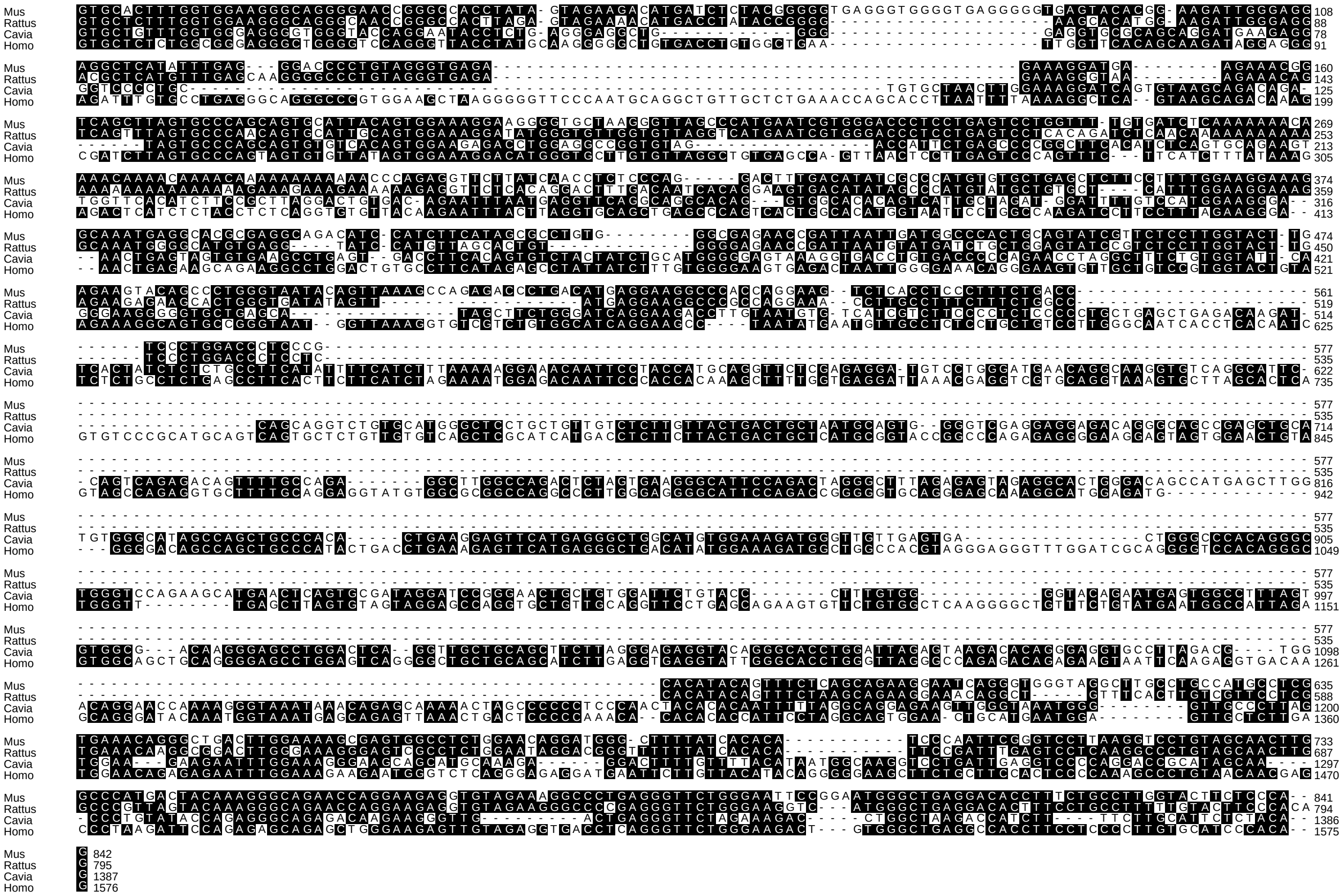

ENSMUSG00000037820 intron 7

Description: Protein-glutamine gamma-glutamyltransferase 2 (Tgm2)
Intron number: 7
Mouse chromosome: 2
Upstream exon length: 136
Downstream exon length: 104
Mouse intron length: 580
Intron alignment length: 1317
Total murinae branch length: 0.15253
K_score: 0.07437
Scaling factor: 0.75616

ENSMUSG00000037820 exon 7 (ORF 2)

```
Mus     TGCTGCGGTGCCTCGGCATCCCTACCAGAGTGGTGACCAACTACAACTCCGCCCACGACCAGAACAGCAACCTGCTCATC 80
Rattus  TGCTGCGGTGCCTTGGCATCCCTACCAGAGTGGTGACCAACTACAACTCCGCCCACGACCAGAACAGCAACCTGCTCATC 80
Cavia   TGCTGCGGTGCCTTGGCATCCCCACCCGAGTCGTGACCAACTTTAACTCAGCCCACGACCAGAACAGCAACCTGCTCATC 80
Homo    TGCTGAGGTGCCTGGGCATCCCTACCCGCGTCGTGACCAACTACAACTCGGCCCATGACCAGAACAGCAACCTTCTCATC 80

Mus     GAGTACTTCCGAAATGAGTTCGGGGAGCTGGAGAGCAACAAGAGCGAGATGATCTG 136
Rattus  GAGTACTTCCGAAACGAGTACGGGGAGCTGGAGAGCAACAAGAGCGAGATGATCTG 136
Cavia   GAGTACTTCCGAAACGAGTCTGGGGAGATCGAGGGGAACAAGAGCGAGATGATCTG 136
Homo    GAGTACTTCCGCAATGAGTTTGGGGAGATCCAGGGTGACAAGAGCGAGATGATCTG 136
```

ENSMUSG00000037820 exon 8 (ORF 1)

```
Mus     GAACTTCCACTGCTGGGTGGAGTCCTGGATGACCAGGCCAGACCTACAGCCGGGCTATGAGGGGTGGCAGGCCATTGACC 80
Rattus  GAATTTCCACTGCTGGGTGGAGTCCTGGATGACCAGGCCAGACCTACAGCCAGGCTATGAGGGGTGGCAGGCCATTGACC 80
Cavia   GAACTTCCACTGCTGGGTGGAGTCGTGGATGACCAGGCCGGACCTGGAGCCTGGGTACGAGGGGTGGCAGGCCCTGGACC 80
Homo    GAACTTCCACTGCTGGGTGGAGTCGTGGATGACCAGGCCGGACCTGCAGCCGGGGTACGAGGGCTGGCAGGCCCTGGACC 80

Mus     CCACACCACAGGAGAAGAGCGAAG 104
Rattus  CCACACCGCAGGAGAAGAGCGAAG 104
Cavia   CCACACCCCAGGAGAAGAGTGAAG 104
Homo    CAACGCCCCAGGAGAAGAGCGAAG 104
```

ENSMUSG00000037820_intron_7

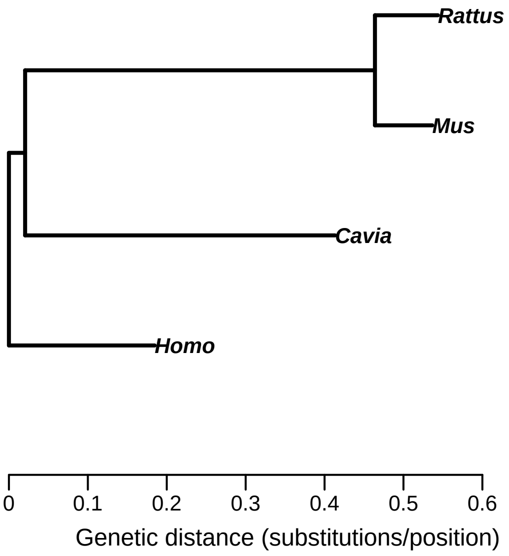

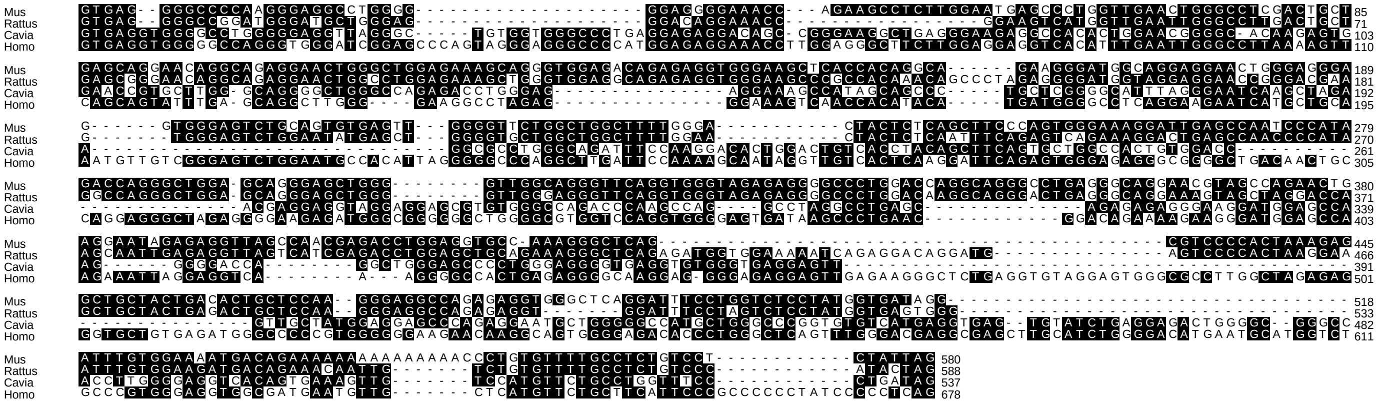

ENSMUSG00000039018 intron 8

Description: Mitochondrial GTPase 1 Precursor (Mtg1)
Intron number: 8
Mouse chromosome: 7
Upstream exon length: 97
Downstream exon length: 82
Mouse intron length: 345
Intron alignment length: 471
Total murinae branch length: 0.14684
K_score: 0.0614
Scaling factor: 0.75738

ENSMUSG00000039018 exon 8 (ORF 0)

```
Mus     GTATGTGAGCGTCCACTGGTATTCTTGCTGGATACTCCTGGGGTGCTGGCTCCTCGGATTGAAAGCGTGGAGACAGGCCT 80
Rattus  GTATGTGAGCGTCCACTGATGTTCTTGCTGGATACCCCTGGGGTGCTGGCTCCTCGGATTCAAAGTGTGGAGACAGGCCT 80
Cavia   GTGTCTGAGCGGCCACTGATGTTTCTATTGGACACCCCTGGGGTGCTGGCTCCTCGGATTGGAAGTGTAGAGACAGGCTT 80
Homo    GTCTCTGAGCGGCCCCTGATGTTCCTGTTGGACACTCCTGGCGTGCTGGCTCCTCGGATTGAAAGTGTGGAGACAGGCCT 80

Mus     GAAGCTGGCCCTGTGTG 97
Rattus  GAAGCTGGCCCTGTGTG 97
Cavia   GAAGCTGGCCTTGTGTG 97
Homo    GAAGCTGGCCCTGTGTG 97
```

ENSMUSG00000039018 exon 9 (ORF 2)

```
Mus     GAACTGTGTTGGACCACCTTGTTGGGGAAGAGACCATGGCCGATTATCTCCTTTACACCCTCAACAGGCATGGGCTCTTT 80
Rattus  GAACTGTGTTGGACCACCTTGTTGGGGAAGAGACCATGGCTGATTATCTCCTTTACACCCTCAACAGGCATGGGCTATTT 80
Cavia   GAACAGTGTTGGATCACCTGGTTGGGGAGGAGACCATGGCCGACTACCTCCTCTACACCCTTAACCAGCACCAGATGTTC 80
Homo    GAACGGTGCTGGACCACCTGGTCGGGGAGGAGACCATGGCTGACTACCTGCTGTACACCCTCAACAAACACCAGCGCTTT 80

Mus     GG 82
Rattus  GG 82
Cavia   AA 82
Homo    GG 82
```

ENSMUSG00000039018_intron_8

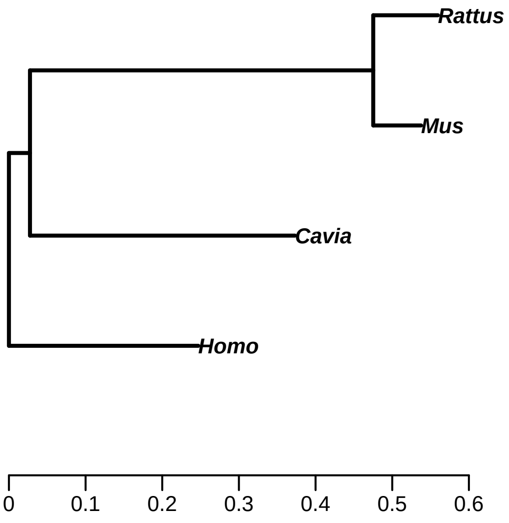

Genetic distance (substitutions/position)

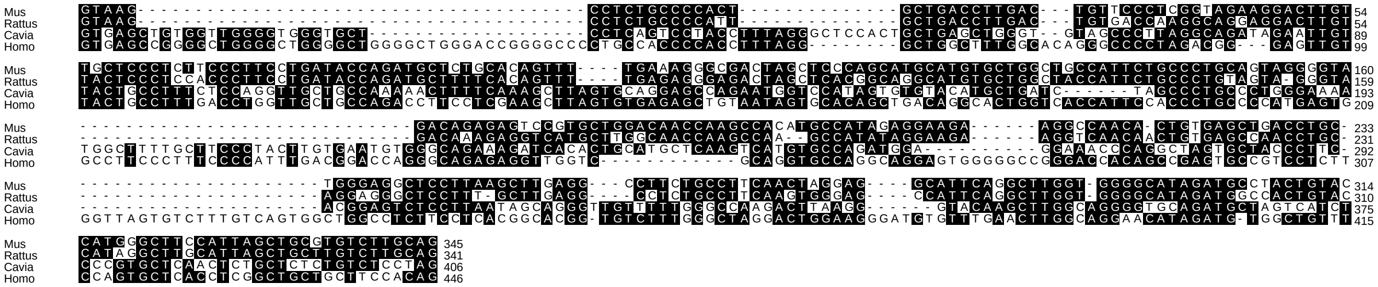
Mus
Rattus
Cavia
Homo

ENSMUSG00000008932 intron 6

Description: Excitatory amino acid transporter 5 (Slc1a7)
Intron number: 6
Mouse chromosome: 4
Upstream exon length: 100
Downstream exon length: 234
Mouse intron length: 551
Intron alignment length: 713
Total murinae branch length: 0.11760
K_score: 0.08609
Scaling factor: 0.75784

ENSMUSG00000008932 exon 6 (ORF 2)

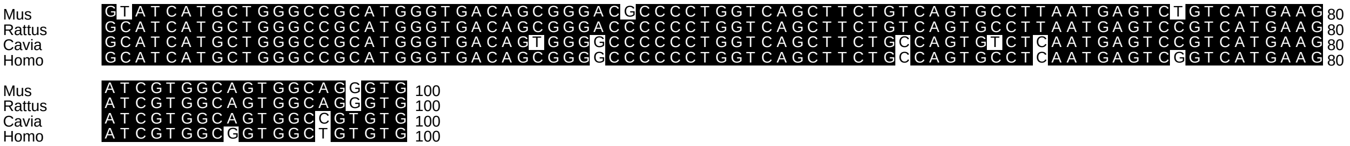

ENSMUSG00000008932 exon 7 (ORF 1)

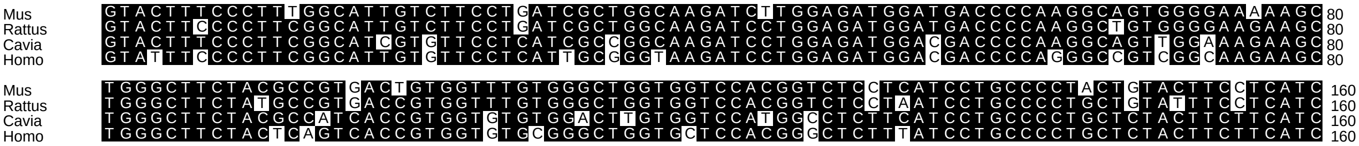

ENSMUSG00000008932_intron_6

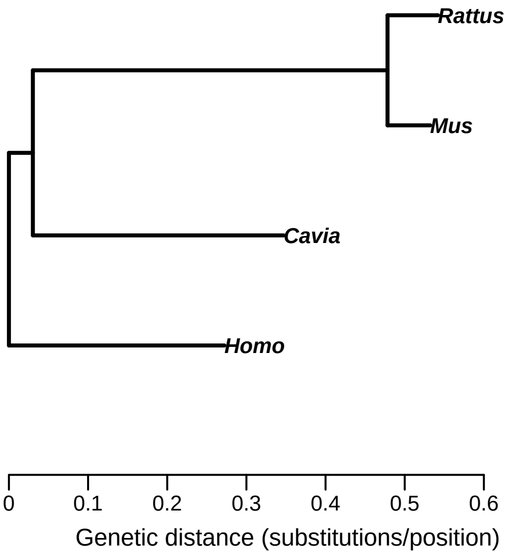

```
Mus     GTAAGTCTCCTCCAAGGAGAGCTCTCCTTCCCCTCCTCCTGCCCT----------------GGACACATCACTGAACCGACCTCAGTGTGTTCTT-AGGAGCTCAACAAA 93
Rattus  GTAAGTCTCCTCCAGGGAGAGTCCTCTTTCCCCCACTCCTGTCCT----------------GGACACATCACTGAACCATTCTCAGTGCGTTCTTAAGGAGCTCAACAAA 94
Cavia   GTAAGCCTCTCG--GGATGGGTCCCCTTTCGCCCCATCCACCCCTCTGGTTGCGGGTCCTTGGAGAGACACTGAGAGCTGTCCCATCCTCTGTGTCCAGAG--------- 99
Homo    GTAAGCCTCCTC--ATTTA---CCCACCTCACCCCGGCCAGCCCT--------GGGTGGATGGAGGGAAGACAAGACCCCACCCTCTTCTCCCGTAAAGTGAGCAGAAAG 97

Mus     CTGTACAGGAGGCTCAGCCTGGACCTGGGCCACCAGGGCTAGCTGACTGCACG---GGGC-TCCCAGTCCAATCCAGCCTCA---------------------------- 171
Rattus  CTGCACAGGAG-----GCCTGGACCTGGGCCACGAGGGCTAGCTGACTGACTGCACAGGT-TCCCAGCCCAATCCAGCCTTG---------------------------- 170
Cavia   --------AGACTCAGCCAGGGGCTGGGGCCTCCAGAGCTACCTG--TGTTTGCTTGGAT-TCCCAGCCCTGACCAGGCCTGGTTCCCCCAACCTGGTTCTGCCCCTCGC 198
Homo    CTGTGCCCAGGCTCAGCCCTGGGCCGGGGCCTCCGGAGCTACATG--TGTTTCCATGGGTCCCCCAGCCCCAGCCAGGCCTGGCTCCGGC--CCTGGGCCTGCCCCTTCC 203

Mus     ------------------------------CCTTCTGCTGTCTTGGCCTCGGTTTCCCTGTGTTGAGGAGCCTGCTTAGCTCGGCTCTTGAAGAAAGCGCACAGTGGAAG 251
Rattus  ------------------------------CCTTCTGCTGTCTTGGCCTCAGTTTCCCCATGTT---GAGCCTGCTTAGCTCGGCTCTTGAAGAAAGCATACAGTGGAAT 247
Cavia   AGCCTGTTGTGAAGGACAGCTCGGCCTGGTTTGACTGC-CTCTTGGCCTCAGTTTCCCCATACTATGGACTC----CAGTTCAAATTTTGAAGAAGGAACACAGTGGAAG 303
Homo    AGCCTGGTCTAAG----AGCTCTGCCCGGCTCTGCTGCTCTCCTGGGCTCAGTTTCTCCATCACATGGAGCCTTATCAGCTCAGCTTTTGGAGAAAGGA-AAGCTGGCGG 308

Mus     TGAGAATCAGTAACCACT--------CCTGGTTCAGAGTTCAGACCTTAGGTGCCTGATAGTCAGAGGAGTGTGCAGAGGACT-TGGTCACCTTGGAG----GCGTGGCC 348
Rattus  CGAGAATCAGTAACCAAT--------CCTGGTTCAGAGTTCAGACCTTAGATGCCTGAT----AGAGTTGTGTGCAGAGGACTGTGGTCACCTAGGAG----GCATGGCC 341
Cavia   GGAAAATCAGCTGCCA----------CTAAACTCCTGGTTTATATCTTAGGAATCCTCT-------AGGGCTTCCA--------GGGCCACTCAAGAG----GTGTGG-- 382
Homo    TAAGGACTAGCCCACAGTTCAGAGTCCTCGGCCCACAGTTCAGAGTCCAGGAGCTCAGT-------GGGGAACGCAGAGGGA--GGGCTGCCCAGGCGCACTGTGTAGAC 409

Mus     TTG--------CTGAGCTAAAATGGGGCTTCTTCAAGGACATGACAG--------------------GCTATCTAGGGCCAGGGCAACTGAGGGA--------------- 415
Rattus  TTG--------CTGAGCTAAAATGAGGCTTCTTCAAGGACATAGCAG--------------------TCTGTCTAGGGCTGGGGCAACTGAGGGA--------------- 408
Cavia   -----------CATGGCTGGAATGGGGTTGCCCGAGGGAAGGGACAGAGTAGGCAGGCAGGCCAGAGGTTGTCCAGG--CAGGGTGCTTGAGGCAAGGGCACCTACAATG 479
Homo    TCTGGCGTTGTTGGGCCTGAAATGGGGGCCTCCTGAAGGCAGGGCGG---GGGAAGGAGTGGCAGAGGTTGTCTGGAGCTGGGGCGGTGGAAGTAACGGTATGAAGGACG 516

Mus     ------CCTGGCTCTCAGCTGGGCACCT--------CAGTCTCACCCAGTCCTCCT--GAGCCATTAGGAGCTTGA-GATGAGATCACACAGTGACAAGGTGCCAGGTAG 508
Rattus  ------CCTGGCTCTCAGCTGGGCACCT--------CAGTCAGTCTCA-CCCTTGT--GAGCCACAAGGGGCTTGA-GATGAGATCACACAGTGACAAGGTGCCAGGTGG 500
Cavia   CTACCT-----------GCAGGATACCTTGCTTACACAGCCTTGTCCCAGCCTCGCATGAGCCCC--GAGGCCCAG-----------------ATTGTGGCACCGAGGCT 559
Homo    CCATGTGCTGAGCCTGGGCCGGGCACCTTG-----GCAGTCTCGCTCTGTCCTCGCAGGAGCCCCAGGAAGCTCAGGGGGAAGGCCGCGCAGCGATGCGGGGCTGGGGCC 621

Mus     GAGGGGAT---------CCTGAACTGAACCCGAGACCACTCTGCCA-CCCCAG 551
Rattus  GAGGGGAC---------CCTGAATTGAACCCGAGGCCATTCTGCCA-CTCCAG 543
Cavia   GCAAACCTGGCT--CTGCCTAGCACCGAGGCGAGGCTGTCCCGTCA-CCCCAG 609
Homo    G-GGGTGTGTCTGCCTGCCTGGTTCCTGAGCCTGGCTGCCCCGCCGCCCCCAG 673
```

ENSMUSG00000024833 intron 14

Description: DNA polymerase alpha subunit B (Pola2)
Intron number: 14
Mouse chromosome: 19
Upstream exon length: 109
Downstream exon length: 107
Mouse intron length: 225
Intron alignment length: 311
Total murinae branch length: 0.20809
K_score: 0.04635
Scaling factor: 0.75822

ENSMUSG00000024833 exon 14 (ORF 1)

```
Mus     CTCCGGCTCCCACCTTGTCTTTGTCCCGTCTCTGAGGGATGTCCATCATGAGCCAGTATACCCACAGCCGCCTTTCACCT 80
Rattus  CTCCGGCTCCCACCTTGTCTTTGTCCCGTCTCTGAGGGATGTGCACCACGAGCCAGTCTACCCACAGCCACCTTTCACCT 80
Cavia   CTCCGGCTCCCACCTTGTCTTTGTCCCATCCCTGAGAGACGTGCACCACGAGCCTGTGTACCCGCAGCCGCCATTCAGCT 80
Homo    CTCCGGCTCCCACCTTGTCTTTGTCCCGTCATTGAGAGATGTGCACCATGAGCCTGTGTACCCCCAGCCGCCTTTCAGCT 80

Mus     TCTCCGAGCTGTCTCGAGAGGACAAAAAG 109
Rattus  TCTCCGAACTGCCTCGAGAGGACAAGAAG 109
Cavia   ACCCCGATCTGCCTCGAGAGGACAGAAAG 109
Homo    ACTCCGATCTGTCTCGAGAGGACAAAAAG 109
```

ENSMUSG00000024833 exon 15 (ORF 0)

```
Mus     CGAGTGCAGTTTGTGTCAGAGCCCTGCAGCCTCTCCATAAATGGAGTGATGTTTGGTTTGACATCCACGGACCTGCTGTT 80
Rattus  CGAGTGCAGTTTGTGTCAGAGCCCTGCAACCTCTCCATAAATGGGGTGATGTTTGGTTTGACATCCACGGACCTGCTGTT 80
Cavia   CGGGTACAGCTGGTGTCTGAGCCCTGCAGCCTCGCCATAAATGGGGTGATCTTCGGCTTGACATCCACGGACCTGCTGTT 80
Homo    CAAGTACAGTTTGTGTCCGAGCCCTGCAGCCTCTCCATAAACGGAGTGATCTTCGGCTTGACATCCACAGATCTGCTTTT 80

Mus     CCACATCGGGGCTGAGGAGATCTTTAG 107
Rattus  CCACATTGGGGCCGAGGAGATCTGTAG 107
Cavia   CCACATGGGGGCCGAAGAGATCTGCAG 107
Homo    CCACCTGGGGGCCGAGGAGATCAGTAG 107
```

ENSMUSG00000024833_intron_14

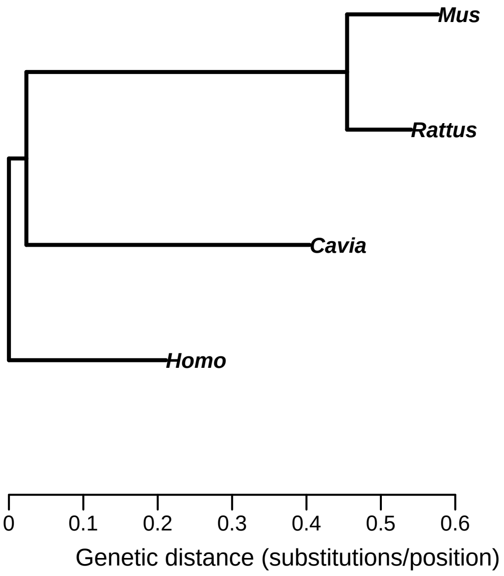

```
Mus     GTACCAGTGCC--------------------------------CGGCTCGGGCCGGCCATCTGTGGGTGGGGTGAAGACCGCCCTGGGGAGGAGGGCAGAGAAACCCTCT 78
Rattus  GTACCAGTGCCTGCC----------------------------CACCTCTGGCCAGCCAGCAGTGGGTGGGGTGAAGACAGCCCTGGCAGTGAGGGCAGAGGAGCCCTCT 82
Cavia   GTACCA--------------------------------------TGCTCCCTGCACCCGGCTGCA---------------GCCCTGAACGGGAGGGTGGAAAGGGCTCCT 57
Homo    GTAGCAGCACCCACTCCTTGACCAGCAGGCAGCCAGAATCCAGCATGCCTCTGCCACTAGCTCTG---------------GCCCTGAAGGGGAGGGTGGAGAGGCCTCTT 95

Mus     CAGGAGG------GAGCACAGCCCCTAAGTCCG-----------CAACAGAGATGTCATAGG--ACCACCCTTGG-GTGGTCCTTGGTGGTCTGCAGGCCCCAGCTTTAG 168
Rattus  CAGGATC------GGGCAAAGCCCCTAAGTGCC-----------CAGCATAAGTGTCATAGGTTGCTGCACTTGGCTTGGTCCCCAGTGGTCTGCAGGCCCCATCTTCGG 175
Cavia   CTGGGTACACACATAACTC-ACTCTTGAACTCCTTTTCGTGA--CGGCAAAGGTGGGTGTTG------------TTTCGCTGCACATGGGTTTG---TCTTTTTCCATGG 149
Homo    TAGGGTGTGCGTACAGCCTGAATCTTAAACTCCTTTTTTTAAACAGGCAAATGTGTCCAAAA------------GCTTGATGTATGTTGGTTTGGTCCCCCTTTCCGTGG 193

Mus     TG-------------------------------GGAAAGAACCCTCTGGCTGTTGGATGCGAGTATTCATGTCAGC---TCCTCCTTTCAG 225
Rattus  TG-------------------------------GGGAAGAGCTCTCT---TGTTGGATGCAAGTGTTCATGTCAGC---TCCTCCTTTCAG 229
Cavia   TCACCTGCCATCTCG------------------GGGAAGGAGTTTCTGGCTGTTCTATAAATGTGTGCATCGCCCC---TTCTCGTTCTAG 219
Homo    TCTCCCCAGCCCTCAGACAACCCCCACTTTCATGGGAAGATGTTTCTAGCTTTGGCATACAAATGTTTGTTTCAATCCGTTCTCATTTTAG 284
```

ENSMUSG00000020315 intron 33

Description: Spectrin beta chain, brain 1 (Spnb2)
Intron number: 33
Mouse chromosome: 11
Upstream exon length: 174
Downstream exon length: 43
Mouse intron length: 1592
Intron alignment length: 1921
Total murinae branch length: 0.18963
K_score: 0.08678
Scaling factor: 0.75832

ENSMUSG00000020315 exon 33 (ORF 1)

```
Mus     TATATTGTGTCATAAATAACCAAGAAATGGGCTTCTATAAAGATGCCAAGAGTGCTGCTTCTGGCATCCCCTACCACAGT 80
Rattus  TTTATTGTGTCATAAATAACCAAGAAATGGGCTTCTACAAAGATGCCAAGAGTGCTGCTTCTGGCGTCCCCTACCACAGT 80
Cavia   TTTATTGTGTCATAAATAACCAAGAAATGGGTTTCTATAAAGATGCCAAGACTGCTGCTTCTGGAATCCCCTACCACAGT 80
Homo    TTTATTGTGTCATAAATAACCAAGAAATGGGTTTCTACAAAGATGCAAAGACTGCTGCTTCTGGAATTCCCTACCACAGC 80

Mus     GAGGTCCCTGTGAGTTTGAAAGAGGCCATCTGCGAAGTGGCCCTTGATTACAAAAAGAAGAAGCACGTGTTCAAGCTAAG 160
Rattus  GAGGTCCCTGTGAGTTTGAAAGAGGCCATCTGCGAAGTGGCCCTTGATTACAAGAAGAAGAAGCATGTGTTCAAGCTAAG 160
Cavia   GAGGTCCCTGTGAGTTTGAAAGAAGCTGTCTGTGAAGTAGCTCTTGATTACAAAAAGAAGAAACACGTGTTCAAGCTAAG 160
Homo    GAGGTCCCTGTGAGTTTGAAAGAAGCTGTCTGCGAAGTGGCCCTTGATTACAAAAAGAAGAAACACGTATTCAAGCTAAG 160
```

ENSMUSG00000020315 exon 34 (ORF 1)

```
Mus     ACTAAGTGATGGAAACGAGTACCTCTTCCAAGCCAAAGATGAT 43
Rattus  ACTAAGTGATGGCAACGAGTACCTCTTCCAAGCCAAAGATGAT 43
Cavia   ACTAAATGATGGCAATGAGTACCTCTTCCAAGCCAAAGATGAT 43
Homo    ACTAAATGATGGCAATGAGTACCTCTTCCAAGCCAAAGACGAT 43
```

ENSMUSG00000020315_intron_33

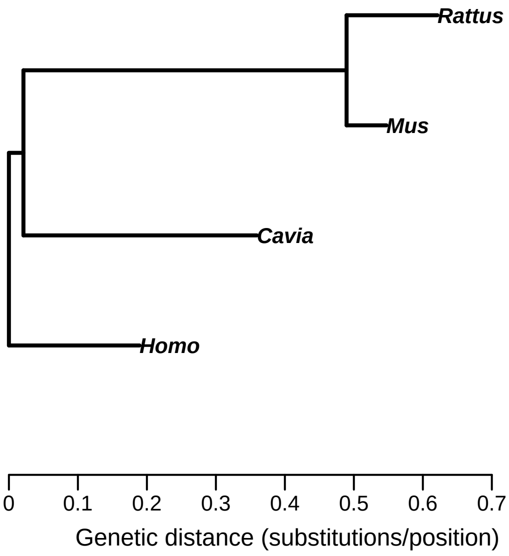

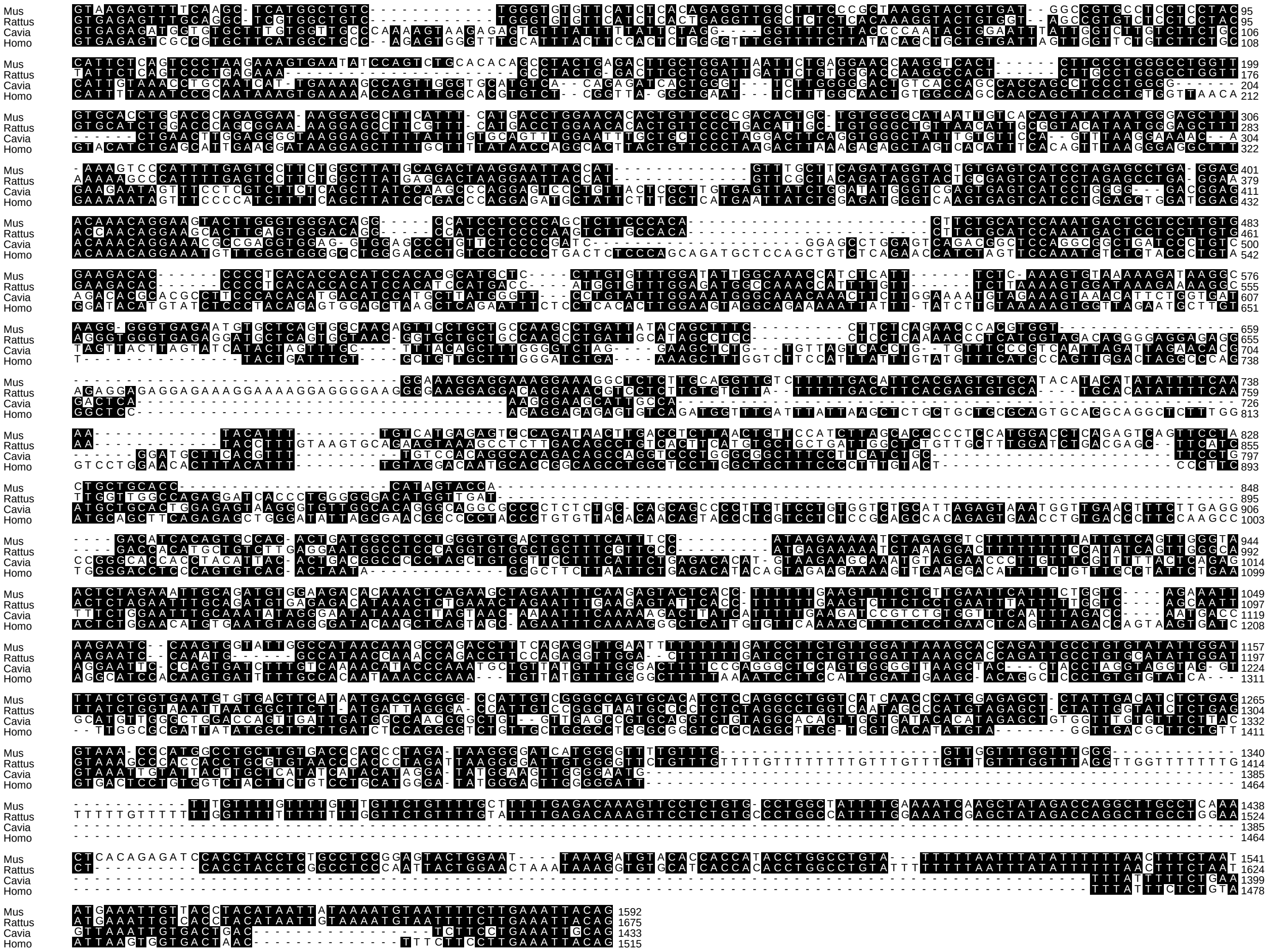

ENSMUSG00000041202 intron 3

Description: Group IID secretory phospholipase A2 Precursor (Pla2g2d)
Intron number: 3
Mouse chromosome: 4
Upstream exon length: 107
Downstream exon length: 146
Mouse intron length: 792
Intron alignment length: 1342
Total murinae branch length: 0.21997
K_score: 0.04952
Scaling factor: 0.75883

ENSMUSG00000041202 exon 3 (ORF 1)

```
Mus     GTGCTGTCAGAAGCATGATTGTTGCTATGCCCACCTGAAGATCGATGGATGCAAGAGCCTGACAGACAACTACAAATACA 80
Rattus  GTGCTGTCAAAAGCACGATTGTTGCTATGCCCACCTGAAGATCGATGGATGCAAAAGCCTGACAGACAATTACAAATACA 80
Cavia   GTGCTGCCTGGAACACGATTGCTGTTATTCCCTCCTGAAGACTCAGCGATGCAAGCCCCAGACAAATCACTACAAATACA 80
Homo    GTGCTGCCAGACCCATGACTGCTGCTATGACCACCTGAAGACCCAGGGGTGCAGCATCTACAAGGACTATTACAGATACA 80

Mus     GCATCTCCCAGGGCACTATCCAGTGCT 107
Rattus  GCATCTCTGAGGGCGTTATCCAATGCT 107
Cavia   ACTTTTCCCTGGGAGACATCCAGTGCT 107
Homo    ACTTTTCCCAGGGGAACATCCACTGCT 107
```

ENSMUSG00000041202 exon 4 (ORF 2)

```
Mus     CTGACAACGGGAGCTGGTGTGAAAGGCAACTGTGTGCTTGTGACAAGGAGGTGGCCTTGTGCTTGAAGCAAAACCTGGAT 80
Rattus  CTGACCAAGGGAGCTGGTGCGAAAGGCAACTGTGTGCTTGTGACAAGGAGGTGGCCTTGTGCCTGAAGCAAAACCTGGAG 80
Cavia   CTGACAAGGGCAGCTGGTGCGAGAAGCAGCTGTGTGCCTGTGACAAGGCGATGGCCTTCTGCCTGAAGAGCAGCCTGAAC 80
Homo    CTGACAAGGGAAGCTGGTGTGAGCAGCAGCTGTGTGCCTGTGACAAGGAGGTGGCCTTCTGCCTGAAGCGCAACCTGGAC 80

Mus     AGCTACAATAAGCGCCTGCGTTACTACTGGCGGCCCCGTTGCAAAGGCAAGACTCCAGCATGCTAA--------- 146
Rattus  AGCTACAACAAGCGACTGCGGTACTACTGGAGGCCCCGCTGCAAAGGCCAGACTCCCACATGCTAG--------- 146
Cavia   ACCTACCAGAAGAAGCTGCGATTCTACCTGAGGCCCAAATGCAAGGGCACGACTCCTCCATGCACAGGAGCTGCC 155
Homo    ACCTACCAGAAGCGACTGCGTTTCTACTGGCGGCCCCACTGCCGGGGGCAGACCCCTGGGTGCTAG--------- 146
```

ENSMUSG00000041202_intron_3

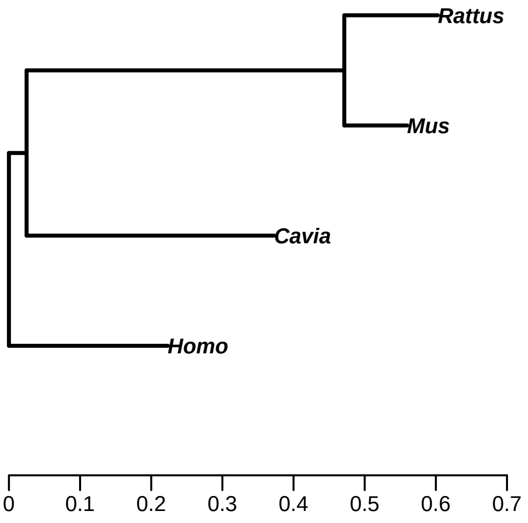

Genetic distance (substitutions/position)

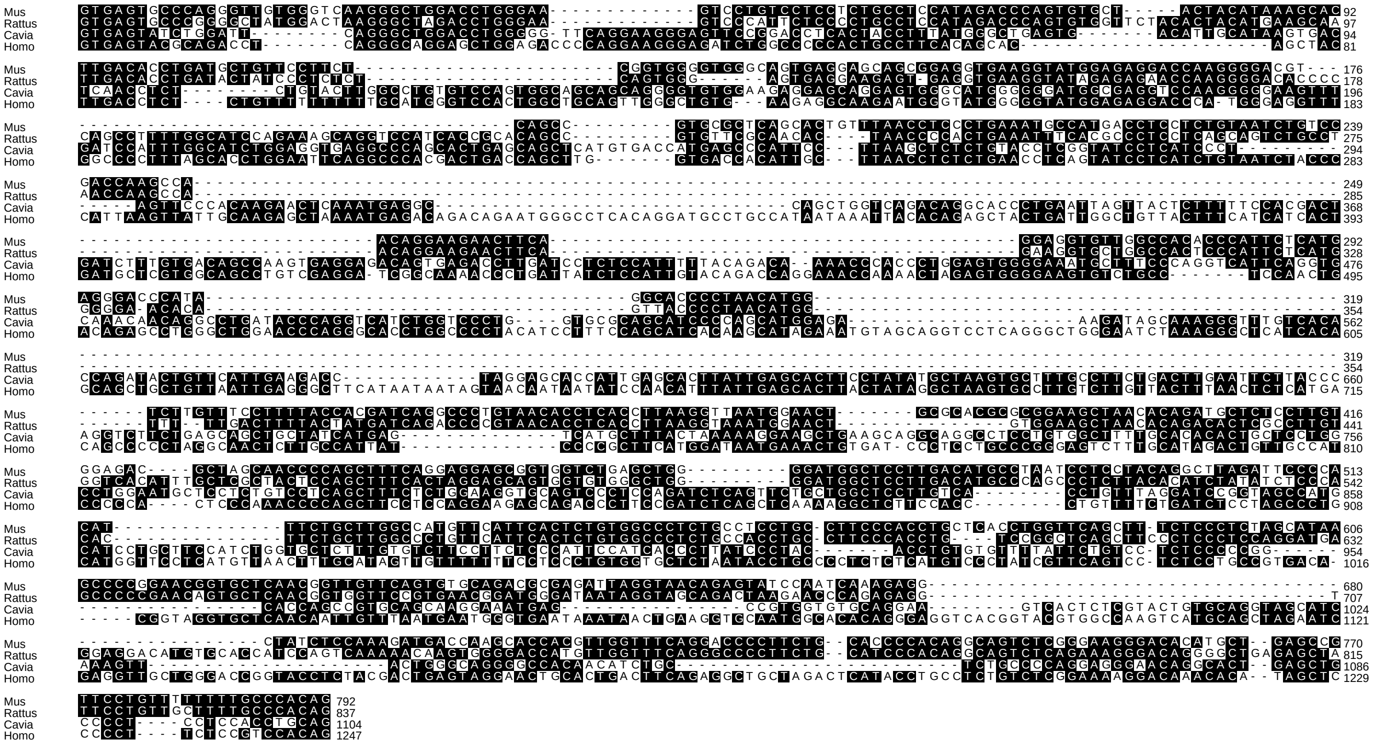

ENSMUSG00000027315 intron 7

Description: Kunitz-type protease inhibitor 1 Precursor (Spint1)
Intron number: 7
Mouse chromosome: 2
Upstream exon length: 51
Downstream exon length: 171
Mouse intron length: 1363
Intron alignment length: 1658
Total murinae branch length: 0.21882
K_score: 0.05026
Scaling factor: 0.76136

ENSMUSG00000027315 exon 7 (ORF 2)

```
Mus     ACACCAGCGGCTTTGATGAGCTTCAGAATATCCATTTCCTCAGTGACAAAG 51
Rattus  ATAGCAGCGGCTTTGATGAACTTCAGAGTATCCACTTCCTCAGTGACAAAG 51
Cavia   ATACCACTGGCTTTGATGAGCTCCAGAACATCCATTTCCCCAGTGACAAAG 51
Homo    ACACGAGTGGCTTTGACGAGCTCCAGCGCATCCATTTCCCCAGTGACAAAG 51
```

ENSMUSG00000027315 exon 8 (ORF 2)

```
Mus     GGTACTGTGCAGAGCTGCCAGACACTGGGTTTTGCAAGGAGAACATCCCACGCTGGTATTACAACCCATTCAGTGAACGC 80
Rattus  GGTACTGTGCAGAGCTGCCAGACACTGGATTTTGCAAGGAGAACATCCCACGCTGGTATTACAACCCATTCAGTGAACGC 80
Cavia   GGCACTGCGTGGACCTGCCAGATACAGGGGTCTGCCAGGAGAGCATCCCGCGCTGGTACTACAACCCCCTTTAGTGAACAC 80
Homo    GGCACTGCGTGGACCTGCCAGACACAGGACTCTGCAAGGAGAGCATCCCGCGCTGGTACTACAACCCCCTTCAGCGAACAC 80

Mus     TGTGCCCGATTCACCTATGGTGGTTGCTATGGGAACAAGAACAACTTTGAGGAGGAACAGCAGTGTCTTGAGTCCTGCCG 160
Rattus  TGTGCCCGCTTCACCTATGGTGGTTGCTACGGGAACAAGAACAACTTTGAGAAGGAACAGCAGTGTCTTGAGTCCTGTCG 160
Cavia   TGCGACCGCTTTACCTACGGTGGTTGTTACGGCAACAAGAACAACTTTGAGGAAGAGCAGCAATGTCTGGAATCCTGTCG 160
Homo    TGCGCCCGCTTTACCTATGGTGGTTGTTACGGCAACAAGAACAACTTTGAGGAAGAGCAGCAGTGCCTCGAGTCTTGTCG 160
```

ENSMUSG00000027315_intron_7

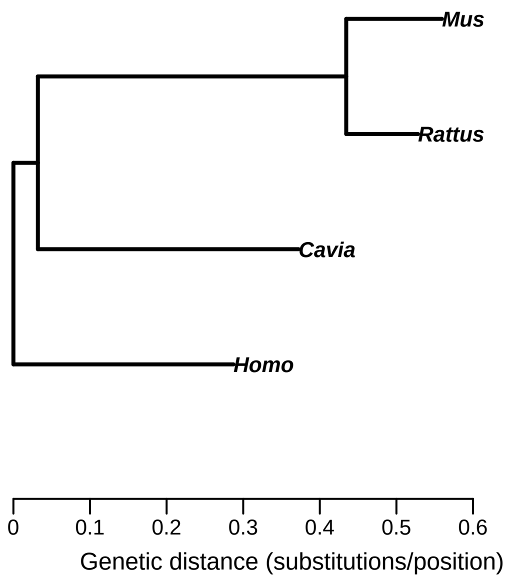

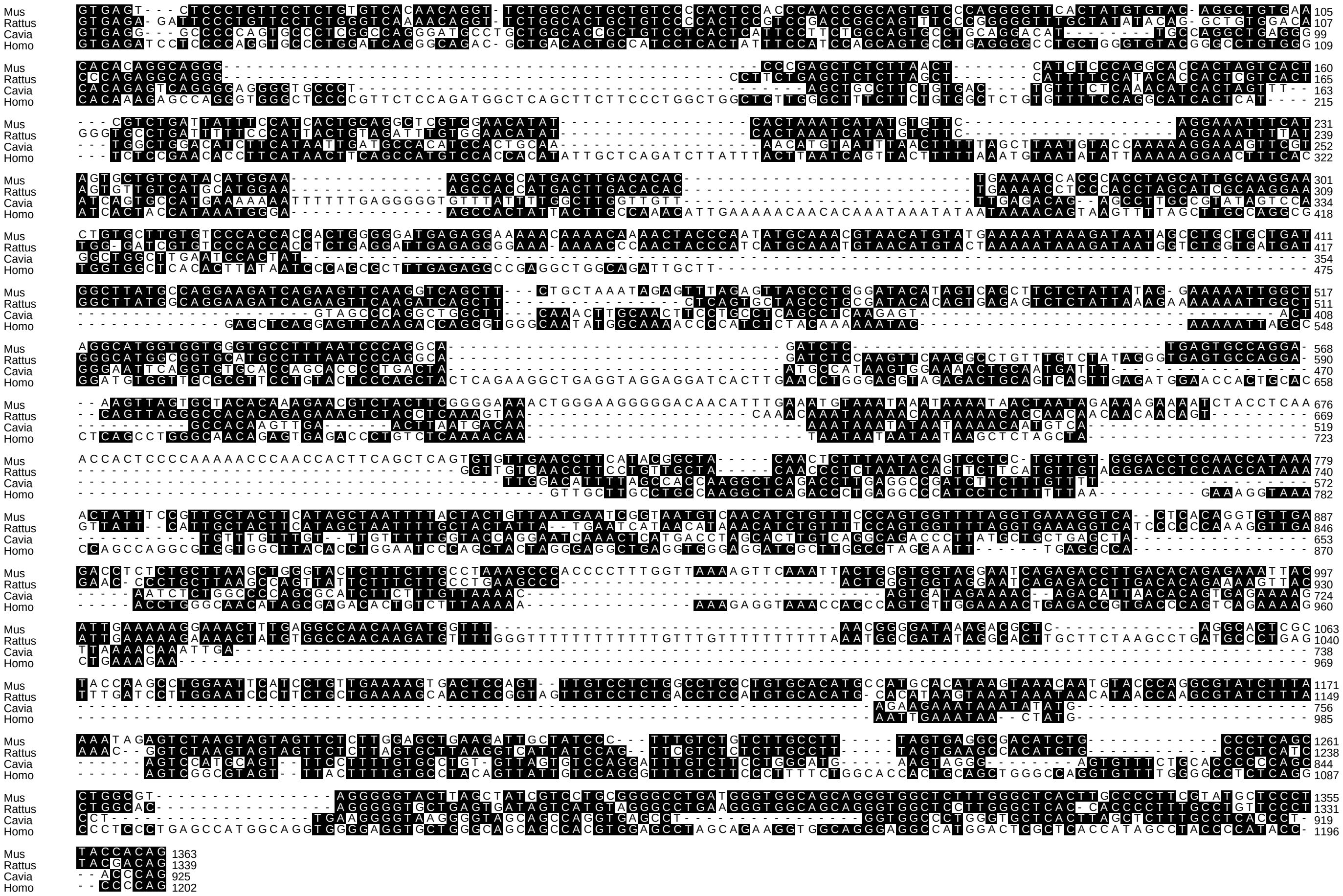
Mus
Rattus
Cavia
Homo

ENSMUSG00000035314 intron 10

Description: Glycerophosphodiester phosphodiesterase domain-containing pr (Gdpd5)
Intron number: 10
Mouse chromosome: 7
Upstream exon length: 219
Downstream exon length: 158
Mouse intron length: 472
Intron alignment length: 592
Total murinae branch length: 0.13595
K_score: 0.06386
Scaling factor: 0.7639

ENSMUSG00000035314 exon 10 (ORF 0)

```
Mus     CCAGTCCATCTGCAGCCTGGCAGAGCTGCTGGAGCTGGCCAAGGGCAATGCCTCGCTGCTGCTCAACCTTCGGGACCCGC 80
Rattus  CCAGTCCATCTGCAGCCTGGCAGAGCTCCTGGAGCTAGCCAAAGGCAACGCTACTCTGCTGCTCAACCTTCGGGACCCAC 80
Cavia   CCAATCCATTTGTAGCCTGGATGAGCTGCTGGAGCTGGCTAAGGGCAACGCCACGCTGCTGCTCAACCTGCGGGAACCAC 80
Homo    CCAGTCCATCTGCAGCCTGGCAGAGCTCCTGGAGCTGGCCAAGGGCAATGCCACACTGCTGCTCAACCTGCGTGACCCGC 80

Mus     CCCGTGATCACCCCTACCGTGGCAGCTTCCTCAACGTCACACTGGAGGCCGTGCTGCGTTCAGGCTTCCCCCAGCACCAG 160
Rattus  CCCATGATCACCCGTACCGTGGCAGCTTCCTCAACATCACACTGGAGGCCGTGCTCCGTTCAGGCTTTCCACAGCACCAG 160
Cavia   CCCGAGACCATCCCTTTCGAAGCAGCTTTATTAATGTCACGCTGGAGACCCTGCTGCGCTCTGGCTTCCCGCAGCACCAG 160
Homo    CCCGGGAGCACCCCTACCGCAGCAGTTTTATCAACGTGACTCTGGAGGCCGTGCTGCACTCCGGCTTCCCCCAGCACCAG 160
```

ENSMUSG00000035314 exon 11 (ORF 0)

```
Mus     GTCATGTGGCTATTTAACAGGCAGAGGCCTTTGGTACGGAAGATGGCTCCTGGCTTCCAGCAAACATCTGGATCCAAAGA 80
Rattus  GTCATGTGGCTGTTTAACAGGCAGAGGCCTTTGGTACGGAAGATGGCTCCTGGCTTCCAGCAAACATCTGGGTCTAAAGA 80
Cavia   GTCATGTGGCTGCCTAACAGGCAGAGGCCCTTCGTGCGGAAGGTGGCTCCTGGCTTCCAACAGACGGCAGGCTCGAAGGA 80
Homo    GTCATGTGGCTGCCTAGCAGGCAGAGGCCCCTGGTGCGGAAGGTGGCTCCCGGCTTCCAACAGACATCAGGCTCCAAGGA 80

Mus     AGCCATCGCTAACTTACGGAAAGGTCACATCCAGAAGCTGAACCTCCGCTACACTCAGGTGTCCCACCAGGAGCTCAG 158
Rattus  AGCCATCGCCAGCCTACGGAGAGGTCACATCCAGAAGCTCAACCTCCACTATACTCAGGTGTCCCACCAGGAGCTCAG 158
Cavia   GGCATTGGCCAGCCTGCGGAGAGGCCATATCCAGCGGCTGAACCTGCGCTACACTCAGGTGTCCAGCCAGGAGCTCAG 158
Homo    GGCAGTCGCCAGCCTGCGGAGAGGCCACATCCAGCGGCTGAACCTGCGCTACACTCAGGTGTCCCGCCAGGAGCTCAG 158
```

ENSMUSG00000035314_intron_10

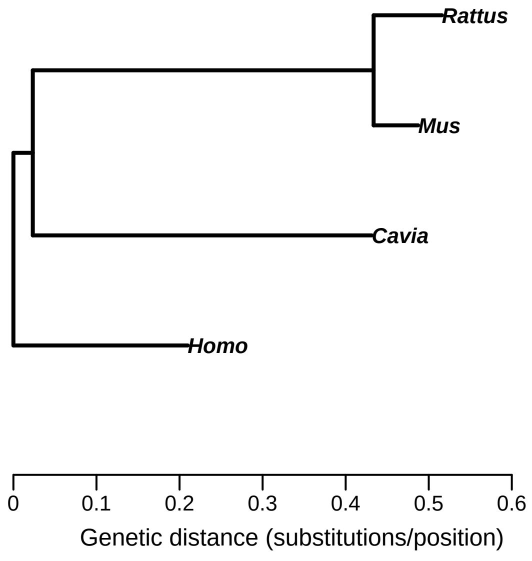

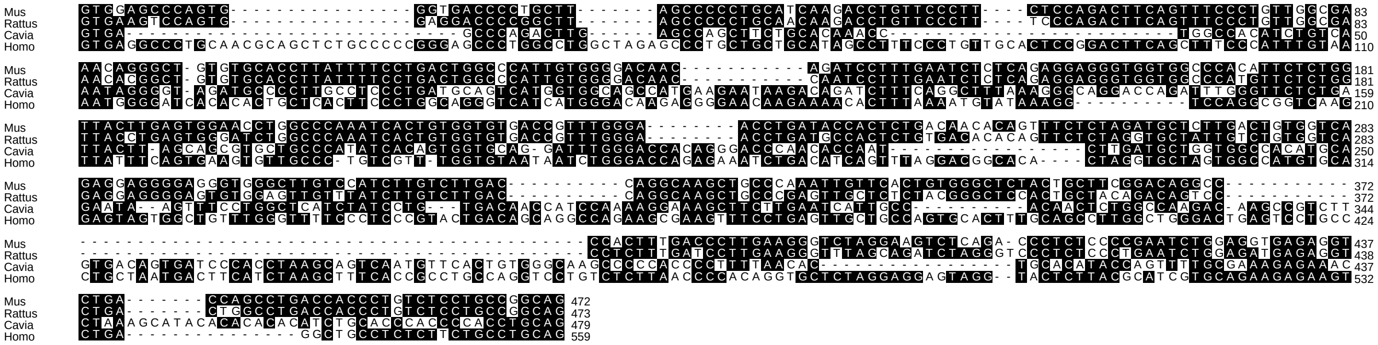
Mus
Rattus
Cavia
Homo

ENSMUSG00000063704 intron 1

Description: Mitogen-activated protein kinase 15 (Mapk15)
Intron number: 1
Mouse chromosome: 15
Upstream exon length: 69
Downstream exon length: 99
Mouse intron length: 916
Intron alignment length: 1595
Total murinae branch length: 0.18664
K_score: 0.0733
Scaling factor: 0.76517

ENSMUSG00000063704 exon 1 (ORF 0)

```
Mus     ATGTGTGCTGCCGAGGTGGACCGTCATGTAGCCCAGAGATACCTGATCAAGCGGAGGCTTGGGAAGGGG 69
Rattus  ATGTGTGCTGCCGAGGTGGACCGTCATGTATCCCAGAGATACCTGATCAAGCGGAGGCTTGGGAAGGGG 69
Cavia   ATGTGTACCGCCGAGGTGGACAGGCATGTAGTCCAGAGATACCTGCTCAAGCGGCAGCTCGGGAAGGGG 69
Homo    ATGTG---CACCGTAGTGGACCCTCGCATTGTCCGGAGATACCTACTCAGGCGGCAGCTCGGGCAGGGG 66
```

ENSMUSG00000063704 exon 2 (ORF 0)

```
Mus     GCCTATGGCATTGTGTGGAAGGCCATGGACCGGAGGACTGGCGAGGTTGTGGCCATCAAGAAAATCTTTGATGCCTTTAG 80
Rattus  GCCTACGGCATTGTATGGAAGGCCATGGACCGGAGGACTGGTGAGGTAGTGGCCATCAAGAAAATCTTTGATGCCTTTAG 80
Cavia   GCCTATGGTATTGTGTGGAGGGCAGTGGATCGGAGGACTGGTGAGGTTGTGGCCATCAAGAAAATCTTTGATGCCTTTAG 80
Homo    GCCTATGGCATTGTGTGGAAGGCAGTGGACCGGAGGACTGGTGAGGTCGTGGCCATCAAGAAAATCTTTGATGCTTTTAG 80

Mus     GGACCAGATAGATGCTCAG 99
Rattus  GGACCAGACAGATGCTCAG 99
Cavia   GAATAAGACAGATGCTCAG 99
Homo    GGATAAGACAGATGCCCAG 99
```

ENSMUSG00000063704_intron_1

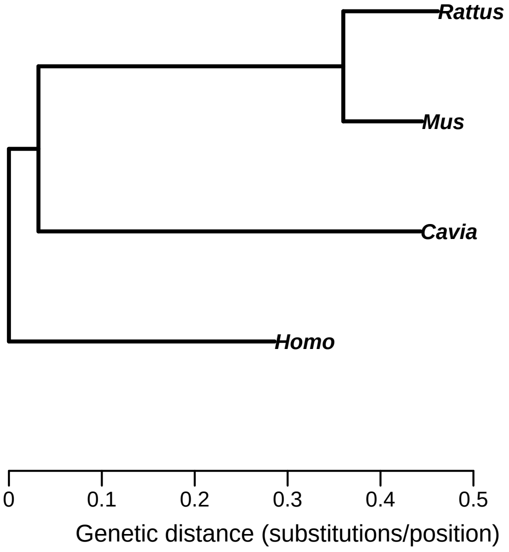

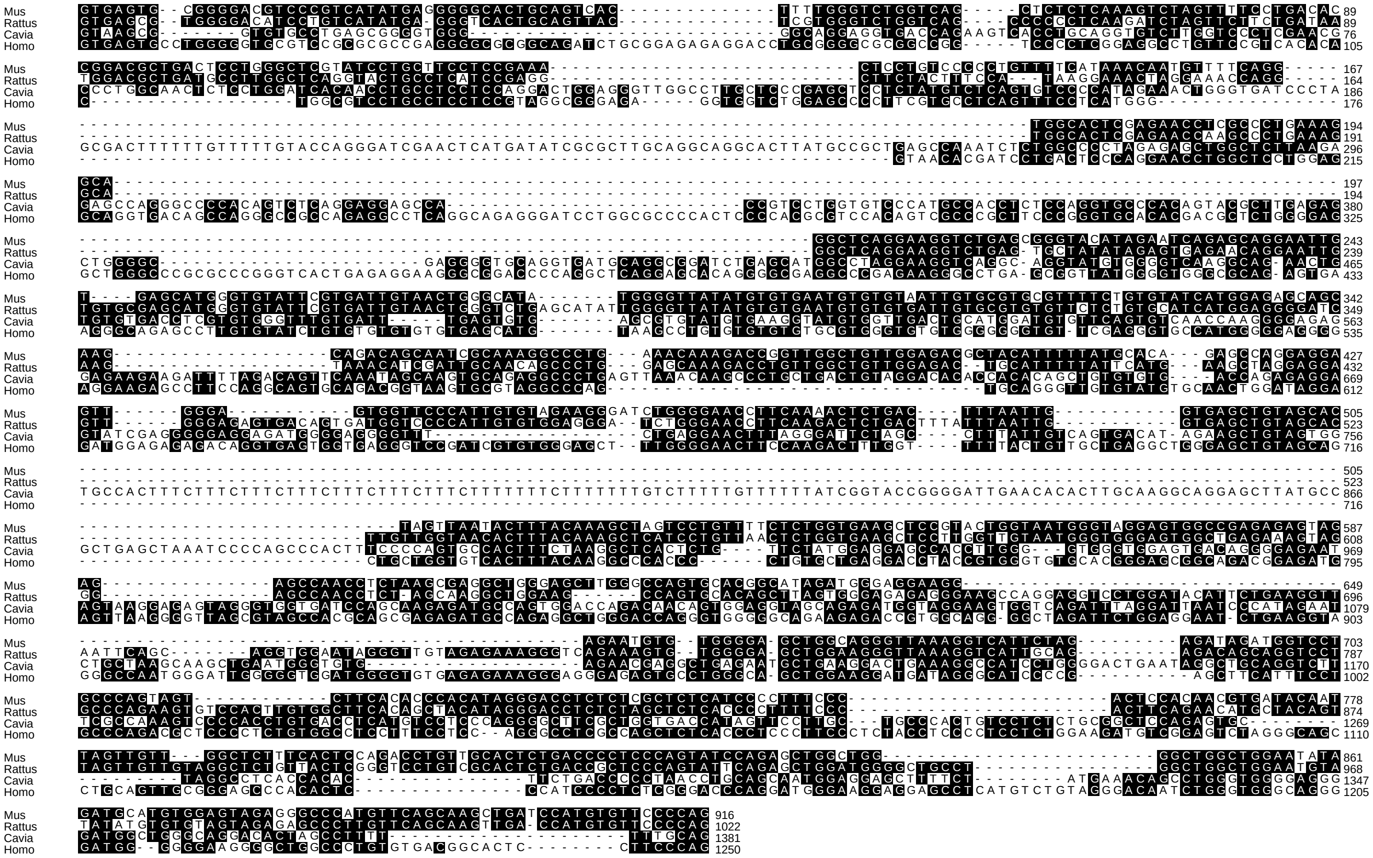
Mus
Rattus
Cavia
Homo

ENSMUSG00000023367 intron 2

Description: Transmembrane protein 176A (Tmem176a)
Intron number: 2
Mouse chromosome: 6
Upstream exon length: 111
Downstream exon length: 57
Mouse intron length: 1085
Intron alignment length: 1189
Total murinae branch length: 0.18632
K_score: 0.04616
Scaling factor: 0.76627

ENSMUSG00000023367 exon 2 (ORF 0)

```
Mus     GTGGTGCAGACTGTGCTGGGGGCTCTGAGTGTGGTTCTGGGTGGAACCCTCTACATAGGCCATTATTTAGCCATGTATTC 80
Rattus  GTGGTGCAGATCGTGCTGGGGATCCTGAGTGTGGTTCTGGGCGGAATTCTCTACATCTGCCACTATTTAGCCATGAATAC 80
Cavia   GTGGTACAGATCATGCTGGGACTCCTAAGCGGGGTCCTGGGAGGATTCCTCTACATCTTCCAGTGCCGCACCCTGTGCAC 80
Homo    GTGATGCAGATCGTGCTGGGGATCTTGAGTGCAGTCCTAGGAGGATTTTTCTACATCCGCGACTACACCCTCCTCGTCAC 80

Mus     CGAAGGCGCCCCCTTCTGGACTGGGATCGTG 111
Rattus  CCAGGGTGCCCCATTCTGGACCGGGATCGTG 111
Cavia   CTCAGGTGCCGCCCTCTGGACTGGGGTCGTG 111
Homo    CTCGGGAGCTGCCATCTGGACAGGGGCTGTG 111
```

ENSMUSG00000023367 exon 3 (ORF 0)

```
Mus     GCTATGCTGGCTGGAGCTGTTGCCTTCCTTCACAAGAAACGGGGTGGTACCTGCTGG 57
Rattus  GCTATGCTGGCTGGAGCTGTCGCCTTTCTTCAAAAGAAACGGGGTGGTACCTGCTGG 57
Cavia   GCTGTGCTGGCTGGAGCTGCTGCCTTTGTGTACAATAAACGAGGTGGGATCTGTTGG 57
Homo    GCTGTGCTGGCTGGAGCTGCTGCCTTCATTTACGAGAAACGGGGTGGTACATACTGG 57
```

ENSMUSG00000023367_intron_2

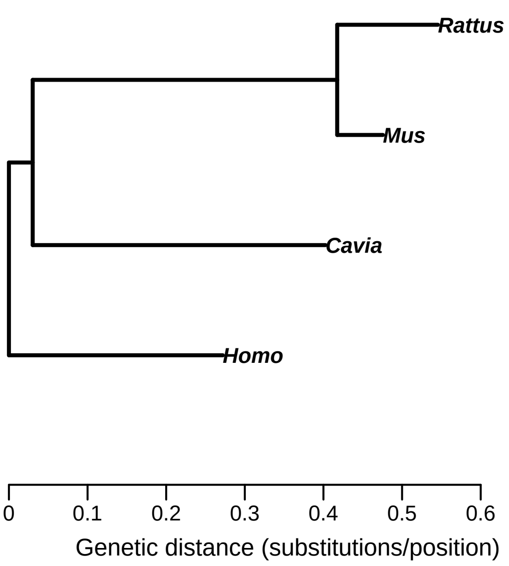

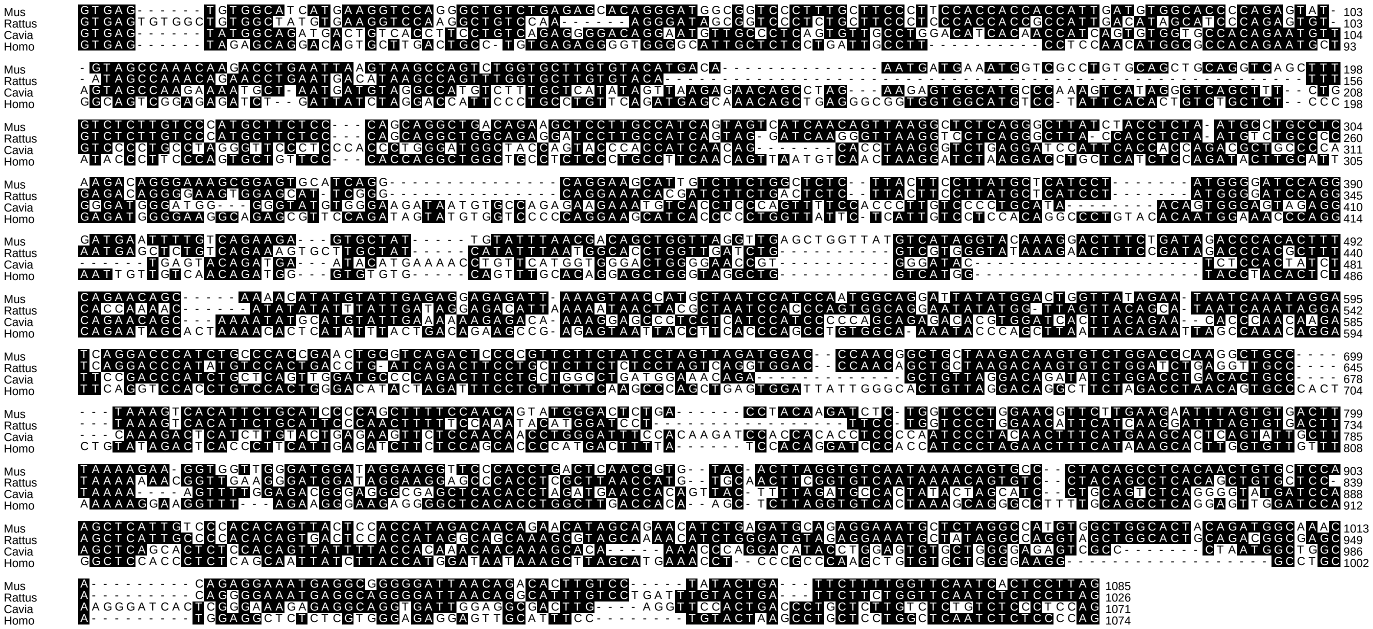
Mus
Rattus
Cavia
Homo

ENSMUSG00000022390 intron 14

Description: ubiquitous tetratricopeptide containing protein RoXaN (Zc3h7b)
Intron number: 14
Mouse chromosome: 15
Upstream exon length: 101
Downstream exon length: 182
Mouse intron length: 965
Intron alignment length: 1254
Total murinae branch length: 0.16056
K_score: 0.07148
Scaling factor: 0.76817

ENSMUSG00000022390 exon 14 (ORF 0)

```
Mus     ATCTGCTTCGACAGCAAGCCCCGGATCATCAGCAAAGGGACCAAGGACTCTCCATCTGTCTGCTCCAACCTGGCTGCCAA 80
Rattus  ATCTGCTTCGACAGTAAGCCCCGGATCATCAGCAAAGGCACCAAGGACTCTCCGTCTGTCTGTTCCAACCTGGCTGCCAA 80
Cavia   ATTTGCTTTGACAGTAAGCCTCGGATCATCAGCAAAGGCACCAAGGACTCTCCGTCAGTCTGCTCCAACCTGGCTGCCAA 80
Homo    ATCTGCTTTGACAGTAAACCCCGGATCATCAGCAAAGGCACCAAGGACTCTCCGTCTGTCTGCTCCAACCTGGCTGCCAA 80

Mus     GCACAGCTTTTACAACAACAA 101
Rattus  GCACAGCTTTTACAACAACAA 101
Cavia   GCACAGCTTCTACAACAACAA 101
Homo    GCACAGCTTCTACAACAACAA 101
```

ENSMUSG00000022390 exon 15 (ORF 1)

```
Mus     GTGCCTGGTGCACATCGTCCGCTCCACCTCTCTCAAGTACTCCAAGATCCGTCAGTTCCAGGAGCACTTCCAGTTTGACG 80
Rattus  GTGCCTGGTGCACATCGTCCGCTCCACCTCTCTCAAGTACTCCAAGATCCGGCAGTTCCAGGAGCACTTCCAGTTTGACG 80
Cavia   GTGCCTGGTGCACATCGTCCGCTCCACCTCCCTCAAGTACTCCAAGATCCGCCAGTTCCAGGAGCACTTCCAGTTCGACG 80
Homo    GTGCCTGGTGCACATCGTCCGCTCCACCTCCCTCAAGTACTCCAAGATCCGCCAGTTCCAGGAGCACTTCCAGTTCGACG 80

Mus     TGTGCCGCCACGAGGTGCGCTATGGTTGCCTGCGCGAGGACAGCTGCCACTTCGCCCATAGTTTCATTGAGCTCAAGGTC 160
Rattus  TGTGCCGCCACGAGGTGCGCTATGGTTGCCTGCGCGAGGACAGCTGCCACTTCGCCCATAGTTTCATTGAACTCAAGGTC 160
Cavia   TCTGCCGCCACGAGGTACGCTACGGCTGCCTGCGGGAAGACAGCTGCCACTTCGCCCACAGTTTCATCGAGCTCAAAGTG 160
Homo    TGTGCCGCCATGAGGTGCGCTACGGCTGCCTGCGGGAGGACAGCTGCCACTTCGCCCACAGCTTCATCGAGCTCAAGGTC 160
```

ENSMUSG00000022390_intron_14

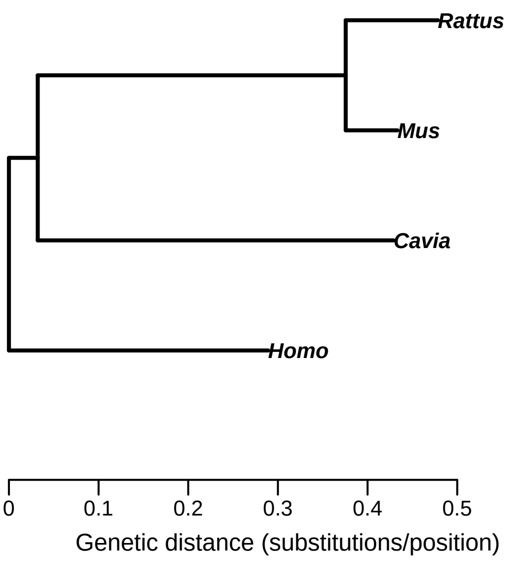

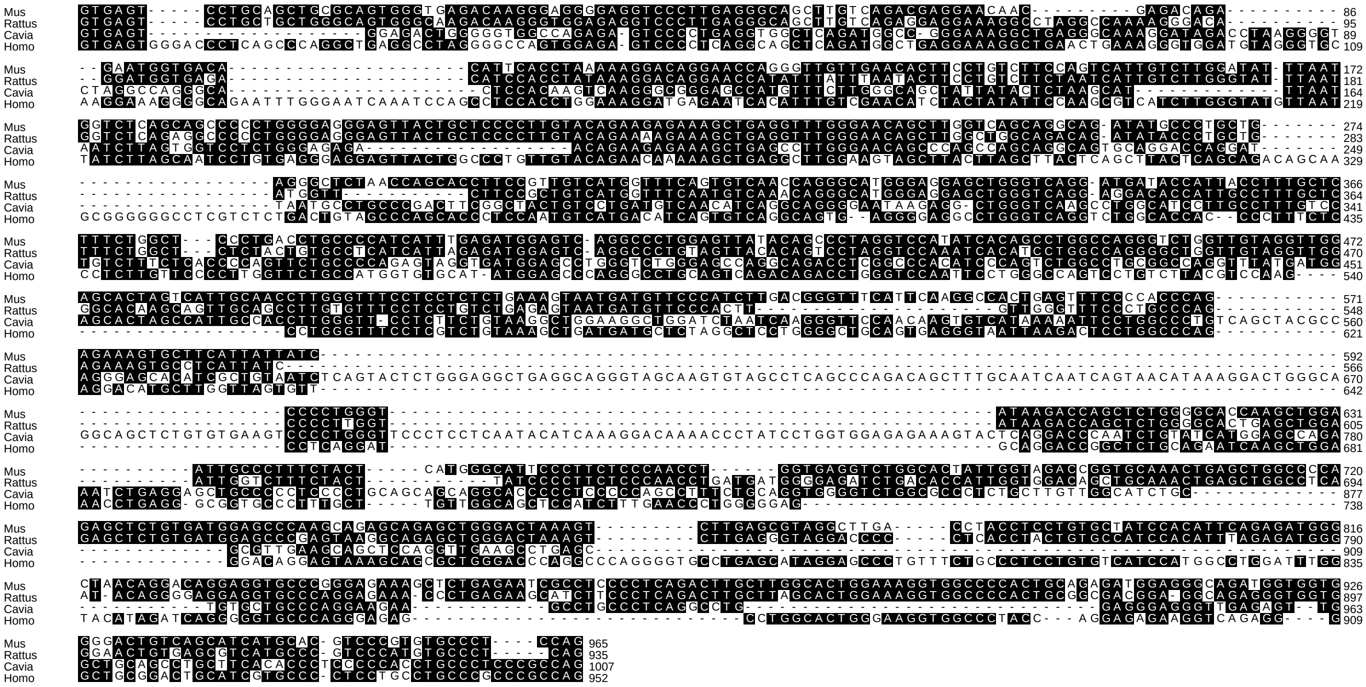

ENSMUSG00000046591 intron 3

Description: Uncharacterized protein C15orf42 homolog (5730590G19Rik)
Intron number: 3
Mouse chromosome: 7
Upstream exon length: 239
Downstream exon length: 235
Mouse intron length: 1549
Intron alignment length: 2119
Total murinae branch length: 0.18796
K_score: 0.07652
Scaling factor: 0.7687

ENSMUSG00000046591 exon 3 (ORF 2)

```
Mus     AATTGTCCTAAGAGGCTCAGTGGCACAGTGGTCTCTCCCAGTGAGTAGTGCTTTAGGCACTGACAGTTGGATGCTACAAA 80
Rattus  AATTTTCCTAAAAGGCTCAGTGACACAGTGGTCTCTCCCAATGAGCAGTGCTTTAGGCACTGACAACTGGATGCTACAAA 80
Cavia   AATTTTTTTAAAAGGCTCAGTGGCCCAGTGGTCTCTCCCGATGAGTGGCACTTTGGGCACCGACAGCTGGATGCTACAGA 80
Homo    AATTTTTCTAAAAGGCTCAGTGGCCCAGTGGTCTCTCCCAACGAGCAGCACTTTGGGCACTGACAGCTGGATGCTAGGAA 80

Mus     GTCCAGAGGAACACAGGTCAACTCAAAGGCTGTTGTTTCAGGAGCTGGTAAGCAGGCTGACTGCTGAAGAATTCCATCTG 160
Rattus  GTCCAGAGGAACACAAGTCAACTCAAAGGCTGCTGTTTCAGGAGCTGGTAAGCAGGCTGACTGCTGAAGAATTCCATCTG 160
Cavia   GTCCAGAGGAGAACAGACTGACTCAAGGGCTCTTATTTCAGCAGCTGGCAAGCAGTCTGATGGCTGAAAAGTTACATCTG 160
Homo    GTCCAGAGGAGAGCACAGCAACTCAAAGGCTGTTATTTCAGCAGTTGGTAAGCAGGCTGACTGCTGAAGAGTTACACCTG 160
```

ENSMUSG00000046591 exon 4 (ORF 0)

```
Mus     GTTGCCAGTGTGGATCCTGGTGAAGGCTGGCCCCCCATAACGGGAATTATTTCCCCATTCTCTGCCAATGCCATGATTCT 80
Rattus  GTGGCCAGTGTGGATCCTGGTGACGGCTGGCCCCCCATCACGGGAATTATCTCCCCATTCTCTGCCAATGCCATGATTCT 80
Cavia   GTTGCCAGTGTGGACCCGGGTGAAGGCTGGCCCCCTGTCACCGGAATCCTTTCCCCACTGTCTGCCAGTGCTATGATTCT 80
Homo    GTTGCTGATGTGGACCCTGGTGAAGGCCGGCCCCCCATCACTGGAGTTATTTCCCCACTCTCTGCCAGTGCTATGATCCT 80

Mus     CACAGTGTTCCGAGCCAAAGAAGCTGAATTTCAAAGCCATTTTCTCCAAACAGCTGCGACTGAAGGTTCCCAGGATACAG 160
Rattus  CACAGTGCTCCGAGCCAAAGAAGCTGAATTTCAAAGACATTTTCTCCAGACAGCTGTGACTGAAGGTTCCCAGGATGTAA 160
Cavia   CACTGTGTTCCACGTCAGGGAAGCTGAATTCCAAGGACATTTTCTCCAAACGGTCGCAGCCGAAGGCCCCCAGGATACAA 160
Homo    CACTGTGTGCCGCACCAAGGAGGCTGAATTTCAACGACATGTTCTCCAAACAGCTGTGGCTGACAGCCCCCGGGACACAG 160
```

ENSMUSG00000046591_intron_3

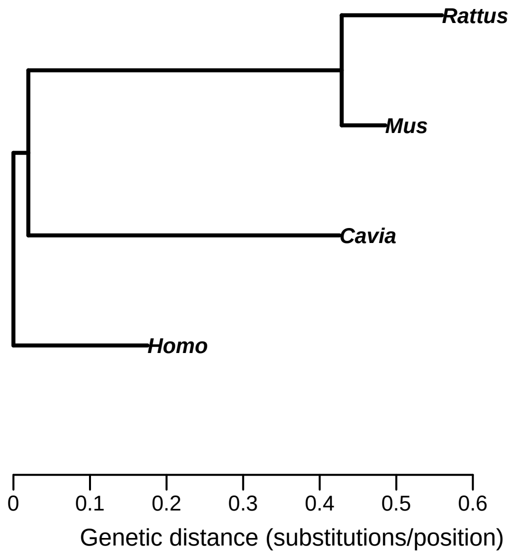

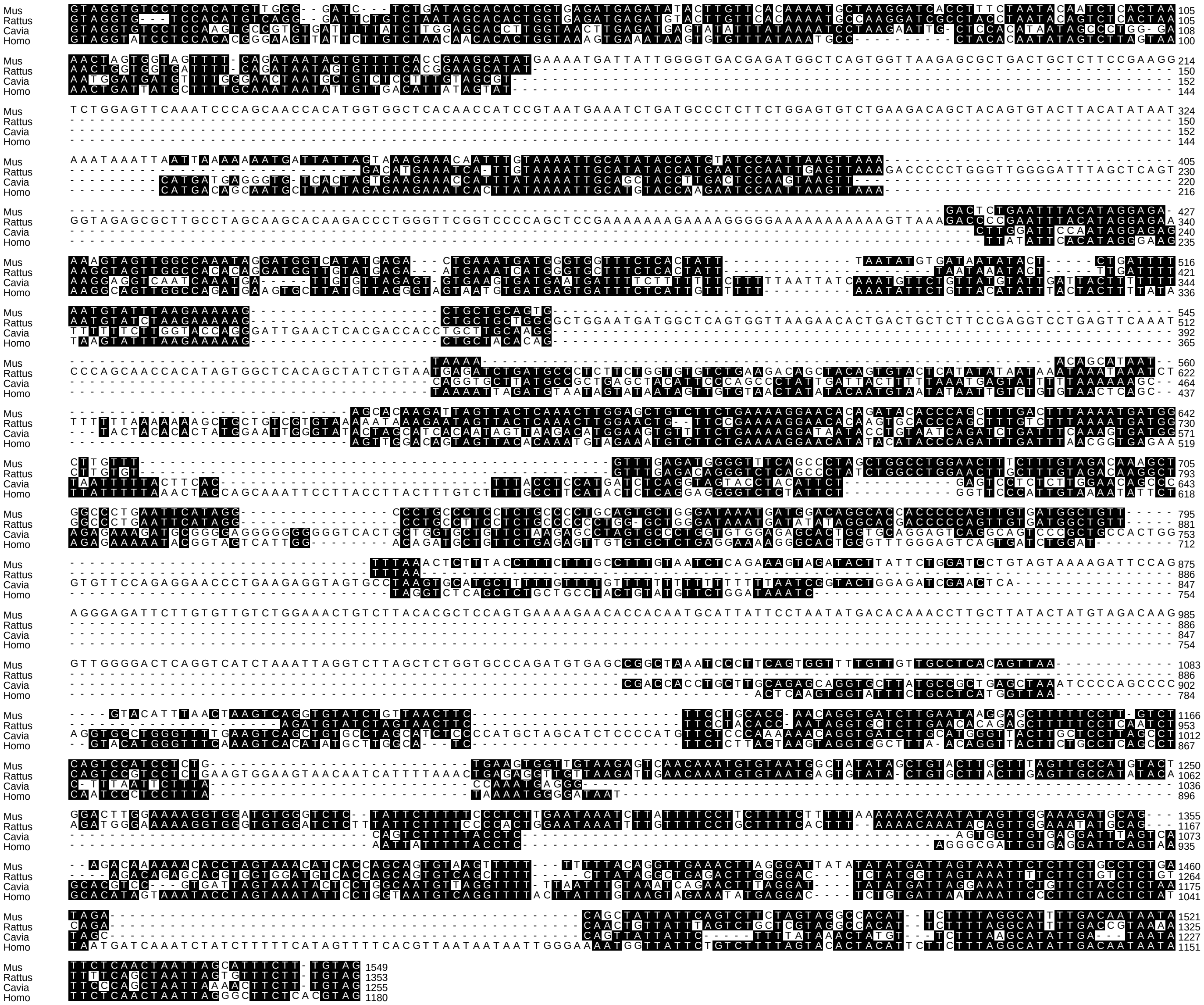

ENSMUSG00000059901 intron 13

Description: a disintegrin-like and metallopeptidase (Adamts14)
Intron number: 13
Mouse chromosome: 10
Upstream exon length: 134
Downstream exon length: 124
Mouse intron length: 282
Intron alignment length: 398
Total murinae branch length: 0.29097
K_score: 0.08672
Scaling factor: 0.76891

ENSMUSG00000059901 exon 13 (ORF 2)

```
Mus     ATGCCCAGAAGTGCGAGCTCATTTGCCAGTCTGCCGACACTGGAGATGTGGTATTTATGAACCAAGTAGTCCACGATGGG 80
Rattus  AGGCCCAGAAGTGCGAGCTCATTTGTCAGTCGGCAGACACCGGAGACGTGGTGTTTATGAACCAAGTGGTGCATGATGGG 80
Cavia   ACACCCAGAAGTGTGAGCTGATCTGCCAGTCAGCGGACACGGGCGAGGTGGTGTACATGAACCAAGTGGTCCACGATGGG 80
Homo    ACGCCCAGAAGTGTGAGCTGATCTGCCAGTCGGCGGACACGGGGGACGTGGTGTTCATGAACCAGGTGGTTCACGATGGG 80

Mus     ACACGCTGCAGCTATCGCGATCCTTACAGCGTCTGTGCCCGTGGCGAGTGTGTG 134
Rattus  ACGCGCTGCAGTTACCGAGATCCTTACAGTGTCTGTGCCCGTGGAGAGTGCGTG 134
Cavia   ACACGCTGCAGCTACCGGGACCCGTACAGTGTCTGTGCCCGTGGCGAGTGTGTG 134
Homo    ACACGCTGCAGCTACCGGGACCCATACAGCGTCTGTGCGCGTGGCGAGTGTGTG 134
```

ENSMUSG00000059901 exon 14 (ORF 0)

```
Mus     CCCTTTGGTTGCGACAAGGAGGTGGGATCCATGAAGACGGATGACAAGTGCGGTGTCTGCGGTGGGGACAATTCTCACTG 80
Rattus  CCCTTTGGTTGCGACAAGGAGGTGGGATCCATGAAGACGGATGACAAGTGCGGTGTCTGCGGTGGGGACAACTCTCACTG 80
Cavia   CCCTTTGGCTGTGACAAGGAGATGGGGTCCATGAAGGCAGATGACAAGTGCGGAGTTTGTGGCGGTGACAATTCCCACTG 80
Homo    CCTGTCGGCTGTGACAAGGAGGTGGGGTCCATGAAGGCGGATGACAAGTGTGGAGTCTGCGGGGGTGACAACTCCCACTG 80

Mus     TAGGACTGTGAAGGGGACTCTGGGAAAGGGCTCCAAGCAGGCAG 124
Rattus  CAGGACTGTGAAGGGGACACTGGGAAAGGCCTCCAAGCAAGCAG 124
Cavia   CAGGACCGTGAAGGGGACGCTGGGCAAAGCCTCCAAGCAGGCAG 124
Homo    CAGGACTGTGAAGGGGACGCTGGGCAAGGCCTCCAAGCAGGCAG 124
```

ENSMUSG00000059901_intron_13

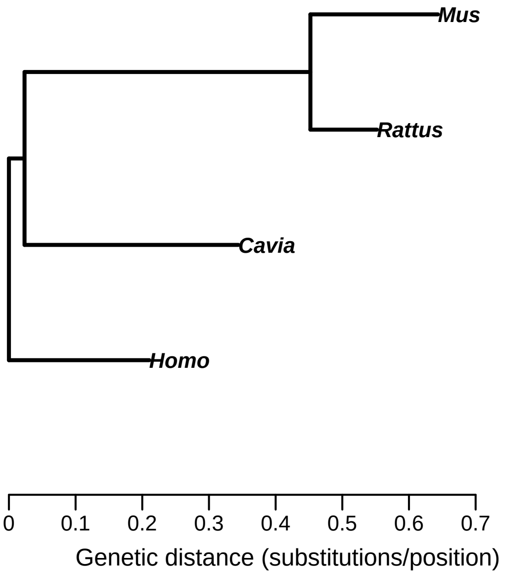

```
Mus     GTGGGTACCCCTGCGAGCTGCTTATACTCTGCTGGCATGGAGGACTGAATGGGTATTACTCACCTACC------------------------------------------ 68
Rattus  GTGGGTACCCCCGCAAGCTGCTCACGCTCTGCTGCCTCAGGGGCCTAGAGGGCTATGCTGCCATTTCTCGGGCCCTGGGATAAGGTATTGGCCACTAC------------ 98
Cavia   GTGGGTGTCATCCAAGG-----------------------------GACGCTCCACAGTGCCGTCCTCATGTTCCATAGGAGATAGCATGGCCATATCTGCTGCACTCAG 81
Homo    GTGGGTGCACCCCCAGCCACCCCGACTACCG--GCACAGGGAGACTGGAGGGCTGCACTGTAGCCCCTCCTGCCTGGGAAGATGAGCATTGCCTCACCGGCCCACTCGCA 108

Mus     -----------ACACCTAGGGGCAGCTAGGGCTGC----------------------CTACACCATTAGAGAAGTGAGGTGACCCAGGCTAAGA---------------- 129
Rattus  -----------CAACCTGAGAGCAGCTAGGGCTACTGCAGAGGGGTCCCTTCCTGTCCTACACCATTAGAGAAGCGAGGTGACCCAGGCTAAGA---------------- 181
Cavia   GAAGCCCCTGAGTACTCTGTATAGCCCTAGGCTGCAGGAAAGGGGTCACTTACTGCCCAGCCCCATT-CCCAGGAGAGGAGA---AGGCAAG------------------ 169
Homo    ACACCCTGTTGGTCCTCAGCATAGCCCTGGGGTGCTGGA-AGGGGCTAATTATTGCTCAGCCCCATTGGAGGGGAGAGGAGA---TGGCAAGAATCCTCATCACGCCGGG 214

Mus     ---------CCACCTGGGTCACAGGGTGGATTTG----------------GGGGAGTTCTCGTGTGCCAGGGTCCCCTCTTTGGGCAGGGCCAGGAATCTCAGGAGGAAG 214
Rattus  ---------CCACCTGGGCCACAGGGTAGATTTG----------------GGGGAGTTCTCATGCACCAGGGTTCCCCCTTCAGCCAGGACCAGG------AGGGAGCAG 260
Cavia   ------------TTTAGGTCCCATGGTGAATTCGAGGGAGGGTCCTGTCTTGGGAGGCCTTTTGGGCCAGGATTCCTCGCCAGGG-AGGGCCTGGGG----ACTGGGAAG 262
Homo    TGTAAGACCTCACCCAGGTCCCACGGTGAATTCGTGCCAGAATCCTGTCCAGGGACATCTCCCAGGCCAGAGGTCTTCTCCTGACCAGGGCCTGGGG----ATGGGAATG 320

Mus     TCAGTCGGGGAGCCTTTCTAGGTGGATACAGGAGAGCCTCCATCTGCTGATGTCACCTCCACCCCTAG 282
Rattus  TGGGCCAGAGAGT---------TGGAGGCAGGGGAGCCTCCTCCTGCTGACTTCGTCTCCACCCCCAG 319
Cavia   AGAGCCTGGGTGGGGT--------------------ACTCTTCCCATTCATCTCACCTCTG-CCTCAG 309
Homo    GGAGCCCCGGGGTGGC----GGTGGCGGCAGGGGAGCCTCCTCCCACTGACCTCACCTCTGGCCTCAG 384
```

ENSMUSG00000039849 intron 2

Description: Phosphorylated CTD-interacting factor 1 (Pcif1)
Intron number: 2
Mouse chromosome: 2
Upstream exon length: 125
Downstream exon length: 138
Mouse intron length: 929
Intron alignment length: 1770
Total murinae branch length: 0.22924
K_score: 0.05179
Scaling factor: 0.76961

ENSMUSG00000039849 exon 2 (ORF 2)

```
Mus     AGGAGCTGGTACATGCAGGCTGGGAGAAGTGCTGGAGCCGGAGGGAGAGCCGTCCCTACTACTTCAACCGATTCACCAAC 80
Rattus  AGGAACTGGTACATGCAGGCTGGGAGAAGTGCTGGAGCCGAAGGGAGAGCCGTCCCTACTACTTCAACCGATTCACCAAC 80
Cavia   AGGAGCTTGTGCACGCAGGCTGGGAGAAGTGCTGGAGCCGGAGGGAAAATCGTCCCTACTACTTCAACCGATTCACCAAT 80
Homo    AGGAGCTGGTGCATGCAGGCTGGGAGAAGTGCTGGAGCCGGAGGGAGAATCGTCCCTACTACTTCAACCGATTCACCAAC 80

Mus     CAGTCTCTGTGGGAGATGCCCGTGCTGGGTCAGCACGACGTGCTT 125
Rattus  CAGTCCCTGTGGGAGATGCCTGTGCTGGGTCAGCACGATGTGCTT 125
Cavia   CAGTCCCTGTGGGAGATGCCTGTGCTGGGGCAGCATGATGTGATT 125
Homo    CAGTCCCTGTGGGAGATGCCCGTGCTGGGGCAGCACGATGTGATT 125
```

ENSMUSG00000039849 exon 3 (ORF 0)

```
Mus     TCGGACCCTCTGGGGCTGAATGCAACCCCACTGCCCCAAGACTCAAGCTTGGTGGAAACGCCCCCAGTAGAGAACAAGTC 80
Rattus  TCGGACCCTTTGGGGCTAAATGCAACCCCACTGCCCCAAGACTCAACCTTAGTGGAAACTCCCCCAGTAGAGAACAAGCC 80
Cavia   TCGGACCCTTTGGGGCTGAATGCGACCCCACTGCCCCAAGACTCAAGCTTGGTGGAAACCCCCCTGGCTGAGAACAAGGC 80
Homo    TCGGACCCTTTGGGGCTGAATGCGACCCCACTGCCCCAAGACTCAAGCTTGGTGGAAACTCCCCCGGCTGAGAACAAGCC 80

Mus     CAGAAAGCGACAGCTCTCAGAGGAGCAGCCAAGTGGCAACGGAGTGAAGAAGCCCAAG 138
Rattus  CAGAAAGCGACAGCTCTCAGAAGAGCAGCCAAGTGGCAATGGAGTGAAGAAGCCCAAG 138
Cavia   CAGAAAGAAGCAGCTCTCGGAAGAGCAGCCAAGTGGCAATGGTGTAAAGAAGCCCAAG 138
Homo    CAGAAAGCGGCAGCTCTCGGAAGAGCAGCCAAGCGGCAATGGTGTGAAGAAGCCCAAG 138
```

ENSMUSG00000039849_intron_2

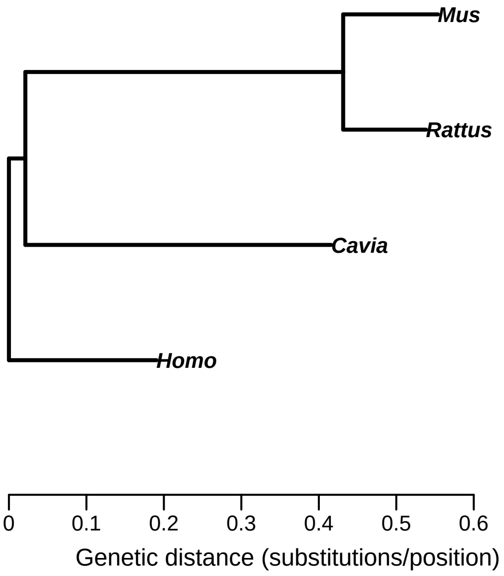

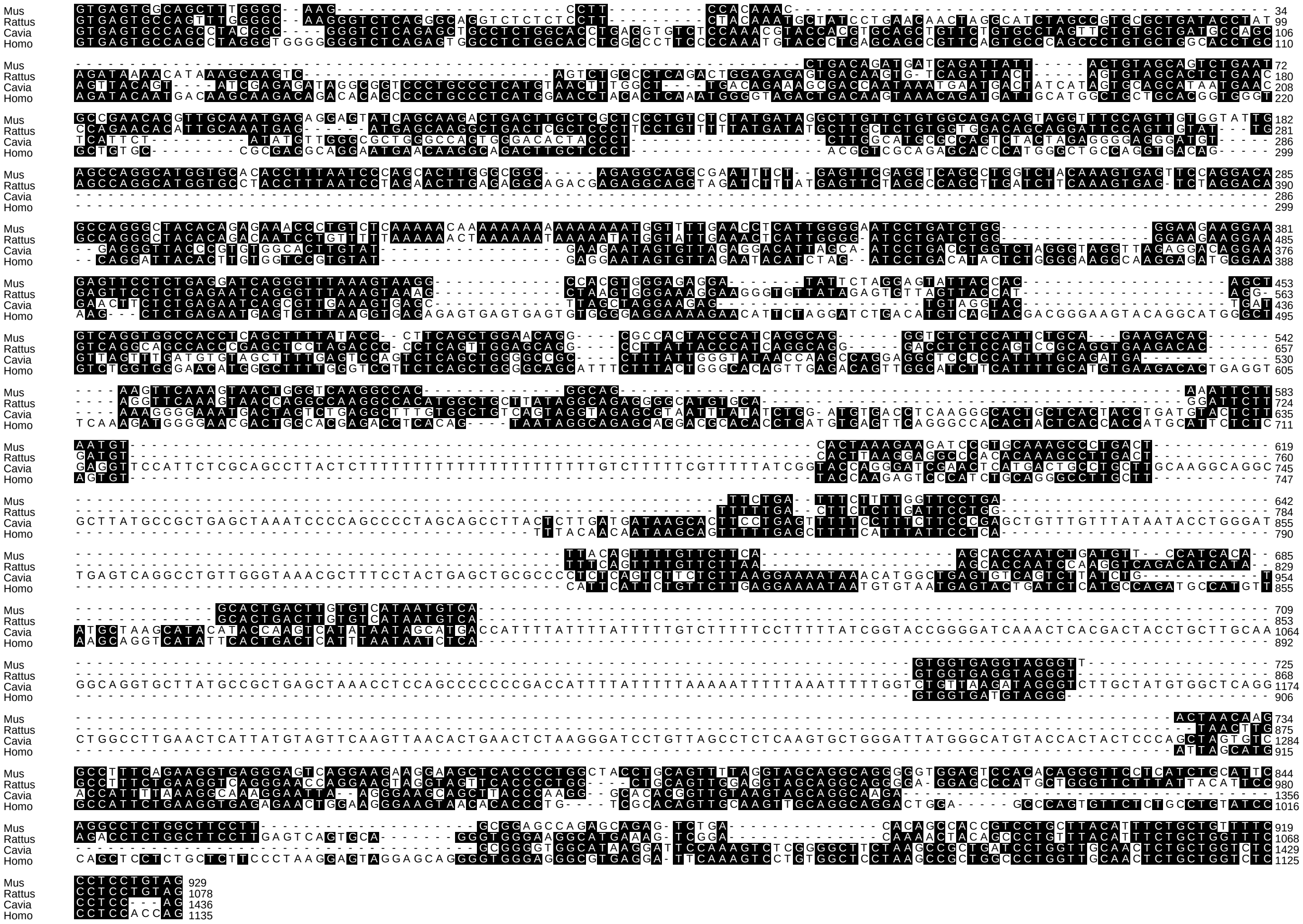

Mus
Rattus
Cavia
Homo

ENSMUSG00000021559 intron 9

Description: Death-associated protein kinase 1 (Dapk1)
Intron number: 9
Mouse chromosome: 13
Upstream exon length: 90
Downstream exon length: 93
Mouse intron length: 1156
Intron alignment length: 1078
Total murinae branch length: 0.14851
K_score: 0.04788
Scaling factor: 0.7698

ENSMUSG00000021559 exon 9 (ORF 0)

```
Mus     CCTAAAGACACCCAACAAGCACTTAGTCGAAAAGCCTCAGCAGTAAACATGGAGAAATTCAAGAAGTTTGCAGCTCGGAA 80
Rattus  CCTAAAGACACCCAACAAGCACTTAGTCGAAAAGCCTCAGCAGTAAACATGGAGAAATTCAAGAAGTTTGCAGCTCGGAA 80
Cavia   CCTAAAGATACGCAACAAGCACTTAGTAGAAAAGCATCAGCAGTAAATATGGAGAAATTCAAGAAGTTTGCAGCCCGGAA 80
Homo    CCTAAAGATACACAACAGGCACTTAGTAGAAAAGCATCAGCAGTAAACATGGAGAAATTCAAGAAGTTTGCAGCCCGGAA 80

Mus     AAAATGGAAA 90
Rattus  AAAATGGAAA 90
Cavia   AAAATGGAAA 90
Homo    AAAATGGAAA 90
```

ENSMUSG00000021559 exon 10 (ORF 0)

```
Mus     CAATCTGTTCGCTTGATATCACTGTGCCAAAGATTATCCAGGTCATTTTTGTCCAGAAGTAACATGAGTGTCGCCAGGAG 80
Rattus  CAATCTGTTCGCTTGATATCACTGTGCCAAAGATTATCCAGGTCATTTTTGTCCAGAAGTAACATGAGCGTTGCCAGGAG 80
Cavia   CAATCCGTTCGCTTGATATCACTGTGCCAAAGACTGTCTAGGTCATTCTTGTCCAGAAGTAACGTGAGTGTTGCCAGAAG 80
Homo    CAATCCGTTCGCTTGATATCACTGTGCCAAAGATTATCCAGGTCATTCCTGTCCAGAAGTAACATGAGTGTTGCCAGAAG 80

Mus     TGATGATACTCTG 93
Rattus  CGATGATACTCTG 93
Cavia   TGATGATACGCTG 93
Homo    CGATGATACTCTG 93
```

ENSMUSG00000021559_intron_9

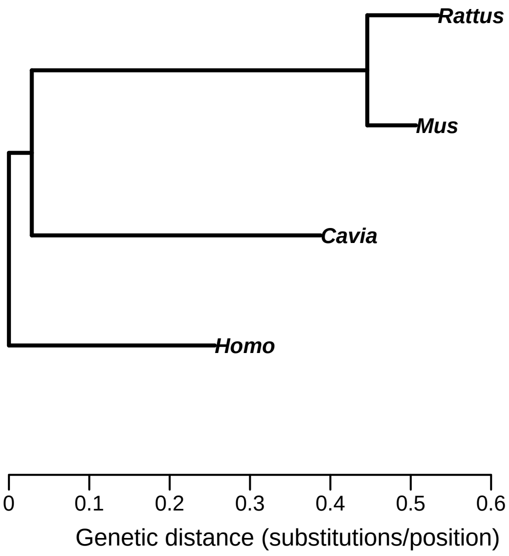

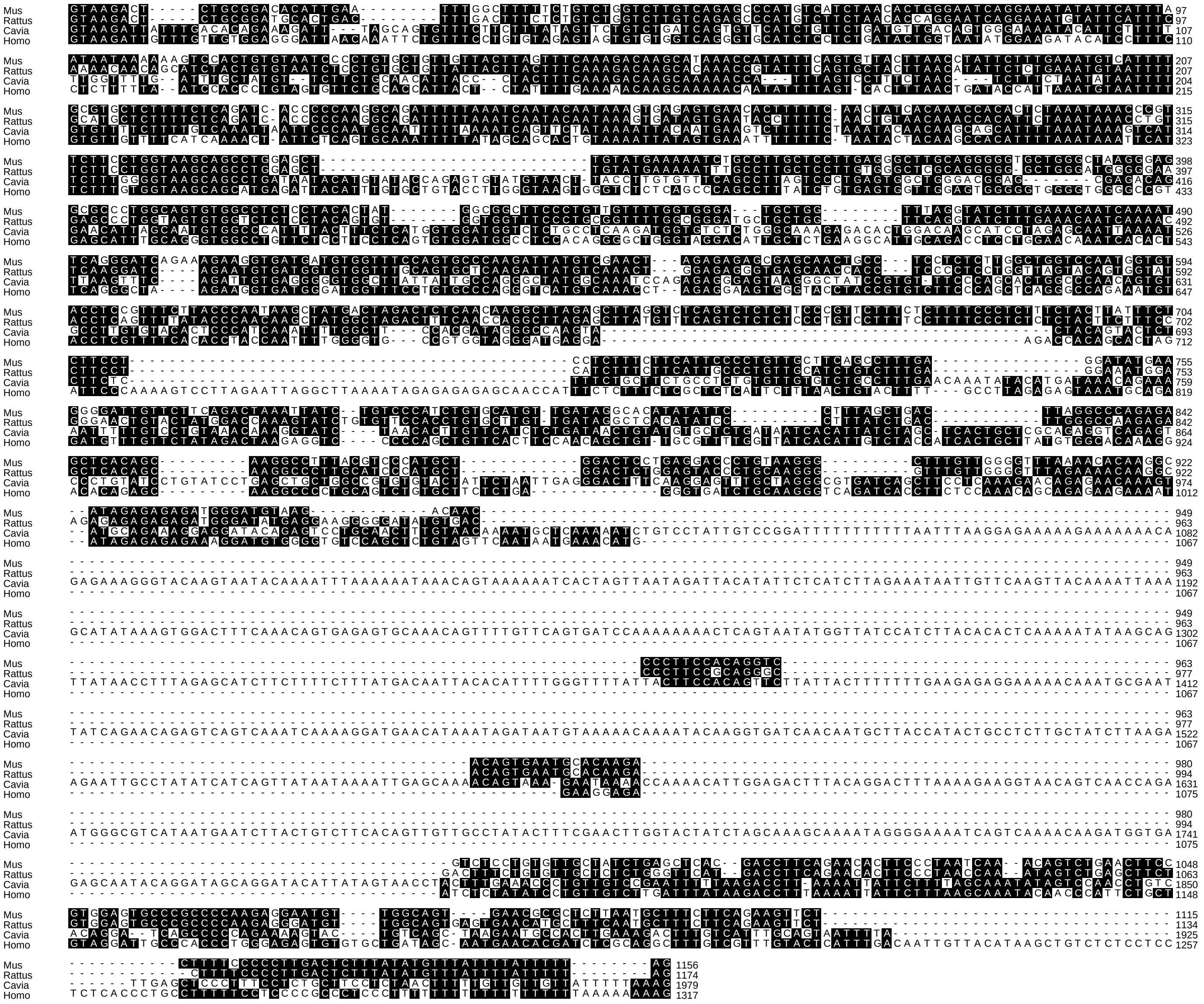

ENSMUSG00000032590 intron 12

Description: Acylamino-acid-releasing enzyme (Apeh)
Intron number: 12
Mouse chromosome: 9
Upstream exon length: 98
Downstream exon length: 52
Mouse intron length: 519
Intron alignment length: 786
Total murinae branch length: 0.20385
K_score: 0.08424
Scaling factor: 0.77024

ENSMUSG00000032590 exon 12 (ORF 2)

```
Mus     AGAGCTTCTCTGGGATCTACTGCAGCCTCCTTCCTTTGGGATGCTGGTCAGCTGACAGCCAGAGAGTGGTCTTCGACTCG 80
Rattus  AGAGCTTCTCTGGAATCTACTGCAGTCTTCTTCCTTTGGGATGCTGGTCAGCTGACAGCCAGAGAGTAGTCTTTGACTCT 80
Cavia   AGAGCTTCTCTGGAATTTACTGCAGCCTTCTGCCTCTGGGTTGCTGGTCAGCTGATAGCCAGAGAGTAGTCTTTGACTCT 80
Homo    AGAACTTCTCTGGGATCTACTGCAGCCTTCTGCCTTTGGGATGCTGGTCAGCTGACAGCCAGAGAGTGGTCTTTGACTCG 80

Mus     GTCCAGCGCAGTCGGCAG 98
Rattus  GCCCAGCGCAGTCGGCAG 98
Cavia   GCTCAACGGAGCCGGCAG 98
Homo    GCTCAGCGCAGCCGGCAG 98
```

ENSMUSG00000032590 exon 13 (ORF 0)

```
Mus     GACCTGTTTGCTGTGGACACCCAGACAGGAAGTGTCACCTCCCTGACAGCTG 52
Rattus  GACCTGTTTGCTGTGGACACCCAGACGGGCAGCATCACCTCCCTGACAGCTG 52
Cavia   GACCTGTTTGTAGTGGACACCCAGACGGGCAGTGTGACGTCCCTAACAGCTG 52
Homo    GACCTGTTTGCTGTGGACACCCAAGTGGGCACTGTGACCTCCCTCACAGCTG 52
```

ENSMUSG00000032590_intron_12

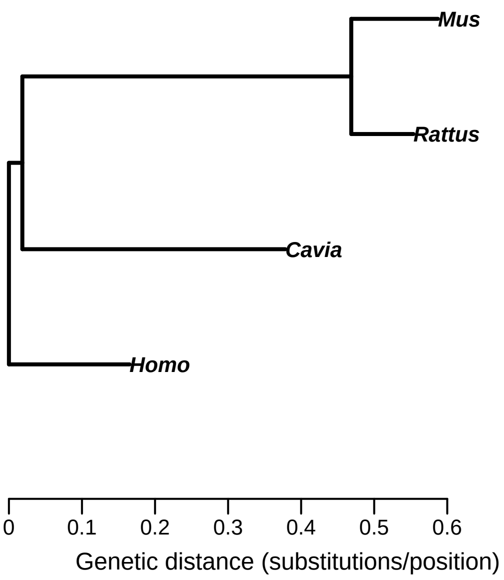

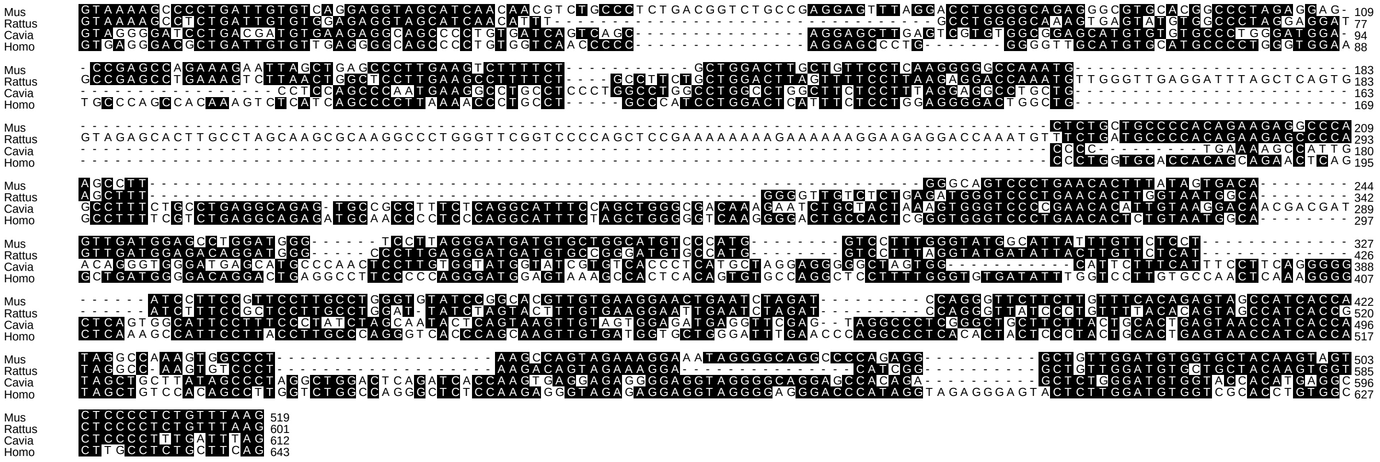
Mus
Rattus
Cavia
Homo

ENSMUSG00000025737 intron 1

Description: WD repeat-containing protein 24 (Wdr24)
Intron number: 1
Mouse chromosome: 17
Upstream exon length: 481
Downstream exon length: 178
Mouse intron length: 967
Intron alignment length: 1757
Total murinae branch length: 0.20569
K_score: 0.04738
Scaling factor: 0.77061

ENSMUSG00000025737 exon 1 (ORF 0)

```
Mus     TCCCGCAACAAGCAGGATCAGCTGTTCACAGAACACAAGCGCACAGTGAACAAAGTCTGTTTCCATCCCACCGAGGCCCA 80
Rattus  TCCCGCAACAAACAGGATCAGCTGTTCACAGAACACAAGCGCACAGTGAACAAAGTCTGTTTCCATCCCACTGAGGCCCA 80
Cavia   TCCCGTAACAAGCAGGACCAGCTGTTCACAGAACACAAACGCACTGTAAACAAAGTCTGCTTCCACCCCACTGAGGCCCA 80
Homo    TCCCGCAACAAGCAGGACCAGCTGTTCACAGAACACAAGCGCACGGTAAACAAAGTCTGCTTCCACCCCACCGAAGCCCA 80

Mus     TGTGCTGCTCAGTGGCTCTCAGGATGGCTTCATGAAGTGCTTTGACCTCCGAAGGAAGGACTCTGTCAGCACCTTCTCTG 160
Rattus  TGTGCTGCTCAGTGGCTCTCAAGATGGCTTTATGAAGTGCTTTGATCTCCGAAGGAAGGACTCTGTCAGCACCTTCTCTG 160
Cavia   TGTGCTGCTTAGTGGCTCACAAGATGGCTTCATGAAGTGCTTTGACCTCCGCAGGAAGGATTCTGTCAGCACTTTCTCAG 160
Homo    CGTGCTGCTCAGTGGCTCCCAGGATGGCTTCATGAAGTGCTTTGACCTCCGCAGAAAGGACTCTGTCAGCACCTTCTCGG 160
```

ENSMUSG00000025737 exon 2 (ORF 2)

```
Mus     GCCAGTCTGAGAGCGTGCGGGATGTGCAGTTCAGTATCCGTGACTACTTTACCTTTGCTTCCACCTTTGAGAATGGCAAC 80
Rattus  GCCAGTCTGAGAGCGTGCGGGACGTGCAGTTCAGTATTCGTGACTACTTTACCTTTGCTTCCACCTTTGAGAACGGCAAT 80
Cavia   GCCAATCTGAGAGTGTGCGAGATGTCCAGTTCAGTATCCGGGACTATTTCACATTTGCCTCTACCTTCGAGAATGGCAAC 80
Homo    GCCAGTCGGAGAGCGTGCGGGACGTGCAGTTCAGTATCCGGGACTACTTCACCTTCGCCTCCACCTTTGAGAACGGCAAT 80

Mus     GTACAGCTCTGGGACATCCGCCGACCCGACCGCTGTGAGAGGATGTTCACAGCCCACAATGGGCCTGTCTTCTGCTGTGA 160
Rattus  GTGCAGCTCTGGGACATCCGCCGTCCCGACCGGTGTGAAAGGATGTTCACAGCCCACAATGGGCCTGTCTTCTGCTGTGA 160
Cavia   GTGCAACTCTGGGACATCCGACGTCCTGACCGATGTGAGAGGATGTTCACAGCCCATAATGGGCCTGTCTTCTGCTGCGA 160
Homo    GTGCAGCTCTGGGACATCCGGCGTCCCGACCGGTGCGAGAGGATGTTCACAGCCCACAACGGACCCGTCTTCTGCTGCGA 160
```

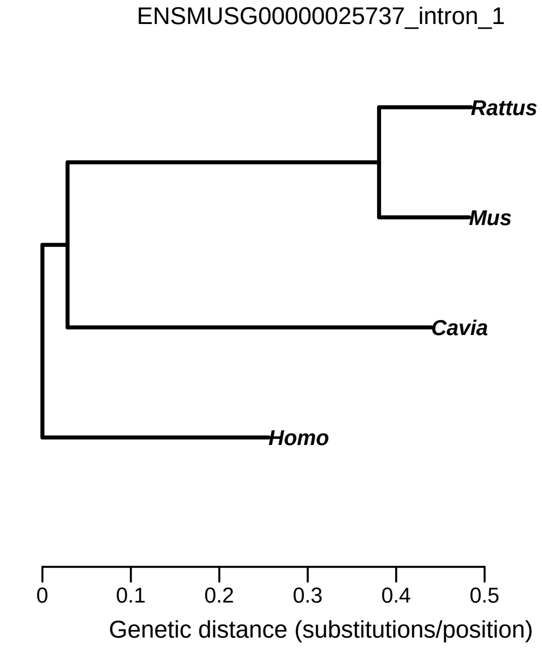

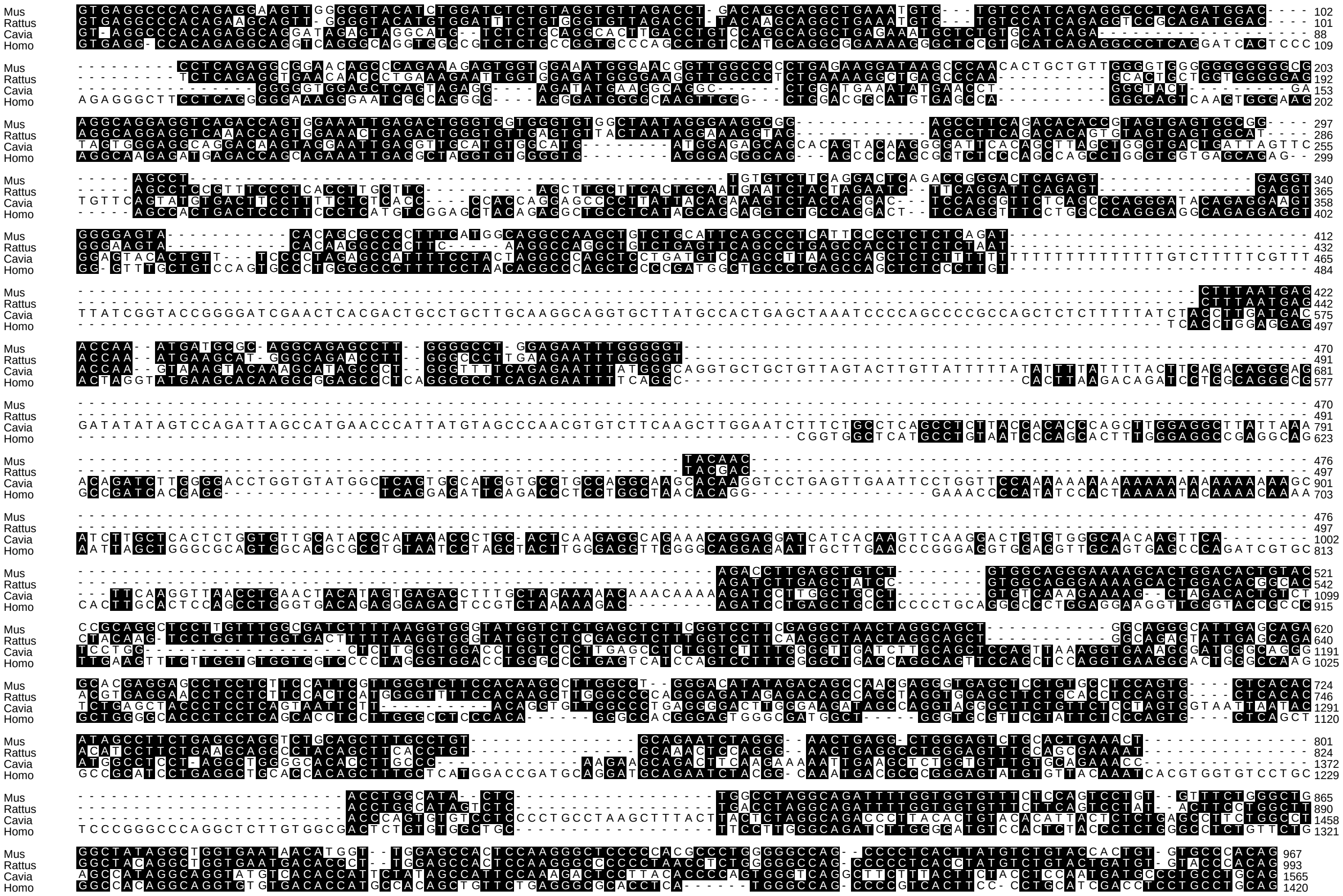
Mus
Rattus
Cavia
Homo

ENSMUSG00000030708 intron 5

Description: DnaJ homolog subfamily B member 13 (Dnajb13)
Intron number: 5
Mouse chromosome: 7
Upstream exon length: 114
Downstream exon length: 114
Mouse intron length: 1461
Intron alignment length: 2590
Total murinae branch length: 0.18730
K_score: 0.07453
Scaling factor: 0.77087

ENSMUSG00000030708 exon 5 (ORF 0)

```
Mus     GTACTAAATGAAGACAGATATTCTTCTACCATCAAGGACAAGATCCTGACCATCGATGTGAGACCTGGATGGAGGCAGGG 80
Rattus  GTGCTAAATGAAGATGGGTATTCCTCTACCATCAAGGACAAGATCCTGACCATCGATGTGAGGCCTGGGTGGAGGCAGGG 80
Cavia   GTGCTGAATGATGACGGGTACTCCTCTACCATCAAGGACAAGATCCTGACAATTGATGTGCGGCCTGGATGGAGGCAGGG 80
Homo    GTGCTGAACGAGGATGGGTACTCCTCCACCATCAAGGACAAGATCCTGACCATTGATGTGAAGCCCGGTTGGAGGCAGGG 80

Mus     CACACGCATCACCTTTGAGAAGGAAGGGGACCAG 114
Rattus  CACGCGCATCACCTTTGAGAAGGAAGGGGACCAG 114
Cavia   CACCCGCATCACCTTCGAGAAGGAGGGAGATCAG 114
Homo    CACACGCATCACCTTTGAGAAGGAAGGGGACCAG 114
```

ENSMUSG00000030708 exon 6 (ORF 0)

```
Mus     GGCCCCAACATCATCCCTGCTGACATTATCTTCATTGTGAAGGAGAAGCTGCACCCTCGCTTCCGCAGGGAGCATGACAA 80
Rattus  GGCCCCAACATTATCCCGGCCGATATTATCTTCATCGTAAAGGAGAAGCTGCACCCTCGCTTCCGCAGGGAGCAGGACAA 80
Cavia   GGCCCCAACATCATCCCAGCTGACATCATCTTCATTGTCAAGGAGAAGCTGCACCCTCGCTTCCGCAGGGAGAATGACAA 80
Homo    GGCCCCAACATCATCCCAGCAGACATCATTTTCATCGTAAAGGAGAAGCTACACCCTCGCTTCCGCAGGGAGAATGACAA 80

Mus     CCTCTTCTTCGTGTACCCCATTCCTTTGGGCAAG 114
Rattus  CCTCTTCTTTGTGTACCCCATTCCTTTGGGCAAG 114
Cavia   CCTCTTCTTTGTGAACTCCATCCCCTTGGGCAAG 114
Homo    CCTCTTCTTCGTGAACCCCATCCCTCTTGGCAAG 114
```

ENSMUSG00000030708_intron_5

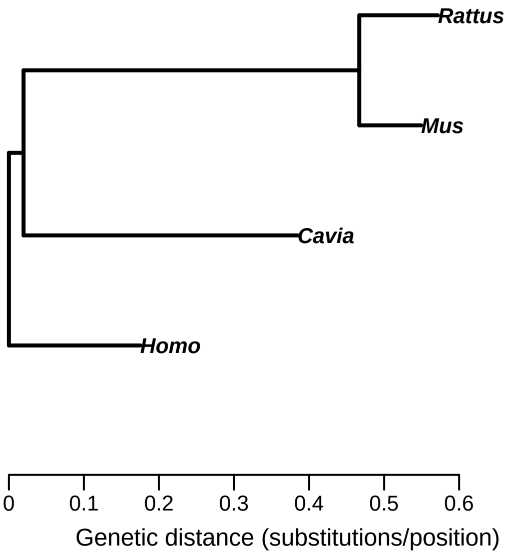

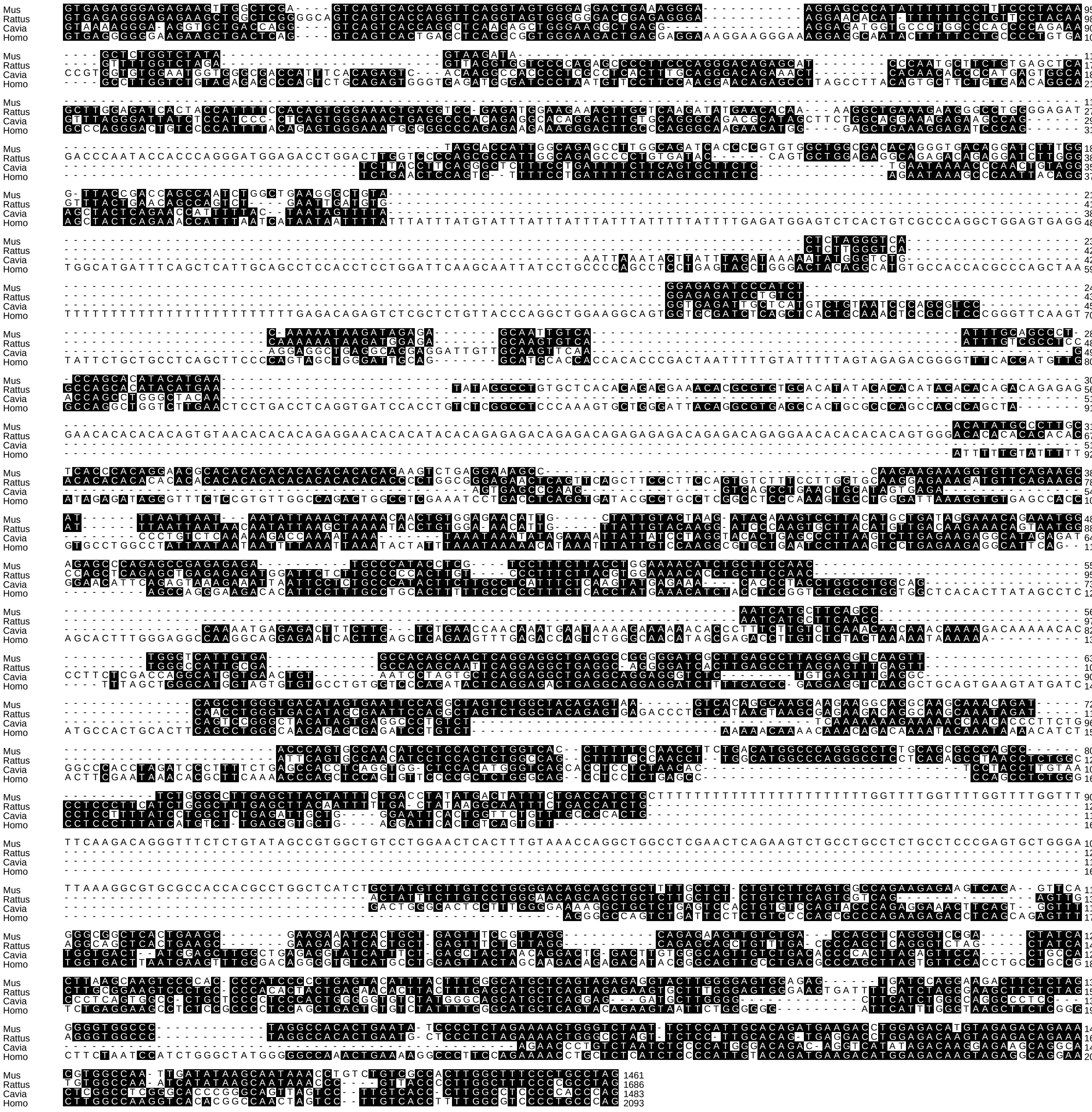

ENSMUSG00000042268 intron 10

Description: Solute carrier family 26 member 9 (Slc26a9)
Intron number: 10
Mouse chromosome: 1
Upstream exon length: 78
Downstream exon length: 96
Mouse intron length: 561
Intron alignment length: 634
Total murinae branch length: 0.17554
K_score: 0.03214
Scaling factor: 0.77113

ENSMUSG00000042268 exon 10 (ORF 0)

```
Mus     GTGGCCAGCTTGTGTGTGTCTCTGGTGGTGATGATCACCATGCTGGTCCTGGGATCCTATCTGTACCCTCTCCCCAAG 78
Rattus  GTGGCAAGCTTGTGTGTGTCTCTGGTGGTGATGATCACCATGTTGGTCCTGGGATCCTATCTGTACCCTCTCCCCAAG 78
Cavia   GTGGCAAGTCTGTGCGTGTCTCTGGTGGTGATGATCACCATGCTGGTCCTGGGATCCTATCTGTACCCTCTCCCTAAG 78
Homo    GTGGCCAGCCTGTGTGTGTCTCTGGTGGTGATGATCACCATGCTGGTCCTGGGGATCTATCTGTATCCTCTCCCTAAG 78
```

ENSMUSG00000042268 exon 11 (ORF 0)

```
Mus     GCTGTGCTAGGTGCCCTGATCGCTGTCAATCTCAAGAACTCCCTGAAGCAGCTCACTGACCCCTACTACCTCTGGAGGAA 80
Rattus  GCTGTACTAGGTGCCCTGATCGCTGTCAACCTCAAGAACTCCCTGAAGCAACTCACTGACCCCTACTACCTCTGGAGGAA 80
Cavia   GCTGTGCTGGGAGCCCTGATAGCTGTCAACCTCAAGAACTCCCTCAAGCAGCTCACTGACCCCTACTACCTGTGGAGGAA 80
Homo    TCTGTGCTAGGAGCCCTGATCGCTGTCAATCTCAAGAACTCCCTCAAGCAACTCACCGACCCCTACTACCTGTGGAGGAA 80

Mus     GAGTAAGCTAGACTGT 96
Rattus  GAGTAAGCTGGACTGC 96
Cavia   GAGCAAGCTGGACTGT 96
Homo    GAGCAAGCTGGACTGT 96
```

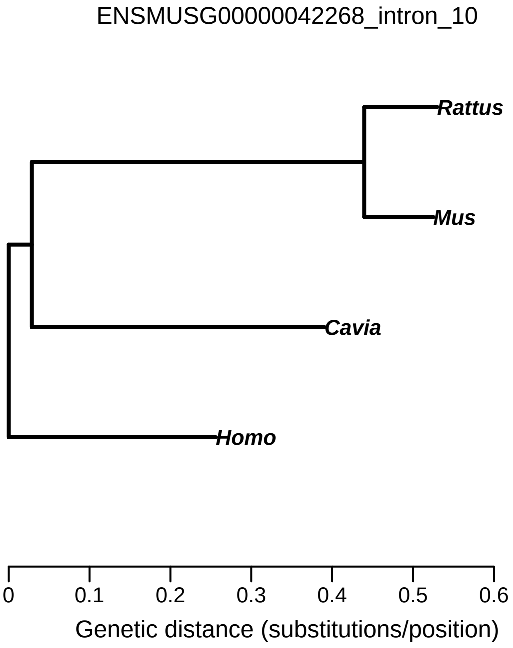

```
Mus     GTAAGAGCCCAACTGAG------------------GGGGAGACTGGGGGAACAGTGACCGCCTGCAGCAA-AACAATCCCTATGAGTGGGAGTAGCGCTTGGTTG----- 86
Rattus  GTAAGAGCCCAACTGAGAGCAGGAGGTAAGGGGATGGGGGAGGGGGGGGGGCAGTGACAGCCTGCAGCAA-AATAATTTCTATGAGTGGGAGTAACACTTGGTTG----- 104
Cavia   GTAAGAGCCCGTCTGGA------------------------AAGCAGAAGACTGTGGGAGACTCCAGTAGGAACCTTTTTGGGCAGT---------ACTTCGAAATGACC 77
Homo    GTAAGAGCCCAGCCATC------------------------GAGCAGAAGTCAACGAAAGACTCCAATAAGAACAATCCCTGAGAGTTGTGTGGCACTTTACGGACCACA 86

Mus     ----GCCATCACTGTCACCTTTGGTTTCCGTTAT--CCTACGAAGTAAATTATGTAAATTTTATTC-CTCCATTGTATGGGTGAGGGGGAGACTGAAGCTGACTGTCTGG 189
Rattus  ----GCCATCACTGTCACATTTGGTGTCTGTTAC--CCTACAAGGTAAATGATACAAACCTTATTC-CCCCATTGTACAGGTGA--GGGAGACTGAGGCTGGCTGGCTGA 205
Cavia   AAGTGATGTCATTATCACTCTTAGTCTTGGTCACAGCCTGTAAGGCACACAGGACAGGTTTTCTTCTCTCCATTTTGCAGGTAA--GAGAGGCTGAGC------------ 173
Homo    AAGTGCCACTGTTGTCACACTTAGTCTCAACCACAAACTGTGAGGTAGACAATGCAGGTTTTATCCTCCCCATTTTACAGGTGA--AGGAAACTGAGTCTGAGAGTCTAA 194

Mus     CTTGT--------CGGTGAGACGGCTGCTGGGTA--------------GTGTGTTACTGATTCCAACCTCCGGCTTTCTGG-----GAGTTTTATTCCTAGGCAGGAGAA 272
Rattus  CTTGT--------CAGTGAGACAGCTGCTGTGTAGGATTGATTTTACTGTATCTGACTAATGTCAACCTCCGGCTTTCTGG-----GAGTTTTATCCCCAGGCTGGAGAA 302
Cavia   -------------TGGTGAAACAGTT------------------CACAGTCCAGGGCTGGTTTCAGACTCCTTCCTTCTAGCCCTAGAGGCTGACCTGCAGGC------- 245
Homo    GTAACCTTGTCCATAGTGAGGCAGCT------------------TACAGCGCAGGGCTGGTCCCAAACTCCAGCCTTCTGGCCTCAGAGTCTAATCCCTAGGC------- 279

Mus     TAGTGGGCAAGG-TATCTGGTATTTCAGGGCACCCTCAGAGCCTCTCACAAAGATGTTAAGGTGTGAGCCATCCAAGGATGCAGGGAGAGGAGAAATGTAAAACTGGGCC 381
Rattus  TAGAGGGCAAGGTTATCTGGTACTTCAGGATCCCCTCAGAGCCTCTCACAATGATGTTAAGGTATGAGCCATTCAGGGGTACAGGAAGAGGGGAAATGTAAAACTGGGCC 412
Cavia   -----------------------------ACTATGGGAGATCTCAGTTGGGAGG-CTTCAAGCATGGGCTGCATAAGGAGGAGGGGAGAGAGGAAGGACAAAGATAAATC 325
Homo    -AACATTTGCACCTACCCACGAGTACCAGGCTCTTATATAGCCCAGCTAGGAGGGCTCTAGGCATGCGTCATTTAGAGATGAGGGAAGAGAGATAGGGAAAGGATGGGGC 388

Mus     CAGAACGAAGTGTGTGGCTCCAGAGGCTACACTTGCCAATCCTCAGATTGA--GGAGGAATT------------TGGGGAAAGAATTCTGGAACTTTTGCCTTGTCCTGC 477
Rattus  CAGAATGAAGTATATGGCTCAAGAGGCTACATTC-TCATTCCTGAGACTGA--GGAGGAGTT------------TGGAGGAAAAG-------------GCCTTGTCCTGC 494
Cavia   ----------------------------------------CCACAGCTTGAGTGGAGAGATTTGAGGGCTCTG-TAGGGGATGCATTGTGGGGCTCCAGCTCTGTCCTGC 394
Homo    CAGGAAGGACCCCATGGCTCTAACGCCAGCACTTTCCAAACCTAAGGTCGAATGCAGAGATTTGGGGGATCAGCCAGGGGAGGTGTTCCAGAACTCCGTCTCTGTCCTGC 498

Mus     CAGACTCCAGGGTCAGGCATGTGTAGGAAGGCCAGGGTGATGGGGATATCTCAAATGACACGTGGCAATGTTCTGCTTCTCCAG 561
Rattus  CTGACTCCAGGGTCAGACATGTGTAGGAAGGCCAGGATGAAGGGGATACCTCAAATGACACCAAGCAATGTTCTACTTCTCCAG 578
Cavia   CAGGCTGCAGGGTCAGGTGTGTGGGAGAAGGGCAGG-TGAATGGGCTACTCCAGCCGATGCCAAGGGGTGCTCTGATTCTCCAG 477
Homo    CAGGCCTTGGGGTCGGGTATGCGCAGGAGGGCAAAA-AGAAGGGGAGACCCTGGG-GTCCTGGAGCAATGTTCTGCTTCTCTAG 580
```

ENSMUSG00000028381 intron 7

Description: Ceramide glucosyltransferase (Ugcg)
Intron number: 7
Mouse chromosome: 4
Upstream exon length: 87
Downstream exon length: 190
Mouse intron length: 907
Intron alignment length: 1114
Total murinae branch length: 0.24108
K_score: 0.03288
Scaling factor: 0.77181

ENSMUSG00000028381 exon 7 (ORF 1)

```
Mus     AGGTTGGAGGTTTTCAATGTCTACTCAAGTTGCCATGCAAAACTCTGGTTCGTACTCAATTTCTCAGTTTCAATCCAGAA 80
Rattus  AGGCTGGAAGTTTTCAATGTCTACGCAAGTTGCCATGCAAAACTCTGGTTCATACTCCATTTCTCAGTTTCAATCCAGAA 80
Cavia   AGGTTGGAGGTTTGCAATGTCCACTCAAGTTGCAATGCAAAACTCTGGTTCATACTCAATTTCTCAGTTTCAATCCAGAA 80
Homo    AGGTTGGAGGTTTGCAATGTCCACTCAAGTTGCAATGCAAAACTCTGGCTCATATTCAATTTCTCAGTTTCAATCCAGAA 80

Mus     TGATCAG 87
Rattus  TGATCAG 87
Cavia   TGATCAG 87
Homo    TGATCAG 87
```

ENSMUSG00000028381 exon 8 (ORF 1)

```
Mus     GTGGACCAAATTGAGAATTAACATGCTTCCTGCTACAATAATTTGTGAGCCAATTTCAGAATGCTTTGTTGCCAGTTTAA 80
Rattus  GTGGACCAAATTGAGGATTAACATGCTTCCTGCCACAATAATTTGTGAGCCAATTTCAGAATGCTTTGTTGCCAGTTTAA 80
Cavia   GTGGACCAAATTGCGAATTAACATGCTTCCCGCTACAATCATTTGTGAGCCAATTTCAGAATGTTTTGTTGCCAGTCTGA 80
Homo    GTGGACCAAACTACGAATTAACATGCTTCCTGCTACAATAATTTGTGAGCCAATTTCAGAATGCTTTGTTGCCAGTTTAA 80

Mus     TTATTGGGTGGGCAGCCCACCATGTATTCAGATGGGATATCATGGTCTTCTTCATGTGCCACTGCCTGGCATGGTTTATA 160
Rattus  TTATCGGGTGGGCAGCCCACCACGTGTTCCGATGGGATATCATGGTTTTCTTCATGTGTCACTGCCTGGCATGGTTTATA 160
Cavia   TTATTGGATGGGCAGCCCACCACGTATTCAGATGGGATATCATGGTATTTTTCATGTGTCATTGCCTGGCGTGGTTTATA 160
Homo    TTATTGGATGGGCAGCCCACCATGTGTTCAGATGGGATATTATGGTATTTTTCATGTGTCATTGCCTGGCATGGTTTATA 160
```

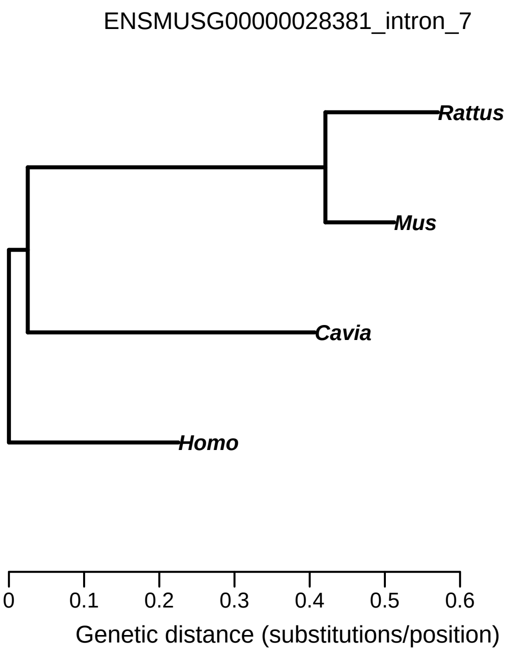

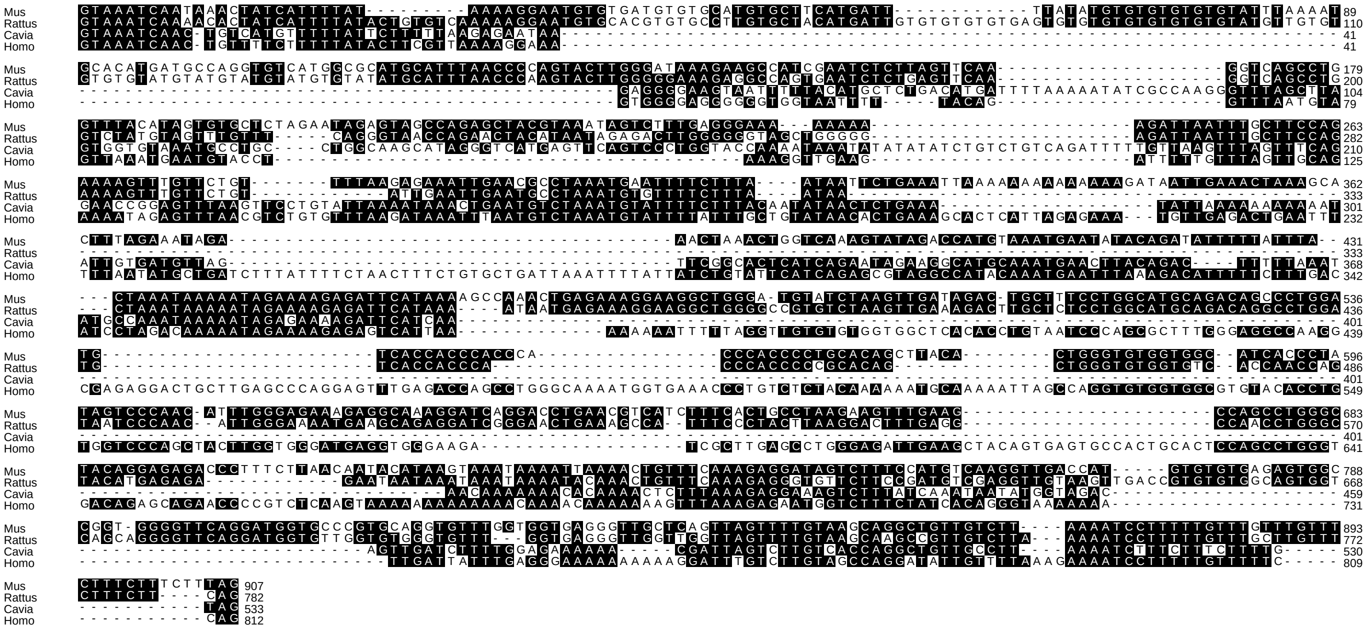
Mus
Rattus
Cavia
Homo

ENSMUSG00000032122 intron 12

Description: Sugar phosphate exchanger 2 (Slc37a2)
Intron number: 12
Mouse chromosome: 9
Upstream exon length: 86
Downstream exon length: 49
Mouse intron length: 643
Intron alignment length: 471
Total murinae branch length: 0.21758
K_score: 0.03963
Scaling factor: 0.77212

ENSMUSG00000032122 exon 12 (ORF 2)

```
Mus     GTGGCATCATGGCAGGGCTCATCAGCGACTACACCAATAGCAGGGCCACCACTTGCTGCATCATGCTGATCTTGGCTGCT 80
Rattus  GTGGCATCATGGCAGGGCTCATCAGCGACTACACCAATGGCAGGGCCACCACTTGCTGCGTCATGCTGATCTTGGCTGCT 80
Cavia   GTGGCATCCTGGCAGGGCTCATCTCAGACTACATCAATGGCAGGGCCACCACTTGCTGCGTCATGCTGATCTTGGCTGCC 80
Homo    GCGGCATCGTGGCAGGGCTCGTCTCTGACTACACCAATGGCAGGGCCACCACTTGCTGTGTCATGCTCATCTTGGCTGCC 80

Mus     CCCATG 86
Rattus  CCCATG 86
Cavia   CCGGTG 86
Homo    CCCATG 86
```

ENSMUSG00000032122 exon 13 (ORF 0)

```
Mus     ATGTTCCTGTACAACTACATTGGCCAGAATGGGATAACCAGTTCCATAG 49
Rattus  ATGTTCCTGTACAACTACATTGGCCAGAATGGGATAACCAGCTCCATAG 49
Cavia   ATGTTTCTGTACAATTCTGTTGGCCAGAACGGGATCACCAGCTCCATAG 49
Homo    ATGTTCCTGTACAACTACATTGGCCAGGACGGGATTGCCAGCTCCATAG 49
```

ENSMUSG00000032122_intron_12

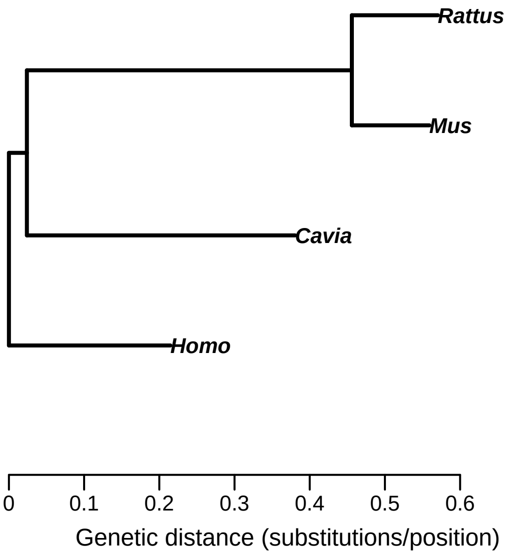

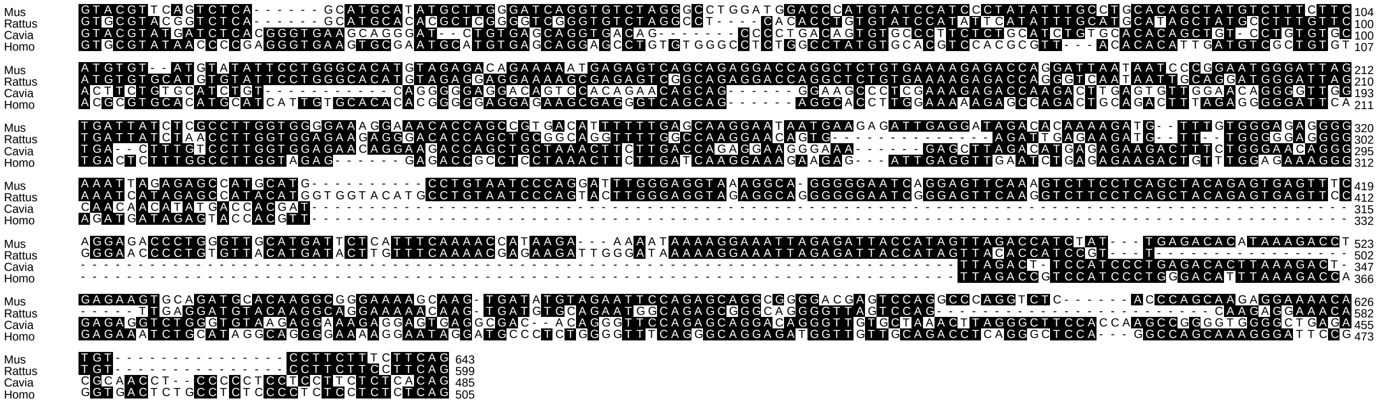
Mus 104
Rattus 100
Cavia 100
Homo 107
Mus 212
Rattus 210
Cavia 193
Homo 211
Mus 320
Rattus 302
Cavia 295
Homo 312
Mus 419
Rattus 412
Cavia 315
Homo 332
Mus 523
Rattus 502
Cavia 347
Homo 366
Mus 626
Rattus 582
Cavia 455
Homo 473
Mus 643
Rattus 599
Cavia 485
Homo 505

ENSMUSG00000051235 intron 1

Description: Flap endonuclease GEN homolog 1 (Gen1)
Intron number: 1
Mouse chromosome: 12
Upstream exon length: 161
Downstream exon length: 187
Mouse intron length: 1338
Intron alignment length: 1526
Total murinae branch length: 0.13348
K_score: 0.08029
Scaling factor: 0.7727

ENSMUSG00000051235 exon 1 (ORF 0)

```
Mus     TGGGAGTGAATGACTTATGGCAAATTCTAGAGCCTGTAAAGCAACATATCCACTTGCAAGATCTTAGTGGGAAAACCATT 80
Rattus  TGGGAGTGAATGACCTGTGGCAAATTTTGGAGCCTGTAAAGCAACATATCCACTTGCAGGATCTTTGTGGGAAGACCATT 80
Cavia   TGGGAGTGAATGACTTGTGGCAGATTTTGGAGCCTGCTAAGCAACACATCCACTTACAGAATCTCAGTGGGAAAACCATC 80
Homo    TGGGAGTGAATGACTTGTGGCAAATTTTGGAGCCTGTTAAGCAACACATCCCCTTGCGTAATCTTGGTGGGAAAACCATT 80

Mus     GCCGTTGATTTGAGTCTCTGGGTATGCGAGGCACAGACAGTGAAGAAAATGATAGGAACTGTCAAGAAGCCCCACCTCAG 160
Rattus  GCCGTTGATTTGAGTCTCTGGGTATGCGAGGCACAGACAGTGAAGAAAATGATTGGAACTGTCATGAAGCCCCACCTCAG 160
Cavia   GCAGTTGATCTGAGTCTCTGGGTGTGTGAGGCACAGTCTGTCAGAAAGATGATGGGAACTGTGAGGAAGCCTCACCTCAG 160
Homo    GCAGTTGATCTGAGTCTCTGGGTGTGTGAGGCACAGACAGTCAAAAAAATGATGGGCAGCGTCATGAAGCCCCACCTCAG 160
```

ENSMUSG00000051235 exon 2 (ORF 1)

```
Mus     GAACCTATTTTTTTCGTATTTCATATTTAACTCAAATGAATGTAAAACTGGTGTTTGTAATGGAAGGGGAGCCACCAATGC 80
Rattus  GAACCTATTTTTTTCGTATTTCATATTTAACTCAAATGAATGTAAAACTGGTGTTTGTGATGGAAGGGGAGCCACCAAAGC 80
Cavia   GAACTTATTTTTTTCGTATCTCATATTTAATGCAAATGGATATCAAACTGGTGTTTGTTATGGAAGGGGAACCACCAAAGC 80
Homo    GAACTTATTTTTTTCGTATCTCATATTTAACACAAATGGATGTAAAACTGGTATTTGTTATGGAAGGGGAACCACCAAAGC 80

Mus     TGAAAGCTGATGTCATAAGCAAGAGGACTCAGACTCGTTACGGGCCTTCTGGAAAATCAAGGTCTCAGAAAACAGGGAGA 160
Rattus  TGAAAGCTGATGTCATGAACAAGAGGACTCAGACTCGATACGGGCCTTCTGGAAAATCGAGGTCTCAGAAAACAGGGAGA 160
Cavia   TGAAAGCTGATGTCATGAACAAGAGGAATCAGATTCGGTATGGGCTTTCTGGAAAAACATGGTCTCAGAAATCAGGAAGA 160
Homo    TGAAAGCTGATGTCATAAGCAAGAGGAATCAGTCTCGGTATGGGTCTTCTGGAAAATCGTGGTCTCAGAAAACAGGGAGA 160
```

ENSMUSG00000051235_intron_1

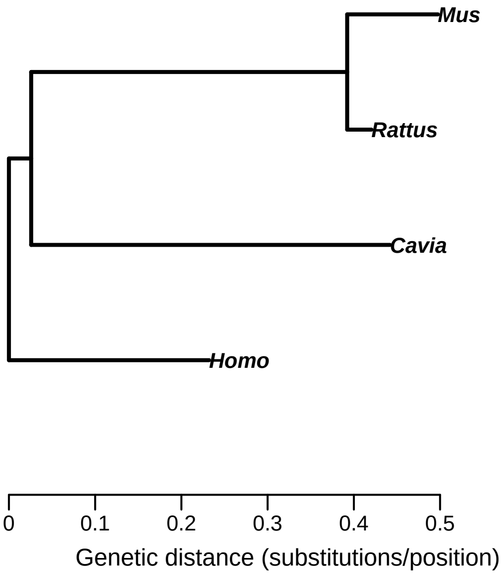

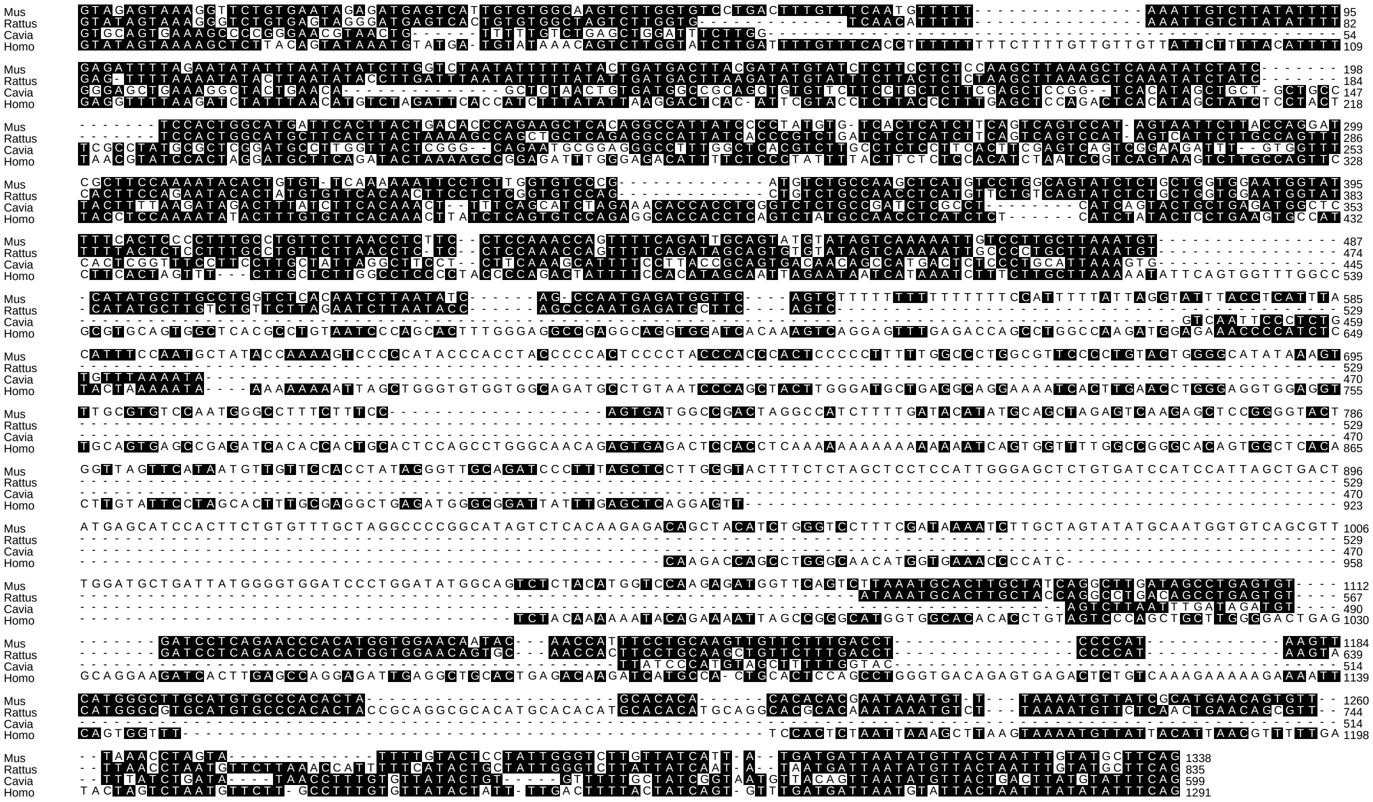
Mus
Rattus
Cavia
Homo

ENSMUSG00000027375 intron 2

Description: Myelin and lymphocyte protein (Mal)
Intron number: 2
Mouse chromosome: 2
Upstream exon length: 168
Downstream exon length: 126
Mouse intron length: 1506
Intron alignment length: 1909
Total murinae branch length: 0.13549
K_score: 0.06579
Scaling factor: 0.77313

ENSMUSG00000027375 exon 2 (ORF 0)

```
Mus     AGGCCTGGTGTGGATCCTGATTGCCTCCTCCCTGGTACCCTTGCCCCTGGCCCAGGGCTGGGTGATGTTTGTGTCTGTGT 80
Rattus  AGGCCTGGTGTGGATCCTGATCGCCTCCTCCCTGGTACCCATGCCCCTGGTCCAGGGCTGGGTGATGTTTGTGTCTGTGT 80
Cavia   GGGCCTGGTGTGGATCCTGGGTGGCCGCCTCCCGGGTGCCCATCCCCCTGATCCAGGGCTGGGTGATGTTCGTGTCTGTGT 80
Homo    GGGCCTGGTGTGGATCCTGGTGGCCTCCTCCCTGGTGCCCTGGCCCCTGGTCCAGGGCTGGGTGATGTTCGTGTCTGTGT 80

Mus     TCTGCTTTGTGGCCACCACTTCCCTGATGATCTTGTACATAATTGGTACTCATGGCGGTGAGACTTCCTGGATCACACTG 160
Rattus  TCTGCTTCCTGGCCACCACTTCCCTGATGGTCATGTACATAATTGGTACTCATGGTGGGGAGACTTCCTGGATCACACTG 160
Cavia   TCTGCTTCGTGGCCACCACGGCCCTGCTCATCCTGTACATGATTAATGCCCACGGCGGGGAGACATCCTGGGTCACGCTG 160
Homo    TCTGCTTCGTGGCCACCACCACCTTGATCATCCTGTACATAATTGGAGCCCACGGTGGAGAGACTTCCTGGGTCACCTTG 160
```

ENSMUSG00000027375 exon 3 (ORF 0)

```
Mus     GATGCAGCCTACCACTGTGTGGCTGCCCTATTTTACCTCAGTGCCTCAGTTCTGGAAGCCCTGGCCACCATCTCAATGTT 80
Rattus  GATGCAGCCTACCACTGTGTGGCTGCCCTGTTTTACCTCAGTGCCTCAGTCCTGGAAGCCCTGGCCACCATCACAATGTT 80
Cavia   GACGCAGCCTACAACTGTGTGGCTGCCCTGCTTTACCTCAGCGCCTCGGTCCTGGAAGCCTTGGCCACCATCCTCACGCG 80
Homo    GACGCAGCCTACCACTGCACCGCTGCCCTCTTTTACCTCAGCGCCTCAGTCCTGGAGGCCCTGGCCACCATCACGATGCA 80

Mus     TGATGGCTTTACTTACAAGCATTACCATGAAAACATCGCCGCAGTG 126
Rattus  CGATGGCTTTACCTACAGGCATTACCATGAGAACATCGCTGCAGTG 126
Cavia   AGATGGTTACACCTACAAGCAGTACCACGAAAACATCTCTGCTGTG 126
Homo    AGACGGCTTCACCTACAGGCACTACCATGAAAACATTGCTGCCGTG 126
```

ENSMUSG00000027375_intron_2

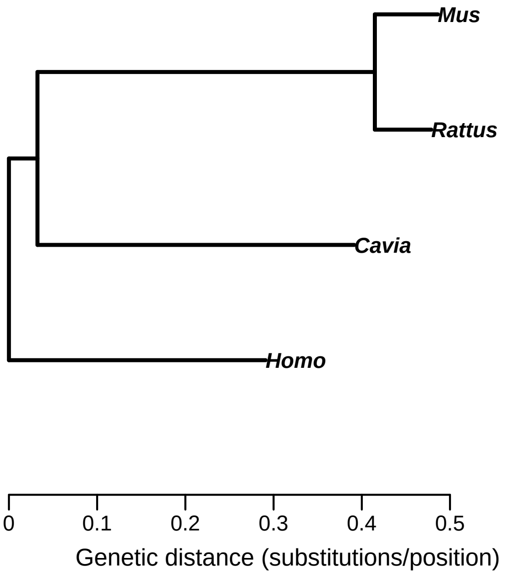

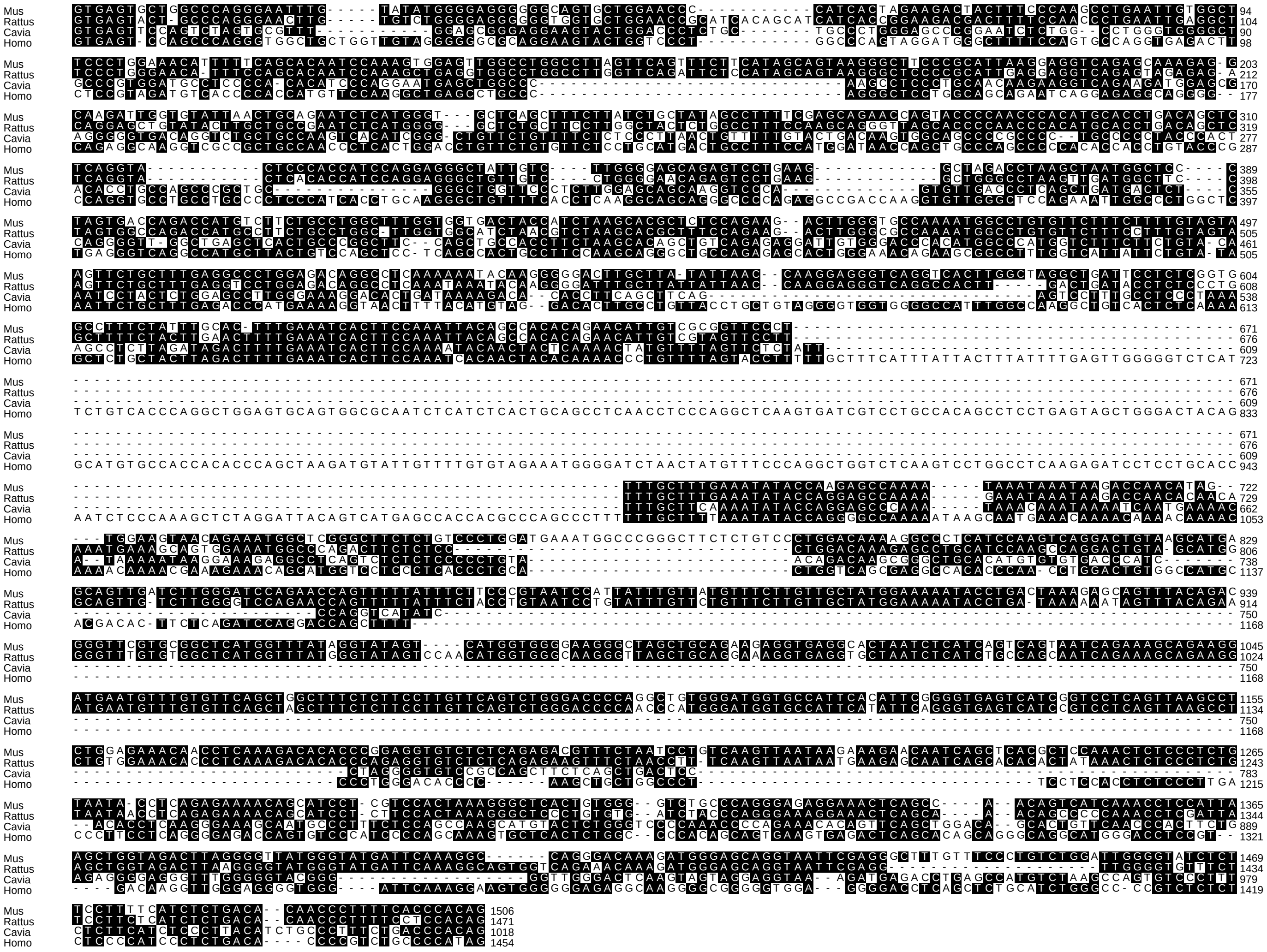
Mus
Rattus
Cavia
Homo

ENSMUSG00000022390 intron 13

Description: ubiquitous tetratricopeptide containing protein RoXaN   (Zc3h7b)
Intron number: 13
Mouse chromosome: 15
Upstream exon length: 206
Downstream exon length: 101
Mouse intron length: 1349
Intron alignment length: 2077
Total murinae branch length: 0.23634
K_score: 0.07694
Scaling factor: 0.77405

ENSMUSG00000022390 exon 13 (ORF 2)

```
Mus     CGCCTACCATCAGGAGGAGATCGATGTGTGGACAGAGGAGCGGAAGGGCACACTCAACCGGGACCTGCTCTTTGACCCTC 80
Rattus  CGCCTACCATCAGGAGGAGATCGATGTGTGGACAGAGGAGCGGAAGGGCACCCTCAACCGGGACCTGCTCTTTGACCCTC 80
Cavia   CGCCTACCATCAGGAAGAGATCGACGTGTGGACTGAGGAGCGGAAGGGCACCCTCAACCGTGACCTGCTTTTCGACCCAC 80
Homo    CGCCTACCATCAGGAGGAGATCGACGTGTGGACCGAGGAGCGGAAGGGCACCCTCAACCGCGACCTGCTCTTCGACCCGC 80

Mus     TAGGGGGTGTCAAGCGTGGCAGCCTCACCATTGCCAAGCTCCTGAAGGAGCACCAGGGCATCTTCACCTTCCTCTGTGAG 160
Rattus  TAGGGGGTGTCAAGCGTGGCAGCCTCACCATTGCCAAGCTCCTCAAGGAGCACCAGGGCATCTTCACCTTCCTCTGTGAG 160
Cavia   TGGGGGGTGTCAAGCGCGGCAGCCTCACCATCGCCAAGCTCCTCAAGGAGCACCAGGGCATCTTCACCTTCCTCTGCGAG 160
Homo    TGGGGGGTGTTAAGCGCGGCAGCCTCACCATCGCCAAGCTCCTGAAGGAGCACCAGGGCATCTTCACCTTCCTCTGCGAG 160
```

ENSMUSG00000022390 exon 14 (ORF 0)

```
Mus     ATCTGCTTCGACAGCAAGCCCCGGATCATCAGCAAAGGGACCAAGGACTCTCCATCTGTCTGCTCCAACCTGGCTGCCAA 80
Rattus  ATCTGCTTCGACAGTAAGCCCCGGATCATCAGCAAAGGCACCAAGGACTCTCCGTCTGTCTGTTCCAACCTGGCTGCCAA 80
Cavia   ATTTGCTTTGACAGTAAGCCTCGGATCATCAGCAAAGGCACCAAGGACTCTCCGTCAGTCTGCTCCAACCTGGCTGCCAA 80
Homo    ATCTGCTTTGACAGTAAACCCCGGATCATCAGCAAAGGCACCAAGGACTCTCCGTCTGTCTGCTCCAACCTGGCTGCCAA 80

Mus     GCACAGCTTTTACAACAACAA 101
Rattus  GCACAGCTTTTACAACAACAA 101
Cavia   GCACAGCTTCTACAACAACAA 101
Homo    GCACAGCTTCTACAACAACAA 101
```

ENSMUSG00000022390_intron_13

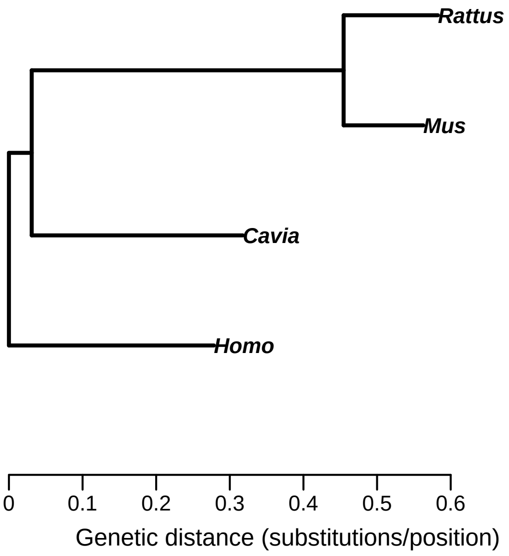

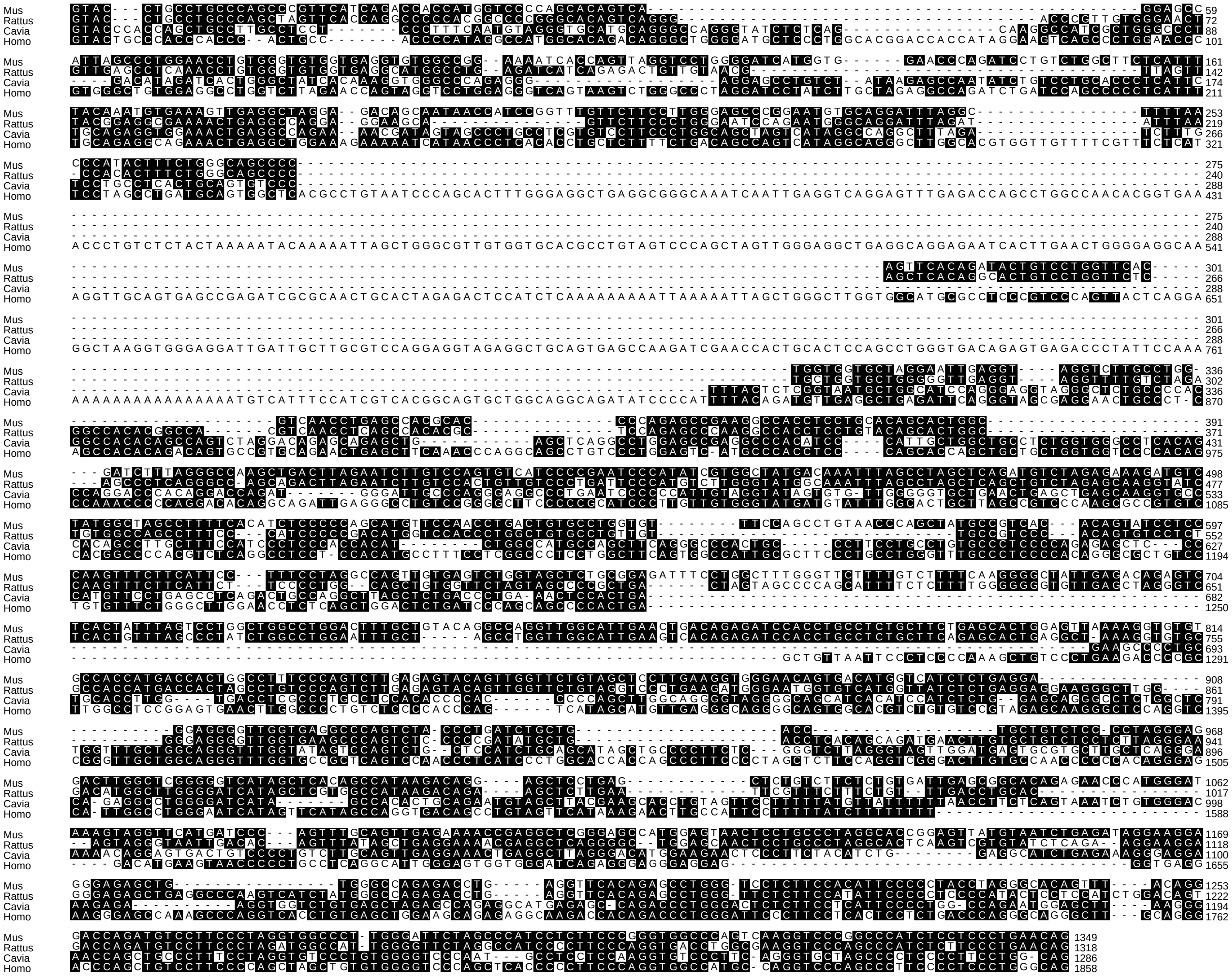

Mus
Rattus
Cavia
Homo

ENSMUSG00000001761 intron 9

Description: Smoothened homolog Precursor (Smo)
Intron number: 9
Mouse chromosome: 6
Upstream exon length: 186
Downstream exon length: 149
Mouse intron length: 344
Intron alignment length: 567
Total murinae branch length: 0.20869
K_score: 0.06199
Scaling factor: 0.7742

ENSMUSG00000001761 exon 9 (ORF 1)

```
Mus     TGCCTACCAAGAAGCCCATTCCTGACTGTGAGATCAAGAATCGGCCCAGCCTCCTGGTGGAGAAGATCAATCTATTTGCC 80
Rattus  TGCCTACCAAGAAGCCCATTCCTGATTGTGAGATCAAGAATCGGCCCAGCCTCCTGGTGGAGAAGATCAATCTGTTTGCC 80
Cavia   TGCCCACCAAGAAGCCCATTCCTGACTGTGAGATCAAGAATCGCCCCAGCCTCCTGGTAGAGAAGATTAACCTATTTGCT 80
Homo    TGCCCACCAAGCAGCCCATCCCTGACTGTGAGATCAAGAATCGCCCGAGCCTTCTGGTGGAGAAGATCAACCTGTTTGCC 80

Mus     ATGTTTGGCACTGGCATTGCCATGAGCACCTGGGTCTGGACCAAGGCCACCCTGCTCATCTGGAGGCGCACCTGGTGCAG 160
Rattus  ATGTTTGGCACTGGCATTGCCATGAGCACCTGGGTCTGGACCAAGGCCACCCTGCTCATCTGGAGGCGCACCTGGTGCAG 160
Cavia   ATGTTCGGGACCGGCATTGCCATGAGCACCTGGGTTTGGACTAAAGCCACTCTGCTCATCTGGAGGCGCACCTGGTGCAG 160
Homo    ATGTTTGGAACTGGCATCGCCATGAGCACCTGGGTCTGGACCAAGGCCACGCTGCTCATCTGGAGGCGTACCTGGTGCAG 160
```

ENSMUSG00000001761 exon 10 (ORF 1)

```
Mus     GTTGACTGGGCACAGTGATGATGAGCCCAAGAGAATCAAGAAGAGCAAGATGATCGCCAAGGCCTTCTCTAAGCGGCGTG 80
Rattus  GTTGACTGGGCACAGTGATGATGAACCCAAGAGAATCAAGAAAAGCAAGATGATTGCCAAGGCCTTCTCTAAGCGGCGTG 80
Cavia   GCTGACTGGGCAGAGTGATGACGAGCCTAAAAGGATCAAGAAGAGTAAGATGATTGCCAAGGCTTTCTCCAAGCGGCGTG 80
Homo    GTTGACTGGGCAGAGTGACGATGAGCCAAAGCGGATCAAGAAGAGCAAGATGATTGCCAAGGCCTTCTCTAAGCGGCACG 80

Mus     AGCTGCTGCAGAACCCGGGCCAGGAGCTCTCCTTCAGCATGCACACTGTCTCCCATGATGGACCTGTTG 149
Rattus  AACTGCTGCAGAACCCGGGCCAGGAGCTCTCCTTCAGCATGCACACTGTCTCCCATGATGGACCTGTTG 149
Cavia   AGCTACTGCAGAACCCAGGCCAAGAACTGTCCTTCAGCATGCACACGGTCTCCCACGACGGGCCAGTGG 149
Homo    AGCTCCTGCAGAACCCAGGCCAGGAGCTGTCCTTCAGCATGCACACTGTGTCCCACGACGGGCCCGTGG 149
```

ENSMUSG00000001761_intron_9

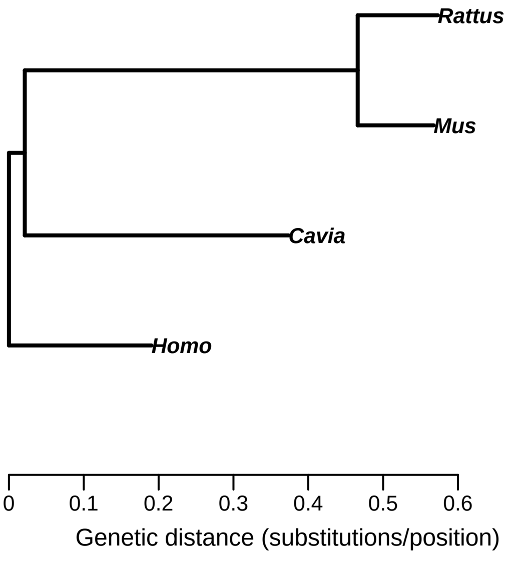

```
Mus     GTGGGGTCAGCAGCTGTCTGATCTTGT----------------------------------------------------------------------------------- 27
Rattus  GTGGGGTCAGCAGCCATCTAATCTTGT----------------------------------------------------------------------------------- 27
Cavia   GTAGAGTCAGTAGCCAGCCCATCCTACTGCTCCAGTCTTACAGGGCCGGAGATGTAGCACAGCAGCGCATTTCTTGCCTGGCAAACTCAAGCAAGTTAGTGAGTTTGATT 110
Homo    GTGGGCATGGCAGCCAGCCCCTCCTGC----------------------------------------------------------------------------------- 27

Mus     ----------------CCTTCCTCTCTTACCCTCAGCCTCACGTCTTCACT----TGAGGGCTTTGTCTTACATGCTTCCAAAACAGACTGGGGCAGTGTTTGCCAAAAG 117
Rattus  ----------------CCTTCCCCTCTTACTCTCAGCCTTATGTCTTCACT----TGAGGGCCTTGTCTTATGCTTCCAAAGCACAGACT-GGGCAGGGTTTGCC-AAAG 115
Cavia   CGCTGTACCAAAAAAACCTGCCAATCTTACTGTCAGCCTCAGCACCCCACCCCTGTTTAGGTTTAGCAGTCC---------------------------TTTTCCTGCAC 193
Homo    ----------------CCTGCCCGCCTCACCCTCAGCCTTGGGACCCCATC----TTTAGGTTTTGTCGGGT---------------------------CCTGCCTCTAG 90

Mus     CAAGTCTGGATGGCTAA---------TTGCTTAGCTGCTCACCACTGCCCTCTAG---------------------------------------------GGGACAGTGA 173
Rattus  CAAGTCGGGATGGCTGG---------TTTCTTAGCAGCTCACCGCTGCCCTCTAG---------------------------------------------GGGACAGCGA 171
Cavia   TAATCTGTGGCAGCAATTTTTCAGGCCTTCTCTGTGGCTCGCCACTGCCCCCTGGTGACA-------------------------------CCTAGGCCTGGCTCAGTG- 271
Homo    CA-------------------CAGCCTTGGTCAGTGGTTCACCGCTGCCCCCTGGTGGCACCTTCTGTCCTTGGGTGGCCTGATGCCTGGGCCTGGGCCTGGGCCAAGGG 181

Mus     G---------------------------AACCAGCGCTTTTCAAGTAGGAGCCACAGGAGCCCTGCATTCTGGGCATGTTTTGTTGCTGTGAAGGCCTCAGCTCCT--CA 254
Rattus  GGGGA-------------------GTCAGAACCAGAGTTTTGGAGGAGGAGCCGCAGGAGCCCTGCATTCTAGGCATGTTTTGTTGCTGTGAAGGCCTCTGCTCCT--CA 260
Cavia   ----TTTCATCAGCGTTCTCTTCTGTGAAT-GAGTGATTTGAGGGCCAGGCACACAGGAGCCTCACATGCCAGGCTCACTTCATCACAGGGCAGGTTTGTCCTCCTCAGA 376
Homo    CAGAGCCAGCTGCCATCACCTTTTAAGAGCGGAGTGATTGGAGGGCTGGGCACACAGGAGCTCTGCATTCTGGCCCCACTTCTTTGCAGAGAAGGCCTCTACTCCT--GA 289

Mus     GGACTTAAA------------CC-------------------------TAGAATCCCAGGTCTTTCGATAGATTTGGAAGGCAGCGGCTGCTCTTGCACTTGCCAGCCCC 327
Rattus  GAGTCCTCAGGACTCCGGGTCCT-------------------------TAGAATCCCAGGCCTTTCCACAGACTTGAAAGGAAGT-GCTGCTCCTGCACTTGCAAGCCCC 344
Cavia   AGCCACGAGGGGCCTGAGGCCTTGCGTTGTCTCCTTCTCTGAAAGGAATGGCATCACTGGTCTTTGACAAGATTTGAGGGGAAGCGGCTACTCTTCCACTTCCTGGTGTC 486
Homo    GTCCTTGAAGGACTTGAGGCCCTTGGGAGCCTCCTTCTCTGGAAAGAATGGCATCGCTGGCCCTTCCCAAGATTTGATGGGAAGTGGCAGCTTCTTCACGCTCCTTCCCT 399

Mus     ACCCCTTCTGTTCTCAG 344
Rattus  ACCCCTTCTGTTCTCAG 361
Cavia   ACTCTTTCTGCTCTTAG 503
Homo    ATCCCTTCTGCTCTCAG 416
```

ENSMUSG00000025036 intron 7

Description: Sideroflexin-2 (Sfxn2)
Intron number: 7
Mouse chromosome: 19
Upstream exon length: 67
Downstream exon length: 50
Mouse intron length: 570
Intron alignment length: 790
Total murinae branch length: 0.18664
K_score: 0.03439
Scaling factor: 0.77534

ENSMUSG00000025036 exon 7 (ORF 0)

```
Mus     AGAGCTGCGGCTGTGGGCATCGCACAAGTGGTTATCTCTCGGATCACCATGGCAGCCCCTGGCATGA 67
Rattus  AGAGCTGCGGCTGTGGGCATCACACAAGTGGTTATTTCTCGGATCACCATGGCAGCCCCTGGCATGA 67
Cavia   AGAGCTGCAGCCATAGGCATTGCCCAAGTGGTTATTTCCCGGATCACCATGGCAGCTCCTGGCATGA 67
Homo    AGAGCTGCGGCCATAGGCATCACCCAAGTAGTTATTTCTCGGATCACCATGTCAGCTCCTGGGATGA 67
```

ENSMUSG00000025036 exon 8 (ORF 2)

```
Mus     TCCTGTTGCCTGTCATCATGGAGAGGCTGGAGAGACTACACCTGATGAAG 50
Rattus  TCCTGTTGCCAGTCGTCATGGAGAGGCTGGAGAGACTACACCTGATGAAG 50
Cavia   TCTTGCTACCAGTCATCATGGAAAGGCTAGAGAAACTGCACTTCATGAAG 50
Homo    TCTTGCTGCCAGTCATCATGGAAAGGCTTGAGAAATTGCACTTCATGCAG 50
```

ENSMUSG00000025036_intron_7

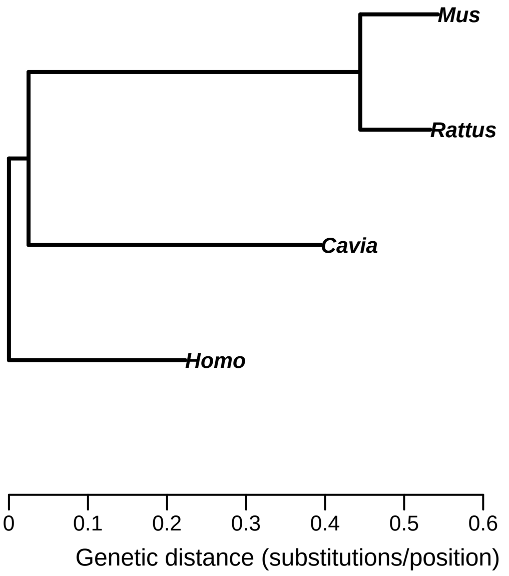

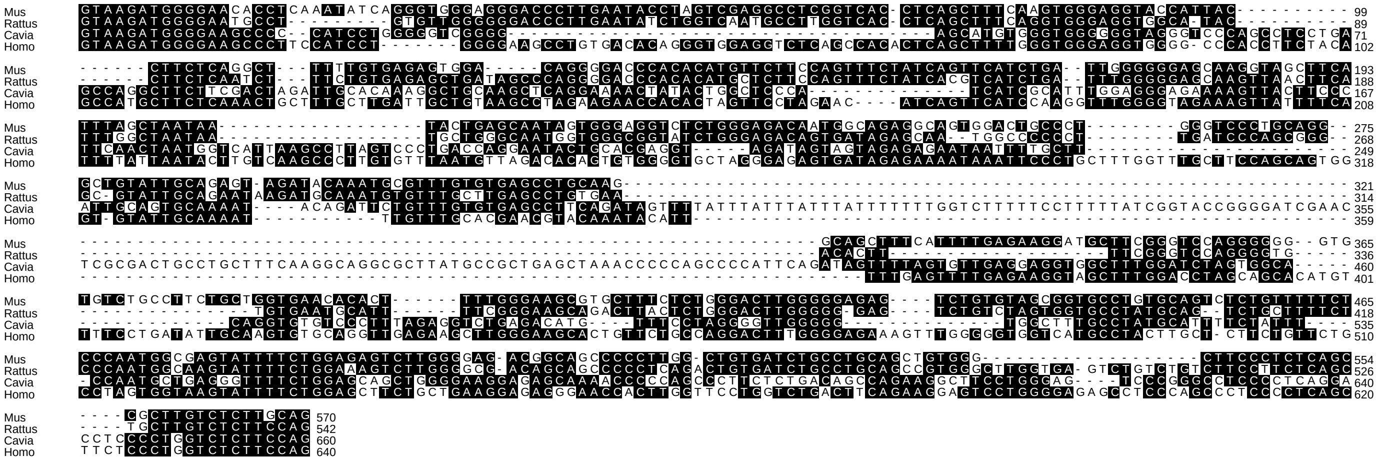

Mus
Rattus
Cavia
Homo

ENSMUSG00000021113 intron 6

Description: snRNA-activating protein complex subunit 1 (Snapc1)
Intron number: 6
Mouse chromosome: 12
Upstream exon length: 69
Downstream exon length: 63
Mouse intron length: 583
Intron alignment length: 744
Total murinae branch length: 0.25774
K_score: 0.08749
Scaling factor: 0.77646

ENSMUSG00000021113 exon 6 (ORF 0)

```
Mus     AATCCCTCCTTAAAACCCAAACTTAAAGATGGAGAAGAGAACGGTGAAGGCTCTTCAGAGGAGCCAGAG 69
Rattus  AATCCCTCCTTAAAACCGAAACTTAAAGATGGAGAGGAGGATGGCGAAGGCCCCTCAGAGGAGCCGGAG 69
Cavia   AATCCATCCTTAAAATCAAATCTTAAAGATGAAGAAGAACAGAGAGAAGGAGATACACGAGAATCACAG 69
Homo    AATCCATCCTTAAAGTCAAAAACTAATGATGGAGAAGAAAAAAATGGAAGGAAATTCACAAGAAACGGAG 69
```

ENSMUSG00000021113 exon 7 (ORF 0)

```
Mus     AGATGTGAGAGAGCTGTGTCTTTAGCGAAAATAAAAGCGAAAGCCTTTTCAGCTGTTGTCCCG 63
Rattus  AGATGTGAAAGAGCAGTGTCTTTGGCAAAAATAAAAGCAAAAGCATTTTCAGCTGTTGCCCAG 63
Cavia   AGATGTGAAAGAGCTGAATCATTAGCAAAAATAAAATCAAAAGCCTTTTCAGTTGTTGTTCAG 63
Homo    AGATGTGAAAGGGCAGAATCATTAGCGAAAATAAAATCAAAGGCCTTTTCAGTTGTCATACAG 63
```

ENSMUSG00000021113_intron_6

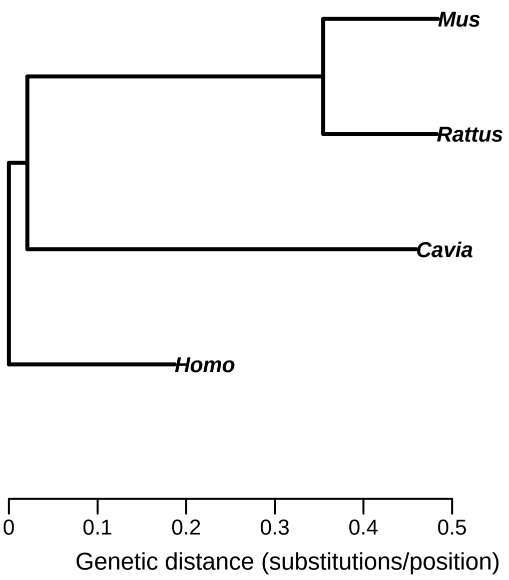

```
Mus      GTCTG---------------------------CAGAATGTTTACGGCGT-CTTTCCCTTTATGTGTGAC--TAATTCTGCTTCATAGGTGTTATGACCCTTGGGCCGAAT 80
Rattus   GTCAG-----------------------------CAGCTGTAACAGCGTCCTCTCCTTTTCTGTGTGGC--TAATTCTGCTTCCTGAGTGTTGTGACCCTTGGTCTGTGG 79
Cavia    GTCAGACCACAGCTCTGTGATCCCTTGCGGGGCAGCAGTTCACTTAGGTTCTTTGCATCTTTTTGTGGCCATGTTTGTGTATATAGAGCATCATGATCGTTAGTCTGCAG 110
Homo     GTCAGAAAACTTTGCAA-----------------------------------TTCATATTATGTGTGGCTGTGATTCTGTATACTGAGCATTGTGACCCATGGTCAGTAT 75

Mus      TATGGAA---ACACAGGAAAACTCCATTT-------------------AGTCTGTTCTTCACACGAACTGTT---GAGGTCACTCTGAGCGCTTGTCGAGAGTGAGA--T 163
Rattus   TATGAAA---ACACAGGAAAAGCCCAGTCTCTGTGTTCTGTGGTCGCAAGTCTGTTCTTCACATGAACTTTG---CAGCGCAGGCCGAC--------AGTGATCAGA--T 173
Cavia    CAGAATAACTATGAGAAAAAGTTTTGAATCCCGAGTGCTTGTGACACTGGTCCGATCAGTCCTGGAGCCTTGTGCAGGGCCGTGTGAGGCATGAGT-GGGCGTGAGC--T 217
Homo     TATAAGACATATAAGAGAAAATTCTGAATCATGCTTCTGTGAAAAGGTAGTTTGTTCTTTACTGGAAGCCTTGTGGAGGCCACATCAGGTGTAGATTGGGAGAGAGGATG 185

Mus      ATTAGGGATTGGAGCAGGCAGGGCC------GAGTGATGGGAGCTGTGTCGGTTC--------------TCATGCAGGTTTGTGGGCTGTTTTCCTGCTTTTCAGCCTTT 253
Rattus   GTTAGCAATTTGAGCAGGTGGGGCA------GAGTGGTGGGGGTGCTGTCACTTC--------------TCGGGCAGGGT--GAGGCTGTGTGCTTGCGGTCTTCTCTTT 261
Cavia    GTGACCAGGTTTAATGGGGAAAGAAA-----GCTGGTGGAGAGGGCCCACGGTTC--------------TTAGG-------------------------TGCCAGCCTTT 283
Homo     ATAATAATTTTCCGCAGGGAAAGGAAAGCTTGACTATGGAAAGTGCCCTCAGTTCGTAGAGGATCAGGATCAGGCTTTTGTAATATATCTTTTCTCACTTGTCAGCCTTT 295

Mus      GGGCCCTGGTGACACCTAGGC-------ACATGAGCAGTTTCTCATGGCCAGTGTTCCAACTGATCTG-------------TGAAGTCATCTTTAGGCACAGGCAGATGA 343
Rattus   CCGCCCTGGTGACACCTCAGC---------------------ACTTGAGCAGTGTTTCATCTGACCTG-------------TGGAGCCATCTTTGGGCACAGGAAGATGA 337
Cavia    CAGCCAGGGTGCGGCCCAGGC-----TACAATCCACTGTTTGCTGCCTGCAGAGTTTTTATTCATCCTCGAGGGTGAGAC-TTACGCTGTCTTTGGGCCAGAGTGGCCAA 387
Homo     CAACCAGTGTAGAGCCCAAGCTATTATACTATATACTGCTTCCTGCCTCCAGAGTTTTCATCCATCTTAGTGGATGAGACAAGGAGCCATCTTTGAGTATAGGCAGATGA 405

Mus      GGGAATG------------------GCTTAATCCCTAATGGTTTGTGTCT--CCTTCCTTTGTGAGCCTTGAAGGAAATGG---TCCACCAATGAGCATGGCATTAT--C 428
Rattus   GGGAATG------------------GCTTAATCCCTAAGGCTTTGTTTCT--CCTTTCTCTGTGGGCCTTGAAGGAAATGG---CCCACCAATGAGGATGGCATTATGTC 424
Cavia    GGAAGTCATGGTGCTCAAGGCCT--AGTCTGTCAGCAGGGCTTCCGCCCCGGTCTGGTCGCTTGGGCTTTA----------------------------GGCAGCAGGGC 467
Homo     GGAGGGAATGGCAGTTCAGGGCTCTGCCTGATCACCCAGGCTTTCATTCATACTGTCCTCCTTGAATCCTGAATGAAAATGATCTCTAACTATGGGGATGGTGTTATGGC 515

Mus      ACCCGCCATGTGGCAGGCAGA-----------------GGGTATCTG-----------TCA------CGTGTGATTTCTTACCAGTGTGT-TCATAAACTGTCTTAATCC 503
Rattus   ACCTGCCATGTGGCAGCAGAG-----------------G-------------------CCC------TGTGTGATTTCTTATCAGTGTGT-GCCTAAACAATCTTAATCC 491
Cavia    CTCAGCTGTGTCTGCTCACGG-----------------GGGGATCTGCGCTCCCTCCTCCC------TGTTGGGTTTCCTTTTGCTACGCAGCCTTCTGTCTGTTATTCA 554
Homo     ACCTGCTACATCTGTGAGAGAATATGATGTATGTTTATGTGTGTGTACTCAATGTATACCCAATGTATGTTAGTTTCTTTTACCCTACATTGTGTCTGAAATCTTATTTC 625

Mus      CAAGGATAGGTCCTTGCGTTTTCACAATTTC-CCTAACGTTTATG---TTTCAACTTGGTTTCCCTTGTTTATTTTCCTTCCAG 583
Rattus   TGAGGGTAGGTCATTGGGTTTTCATAATTTC-CCTAACATTTATG---TTTCAGCTTGGTTTTCCTTGTTTATTTTCCTTCCAG 571
Cavia    TGGTAACAGACATGCAG---TTGATGATTTCACCTGGTGTCTGTGAGTTTTAACTCTGTTTTTCCTTCTTTATTTTTATCTCAG 635
Homo     TGATAATAAATACTTGGGTATTCAGTTTTTCACCTAATGTTTGTGTGTTTTAACTTT--TTTTTCCTTCTTATTTTACATCCAG 707
```

ENSMUSG00000021492 intron 4

Description: coagulation factor XII (F12)
Intron number: 4
Mouse chromosome: 13
Upstream exon length: 71
Downstream exon length: 111
Mouse intron length: 328
Intron alignment length: 497
Total murinae branch length: 0.22002
K_score: 0.02086
Scaling factor: 0.77707

ENSMUSG00000021492 exon 4 (ORF 1)

```
Mus     GTGTGCTACCACCCCCAACTTTGATGAAGATCAGCAATGGGGATACTGCTTGGAGCCCAAGAAAGTGAAAG 71
Rattus  GTGTGCTACCACCCCCAACTTTGACGAGGACCAGCAATGGGGATACTGCTTGGAGCCCAAGAAAGTGAAAG 71
Cavia   GTGTGCCACCACCCCCAACTTTGACCAGGACCAGCAATGGGCATACTGCCTGGAGCCCAAGAAAGTAAAAG 71
Homo    GTGTGCTACCACCCCCAACTTTGATCAGGACCAGCGATGGGGATACTGTTTGGAGCCCAAGAAAGTGAAAG 71
```

ENSMUSG00000021492 exon 5 (ORF 2)

```
Mus     ACCATTGCAGCAAACACAACCCGTGCCACAAAGGAGGGACATGTATCAACACCCCCAATGGGCCACACTGTCTCTGCCCT 80
Rattus  ACCATTGCAGCAAACACAGCCCCTGCCACAAAGGAGGGACGTGTGTCAACACCCCCAACGGCCCGCACTGTCTCTGCCCT 80
Cavia   ACCACTGCAGCAAACACAACCCCTGCCAGAGAGGAGGGATCTGTGTCAATACGTTGAGCAGCCCTCACTGCCTCTGTCCA 80
Homo    ACCACTGCAGCAAACACAGCCCCTGCCAGAAAGGAGGGACCTGTGTGAACATGCCAAGCGGCCCCCACTGTCTCTGTCCA 80

Mus     GAACACCTCACTGGGAAACATTGCCAGAAAG 111
Rattus  GAACACCTCACCGGGAAACATTGCCAGAGAG 111
Cavia   GACCACCTCACTGGGAAACACTGTCAGAGAG 111
Homo    CAACACCTCACTGGAAACCACTGCCAGAAAG 111
```

ENSMUSG00000021492_intron_4

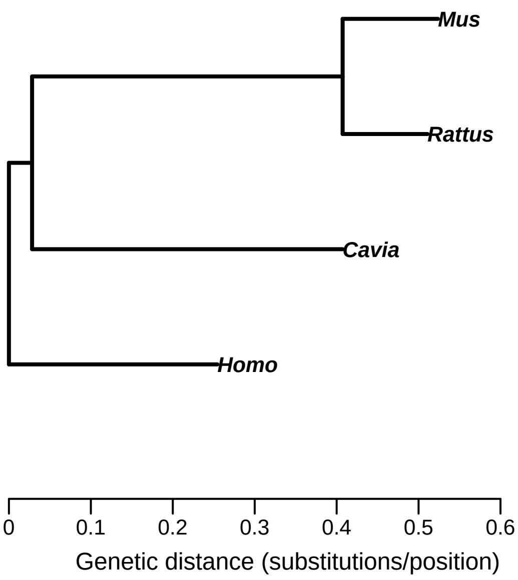

```
Mus     GTATGTATGGCATGCAGGCCCC-GGGTGGCTCAAGGCTGTGTGTGTCTTGCTGCTCACTCC-----GTTCCCACACACCTACTCACCTACTAAGCATCATCAGATGCCAA 104
Rattus  ----GTATGGCATGCAGGCCCC-GGGTGACTCAAGACTGTGTGTGCCCTGCTCCTCACTCCACTCGGTTCCCACACACCTTCTCGGGTGCCACA-------------CAC 92
Cavia   ----GTATGCCCCACAGCATCTGGGGTGGCCCAGGGCC-------------------------------CTCTCCCATCAGTTCTGCTCCTGAGTATCACTGGATCTCAC 76
Homo    ----GTGCTACACACAGCCTCTGGGGTGGCCTGGGGCT----------------------------CTCTCCTCCCGCCTCATTACTCTCCTGGTATCACCAGACCCCAC 78

Mus     ACACTTGGGATTCTGGGCCCCGCCCTTTTCCTTCTCTTACAGCATTCTTTCAAAAGTCAGAGGAAGAATTCTGGGGAAAGAGGCCACCATTGTCCAGATGAGCTTG---- 210
Rattus  ACACTTGGGATTCCTGGCCCAGCCCCTTCCCTTCTCCTACAGAGTCCTTTCAAAAGTCAGAGGGAGAATTCTGGGGGAAGAGGCCACCCTTGTCCAGATGAGTTTAATTT 202
Cavia   ACAGCTGGATTCTGTATCCTTGTCCTTT--CCTCCTTCACAATATCTTTTAGGAAGCCCGAGGGAGAGTTCTGAACAGAGATAGTCTGATTTTGTAGATAAA--TAAACT 182
Homo    ACACCTGGGATTCTGGACCCAGCCCCTTCTCTCCCTCCACAATACCCTTTGGAAGTCCAGAGGGAGAGTTCTGGGAAGGAGTGGTCCCATTTTGCAGGTGGG--TAAACC 186

Mus     AGGGTCTAGAAGCTTGGGGTTCTATGGTTTGATGACAAGCAGGTGCAGGCAGGGTATTGGTCCCTACATATGTCCTTCCGACTCCGGAAGTGCTTCGCTCTTGTTA---T 317
Rattus  AAGGTCTAGAAGCTTGGGGTGCCAGGGTTCTATGACAAGCAGGTTCAGGCAAGGCATTAG----------TGTCACTTGGACTCTGG--GTACTTCTTTCTTTTTTTTTT 300
Cavia   AAGACTTGGCAACTTGG---TCTAAAGTCTCATGACAAGGAGGTTCAGGTAGGGACTTGGCCCTCGCCTGTGTGACTCAGGCTCACC--CTGCTTCCTCCTGGTTTCTGT 287
Homo    AAG-CTTGGAAACTTGGAGTAGCAAGGTCACAAGGCAAGTAGGTTCAAGAAGGGCCTTGGCCCCCAGCTGTGTGACTCAGCTCCCTG--CTCTTCCTTCCACCATG---T 290

Mus     CTA--------------------------------------------------------------------------------------------------------- 320
Rattus  TTAAACTCCCCCCCCCCTGGTTTTTTTTTTTTTTTTTTTTTTTTTTTTTTTTTTTTTTTTTTTGGAGCTGGGGACTGAACCCAGGGCCTTGCGCTTGCTAGGCAAGCGCT 410
Cavia   CTG--------------------------------------------------------------------------------------------------------- 290
Homo    CCA--------------------------------------------------------------------------------------------------------- 293

Mus     -------------------------------------------------CCTCTCAG 328
Rattus  CTACCACTGAGCTAAATCCCCAACCCCTGGGTACTTCTTTCTTGAATCCTCTCTCAG 467
Cavia   ------------------------------------------------------TAG 293
Homo    -------------------------------------------------TCTCTCAG 301
```

ENSMUSG00000003234 intron 17

Description: ATP-binding cassette sub-family F member 3 (Abcf3)
Intron number: 17
Mouse chromosome: 16
Upstream exon length: 89
Downstream exon length: 92
Mouse intron length: 261
Intron alignment length: 274
Total murinae branch length: 0.30050
K_score: 0.07523
Scaling factor: 0.77728

ENSMUSG00000003234 exon 17 (ORF 0)

```
Mus     GTTGGGGAGAATGGGGCTGGGAAATCTACTATGCTGAAGCTGCTCATGGGGGACCTGTCTCCTGTTCGGGGTATCAGGCA 80
Rattus  GTCGGGGAGAATGGGGCTGGGAAATCCACTATGCTGAAGCTGCTCATGGGGGACCTGGCTCCTGTTCGGGGTATCAGGCA 80
Cavia   GTTGGGGAGAATGGGGCCGGGAAGTCTACCATGCTGAAGCTGCTCTTGGGAGACCTGGCACCAGTCCGTGGCATCAGGCA 80
Homo    GTTGGAGAGAATGGGGCTGGGAAGTCTACCATGCTGAAGCTGCTTTTGGGGGACCTGGCACCTGTTCGGGGCATCAGACA 80

Mus     TGCCCACAG 89
Rattus  TGCCCACAG 89
Cavia   CGCCCACAG 89
Homo    CGCTCACAG 89
```

ENSMUSG00000003234 exon 18 (ORF 1)

```
Mus     GAATCTGAAGATAGGCTATTTTAGCCAGCACCACGTGGAACAGCTAGACTTGAATGTCAGTGCTGTGGAACTCCTGGCTC 80
Rattus  GAATCTGAAGATAGGCTATTTCAGCCAGCACCATGTGGAGCAGCTAGACTTGAATGTCAGTGCTGTGGAACTCCTGGCTC 80
Cavia   GAATCTCAAGATTGGCTACTTCAGCCAGCACCATGTGGAGCAGTTGGACCTGAATGTCAGTGCTGTGGAGCTGCTGGCAC 80
Homo    GAATCTGAAGATTGGCTATTTCAGCCAGCACCATGTGGAGCAGCTGGACCTAAACGTCAGTGCTGTGGAACTGCTGGCAC 80

Mus     GAAAATTTCCTG 92
Rattus  GAAAATTTCCTG 92
Cavia   GCAAGTTCCCAG 92
Homo    GCAAGTTTCCTG 92
```

ENSMUSG00000003234_intron_17

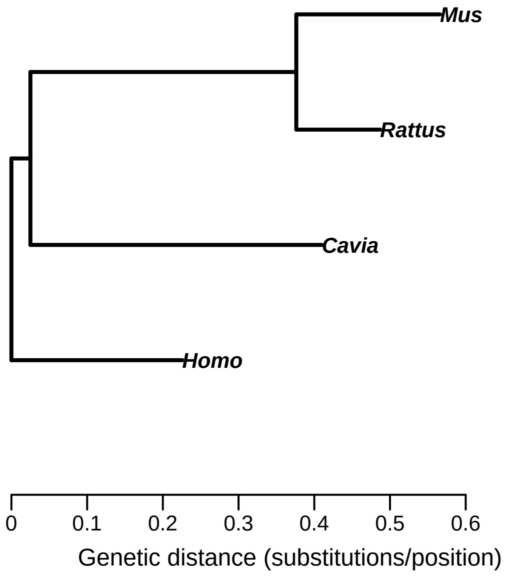

```
Mus     GTAATGGATCCCCACCCCACCCCCAGTGAACTGTGCACACACTTGAACTCAGCTACATATACTACTCCACAGCGCTCAGGTAATGGATTCCCCCCATGAACTGTATGCAC 110
Rattus  GTAATGGATCCCCCTGACCCAATCAATGAAC-----------------------------------------------------------------------ACACGCAC 39
Cavia   GTAAGCTGCCCC------------------------------------------------------------------------------------------GCAGGCT- 19
Homo    GTCAGGCCCACCC-----------------------------------------------------------------------------------------GCACCCCT 21

Mus     ACTCACCTCACACACATTCAGCTGCCTATATATTCCACAGCCCATAGGTAATGGACCCCACCCCACTCCATGAACTATGTGCACACTCATACACATTCA-GCTGCCTATA 219
Rattus  ACACAC---------------------------------------------------------------------ACACACACACACACACACACTCA-GCTGCCTGTA 78
Cavia   ---------------------------------------------------------------------------ACCACCACACCTTCCCGCACCCCGGCCTCCTGCA 53
Homo    GCCCCC---------------------------------------------------------------------ATGAGCACATTTGCAGGCACCCATGCTGCCTGCG 61

Mus     CTACTTCACAGTTGTGCTTCCTACCTTCATTTTT------------GCCTCCAG 261
Rattus  CTGCTTCACAGTTGTGCTTCCTGCTTTGTTTTT-------------GTCTGCAG 119
Cavia   CAGCCTC----------CCTTCGCCCCCTTTCCC------------CTCTGCAG 85
Homo    CTCCTTCGTGGCCATTGCCTTTGTCTGTTTTTCCACCTCGGCTTCTGCCTGCAG 115
```

ENSMUSG00000021559 intron 14

Description: Death-associated protein kinase 1 (Dapk1)
Intron number: 14
Mouse chromosome: 13
Upstream exon length: 99
Downstream exon length: 198
Mouse intron length: 850
Intron alignment length: 2150
Total murinae branch length: 0.13485
K_score: 0.05011
Scaling factor: 0.7781

ENSMUSG00000021559 exon 14 (ORF 0)

```
Mus     TCTGGAGAGACAGCTCTTCACGTGGCAGCCCGCTATGGCCATGCAGATGTGGTTCAACTACTGTGCAGTTTTGGCTCTAA 80
Rattus  TCTGGAGAGACAGCTCTTCACGTGGCAGCCCGCTATGGCCATGCAGATGTGGTTCAACTCTTGTGCAGTTTTGGCTCTAA 80
Cavia   TCTGGAGAGACAGCCCTGCACGTGGCAGCTCGCTATGGCCACGCAGATGTAGTTCAGCTTCTGTGTAGCTTCGGATCCAA 80
Homo    TCTGGAGAGATGGCCCTCCACGTGGCAGCTCGCTATGGCCATGCTGACGTGGCTCAGTTACTGTGCAGCTTCGGCTCAAA 80

Mus     TCCTGATTTCCAGGACAAG 99
Rattus  TCCTGATTTCCAGGACAAG 99
Cavia   TCCTGACTTCCAGGACAAG 99
Homo    TCCCAATATCCAGGACAAG 99
```

ENSMUSG00000021559 exon 15 (ORF 0)

```
Mus     GAAGAGGAAACCCCCCTGCACTGTGCTGCCTGGCATGGCTATTACTCCGTGGCTAAAGCTCTTTGTGAAGTTGGCTGCAA 80
Rattus  GAAGAGGAAACCCCCCTGCACTGTGCTGCCTGGCACGGCTATTACTCCGTGGCTAGAGCTCTTTGTGAAGTTGGCTGTAA 80
Cavia   GAAGAGGAAACACCCCTGCACTGTGCCGCTTGGCATGGCTATTACTCTGTGGCCAAAGCCCTCTGCGAAGCCGGCTGTAA 80
Homo    GAAGAAGAAACCCCCCTGCACTGTGCTGCTTGGCACGGCTATTACTCTGTGGCCAAAGCCCTTTGTGAAGCCGGCTGTAA 80

Mus     CGTGAATATCAAGAATCGGGAGGGAGAGACCCCATTGCTGACGGCGTCTGCCAGGGGCTATCATGACATTGTGGAGTGTC 160
Rattus  CGTGAATATCAAGAACCGGGAGGGAGAGACCCCACTGCTGACAGCTTCTGCCAGGGGCTACCATGACATTGTGGAGTGTC 160
Cavia   TGTGAACATCAAGAATCGGGAAGGAGAGACACCGCTCCTGACAGCCTCCGCGAGAGGCTATCATGACATCGTGGAGTGTC 160
Homo    CGTGAACATCAAGAACCGAGAAGGAGAGACGCCCCTCCTGACAGCCTCTGCCAGGGGCTACCACGACATCGTGGAGTGTC 160
```

ENSMUSG00000021559_intron_14

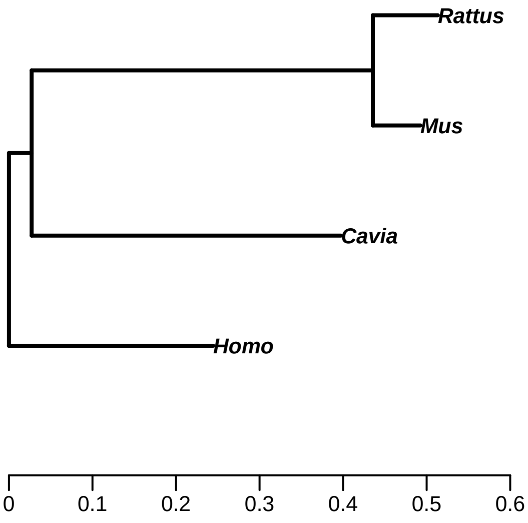

Genetic distance (substitutions/position)

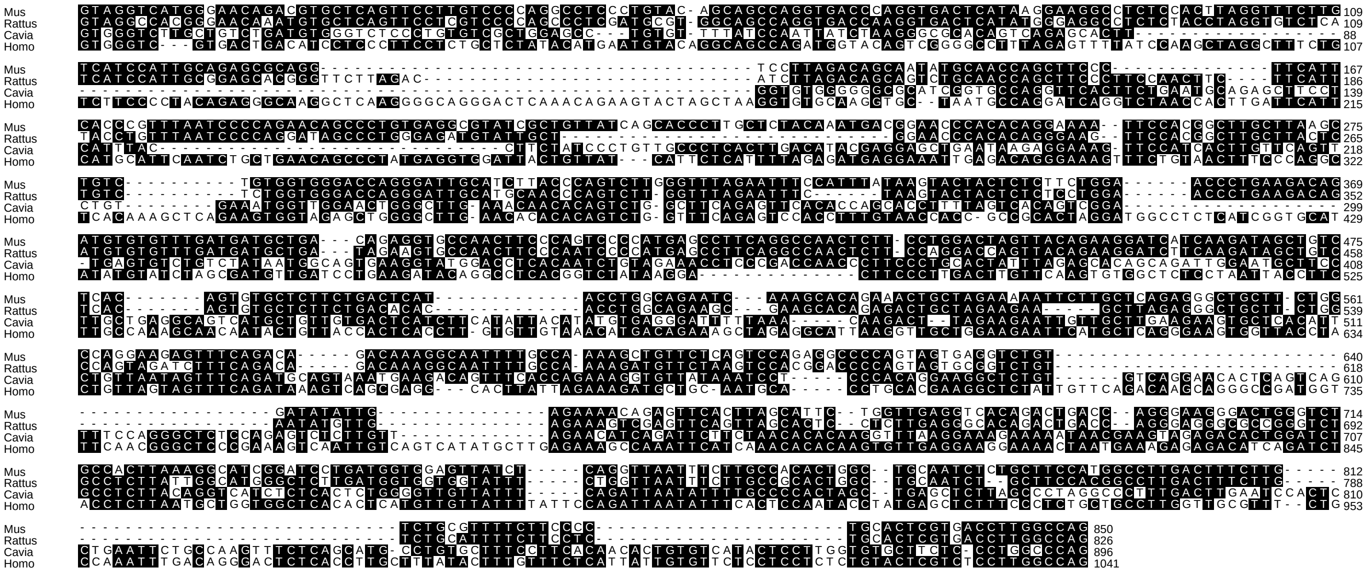
Mus
Rattus
Cavia
Homo

ENSMUSG00000036854 intron 1

Description: Heat shock protein beta-6 (Hspb6)
Intron number: 1
Mouse chromosome: 7
Upstream exon length: 42
Downstream exon length: 123
Mouse intron length: 1199
Intron alignment length: 1514
Total murinae branch length: 0.19814
K_score: 0.07685
Scaling factor: 0.77868

ENSMUSG00000036854 exon 1 (ORF 0)

```
Mus     ------------------------------------------------------------------------CCCAGGGT 8
Rattus  CCGTGCTTCAGCTCCTTTACCGGGTTTTTCCACTCCGGGACGCCTCTTTGACCAGCGTTTCGGCGAAGGGCTGCTTGAGG 80
Cavia   CCGCGCTTCAGCCCCGTTGCCCGGGCTGTCCGCGCCGGGTCGCCTCTTTGACCAGCGCTTCGGCGAGGGACTGCTGGAGG 80
Homo    CCGCGCCTCGGCCCCGTTGCCCGGACTTTCGGCGCCCGGACGCCTCTTTGACCAGCGCTTCGGCGAGGGGCTGCTGGAGG 80

Mus     CCCAACTG-------------CCACAATGATTGCCAC-----ACCTATAAG----------------------------G 42
Rattus  CAGAGCTGGCTTCACTGTGCCCTGCTGCGATCGCGCCCTACTATCTGCGCGCCCCCAGTGTGGCGTTACCCACAGCCCAG 160
Cavia   CCGAACTGGCTACGCTCTGCCCTGCTGCGCTCGCCCCCTACTACCTGCGCGCCCCCAGCGTGGCGCTGCCCACCGCCCAG 160
Homo    CCGAGCTGGCTGCGCTCTGCCCCACCACGCTCGCCCCCTACTACCTGCGCGCACCCAGCGTGGCGCTGCCCGTCGCCCAG 160
```

ENSMUSG00000036854 exon 2 (ORF 0)

```
Mus     GTGTCCACGGACTCTGGGTATTTTTCCGTGCTGCTGGATGTGAAGCACTTCTTGCCAGAGGAAATCTCTGTCAAGGTGGT 80
Rattus  GTGCCCACGGACCCTGGGTATTTTTCTGTGCTGCTGGATGTGAAGCACTTCTCGCCAGAGGAAATCTCTGTCAAGGTGGT 80
Cavia   GTGTCGACCGACCCAGGCCACTTCTCGGTGCTGCTGGACGTGAAGCACTTCTCGCCGGAGGAAATAGCCGTCAAGGTGGT 80
Homo    GTGCCGACGGACCCCGGCCACTTTTCGGTGCTGCTAGACGTGAAGCACTTCTCGCCGGAGGAAATTGCTGTCAAGGTGGT 80

Mus     TGACGACCATGTGGAGGTCCATGCTCGGCACGAGGAGCGCCCG 123
Rattus  TGGTGACCATGTGGAGGTCCATGCTCGGCATGAGGAGCGCCCA 123
Cavia   TGGCGAGCACGTGGAGGTGCACGCGCGCCACGAGGAGCGTCCG 123
Homo    GGGCGAACACGTGGAGGTGCACGCGCGCCACGAGGAGCGCCCG 123
```

ENSMUSG00000036854_intron_1

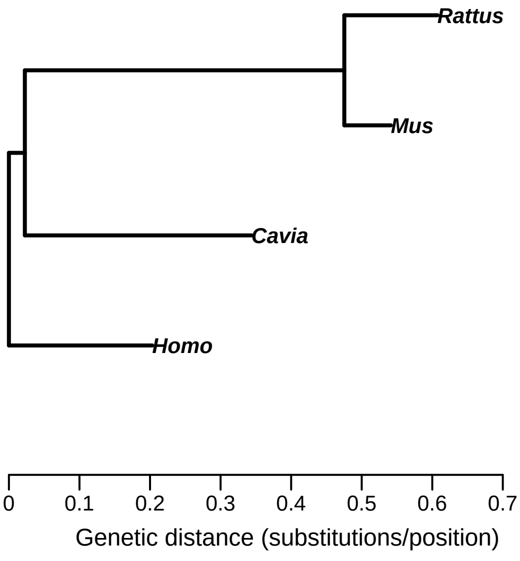

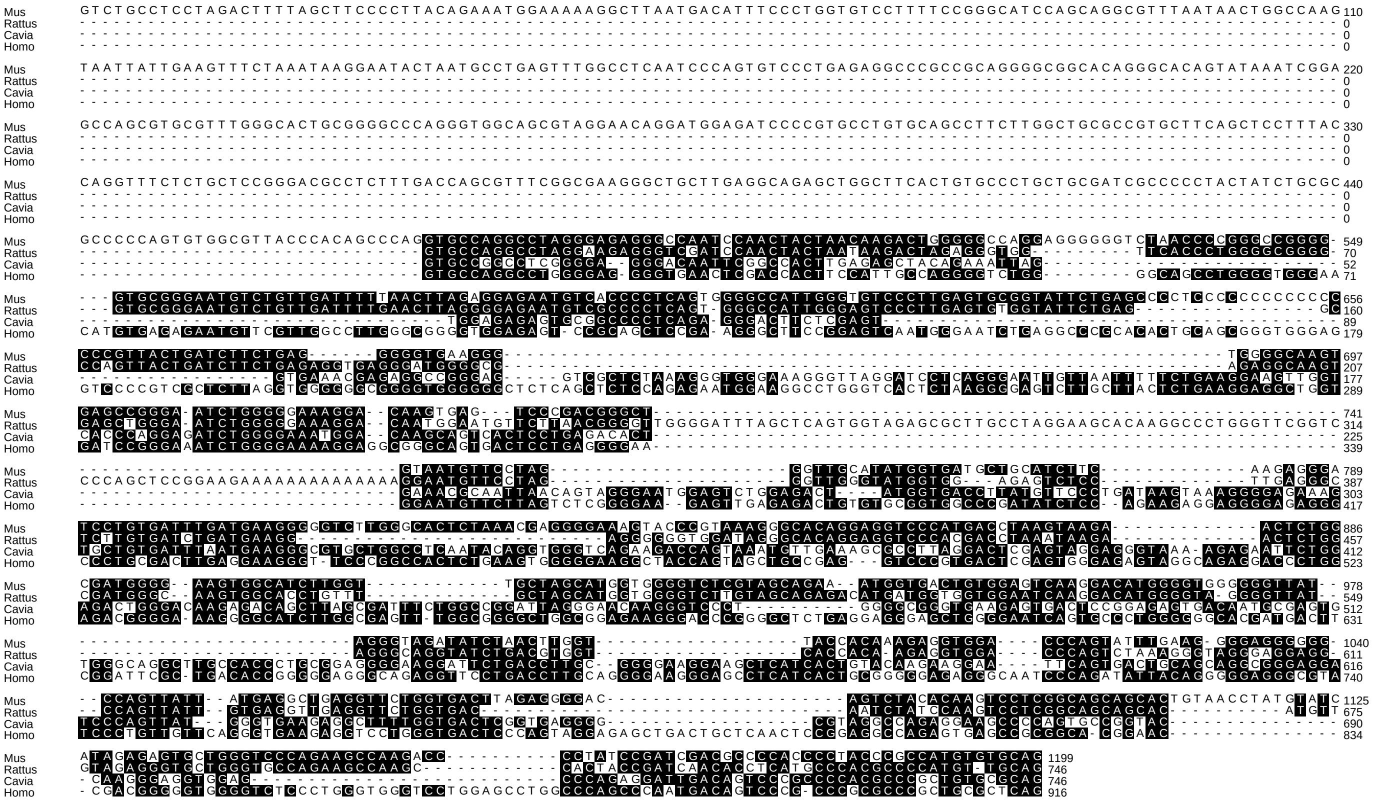

ENSMUSG00000078667 intron 3

Description: Uncharacterized protein C1orf189 homolog (1700094D03Rik)
Intron number: 3
Mouse chromosome: 3
Upstream exon length: 130
Downstream exon length: 102
Mouse intron length: 955
Intron alignment length: 1182
Total murinae branch length: 0.20488
K_score: 0.0878
Scaling factor: 0.77933

ENSMUSG00000078667 exon 3 (ORF 1)

```
Mus     GTGGCGGAATTTTCATATTCACTGTATGTGGCAGACAACATTGGACCAACGGAGAAACCTATTTGCTGCCCTAAGGATGA 80
Rattus  GTGGCAGAATTTTCATACTCAATGTTTGTGGCAGATGACATTGGACCAACGGAGAAACCTGTATGCTACCCTAAGGATGC 80
Cavia   GTGGCAAAAGTTACATACTCATTGTATGTGGCAGATGACATTGAGCCAGAGAAGAAACCCATATGCTATCCTAAGGATGC 80
Homo    GTGGCGAAATTCACATACTCACTGTCTGTGGCAAATGGCATTGGGCCAGAGAAGAAACCCGTATGCCACCCTAAGGATGC 80

Mus     AGGATACTAAGGAACAGGAATTAGCACTGTCTAACAAACAGCTACTAGTG 130
Rattus  AGGACACTATGGAACAGGAATTAGCACTGTCCAACAAACAGCTCCTGCTG 130
Cavia   AGGCCACCATGGAACAAGAATTGGCGCTAGCTAACAAACAACTGCTGGTG 130
Homo    AGGACACCATGGTACAGGAATTGGCACTGGCCAAAAAGCAACTACTAATG 130
```

ENSMUSG00000078667 exon 4 (ORF 0)

```
Mus     GTCCGTCAAGCTGCCTTACACGAGCTGTTTGAAAAGGAGTATCAGCAGTACCAGCAGGAACTCAATCAGATGGGCAAAGC 80
Rattus  GTCCGTCAAGCTGCCTTGCACGAGCTGTTTGAAAAAGAATATCAACAGTACCAGCAGGAGCTCAATCGAATGGGCAAAGC 80
Cavia   GTCCGGCAAGCTGCCCTGCACCAGCTATTTGAAAAGGAGCATCAGCAGTACCAGCAGGAACTCAACCAGATGGGCAAAGC 80
Homo    GTCCGTCAAGCTGCCCTGCACCAGCTGTTTGAAAAGGAGCATCAGCAGTACCAGCAGGAACTAAATCAGATGGGCAAAGC 80

Mus     TTTTTATGAGGAAAGACTCTGA 102
Rattus  TTTTTATGAGGAGAGACTCTGA 102
Cavia   TTTTTATGTGGAGAGACTCTGA 102
Homo    TTTTTATGTAGAGAGATTCTGA 102
```

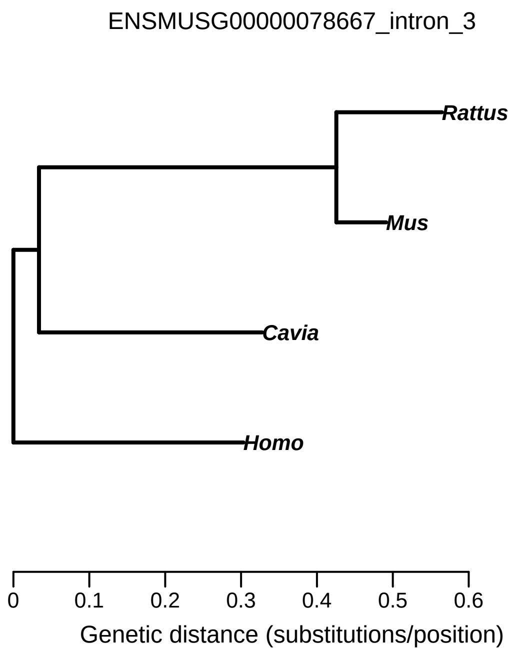

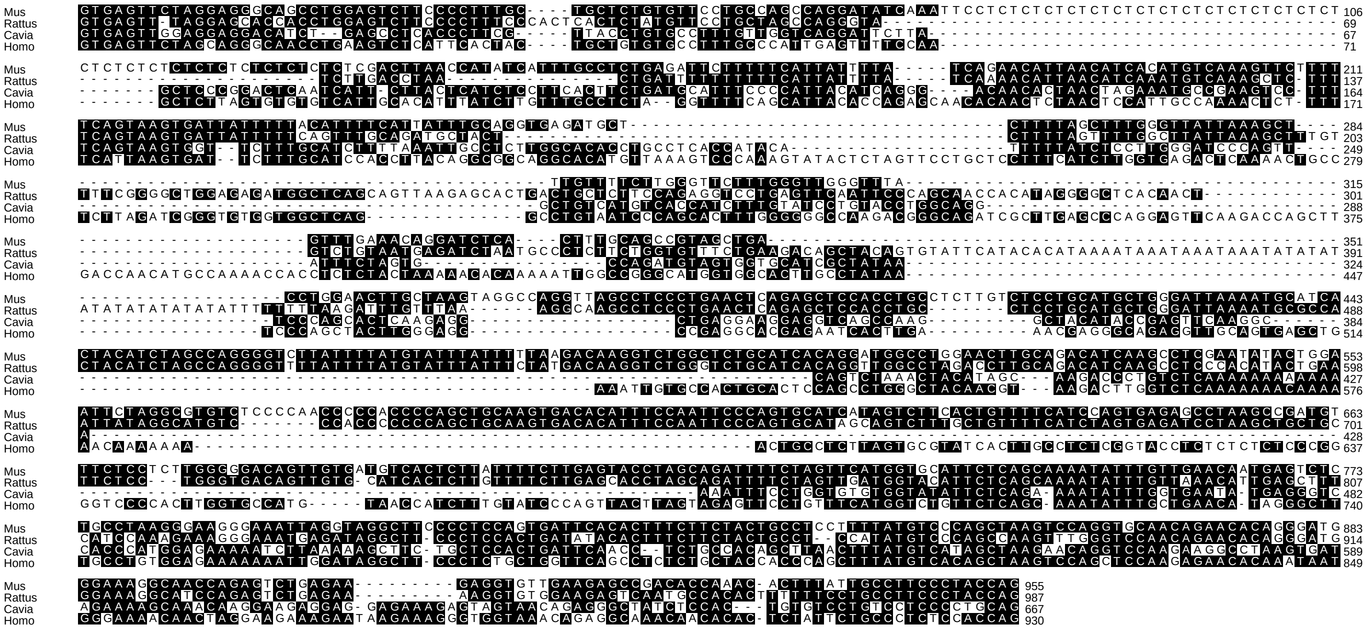

ENSMUSG00000045620 intron 3

Description: Outer dense fiber protein 3-like protein 1 (Odf3l1)
Intron number: 3
Mouse chromosome: 9
Upstream exon length: 114
Downstream exon length: 482
Mouse intron length: 662
Intron alignment length: 1028
Total murinae branch length: 0.14684
K_score: 0.07202
Scaling factor: 0.77959

ENSMUSG00000045620 exon 3 (ORF 2)

```
Mus     GGATCATAGACATAAACAGCCCCGGACCTTGCTATTTCTTGAATCCCAAAGTAACTCGTTTTGGAATATCCACCTGCCCC 80
Rattus  GGATCACTGACGAAAACAGCCCTGGACCTTGCTATTTTTTGGATCCCAAAGTAACTCGTTTTGGAATATCTACCTGTCCC 80
Cavia   GGATCACAGACATCTGTAGCCCAGGACCCTGCTATTGCTTGGATCCCAAAATAACCCGCTTTGGAATGTCCAGCTGCCCA 80
Homo    GGATGGTGTGCCACAGCAGCCCTGGGCCTTGCTATCTCTTGGATCCCAAAATAACTCGGTTTGGAATGTCCAGCTGCCCG 80

Mus     CAGGTCCCCATGGAGGAGCGCATCTCCAATCCAC 114
Rattus  CAGGTTCCCATGGAGGAGCGCATCCCCAACCTAC 114
Cavia   CAGGTGCCCATGGATGGCCGTGTCACCAGCCTGC 114
Homo    CAGGTCCCCATGGAGGAGCGCATCTCCAACCTGC 114
```

ENSMUSG00000045620 exon 4 (ORF 2)

```
Mus     GCATAAATTGCATGCCAGCCTCCTGCAAGTACAACCTTGAGAAGACTCGACCCTCTGGGGAACGTCAGCCTCCCCAGTAC 80
Rattus  GCATAACTAGTACGCCAGCCTCTTGCTACTACAACCTCCAGAAGACCCAACCCTCTGGGGAACGTAGGCCTCCCCAGTAC 80
Cavia   GTCTGAGCCCCACACTGGCCCCCTGCCACTATAATCTGGAGAAGACCCATCCCCCCGATGAGTGCAGGGCTCCCCAGTAC 80
Homo    GCCTGAACCCCACCCTCGCATCCTGCCAGTACTACTTTGAGAAGATCCACCCACCGGGGGAACGCAGGGCTCCCCAGTAC 80

Mus     ACTTTTGGCTACCGGTGTCCATATAGAGTGATGGATCCCAACCCTGCTCCTAACCAGTACCAGCTGCCAGTCACTCTGGG 160
Rattus  TCTTTTGGCTATCGGTGTCCATATAGAGTGATGGACCCCAATCCGGCTCCTAACCGGTACAAGCTGCCATCCTCTCTGGG 160
Cavia   TCCTTTGGCTATCGGTGCCCAGTTCGAGTGATGGATCCCAACCCGGCCCCCAATTGCTACCAGCTGCCCCTCTCACTGGG 160
Homo    ACGTTTGGCTACCGGCGCCCATACAGAGTGATGGACCTCAACCCGGCTCCCAACCAGTACCAGATGCCACTCTTGCTGGG 160
```

ENSMUSG00000045620_intron_3

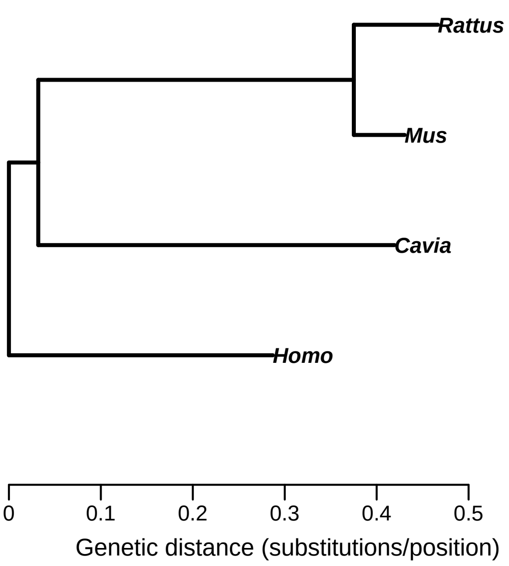

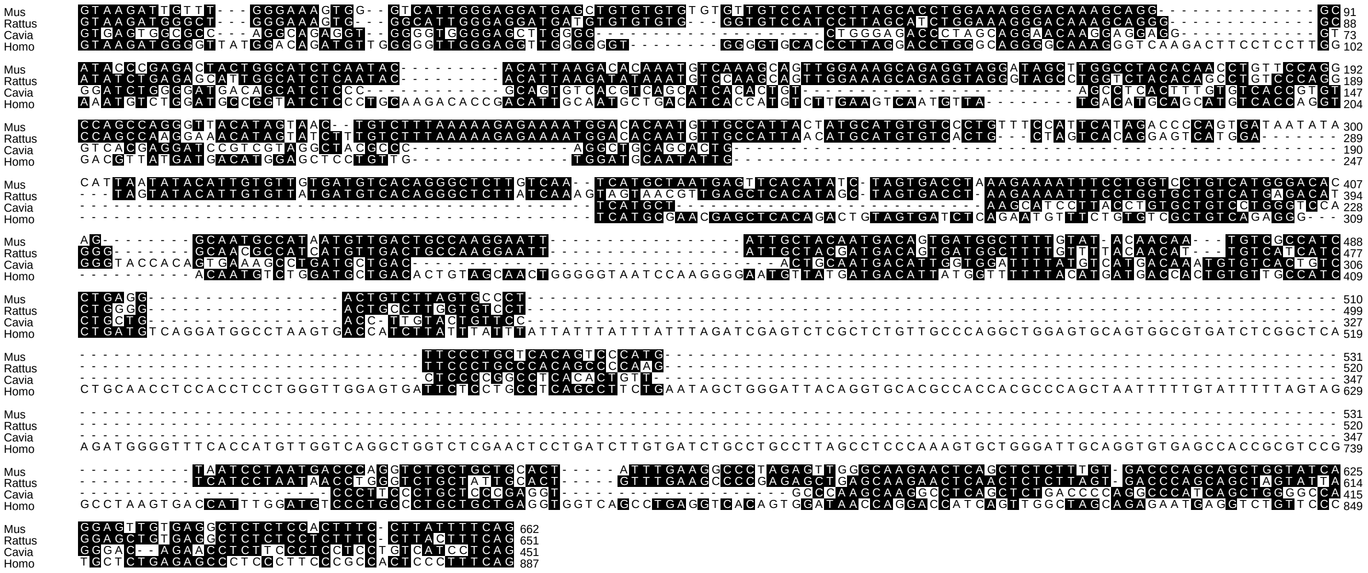
Mus
Rattus
Cavia
Homo

ENSMUSG00000034088 intron 10

Description: Vigilin (Hdlbp)
Intron number: 10
Mouse chromosome: 1
Upstream exon length: 140
Downstream exon length: 105
Mouse intron length: 1392
Intron alignment length: 1768
Total murinae branch length: 0.18537
K_score: 0.02681
Scaling factor: 0.77971

ENSMUSG00000034088 exon 10 (ORF 2)

```
Mus     TCAACAGAATCAAAGACCAGTACAAAGTGTCTGTGCGCATCCCCCCTGACAGTGAAAAGAGTAACCTGATCCGAATTGAG 80
Rattus  TCAACAGAATCAAAGACCAGTACAAAGTGTCTGTGCGCATCCCCCCTGACAGCGAGAAGAGTAACCTGATCCGAATTGAG 80
Cavia   TAAACAGAATCAAAGACCAGTACAAGGTGTCCGTGCGCATCCCTCCTGACAGTGAGAAGAGCAACCTGATCCGCATTGAG 80
Homo    TAAACAGAATCAAAGACCAGTACAAGGTGTCCGTGCGCATCCCTCCTGACAGTGAGAAGAGCAATTTGATCCGCATCGAG 80

Mus     GGAGACCCACAGGGTGTTCAGCAGGCCAAGCGGGAGCTGCTGGAGCTTGCTTCTCGCATG 140
Rattus  GGAGACCCACAAGGTGTGCAACAGGCCAAGCGGGAGCTGCTGGAGCTTGCTTCTCGCATG 140
Cavia   GGGGACCCGCAGGGCGTGCAGCAGGCCAAGCGGGAACTGTTGGATCTTGCCTCTCGCATG 140
Homo    GGGGACCCACAGGGCGTGCAGCAGGCCAAGCGAGAGCTGCTGGAGCTTGCATCTCGCATG 140
```

ENSMUSG00000034088 exon 11 (ORF 0)

```
Mus     GAAAATGAGCGCACCAAGGATCTAATCATCGAGCAAAGATTTCATCGCACAATCATTGGGCAGAAAGGTGAACGGATTCG 80
Rattus  GAAAATGAGCGCACCAAGGATCTAATCATTGAGCAAAGATTTCATCGCACAATCATTGGGCAGAAAGGTGAACGGATTCG 80
Cavia   GAAAATGAACGTACCAAGGATCTAATCATTGAGCAGAGATTTCATCGCACAATCATTGGGCAGAAAGGTGAACGGATCCG 80
Homo    GAAAATGAGCGTACCAAGGATCTAATCATTGAGCAAAGATTTCATCGCACAATCATTGGGCAGAAGGGTGAACGGATCCG 80

Mus     AGAAATTCGTGACAAATTCCCAGAG 105
Rattus  AGAAATTCGTGACAAATTCCCAGAG 105
Cavia   TGAAATTCGTGATAAATTCCCAGAG 105
Homo    TGAAATTCGTGACAAATTCCCAGAG 105
```

ENSMUSG00000034088_intron_10

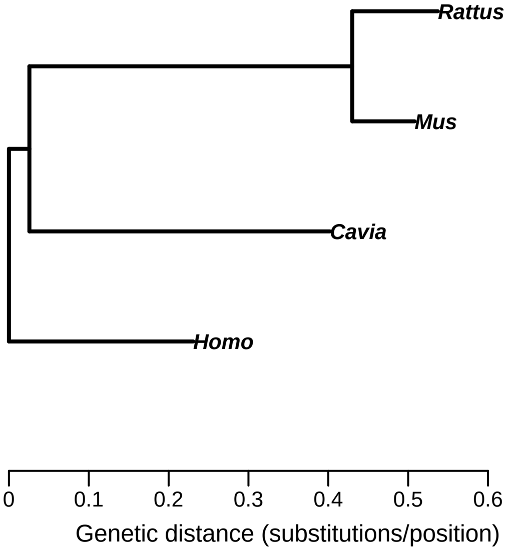

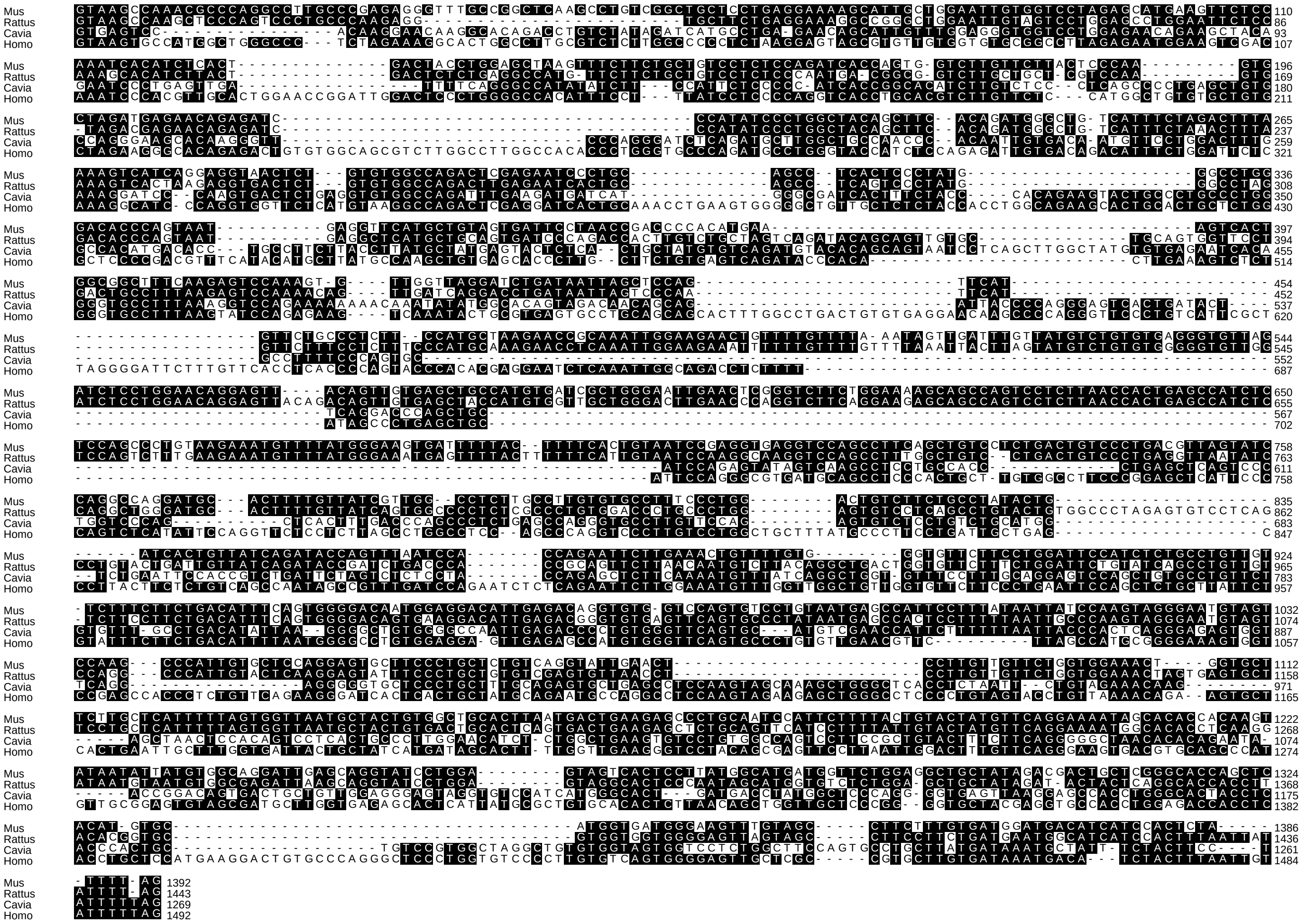
Mus
Rattus
Cavia
Homo

ENSMUSG00000032508 intron 1

Description: Myeloid differentiation primary response protein MyD88 (Myd88)
Intron number: 1
Mouse chromosome: 9
Upstream exon length: 328
Downstream exon length: 135
Mouse intron length: 921
Intron alignment length: 1149
Total murinae branch length: 0.17881
K_score: 0.07655
Scaling factor: 0.78115

ENSMUSG00000032508 exon 1 (ORF 0)

```
Mus     GAGTACTTGGAGATCCGAGAGCTGGAAACGCGCCCTGACCCCACTCGCAGTTTGTTGGATGCCTGGCAGGGGCGCTCTGG 80
Rattus  GAGTACTTGGAGATCCGCGAGTTTGAGACGCGCCCCGATCCCACTCGCAGTTTGTTGGATGCCTGGCAGGGGCGCTCTGG 80
Cavia   GACTACTTGGAGATCAGACAGCTGGAGTCGCGCCTTGACCCCACCGGCAGCCTGCTGGACGCCTGGCAGGGCCGACCTGG 80
Homo    GAGTACTTGGAGATCCGGCAACTGGAGACACAAGCGGACCCCACTGGCAGGCTGCTGGACGCCTGGCAGGGACGCCCTGG 80

Mus     CGCGTCTGTCGGCAGGCTGCTAGAGCTGCTGGCCTTGTTAGACCGTGAGGATATACTGAAGGAGCTGAAGTCGCGCATCG 160
Rattus  CTCGTCGGTCGGCAGGCTGCTAGAGTTGCTAGCCTTGTTAGACCGTGAGGATATACTGTATGAACTGAAGGACCGCATCG 160
Cavia   AGCGTCCGTGGGCCAGCTGCTGGAGCTGCTCGCCAAGCTGGACCGCAACGACGTGCTGTTGGAACTGAGGTCCAGCATCG 160
Homo    CGCCTCTGTAGGCCGACTGCTCGAGCTGCTTACCAAGCTGGGCCGCGACGACGTGCTGCTGGAGCTGGGACCCAGCATTG 160
```

ENSMUSG00000032508 exon 2 (ORF 2)

```
Mus     AGGAGGACTGCCAGAAATACTTAGGTAAGCAGCAGAACCAGGAGTCCGAGAAGCCTTTACAGGTGGCCAGAGTGGAAAGC 80
Rattus  AGGAGGACTGCCAGAAATACATACGCAACCAGCAGAAACAGGAGTCTGAGAAGCCCTTGCAGGTGGCCAGAGTGGAGAGC 80
Cavia   AGGAGGATTGCCAAAAGTACATTTTGAAGCAGCAGCAGGAGGAGTCTGAGAAACCCTTACAAGTGGCCAGAGTGGACAGC 80
Homo    AGGAGGATTGCCAAAAGTATATCTTGAAGCAGCAGCAGGAGGAGGCTGAGAAGCCTTTACAGGTGGCCGCTGTAGACAGC 80

Mus     AGTGTCCCACAAACAAAGGAACTGGGAGGCATCACCACCCTTGATGACCCCCTAG 135
Rattus  AGTGTCCCACAGACAAAGGAACTGGGAGGCATCACCACCCTCGATGACCCCCTGG 135
Cavia   AGTGTCCCACGCACAGAAGAGCCTTTTGGCATCACCACACTGGATGACCCTCTGG 135
Homo    AGTGTCCCACGGACAGCAGAGCTGGCGGGCATCACCACACTTGATGACCCCCTGG 135
```

ENSMUSG00000032508_intron_1

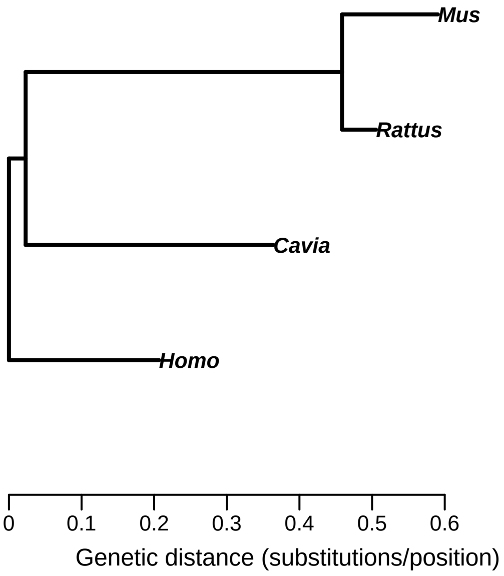

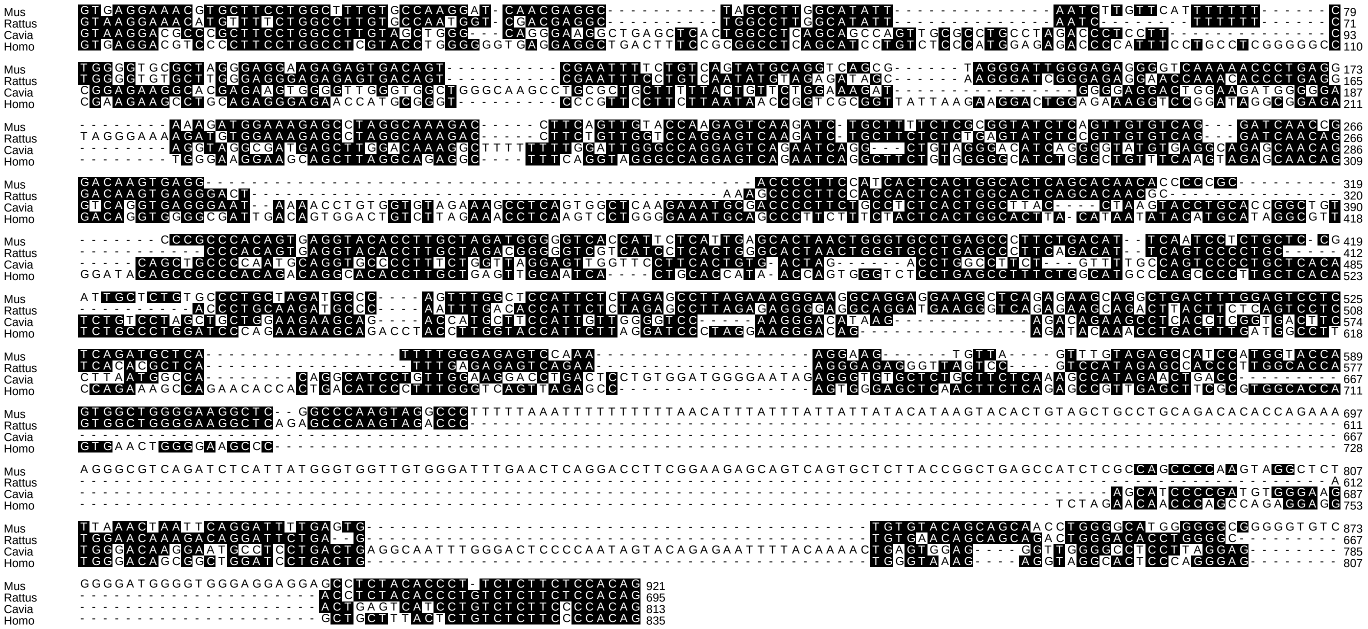
Mus
Rattus
Cavia
Homo

ENSMUSG00000015971 intron 12

Description: Actin-related protein 8 (Actr8)
Intron number: 12
Mouse chromosome: 14
Upstream exon length: 164
Downstream exon length: 144
Mouse intron length: 1205
Intron alignment length: 1981
Total murinae branch length: 0.26340
K_score: 0.05536
Scaling factor: 0.78138

ENSMUSG00000015971 exon 12 (ORF 2)

```
Mus     TGATGATACCAAAAAGAAGATGTATAGCTCTATCCTGGTTGTGGGAGGCGGTTTGATGTTTCATAAGGCTCAAGAGTTTC 80
Rattus  CGACGATACTAAAAAGAAGATGTATAGCTCTATCCTGGTGGTGGGAGGCGGTTTGATGTTTCATAAAGCTCAAGAGTTTC 80
Cavia   TGATGATACCAAAAAGAAGATGTACAGCTCCATCCTGGTGGTGGGAGGTGGTTTGATGTTCCATAAAGCACAAGAATTTC 80
Homo    TGACGACACCAAAAAGAAGATGTACAGCTCCATCCTAGTGGTGGGAGGTGGTTTGATGTTTCATAAAGCTCAAGAATTTC 80

Mus     TCCAGCACAGAATTCTCAACAAAATGCCGCCTTCATTCAGGCGAATTATTGAAAACGTGGATGTGATCACAAGGCCCAAG 160
Rattus  TCCAGCACAGAATTCTCAACAAAATGCCACCTTCATTCAGGCGAATTATTGAAAATGTGGATGTGATCACAAGGCCCAAG 160
Cavia   TCCAGCACAGAATTCTCAACAAAATGCCCCCATCATTTAGGCGAATTATTGAAAATGTAGATGTGATCACAAGGCCCAAG 160
Homo    TGCAGCACAGAATTCTCAACAAAATGCCACCATCCTTCAGGCGAATTATTGAAAATGTGGATGTGATCACAAGGCCTAAG 160
```

ENSMUSG00000015971 exon 13 (ORF 0)

```
Mus     GACATGGACCCCCGGCTGATCGCATGGAAAGGAGGTGCAGTACTGGCGTGTTTGGACACCACTCAGGAACTGTGGATTTA 80
Rattus  GACATGGATCCCCGGCTGATTTCGTGGAAGGGGGGTGCAGTGTTGGCGTGTTTGGACACCACTCAGGAACTGTGGATTTA 80
Cavia   GACATGGATCCCCGGCTGATTGCGTGGAAAGGAGGAGCAGTGCTGGCGTGTTTGGACACGACACAGGAACTGTGGATTTA 80
Homo    GACATGGACCCCCGGCTGATTGCATGGAAAGGAGGGGCAGTGTTGGCTTGTTTGGATACAACACAGGAACTGTGGATTTA 80

Mus     TCAGCGAGAGTGGCAACGCTTTGGTGTCCGAATGTTACGGGAACGAGCTGCCTTTGTGTGGTGA 144
Rattus  CCAGCGAGAGTGGCAACGCTTTGGTGTCCGAATGCTACGGGAACGAGCTGCCTTTGTGTGGTGA 144
Cavia   TCAGCGAGAATGGCAGCGCTTTGGTGTCCGCATGCTGCGAGAGCGGGCTGCTTTTGTGTGG--- 141
Homo    TCAGCGAGAGTGGCAGCGCTTTGGTGTCCGCATGTTACGAGAGCGGGCTGCGTTTGTGTGGTGA 144
```

ENSMUSG00000015971_intron_12

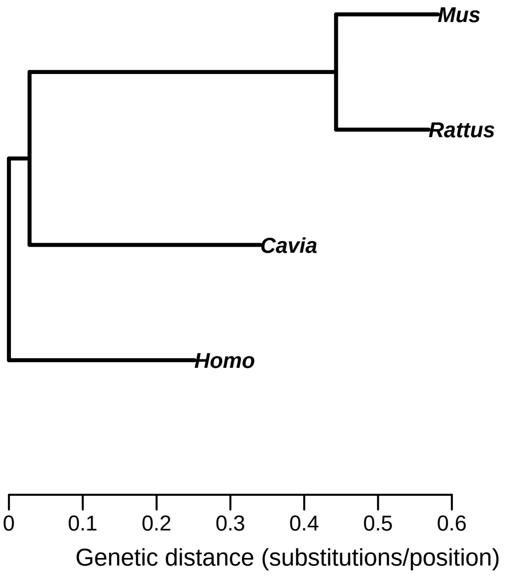

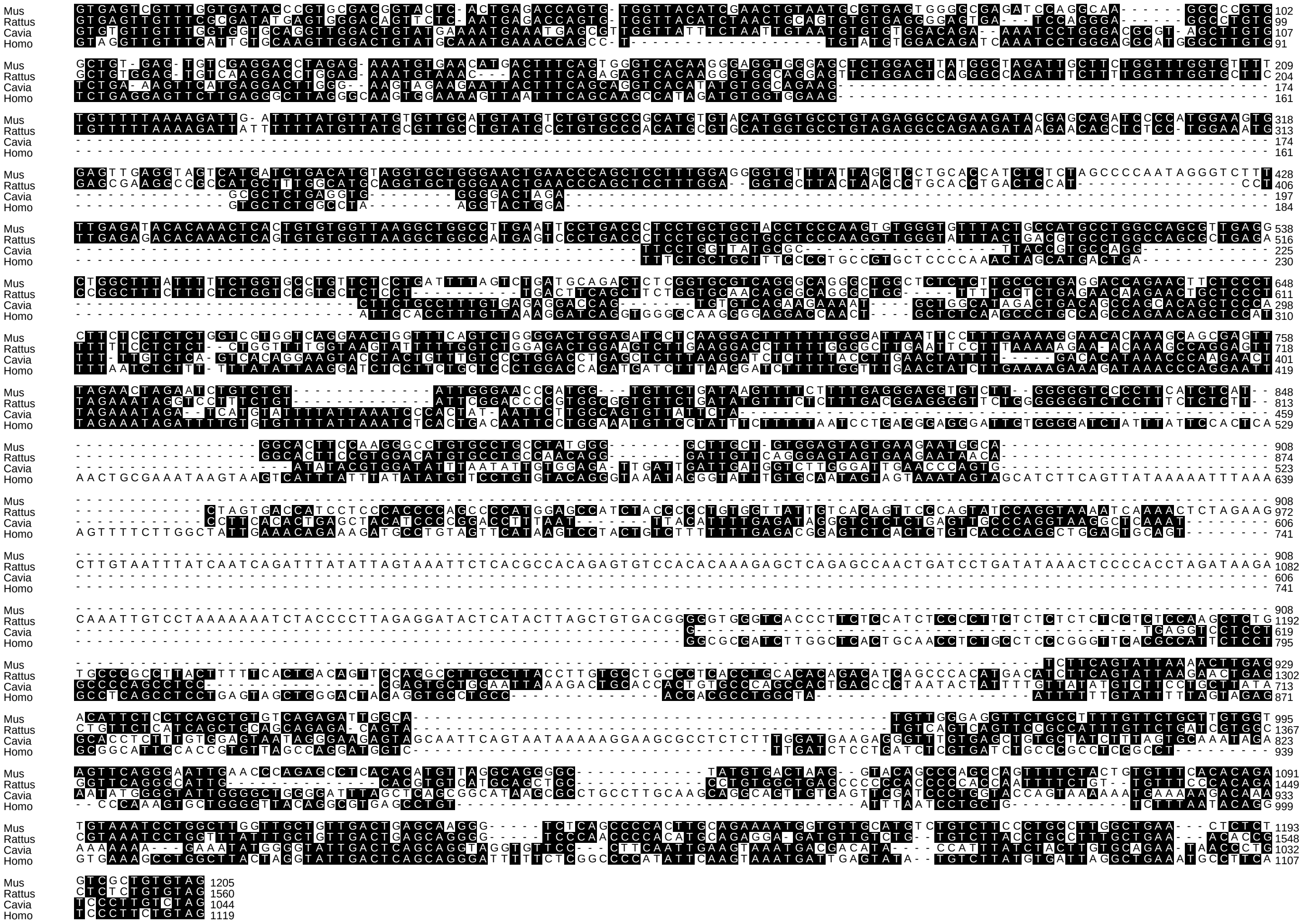
Mus
Rattus
Cavia
Homo

ENSMUSG00000042268 intron 12

Description: Solute carrier family 26 member 9 (Slc26a9)
Intron number: 12
Mouse chromosome: 1
Upstream exon length: 107
Downstream exon length: 70
Mouse intron length: 943
Intron alignment length: 1002
Total murinae branch length: 0.19785
K_score: 0.04491
Scaling factor: 0.7814

ENSMUSG00000042268 exon 12 (ORF 0)

```
Mus     TGTGTCTGGGTGGTGAGCTTCCTCTCCTCGTTCTTCCTGAGCCTGCCCTACGGTGTCGCAGTGGGTGTAGCCTTCTCCAT 80
Rattus  TGCGTCTGGGTGGTGAGCTTCCTCTCCTCCTTCTTCCTGAGCCTGCCCTATGGCGTTGCAGTGGGTGTGGCCTTCTCCAT 80
Cavia   TGCGTCTGGGTGGTGAGCTTCCTCTCCTCCTTCTTCCTGAGCCTGCCATATGGTGTGGCAGTGGGTGTCGCCTTCTCTGT 80
Homo    TGCATCTGGGTAGTGAGCTTCCTCTCCTCCTTCTTCCTCAGCCTGCCCTATGGTGTGGCAGTGGGTGTCGCCTTCTCCGT 80

Mus     CCTGGTTGTGATCTTCCAGACCCAGTT 107
Rattus  CCTGGTTGTGATCTTCCAGACCCAATT 107
Cavia   CCTGGTTGTGGTCTTCCAGACCCAGTT 107
Homo    CCTGGTCGTGGTCTTCCAGACTCAGTT 107
```

ENSMUSG00000042268 exon 13 (ORF 1)

```
Mus     TCGAAATGGCTCCACACTGGCCCAGGTCATGGACACGGACATCTATGTGAACCCCAAGACCTACAACAGG 70
Rattus  TCGAAATGGCTCCACGCTGGCCCAGGTCATGGACACAGACATCTATGTGAACCCCAAGACCTACAACAGG 70
Cavia   TCGGAATGGCTATGTGCTGGCCCGGGTCATGGACACTGACATCTATGTGAACCCCAAGACATACAGTAGG 70
Homo    TCGAAATGGCTATGCACTGGCCCAGGTCATGGACACTGACATTTATGTGAATCCCAAGACCTATAATAGG 70
```

ENSMUSG00000042268_intron_12

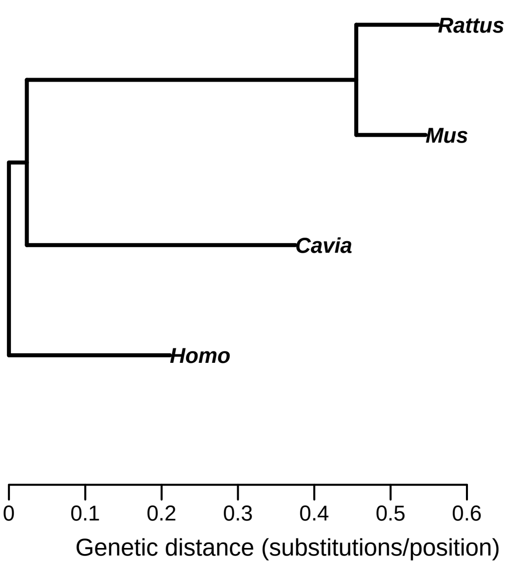

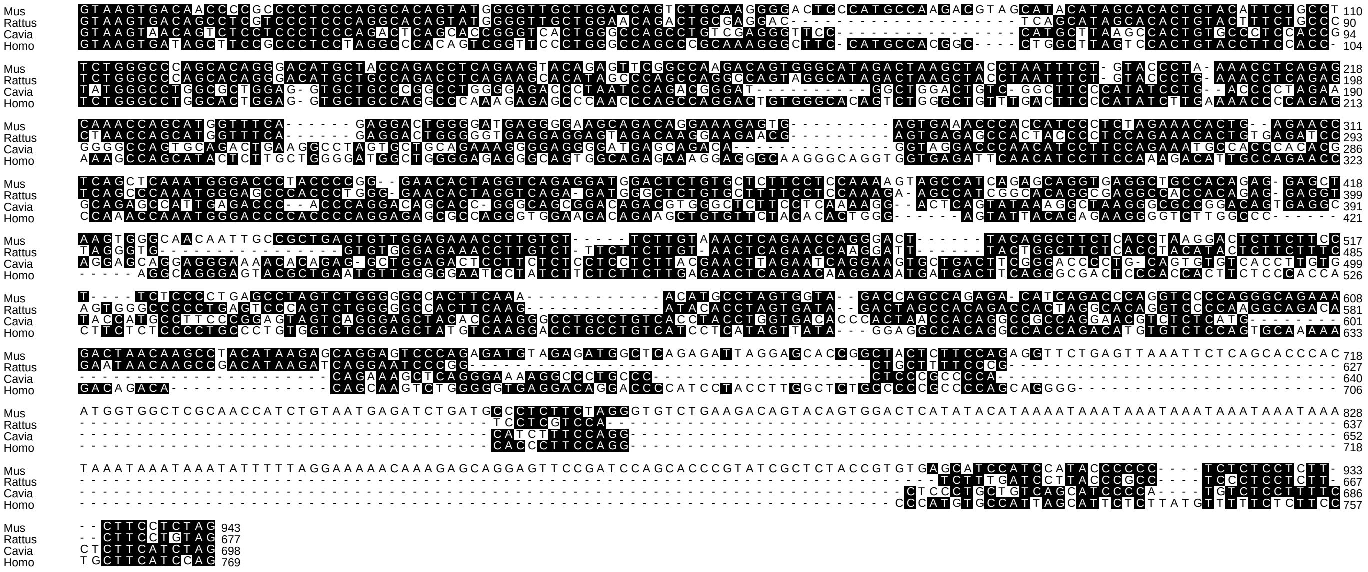
Mus
Rattus
Cavia
Homo
110
90
94
104
218
198
190
213
311
293
286
323
418
399
391
421
517
485
499
526
608
581
601
633
718
627
640
706
828
637
652
718
933
667
686
757
943
677
698
769

ENSMUSG00000024659 intron 9

Description: Annexin A1 (Anxa1)
Intron number: 9
Mouse chromosome: 19
Upstream exon length: 96
Downstream exon length: 59
Mouse intron length: 896
Intron alignment length: 1587
Total murinae branch length: 0.18167
K_score: 0.05399
Scaling factor: 0.78171

ENSMUSG00000024659 exon 9 (ORF 2)

```
Mus     TGTTTCAGAATTACGGAAAGTACAGTCAACATGACATGAACAAAGCTCTGGATCTGGAACTGAAGGGTGACATTGAGAAG 80
Rattus  TGTTTCAGAATTATAGAAAGTACAGTCAACATGACATGAACAAAGCCCTGGATCTGGAACTGAAGGGTGACATTGAGAAG 80
Cavia   TGTTTCAGAAATACACCAAGTACAGTCAGCATGACATGAACAAAGCTCTGGACCTGGAGCTGAAAGGTGACATTGAAAAC 80
Homo    TGTTTCAGAAATACACCAAGTACAGTAAGCATGACATGAACAAAGTTCTGGACCTGGAGTTGAAAGGTGACATTGAGAAA 80

Mus     TGCCTCACAACCATCG 96
Rattus  TGCCTCACAACCATTG 96
Cavia   TGCCTCACAGCCATCG 96
Homo    TGCCTCACAGCTATCG 96
```

ENSMUSG00000024659 exon 10 (ORF 2)

```
Mus     TGAAGTGTGCCACCAGCACTCCAGCTTTCTTTGCCGAGAAGCTGTACGAAGCCATGAAG 59
Rattus  TGAAGTGTGCCACCAGCACTCCAGCTTTCTTTGCTGAGAAACTGTATGAAGCCATGAAG 59
Cavia   TGAAATGTGCCACAAGCACACCTGCTTTCTTTGCTGAGAAGCTTCATCTGGCCATGAAG 59
Homo    TGAAGTGCGCCACAAGCAAACCAGCTTTCTTTGCAGAGAAGCTTCATCAAGCCATGAAA 59
```

ENSMUSG00000024659_intron_9

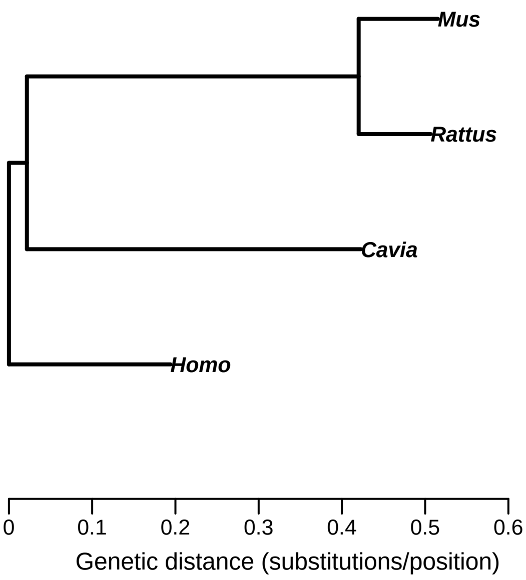

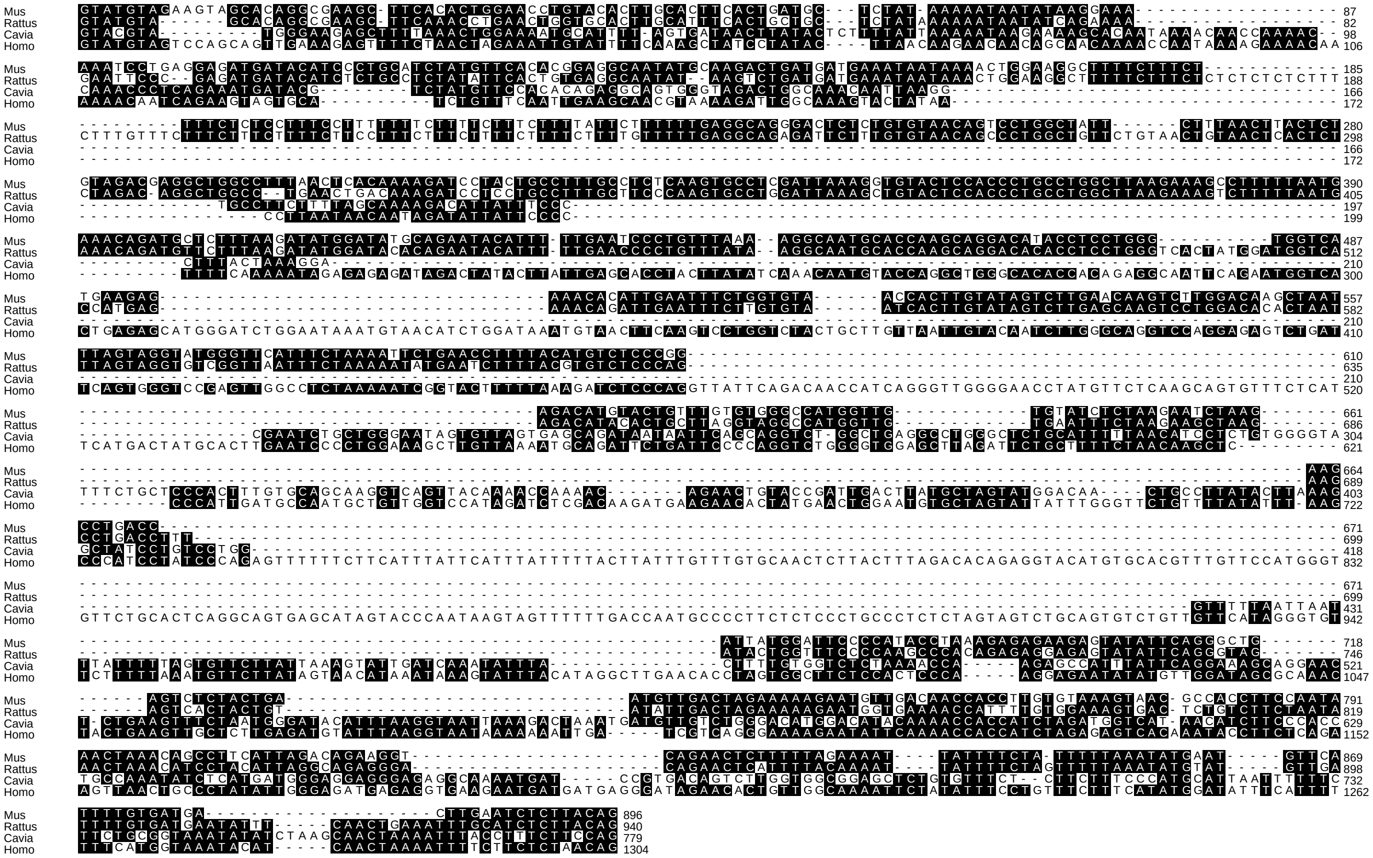
Mus
Rattus
Cavia
Homo

ENSMUSG00000030996 intron 2

Description: GPI-linked NAD (Art1)
Intron number: 2
Mouse chromosome: 7
Upstream exon length: 766
Downstream exon length: 42
Mouse intron length: 1294
Intron alignment length: 1907
Total murinae branch length: 0.21089
K_score: 0.07315
Scaling factor: 0.78193

ENSMUSG00000030996 exon 2 (ORF 0)

```
Mus     ATCAGGGGCTACTCCTTTTTCCCTGAAGAGGAGGAGGTGCTGATCCCTCCTTTCGAAACTTTCCAGGTGATCAATACAAG 80
Rattus  ATCAGGGGCTACTCCTTTTTCCCTGAAGAGGAAGAGGTGCTGATCCCCCCTTTCGAAACTTTCCAGGTGATCAATACCAG 80
Cavia   ATCAGAGGCTACTCCTTCTTCCCTGGAGAGGAAGAGGTGCTGATCCCCCCCTTTGAAACCTTCCAGGTGATAAATGCCAG 80
Homo    ATCAAGGGCTACTCCTTCTTCCCTGGAGAGGAAGAGGTGCTGATCCCCCCCTTTGAGACCTTCCAAGTGATCAATGCCAG 80

Mus     CCGACCCACCCAGGGTCCCGCACGCATCTACCTCCGCGCTCTGGGCAAACGCAGTACATACAACTGTGAATACATCAAAG 160
Rattus  CCGACCCGCCCAGGGTCCTGCACGCATCTACCTCCGTGCTCTGGGCAAACGCAGCACATACAACTGCGAATACATAAAAG 160
Cavia   CCGACCCGCCCAGGGCCCTGCTCGCATCTACCTCCGGGCCCTGGGCAAGCGCAGCATGTACAACTGTGAGTACATCAAAG 160
Homo    CAGACTGGCCCAGGGCCCCGCCCGCATCTACCTCCGAGCCCTGGGCAAGCACAGCACCTACAACTGCGAGTACATCAAAG 160
```

ENSMUSG00000030996 exon 3 (ORF 2)

```
Mus     AAAAGAAGTGCAGGTCTGGGCCCTGCTGGCTGGGTAGCTCAG 42
Rattus  AAAAGAAGTGCAGGTCTGGGCCCTGCTGGCTGAATAGCTCAG 42
Cavia   ACAAGAAGTGCAAATCTGGGCCCTGCCGACTGGATAGCTCAG 42
Homo    ACAAGAAGTGCAAGTCTGGGCCTTGCCATCTGGATAATTCAG 42
```

ENSMUSG00000030996_intron_2

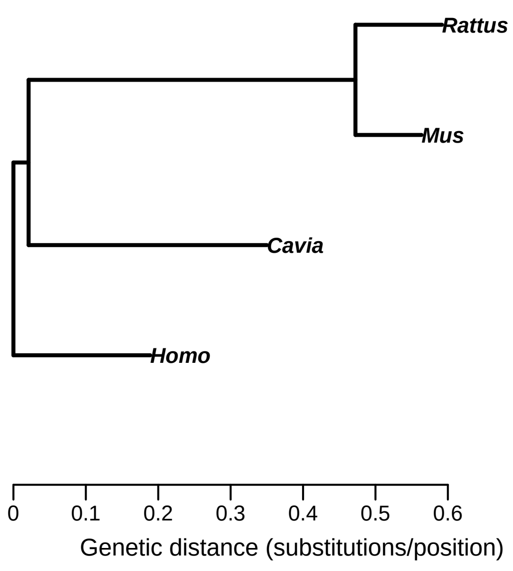

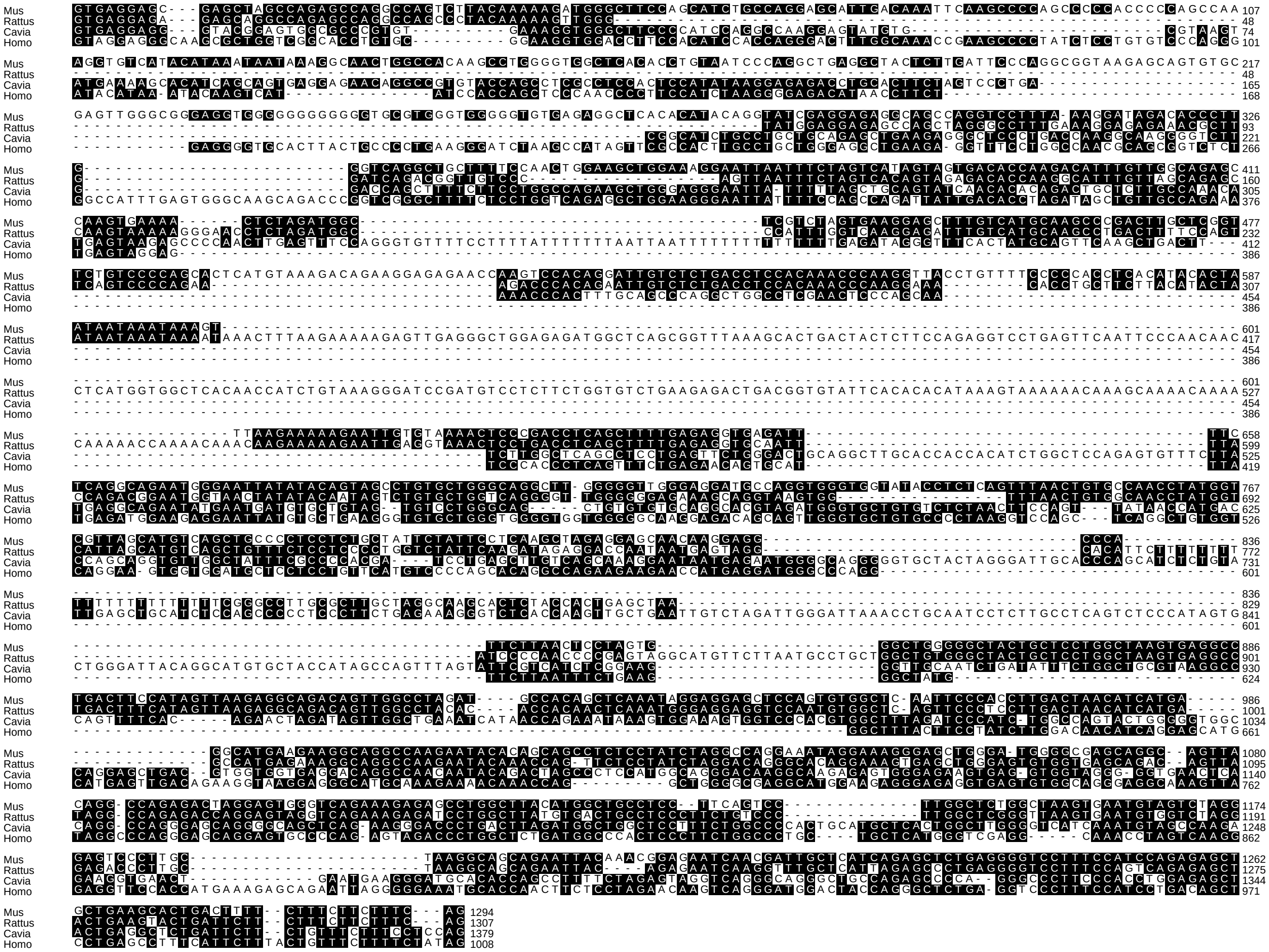
Mus
Rattus
Cavia
Homo

ENSMUSG00000034926 intron 7

Description: 24-dehydrocholesterol reductase Precursor (Dhcr24)
Intron number: 7
Mouse chromosome: 4
Upstream exon length: 198
Downstream exon length: 179
Mouse intron length: 357
Intron alignment length: 454
Total murinae branch length: 0.21426
K_score: 0.05676
Scaling factor: 0.78199

ENSMUSG00000034926 exon 7 (ORF 0)

```
Mus     CCTCTTCGGCTGGATGGTGCCTCCCAAGATCTCCCTCCTGAAGCTGACCCAGGGCGAGACGCTACGCAAGCTGTACGAGC 80
Rattus  CCTCTTTGGCTGGATGGTGCCTCCCAAGATCTCCCTCCTGAAGCTGACCCAGGGCGAGACTCTGCGTAAACTGTATGAAC 80
Cavia   CCTCTTTGGCTGGCTGGTGCCCCCCAAGATCTCACTGCTGAAGCTGACCCAGGGCGAGACCCTGCGCAAGCTCTACGAGC 80
Homo    CCTCTTTGGCTGGATGGTGCCTCCCAAGATCTCCCTCCTGAAGCTGACCCAGGGTGAGACCCTGCGCAAGCTGTACGAGC 80

Mus     AGCACCACGTGGTGCAGGACATGCTGGTGCCCATGAAGTGCATGTCACAGGCCCTGCATACCTTCCAAAATGACATCCAC 160
Rattus  AGCATCATGTGGTACAGGACATGCTGGTGCCCATGAAATGCCTGTCTCAGGCCCTGCATACCTTCCAAAATGACATCCAC 160
Cavia   AGCACCATGTGGTACAGGACATGCTGGTGCCCATGAAGTGTCTGCGGCAGGCCCTGCACACTTTCCACAACGACATCCAT 160
Homo    AGCACCACGTGGTGCAGGACATGCTGGTGCCCATGAAGTGCCTGCAGCAGGCCCTGCACACCTTCCAAAACGACATCCAC 160
```

ENSMUSG00000034926 exon 8 (ORF 0)

```
Mus     GTCTACCCCATCTGGCTGTGCCCATTCATCCTGCCCAGCCAGCCAGGACTAGTGCATCCCAAGGGAGATGAAGCAGAGCT 80
Rattus  GTCTACCCCATCTGGCTGTGCCCGTTCATCCTGCCCAGCCAGCCAGGACTTGTGCATCCCAAGGGAGATGAGGCTGAGCT 80
Cavia   GTCTACCCCATCTGGCTGTGCCCATTCATCCTGCCCAGCCAGCCAGGCCTGGTGCACCCCAAGGGAGATGAGACTGAGCT 80
Homo    GTCTACCCCATCTGGCTGTGTCCGTTCATCCTGCCCAGCCAGCCAGGCCTAGTGCACCCCAAAGGAAATGAGGCAGAGCT 80

Mus     CTACGTGGACATCGGGGCATACGGGGAGCCACGTGTGAAGCACTTCGAGGCCAGGTCCTGCATGAGGCAGCTGGAGAAGT 160
Rattus  CTACGTGGACATCGGGGCATATGGGGAGCCACGGGTGAAGCACTTCGAGGCCAGGTCCTGCATGAGGCAGCTGGAGAAGT 160
Cavia   CTATGTGGACATCGGCGCCTATGGAGAGCCACGTGTGAAGCACTTCGAGGCCAGGTCCTGCATGCGGCAGCTGGAGAAGT 160
Homo    CTACATCGACATTGGAGCATATGGGGAGCCGCGTGTGAAACACTTTGAAGCCAGGTCCTGCATGAGGCAGCTGGAGAAGT 160
```

ENSMUSG00000034926_intron_7

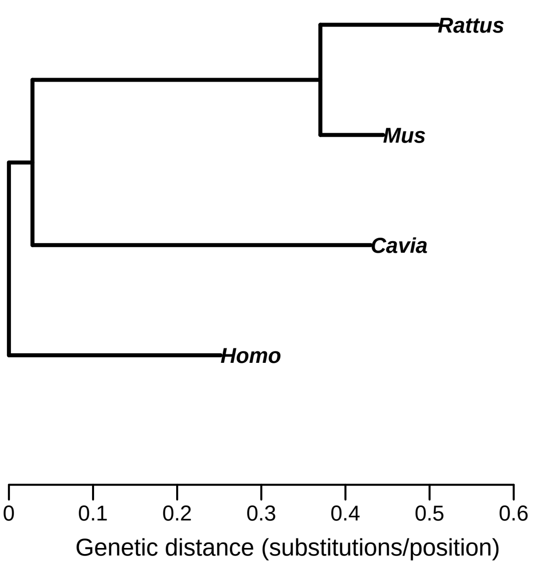

```
Mus     GTAAGCTGGGTGACCAGGCGGGCGTGG------------------TCCCATGGAACCCA-GGTCTTCTCCCTAGCTCTGTGTCGCCCACCCATC--TGTGTATCTGCCCT 89
Rattus  GTAAGCTGAGTGTCCAAGTGGGCATGG------------------TCCCATGGAACCCA-GGCCTTCTCCCTTGAGCTGTGTCACCCACCCATCTTTGTGTATCCGCCCT 91
Cavia   GTGAGTGGC-------AGCAGGCGTGG-------------------CCCCAGGACTTCA-CAGCCTCTCCCCTGAGCC-CACAGCCCACCTGCT-GGGCCTGTCTGCCCT 81
Homo    GTGAGTGGG-------GCAGGGCAGGGAGGGCGCACCAGGTGCATTCCCTTGGAATCCATGGTCTCCTCCCCTGAACCGTGTGACCGAC--CTT--GGGCACACTGCCCT 99

Mus     CTGGGCCTTGGTTTCTCCAACTATACAATTAGGATCTT----------------------AGCCCACCCAGGAACTGT-------------------------------- 145
Rattus  CTGGGCCTCAGTTTCTCCAGCTGTATGATTAGGGTCTT-----------------------GCCTGCTCTGGGACCAT-------------------------------- 146
Cavia   CTCGGCCTCAGTTTTTCCAACTGTAAAGTCAGGCTTTGTCCCTATTACTGCAGAGGCTTCAGTC-ACTTGGCAGTGGC-------------------------CCTGGTG 166
Homo    CTGGGT----GTTTTCCCAACTTTAAGATGAGGTCCTTGTCCCTATGTCTGCTGAGGCCTCAACCCACTCAGTGATGGCCCCCCTGGGAGAGGAGTCATAGGGTCTGGCA 205

Mus     ---AGTACCCTGATAAGCTGCTTTAGAGTGGCCCCGTAATATGGAAGTATTTGG-----------CAGGGCTGGAG---GGAGAACTGAAGCTGGAAGCAAGCACCTGGG 238
Rattus  ---AGTGCCCTGATAAGCTGCTTCTGAGTGGCCCCATGATATGGGGGTATCTAG-----------CAGGACTGCAG---GGGGAGCTGAAGCTAGGAG------------ 227
Cavia   ACAAGCACCTCTGTGAACAGCTCCTGTG---------------------------------------------------GGGGCATCAGGGCTGGTAACT---------- 215
Homo    TCGAGCACTCCCATGACCTGCTCCGATTGTGTCCTCCAAGGTGGTGGAGTCAGGGTGTTCTCAGCCAGGGCTGGGGGGCGGGGCAGTGAAGCTGAGAGCA---------- 305

Mus     ACCCTCTAGTGGGTTACAAAGT----CAAAGTTCCCGTTTGCAAGGATGTGGGTGAGCTAGTCCCGGGTCTGCTACCTGCT-GGGAAAAGACAGACCAAGCTGTTTCTCT 343
Rattus  --CCTCTAGTGAGTTACAAAGT-------------TCCTTTGTGGGATGTGGATGATTTAGTCCTGTGTCTGCTGCCTGCT-GAGAAAAAATAGACCAACCTGTTTCTCT 321
Cavia   --CATGCGTTGGGCAGCAGAGC-------AGGGCCCCTGTGGGAGGGTGGAAG-----------------TGTCACCTGCCCAAGAAGAGGCACCATTGCCCACCTGGCC 299
Homo    --CCTGAGGGGGTCTTGAGAATGCTGCAAAGCCTTCATTTGGGTGGGTATGAGCGGGCCAGCCATGAGCCTGTCACCTGTTGGAGAAAAAGCACCAATACCCACCTCCCT 413

Mus     GACCTCTCACTCAG 357
Rattus  GACCTCCC----AG 331
Cavia   GGCCTCCCC---AG 310
Homo    GGTCTACCC---AG 424
```

ENSMUSG00000042477 intron 5

Description: Transcription factor AP-2 epsilon (Tcfap2e)
Intron number: 5
Mouse chromosome: 4
Upstream exon length: 119
Downstream exon length: 142
Mouse intron length: 932
Intron alignment length: 1073
Total murinae branch length: 0.28969
K_score: 0.07796
Scaling factor: 0.78243

ENSMUSG00000042477 exon 5 (ORF 1)

```
Mus     GGCCAAGTCCAAAAATGGAGGCCGGTGTCTGCGGGAACGGCTGGAGAAGATTGGGCTCAACTTGCCAGCTGGTCGTCGGA 80
Rattus  GGCCAAGTCCAAGAATGGGGGCCGCTGTCTGCGGGAACGGCTGGAGAAGATCGGGCTCAACCTGCCAGCTGGGCGACGGA 80
Cavia   GGCCAAGTCCAAGAATGGGGGCCGGTGCCTGCGGGAACGGCTGGAGAAGATTGGGCTTAACCTCCCAGCAGGCCGGCGGA 80
Homo    GGCCAAGTCCAAAAATGGGGGCCGGTGTTTGCGGGAACGGTTAGAGAAGATTGGGCTCAACCTGCCAGCTGGCCGTCGCA 80

Mus     AGGCAGCCAACGTGACTCTGCTGACCTCGCTGGTGGAAG 119
Rattus  AGGCCGCCAACGTGACTCTGCTGACCTCACTGGTGGAGG 119
Cavia   AGGCTGCCAATGTGACACTGCTGACTTCGCTGGTGGAGG 119
Homo    AGGCCGCCAATGTGACGCTGCTGACTTCGCTAGTGGAAG 119
```

ENSMUSG00000042477 exon 6 (ORF 2)

```
Mus     GAGAAGCTGTGCACCTGGCTCGGGACTTTGGTTACGTTTGTGAGACTGAGTTTCCAGCCAAGGCGGCTGCTGAGTACCTA 80
Rattus  GAGAAGCTGTGCACCTGGCTCGGGACTTTGGTTATGTCTGTGAGACTGAGTTTCCAGCCAAGGCGGCTGCTGAGTACCTA 80
Cavia   GAGAGGCTGTGCACCTGGCCAGAGACTTCGGCTATGTCTGCGAGACTGAATTCCCAGCCAAAGCAGCCGCTGAGTACCTG 80
Homo    GAGAGGCCGTGCACCTGGCCCGAGACTTCGGTTACGTCTGTGAGACGGAGTTCCCAGCCAAGGCAGCTGCCGAGTACCTG 80

Mus     TGCCGACAGCATGCCGACCCTGGGGAGCTACACAGCCGCAAGAGCATGCTGCTGGCTGCCAA 142
Rattus  GGCCGACAGCACGCTGACCCTGGGGAACTGCACAGCCGCAAGAGCATGCTGCTGGCTGCCAA 142
Cavia   TGCCGACAGCATGCTGACCCTGGGGAGTTGCACAGCCGCAAGAACATGCTGCTGGCTGCCAA 142
Homo    TGCCGACAGCACGCTGACCCGGGGGAGCTGCACAGCCGCAAGAGCATGCTGCTGGCTGCCAA 142
```

ENSMUSG00000042477_intron_5

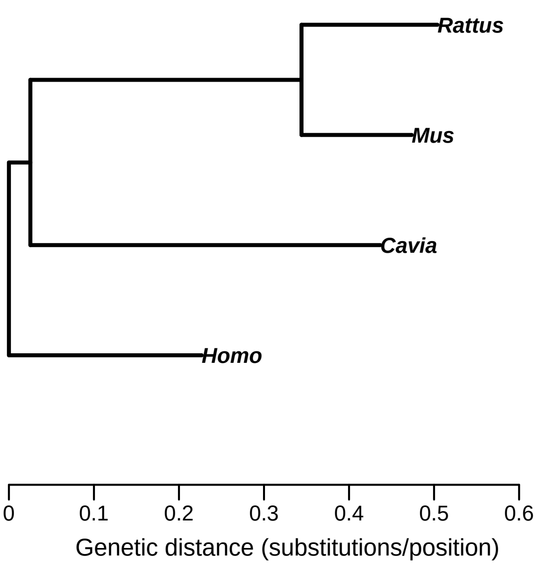

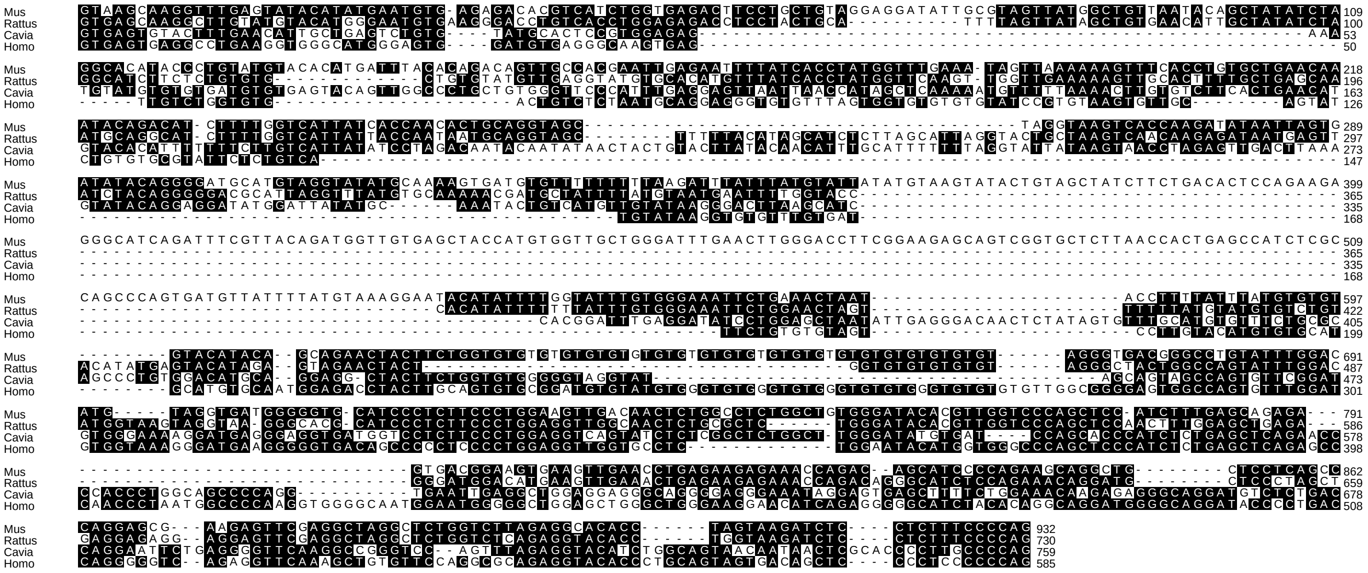
Mus
Rattus
Cavia
Homo

ENSMUSG00000033029 intron 1

Description: UPF0193 protein EVG1 homolog (1700088E04Rik)
Intron number: 1
Mouse chromosome: 15
Upstream exon length: 103
Downstream exon length: 63
Mouse intron length: 1580
Intron alignment length: 3231
Total murinae branch length: 0.20404
K_score: 0.05034
Scaling factor: 0.78276

ENSMUSG00000033029 exon 1 (ORF 0)

```
Mus     ATGGCTTCCCAGGAAAGGGTGGACTCGGTGACCAAAGGAACCGGGTTCCGCCGCTGCCGTAAGCAAGCAGGTTACACGCC 80
Rattus  ATGGCTTCACAGGAGAGGGTGGACGCAGTGGCAAACGGAACCGGGTTCCACCGCTGCCGTAAGCAAGCCGCTTACACGCC 80
Cavia   ATGGCTTCGAAGGACAGGGTGGAGACTGTGACGACAAGAACAGGGTTTGGGTGCTGCCCCAAGCCGGCCGCTTACACCCC 80
Homo    ATGGCTTCACAGAAGCAGATGGAGGTAGTGACCAAAGGAACTGGGTTCCGGCGCCGCCCCAAGACCATCACTTACACCCC 80

Mus     GGGGACTTGTGAGCTGCTCAGAG 103
Rattus  GGGGACCTGTGAGCTTCTCAGAG 103
Cavia   AGGAACCCGCGATCTGCTCAAAG 103
Homo    GGGGACCTGCGAGCTGCTCAGAG 103
```

ENSMUSG00000033029 exon 2 (ORF 2)

```
Mus     TTATGATGAAGGAGTCTAAACTGACAAACTTCCAGCAACGCCACATCATGGACACCATGAAAA 63
Rattus  TGATGATGAAGGAGTCCAAACTGACAAACTTCCAGCAACGCCATATCATGGACACCATGAAAA 63
Cavia   CTATGATGAAGGAATCGAAGCTCACTAACTTCCAGCAGCGCCACATCATGGACACCATAAAAA 63
Homo    TGATGATGAAGGAATCCAAACTGACGAACATCCAGCAGCGCCACATCATGGACATCATGAAAA 63
```

ENSMUSG00000033029_intron_1

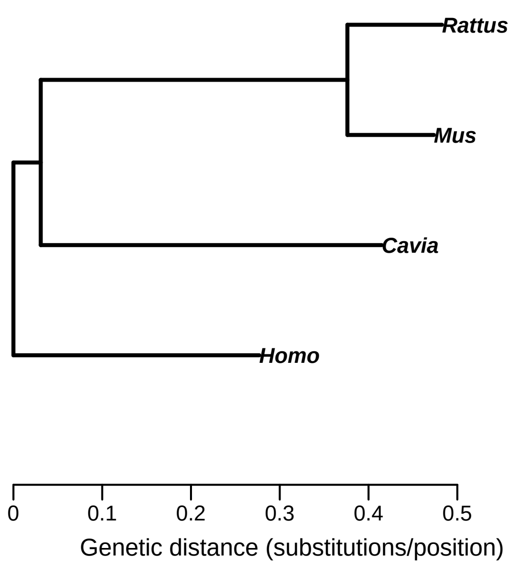

```
Mus     GTCAGTGCATTAGGG-----------------------------------------------------------------------------------------------  15
Rattus  GTTAGTGCATTAGCG-----------------------------------------------------------------------------------------------  15
Cavia   GTCAGTGGACTA--------------------------------------------------------------------------------------------------  12
Homo    GTCAGTCCGTTAAAGCAGCGATCCCAGCCTTTTTGGCACCAGGGACCGGTTTCGTGGAAGACAATTTTTCCACTGACGGAGGTGTGGGGAATGGTTTCGGGATGAAACTG  110

Mus     --------------------------------------------------------------------------------------------------------------  15
Rattus  --------------------------------------------------------------------------------------------------------------  15
Cavia   --------------------------------------------------------------------------------------------------------------  12
Homo    TTCCACCTCAGATCATCAGGTATTAGTTAGATTCTCATAAGGAGCGTGCAACCTAGATCCCTCGCGTGCGCAGTTCACAATAGGGTTCGCGCTCCTATGAGAATCTAAGG  220

Mus     --------------------------------------------------------------------------------------------------------------  15
Rattus  --------------------------------------------------------------------------------------------------------------  15
Cavia   -----------------------------------------------------------------------------------------------------------GAG  15
Homo    CCGTCACTGACCTGAAAGGAGGCGGTGAGGCTCGCTAGCCTGCCGCTCACCTCCTGCTGTGCGGCCCGGTTCCTAACAGGCCAGGGACTGGTACCAGTCCACGGACGGAG  330

Mus     ----------------------------------------------------------------------------GATCAACGACCCGAGCGGTGCGGTTTTTCCTT--  47
Rattus  ----------------------------------------------------------------------------GACCAAAGACTTGAGCGGTGCGGTTTTCCCTT--  47
Cavia   GGCTAGGAGTGTACAGAACGCAGGAGAGGAGTGG--CCATAACAGCCTCCTGTGTAAACTCAGTTCGCTCCTACCTGTCCAGTGGCTTGGGAATGGCTGTTTCCCCTCAC  123
Homo    GGTTGGGGACCCCTGAGTTAAAGGATGAGAGAAGAATGGTGGTAGTTACCCTCTGTAACTCAGTTCCCCGTACCTGACCTGTGGCTT----AGCACTATTTTCCTTTAC  436

Mus     -----AAGAGCTTGGGA--TTCTGAAAGGTTC--------------------------------------------------GGGGAAGAATC-----------------  83
Rattus  -----AAGAGCTTGGGA--TTCAGAAGGGTTC--------------------------------------------------TGGGGAGAATC-----------------  83
Cavia   AAATAAGGAGTTCAGAAAGTTCAGAATATTGC--------------------------------------------------------------------CTATCCTGAC  165
Homo    AGATAAGGAGCCTGGGG--TTCAGAAAGCTTCAAAATTCTACTGCATATGCCTTATTCGGGGGCGGGGCGGGGCGGGGGGGAGGGGAAGGTTCAAGGTGCTACACCTTGC  544

Mus     --AAAGACCAGAGATGGAGAATTTTGGAATGGTTAGT-------------------------------------------------------------------------  118
Rattus  --AAAGGCCAGAGATGGAGAATTTTGGAATGGTTAGC-------------------------------------------------------------------------  118
Cavia   CAAAAGTCAAAGCGCTAAGAATTTTGGAATAGTTAACGCCTACTTTTTTTTTTTTTTTTTTTTTGATAGCAGGAATCCAGCTGATAATCTCACACTTAGCAATACTTTTC  275
Homo    CAAAAGTCAAAGAACTAAAATTTTTAGAGTAGTTAAC-------------------------------------------------------------------------  581

Mus     --------------------------------------------------------------------------------------------------------------  118
Rattus  --------------------------------------------------------------------------------------------------------------  118
Cavia   TTTCTTTCTTTTTTTTTTTTTTTTTGGGGGGGGGTACCAAGGTTTGAATTCAGGACCTCACACTTGCTAGGCAGGTGCTTATGCTGCTGAGCTAAATCCCCAACCACCCC  385
Homo    --------------------------------------------------------------------------------------------------------------  581

Mus     --------------------------------------------------------------------------------------------------------------  118
Rattus  --------------------------------------------------------------------------------------------------------------  118
Cavia   TCCTTTTTGAAACAAGTTTTCACCTTGTAGTCCCAGCTGGTCTCAAACTTGCGGTCTTCCTGCCTCAGCTTCCCAAGTGCTGGGATCATAGGTATATGCTAGGGTCCAAT  495
Homo    --------------------------------------------------------------------------------------------------------------  581

Mus     -------TCATCTTTTACAT----------GCACTTTCAATGAAGC------------------------------------------CTATCTCCT---CACTGCAGTG  166
Rattus  -------TCATCTTTTACGT----------GCACTTTCAATGAAGC------------------------------------------CTATCTCCTTCCCACCACAGTA  169
Cavia   TTTCTGTGAATCCTTTACAATACAGTGTCAGCATTCCCACAGAAACTTTGTCTCCTACCCTTCCCCTTCTGCCTCCCTCTCTTCCTCTTCCTCCTATCATCACCACAGGG  605
Homo    -TCCTATGCATCCTTTAGATCACACTTTCAACTTTCCCGAGGAAGA----------------------------------------CTTCTGCTCCTCACCACCACAGTA  650

Mus     GGACC------------------TTCACAGTACCAAATTCAATTCCCTTCACCAGGCTTAG-ACATTATACAGTCTTAGAGGTGTTTAGTGTCAC---------------  242
Rattus  GGACC------------------TTCACAGTACCACATTCAACTCCCTTCACCGGGTTTAGCATAATATATAGTCTTACAGGTGTTTAGTGTCCCAGCTATCAT------  255
Cavia   GGACCAGCTGTTACATGGCCATCTGCACACTACCGAATACATTTCCTTTCACTGGGCTCAGAACAATGTACACTT----AGGCATTTTGTGTCACATTTGCCCCCTAGAA  711
Homo    GGATCAACTGTTATGTGC--------------CCTCACACATTTTCCTTCACTGGGCTTAGCACAAGCTGTAGTC----AGTTATTTGGTGTCCCATCTATC--------  734

Mus     --------------------------------------------------------------------------------------------------------------  242
Rattus  --------------------------------------------------------------------------------------------------------------  255
Cavia   GTTGAATTCTTTCTCTTTTTTTGGGGGGGGGGGGCAGGGTCTCACTGTGTAGTTCAGGGTGACTTTGAACTCACTGTGGAGTGCAAGCTGGCCTTGAACTCACAGAAATC  821
Homo    --------------------------------------------------------------------------------------------------------------  734

Mus     --------------------------------------------------------------------------------------------------------------  242
Rattus  --------------------------------------------------------------------------------------------------------------  255
Cavia   CTCCTGCCTCAGCCTCCCACATGCTAGGATTCAGGCATGCACCACCATATCTGGCCATTTTTTCTTTTTTTCCAGATAAGTTCTCCCCATGTAGTTCAGGCTGTGCTTGA  931
Homo    --------------------------------------------------------------------------------------------------------------  734

Mus     -------------------------------------------------------------------------------------------------CTTCTAAATGGTG  255
Rattus  -------------------------------------------------------------------------------------------------CTTCTAAATGGTG  268
Cavia   ACTCACTATGGAGCCCAAACTGGCCTTGAAATTGCAGTGATCCTCCTGCCTCAACCTCCCGAAAGCTAAGATTACAGGCATGTGCCCCCACACCTGGCTCCTAAATGATT  1041
Homo    -------------------------------------------------------------------------------------------------CCTCCAGATCATG  747

Mus     AACACCACCAGGCAAAGAAACGTGCCTGTTTCTGCTCACAACTGTAGCTCCAGCCTCTGCTAC---ATGAATGAGCATGTATGCACTTAATTATTCAACGAC--------  354
Rattus  AATGCCACCAGGCAAAG-AACGAGCCAGCTTCCGCACACAATTGTAGCTCCAGACTCTGCTAT---GTGAATGAACCTGCATGCATTTAATTATTCAACGAC--------  366
Cavia   AATTCTATCAGGCAGG--ACTGTGACTGTTTCTACTCATAACTGTAGCTTCTACCTAGGCAC----ATGAGTGAATAAATATTCCTTTAATCAGATAAAAAA--------  1137
Homo    AATTTCATCAGGCAGGA-ACCGTGCCTGTTTCGGCCCACAATTGTAGCTCCAGCCCCTGAAACATGATGAACAAGTATTCATGTGTTCTTTTACTCAACAACTTTTTTGT  856

Mus     ---------------------AAAAACCTAATGTGTGATTTGGATATTGTCCTAGGTACTAGGAATAGAGTGTTGAGAGGATTGCAAAGGACAACTCTTGCTCTCAGAAA  443
Rattus  --------------------TAAAAACCCAATATATGATTTGGATATTGTCCTAGGTATTCGGAATAGAGTGTTGAGAGGATTGCGAAAGCCAATTCTTGCTCTTATAGA  456
Cavia   ------------------TGTTTAAATCTACCATGTGCTTTGGGTACT----TGGGGACAGCAATGGAAAAACAGAAAACTGCCAAAG-----TTCTTGCTGTTACAGA  1220
Homo    TTTTTTCCAACAACAACATTTTTAAGTCTACCATGTGCCTTAGACTCTGTTTTAGGTGCTGGGGATACAGCAGTGAAAGAA-----------------------------  937

Mus     ACTGATAACACAGGAGGGTAACAAGAAAAATGAACAGGAGAAAATTATAGAGCTGAAG----------------GTGTTGCTCAGTGACAGAGTATATTCTAGGCAGATG  537
Rattus  GCTAATAGTACAGGAGGGTAACAAGAAAAATTAACAATAGAAAATTATAGAGCTGG-G----------------GTGTCACTCAGTGATAGAGTATATTCTAGGCAGATG  549
Cavia   GCTAATATTAGAGTAGGG-----------AATAAGAGTAAAAATACGTAGGACTGGCTACGTATTTTTATTTTAGAATAACTCAGAAGTAGAGTACGTGCTAACATGCCT  1319
Homo    -----------AATGAGGTGGCTCAAACCGGTAATCCCAGCACTTTGGGAGGCCAAGG--------------------------TGGCAGATCACCT-------------  998

Mus     TGGAGTCTGGGTTTCAGCCCCAGCACACCATACACA----AAAAAGTCTGTGTGTGTTACAGTTGGAGAGCTGGCTCA--GTGGTTAGGAACATGTACTGCCCTTTGAAA  641
Rattus  TGAAGTCTGGATTTCAGTCCCAGCACACCACACGCC----AAAATAAGTGTGTGTGTTACAGTTGGAGAGATGGCTCA--GTGGTTAAGAGCATTTACTACTCTTTGAAA  653
Cavia   GAGAACCTAGGTTCAATCCCCAGCACCACACATACACATCAAAAATAAAATATTTTTTAAAA-TGGAACCTTAGCCAGTCATGGTGGCATACTCCTGTAATC--------  1420
Homo    -GAGGTTAGGAGTTCACGACCAGCCTGACCAACATG---GAGAAACCCAGTCTCTACTAAAAGTACAAAATTAGCCGGGCGTGGTGGCGCATGTCTGTAATC--------  1096

Mus     GCTCATTCCCCAGCACCCATGTTAGCCAGTCACCAAAAAAGTGTGTG--TGTCTTAGGCATATGTAA--------------------TGGGAGGATTGCC-TGAGCCCAA  728
Rattus  GTTCAGTCCCCAGCACCCATGTTAGCCAGTCACCAAAAAAGTGTGTGTTTGTCTTAGGCAGATATAAAGCTATGCAAGCTTACTACTTGGGAGGATCGCC-TGAGCCCAA  762
Cavia   -------------------------CCAG-CACTCAGGAGACTGAGG----------------------------------------CAGGAGGATCATTGTGAGTTTAA  1464
Homo    -------------------------CCAGCTACTTGGGAGGCTGAGG----------------------------------------CAGGAGAATTCCT-TGAACCCAG  1140

Mus     GA-------------------------AAAGCTAGCCTAGATAATATAGTAAGAACCTATCCTTGAAATAAAATAAGGACTGGAAAGATGGCTCAGAGATTAAGAGCACT  813
Rattus  GA-------------------------AAAGCGAGTCTGTATAATATAGTAAGAACCTATCCT-----TAATATAAGGGTTGGAGAGATGGCTCAGAGATTAAGAGCACT  842
Cavia   GGCCAACCTGGGCTACAAAGTAAGTTCAAGTCCAGCCTGAACCGCCCAGGGAGACCCTGTCTCAAAAAGAAAGAAAATGTT------------------------ACTGT  1550
Homo    GA---------------------------------------------------------------------GGTGGAGATTG----------------------------  1153

Mus     G---GCTATTCTCAAGGACCCAAGTTCAATC-----------------------------------CCCCACAACCATATCAGGTGGGTCAAAGCAATGTGTAACTCTAG  885
Rattus  GACTGCTAGTCTCAAGGACCCAAGTTCAATT-----------------------------------CCCTACAACCATGTCAGGTGGGCCAAAACAGTCTATAACTCTAG  917
Cavia   AGCTCTTCCTGTAAGCAACCTGAGGTCACCTGTGAAAATGGCTTGCTACTCTCTTGACCCAGAAAACCCCACA---AAATCATGCAAATCAAGATGCTCAAATCTTCGTG  1657
Homo    ------------CAGTGAGCTGAGGTCGCGC-----------------------------------CACTGCACTCCAGCCTGG--------------------------  1190

Mus     CTTCCTATGAG----TCAGTGCC-TTTCCCTGCCCTCCACAGGCAGCCATACACGCATAGCTACACACTCACGCACGCGCGCGCGCACACACACACACACACACACACAC  990
Rattus  CTTCCTATGAT----TCAGTGCCTTTTTCTGGCCTTCACAGACACCCATACACACAT------ACATTCACACACGCGCTCTCTCTCTGTCTCTCTCTCTCTCTCACTC  1017
Cavia   TTCACTTTAAGAACATCTGTGAAACTGCCCAGGCCATGAAGGATACGTATATAGGAAGGCCACCAAATATCTGAAGG---------------------------------  1734
Homo    --------------------------------------------------------------------------------------------------------------  1190

Mus     ACACACGACCTTTTATTTTTTAAGATTTGTTTGTTTATTTATTATGGATAGTGTTCGGCCTGCATGTATGCCTGCAGGCCAGAAGAGGGCACCAGATCCCATTACAGATG  1100
Rattus  TCTCTCTCTCTCTTTTTCTTTTTTCTTTTTTT------------------------------------------------------------------------------  1049
Cavia   ---------ATGTCACATTAGAGAAGCAATGTGTGCCACTCTGGCGTTACGATGGTGGAGTTGGTAGGTTAGCTCGGGCCAAGTGATGGGGCTGGACCCAGGTCCGTGG  1835
Homo    --------------------------------------------------------------------------------------------------------------  1190

Mus     GTTGTGAGCTTCCACGTGGTTGTTG--------------------------------------GGAATTGAACTCAGGACCTCT----------GGAAGAGCATCATATA  1162
Rattus  ----------------TCGGAGCTG--------------------------------------GGGACCGAACCCAGGGCCTTCCTCTTGCTAGGCAAGTGCTCTACCAC  1105
Cavia   CCCAAAGAGTGCTGAATTTTTGCTGCGCGTGTTTAAAAATGCAGAGAGTAATGCTGAACCGAAGGGTTTAGATGTAGATTCTCTGTTACTGAATATAACCAGGTGAACAA  1945
Homo    ---------------------------------------------------------------GCAACAAGATGGAAGCTCCATCTCA----------------------  1215

Mus     TGCTCTTG-------------------------------------------ATATGTGAGTCATCTCTCCAGCCCACATACAATCTT-----------------------  1206
Rattus  TGAGCTAA-------------------------------------------ATCCCCAACCCCTCTCTCTCTCTC--------TCTT-----------------------  1141
Cavia   AGCACCCAAGATGTGCCGCTGTACTTACAGAGCTCAGGGCCAGATTCACCCACACATGAGCTCCCCTGCCACAGCGAGAGGATCCTTCCTGAAAGGAACAAACTGTGC  2055
Homo    --------------------------------------------------------------------------------------------------------------  1215

Mus     ------------------------AAAGGGAGAAAAGCTGCATATAGTGGCTCATGTCTTTGATTCTAGCACTCTGAAGAAAGAGGTGGGTGAGTCTTTGTGAGTTAGAG  1292
Rattus  ------------------------AAGGGAAGAAAAGCT----------GCTCATGTCGTTGATTCT---------GAGAAAGAGGTGGGTGGACGTGTGTGTG-----A  1203
Cavia   CTAAACAGGAGGAGAAAGCTACACAGAAGAAAACAATATCCCAGAAGAAACTGAAG--------------------AAACAAAAACTTAGGCACGGAATAAATTAAAC  2145
Homo    ------------------------AAAAAAAAAAAAA---------------------------------------AAAAAAAAAAAAAAAAAAAAAGAGAAAGAGGAAA  1262

Mus     GCTTGCCCAATGTACATACCAAATTTCAGGACAGCCAGGCTACAAAGTGAGACCGTTTCTATAAA---------------------------------------------  1357
Rattus  GGCTGCCCAATGTATATACCAAGTTATGA------------CCATAGTGAGACTATGTCTATAAA---------------------------------------------  1256
Cavia   ATGAAATAAACGCAAATAAAAGTTAAAAAATTAGCTTCATATAAAACAGCAGCAGGGCTGGGGATGTAGCTCAGTGATAGAGCACTTGCCTAGCATGTACAAGGCCCTG  2255
Homo    AAATATTTAACAGAGGGCAGAAATTTTAT---------------------------------------------------------------------------------  1291

Mus     ---------------------TAAACAAATAAATAAAAAGAG----------------------AATAAAAAAGATAAATTAATGAATAAATACAAGAAAAGGAAA--  1422
Rattus  ---------------------TAAACCAATAAATAAAAAGGT----------------------AGA-----------AAACAAATGAATAAATACAAGAAAAGGAAA--  1310
Cavia   AGTTCAATCCTCAGTATCATTTAAAAAAAAAAAAAAGCAGAGTATCCCTTGAGTCAACCTGGCAATACAGCAAAATCCCATCTTAAGAAAAAAAAAAAAAAAAAGGTGGGG  2365
Homo    ---------------------TAAACCAGCAAAAA------------TTCTTATCATACAGCTAATATAGAAGGATAACAATAAAATAAAATATATAGA-----------  1357

Mus     ------------------------------------------------------------------------------------AGAACCTTAAGTGACAGGCACTCTAA  1448
Rattus  ------------------------------------------------------------------------------------AA-ACCAGAAGTGGCATGCACTCTGG  1335
Cavia   TGGGGTACTGGGAATCAAACTCAGGGTCTTGTCCTTGTCTAACAGGCACTGCACTGAGTTACAGACCCCAGCCCTCAAAAAATCAAATTAGGTAAAGACATGTGCCATAG  2475
Homo    ------------------------------------------------------------------------------------ATTTTAGGTGGTCGTAAGTGCTATAG  1383

Mus     AGAAGATGGAGTAGGCAGAGGAGTGGAGGAG--CTCAGTGCAGAATGGAGGGG-------------GGGGGCGGGCAAGGGCGGGACCTG-GTGGGATGAGGTGTGTCTG  1542
Rattus  AGAAGATGAAGTAGGCAGAGGAGTGGGAGAG--CTCGGTGCAGAGCTGAGGAGACAGTGCTACAGTGGGGCATTGCAGGGGTGGGTCATG-GTCAGGGGAGG-GTGCCTG  1441
Cavia   AAAAAGTGAAG------GGAGAGACAGGCAGCTCTCAGTGCAGAATGG--------------AGGCAGGAGGTGATAGGGGAAGGGAAG-GGGAAGGGAG---------  2555
Homo    AAAAAACGAAGCAGGAAAGATAGAGAAGGCATGCTCAGTGCAGAATGGGG---------CTAGGATGTAGGGTTTAAATTTCAGTAGGGTGTCAGGTGAAGGCATCCGC  1484

Mus     CTGAAGCTCCAGA--CGTCAGCCCCGGCTTTATTTT-GCAG  1580
Rattus  CTGAAGCTCCGGA--ACTCAAAGATGGCTTTATTTTTGCAG  1480
Cavia   ---------------CCTCACAGACGTGCTTACTCTTGCAG  2581
Homo    CTGCAGCCCAAGAGGCCTCATAGAATGCTTTGTCGT-GCAG  1524
```

ENSMUSG00000038429 intron 14

Description: Ubiquitin carboxyl-terminal hydrolase 5 (Usp5)
Intron number: 14
Mouse chromosome: 6
Upstream exon length: 89
Downstream exon length: 192
Mouse intron length: 706
Intron alignment length: 1178
Total murinae branch length: 0.18457
K_score: 0.08408
Scaling factor: 0.7844

ENSMUSG00000038429 exon 14 (ORF 1)

```
Mus     GACCACGCGCTTTGCCTCCTTCCCCGACTACCTGGTCATCCAGATCAAGAAGTTCACCTTTGGCTTAGACTGGGTGCCCA 80
Rattus  GACTACACGGTTTGCCTCCTTCCCTGACTACCTGGTCATCCAGATCAAGAAGTTCACCTTTGGCTTAGACTGGGTGCCCA 80
Cavia   GACCACACGGTTTGCCTCATTCCCTGACTACCTGGTCATCCAGATCAAGAAATTCACCTTCGGCTTAGACTGGGTGCCCA 80
Homo    GACCACACGATTTGCCTCATTCCCTGACTACCTGGTCATCCAGATCAAGAAGTTCACCTTCGGCTTAGACTGGGTGCCCA 80

Mus     AGAAGCTGG 89
Rattus  AGAAGCTGG 89
Cavia   AGAAACTGG 89
Homo    AGAAACTGG 89
```

ENSMUSG00000038429 exon 15 (ORF 2)

```
Mus     ATGTGTCCATTGAGATGCCAGAGGAGCTCGATATCTCCCAGCTGAGGGGCACAGGGCTACAGCCGGGAGAGGAGGAGCTG 80
Rattus  ATGTGTCCATTGAGATGCCAGAGGAGCTAGATATTTCCCAGCTGAGGGGCACAGGGCTACAGCCCGGAGAGGAGGAGCTG 80
Cavia   ATGTGTCCATTGAGATGCCAGAAGAGCTGGACATCTCCCAGCTGAGGGGTACAGGGCTGCAGCCAGGAGAGGAGGAGTTG 80
Homo    ATGTGTCCATCGAGATGCCAGAGGAGCTCGACATCTCCCAGTTGAGGGGCACAGGGCTGCAGCCCGGAGAGGAGGAGCTG 80

Mus     CCTGACATTGCCCCACCCCTGGTCACTCCGGATGAGCCCAAAGGTAGCCTTGGTTTCTATGGCAACGAAGACGAAGACTC 160
Rattus  CCTGACATTGCCCCACCCCTGGTCACTCCGGATGAGCCCAAAGGTAGCCTTGGTTTCTATGGCAACGAAGACGAAGACTC 160
Cavia   CCTGACATTGCCCCACCCCTGGTCACTCCGGATGAGCCCAAAG------------------------------------- 123
Homo    CCAGACATTGCCCCACCCCTGGTCACTCCGGATGAGCCCAAAGGTAGCCTTGGTTTCTATGGCAACGAAGACGAAGACTC 160
```

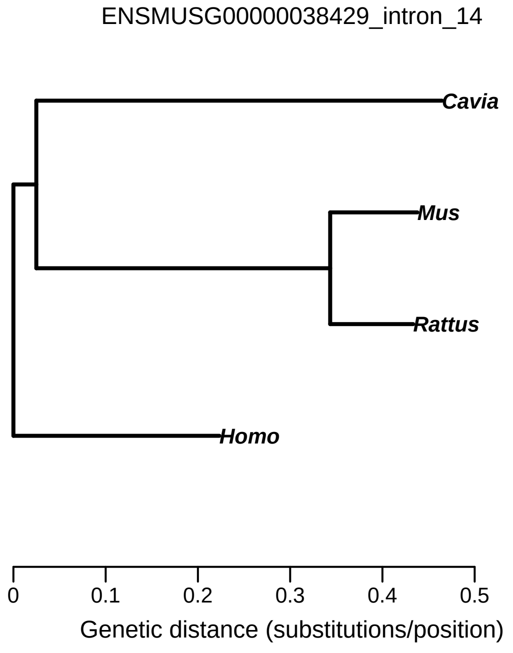

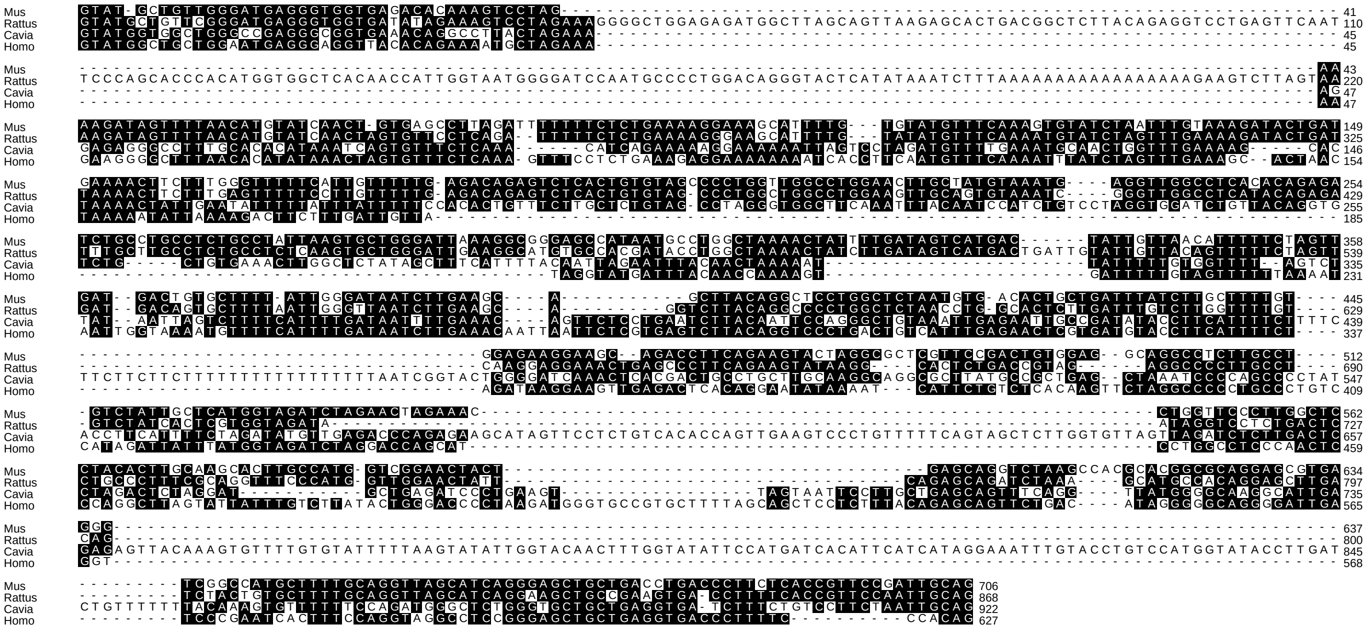
Mus
Rattus
Cavia
Homo

ENSMUSG00000020315 intron 27

Description: Spectrin beta chain, brain 1 (Spnb2)
Intron number: 27
Mouse chromosome: 11
Upstream exon length: 139
Downstream exon length: 85
Mouse intron length: 944
Intron alignment length: 1062
Total murinae branch length: 0.16441
K_score: 0.08489
Scaling factor: 0.78527

ENSMUSG00000020315 exon 27 (ORF 1)

```
Mus     GGATGTGTCATCTGTTGAACTGTTAATGAATAATCATCAAGGTATCAAAGCTGAAATTGATGCTCGTAATGACAGCTTTA 80
Rattus  GGATGTGTCATCTGTTGAACTGTTAATGAATAATCATCAAGGCATCAAAGCTGAAATCGATGCTCGTAATGACAGTTTTA 80
Cavia   GGATGTATCATCTGTAGAATTATTAATGAATAATCATCAAGGTATCAAAGCTGAAATTGATGCTCGTAATGACAGTTTTA 80
Homo    GGATGTATCATCTGTTGAACTCTTAATGAATAATCATCAAGGCATCAAAGCTGAAATTGATGCACGTAATGACAGTTTCA 80

Mus     CAGCCTGCATTGAGCTTGGGAAATCCCTGCTGGCACGGAAACACTATGCTTCTGAGGAG 139
Rattus  CAGCTTGCATTGAACTTGGGAAAGCCCTGCTGGCAAGGAAACACTATGCTTCTGAGGAG 139
Cavia   CAACCTGCATTGAACTTGGGAAGTCCCTGTTGGCAAGAAAACACTACGCGTCTGAAGAG 139
Homo    CAACCTGCATTGAACTTGGGAAATCCCTGTTGGCGAGAAAACACTATGCATCTGAGGAG 139
```

ENSMUSG00000020315 exon 28 (ORF 0)

```
Mus     ATCAAGGAAAAGTTACTGCAGCTGACAGAGAAAAGAAAAGAAATGATTGACAAGTGGGAAGACCGGTGGGAGTGGTTAAG 80
Rattus  ATCAAGGAAAAGTTACTGCAGTTGACGGAGAAAAGAAAAGAAATGATCGACAAGTGGGAAGACCGATGGGAGTGGTTAAG 80
Cavia   ATCAAGGAAAAGTTACTGCAGTTGACTGAAAAGAGAAAAGAAATGATTGACAAGTGGGAAGACCGATGGGAATGGTTAAG 80
Homo    ATCAAGGAAAAATTACTGCAGTTGACGGAAAAGAGGAAAGAAATGATCGACAAGTGGGAAGACCGATGGGAATGGTTAAG 80

Mus     ACTGA 85
Rattus  ACTGA 85
Cavia   ACTGA 85
Homo    ACTGA 85
```

ENSMUSG00000020315_intron_27

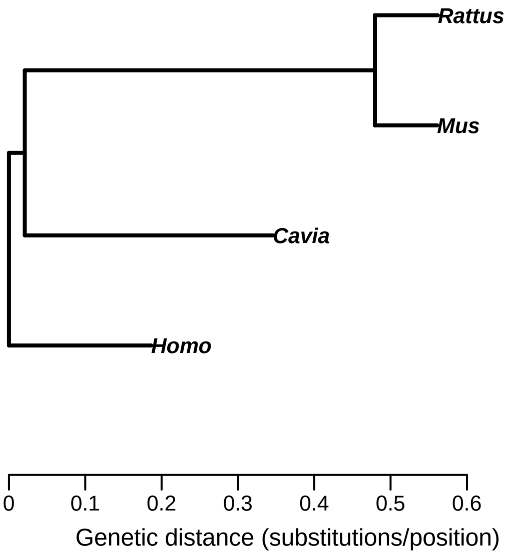

```
Mus     GTAGGATGC-----TGCTTTGGGACCTGGTGTGAACTCAGCCAGTGTTGGCATGTAAAAGGATTTTATTTTGCAACTGTGTCTTTGAA---------------------G 84
Rattus  GTAGGATGCTGCATTGCTTTGGGACCTGACATGAACTCAGCCAGTGTTAGGATGTAAAATGCTTTTATTTTGCA--TGTGTCTTTGAA---------------------G 87
Cavia   GTAGAGTGCTGCTTTACTTTGGGGTCTTGGGACAGATGCCCTGGTATTAAGGTGTAAAGTGTCTGTTCTGTATA--TATTCATGTGCAGACCCAGAAATATGTAAAGTTG 108
Homo    GTAGGTTGCTACTTTGCTTTGGAGCT----------GCAGCTGGCGTCAAGATGTAAAATGACTTTGTTTTATA--TGTTTGTGTGTG---------------------- 76

Mus     CCTCCCACAATCTACAAGGATTGATTCCAGGACTTCTGTGAACACTAGAATCCATAGATGGCTAGATACCATATGTGAAATGACATAGGGCTTATTCATAACTTTAGAAC 194
Rattus  TCTCCCACAATCCATAGGGATTGGTTCCAAGACCTCTCTGAATAGTAGAATCCATAGATGGTTAGATAACATATGTAAAATGTCATAGGATTTATTCAAAACTTTAGAAC 197
Cavia   TCCTTCATTATCCACAGGG-TGTATTTCAGGACCGCCAGAAGTACCAAAATCCGAGAATGCTTAAATCCCACATATAAAATGGCACAGTACTGTGTATGAAT-------- 209
Homo    -------------------------------------------------------------------------------------------------------------- 76

Mus     ATCTCTGGACCCTGTGTAATACCTAACACAGGGTAAATCACTGCTGTACTATAGTAAGGAATGACAAAAGCCAAGTCTACGTGTTCAGTGTAGTTTCAGTTTTTCTAATA 304
Rattus  ATCTCTGGACCTCGTGTCATACCTAACACAGGGGAG---TCTGTTGTACTGTAGTAAGAAATAAC--AAGCCAAGTCCATGTGTTCAGTGTCATTGCAGTTTTTCTGATA 302
Cavia   --------------------------------------------------------------------------AGACACATGCTCAGATGTACTGCATGTCACCTCTAG 245
Homo    ------------------------------------------------------------------------------------TCAGAGATACTGTG------------ 90

Mus     TTTTCATTCTGTGTATTTGTGTGCTTGAGCACAAGGCCTACTTTAACAGGGCCTACAATACACGTGCAAGTGAGTACGCTTCTTTACATCATTTTGAAGCATATTCTGTC 414
Rattus  TTTTTGTTCTGTGTAGTTGTGCACTTGAGTACAA---------------GGCCTACAATACAC-TGCAAGTGAGTGCATTTCTTTACATCATTTTGAAGCTTAATCTGTC 396
Cavia   ATGTGCACTCGCA-ATACACATACTTAGATTTCA-------------------------------------AAACTTGCTTCTTT-------------------CCTGCC 298
Homo    -TTTACATTCTTATACACACACACATATGTTTTA-----------------------------------------AAACTCCTTT-------------------CCTGTG 139

Mus     TGCCTAGAAAAA--CTGTTACATACAACTGTAGACCCAAAACTGGTAATTGTTTGT--CCAGAGATAATTTTTGAGGGGTCCAGGTATAAAAACAGCCTGTGAACTCATC 520
Rattus  TGCTTAGAATAA----CTTACATAAAACCGTAGACCCCAAACCAGCAATTGTTTAG--CCAGAGATCATTTTTGAGGGTTCCAGGTATAAAAACAGCCTGTGAACTCCTC 500
Cavia   TGCCAAGACTAAGACAGTTACACAAGACTCTAGGCTCAGAGCCAGTAACTGCATGGCACAAGAGACAGATTTGAGGGCCCAGA---TTGAGAAGTGGTCTCACTTTCCTC 405
Homo    CGTCTAGAATAATGCAATTATGCAAGACTCTAGGCCCAG-GCTGGTAAATCTCTGGCACAGAAGACAATTTTTGAGGGCTACA-----AAATGGACTCCGCATCCTCCCC 243

Mus     AGTGG------------GATGG----------CTCTGGCTATCACTCAACTCTGCGTTGCATTTGGCTGATGGCTGCA-GCTCTGGTCAGAACGATGGCACTGAT----- 602
Rattus  GGTGG------------GATGG----------CTTTGGCTGTCTCTTATCTC------------------TGGCTGCA-GCTCTGGTCAGGACAGTAGCACTGAT----- 564
Cavia   CTTTAGTACTCAGTC--GCTGACACTCTTCTCCGTTGTCATTTGCTGGGTCCCTTTTTTTCTCAGTGAGATGACTGCATATTCTGGTTAGGACA---------------T 498
Homo    AATCAGTACTCTTTCTAGGTGACGCCAATGTCCATTGACTATGCCCTAGATCAGTTTGCTGTTGAGTGCATGGTTGGTGATTCTGGTTAGGATGGTGGTAATAAAGAGGT 353

Mus     ---------GTCTTTTTTGTAGCATCCTTCACAGGAATGGATGTC-TCTCAGAAAAGCCAAGGCTTTGTCGGTATTACTGTCCTTCTCTTCATAAACGTTAACTGTTTCA 702
Rattus  ---------GTTCCCTTCATAACACCCTTCACAGGAGTGGATGTC-TCTTGGAAAAGCCAAGGCTTTGTCGGTATTGTCGTCCTTCTTTTCATGAACGTT-ACTGTGTCG 663
Cavia   AAATGATAAATTCACTGTGTACTTCCCTTCATGGAAATGGGTGTCTTCTCAGAGG---CAGGGCTTTGCCTCTCTTGCTAGCCCTCT-GTCGTAACTATA-TTTGCTGTA 603
Homo    AAATTATTAGGTGCGTGTGTGGCCCCCTGCATGGGAATGGGCATCCTCTCCAAGAGGTCAGGGCTTTGTCCGTCTTGCTGTCCCTC---TCCTGAACGTG-TTTGCCGCA 459

Mus     GGAAGCCCC----AGAGTCTATCGAAATAGCACACAGTTTGCTACATCAGCACAGGCAGCTTTGAATAGAACTCCCACCCCTTCTGGAAA---ACTAGCCATGTTTTTCT 805
Rattus  GGGAGCTGT----ACAGTCTAGTGACACAGCACACAGTTTGCTGCCTCAGCACAGGCAGCTTGAGCTAGAACTTCCACCCCTTCTGGAAC---ACTAGCTGTGTTCTTCC 766
Cavia   GGGAGCCCT------------------------GGAGTCTAATGCATC-----------------------CTTGGTTTCCTCACAAAAA---------TGCAGCTTTCT 657
Homo    GGGAGCCTCCTCACGACAAAACAGCTTTGGCAGGGCTTCCGCGCCTTC-----------------------CTCCAGCCCCTTCTGGAAGGAGCAGTGCTATGCTTTTCT 546

Mus     CTCCAGGCTCAA------GACATGAAATAATACATTTAAGC-------TGATACTTAGCAGCTTAG-------------GGGAAAATAGATTTTCTTGTACCAAGTGCTC 889
Rattus  CTCCACTCCCAA------GACATGAAATAATACATTTAAGC-------TGATACTTTGCAGCGTAG-------------AGGAAAATACATTTTCTTGTACAAAGTGCTC 850
Cavia   CTCCTCTCCCAG------GACA-----------GACTGGGC-------AGACATTTCCTAGCTTAGTGGTA--CTCTGCAGAAAATGAGCTTTGCTTATATGAAGTGACC 741
Homo    TTCCTCTCCCAGTCCTCAGACA-----------GACTGAGCAGTCCTTAGATATCTCCCAGCTTAATGGTGGTCTTTGCAGAAAATGTATTTTTCTAGTATAAAATGACC 645

Mus     ATCCGGGACAGA------CACAAGGTTGCAGTGTTTTATTAAGG-----------CCCTTGTTTTTGTGCAG 944
Rattus  ATCCAGGACACA------CAAAAGATTGCAGTGTTTTATTAAGG-----------CCCTTGTTTTTGTGCAG 905
Cavia   CGTCAAGGGGGAAGCATGTGGATGTGTGGTGCATTCTACTAAGATCTCCCCTGG-TCCTTTCTTTCATGCAG 812
Homo    CCATCAAGAGGA------------TGTGGTGCATTCCATTAAGATGTCCCGGGGATCCTCTTTTTCATACAG 705
```

ENSMUSG00000043441 intron 1

Description: Probable G-protein coupled receptor 149 (Gpr149)
Intron number: 1
Mouse chromosome: 3
Upstream exon length: 984
Downstream exon length: 193
Mouse intron length: 884
Intron alignment length: 1044
Total murinae branch length: 0.17759
K_score: 0.04979
Scaling factor: 0.78652

ENSMUSG00000043441 exon 1 (ORF 0)

```
Mus     GTTTGCCCCGGGTCAGCCGGCTGCCTCCGGCGCTGGAGCCGGCAGGCGAGAGAATCCCGGGACTCCCCAGGGCACCAACA 80
Rattus  GTTTGCACCGGGTCAGCCTGCTGCCTCTAGCGCTGGAGCCGGCAAGCGAGAGAATCTCTGGACTCCCCGTGGCTCGAGCA 80
Cavia   TTTTGGGCCGGATCTGGCGGCTGCCGCCGGGACCAGGACCGGGACCGGAGCCTGCATGCGTGGACAGCCAGGCACCAGGA 80
Homo    GTTCGGACCGGGTGCGCCCGCTGCCGCTGGGGCTGAAGCCTGCAGGCGTGAGAACCGGGGGACTCTCTATGGCACCAGGA 80

Mus     GCTTCCCCTTGAGCTTGGCACAGAAGCGCTTTTCTTTGATTCTAGCGCTGACGAAAGTCATCCTTTGGCTACCCATGATG 160
Rattus  GCTTCCCCGTGAGCCTGGCACAGAAGCGCTTTGCTTTGATCCTAGCGCTGACTAAAGTCATCCTCTGGCTGCCTATGATG 160
Cavia   GCTTCGCCAGGAGCACAGCGCAGAAGCGCTTCGCTTTGATCCTAGCGTTGACAAAAGTCGTTCTCTGGCTGCCCTTGATG 160
Homo    GCTTCACCGTGAGCGTAGCGCAGAAGCGCTTCGCTTTGATCCTAGCGCTTACAAAAGTCGTCCTTTGGCTGCCCATGATG 160
```

ENSMUSG00000043441 exon 2 (ORF 0)

```
Mus     ATCCACATGGTGGTAAAACACGTGGTGGGGTTTCAGAGCCTTCCGGTCGATATGCTCAGCTTTCTACTAAGCCTGCTGGC 80
Rattus  ATCCACATGGTGGTAAAACACGTGGTGGGGTTTCAGAGCCTTCCGGTCGATATGCTCAGCTTTCTACTAACCCTGCTGGC 80
Cavia   ATCCATATGGTGGTCAAGCACGTGGCGGGAGTTCAGTGCCTTCCCTTCGAGTCCCTCGGCTTTCTGCTCACCCTGCTGGC 80
Homo    ATGCACATGGTGGTCCAGAACGTCGTGGGGTTTCAGAGCCTTCCCTTGGAGACATTCAGCTTTCTACTTACCCTGCTGGC 80

Mus     CAGCAGTGTAACACCAGTGTTTGTCTTATCCAAACGTTGGGCCCACTTGCCTTGTGGCTGCATCATCAACTGCCAGCCAG 160
Rattus  CAGCACTGTAACACCAGTGTTTGTCTTGTCTAAACGATGGGCCCACTTGCCTTGTGGCTGCATCATCAACTGCCAGCCTG 160
Cavia   TGCCACTGTGACCCCAGTGTTTGTCTTGTCCAAACGCTGGACCCACTTGCCCTGTGGCTGCATCATCAACTGCAGGCAGG 160
Homo    CACCACTGTAACCCCAGTGTTTGTCTTGTCCAAACGCTGGACCCACTTGCCCTGTGGCTGCATCATCAACTGCAGGCAGA 160
```

ENSMUSG00000043441_intron_1

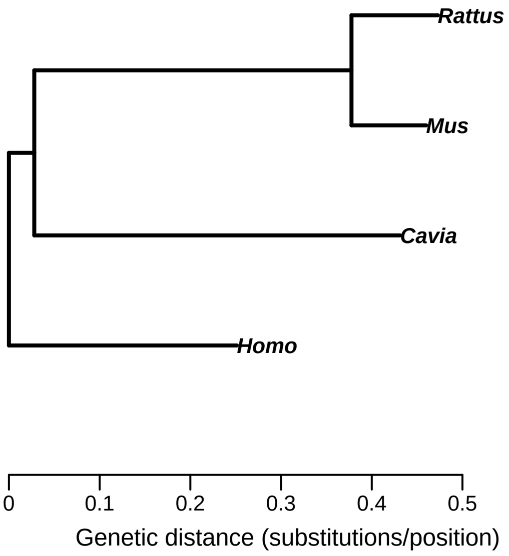

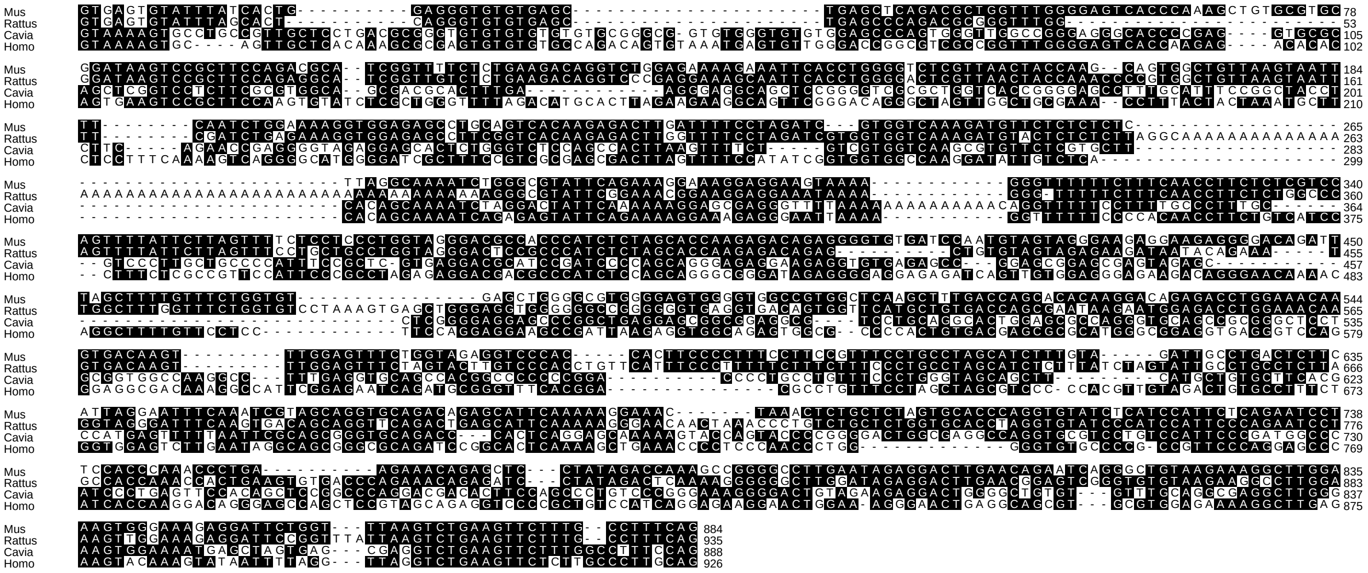

ENSMUSG00000023084 intron 9

Description: Leucine-rich repeat-containing protein C10orf92 homolog (4933430H15Rik)
Intron number: 9
Mouse chromosome: 3
Upstream exon length: 90
Downstream exon length: 150
Mouse intron length: 760
Intron alignment length: 441
Total murinae branch length: 0.14154
K_score: 0.05815
Scaling factor: 0.78701

ENSMUSG00000023084 exon 9 (ORF 0)

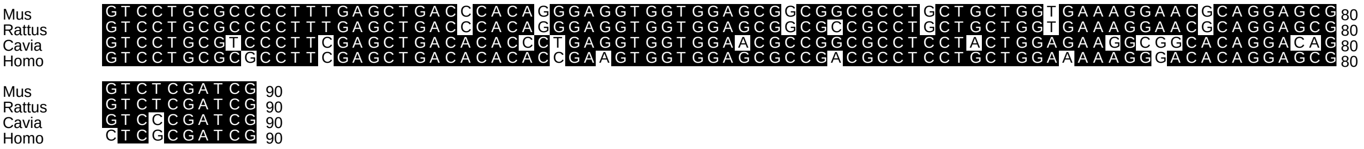

ENSMUSG00000023084 exon 10 (ORF 0)

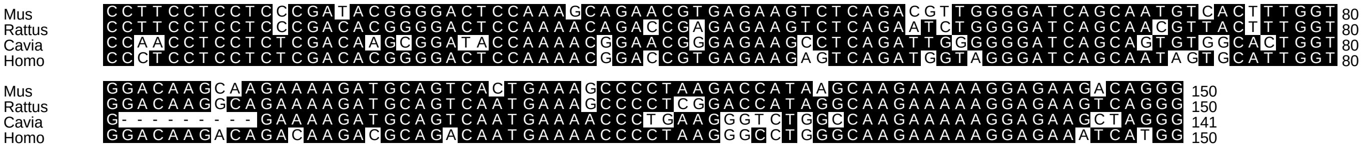

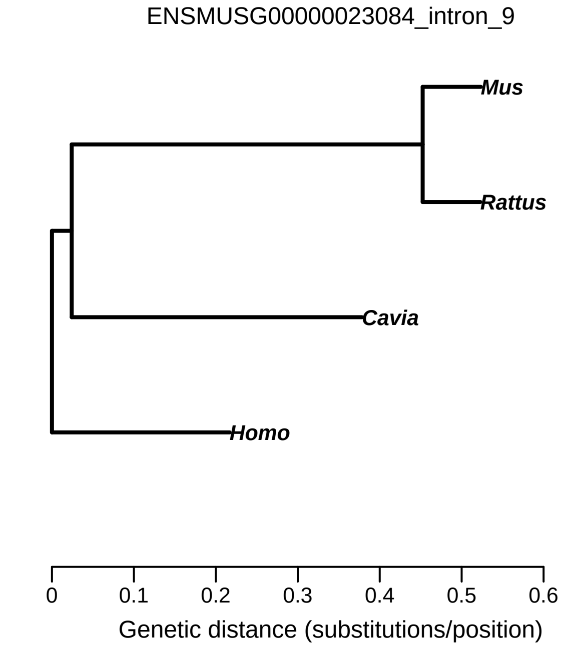

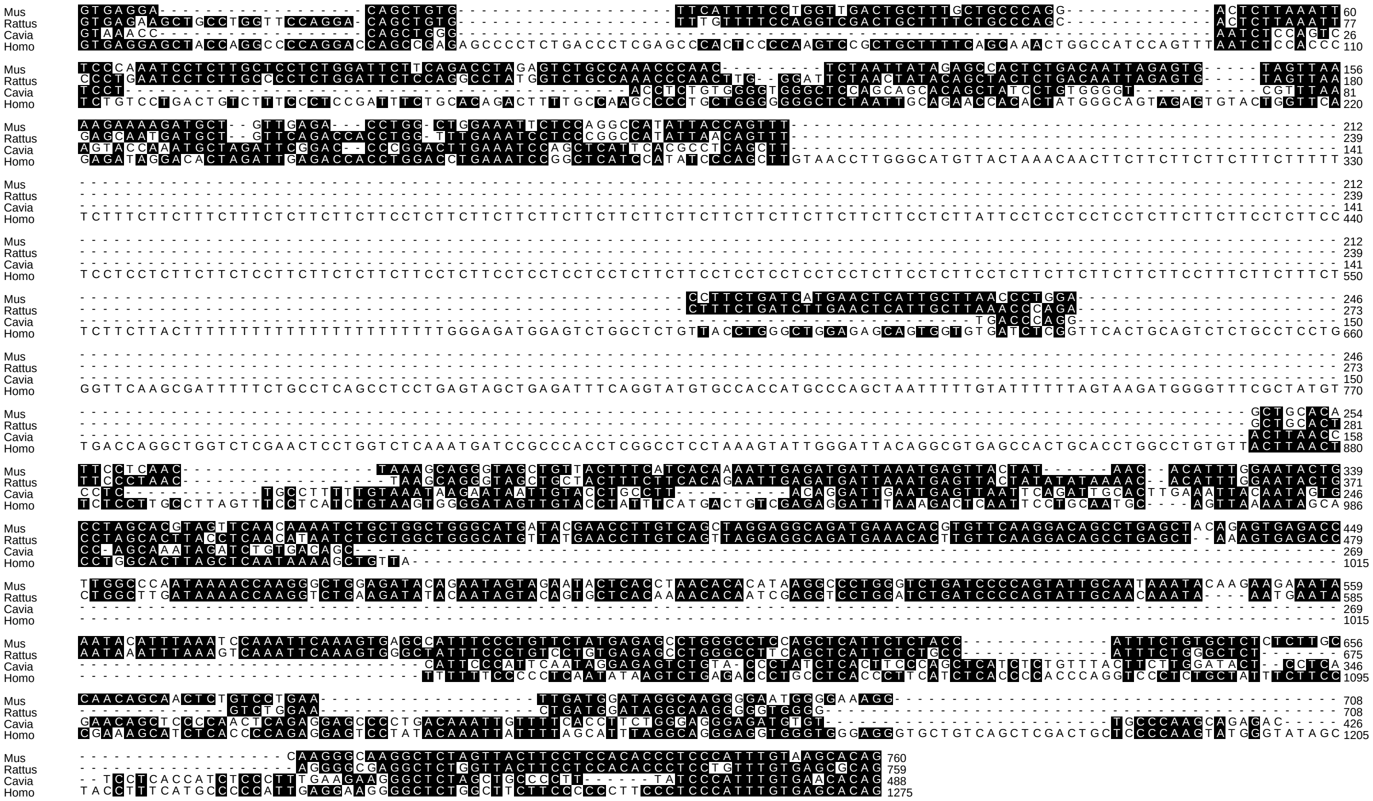
Mus
Rattus
Cavia
Homo

ENSMUSG00000074923 intron 1

Description: Serine/threonine-protein kinase PAK 6 (Pak6)
Intron number: 1
Mouse chromosome: 2
Upstream exon length: 204
Downstream exon length: 657
Mouse intron length: 728
Intron alignment length: 1188
Total murinae branch length: 0.19774
K_score: 0.06703
Scaling factor: 0.78703

ENSMUSG00000074923 exon 1 (ORF 0)

```
Mus     GCAGAACTTCCAGCATCGTGTCCACACCTCCTTTGACCCCAAGGAAGGCAAGTTTGTGGGTCTGCCCCCACAATGGCAGA 80
Rattus  ACAGAACTTCCAGCACCGTGTTCACACCTCATTTGACCCCAAGGAAGGCAAGTTCGTGGGCCTGCCCCCACAATGGCAGA 80
Cavia   ACAGAACTTCCAACACCGTGTCCACACTTCCTTTGACCCTAAAGAGGGCAAATTTGTGGGCCTCCCGCCACAATGGCAAA 80
Homo    ACAGAACTTCCAGCACCGTGTCCACACCTCCTTCGACCCCAAAGAAGGCAAGTTTGTGGGCCTCCCCCCACAATGGCAGA 80

Mus     ACATTCTGGATACTCTGAGGCGGCCCAAGCCTGTGGTGGATCCTTCTCGAATCACACGGGTGCAGTTGCAGCCCATGAAG 160
Rattus  ACATCCTGGATACTCTGAGGCGGCCCAAGCCTGTGGTGGATCCTTCTCGAATCACACGGGTGCAGTTGCAGCCCATGAAG 160
Cavia   ACATCCTGGACACACTACGACGGCCCAAGCCTGTGGTGGACCCTTCGCGTATCACGCGGGTACAGCTTCAGCCCATGAAG 160
Homo    ACATCCTGGACACACTGCGGCGCCCCAAGCCCGTGGTGGACCCTTCGCGAATCACACGGGTGCAGCTCCAGCCCATGAAG 160
```

ENSMUSG00000074923 exon 2 (ORF 0)

```
Mus     ACAGTGGTACGGGGCAGCTCAGTGCCCACGGAGGGCTACATCTCCGGGCTGCTCAACGACATTCAGAAGTTGTCAGTTAT 80
Rattus  ACGGTAGTACGGGGCAGCTCAGTGCCCACAGAGGGCTACATCTCCGGGCTGCTCAATGACATTCAGAAGTTGTCAGCTAT 80
Cavia   ACAGTGGTGCGGGGCAGTTCTGTGCCCACAGATGGCTATATCTCAGGACTGCTCAATGACATCCAGAAGTTGTCGGTCAT 80
Homo    ACAGTGGTGCGGGGCAGCGCGATGCCTGTGGATGGCTACATCTCGGGGCTGCTCAACGACATCCAGAAGTTGTCAGTCAT 80

Mus     CAGCTCCAACACCCTGCGTGGACGCAGCCCCACCAGCCGGCGGCGGGCACAGTCCCTGGGGCTGCTGGGAGATGATCAGT 160
Rattus  CAGCTCCAACACTCTGCGTGGACGCAGCCCCACCAGCCGGCGGCGGGCACAGTCCCTGGGGCTGCTAGGAGATGATCAGT 160
Cavia   CAGCTCCAACACACTCCGCGGCCGCAGCCCCACCAGTCGGCGGCGGGCACAGTCCCTGGGGCTGCTAGGGGACGAGCTCT 160
Homo    CAGCTCCAACACCCTGCGTGGCCGCAGCCCCACCAGCCGGCGGCGGGCACAGTCCCTGGGGCTGCTGGGGGATGAGCACT 160
```

ENSMUSG00000074923_intron_1

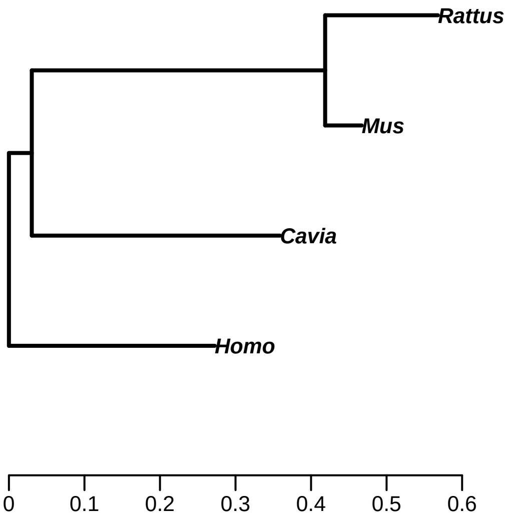

Genetic distance (substitutions/position)

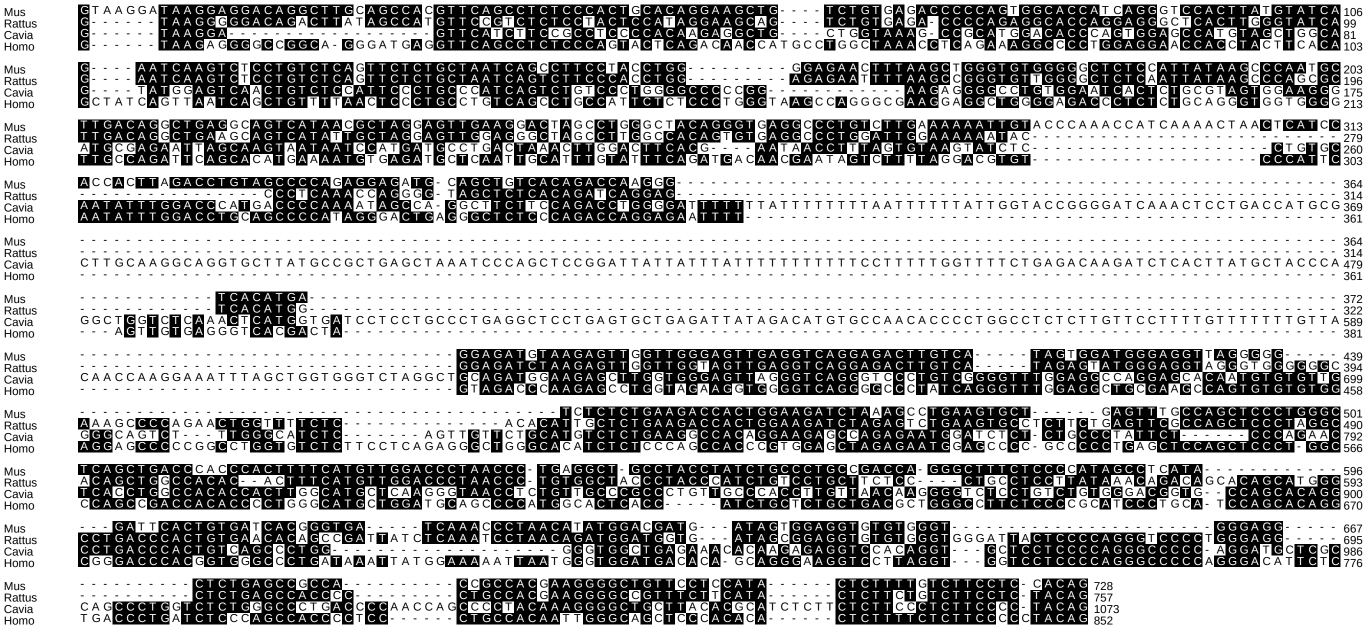
Mus
Rattus
Cavia
Homo

ENSMUSG00000040354 intron 18

Description: Methionyl-tRNA synthetase, cytoplasmic (Mars)
Intron number: 18
Mouse chromosome: 10
Upstream exon length: 187
Downstream exon length: 72
Mouse intron length: 521
Intron alignment length: 762
Total murinae branch length: 0.19074
K_score: 0.07352
Scaling factor: 0.78776

ENSMUSG00000040354 exon 18 (ORF 1)

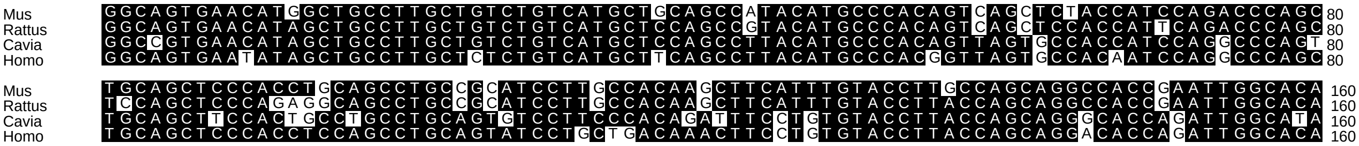

ENSMUSG00000040354 exon 19 (ORF 0)

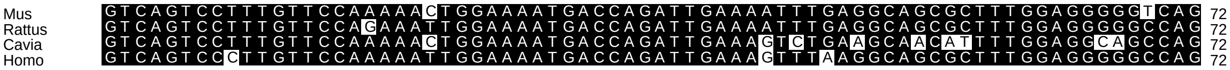

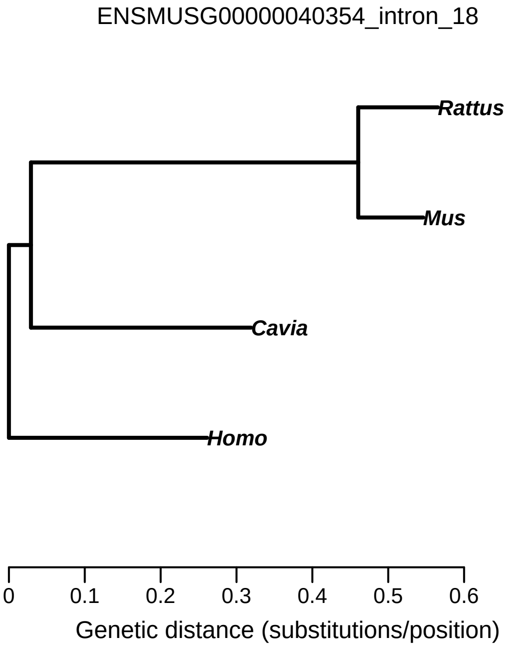

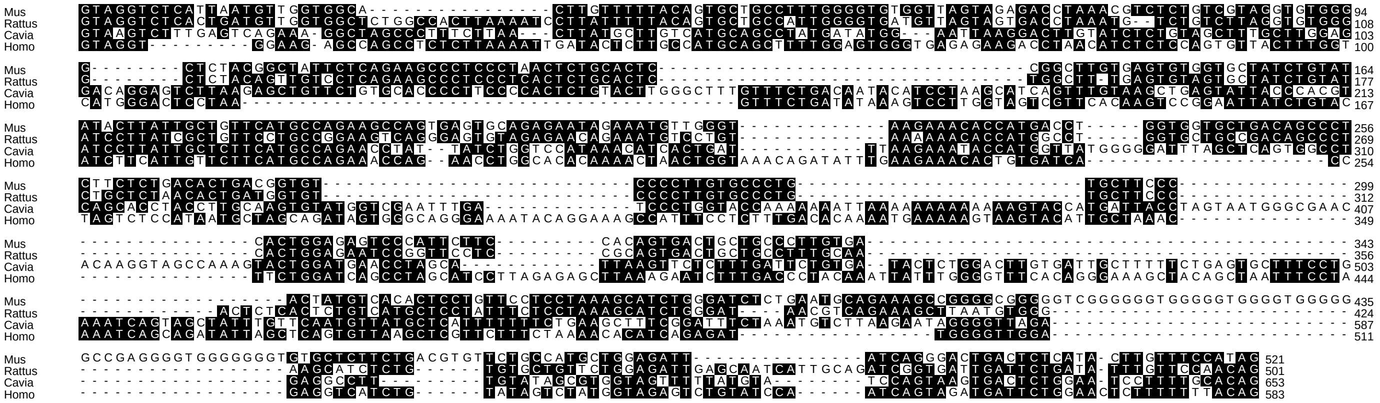
Mus
Rattus
Cavia
Homo

ENSMUSG00000037965 intron 21

Description: zinc finger CCCH-type containing 7A (Zc3h7a)
Intron number: 21
Mouse chromosome: 16
Upstream exon length: 164
Downstream exon length: 190
Mouse intron length: 1535
Intron alignment length: 2076
Total murinae branch length: 0.19618
K_score: 0.07174
Scaling factor: 0.78861

ENSMUSG00000037965 exon 21 (ORF 0)

```
Mus     ACTTCCACTGCTGGATGTGTGGGAAGAACTGTAACAGTGAGAAGCAATGGCAAGACCACATCTCCTCTGAGAAGCACAAG 80
Rattus  ACTTCCACTGCTGGATGTGTGGGAAGAACTGTAACAGTGAGAAGCAGTGGCAAGACCACATCTCCTCCGAGAAGCACAAG 80
Cavia   ATTTTCACTGCTGGATGTGTGGGAAGAACTGTAATAGTGAGAAGCAGTGGCAGGGCCACATTTCTTCTGAGAAGCACAAG 80
Homo    ACTTTCACTGCTGGATGTGTGGGAAAAACTGCAACAGTGAGAAGCAGTGGCAGGGCCACATCTCCTCCGAGAAGCACAAA 80

Mus     GAGAAGGTTTTCCACACTGAGGATGACCAGTACTGCTGGCAGCACCGCTTCCCCACTGGGTACTTCAGTATTTGTGAGAG 160
Rattus  GAGAAGGTTTTCCACACTGAGGACGACCAGTACTGCTGGCAGCACCGCTTCCCCACAGGGTACTTCAGTGTTTGTGACAG 160
Cavia   GAGAAGTTTTTTCATACAGAAGATGACCAGTATTGCTGGCAACACCGCTTCCCAACAGGATATTTTAGTATTTGTGAGAG 160
Homo    GAGAAGGTTTTCCACACCGAGGACGACCAGTACTGCTGGCAGCACCGCTTCCCAACAGGCTATTTCAGTATTTGTGATAG 160
```

ENSMUSG00000037965 exon 22 (ORF 1)

```
Mus     GTATATAAATGGCTCCTGCACAGAAGGAAGCAGCTGTAAATTTGCACATGGAAATGCTGAACTTCATGAATGGGAAGAAA 80
Rattus  GTATATGGATGGCTCCTGCACAGAAGGAAGCAGCTGTAAATTTGCACATGGAAATGCTGAACTTCATGAATGGGAAGAGA 80
Cavia   GTATATGAATGCAATTTGCACTGAAGGAAACAGCTGTAAATTTGCACATGGAAATGCCGAGCTCCAAGAATGGGAAGAAA 80
Homo    GTATATGAATGGCACCTGCCCAGAAGGAAACAGCTGTAAATTTGCACATGGAAATGCCGAACTTCATGAATGGGAAGAAA 80

Mus     GAAGAAAGGCCCTACAGATGAAATTCAAGAAAGCACGGAAAGATCACTTAATTGCCCCAAATGATAATGACTTTGGAAAA 160
Rattus  GAAGGAAGGCCCTTCAGATGAAACTCCAGAAAGCACGGAAAGATCATTTAATTGCCCCAAATGATAATGACTTTGGAAAA 160
Cavia   GAAGAAATGCCCTAAAAATGAAGCTCAGCAAAGCACGGAAAGATCACTTAATTGCCCCAAATGATAATGACTTTGGAAAA 160
Homo    GAAGAGATGCCCTAAAGATGAAGCTCAACAAAGCACGAAAAGATCACTTAATTGGCCCAAATGATAATGACTTTGGAAAA 160
```

ENSMUSG00000037965_intron_21

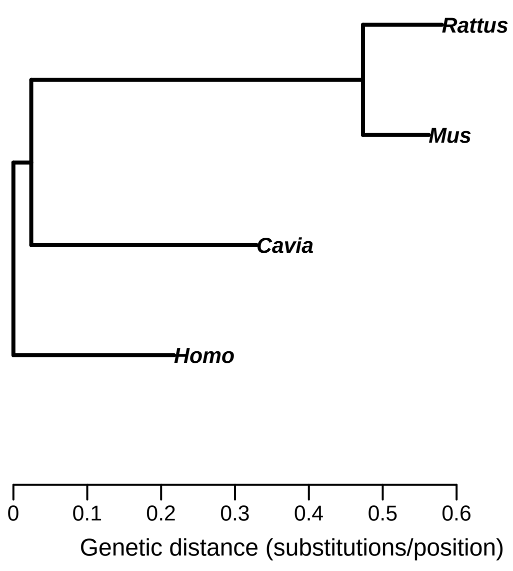

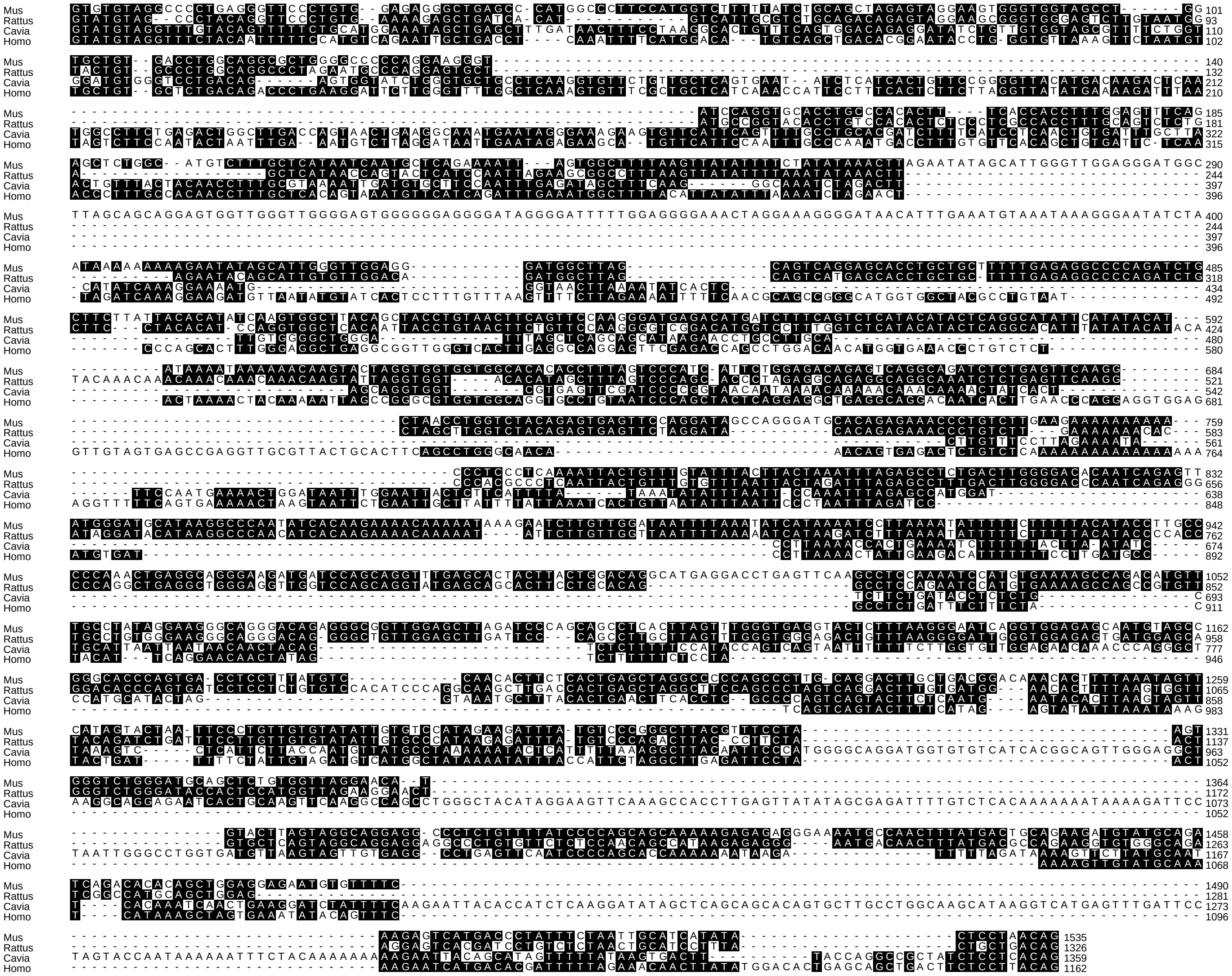
Mus
Rattus
Cavia
Homo

ENSMUSG00000007122 intron 4

Description: Calsequestrin-1 Precursor (Casq1)
Intron number: 4
Mouse chromosome: 1
Upstream exon length: 112
Downstream exon length: 74
Mouse intron length: 358
Intron alignment length: 409
Total murinae branch length: 0.13041
K_score: 0.05402
Scaling factor: 0.789

ENSMUSG00000007122 exon 4 (ORF 0)

```
Mus     GTCCTAGAAGACCCTGTAGAGTTGATTGAAGGTGAACGAGAGCTGCAGGCATTTGAGAATATTGAAGATGAAATCAAACT 80
Rattus  GTCCTAGAAGACCCTGTCGAATTGATTGAAGGTGAACGAGAGCTGCAGGCATTTGAGAACATCGAGGATGAAATCAAACT 80
Cavia   GTCCTGGAAGACCCTGTGGAATTAATTGAGGGTGAACGAGAGCTTCAGGCATTTGAAAATATTGAGGATGAGATCAAACT 80
Homo    GTCCTAGAGGACCCTGTGGAATTGATTGAAGGTGAACGAGAGCTGCAGGCGTTTGAGAATATTGAGGATGAGATCAAACT 80

Mus     CATTGGCTACTTCAAGAGCAAAGACTCAGAGC 112
Rattus  CATTGGCTACTTCAAGAGCAAAGACTCAGAGC 112
Cavia   CATTGGCTACTTCAAGAACAAAGACTCAGAGC 112
Homo    CATTGGCTACTTCAAGAGCAAAGACTCAGAGC 112
```

ENSMUSG00000007122 exon 5 (ORF 2)

```
Mus     ATTACAAAGCCTACGAGGACGCAGCTGAAGAGTTCCATCCCTACATCCCTTTCTTCGCTACCTTCGACAGCAAG 74
Rattus  ATTACAAAGCCTACGAGGATGCAGCTGAAGAATTCCACCCCTACATCCCTTTCTTCGCTACCTTCGACAGCAAG 74
Cavia   ATTACAAAGCCTATGAAGATGCAGCCGAAGAGTTTCATCCCTACATCCCCTTCTTCGCCACCTTTGACAGCAAG 74
Homo    ATTACAAAGCCTTCGAGGATGCAGCTGAGGAGTTTCATCCCTACATCCCCTTCTTCGCCACCTTCGACAGCAAG 74
```

ENSMUSG00000007122_intron_4

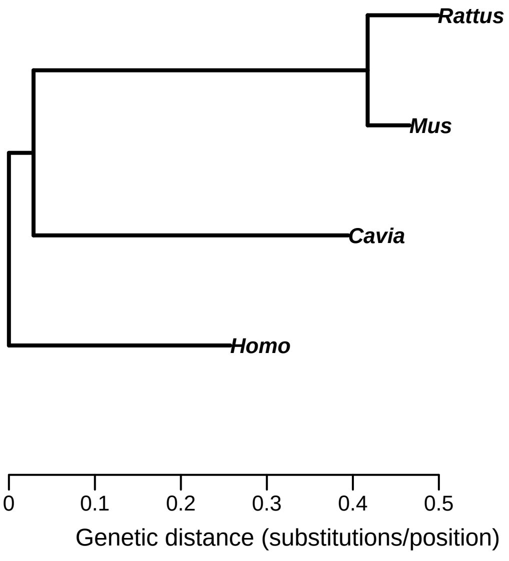

```
Mus     GTGGGTAAC----------------AGCTCTTTCTGAC--------CCACCTATCCTCCTTGCCTCCCTACTTTGGCCAGTGGCCAAGCATCTCTGGTTCTGACCTCCTA 86
Rattus  GTGGGTAAC----------------AGCTCTTTCTGACCCTGCACTCCACCTATCCTCCTTGCCTCCCTCCTTTGGCCAGAGGCTAAGCATGTCTGGTTCTGACCTTCCA 94
Cavia   GTGGGTAACCCTGAGACTTCACTTTCCTCCTCCCCTGC---------TGCCCACCTCCTTTTGTCTTCCTGTCTGTCCACCAGCTCCACATCTTTGTCTC---------- 91
Homo    GTGGGTAACCCTCAGACTCCACTGTGCCCCTCTCTGGA---------TCCCCATCTCACCTGTCCTCCCACCTCCCCACATCACCCAGTGTCTCAGCCTCTACCTCTGCA 101

Mus     GGCCCCCTTCTTCCTTCT--GCACGGTCAGCTCCATCATCTGTC--------CCTGCACTCTGGGGCATTCC---CAAACA-CCCCCAGCATTCCGCTGCTTCCTCTACA 182
Rattus  GGCCCTCTCCTTCCTTCT--GCACCGTCAGCTCCACCATCTGTTCT-ATCTGCCTGCACTCTGGGGCATTCC---CAAACA-CCCCCAGCATTCCACTGCTTCCTCTACA 197
Cavia   ---CTTATTTCTCCTGCT------TGCTAGCTCCATCTTCTAATCT------------CGCTGAGAGA-------CAAACACCCCCCAGGATTCCACCTGTGCTATTATA 173
Homo    CTTCCCACCTCTTCCTCCAGCCGTGGTCAGCACCATCAACAGCTCTGACCTCCCTGTACTCTGGGGGTTCCCAGACAAGCA-CCTCCAGTATTCCACCT----------- 199

Mus     ATTCTATT----------GTATTTTATTCTCAGAGAGCTAGAGTTAGAGAGCTAAAGGGGATGGGTCTCTTCCTGGGACCCACATGTCAAGGAAGACCTGCAGAGAAGAT 282
Rattus  ATTCGATT----------GTATTTTATTCTCAGGGAATTAGAGCT-------TCGAGGGGATGGGTCTCGGGCTGAGACTCACATGTTGGGGAGGATCTGTAGAGAA--- 287
Cavia   TTATAGTTCTAGAGTATCTTACCTCACTCTCAGCCGGTTAG---------------AGGAGTAGCTCTCAGAATGAACCCCACCCACCAACAG----CTGGGGATAGAC- 263
Homo    ------------------TCCCCTCACTCTCGAAGAACTAG--------------AAGGGGTGGGTCTCAAAATGAACCCTGCCTGCAAAGAA----TTGGGGACGA--- 270

Mus     TCCCCTTGGTGGTGGGGGGAGTGATTCTTTGGCAGACCCTGAACATGACTGTGACTGTCCCT---TCCTCCCCCTCAG 358
Rattus  -----------GCCAAGGGGAATGACTCTCTGGCAGACCCTGAAC------GTGACTGTCCCT---TCCTCCCCTTCAG 346
Cavia   -----------CTGAGCAGAACTGATTCCTAGGCAGACCCTCATTCT-----AGATTGACTCTGC-TTTTTTCCCTCAG 325
Homo    ------------TAGTGGGGGATGATTCCCGGGCAGACCCTGGTTTCCCCAGAGACTGACTCTGCATTCCACCCCCCAG 337
```

ENSMUSG00000025218 intron 5

Description: DNA polymerase lambda (Poll)
Intron number: 5
Mouse chromosome: 19
Upstream exon length: 174
Downstream exon length: 129
Mouse intron length: 702
Intron alignment length: 922
Total murinae branch length: 0.22332
K_score: 0.08374
Scaling factor: 0.78936

ENSMUSG00000025218 exon 5 (ORF 0)

```
Mus     CCCGGGAATTGGCAAGCGGATGGCGGAGAAGGTCATGGAGATCCTGGAGAGTGGGCATCTGCGGAAGCTAGACCACATCA 80
Rattus  CCCAGGAGTTGGCAGGCGAATGGCAGAGAAGGTCATGGAGATCCTGGAGAGTGGGCATCTGCGGAAGCTGGACCACATCA 80
Cavia   CCCTGGGGTTGGGAAGCGGATGGCTGAGAAGATACAGGAGATCCTGGAGAGCGGGCATTTGCGGAAGCTGGACCACATCA 80
Homo    CCCTGGGATTGGGAAGCGGATGGCTGAGAAAATCATAGAGATCCTGGAGAGCGGGCATTTGCGGAAGCTGGACCATATCA 80

Mus     GTGACAGTGTGCCTGTCTTGGAGCTCTTCTCCAACATCTGGGGAGCTGGGACGAAGACTGCCCAGATGTGGTACCATCAG 160
Rattus  GCGACAGCGTGCCTGTCTTAGAGCTCTTCTCCAACATCTGGGGAGCCGGGACGAAGACTGCCCAGATGTGGTACCATCAG 160
Cavia   GCGAGAGTGTGCCTGTCCTGCAGCTCTTCTCCAGCATCTGGGGAGCCGGCACCAAGACTGCCCAGATGTGGTACCATCAG 160
Homo    GTGAGAGCGTGCCTGTCTTGGAGCTCTTCTCCAACATCTGGGGAGCTGGGACCAAGACTGCCCAGATGTGGTACCAACAG 160
```

ENSMUSG00000025218 exon 6 (ORF 0)

```
Mus     GGCTTCCGAAACCTAGAAGATCTCCAAAGCCTGGGCTCCCTGACCGCTCAGCAGGCCATTGGCTTGAAGCACTACGATGA 80
Rattus  GGCTTCCGAAGCCTAGAAGATATCCGAGGCCTGGCCTCCCTGACCGCTCAGCAGGCCATTGGCTTGAAGCACTATGATGA 80
Cavia   GGCTTCCGGAGCCTGGAAGACATTCGCAACCTGGCCTCCCTGACTGCCCAGCAAGCCATTGGCCTGAAGCATTACAGTGA 80
Homo    GGCTTCCGAAGTCTGGAAGACATCCGCAGCCAGGCCTCCCTGACAACCCAGCAGGCCATCGGCCTGAAGCATTACAGTGA 80

Mus     CTTCCTGGACCGCATGCCCAGGGAGGAGGCTGCGGAAATTGAGCAGACG 129
Rattus  CTTCCTGGACCGCATGCCCAGGGAGGAGGCTGCAGAAATTGAGCAGATG 129
Cavia   CTTCCTGGAGCGCATGCCCAGAGACGAAGCTGCAGAGATTGAGCAGACA 129
Homo    CTTCCTGGAACGTATGCCCAGGGAGGAGGCTACAGAGATTGAGCAGACA 129
```

ENSMUSG00000025218_intron_5

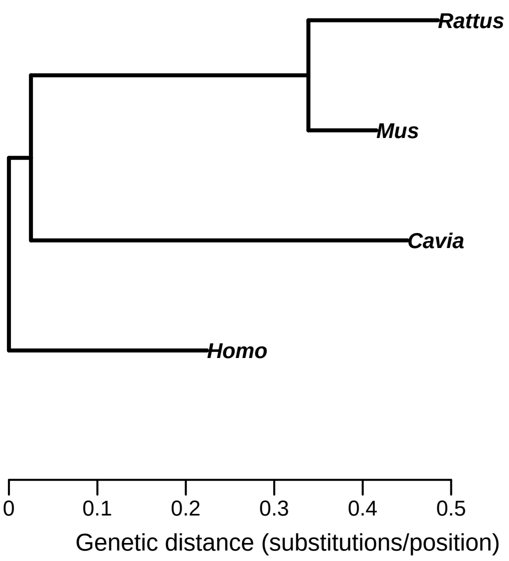

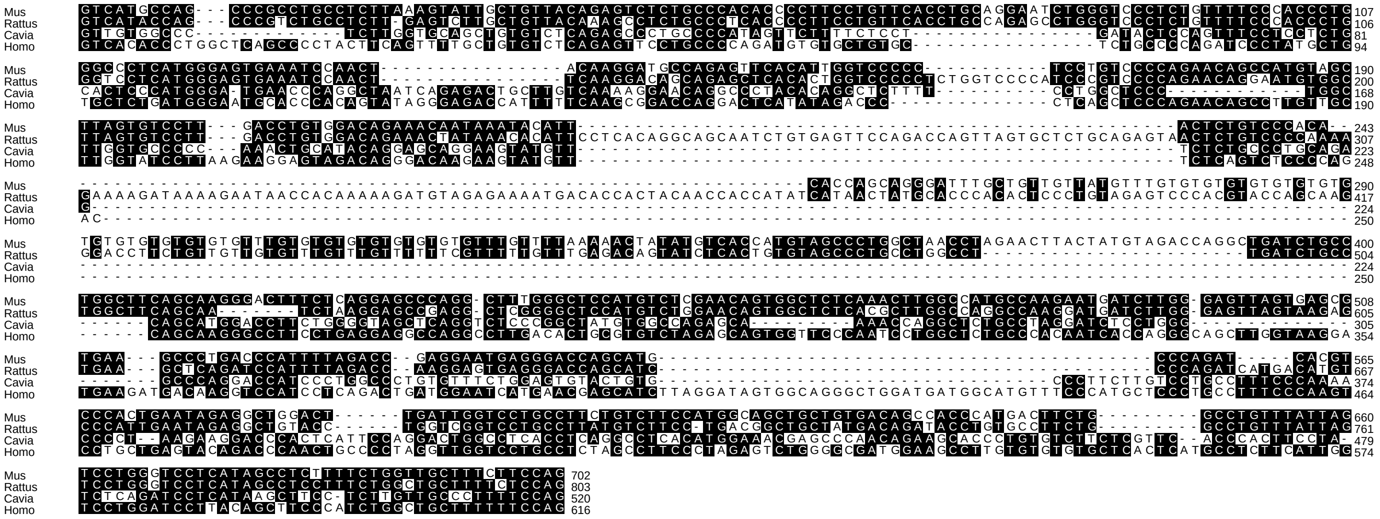
Mus
Rattus
Cavia
Homo

ENSMUSG00000003555 intron 3

Description: Steroid 17-alpha-hydroxylase/17,20 lyase (Cyp17a1)
Intron number: 3
Mouse chromosome: 19
Upstream exon length: 230
Downstream exon length: 87
Mouse intron length: 729
Intron alignment length: 1090
Total murinae branch length: 0.22435
K_score: 0.08621
Scaling factor: 0.78981

ENSMUSG00000003555 exon 3 (ORF 2)

```
Mus     GCTCATCTTCAAGTCAGTAATCAATATCATCTGTACCATCTGCTTCAACATCTCTTTTGAGAACAAGGATCCGATACTGA 80
Rattus  GCCTATCTTCATGTCAGTAACCAACATCATCTGTGCTATCTGCTTCAACATCTCTTATGAGAAAAAGGATCCAAAACTGA 80
Cavia   GTCAATCTTCATGACGGTAGTCAACATCATCTGCATGATCTGCTTCAGTGTCTCATACAAGGAGGGGGACATGGAGTTGG 80
Homo    TCCTGTCTTCGTGGCGGTAACCAATGTCATCTCCTTGATCTGCTTCAATACCTCCTACAAGAATGGGGACCCTGAGTTGA 80

Mus     CTACCATACAGACCTTTACAGAGGGTATTGTGGATGTCCTGGGCCACAGCGATCTGGTGGACATATTCCCGTGGTTGAAG 160
Rattus  CCGCCATAAAGACCTTTACTGAGGGTATCGTGGATGCCACGGGCGACAGAAATCTGGTGGACATATTCCCATGGTTGACG 160
Cavia   TGACCATAAGGCGCTTCACAACAGGCTTCGTGAATAGCCTGAGTGATGACAATCTCGTGGACATATTCCCCTGGCTGAAG 160
Homo    ATGTCATACAGAATTACAATGAAGGCATCATAGACAACCTGAGCAAAGACAGCCTGGTGGACCTAGTCCCCTGGTTGAAG 160
```

ENSMUSG00000003555 exon 4 (ORF 0)

```
Mus     ATTTTTCCCAATAAAAACTTGGAAATGATAAAGGAACACACTAAAATTCGAGAAAAAACACTGGTTGAAATGTTTGAAAA 80
Rattus  ATTTTTCCCAACAAAGGCTTGGAGGTGATAAAGGGTTATGCCAAAGTTCGAAATGAAGTACTGACTGGAATATTTGAAAA 80
Cavia   ATCTTCCCCAATAAAACCCTGGAAATGATAAGGAAGTATACTGAAATCCGAGGAGCCATGCTGAGTAAGATCCTGAAAGA 80
Homo    ATTTTCCCCAACAAAACCCTGGAAAAATTAAAGAGCCATGTTAAAATACGAAATGATCTGCTGAATAAAATACTTGAAAA 80

Mus     ATGCAAG 87
Rattus  GTGCAGG 87
Cavia   GTGCAAG 87
Homo    TTACAAG 87
```

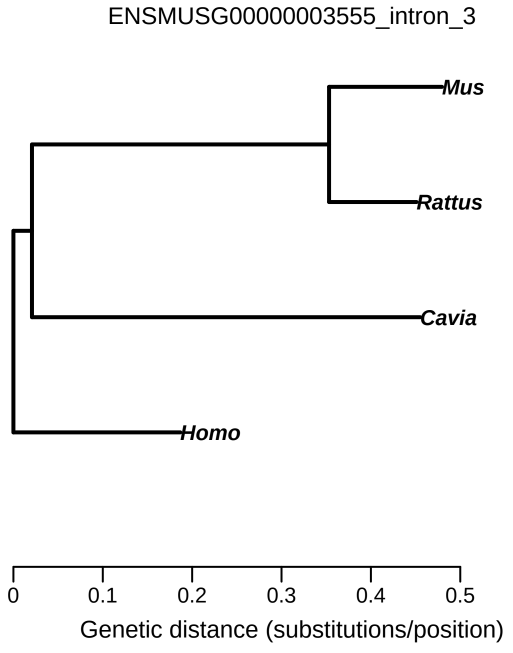

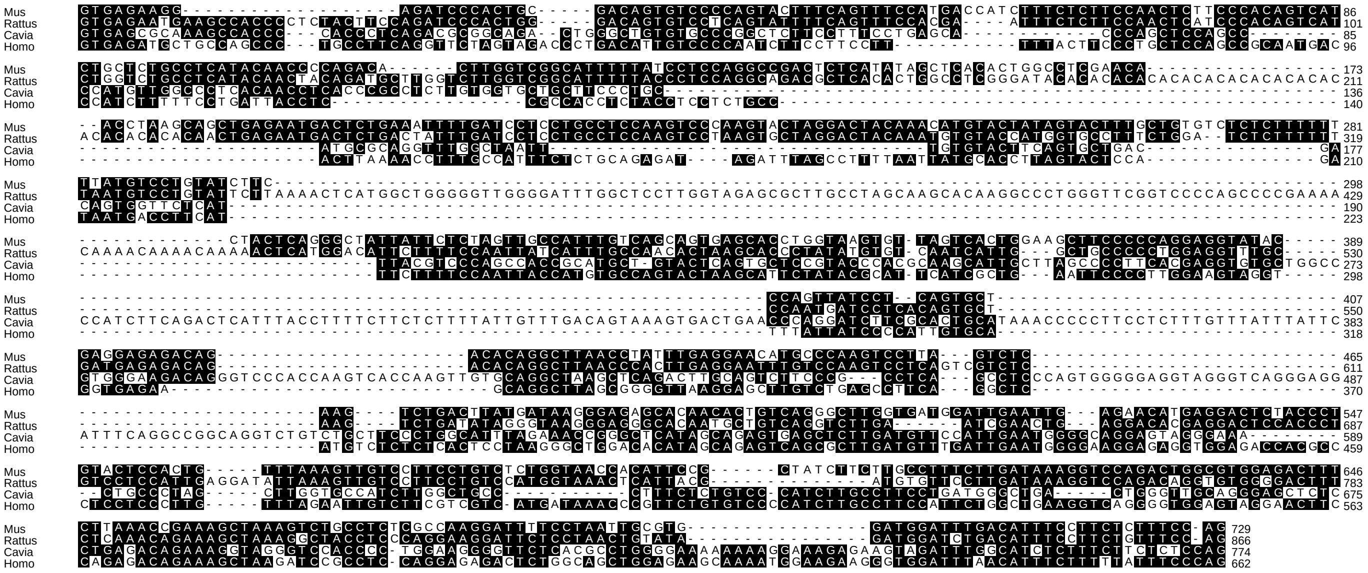
Mus
Rattus
Cavia
Homo

ENSMUSG00000022763 intron 17

Description: Apoptosis-inducing factor 3 (Aifm3)
Intron number: 17
Mouse chromosome: 16
Upstream exon length: 76
Downstream exon length: 105
Mouse intron length: 470
Intron alignment length: 528
Total murinae branch length: 0.09508
K_score: 0.07853
Scaling factor: 0.79009

ENSMUSG00000022763 exon 17 (ORF 2)

```
Mus     GCTACGGAGAAGGCTTCGATGATGTCATCATTCAGGGGGATCTGGAGGAGCTGAAGTTTGTGGCTTTTTATACCAA 76
Rattus  GCTACGGAGAAGGCTTCGATGATGTCATCATTCAGGGGGATCTGGAGGAGCTGAAGTTTGTGGCTTTTTATACCAA 76
Cavia   GCTATGGAGAAGGCTTTGACGATGTCATCATCCAAGGCGACCTGGAGGAGCTGAAATTTGTGGCTTTTTATACCAA 76
Homo    GCTACGGAGAAGGCTTCGACGACGTCATCATCCAGGGGGATCTGGAGGAGCTGAAGTTTGTGGCTTTTTACACTAA 76
```

ENSMUSG00000022763 exon 18 (ORF 1)

```
Mus     AAGTGACGAAGTGATTGCTGTGGCCAGCATGAACTACGATCCCATCGTATCCAAGGTGGCTGAGGTGCTAGCCTCAGGTC 80
Rattus  AGGTGACGAAGTGATTGCTGTGGCCAGCATGAACTACGACCCCATCGTATCCAAGGTGGCCGAGGTGCTGGCCTCAGGTC 80
Cavia   AGGCGACGAGGTGATTGCTGTGGCTAGCATGAACTACGATCCCATCGTATCCAAGGTGGCTGAGGTGCTGGCCTCTGGCC 80
Homo    AGGCGACGAGGTGATCGCCGTGGCCAGCATGAACTACGATCCCATTGTGTCCAAGGTCGCTGAGGTGCTGGCCTCAGGCC 80

Mus     GAGCCATCCGGAAGCGGGAGGTGGA 105
Rattus  GAGCCATCCGGAAGCGGGAGGTGGA 105
Cavia   GTGCCATCCGGAAGCGGGAGGTGGA 105
Homo    GTGCCATCCGGAAGCGGGAGGTGGA 105
```

ENSMUSG00000022763_intron_17

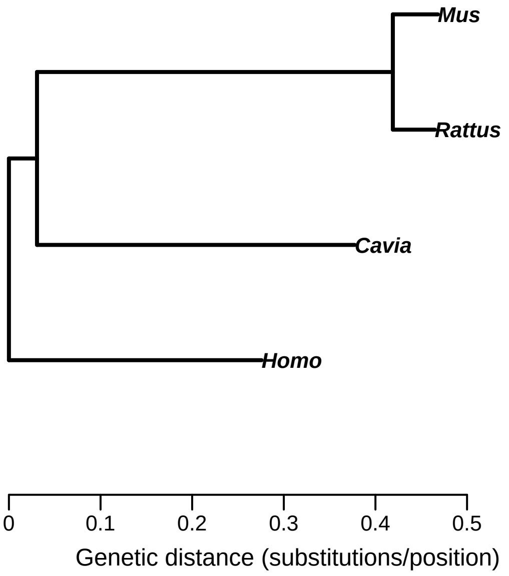

```
Mus     GTGAGAGCAATGGGGTGCGA----TGGGAGCATGGAGCAGTGGGAGTGCTGTTGGGAATCGATAGT----------------------------------GTTAGCAAAG 72
Rattus  GTGAGAGCAACGGGGTGCAA----TGGGAGCATGGAGCAGTGGGAGAGCTGTTGGGAATGGATAGT----------------------------------ATAAGCAAAG 72
Cavia   GTGAGAATGGTGGGGTTCAGGTTGCGGGGGAGGGGGACAGCTGGACCTCAGTGGTGAAGGGAGAATCATTCCAGAAAAGCTCTGCTCCTGAGGAGAAGACATTACCAAAA 110
Homo    GTGAGAGCACCGGGGTGCAGCTT-----GGCGCGAAGCAGCGGGAGCTCAGTCGGGAAGGGGGATTCATCCCAGGCAA----------------------AATCCCAGAA 83

Mus     CAGCAGGG-----GCTAAAAGACAAGTGAATGGCCGCGTGGGGGTGCTGGGCAGGAGATGAGGAGGAAGCAACAACAGTCCTCCCGTGCATCA----------AACAATA 167
Rattus  CAGGAGGGGCTCAGCTAAAAGGCACGTGAATGGCAGGGT-GGGGTATTAGGCAGGAGATGAGGAGGAAACAGCAACAGTCCTCCCATGCATCA----------AACAATA 171
Cavia   CAGTGGGC--------------CAAGGGCAAAGGGAGGCAGCTGGGCAGGAGGGAAGGTAAGCAGGTGGCAGTAATAACTGTCTGCCATCGCAGGGTTGTGGGAGAGTCA 206
Homo    CAAGAGCC-------------------------------------------------------------------CAGCCCTGAGCCCCGCTG----------------- 109

Mus     CTGTGTCTTCTGGATATTTCTTTGAGGTAAGTGTTGACACTGAGGCAGGTAGGTGTCTACAACAAGGAAAGACAAACTGGTGTAGA---CAAGCTGATGCTAAGGCTGGG 274
Rattus  CTGTGTCTTTTGGATATTTCTTTGAGGTAAGTGTTGACACTG----AGGTAGATGTCTACACCAAGGAAAGACAGACTGGGGTAGA---AAAGTGGATGCTAAGACTGGG 274
Cavia   GTGCGTATTCTGAACCGTTCTTTGAGGTGGATGTTGATGCCC---------TGCTTTTACAGTGAGGAAAGTCACATCTGGAGAGATTGCCAGCTCAGGCCCAGCCCAAA 307
Homo    --AGGAGTGCTGGAGCTTCCTT------------------------------------------AGGAAAGCCCGA---------------AGCTTGTGCACGGTCAAGC 160

Mus     CCCATCCCTGCGCTGTGCCAAGGGTTTCTTTGGGCAGCTGACAGTGCTGGGACCAAATGGAGGGCTGGGTGCTCAAGACA-TCTTGTTTCCCTGGACAAGAGCCTTCCCG 383
Rattus  -CCATCCCTGAGCTGTGCCAAGGGTTTCTTTGGGCA-CTGACAGGACTGGGACCAAACGGAGGGCTGGGTGCT-AGGACA-TCTTGTTTCCCTGGACAAGAGCCTTCCCA 380
Cavia   CTCTGCTGAGAGCTGGAGTTGTGCGTT----------------------GGGACAAACAGAGGACTGAATGCTCAGGGTATTTCTGTTTCCCAGGGCAAGATATTACCCT 395
Homo    CGCGATGT-----------------------------------------GCAAAGCAGGGAGGGCTGGGTGCTCAGGCCATTCTTGTTGCCCTGGGGTAGGTCCTTCCCT 229

Mus     GCATCCCAGGTGGACCAGAAGGTGTTAGGATCTCAAGAAGGACCACTGCTGCTAGTGGTC-TCAGGCTGGGGCTCCTTCTCTCCATAG 470
Rattus  GTATCCCAGGTGGACCA-AAGGTGTTGGGGTCTCAAAAAGGACCACTGCTGCTAGTGGTC------CTCAGGCTCCTTCTCTCTGTAG 461
Cavia   GGG-CCTAGGTGGACAGGAGTGTGTCAGGGTCTTCAAGAAAGA-ATGTCAATGGGCAGTCTTTAGGCATAGGTACTCTATCTCCGCAG 481
Homo    GGGCCCCAGATGGACAGCAGTGCATCAGGGTTTTCAAAAAGGGGCTGCTGCCTCGCAGTCCTCAGGCTTGGCCATCCTCTCCTTGCAG 317
```

ENSMUSG00000022096 intron 16

Description: Protein hairless (Hr)
Intron number: 16
Mouse chromosome: 14
Upstream exon length: 165
Downstream exon length: 129
Mouse intron length: 365
Intron alignment length: 495
Total murinae branch length: 0.27071
K_score: 0.06964
Scaling factor: 0.79067

ENSMUSG00000022096 exon 16 (ORF 0)

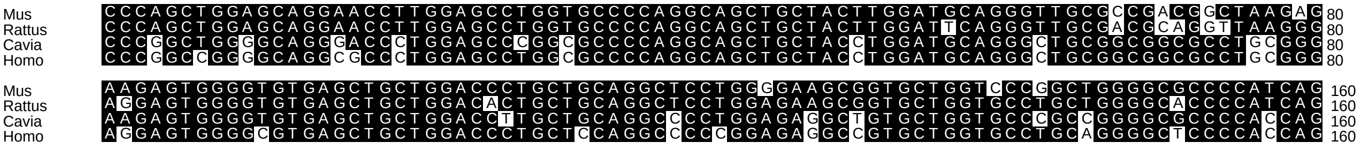

ENSMUSG00000022096 exon 17 (ORF 0)

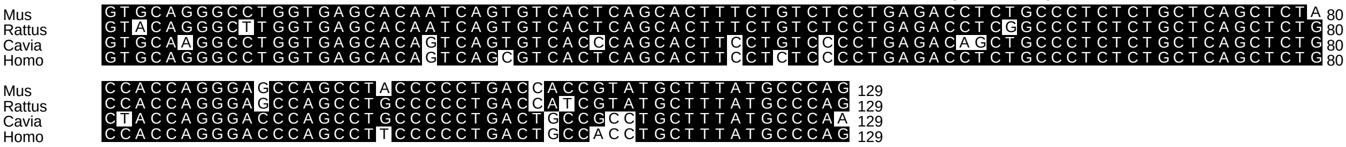

ENSMUSG00000022096_intron_16

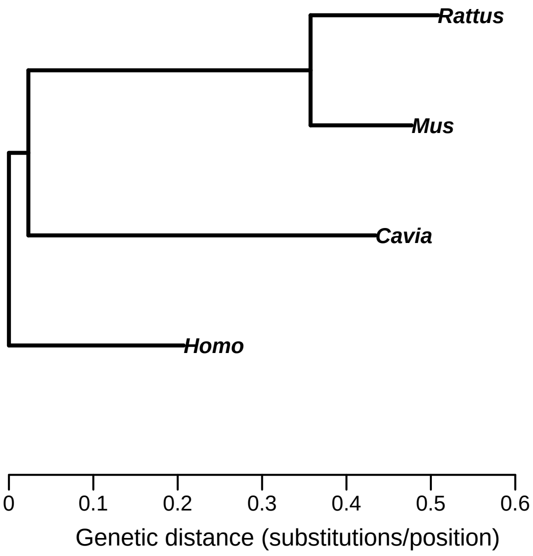

```
Mus     GTGCTTACCTG-GTGGGTGGGGGTTAACAAAAGATCAGAGTATCAGAAGTA----GCAAGGAGTCCTGATCATACAGCATTCTCTACCTGATAGAAAGCTAGGCAGGGCA 105
Rattus  GTGCTTCGCTG-GTGGG---------------------------------------------------------------------------------------------- 16
Cavia   GTGCTTCCCTG-GCGGGGAGGCTCTGTGGAGAGATCGGGCTTTCAGCAACAA---GATCACCCCCAGGGCCAGGCAGTGT-CTCTCTGTGCCTGAGAGTTAG--TAGGGC 103
Homo    GTGCTTCCCAGCGGGCAGGGGCTCTGCGGGGAGATCAGGATGGCAGAAGCGACCCAATCAAGGCCGCAGGCATCCAACATCCCCTCAATACCCAAGAGCCGGGCAGGGCA 110

Mus     GGAAGCCATGGAG--------------------ATAGAAAGCCAAGGGTCTTGAAGTCTTCAAAGCTATGTGGGA----------------------------------- 160
Rattus  ---------------------------------------------------TGGGGTCTTCAAAGCTATGTGGGA----------------------------------- 40
Cavia   GGAGACTGCAGCCCACTGGGGAAAAGAGA----GGAGAGGCTGAGGGATCATGGAGTCAGAGATCCGGTCTGGCT----------------------------------- 174
Homo    GGGAGCCGCACGCTGCTGTGGGGAAGAGATAACGGGGGAGCCAAGAGGCCTTGAAGTCACTGAGCCCAACTGGGACTGGCGCCAGAGTCCACTCCTGGTCTCCTGGGCAT 220

Mus     ----------------------------------CTCTCTCCA------------GCATCCCTCAGGTCTTGTTCTGGCCATGGCCTTTTGAATGTCTGGTTCTGTAACT 224
Rattus  ----------------------------------CTCCCTCCAAAGATCTGATCTAGACTTGGCCAGCTCACTCCTGGCCCTGGCCTTTGGGATGTCTGGTTCTGTAACT 116
Cavia   -------GTAGGGTGCTGATGGAGGGACCT----GTCTCTCCA------------GTGCACCTTTGGCTTGGTTTTGGATGTGACCTGTGGGGTGCCTGGTGCTGCATCC 261
Homo    GCTGATGTCAGGGGGCTCGCAGGGAGACTTTAGGGTCTCTCCA------------GCAGTTCTGAGTCCGGTTTTGGCCACAGGCTTTGAGGTGTCCAGTGGTGCATCC 318

Mus     AGGGTTGGTTACTGTTTTCTGAAAGTTGG---------------------ATAAGGAAGTAGACTTAGTGATCAGAGGCATGGAG--GGGGAATGTGGT-TTGAGCCTCA 310
Rattus  AGGGCT----ACTGCTTTCTGAAAGTTGG----------------------ATGAGAAGTAGACTCACTGATCAGAGGGGTGGAGAAAGGGCGTATAGT-TTAAGCCTCA 199
Cavia   AGGGCT-----TCTGTCCCTGGGAGTTGG----------TGGGGTTCATCACAGGGAGCAGAGCTCAGGAATGGG------GGTAGGGAAATGTGTGACCTCGAACCTTG 350
Homo    AGGAAT-----CTGCTCTCTGAGAGCCAGGGGTCGGGGGTGGTTGTCATTAGAGGAAGCGAACCTCAGGGACCAGGGGGACAGGGAGAGGATGTGTGATCTTGAGCCTCA 423

Mus     ACTCACCCTCTCTTGCCCTTGTTCCGCCCCCCTCCCTGCCCACCCCACGGCACAG 365
Rattus  ACTTACC--CTTTTGCCCTTGTCCC-----------------CGCCATGGCACAG 235
Cavia   GCTGACC--CTCATGCCCACCTCCT-------------------CCCTGGCACAG 384
Homo    ------------CTGTCCATGTCC--------------------CCCCGACACAG 446
```

ENSMUSG00000036565 intron 12

Description: Protein tweety homolog 3 (Ttyh3)
Intron number: 12
Mouse chromosome: 5
Upstream exon length: 174
Downstream exon length: 76
Mouse intron length: 595
Intron alignment length: 781
Total murinae branch length: 0.14914
K_score: 0.04697
Scaling factor: 0.79076

ENSMUSG00000036565 exon 12 (ORF 1)

```
Mus     ATGGGAGGAAGAGACTGCGCCAGGGCCGCGGCAGGCACATGACAGCCTTTACCGAGTGCACATGCCCAGTTTGTACAGC 80
Rattus  ATGGGAAGAGGAGACGGCCCCAGGGCCGAGGCAGGCCCATGACAGCCTTTACCGAGTGCACATGCCCAGCCTGTACAGC 80
Cavia   ATGGAGAAGAGGAGGCTGCCCCAGGGCCGCGGCAGGCACATGACAGCCTTTACCGTGTGCACATGCCCAGTTTGTACAGT 80
Homo    ACGGGGAGGAGGAGGCCGCTCCAGGGCCGCGGCAGGCGCACGACAGCCTCTACCGCGTCCACATGCCCAGCCTGTACAGC 80

Mus     TGCGGAAGCAGCTATGGCAGCGAAGCCAGCATCCCAGCCGCTGCCCACACCGTCAGCAATGCCCCAGTCACCGAGTACAT 160
Rattus  TGCGGAAGCAGCTACGGCAGTGAAGCCAGCATCCCAGCCGCCGCCCACACTGTCAGCAATGCCCCGGTCACCGAGTACAT 160
Cavia   TGCGGCAGCAGCTATGGCAGCGAGACCAGCATCCCAGCTGCGGCACACACAGTCAGCAACGCCCCAGTCACCGAGTACAT 160
Homo    TGTGGCAGCAGCTACGGCAGTGAGACCAGCATCCCGGCCGCGGCCCACACCGTCAGCAACGCCCCGGTCACTGAGTACAT 160
```

ENSMUSG00000036565 exon 13 (ORF 1)

```
Mus     GAGCCAGAATGCCAATTTCCAGAACCCCCGCTGTGAGAACACCCCCCTCATTGGGCGCGAGTCCCCACCGCCCTCA 76
Rattus  GAGCCAGAATGCCAATTTCCAGAATCCCCGCTGTGAGAACACCCCCCTCATTGGGCGCGAGTCCCCACCGCCCTCA 76
Cavia   GAGCCAGAATGCCAATTTCCAGAATCCCCGCTGTGAGAACACTCCTCTCATTGGACGTGAATCCCCACCACCCTCA 76
Homo    GAGCCAGAACGCTAATTTCCAGAACCCCCGCTGTGAGAACACCCCACTCATTGGGCGCGAGTCCCCGCCGCCCTCA 76
```

ENSMUSG00000036565_intron_12

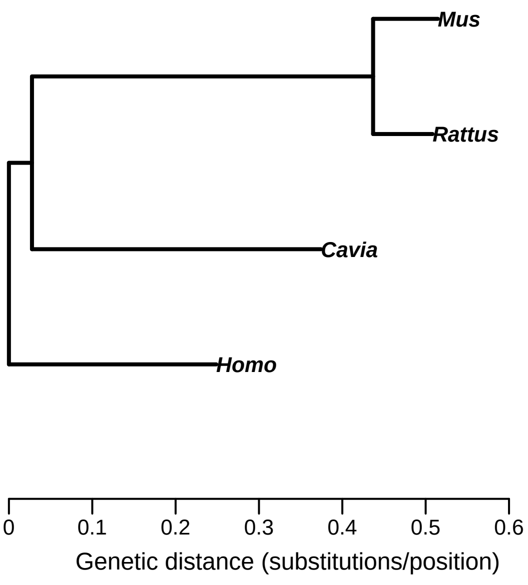

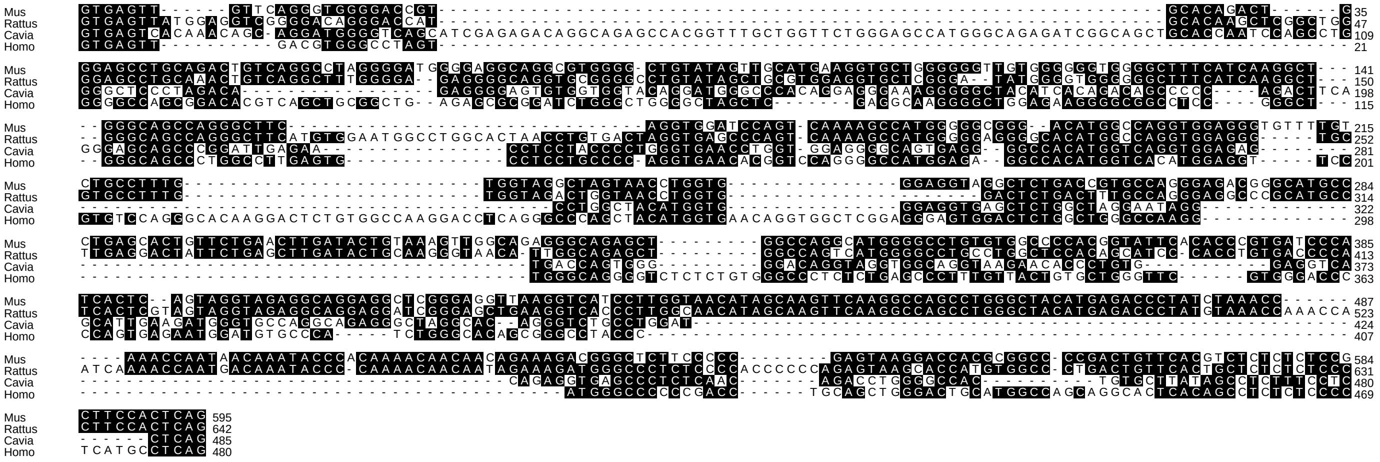
Mus
Rattus
Cavia
Homo

ENSMUSG00000027709 intron 12

Description: Methylcrotonoyl-CoA carboxylase subunit alpha, mitochondrial (Mccc1)
Intron number: 12
Mouse chromosome: 3
Upstream exon length: 110
Downstream exon length: 217
Mouse intron length: 1504
Intron alignment length: 1834
Total murinae branch length: 0.32793
K_score: 0.07336
Scaling factor: 0.79105

ENSMUSG00000027709 exon 12 (ORF 2)

```
Mus     GAGATGAAGTCTCGGTGCATTACGATCCCATGATCGCAAAGCTCGTTGTGTGGGCCTCAGACCGCCAGTCAGCCCTGTCG 80
Rattus  GAGATGAAGTCTCGGTGCATTACGACCCCATGATCGCAAAGCTGGTGGTGTGGGCCTCAGATCGCCAGTCAGCCCTGTCA 80
Cavia   GAGATGACATTTCTGTCCATTATGACCCCATGATCGCAAAGCTGGTCGTGTGGGCTGCAGACCGCCAGGCCGCCCTAACG 80
Homo    GAGACGAAGTTTCCGTGCATTATGACCCCATGATTGCGAAGCTGGTCGTGTGGGCAGCAGATCGCCAGGCGGCATTGACA 80

Mus     AAGCTGAGGTACTGCCTTCATCAGTACAAC 110
Rattus  AAACTGAGATACAGTCTTCATCAGTACAAC 110
Cavia   AAACTGAGGTACAGCCTGCGTCAGTTCAAC 110
Homo    AAACTGAGGTACAGCCTTCGTCAGTACAAT 110
```

ENSMUSG00000027709 exon 13 (ORF 0)

```
Mus     ATTGTCGGCTTGCGCAGCAACGTGGACTTCCTGCTCCGGCTCTCCGGCCACCCAGAGTTTGAGGCTGGGAACGTGCACAC 80
Rattus  ATTGTTGGCTTGCGCACCAATGTGGACTTCCTGCTCCGGCTCTCCGGCCACTCAGAGTTTGAAGCTGGGAATGTACACAC 80
Cavia   ATTGTCGGCCTGCACACCAATGTTGACTTCCTGCTCCACCTGGCCGCACACCCGGCCTTTGCCGCTGGGGCTGTGCACAC 80
Homo    ATTGTTGGACTGCACACCAACATTGACTTCTTACTCAACCTGTCTGGCCACCCAGAGTTTGAAGCTGGGAACGTGCACAC 80

Mus     GGACTTCATCCCTCAGCACCACAAGGACCTGCTGCCGAGTCACAGCACTATAGCCAAAGAGTCTGTGTGCCAGGCAGCTC 160
Rattus  TGACTTCATCCCTCAACACCACAAGGACCTGCTGCCCACTCACAGCACCATAGCCAAGGAATCTGTGTGCCAGGCAGCCT 160
Cavia   CGATTTCATTGCCCAGCACCACGAGGAGCTGCTGCCCAGCAGGAGGGCGGTGGCCAGCGAGTATCTGTGCCAGGCTGCAC 160
Homo    TGATTTCATCCCTCAACACCACAAACAGTTGTTGCTCAGTCGGAAGGCTGCAGCCAAAGAGTCTTTATGCCAGGCAGCCC 160
```

ENSMUSG00000027709_intron_12

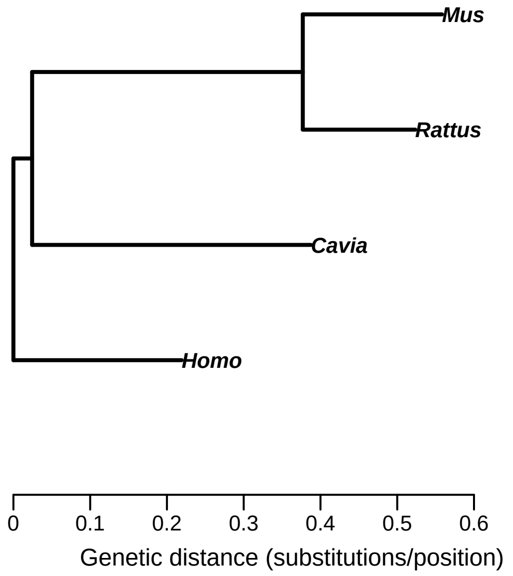

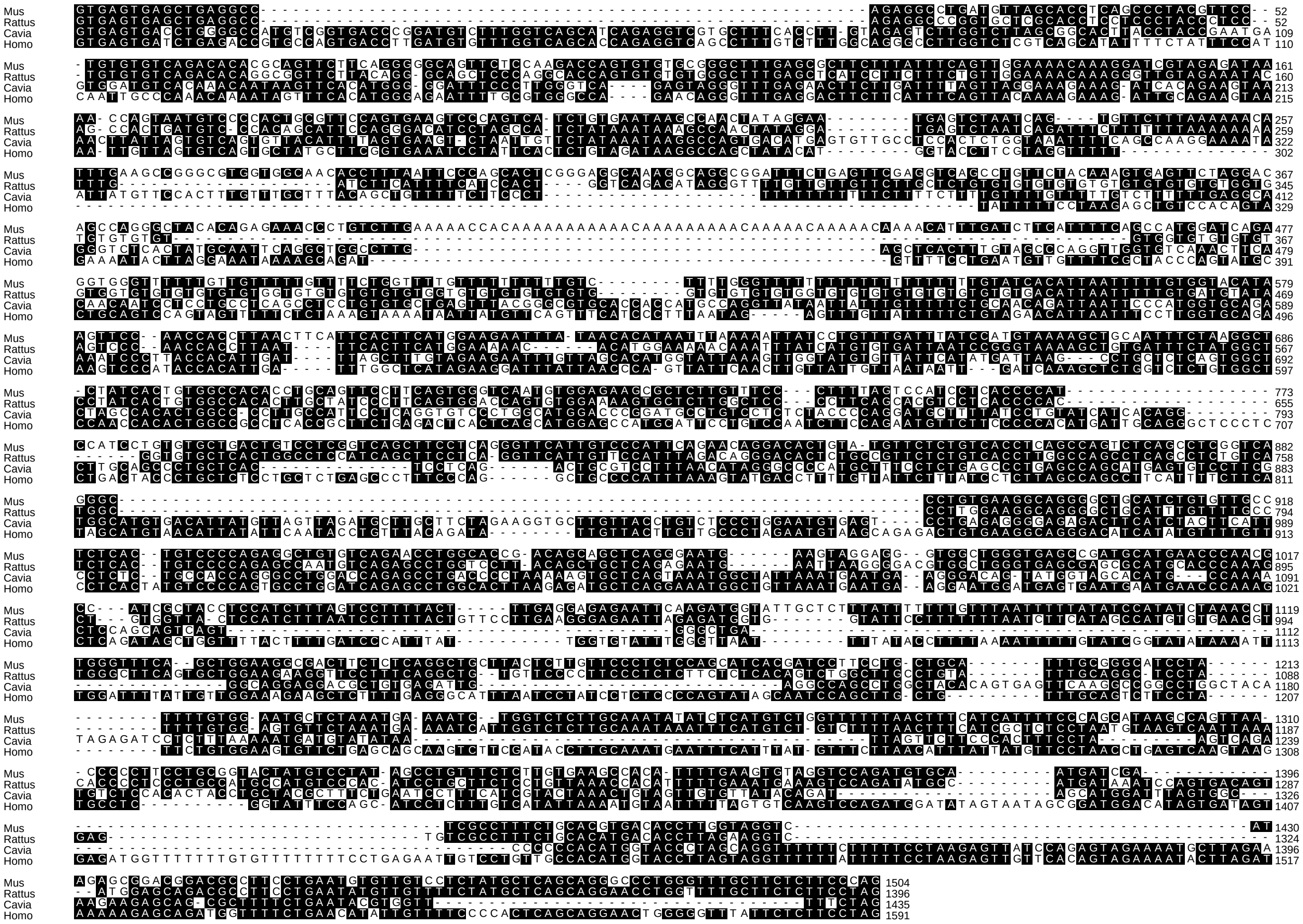

ENSMUSG00000045672 intron 58

Description: Collagen alpha-1 (Col27a1)
Intron number: 58
Mouse chromosome: 4
Upstream exon length: 169
Downstream exon length: 110
Mouse intron length: 1094
Intron alignment length: 1940
Total murinae branch length: 0.21201
K_score: 0.037
Scaling factor: 0.79117

ENSMUSG00000045672 exon 58 (ORF 0)

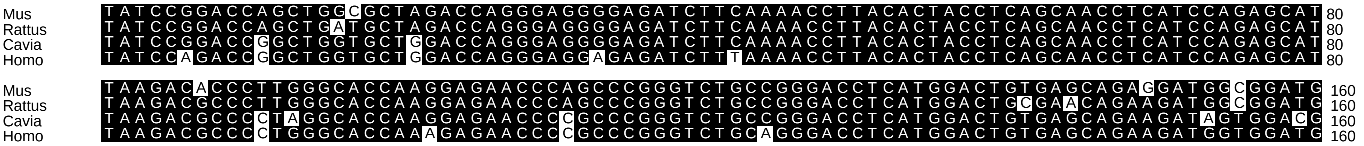

ENSMUSG00000045672 exon 59 (ORF 2)

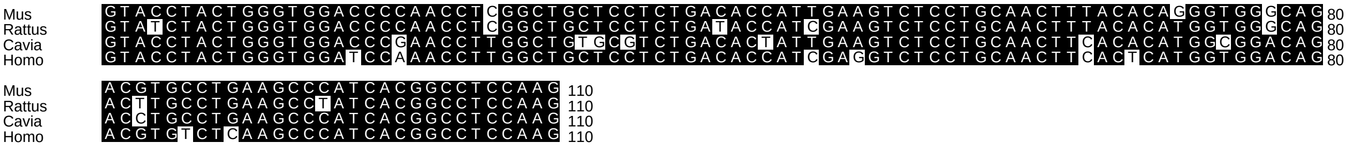

ENSMUSG00000045672_intron_58

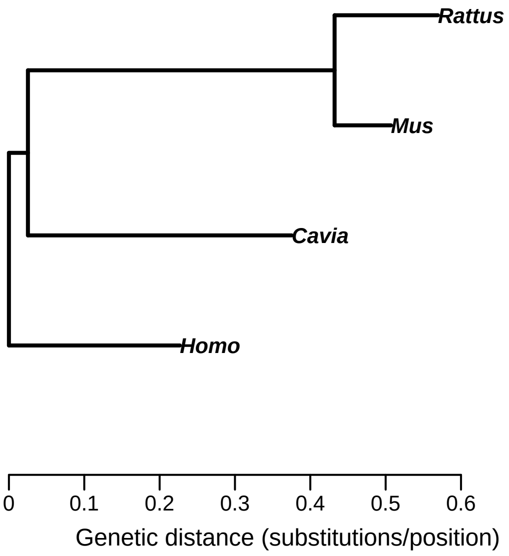

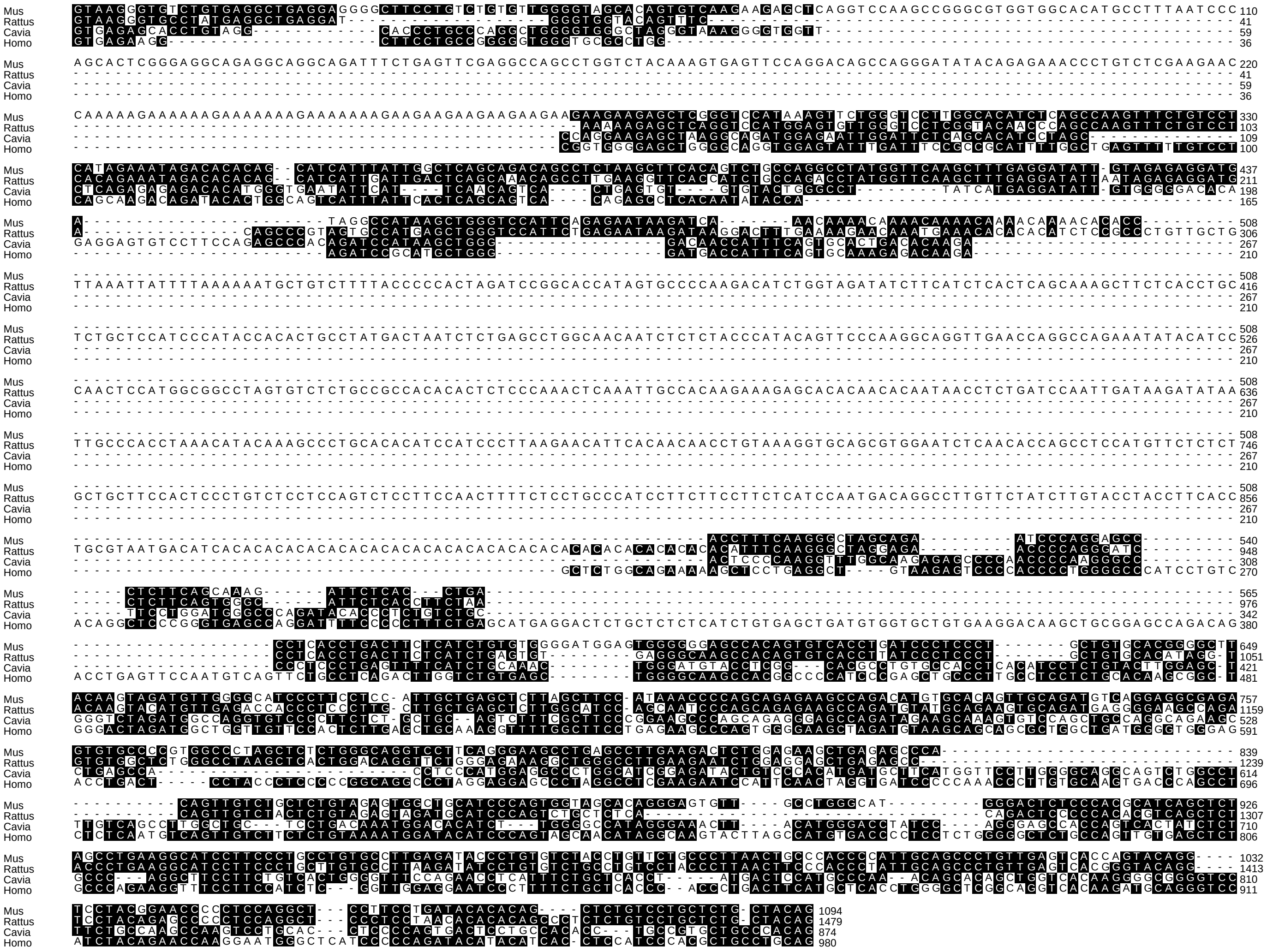
Mus
Rattus
Cavia
Homo

ENSMUSG00000038095 intron 20

Description: Protein strawberry notch homolog 1 (Sbno1)
Intron number: 20
Mouse chromosome: 5
Upstream exon length: 136
Downstream exon length: 113
Mouse intron length: 935
Intron alignment length: 1083
Total murinae branch length: 0.24992
K_score: 0.03585
Scaling factor: 0.79339

ENSMUSG00000038095 exon 20 (ORF 0)

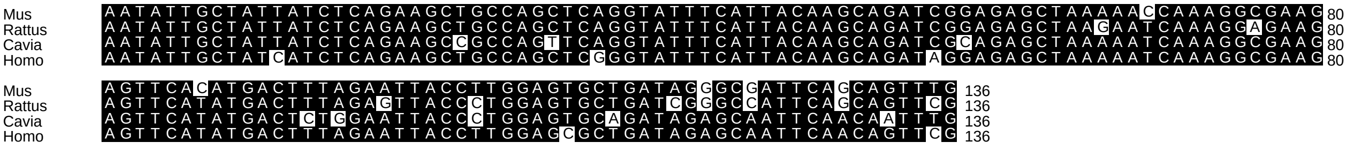

ENSMUSG00000038095 exon 21 (ORF 2)

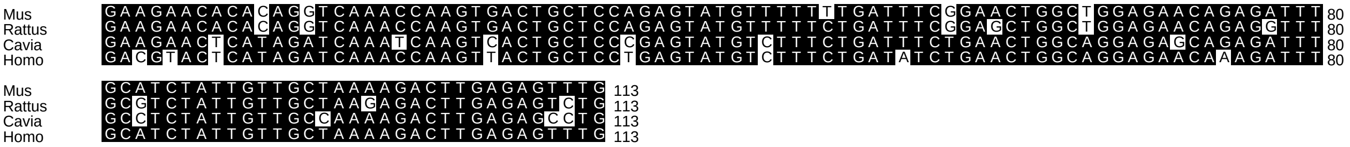

ENSMUSG00000038095_intron_20

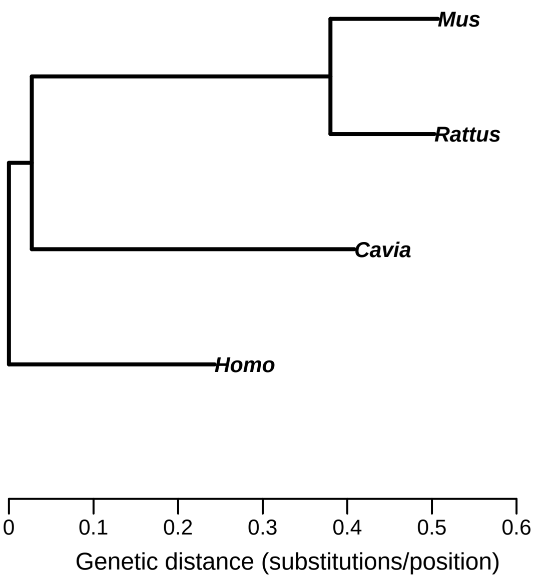

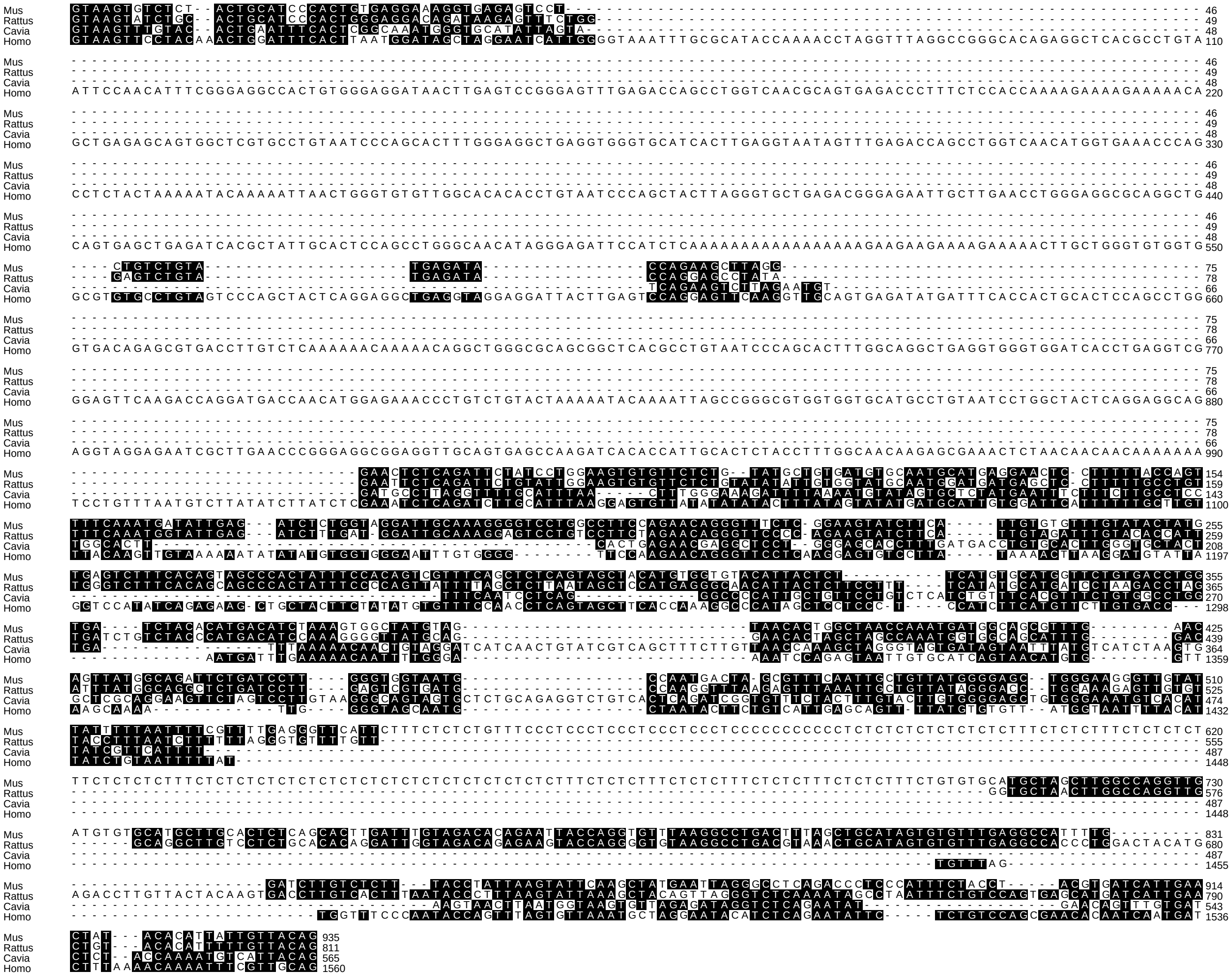
Mus
Rattus
Cavia
Homo

ENSMUSG00000022742 intron 2

Description: Coproporphyrinogen-III oxidase, mitochondrial Precursor (Cpox)
Intron number: 2
Mouse chromosome: 16
Upstream exon length: 144
Downstream exon length: 111
Mouse intron length: 664
Intron alignment length: 695
Total murinae branch length: 0.27407
K_score: 0.08516
Scaling factor: 0.79456

ENSMUSG00000022742 exon 2 (ORF 2)

```
Mus     GAGGAGGTGGCATCACCTGTGTGCTTCAGGACGGGCGTGTGTTTGAAAAGGCCGGGGTGAGCATTTCCGTCGTTCATGGG 80
Rattus  GAGGTGGTGGCATCACCTGTGTGCTTCAGGACGGGCGTGTGTTTGAGAAGGCCGGGGTGAACATTTCTGTCGTTCATGGG 80
Cavia   GAGGTGGTGGCATCAGCTGTGTACTTCAGGATGGTCGTGTTTTTGAAAAGGCTGGAGTGAGCATTTCTGTTGTTCATGGG 80
Homo    GAGGTGGCGGCATCAGCTGTGTACTTCAAGATGGGTGTGTTTTCGAAAAGGCTGGGGTGAGCATTTCTGTTGTTCATGGA 80

Mus     AATCTTTCTGAGGAAGCAGCGAACCAAATGAGAGGCAGAGGCAAAACTCTGAAGACGAAAGATA 144
Rattus  AATCTTTCTGAGGAGGCAGCAAACCAAATGAGAAGCAGAGGAAAAGCCCTGAAGAAGAAAGATG 144
Cavia   AATCTTTCTGAAGAAGCAGCAAAACAGATGAGAAGCAGAGGAAAAGTTCTGAAGACTAAAGATG 144
Homo    AATCTTTCAGAGGAAGCTGCAAAACAAATGAGAAGCAGAGGAAAAGTTCTGAAGACTAAAGATG 144
```

ENSMUSG00000022742 exon 3 (ORF 2)

```
Mus     GTAAATTGCCATTTACTGCTATGGGTGTAAGTTCTGTGATTCACCCCAAGAATCCTTATGCGCCCACCATGCATTTCAAC 80
Rattus  GTAAATTGCCGTTTACTGCTATGGGTATAAGCTCCGTGATTCACCCTAAGAATCCTTATGCACCCACCATGCATTTCAAC 80
Cavia   GTAAATTACCATTTTCTGCTATGGGTGTGAGCTCTGTTATCCATCCTAAGAATCCTCATGCTCCCACTATCCATTTTAAC 80
Homo    GTAAATTGCCATTTTGTGCTATGGGCGTGAGCTCTGTTATCCACCCCAAGAATCCTCATGCTCCTACTATCCATTTCAAC 80

Mus     TACAGATACTTTGAAGTAGAGGAAGCTGACG 111
Rattus  TATAGATACTTCGAAGTAGAAGAAGCTGATG 111
Cavia   TACAGATATTTTGAAGTGGAAGAAGCAGATG 111
Homo    TACAGATACTTTGAAGTAGAAGAAGCTGATG 111
```

ENSMUSG00000022742_intron_2

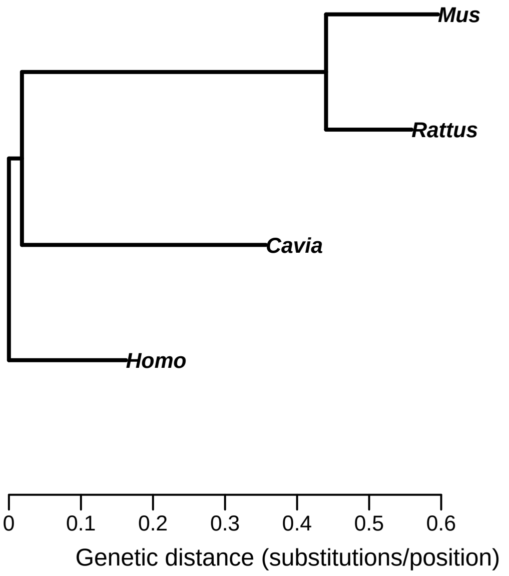

```
Mus     GTAAGTC-CGA----------------TAACTCTCCCTGCCAAC---TCTTTTCCCACCAGTTTTACTTAAAAATAGCGCTATTTATTTATTTATTTATTTATTTATTTT 90
Rattus  GTAAGTC-AGA----------------TATCGTTCCCGGCCAAC----TCTTTCCCGCCAGTTTTACTTAAAAATCAC-----------------------TTTTTGCTT 66
Cavia   GTAAATTGAGATTATTTTAACAGTCTTCAATAATCTCTGCAACC---GTTTTTTCCGTAAGTTTTATTGATAAAT----------------------------------- 72
Homo    GTAAATC-AGACAGTCTTGATAGTCTCTAATAGTCCCTGCAAACCTTATTTTGCCCACAAGTTTTATTTATAAAATAA-----------------------ATATTGCTT 86

Mus     CTGGAACTACCCTTGGATGTGTATGCCCAGTGTACTGGCTGGTTTTGTGTGTCAACTTGACACAGGCTGGAGTTATCCCAGAGAAAGGAGCTTCAGTTGGGGAAGTGCCT 200
Rattus  CTGGAACAGCCATGTGGGATGTAT-------------------------------------------------------------------------------------- 90
Cavia   -TAACACCATTTGGATGGATACAT-------------------------------------------------------------------------------------- 95
Homo    CTGAAAAAAGCCATTCGGGTTCAT-------------------------------------------------------------------------------------- 110

Mus     CCATGAGATCCAGCTGTAAGGCATTTTCTCAATTAGTGATCAAGGGGGAAAGGCCCCTTGTGGGTGGGATCATCTCTAGGCTGGTAGTCTTGGGTTCTATAAGAAAGCAA 310
Rattus  -------------------------------------------------------------------------------------------------------------- 90
Cavia   -------------------------------------------------------------------------------------------------------------- 95
Homo    -------------------------------------------------------------------------------------------------------------- 110

Mus     GCTAAGCAAGCCAGGGGAAGCAAGCCAGTAAGTAACATCCCTCCGTGGCCTCTGCATCAGCTCCTGCTTCCTGACCTGCTTGAGTTCTAGTCCTGACTTCCTTTGGTGAT 420
Rattus  -------------------------------------------------------------------------------------------------------------- 90
Cavia   -------------------------------------------------------------------------------------------------------------- 95
Homo    -------------------------------------------------------------------------------------------------------------- 110

Mus     CAACAGCAAAGTGGAAAGTGTAAGCGGAATAAACCCTTTCCTCCCCAACTTGCTTCTTGGTCATGTTGATAGAAACCCTGACTAAGACACCCAGTAAGCGTGTCCGCATG 530
Rattus  --------------------------------------------------------------------------------------GTGCCCGGTAAGCGTGCTTGCATG 114
Cavia   --------------------------------------------------------------------------------------ATACTAGATAACCATGTCCAAAAT 119
Homo    --------------------------------------------------------------------------------------ATGCC------GCACGTTGACAAA 128

Mus     CGTGAATTGTGAAAATTGTGACTCAGAGACAAGTTACAAAGCAGCCTTTGATAGTGGGTTCAGAGATCAGGAGGGAGTAAATT-GTGT----CAAGCCA-----GGCGGC 630
Rattus  CGTTAATCGTGAACATTGTAACTCAGAGACAAGGGACAAAGCAGCTTCTGATAGTGGACTCAGAAATCATGAGGGAGCAACTT-GTGC----CAAATCA-----GGCCAC 214
Cavia   TACTTTTTGAAAATTATAT-TTTGAGAAACATATTGGAAAACATCTTTTA--AATTGATTCAGAAACCACATGAGAGTTAATTTGTGTATTTCAAGCCT-----GGAAAT 221
Homo    TACTTTTTTAAAATTATGTTTTTAAGTGACAAATTAAAAAGCAGCTTTTGAAAATTGACTTGGAAATCATATTAGAGTGAAATTGTAT----CAAGGCATTCAAGGTAAC 234

Mus     CAGATGCATCTAACTGTGT-TGTTTTACTTTCCAG 664
Rattus  CCAATGCATCCAACCATGT-TGTTTTACTTTCCAG 248
Cavia   TGAATGTACTCTCTCCC---TGTTTTACTTTCCAG 253
Homo    TGAATATAGCTCATGACTCATATTTTACTTTCCAG 269
```

ENSMUSG00000015002 intron 17

Description: Protein EFR3 homolog A (Efr3a)
Intron number: 17
Mouse chromosome: 15
Upstream exon length: 68
Downstream exon length: 128
Mouse intron length: 874
Intron alignment length: 1842
Total murinae branch length: 0.16193
K_score: 0.04958
Scaling factor: 0.79504

ENSMUSG00000015002 exon 17 (ORF 0)

```
Mus     GTTATTGAAACTCGAACTATGGAAGCCCCTTATTTTCTACCAGAGCATATTTTCCGAGATAAATGCAT 68
Rattus  GTTATTGAAACTCGAACTATGGAAGCCCCTTATTTTCTACCAGAGCATATTTTCAGAGATAAGTGCAT 68
Cavia   GTTATTGAAATTCGAACTATGGAAGCTCCCTATTTTCTACCAGAACATATTTTCAGAGATAAGTGCAT 68
Homo    GTTATTGAAATTCGAACTATGGAAGCCCCTTATTTTCTACCAGAGCATATCTTCAGAGATAAGTGCAT 68
```

ENSMUSG00000015002 exon 18 (ORF 1)

```
Mus     GCTTCCGAAATCTTTAGAGAAACATGACAAAAATTTATATTTTTTGACCAACAAGATTGCGGAGTCCCTGGGTGGCAGCG 80
Rattus  GCTTCCGAAATCTTTAGAGAAGCATGACAAAAATTTATATTTTTCTGACCAACAAGATTGCAGAATCCCTGGGTGGAAGCG 80
Cavia   GCTTCCAAAATCTTTAGAGAAGCATGATAAAAATTTATATTTTTCTGACCAACAAGATTGCAGAATCACTCGGTGGAAGTG 80
Homo    GCTTCCAAAATCTTTAGAGAAGCATGAAAAAGATTTGTACTTTCTGACCAACAAGATTGCAGAGTCGCTAGGTGGAAGTG 80

Mus     GGTACAGTGTGGAGAGGCTGACAGTTCCTTATGTGCCACAGGTGACAG 128
Rattus  GGTACAGTGTGGAGAGGCTGTCGGTTCCTTATGTGCCACAGGTGACAG 128
Cavia   GATATGGTATTGAGAGATTATCGGTACCATATGTACCACAAGTAACAG 128
Homo    GATATAGTGTTGAGAGATTGTCAGTTCCGTATGTACCACAAGTAACAG 128
```

ENSMUSG00000015002_intron_17

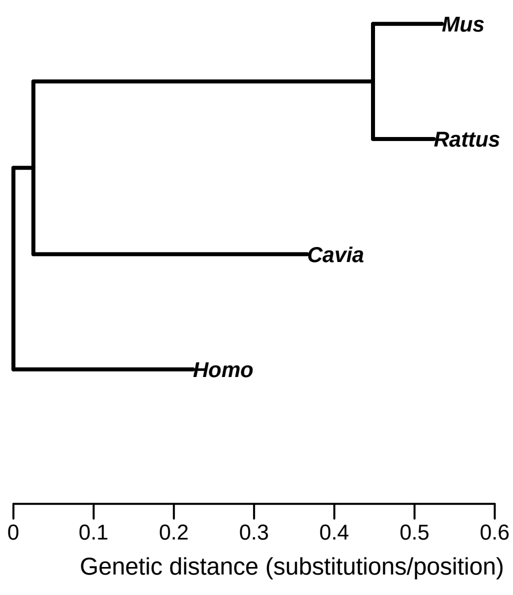

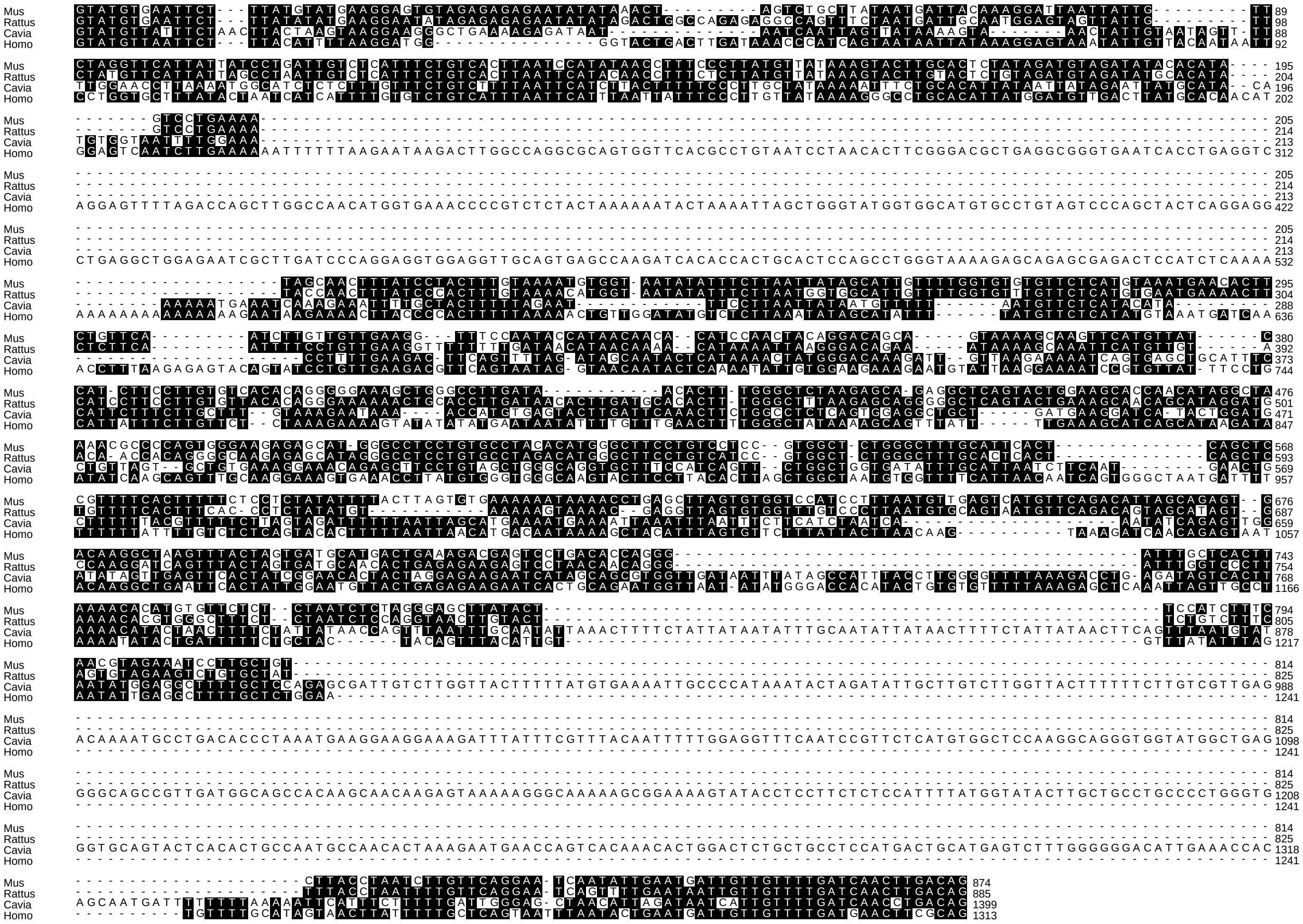
Mus
Rattus
Cavia
Homo

ENSMUSG00000033105 intron 8

Description: Lanosterol synthase (Lss)
Intron number: 8
Mouse chromosome: 10
Upstream exon length: 109
Downstream exon length: 119
Mouse intron length: 425
Intron alignment length: 830
Total murinae branch length: 0.20571
K_score: 0.08934
Scaling factor: 0.79516

ENSMUSG00000033105 exon 8 (ORF 0)

```
Mus     GAACTCTATGTGCAGGATTACGCCAGCATCGATTGGCCAGCGCAGAGGAACAACGTGTCCCCCGATGAGATGTACACGCC 80
Rattus  GAACTCTATGTGGAGGATTATGCCAGCATCGATTGGCCAGCACAGAAGAACAACGTGTGCCCCGATGACATGTACACGCC 80
Cavia   GAGCTTTACGTGGAGGACTATGACAGCATCGACTGGCCAGCACAGAGAAACAACGTGTCTCCTGAAGATGTGTACACACC 80
Homo    GAGCTCTATGTGGAGGACTTCGCCAGCATTGACTGGCTGGCGCAGAGGAACAACGTGGCCCCCGACGAGCTGTACACGCC 80

Mus     ACACAGCTGGCTGCTGCATGTGGTGTATG 109
Rattus  ACACAGTTGGCTGCTGCACGTGGTGTATG 109
Cavia   ACACAGCTGGTTGCTGCGTACAGTGTATG 109
Homo    GCACAGCTGGCTGCTCCGCGTGGTATATG 109
```

ENSMUSG00000033105 exon 9 (ORF 2)

```
Mus     GACTCCTCAACCTGTATGAACGTTTCCATAGCACCAGCCTGCGGAAGTGGGCCGTCCAGATGCTGTATGAACACATTGCA 80
Rattus  GACTCCTCAACCTGTATGAACGTTTCCACAGTACCAGCCTGCGGAAGTGGGCCATCCAGTTGCTGTATGAACATGTTGCA 80
Cavia   CATTCCTCAACCTGTATGAGCGCTTCCACAGCACCTACCTGCGGCACAGGGCCATCCAGGTGCTGTACGAGCACATCGCT 80
Homo    CGCTCCTCAACCTGTATGAGCACCACCACAGTGCCCACCTGCGGCAGCGGGCCGTGCAGAAGCTGTATGAACACATTGTG 80

Mus     GCTGATGACTGTTTCACTAAGTGCATCAGCATCGGCCCG 119
Rattus  GCTGATGATCGGTTCACAAAATGCATCAGCATTGGCCCG 119
Cavia   GCCGATGACCGCTTCTCCAAGAACATCAGCATTGGCCCG 119
Homo    GCCGACGACCGATTCACCAAGAGCATCAGCATCGGCCCG 119
```

ENSMUSG00000033105_intron_8

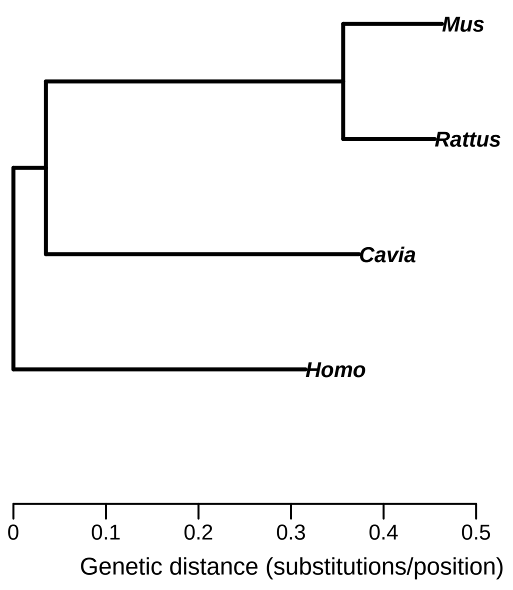

```
Mus     GTGAGTGCCTCCTGAGGGTTGGGAGAGCCCGGGGGCGTGCACAGTTAAGATGTTGCTCAGCTCT--CCACATTAATTCCTCCACTCTGTCGGTGCAACAAATGACCTCTG 108
Rattus  GTAGGTG-CTTCTGAAGGTTGGGAGAGCCTGGGTA---GTGCAGTTAAGATGTTTTTGG---CT--CTCCATTAATTCATTCACTCTGTCAGT----------------- 84
Cavia   GTAGGTGTTTCGTAAGG---CAGTGATCCTGGGCA--ACCAAAGTCAAGGAGTCACTCATTTATGCCCCCATTAGTTCATTTGTCC--TGATTATGGTAGTGGACAACCT 103
Homo    GTGAGCGCCTCCTGAGGGGCCGGCAGGGCAGCCCAGGGTCAGGGTCAGGGTGTCGCCCA--------CTCATTCACGCACTCATCCCCTGCCAGCGGCACTGGGCCACCT 102

Mus     TGCTGGGGCCCTGGAGTGGGAGCTGGAA--AGACAGCGTTGCTCTGGTTCCCCAACAACAGCCAACTGCTGCTCAGTGATACCCCCTCAGCATCCCCTAAGCAGGGCAGG 216
Rattus  -------GCCCTGGAGTAAGAGCTGAAAGCACATATCCTTGCTGTGGTTCCCCCACAACGATCAGCTGCTGCTCAGTGATCCCCATTCAGCATGGCCTGAGCAGGGCAGG 187
Cavia   CCTCTG------------------------------------------------------------------------------CTGGGCCCTGTCCTGAGCAACACTG- 134
Homo    CCTCTGTGCCAGGCCCCAGGGGGCGGGATCTCATCGCCCTGCCCCTCCACCCTGAGAACCAGCTGGTCTTCTAC-------TCTCAGGAGTCCACCCTGTGCAAGGGTG- 204

Mus     ACATGGTGGGAGGTCTGAGGCAGTCCTTCCTTGATGGGA---AGGAGGAAGCCC--AGATGAGGC---AGAGTGAGCGGGAGGAGGGATCCCAGGGGGGAAGAGAGGTCC 318
Rattus  ACGTGGCGGGAGGTATACAGCAGTCCTTCCTAGATGGGAAGGAGGAGGAAGCAC--AGATGAGGC---AGAGCGAGTGGGAGGAGGGATCCCAGGGGAAGAGAGAAGTCC 292
Cavia   ---------------------AGGACCACCTGGGCAGGGCAGAAGAGGAGGCAC--AGGCAAGGGT--GGAGTGAGTAGAATCAGGAGTT----GGGGAGAGAGGGGCTG 215
Homo    -TGTGGTAGGAGGTGTGGGGCAGCCCCTCCTGGGCAGGG---AAGGAGGAGCTCAGAGACCAGGCCTGGGGGTGGGTGGGAGGGGGAAACCC--TGGGAAGGGCAAGTCC 308

Mus     AGATGCTGTAATGTGGAGGACCCATCCCCGTCTGAAAGACTAGGGTGCAGCCTGCCCCACGTATCTCTCCTGTTGCCAGGTGTCCACTAATGTCTTCTCCCCCACAG 425
Rattus  AG-TGCTGTGCTGTGGAGGACCCGTTCCCAGCTGAATAACTAGGACACAGCCTG--CCACATATCTCTCCTGTTGCTGGGTGTCCACTAATGCCTTCTCCCCTGCAG 396
Cavia   AGACACTGTTCCCTGTTAGAAGG-----CTATGGGAATATCAGGTTGGGGCCAG--------AACATATCCCATGTCTGGTGCCCACTGAC-ACTGCTGCCCTGCAG 308
Homo    AGGCGTTGCAGTGCATGCAGCTC-----------------CAGGCTGAGGCCAG-----------TGTCATGGTGTCTGGCATCCACTGAC-CCCTGTCCCCTGTAG 386
```

ENSMUSG00000052301 intron 4

Description: Double C2-like domain-containing protein alpha (Doc2a)
Intron number: 4
Mouse chromosome: 7
Upstream exon length: 110
Downstream exon length: 127
Mouse intron length: 1162
Intron alignment length: 1960
Total murinae branch length: 0.20684
K_score: 0.02466
Scaling factor: 0.79536

ENSMUSG00000052301 exon 4 (ORF 0)

```
Mus     GCCAATAAGCTAAAAACCAAGACACAGAGGAACACACTGAACCCTGTGTGGAATGAGGAGCTGACGTACAGCGGGATCAC 80
Rattus  GCCAATAAGCTAAAAACCAAGACACAGAGGAACACACTGAACCCAGTGTGGAACGAGGAGCTGACGTACAGTGGAATCAC 80
Cavia   GCCAATAAACTAAAAACGAAGACTCAGAGGAACACTCTGAATCCTGTGTGGAACGAGGACCTGATGTACAGTGGGATCAC 80
Homo    GCCAATAAGCTAAAAACGAAGACTCAGAGGAACACACTGAATCCCGTGTGGAATGAGGACCTGACTTACAGCGGGATCAC 80

Mus     GGATGATGACATCACCCACAAGGTGCTCAG 110
Rattus  AGATGACGACATCACCCACAAGGTGCTCAG 110
Cavia   GGATGATGACATTACTCACAAGGTGCTCAG 110
Homo    AGATGACGACATCACGCACAAGGTGCTCAG 110
```

ENSMUSG00000052301 exon 5 (ORF 1)

```
Mus     GATCTCTGTCTGTGATGAGGACAAGCTGAGCCACAATGAATTCATTGGGGAGATCCGAGTGCCCCTCCGCCGCCTCAAGC 80
Rattus  GATCTCGGTCTGTGATGAGGACAAGCTGAGTCACAATGAATTTATTGGGGAGATCCGAGTGCCCCTCCGCCGCCTCAAGC 80
Cavia   GATCTCTGTGTGTGATGAGGACAAGCTGAGCCACAATGAGTTCATCGGGGAGATTCGAGTGCCCCTCCGCCGCCTCAAGC 80
Homo    GATCGCCGTCTGTGATGAGGACAAGCTGAGTCACAATGAGTTTATTGGGGAGATCCGCGTGCCCCTCCGCCGCCTCAAGC 80

Mus     CTTCACAGAAGAAGCATTTTAACATCTGCCTTGAGCGCCAGGTCCCG 127
Rattus  CTTCACAGAAGAAGCATTTTAACATCTGCCTTGAGCGTCAGGTCCCG 127
Cavia   CTTCACAGAAGAAGCATTTTAACATCTGCCTCGAGCGCCAGGTCCCG 127
Homo    CTTCGCAGAAGAAGCATTTTAACATCTGCCTCGAGCGCCAGGTCCCG 127
```

ENSMUSG00000052301_intron_4

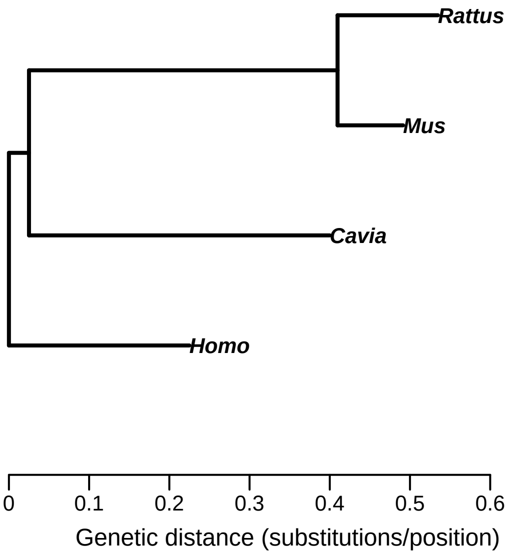

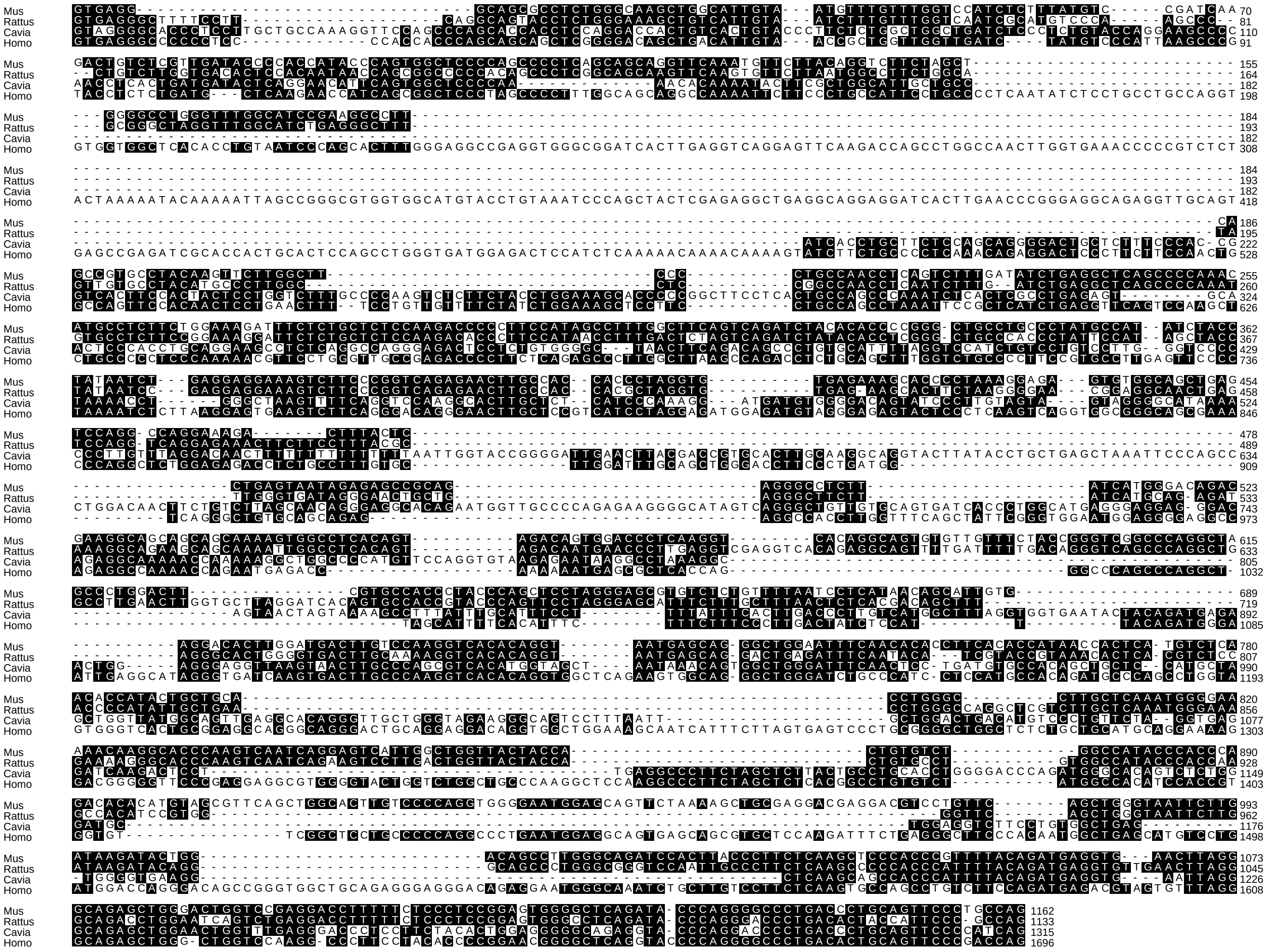

ENSMUSG00000033159 intron 1

Description: Uncharacterized protein C2orf24 homolog (1810031K17Rik)
Intron number: 1
Mouse chromosome: 1
Upstream exon length: 69
Downstream exon length: 109
Mouse intron length: 409
Intron alignment length: 478
Total murinae branch length: 0.15579
K_score: 0.04287
Scaling factor: 0.79671

ENSMUSG00000033159 exon 1 (ORF 0)

```
Mus     ATGGACCTGACTGGGCTCCTGCTGGACGAAGAAGGCGCCTTCTCCCTCACCGGTTTCCAAGACTTCATG 69
Rattus  ATGGACCTGGCTGGGCTCCTACTGGACGAAGAAGGCACCTTCTCCCTCTCTGGTTTCCAGGACTTCACG 69
Cavia   ATGGACTTGGCCGGGCTT---CTGGACGAAGAAGGCACCTTCTCCCTCACTGGCTTTCAAGACTTCACG 66
Homo    ATGGACCTGACCGGGCTCCTGCTGGACGAAGAAGGCACCTTCTCCCTCGCCGGCTTCCAGGACTTCACG 69
```

ENSMUSG00000033159 exon 2 (ORF 0)

```
Mus     GTCCTTCCAGGACACCAAAAGCTAAGTGCTCGAATCCGAAGGAGACTCTATTATGGCTGGGACTTGGAGACAGACTGCAG 80
Rattus  TTTCTTCCAGGACACCAAAAGCTGAGCGCTCGAATCCGAAGGAGACTCTATTATGGCTGGGACTTGGAGACAGACTGCAG 80
Cavia   TTCCTCCCAGGACACCAGAAACTGAGTGCCCGGATCAGAAAGCGACTGTACTATGGCTGGGACTGGGAGGCCGACTGCAG 80
Homo    TTCCTCCCAGGACACCAGAAGCTGAGTGCCCGGATCCGAAGGAGGCTCTACTATGGCTGGGACTGGGAAGCCGACTGTAG 80

Mus     CCTGGAGGAGCTCTCCAGCCCTGTGGCAG 109
Rattus  CCTGGAGGAGCTCTCCAGCCCTGTGGCAG 109
Cavia   CCTGGAGGAACTCTCCAGCCCCGTGGCAG 109
Homo    CCTGGAGGAGCTCTCCAGCCCGGTGGCAG 109
```

ENSMUSG00000033159_intron_1

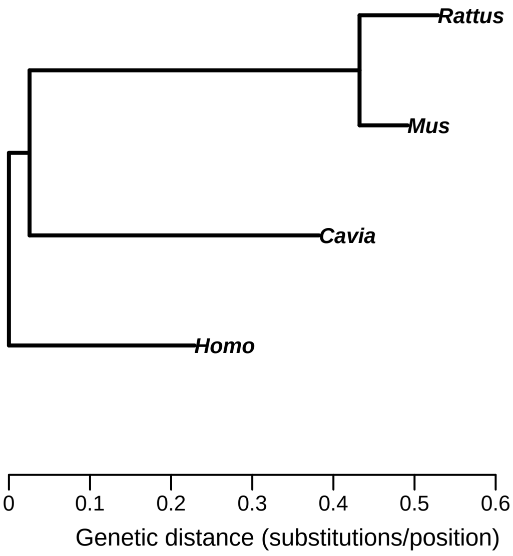

```
Mus     GTGAGAG----GTCCGCCTGTTCTCTGCCTCTGGGAGTCAGAACTCCCAGGCGTTC-TCCTCCAGACCCGAGCGGAATGG-GAGCTCTCTTACTTGTTATGCCCTGGAGT 104
Rattus  GTGAGAGCGCAGTCCGCCTGTTCTCTGCCTTTGGGAGTCGGAACTCCTGGGTGT-----------------------------------TTACTTGCTATGCTCTGTTTT 75
Cavia   GTGAGCGCGCAGCCCCCTGCTTACCC-----TAACGCCCGCGCCTAGCCGCTGCACTCCCTCCCGGCCCAAGAAAGGTAGCTGGCTCGCTTGATGGTCTTGCCCTGGCCT 105
Homo    GTGAGTGGGGGTCCCGCCCCCTCCCC---------------GGCCTCCCGGCGCGC-CCCTCCCGGCCTGAGCGGCGCTGCGAGCTCGCCTGACTGCTGTGC-CGGGACT 93

Mus     GGGCTATTGTT-------------------GAGAGCAATGCCACTTGGGCCTGAAGGCAAAGCCTCAGGTTATAAGAGGGGAACTGCTAGTGATTAGACTGGGGTCCCAT 195
Rattus  GGGCGACTGTT--TAGAGCATGCCGACTTGGAAACCCATGCCACCTGGGCCTGAAGGCAAAGCCTCAGGTTATAAGAGGGGAACGGCTAGTGATTAGACTGGGGT-CCAT 182
Cavia   GGGCACCAGCCGCTGAAACAACATCTACTTGAGGCCTGGGCTCCTTAGGCCCTGAAGCAGAACCGCGGGGTGTAGGGAGGGAGGTAGGGCTGACAATAGTGGGGCTCATT 215
Homo    GGGCGGCAGCTGCTCGAGCAGTGCTTCCTTGAGGCCGCGGCTGCTTGGGCCGGGAGGGAGAGCCGCGGAGTATAGGAATGGGCGTAGGGGTGGCCACACGAGGGCCCTTC 203

Mus     CTGGTTTCTAGTTGTCTAGTCTTCTATTGGGAGCCCTCAGTGATGTCAGAAGCGGGCTTATTGTCTTTCTCCCACTGTGGACAAGACT----AGGCTGCCATTAACAAAT 301
Rattus  CTGATTTCTAATTGTCTAGTCTTCTACTGGGAGCCCTCAGTGATGGCAGGGGCAGGGTGA--AATTGTCTTTCCCTGTGGACAAAACT----AGGCTGCCATTAGCAAAT 286
Cavia   TAGG------------AGACCCTCTCCAGGAAGCCCTTAGTGGGAGC----------CACATATCTTTTTCCCCACGTGGAAGAAGCTGCCGAAGCTGCCATGGGCAAAC 303
Homo    TCGG-----------AGAACTTCTCCCGGGGAACCCTCGATGGGGGTGGGGGCA-----------TTCCTCCCTCTATGGACC---------AGGCTGCCATTGGCAAGC 282

Mus     GATCTTGGGAACTCTGGGCTGGGTAGAGTATAGGCCCGGGT--------------------------------------CAGTACCCAGGATTTCCACCAAACCTTCCC- 372
Rattus  GATCCTGGGAACTCGGGGCTGGGTAGAGTATAGGCCCAGTG--------------------------------------GCATACCCTGGATTTCCACCAAACCCTTCC- 357
Cavia   AATCCGGAGAACTCTGAGCTGGGTGGGGCTCGGGCCCCTGG---------------------CAGTCGCAGCCTCCAGCCAGGAGCCTGGATCTCCATCATACCCTTCCC 392
Homo    GATTTACAGAACGCGGGTCTGGGTAAGGCACGGGCCCCCTAGCAGCGAGGCCGGCCTGTCCCCTGCCCCGCCTTCCAGCCCAGAGCCAGGATCTGCATCATACCTTTCCG 392

Mus     TGCCCTTCACCCTCCACTACTCCT-GGCCCTGTTTCAG 409
Rattus  --CTGTTCACTCTCCACTACTCCT-GGCCCTGTTTCAG 392
Cavia   TGCCCTTCACCCTCTACTACCCCTCTACCATGTTTCAG 430
Homo    TGCCCTTCACCCTCCGCTGCCCCT--GCCCTGTTTCAG 428
```

ENSMUSG00000032841 intron 1

Description: Protor-2 (Prr5l)
Intron number: 1
Mouse chromosome: 2
Upstream exon length: 164
Downstream exon length: 81
Mouse intron length: 1114
Intron alignment length: 2072
Total murinae branch length: 0.15927
K_score: 0.0349
Scaling factor: 0.79687

ENSMUSG00000032841 exon 1 (ORF 0)

```
Mus     CCCGCGGCCTCGCTCCCCTCCTGCCCATTGAATTCCACAAGATGGGCTCCTTCCGCCGGCCTAGACCACGCTTTATGAGC 80
Rattus  CCCGCGGCCTTGCTCCCCTCCTGCCCATTGAGTTCCACAAGATGGGCTCCTTCCGCCGGCCTAGACCACGATTTATGAGC 80
Cavia   CCCGTGGCCTCGCTCCTACCCTGCTCACTGAGTTCCACACCATGGGCTCCTTCCGGAGGCCGAGACCGCGCTTCATGAGC 80
Homo    CCCGCGGCTTCGCTCCCATTCTGCCCGTCGAGTTCCACAAGATGGGCTCCTTCCGCAGGCCTAGACCGCGCTTCATGAGC 80

Mus     TCCCCTGTGCTCAGCGAGCTGCCCCGCTTCCAGGCTGCCCGGCAGGCTCTGCAGCTAAGTTCCAACTCTGCCTGGAACAG 160
Rattus  TCCCCTGTGCTCAGCGAGCTGCCCCGCTTTCAGGCTGCCCGGCAGGCTCTGCAACTAAGTTCCAACTCAGCCTGGAACAG 160
Cavia   TCCCCCGTGCTCAGCGACCTGCCCCGGTTCCAAGCTGCCCGGCAGGCTCTACAGTTGAGCTCCAACTCGGCCTGGAACAG 160
Homo    TCCCCCGTGCTCAGCGACCTTCCCCGATTCCAAGCAGCTCGGCAGGCTCTGCAGCTGAGCTCCAGCTCAGCCTGGAACAG 160
```

ENSMUSG00000032841 exon 2 (ORF 1)

```
Mus     TGTGCAGACAGCTGTGATCAACGTTTTCAAAGGGGGCGGCTTGCAGAGCAATGAGCTGTATGCACTGAACGAAAGCATCA 80
Rattus  TGTGCAGACGGCTGTGATCAACGTTTTCAAAGGGGGCGGCTTGCAGAGCAATGAGCTGTATGCACTGAACGAAAGCATCA 80
Cavia   TGTCCAGACTGCTGTGATCAACGTTTTCAAAGGGGGCGGCTTGCAAAGCAATGAGCTCTATGCACTGAATGAAAACATCA 80
Homo    CGTTCAGACTGCTGTGATCAACGTTTTCAAAGGGGGTGGCTTGCAAAGCAACGAGCTCTATGCCCTGAACGAAAACATCA 80

Mus     G 81
Rattus  G 81
Cavia   G 81
Homo    G 81
```

ENSMUSG00000032841_intron_1

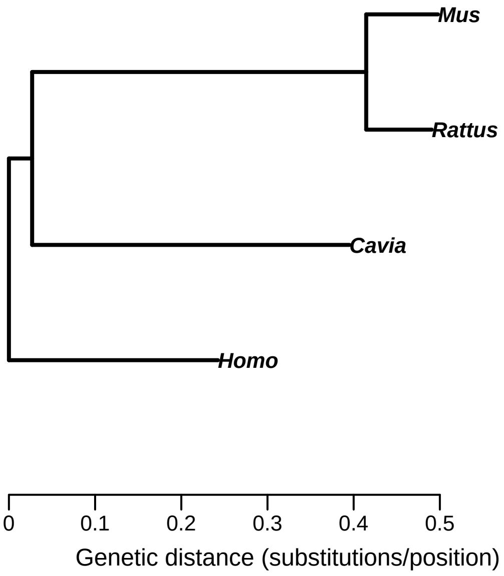

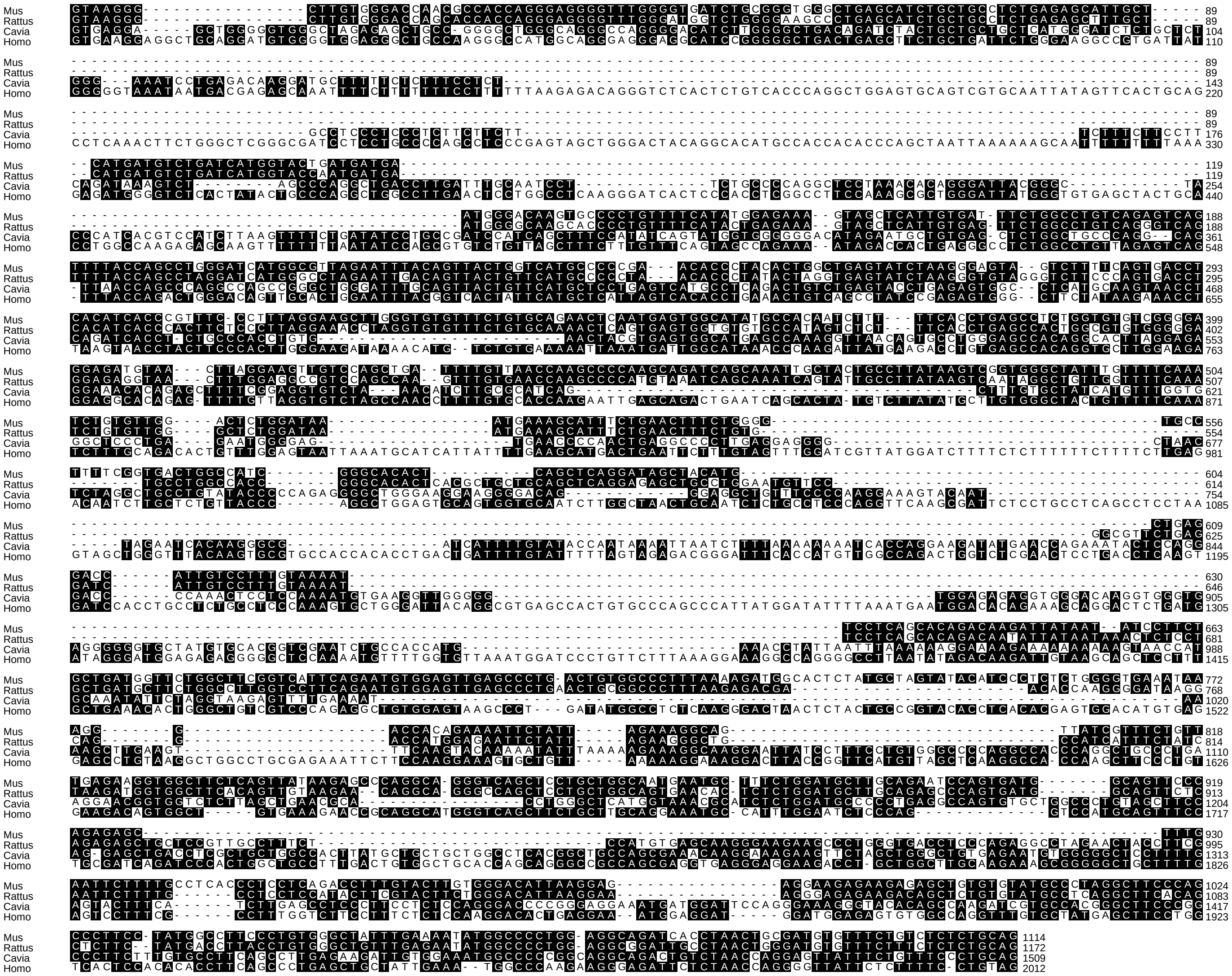
Mus
Rattus
Cavia
Homo

ENSMUSG00000045620 intron 2

Description: Outer dense fiber protein 3-like protein 1 (Odf3l1)
Intron number: 2
Mouse chromosome: 9
Upstream exon length: 111
Downstream exon length: 114
Mouse intron length: 672
Intron alignment length: 900
Total murinae branch length: 0.09451
K_score: 0.07879
Scaling factor: 0.79738

ENSMUSG00000045620 exon 2 (ORF 2)

```
Mus     GTCCAGGGCCTGCCAAGTACCTCCGGTCATCCTGTACTGGCTATATAGCCCATGACATCTCCATGTTCCAGGAGCCAGCT 80
Rattus  GTCCAGGGCCTGCCAAGTACCTGCGGCCATCTTGTACTGGCTACATAGCCCATGACATCTCCATGTTCCAGGAGCCAGCC 80
Cavia   GTCCGGGGCCCGCCAAGTACCTGCGGCCATCGTGCACGGGCTACGTGGCTCATGACAGCTCCATGGTCCGGGGGCCAGTG 80
Homo    GTCCGGGGCCCGCCAAGTACCTCCGGCCATCCTGCACGGGCTACATAGATCATGACATCTCCATGTTCAAGGCACCAGCT 80

Mus     TACAGTCTGCATACACGGCACACTAAGAAAC 111
Rattus  TTCAGTCTGCATACACGGCACACTAAGAAAC 111
Cavia   TTCACCTTGCACAAGAAGCACTCAGAGAAGC 111
Homo    TATACCCTGCATAGCCGGCACTCAGAGAAGC 111
```

ENSMUSG00000045620 exon 3 (ORF 2)

```
Mus     GGATCATAGACATAAACAGCCCCGGACCTTGCTATTTCTTGAATCCCAAAGTAACTCGTTTTGGAATATCCACCTGCCCC 80
Rattus  GGATCACTGACGAAAACAGCCCTGGACCTTGCTATTTTTTGGATCCCAAAGTAACTCGTTTTGGAATATCTACCTGTCCC 80
Cavia   GGATCACAGACATCTGTAGCCCAGGACCCTGCTATTGCTTGGATCCCAAAATAACCCGCTTTGGAATGTCCAGCTGCCCA 80
Homo    GGATGGTGTGCCACAGCAGCCCTGGGCCTTGCTATCTCTTGGATCCCAAAATAACTCGGTTTGGAATGTCCAGCTGCCCG 80

Mus     CAGGTCCCCATGGAGGAGCGCATCTCCAATCCAC 114
Rattus  CAGGTTCCCATGGAGGAGCGCATCCCCAACCTAC 114
Cavia   CAGGTGCCCATGGATGGCCGTGTCACCAGCCTGC 114
Homo    CAGGTCCCCATGGAGGAGCGCATCTCCAACCTGC 114
```

ENSMUSG00000045620_intron_2

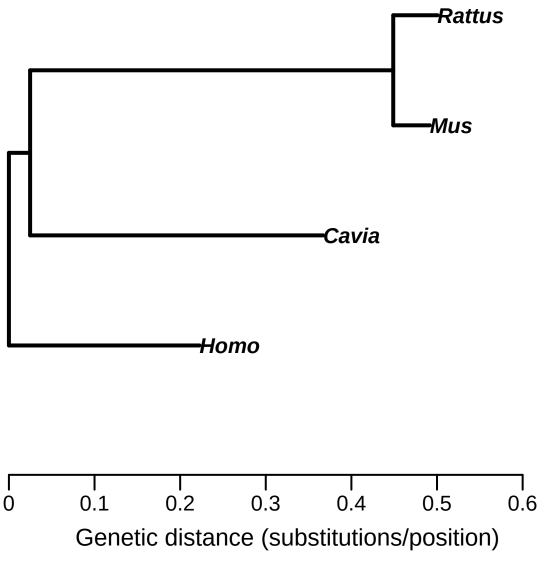

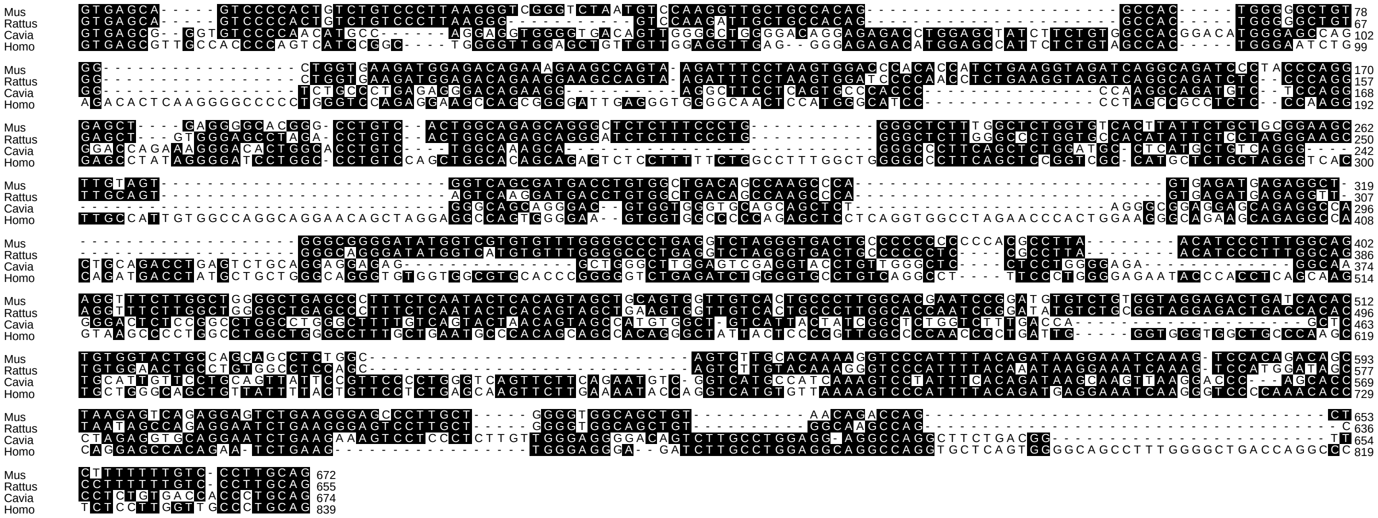

ENSMUSG00000052353 intron 3

Description: Protein KIAA1199 homolog Precursor (9930013L23Rik)
Intron number: 3
Mouse chromosome: 7
Upstream exon length: 139
Downstream exon length: 237
Mouse intron length: 1032
Intron alignment length: 1471
Total murinae branch length: 0.17192
K_score: 0.04942
Scaling factor: 0.79832

ENSMUSG00000052353 exon 3 (ORF 2)

```
Mus     GAAAGCTTGTCATTAAAGACCACCATGAGCACATTGTGCTGCGTACCCGGTACATCCTGATTGATGACGGTGGAGAGCTG 80
Rattus  GAAAGCTCGTCATTAAAGACCGCCATGAGCACATCGTGCTGCGCACGCGGCACATCCTGATTGATGATGGTGGAGAACTG 80
Cavia   GCAAGCTCATCATTAAGGACCACGAGGAGCCCATTGTTTTGCGCACCCGCCACATCCTGATTGACAGTGGAGGAGAGCTG 80
Homo    GCAAGCTGGTCATTAAAGACCACGACGAGCCGATTGTTTTGCGAACCCGGCACATCCTGATTGACAACGGAGGAGAGCTG 80

Mus     CATGCTGGGAGTGCCCTTTGCCCTTTTGAGGGCAATTTCAGTATTGTGCTGTATGGAAG 139
Rattus  CATGCTGGGAGTGCCCTCTGTCCTTTTGAGGGCAACTTCAGTATTGTGCTGTATGGAAG 139
Cavia   CATGCTGGGAGTGCCCTCTGCCCTTTCCAGGGCAATTTCAGCATCATTCTGTATGGAAG 139
Homo    CATGCTGGGAGTGCCCTCTGCCCTTTCCAGGGCAATTTCACCATCATTTTGTATGGAAG 139
```

ENSMUSG00000052353 exon 4 (ORF 1)

```
Mus     GGCTGATGAAAACATCCTGCCAGACCCTTACTATGGCCTGAAGTACATCGGAGTAGACAAAGGAGGCACTCTTGAATTAC 80
Rattus  AGCCGATGAAAGCATCCCGCCGGACCCTTACTATGGCCTGAAGTACATCGGAGTGGGCAAAGGAGGCACTCTTGAGTTGC 80
Cavia   AGCCGATGAAGACATCGAGCCAGACCCTTACTATGGGCTGAAGTACATCGGTGTGGACAGGGGTGGCACCTTGGAACTGC 80
Homo    GGCTGATGAAGGTATTCAGCCGGATCCTTACTATGGTCTGAAGTACATTGGGGTTGGTAAAGGAGGCGCTCTTGAGTTGC 80

Mus     ATGGGCAGAAAAAGCTTTCCTGGACTTTTCTAAACAAGACCCTTCATCCTGGTGGCATGCAGGAGGGAGGATATTTTTTT 160
Rattus  ATGGGCAGAAAAAGCTTTCCTGGACTTTTCTAAACAAGACCCTTCATCCGGGTGGCATGCAGGAGGGAGGATATTTTTTT 160
Cavia   ACGGCCAGAAGAAGCTCTCCTGGACATTTCTGAGCAAGACTCTCCACCCAGGGGGGATGGAGGAGGGAGGCTATTTTTTT 160
Homo    ATGGACAGAAAAAGCTCTCCTGGACATTTCTGAACAAGACCCTTCACCCAGGTGGCATGGCAGAAGGAGGCTATTTTTTT 160
```

ENSMUSG00000052353_intron_3

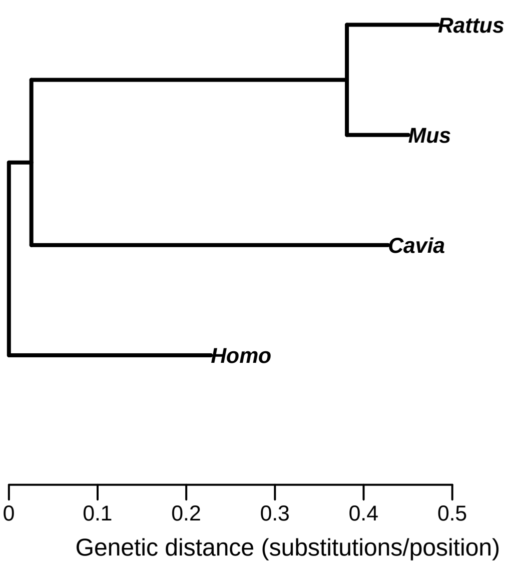

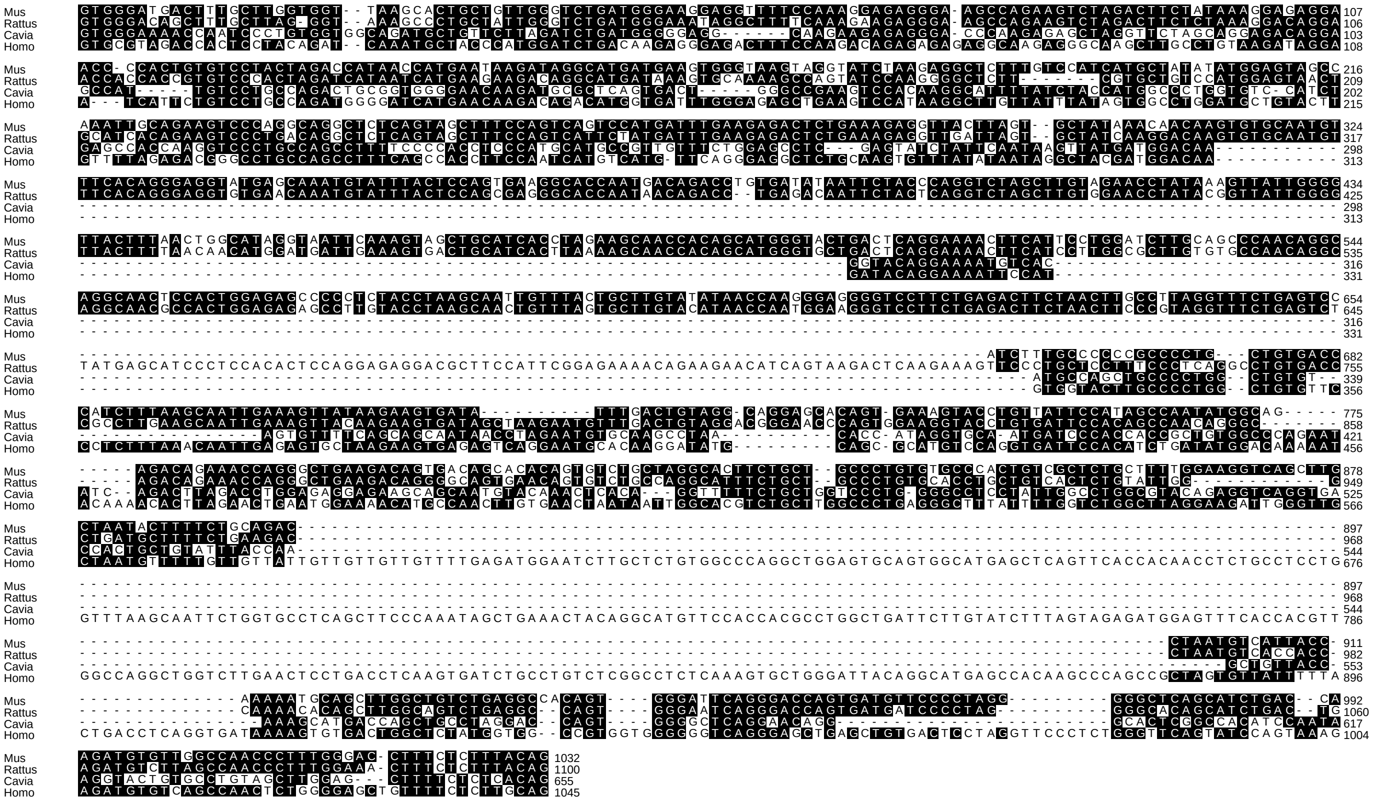
Mus
Rattus
Cavia
Homo

ENSMUSG00000030374 intron 3

Description: Striatin-4 (Strn4)
Intron number: 3
Mouse chromosome: 7
Upstream exon length: 74
Downstream exon length: 79
Mouse intron length: 1059
Intron alignment length: 2016
Total murinae branch length: 0.15625
K_score: 0.08623
Scaling factor: 0.79981

ENSMUSG00000030374 exon 3 (ORF 1)

```
Mus     GGCCAAATATCATAAACTGAAGTTTGGTACAGACCTGAATCAGGGGGAGAAGAAGACAGATCTGTCAGAACAAG 74
Rattus  GGCCAAATATCATAAACTGAAGTTTGGTACAGACCTGAATCAGGGGGAGAAGAAGGCAGATCTGTCAGAACAAG 74
Cavia   GGCCAAATATCATAAACTGAAGTTTGGGACAGACCTGAACCAGGGAGAGAAGAAACCAGATTTATCAGAACAAG 74
Homo    GGCCAAATATCATAAACTGAAGTTTGGGACAGACCTGAACCAGGGGGAGAAGAAAGCAGATGTGTCAGAACAAG 74
```

ENSMUSG00000030374 exon 4 (ORF 2)

```
Mus     TCTCCAATGGCCCTGTAGAGTCGGTCACACTGGAGAACAGCCCATTGGTGTGGAAGGAGGGTCGACAGCTTCTGCGACA 79
Rattus  TCTCCAATGGCCCTGTAGAGTCTGTCACACTGGACAACAGCCCATTGGTGTGGAAGGAGGGGCGACAGCTTCTGCGGCA 79
Cavia   TTCCCAATGGCCCTGTGGAGTCAGTCACCCTGGAGAGCAGCCCGCTGGTGTGGAAGGAGGGACGGCAGCTTCTCCGACA 79
Homo    TCTCCAATGGCCCCGTGGAATCGGTCACCCTGGAGAACAGCCCGTTGGTGTGGAAGGAGGGGCGGCAGCTTCTCCGACA 79
```

ENSMUSG00000030374_intron_3

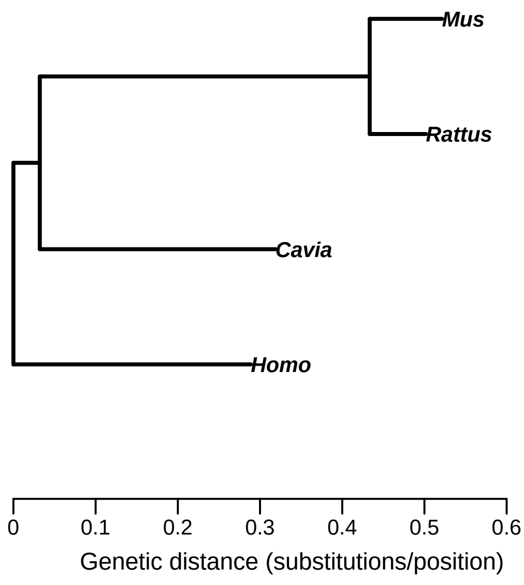

```
Mus     GTACCCAC-CCTTGCAACTTC---CTGCGGCCACCCCAGCAGAGCAGCTGCTTGGCATAGAG------------------------------------------AAGTTG 64
Rattus  GTACCCAC-CCTTGTGTTCTC---CCTCAGCCACCC---CCGGCAGGCTGCTTGGCATAAAA------------------------------------------AAGTTG 61
Cavia   GTACTCCCATCACGTCTGCTC------------------------AAGTTGCCAGGCTGGGG------------------------------------------------ 39
Homo    GTACCCAC-CCTCTGATGCTCGCACTGTCGGCACCCTCCCGCAGAAGCTGAGTGTTCTGGAGAAGCAGGAAGATGGGGTGCCTCTTTCTCACTTTGCTCCTTAGAAGGCA 109

Mus     CACATGGCCATAGGGTG----------GGGTAGAGTTGCAACCTCAGGGGCTCTTCCC-------ACCAGTTCTGTGTCAGACTGTGTCCAGCCTTACCTGTCTGCCAT- 156
Rattus  CAGATGGCCATGGGGGGTAGGGGAGGAGGATAGAGTTGTATCCTCAGGGTCTCTGCCC-------GCCAGTCCTGTGTTAGACTGTGTCCAGCCATACCTGTCTGCCAT- 163
Cavia   ---------------------------------CACTTTTTGTTTCTCTGCTC----TTGCCTGGCACTGTGCTGCCTTCTGTGGAGTGAGGGCCCTCTGGCCTC 107
Homo    GAATCGGCCCCGGGGCT----GGGGAGGGGCGGAATCTCACTCTCAGAGCCCTCCCTATGGGCTGCCAGGTCCCGTGCTGGACCCTGGGGGCCAGTCCCGCTTGGG--T- 212

Mus     ------------------------------CTTGCTGTCCTCCCAAG-------------GACATGATGGATGGCCCAGCATCTC-CTGCATCCTTTT-----GTTGTGA 217
Rattus  ------------------------------CCTGCTGTCCTCCCAAG-------------GACATGATAGCTGCCCCAGCAGCTC-CTGCATCCTCCT-----GTTAGGA 224
Cavia   TGGGGTGAAGGCCCTCTGAGGGATGAGTCCCTTCTGCACAGTGAGGTCCCTCTTGGGGGTGAGCTATCTGAGGCACTGGTAGCACATTGCCTCCCTCTCTGGGAGCCAGA 217
Homo    ------------------------------CCCCCTGTGCAACAGAG--------------------GCAGACACACGAGCAGCTCACGGGATCCCCTTT------GCGAGG 267

Mus     GAATGAGTGTA--ACTATTTATGGTGTGCTG----------TGGAAAGTCTAGGAAAAACTTCATTGAAGAGGTAGTGTTTGCACTGGGTTGTCATTCAGTCATTTACAC 315
Rattus  GAGGGAATGTA--ACTATGTATAGCGCGCTG----------TGGAGAGTGTAGGAAAAACGTCACTG-AGAGGTGGTGTTGGCACTGGGTTGTCATTCAGTCGTTTA--- 318
Cavia   GAAGGGAGCCA--GCTATTTATAGGGTGCTA----------TGAAAAGCTCA-GAAAGACTTCATTGAAGAGGCAGTGTTTGCACTGGGCTGTCATTCAATTATTTC--- 311
Homo    AGTGGGCGCCAGGGCTATTTATAGAGTGCTGGGGACGGTGCGGGAAAGCCGG-GGAAGACGTCACTGAGGAGGTGGCGTTTGAACAGGGTTGCCGTTCAATCGTTCA--- 373

Mus     TAACCCCCCCCCCCCACCTCAATTAATATATGAGGTGCTGGCTTCTTGTCATAGAGCAGGG-CACAGCAGAGAGCAGAATAGATGT-GTGCTTATTCTGTGGGCATTGGC 423
Rattus  ---------CCACCCCCTTCAAATTGATATGTGAGGTGCTGGCTTCCTGTCACAGAGCAGGG-CACAGCAGAGAGCAGAAGAGATGT-GTGCTTGTTCTCCGGGCACTGGC 418
Cavia   --------------TCCAAGGAAAGTTTGTGAGGTGCTGGCTTTATGTCATAGAACAAGGAAAGCAAGATAGGCAGGAG-------GTCTCTGCTCTCTGGGCCCTTGT 399
Homo    -----------TCCAAAAAGACTTTATTTGTAGGGTACAGGCTTTGTATCACAGATCAGGAATACAGCAGAGAGCACGGGAGACATGGTCTCTACCCTCTGGTCCCTTAG 473

Mus     A------GCTTAAGCTTCTGGAAGGAGGA-----------AACAGATGTCCATGGGAGTGCGGGAATGTTTTCGGGTGACACACTA---GTGTCTAGTCAGGTGGGCCCA 513
Rattus  A------GCTTAAGCTTCTGGAAGGAGAG-----------AACAGATGTCTTTGGGGGTGAGGGAATGTTTTCAGGTGAAGCACTA---GTGTCTAGTCAGGTGGACCCA 508
Cavia   AGGAAGGGCCTTCCTTCCTCACAGGAAGATTTCGTTCCTGGGCAGGGGTGAAGGTG-----------------------------------GCGTGTTGGGACAGGGAGTCA 477
Homo    A------GTCTAAGCTCCTTGAAAGATGACTCCGTTCCCCAGCAGGAGAGTCAGGGGAGAGAGCACAGATACAGAGACGCACGCTGAAAGGGTTAGGGGACAGGGAGTCG 577

Mus     GTG--------------------GCAAGGGCACCAGTGTGCACGGTTGTGCCAGTGATGTTTGGAGCTTGGTCCTGGGGCTCTGTGGAGTTCTTGCCTACAGGGCTAAGT 603
Rattus  GTG--------------------GCAAGGGCACCAGTGTTCGTGGTTTTGCCAGTGATGTTTGGAGCTTGGTCCCGGGGCTCTGTGGAGTTCTTGCCTGCGGGGCTAAGG 598
Cavia   GTT-GTGTGCCTGGAGCATTAGGTCAGGGCAGCATGCCCATCAGGCTGGCCTTGGTAACACCAAAGCTTGGCTGAACGGAGCAGTGGAGATCTGGCCTGCCAGGTTGAGA 586
Homo    GTCAGTGTGCCTGGGCAAACAGTGTAGGGCAGAGGATGTGGGAGGCGGGCTGGGTGAGGGGGAAGCTTGGCCGAGGCAAGCTGAGAAGACCTTGTGTGCTCAGCTGATA 687

Mus     GAGGTCTTCCTT--------TCATGCCCTGCTACTGAGATGTACGCTCTTGA------------------CTGGGATGGCAAAGGTACTCAGCCAGGCCTTATGGATAGA 687
Rattus  GAGGTCTTCATT--------TCATGCCATGCTTCTCCTACTTAAGCTCTTGA------------------CTGGGGTAGCCCAGGCATCCAGCCGGGACTTATGGATAGA 682
Cavia   GTGATTATTATTACCTTAGGTCAGATTCCTTCAACTATATGTAAGTTCCTCAAGGCAGG------------TCTGTGTAGCCCCAGACTCTAGTCAGATGCAGTG--CAGG 683
Homo    GTGATGGTCATTACCTGCTGCCAGCTCCTCTCACTAGAACGGAAGTTCCACAAGGGCAGGTCTTTGTTTACCCATGTAGCCCAGGCCCCTGGCCAGGCACAGCG--TAGA 795

Mus     ------------------TGAGCAATGCATAGAGTTAGCTTCCCAGCCAGCTC-------------------------ACCTATAGCCGCTACCCTCGGTTCTCTCCAG-- 753
Rattus  TGTCCAATGGATACCTCATGAGCAATGCACAGAGTGGGTTTCCCAGCCAAACTCACCTCA---GCCGGAGAGGTAGAATCTTTAGCTGCTACCTTCGGTTCTCAGCATGG 789
Cavia   TGCCCAATAAATACTTCATGAATAAAATACTGACCAGATTATCCAGCCTGACCTCCACAATAGTCTGAAGAGGTAGATGCTGTTAATATTCCCCTTACCAC-CAGCAGAG 792
Homo    TGCCCAGGAAAGACT---TGAATAAAATACTAAGCAAATTATCTCATTGCATCCTCTCAGCACTCCCAAGAGGGAGATGCTGTTATCATTAAGCTCATTTCTCAGAGGAC 902

Mus     ---------------------------------------------------------------------------------CCTGCTTGCTCATTCTCTAGAGCTAGACT 782
Rattus  GAAACAGCTGC-------------------------------------------TTCAGGAAGGGCTCAGCTGAGC-TGACTTCAGCCTGCTCATTTCTAGAGCCAAACT 856
Cavia   GAAGCTGAGGCTTAGGTAGGTGAGCTAACCTGCTTCCCCAGTCCCACAGAGGTGTGAAAAGTGGCAGAGCTGCTTTTGATGCCAGCTG--CCCATTCTCAGAACCAGGCT 900
Homo    GAAACTGACAC-----------GGCTTACACTAACTCAGGGTCACACAG----CTGGGATGGGGCAGAGCTGGGCTTGAACCCAGTCGGCCCTTCTCTCAGAACCAGGCC 997

Mus     ACCCACTCCTTGCATAAGAAAGCAGCAGCCAGCCGTGCATGTGGAGGCACAGCTCTGCTATTCTTGGCCGTGGTT--GGCAGGGGCCAGTCAAGAG-CCTAAC----AGG 885
Rattus  ACCCACTCCTTGCATAAGAAAGCAGCAGCCAGCCATGCATGTGGAGGCACAGCTCTGCTATTCTTAGCGGTGGTT--GGCAGGGGCCAGTCAAGAG-CCTAACAGTGAGG 963
Cavia   GGC----TTCTGGCTCGGAAAATAAAAGCCAGCCCAACCAGTGAAGGTAC----------TGGGTGGGTGTGGTT-TGGGGAGTGACAGTCAGGAGACCAGAGATGGAGA 995
Homo    AGC----TTTCTGGTTTTGAGATAACACCC--------------TTATCT----------TGTCGGGGGCCAGTTTGGGGAACCACCAGTCAGGAGACCTAGAGATGAGG 1079

Mus     AGAGCTTCCAGGAGAGGAGTCACTCGG----------------------------------------------------------------------------------- 912
Rattus  AGAGCTTCCAGAAGAGGAGTCACTTGG----------------------------------------------------------------------------------- 990
Cavia   AGTGTTTCTAGGAGAAGAATTGCTGAGAACACTGGGATGGTTGTGCATGCCTATAATCTCAGCACTCTGGAGCCTGAGACAGGAAGATCACCATGAGTTCAAGGCCAGCC 1105
Homo    AACGCTTCAAGGAGGAGGAATCCCCGAGTAA------------------------------------------------------------------------------- 1110

Mus     --------------------------------------------------------AGGGGGAGTGTGGGTGGAGGGGACTCAGGAAATGGGGACAGGC----CTGTGAT 962
Rattus  --------------------------------------------------------ACGGGGAGTGTGGGTGGAGAGCACTCGGAAAGTGAGGACAGGCCTATCTATGAT 1044
Cavia   TAAACTACATAATGAGACCCTATCTCAAAACCGTAAAAAAAAGGTCTTTTAAAACTGAAAACAGGGTGGGGTTAGGACTTTCAACAAGGGACACTGAGC--ACTGGTGTC 1213
Homo    --------------------------------------------------------GGGAAGAGAGCAGGGTTCCAACACTCAGGAAGTGATGCCACAG-GACCTGCGAC 1163

Mus     GGTGGCAGATTCATTCAGACTTGATATGAGAAGATGGAGGGGTGACTGGAGAGTGTCCATGCCCTGACT--GAGACATCTTCCTCTTCTCTCTTT---TTAG 1059
Rattus  GGTGGCAGATTCAGTTAGACTTGATGTGAGAAGATGGAGGGA-GACAGGAGAGTGTCTGTGCCCTGACT--GAGACAGCCTCCTCTTCTCTCTCT---CTAG 1140
Cavia   ACGGATGGATTTGGGCTAG---AGTGAGGAATGTGAGCAAGGTGACGGGTGGGTG--AGTAGCTGTCCTCTGAGATGGCTTCTTCCTTTCTCCCTGCACCAG 1310
Homo    AGTGACCGATTTGGGTTGGAGGGGTGAGGAAGATGGAGGAGGTGATGGGTAGGTGGCAGTGGCCTGACTTAAGACAGCCTCCGTTCTCTCACCCG---CCAG 1262
```

ENSMUSG00000022414 intron 10

Description: Mitogen-activated protein kinase kinase kinase 7-interacting (Tab1)
Intron number: 10
Mouse chromosome: 15
Upstream exon length: 157
Downstream exon length: 208
Mouse intron length: 1166
Intron alignment length: 2063
Total murinae branch length: 0.16625
K_score: 0.06642
Scaling factor: 0.79985

ENSMUSG00000022414 exon 10 (ORF 2)

```
Mus     ---GGGGCCGTGTGTACCCTGTTTCTGTGCCCTACTCAAGTGCCCAGAGCACCAGCAAGACTAGTGTGACTCTGTCCCTC 77
Rattus  ---GGGGCCGTGTGTACCCTGTTTCTGTGCCCTACTCAAGTGCCCAGAGCACCAGCAAGACCAGTGTGACTCTGTCCCTC 77
Cavia   CTGGGGGCCGAGTGTACCCCGTGTCCGTGCCTTATTCAAGCGCCCAGAGCACCAGCAAGACCAGTGTGACCCTCTCCCTC 80
Homo    CAGGAGGACGAGTGTACCCTGTGTCTGTGCCATACTCCAGCGCCCAGAGCACCAGCAAGACCAGCGTGACCCTCTCCCTT 80

Mus     GTCATGCCTTCTCAGGGCCAGATGGTCAACGGCTCTCACAGTGCCTCCACTCTGGACGAAGCCACTCCCACACTCACTAA 157
Rattus  GTCATGCCTTCTCAGGGCCAGATGGTCAACGGCTCTCACAGTGCCTCCACCTTGGATGAAGCCACTCCTACTCTCACTAA 157
Cavia   GTCATGCCCTCCCAGGGCCAGATGGTCAATGGCGCCCATAGTGCCTCCACCCTGGACGAAGCCACCCCCACCCTCACCAA 160
Homo    GTCATGCCCTCCCAGGGCCAGATGGTCAACGGGGCTCACAGTGCTTCCACCCTGGACGAAGCCACCCCCACCCTCACCAA 160
```

ENSMUSG00000022414 exon 11 (ORF 1)

```
Mus     CCAGAGCCCCACTCTGACCCTGCAGTCCACCAACACGCACACCCAGAGCAGCAGCTCCAGCTCTGACGGGGGCCTCTTCC 80
Rattus  CCAGAGCCCCACTCTGACCCTGCAGTCTACCAACACCCACACCCAGAGCAGCAGCTCCAGCTCGGACGGGGGCCTCTTCC 80
Cavia   CCAGAGCCCCACGCTGACCCTGCAGTCCACCAACACGCACACCCAGAGCAGCAGCTCCAGCTCCGACGGGGGCCTCTTCC 80
Homo    CCAAAGCCCGACCTTAACCCTGCAGTCCACCAACACGCACACGCAGAGCAGCAGCTCCAGCTCTGACGGAGGCCTCTTCC 80

Mus     GCTCCAGACCGGCTCACTCACTTCCACCCGGAGAGGATGGCCGTGTGGAGCCCTATGTGGACTTTGCTGAGTTCTACCGA 160
Rattus  GCTCCAGACCGGCTCACTCACTTCCACCTGGAGAGGATGGCAGGGTCGAGCCCTATGTGGACTTTGCTGAGTTTTACCGA 160
Cavia   GTGCCCGGCCGGCCCACTCACTCCCGCCTGGTGAGGACGGCCGTGTGGAGCCCTACGTGGACTTCGCGGAGTTCTACCGC 160
Homo    GCTCCCGGCCCGCCCACTCGCTCCCGCCTGGCGAGGACGGTCGTGTTGAGCCCTATGTGGACTTTGCTGAGTTTTACCGC 160
```

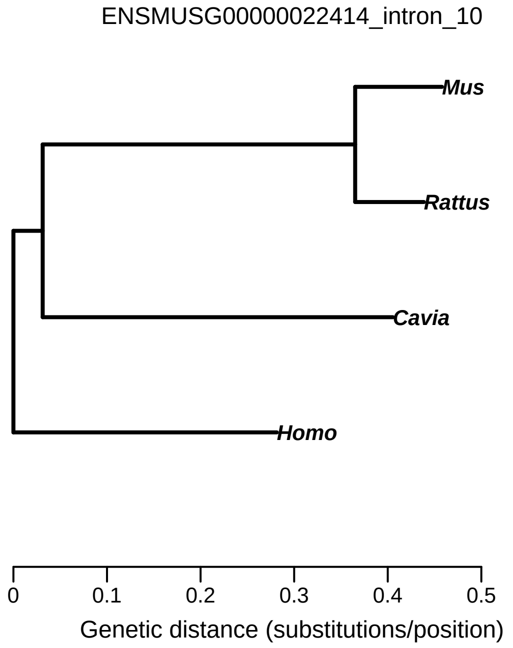

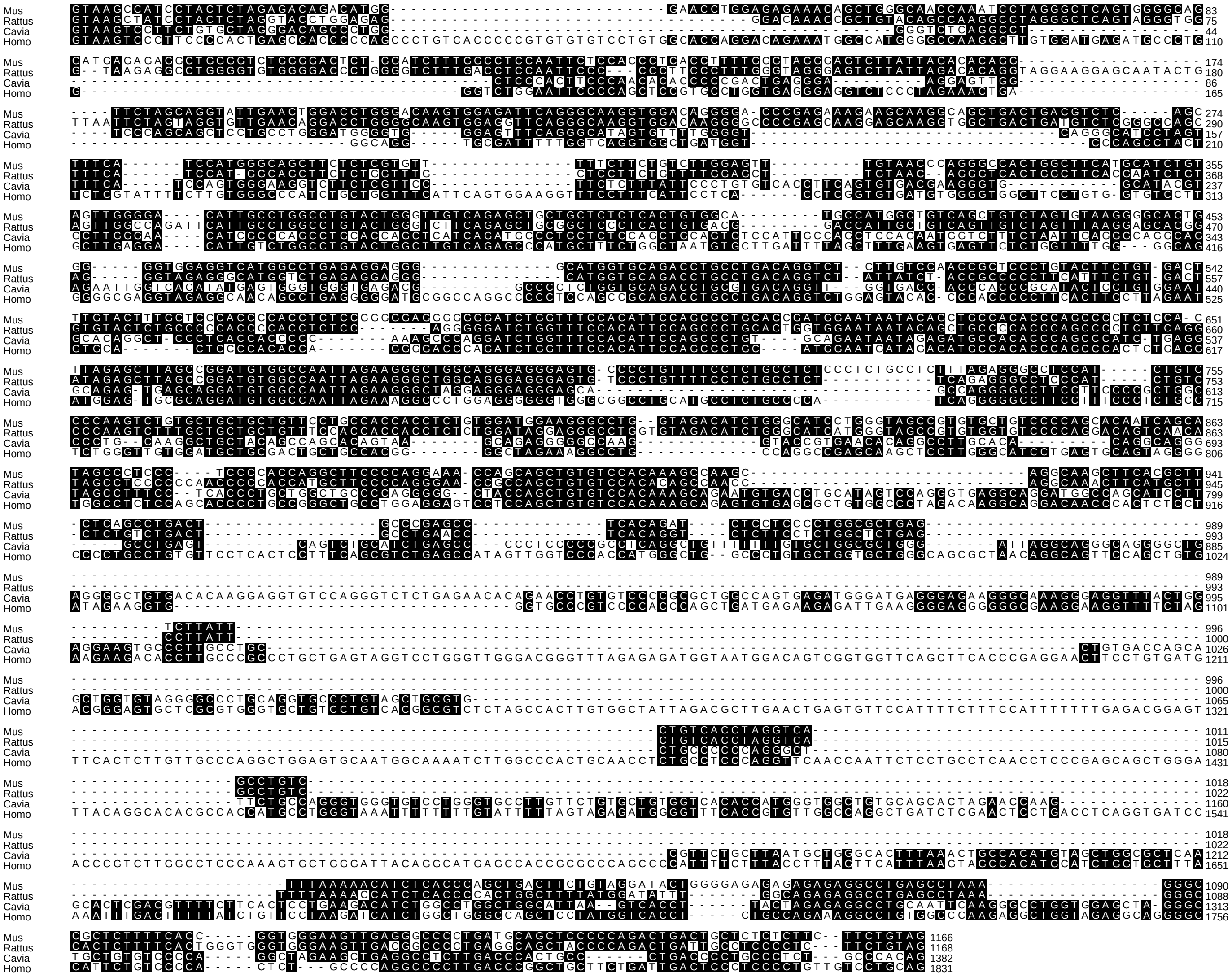
Mus
Rattus
Cavia
Homo

ENSMUSG00000023084 intron 13

Description: Leucine-rich repeat-containing protein C10orf92 homolog (4933430H15Rik)
Intron number: 13
Mouse chromosome: 3
Upstream exon length: 115
Downstream exon length: 122
Mouse intron length: 361
Intron alignment length: 1500
Total murinae branch length: 0.12345
K_score: 0.08336
Scaling factor: 0.79987

ENSMUSG00000023084 exon 13 (ORF 0)

```
Mus     CAGCTGGTTGTTGAAGCTACAGAGATGGTTAACCCTCTCCTGGAGCCCGTGGAACACCGAGATGGGAAGGTCTTCTTGCC 80
Rattus  CAGCTGGTTGTTGAAGCTACAGAGATGGTTAACCCTCTCCTGGAGCCCGTGGAACACCGAGATGGGAAGGTCTTCTTGCC 80
Cavia   CAGCTAGTTGCAGAAACTACAGAGATGATCAACCCTCTCCTGGAGCCAATGGAGCACCGAGATGGGAAGGTTTTCATGCC 80
Homo    ---CTGGTTGTTGAGGCTACTGAGGTGGTCAACCCTCTCCTGGAGCCTGTGGAGCACCGAGATGGGAAAGTTTTCATGCC 77

Mus     TGGGAACAAGGTCCTTTTGCACCTCAACCTCCTCC 115
Rattus  TGGGAACAAGGTCCTCTTGCACCTCAACCTCCTCC 115
Cavia   TGGGAACAAGGTCCTTTTGTACCTCAACCTCGTCC 115
Homo    TGGGAACAAGGTCCTTTTGCACCTCAACCTCATCC 112
```

ENSMUSG00000023084 exon 14 (ORF 2)

```
Mus     GAAACCAAATCACAGAGGTGGGGCTGGAAGGCTTCCTCACTGCTGTGCAATACCAGGTTCAGGTCTCCAAGCCCAAGACT 80
Rattus  GAAACCAAATCACAGAGGTGGGGCTGGAAGGCTTCCTCACCGCTGTGCAATACCAGGTTCAGGTCTCCAAGTCCAAGACT 80
Cavia   GAAACCGCATTACAGAGGTAGGGCTGGAGGGCTTCCTCACAGCAGTGCAGTACCAGGCACAGTTCTCCAAGCCCAAGAGT 80
Homo    GGAACCGCATCACAGAGGTGGGGCTGGAGGGCTTCCTCGCCACGGTGCAGTATCAGATGCAGTTCTCCAAGGCCAAGAGT 80

Mus     TCACCCAAGGCCCCCCTGGGTCTGCTGTGGCTGTCCTTGGCG 122
Rattus  TCACCCAAGGCCCCCCTGGGTCTGCTGTGGCTGTCCTTGGCA 122
Cavia   GCATCCAAGGGCCCAGTGGGGCTGCTGTGGCTGTCCCTGGCG 122
Homo    GCATCCAAGGGTCCAGTGGGGCTGCTGTGGCTGTCCCTGGCT 122
```

ENSMUSG00000023084_intron_13

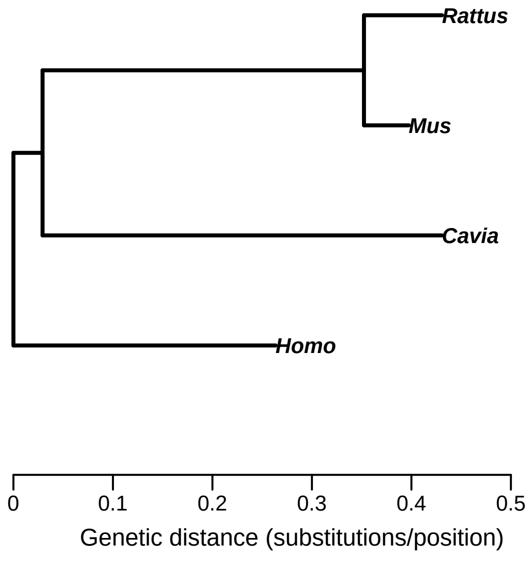

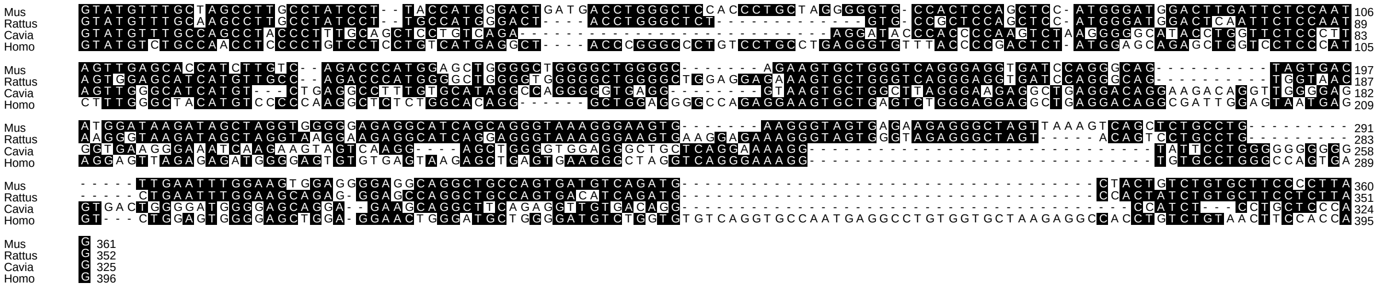
Mus 106
Rattus 89
Cavia 83
Homo 105
Mus 197
Rattus 187
Cavia 182
Homo 209
Mus 291
Rattus 283
Cavia 258
Homo 289
Mus 360
Rattus 351
Cavia 324
Homo 395
Mus 361
Rattus 352
Cavia 325
Homo 396
